# Comparative Mapping of the Wild Perennial *Glycine latifolia* and Soybean (*G. max*) Reveals Extensive Chromosome Rearrangements in the Genus *Glycine*

**DOI:** 10.1371/journal.pone.0099427

**Published:** 2014-06-17

**Authors:** Sungyul Chang, Carrie S. Thurber, Patrick J. Brown, Glen L. Hartman, Kris N. Lambert, Leslie L. Domier

**Affiliations:** 1 Department of Crop Sciences, University of Illinois, Urbana, Illinois, United States of America; 2 United States Department of Agriculture, Agricultural Research Service, Urbana, Illinois, United States of America; The University of Western Australia, Australia

## Abstract

Soybean (*Glycine max* L. Mer.), like many cultivated crops, has a relatively narrow genetic base and lacks diversity for some economically important traits. *Glycine latifolia* (Benth.) Newell & Hymowitz, one of the 26 perennial wild *Glycine* species related to soybean in the subgenus *Glycine* Willd., shows high levels of resistance to multiple soybean pathogens and pests including *Alfalfa mosaic virus*, *Heterodera glycines* Ichinohe and *Sclerotinia sclerotiorum* (Lib.) de Bary. However, limited information is available on the genomes of these perennial *Glycine* species. To generate molecular resources for gene mapping and identification, high-density linkage maps were constructed for *G. latifolia* using single nucleotide polymorphism (SNP) markers generated by genotyping by sequencing and evaluated in an F_2_ population and confirmed in an F_5_ population. In each population, greater than 2,300 SNP markers were selected for analysis and segregated to form 20 large linkage groups. Marker orders were similar in the F_2_ and F_5_ populations. The relationships between *G. latifolia* linkage groups and *G. max* and common bean (*Phaseolus vulgaris* L.) chromosomes were examined by aligning SNP-containing sequences from *G. latifolia* to the genome sequences of *G. max* and *P. vulgaris*. Twelve of the 20 *G. latifolia* linkage groups were nearly collinear with *G. max* chromosomes. The remaining eight *G. latifolia* linkage groups appeared to be products of multiple interchromosomal translocations relative to *G. max*. Large syntenic blocks also were observed between *G. latifolia* and *P. vulgaris*. These experiments are the first to compare genome organizations among annual and perennial *Glycine* species and common bean. The development of molecular resources for species closely related to *G. max* provides information into the evolution of genomes within the genus *Glycine* and tools to identify genes within perennial wild relatives of cultivated soybean that could be beneficial to soybean production.

## Introduction

Soybean (*Glycine max* L. Mer.) is a major source of dietary protein and oil in animal production and for human consumption worldwide [1]. With increasing utilization of soybean for animal feed in countries like China, there is added demand for soybean production [2]. Most of the increased demand for soybean products has been met by expanding the land area devoted to soybean production [3]. However, it is not clear if the expansion of soybean production areas alone will be able to keep pace with this growing demand. In addition, the global movement of soybean pathogens and pests and the emergence of new pathogens, as illustrated by the recent identification of soybean aphids (*Aphis glycines* Matsumura), soybean rust (*Phakopsora pachyrhizi* Syd. & P. Syd) and Soybean vein necrosis virus in North America [4]–[6], necessitates the identification of novel genes that will enable producers to meet the ever increasing demand for soybean production in the face of changing abiotic and biotic stresses.

Because of its narrow genetic base, soybean, like many cultivated crops, lacks diversity found in some of its wild relatives. The genus *Glycine* consists of 28 species split between two subgenera, *Glycine* Willd. and *Soja* (Moench) F. J. Hermann. The subgenus *Soja* contains two annual species, *G. max*, the domesticated species in the genus, and *G. soja* Sieb. & Zucc., both of which are native to Asia. The *Glycine* subgenus contains 26 species, including *G. latifolia* (Benth.) Newell & Hymowitz, that are native to Australia and surrounding islands, and have been shown to possess genes for agronomically valuable traits, such as resistance to *Heterodera glycines* Ichinohe and tolerance to *Sclerotinia sclerotiorum* (Lib.) de Bary. and drought [7]–[13].

To date however, it has not been possible to utilize genes from the perennial *Glycine* species for soybean improvement even though *G. max* and the perennial *Glycine* species share a relatively recent whole genome duplication that occurred between 5 and 13 million years ago [14], [15]. Attempts to hybridize *G. max* and *Glycine* perennials have, with the exception of *G. tomentella* (2n = 78) Hayata, been unsuccessful, even with *in vitro* embryo rescue [16], [17]. Cytogenetic observations have shown aberrant chromosome paring in F_1_ hybrids leading to embryo abortion [18]. Sequence data from *G. latifolia* and *G. tomentella* suggest that differences in intergenic and pericentromeric sequences, including sequences of widely dispersed retrotransposons, have reduced chromosomal pairing during hybridization [19]–[21].

Advances in molecular biology provide tools to circumvent the genetic barriers to capturing the biological diversity present in the perennial relatives of *G. max*. In addition to the genome sequence of *G. max* [15], the genome sequence of the wild annual species *G. soja* was recently determined [22], and shed light on similarities and differences between the two interfertile species. Using the genomic information, high-throughput sequencing and virus-based gene silencing techniques, multiple genes have been identified in soybean [23]–[26]. Even though gene mapping resources developed for *G. max* have not been directly useful in perennial *Glycine* species, the identification of large syntenic blocks between *G. max* and other legume species [27]–[30] suggests that high levels of synteny will be observed between annual and perennial *Glycine* species. The development of methods for cost-efficient discovery and mapping of single nucleotide polymorphism (SNP) markers through methods like genotyping by sequencing (GBS) [31], [32] have made it possible to fine map genes in plant species for which genetic resources are lacking, as is the case with the perennial *Glycine* species. Here, we describe the construction of high-density linkage maps for a perennial relative, *G. latifolia*, of cultivated soybean and compare the orders of mapped SNP markers to their positions in the genome sequences of *G. max* and *Phaseolus vulgaris* L.

## Materials and Methods

### Plant materials

Reciprocal crosses were performed between *G. latifolia* plant introduction (PI) 559298 and PI 559300 (obtained from the USDA Soybean Germplasm Collection in Urbana, Illinois; http://www.ars-grin.gov/npgs/urbana.html) as previously described [19]. Populations were advanced by selfing the F_2_ generation to the F_5_ generation.

### GBS mapping

DNA was extracted from leaf tissue of PI 559298, PI 559300, 146 F_2_ plants and 89 F_5_ plants using a DNeasy Plant Mini Kit (Qiagen, Valencia, CA). DNA samples were digested with *Bfa*I and *Pst*I-HF restriction enzymes (New England Biolabs, Ipswich, MA) as described by Thurber *et al.* [33]. For these experiments, *Bfa*I was selected because it did not produce strong banding patterns in preliminary restriction enzyme digestions of *G. latifolia* DNA and *Pst*I was selected because *G. latifolia* sequence data [19] contained an intermediate number of *Pst*I recognition sites. For example, the previously determined *G. latifolia* sequence data were predicted to contain 2.1×10^4^ *Mlu*I sites, 8.9×10^4^ *Pst*I sites and 3.1×10^5^ *Hind*III sites. Up to 96 samples were sequenced per lane of a HiSeq2000 (Illumina Inc., San Diego, CA) at the W. M. Keck Center at the University of Illinois, Urbana, IL, USA to produced 100-nt single-end reads. In both experiments, DNA from each of the parental lines was independently processed twice to serve as a control for SNP identification. The barcode splitter from TASSEL [34] was used to assign sequence reads to individual lines and remove barcode sequences, which produced 90-nucleotide sequence reads that were analyzed for SNPs. The parsed sequence data for the F_2_ and F_5_ populations have been deposited in the NCBI Short Read Archive as part of project SRP013346. Next, three Perl scripts were used to analyze the sequence reads for the bi-parental populations. First, sequence reads for each individual/line in the F_2_ and F_5_ populations and from the parental lines, PI 559298 and PI 559300, were aligned using Bowtie [35] to a *G. latifolia* pseudo-reference sequence, which was generated by sequencing 180-bp, 500-bp paired-end and 3-kb, 8-kb, and 15-kb mate-pair libraries prepared from *G. latifolia* PI 559298 DNA, sequenced on an Illumina HiSeq 2000, and *de novo* assembled using ALLPATHS-LG [36] (Chang et al., manuscript in preparation). The resulting assembly contained 16,423 scaffolds representing 1,069 Mbp, with an N50 of 235 Kb. Bowtie2 [37], which allows for insertions and deletions (indels), was also evaluated for read mapping, but at the high stringencies for matching employed, few indels were detected and the output from Bowtie was parsed more directly to SNP calls than output from Bowtie2. Second, SNPs were called when at least three reads from both replications of PI 559298 differed from both replications of PI 559300. Finally, Bowtie output files for each individual/line were used to assess allelic frequencies for each SNP using a custom Perl script, which ignored SNPs in low quality sequence reads (average quality scores of 40 or less). Based on allelic frequencies at each locus for each line, the Perl script then created a genotype matrix file for linkage analysis. Markers with less than 30% missing data and whose segregation did not differ significantly (*P*>0.05) from expected segregation ratios were selected for *de novo* linkage map construction.

Linkage maps were constructed using MSTMap [38] with a *P*-value  =  1.0^−9^, and visualized using MapDraw [39]. Consensus linkage maps were constructed for *G. latifolia* from the F_2_ and F_5_ data using MergeMap [40]. A weight of 5.0 was assigned to the F_5_ linkage maps and a weight of 1.0 to F_2_ linkage maps to reflect the higher confidence in the quality of the maps because of the reduced potential for errors in calling of heterozygous genotypes in the F_5_ population relative to the F_2_ population. To assess the synteny between *G. latifolia* linkage groups (LGs) and *G. max* chromosomes, SNP-containing sequences from *G. latifolia* were aligned to the *G. max* genome sequence [15] using BLAST [41]. For comparisons with *P. vulgaris* chromosomes, *G. latifolia* SNP-containing sequences and *G. max* gene models [15] were aligned to *P. vulgaris* chromosomes (http://www.phytozome.net/commonbean.php) using BLAST and visualized with MizBee [42]. When *G. latifolia* sequences aligned at more than one location, the most syntenic location was chosen for these analyses.

## Results

### Mapping GBS SNP markers in *G. latifolia* populations

**Figure 1:**
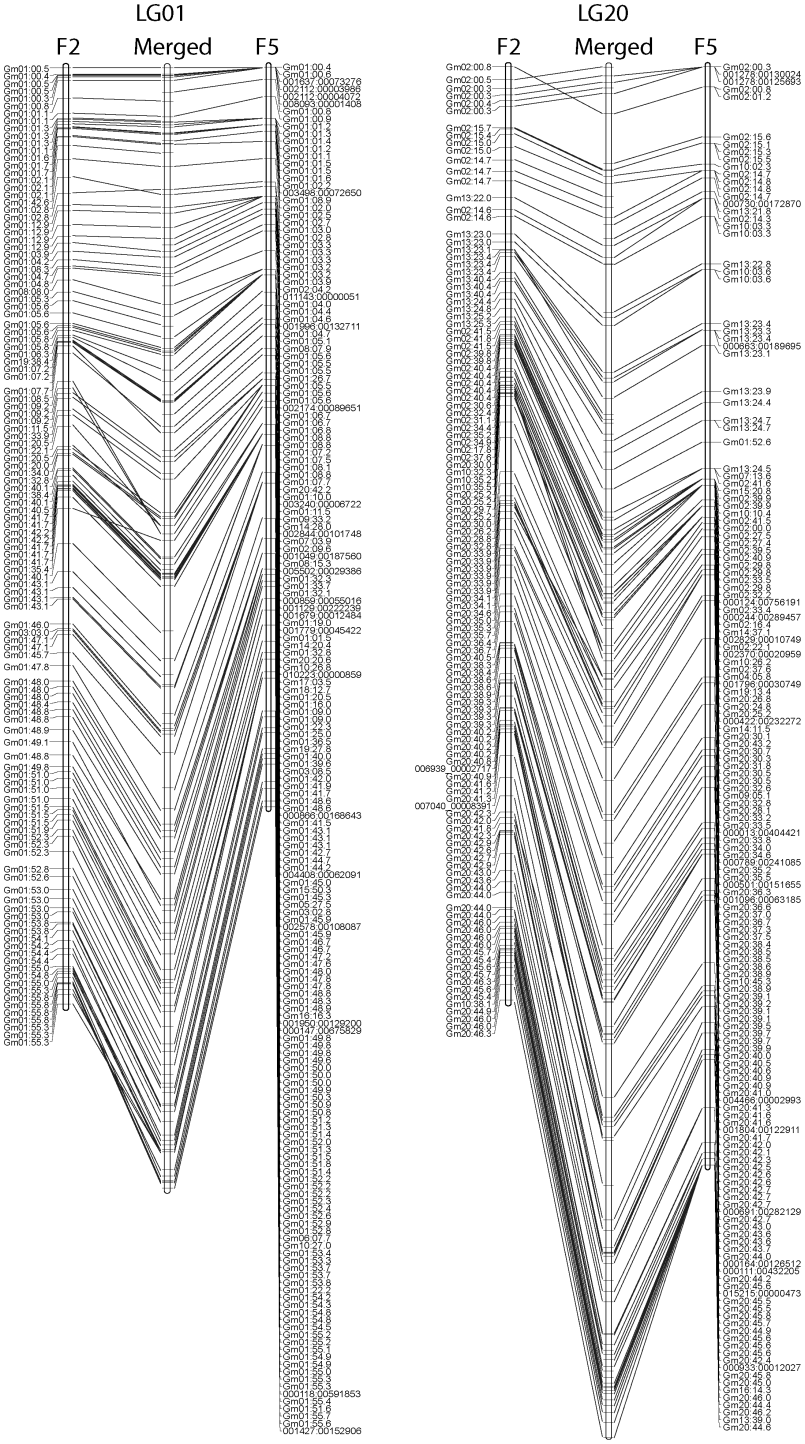
Comparison of F_2_, F_5_ and merged linkage maps for GBS SNP markers for *Glycine latifolia* linkage groups 1 and 20. Orders of SNP markers were very similar between the F_2_ and F_5_ populations. In some cases, markers that segregated in the F_2_ population co-localized in the F_5_ population, which may have resulted from errors in calling heterozygous loci in the F_2_ population. While linkage group 1 showed a high level of collinearity with *G. max* chromosome 1, linkage group 20 had regions of collinearity with multiple *G. max* chromosomes. Even so, there was good agreement in marker order between the F_2_ and F_5_ populations for linkage group 20. Markers were named for the *G. max* chromosome and the nucleotide position on the chromosome (×10^−6^) to which the SNP-containing sequences aligned. Markers that did not align to a *G. max* chromosome were named for the *G. latifolia* scaffold containing the SNP.

Genotyping by sequencing of the F_2_ population produced a total of 4.00×10^8^ 100-nt reads, of which 1.70×10^8^ passed all quality controls and uniquely aligned to PI 559300 sequences. After barcodes were removed, 90 nt were used for SNP discovery. In the F_2_ population, 5,160 markers could be reliably scored between the parental lines PI 559298 and PI 559300. Linkage maps constructed from that initial data set represented over 13,000 centimorgans (cM), which was significantly larger than *G. max* (2,296 to 2,550 cM) and previous *G. latifolia* (1972 cM) linkage maps [19], [43]–[46] and likely resulted from errors in calling heterozygous genotypes because of low coverage at some loci. The data set was reprocessed to exclude markers with more than 30% missing data and with segregation ratios that differed significantly from 1∶2∶1 (*P*>0.05), which resulted in 2,377 markers (Table S1). The average depth of coverage for the selected SNPs was 32 reads per locus and ranged from 0 to 270 reads. The markers formed 20 large LGs (Figure S1), with an average of 119 markers per LG (Table 1), and a total length of 2,305 cM. To confirm marker orders, an F_5_ population was analyzed by the same procedures. The analysis produced a total of 1.92×10^8^ 100-nt reads, of which 1.05×10^8^ passed all quality controls and uniquely aligned to PI 559300 sequences. The data produced 7,081 SNPs between the parental lines, from which 3,110 GBS markers (Table S2) were selected using similar criteria and analyzed in an F_5_ population. Average depth of coverage for the selected SNPs was 21 reads and ranged from 0 to 264 reads. As with the F_2_ population, most of the markers formed 20 large LGs (Figure S2), with an average of 155 markers per LG and a total map length of 3,110 cM. A total of 1,777 markers were shared between the two populations with 600 markers unique to the F_2_ population and 1,333 markers unique to the F_5_ population. The orders of shared markers were very similar in linkage maps constructed from the F_2_ and F_5_ populations (Figure 1). In some cases, markers that appeared to segregate in the F_2_ population did not segregate in the F_5_, presumably caused by errors in calling heterozygous loci in the F_2_ population. The shared markers were used as a framework to construct consensus linkage maps for *G. latifolia* (Figures 1 & S3). The merged consensus maps contained 3,710 markers (Table 1).

**Table 1:** Comparison of the numbers of SNP markers in F_2_, F_5_, merged and Chang *et al*. [19] maps.

	Markers				Length (cM)			
Linkage Group	F_2_	F_5_	Merged	Chang *et al*. [19]	F_2_	F_5_	Merged	Chang *et al*. [19]
1	123	185	206	17	116.7	92.2	139.8	117.44
2	152	192	229	15	142.6	162.6	220.4	196.71
3	87	127	157	10	91.4	103.6	137.7	46.96
4	107	138	158	13	121.0	95.8	153.4	124.51
5	139	155	202	8	137.5	94.3	180.3	62.18
6	157	207	240	13	159.5	119.4	183.1	112.52
7	80	128	144	15	85.4	93.8	129.7	93.96
8	150	175	214	13	129.9	141.8	175.2	151.24
9	126	175	201	14	129.6	118.4	153.7	94.25
10	102	149	174	11	112.7	132.0	171.7	98.99
11	106	128	164	13	114.4	104.0	132.3	103.77
12	94	103	130	16	91.9	100.9	118.4	116.93
13	115	169	193	15	96.9	94.0	130.6	156.41
14	85	75	125	12	79.1	39.5	95.8	51.73
15	114	164	185	12	104.4	98.3	125.9	103.64
16	119	155	182	9	102.8	95.9	144.7	64.38
17	136	167	202	11	126.9	90.6	147.5	67.76
18	130	177	199	19	124.4	98.8	156.1	90.93
19	131	179	214	11	121.8	94.4	147.7	44.11
20	124	162	191	11	116.3	136.5	170.3	73.8
Total	2377	3110	3710	258	2305.2	2106.8	3014.3	1972.2

### Synteny between *G. latifolia* linkage groups and *G. max* chromosomes

**Figure 2:**
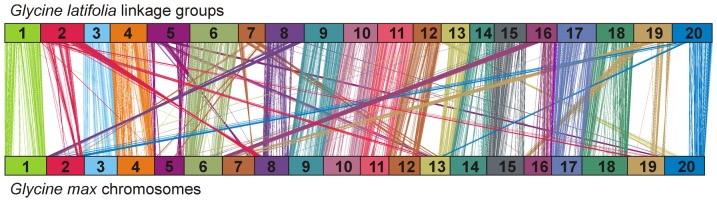
Synteny between *Glycine latifolia* linkage groups and *G. max* chromosomes. Sequences containing mapped *G. latifolia* SNPs were aligned to the *G. max* genome sequence. *Glycine latifolia* linkage groups (top) and physical maps for each *G. max* chromosome (bottom) are displayed as linear arrays. Vertical and diagonal lines connect genetic and physical locations of SNP markers between the two species.

Because little information is available on *G. latifolia* chromosomes, or chromosomes of any other perennial *Glycine* species, *G. latifolia* LGs were named for the *G. max* chromosomes to which *G. latifolia* SNP-containing sequences predominantly aligned. When mapped orders of *G. latifolia* SNPs were compared to positions of their sequences in the *G. max* genome, *G. latifolia* LGs 1, 3, 4, 6, 9, 10, 11, 12, 14, 15, 17, and 18 showed a high degree of collinearity with the corresponding *G. max* chromosomes and no interchromosomal rearrangements (Figure 2). In contrast, the remaining eight chromosomes each had at least one interchromosomal rearrangement relative to *G. max* (LG 13: one rearrangement; LG16: two rearrangements; LGs 2, 5, and 8: three rearrangements; LGs 7 and 20: four rearrangements and LG 19: five rearrangements). For example, *G. latifolia* LG 13 contained regions syntenic with *G. max* chromosomes 13 and 20 and *G. latifolia* LG 2 contained regions syntenic to *G. max* chromosomes 1, 2, 8, 13 and 19 (Figure 2). *Glycine latifolia* chromosome 8 appeared to be the product of two translocations between *G. max* chromosomes 2 and 8. Similarly, *G. latifolia* LG 20 appeared to contain a reciprocal translocation between *G. max* chromosomes 2 and 20. Syntenic blocks in rearranged LGs corresponded to between 0.3 Mb and 30 Mb (between *G. latifolia* LG 8 and *G. max* chromosome 8) in the *G. max* genome with an average of 6.9 Mb. Even though we described the structure of *G. latifolia* linkage groups as products for rearrangements relative to *G. max*, the structures of the ancestral chromosomes is not known. Hence, in some cases, *G. latifolia* linkage groups may have under gone fewer rearrangements than *G. max* chromosomes. Singleton markers (single *G. latifolia* SNP markers that aligned to a *G. max* chromosome without at least a second proximal collinear marker) were ignored for these analyses.

**Figure 3:**
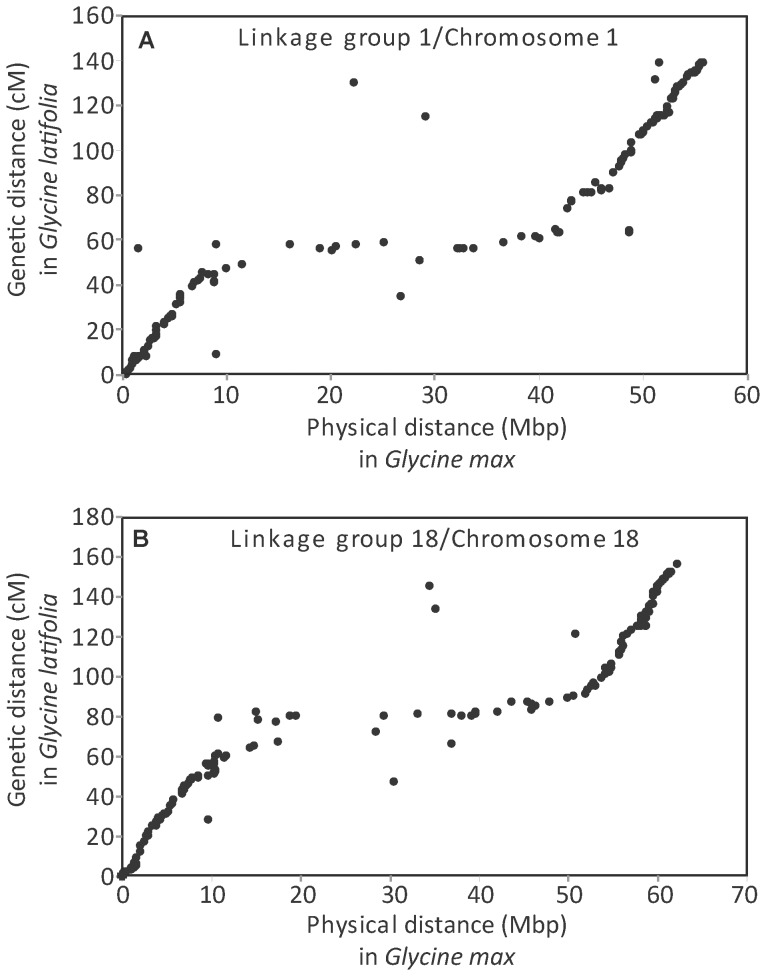
Comparison of genetic distances in *Glycine latifolia* to physical distances in *G. max* for linkage groups/chromosomes 1 and 18. *Glycine latifolia* linkage groups 1 and 18 showed a high degree of collinearity with the corresponding *G. max* chromosomes. The genetic distances in *G. latifolia* were plotted against the physical locations of the SNP markers on the *G. max* chromosomes 1 and 18. As in *G. max*, the predicted ratios of genetic and physical distances varied along *G. latifolia* linkage groups. The slopes were steeper near the ends of linkage groups and flatter near the center in regions predicted to correspond to centromeres, where recombination is lower.

As with molecular markers in *G. max*, comparison of the genetic distances between GBS markers in *G. latifolia* and the physical distances between positions to which the SNP-containing sequences aligned on *G. max* chromosomes, indicated that there was reduced recombination in regions that corresponded to *G. latifolia* centromeres (Figure 3). Points deviating from the main line may represent mis-aligned, or mis-mapped sequences or intrachromosomal rearrangements. No *G. latifolia* markers were identified that aligned to 13.7 Mb and 15.0 Mb in the central regions of *G. max* chromosomes 5 and 20, respectively, which may have been caused by low GBS marker density (i.e., lack of *Pst*I cutting sites) or low sequence conservation in highly repetitive pericentromeric regions.

### Similarities between *G. latifolia* linkage groups and *Phaseolus vulgaris* chromosomes

**Figure 4:**
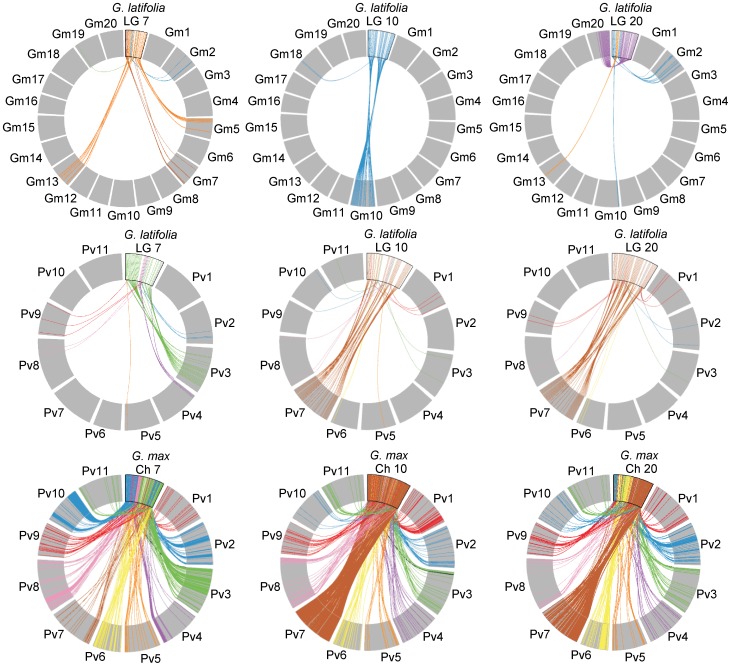
Comparison of synteny of individual *Glycine latifolia* linkage groups 7, 10 and 20 with *G. max* and *Phaseolus vulgaris* chromosomes. *Glycine latifolia* linkage groups (A & B) and *G. max* chromosomes (C) are placed at the top of each circle with colored lines connecting positions of *G. latifolia* SNP markers (A & B) or *G.max* gene model (C) sequences to positions in *G. max* (Gm01 – Gm20) or *P. vulgaris* (PV01 – PV11) chromosomes, represented by gray boxes. *Glycine latifolia* linkage groups 7 and 20 appeared to have larger syntenic blocks with single *P. vulgaris* chromosomes than *G. max* chromosomes. Both *G. latifolia* linkage groups 10 and 20 showed large blocks of synteny with *P. vulgaris* chromosome 7, as did *G. max* chromosome 10. Synteny maps were constructed using MizBee [42].

The progenitor of *P. vulgaris* diverged from the *Glycine* about 19 million years ago [47], [48]. While 3,664 SNP-containing sequences from *G. latifolia* aligned to the *G. max* genome, 2,063 *G. latifolia* sequences aligned to *P. vulgaris* chromosomes. For *G. max*, the sequences of 30,327 gene models aligned to *P. vulgaris* chromosomes. As reported for comparisons between *G. max* and *P. vulgaris* [49], extensive blocks of synteny were observed between *G. latifolia* LGs and *P. vulgaris* chromosomes (Figure 4). McClean *et al.* observed that the pericentromeric regions of *G. max* chromosomes 10, 12, 14, 17, 18, and 20 had extensive syntenic blocks with *P. vulgaris* chromosomes. *Glycine latifolia* LGs 7 and 20 appeared to have larger syntenic blocks with single *P. vulgaris* chromosomes than with *G. max* chromosomes. Both *G. latifolia* LGs 10 and 20 showed large blocks of synteny with *P. vulgaris* chromosome 7, as did *G. max* chromosome 10. The results suggest that *G. max* chromosome 7 and 20 have been reorganized after the whole genome duplication event, but *G. max* chromosome 10 and *G. latifolia* LGs 7, 10 and 20 appear to have retained gene orders more similar to shared ancestral chromosomes.

## Discussion

*Glycine latifolia* is a perennial relative of soybean that is native to eastern Australia with a trailing or twining growth habit [50]. Like *G. max*, the genome of *G. latifolia* contains 2*n* = 40 morphologically similar chromosomes [50]. In this study, we used GBS to construct high-density linkage maps for *G. latifolia* and showed that eight of the 20 *G. latifolia* LGs were rearranged relative to *G. max* chromosomes. Linkage maps were constructed *de novo* from F_2_ and F_5_ populations to confirm marker orders. Genotyping by sequencing has been applied to several plant species with and without reference genome sequences. For example, Sonah et al. [51] reported the application of GBS to a set of eight diverse soybean genotypes and identified from 4,028 to 5,807 SNPs between pairs of soybean lines by aligning sequence reads to the *G. max* genome sequence. Similar to results reported here, Ward et al. [52] produced genetic maps for raspberry (*Rubus idaeus* L.) without a reference sequence consisting of 2,391 and 4,521 markers. Russell et al. [53] used 1,901 SNPs identified using GBS without a reference sequence to map quantitative trait loci in blackcurrant (*Ribes nigrum* L.). Similar to this study, Ma et al. [54] identified 3,745 SNP by GBS in *Miscanthus sinensis* Anderss, a potential bioenergy crop [55], and used them to compare the genome organization of *M. sinensis* to those of *Brachypodium distachyon* L., Oryza sativa L., *Sorghum bicolor* (L.) Moench, and *Zea mays* L. by aligning the SNP-containing sequences to the heterologous genome sequences.

Mahama et al. [56] genetically and cytologically identified seven different chromosomal translocation lines in soybean and reported that crossing a soybean line homozygous for a single translocation with a wild-type soybean line resulted in significant levels of pollen abortion, ovule abortion, and reduction in seed set. Hence, it is not surprising that crosses between *G. latifolia* and *G. max* that involve eight chromosomes with multiple translocations do not produce fertile progeny [57]. Following embryo rescue and colchicine treatment to double chromosome numbers [58], it may be possible to recover lines that retain unrearranged *G. latifolia* chromosomes from wide-hybridization experiments between *G. latifolia* and *G. max*, but lines containing chromosomes with multiple translocations would likely not be fertile.

Using fluorescence *in situ* hybridization–based karyotyping of the seven soybean translocation lines, Findley et al. [59] identified reciprocal translocations between *G. max* chromosomes 1 and 8; 2 and 8; 2 and 11; 4 and 13; and 5 and 13. It is interesting that *G. latifolia* LGs also showed reciprocal exchanges between chromosomes 2 and 8, which may indicate the presence of recombination hotspots in the rearranged chromosomes. It has been difficult to identify translocations in soybean because of its small and morphologically similar chromosomes [60]. Consequently, it is possible that other recombination hotspots remain to be identified.

Based on hybridization success, hybrid seed viability, fertility of F_1_ plants in intra- and interspecific hybrids and degree of meiotic chromosome pairing, species within the genus *Glycine* have been assigned genome types [61]. *Glycine latifolia,* along with *G. microphylla* Tindale, and *G. tabacina* (Labill.), Benth (all 2n = 40) contain B genome types. In interspecific crosses, *G. latifolia, G. microphylla* and *G. tabacina* produce vigorous F_1_ plants with normal seed set [61]–[64], which suggests that *G. microphylla* and *G. tabacina,* other than paracentric inversions [65], have chromosome structures very similar to those found in *G. latifolia*. In contrast, F_1_ plants from crosses between D-genome perennial species (i.e., *G. tomentella*) show seedling lethality [50], [63]. It has been possible to recover plants from crosses between *G. tomentella* and *G. max*, using *G. tomentella* lines with 78 chromosomes [16], [17]. The 2n = 78 *G. tomentella* lines may contain a set of 20 chromosomes that have fewer rearrangements than *G. latifolia* that facilitate chromosome pairing during crossing with *G. max*.

Generally, nuclear genomes of closely related species show high degrees of collinearity that degrade with increasing phylogenetic distance, but the rates at which chromosomes diverge vary widely among taxa [66]. In comparisons of genetic maps between *Arabidopsis thaliana* (L.) Heynh. and *Brassica nigra* (L.) W.D.J. Koch, Lagercrantz [67] estimated that there had been about three chromosomal rearrangements per million years since the two species had diverged 11 to 35 million years ago [68]. In contrast, Koch et al. [69] estimated that *Arabidopsis lyrata* L. and *Capsella rubella* Reut. had undergone less than 0.09 rearrangements per million years since the two lineages diverged 10 to 14 million years ago. Like *G. tomentella*, *G. latifolia* diverged from the progenitors of *G. max* between 5 and 7 million years ago [20], [70], which would mean that the genomes of *G. latifolia* and *G. max* have undergone up to five interchromosomal rearrangements per million years. This number is higher than in the studies mentioned above and may indicate a higher rate of chromosomal instability or simply that a higher density of markers was used which permitted the detection of a larger number of chromosomal rearrangements. In addition to being geographically isolated from the progenitors of *G. max*, *G. latifolia*, like other 2n = 40 perennial *Glycine* species, often produces seed from cleistogamous flowers [60], which reduces its opportunities for outcrossing and possibly removing some of the selection pressure against chromosomal rearrangements. As a consequence, more translocations may have been preserved in *Glycine* species than in other non*-Glycine* species examined that outcross more readily.

As functions are assigned to *G. max* genes, the perennial *Glycine* species become important sources of new alleles that can be isolated and moved into *G. max* by standard transformation or developing gene replacement technologies [71]–[73]. Jones et al. [74] recently demonstrated the feasibility of this approach by using *Agrobacterium*-mediated transformation to transfer a functional gene for resistance to late blight (caused by *Phytophthora infestans* (Mont.) DeBary) from *Solanum venturii* Hawkes & Hjerting to cultivated potato (*Solanum tuberosum* L.). Gene identification in perennial *Glycines* species would be greatly aided by determining the genomic sequences for at least a subset of the species. Because many of the chromosomes of perennial *Glycine* species are collinear with their *G. max* homologues, the *G. max* genome sequence could be used as a reference to assemble genome sequences of perennial *Glycine* species. Using the heterologous *G. max* reference sequence will reduce the depth of sequence coverage needed for genome assembly compared to *de novo* genome sequencing [75], [76]. Even though genetic hybridization may not be possible by standard means, methods for high-throughput gene mapping and identification afforded by next-generation sequencing provide tools to capture the variation present in the wild species.

## Supporting Information

Figure S1 Genetic linkage maps of GBS markers in a *Glycine latifolia* F_2_ population.
(TIF)

Figure S2 Genetic linkage maps of GBS markers in a *Glycine latifolia* F_5_ population.
(TIF)

Figure S3 Consensus genetic linkage maps for *Glycine latifolia* derived from 3710 GBS markers.
(TIF)

Table S1 GBS SNP Genotype data for the F_2_ mapping population.
(DOCX)

Table S2 GBS SNP Genotype data for the F_5_ mapping population.
(DOCX)
