# Supplementary material for: Comparative Mapping of the Wild Perennial Glycine latifolia and Soybean (G. max) Reveals Extensive Chromosome Rearrangements in the Genus Glycine
Source: PLoS One. 2014 Jun 17;9(6):e99427. doi: 10.1371/journal.pone.0099427 (PMC4061007; doi:10.1371/journal.pone.0099427)
Supplement: Table S1 — GBS SNP Genotype data for the F2 mapping population. (DOCX) [file pone.0099427.s004.docx]

Table S1. GBS SNP genotype data for F_2_ mapping population

#lines 11_101 11_102 11_15 11_27 11_6 15_12 15_13 15_14 15_18 15_19 15_21 15_22 15_24 15_25 15_9 18_1 18_19 18_20 18_21 18_3 18_6 18_7 18_9 20_12 20_17 20_19 20_2 20_24 20_4 20_6 20_8 25_105 25_108 25_114 25_115 25_119 25_125 26_1 26_12 26_14 26_15 26_19 26_2 26_25 26_26 26_4 26_5 26_8 26_9 27_14 27_18 27_2 27_22 27_23 27_26 27_27 27_5 27_8 29_101 29_104 29_105 29_107 29_112 29_113 29_114 29_115 29_126 36_10 36_14 36_16 36_18 36_25 36_26 36_29 36_3 36_5 39_110 39_111 39_112 39_118 39_120 39_122 39_125 3_10 3_12 3_13 3_15 3_17 3_22 3_25 3_29 3_3 3_31 3_35 3_37 3_4 3_5 3_6 3_9 4_1 4_19 4_2 4_21 4_22 4_24 4_25 4_29 4_3 4_30 4_31 4_32 4_37 4_38 4_40 4_5 4_7 5_13 5_18 5_20 5_22 5_24 5_27 5_29 5_30 5_5 5_6 5_7 5_9 6_1 6_10 6_27 6_3 6_30 6_4 7_1 7_107 7_13 7_18 7_26 7_27 7_28 7_30 7_5 7_6

*002325_00051613 BHHHB-H-B-H-HHHHAHAHBHH-BA--H-BHHAHHHBAA-H-A-HHHHBHHHHA-HHHHHHHHAHH--H-H-BHBH-HHAHBH-HHH-BBHHBBHHBHBHH-BAH--AH-ABH-AH-AHHAH--HHH--HHHHABH---HHAB

*008173_00002266 HBHHHHBABHHBHHAHAHHHHHBHHHHHHHHHHHHAAHAHHHHBBHHHAABHAAHAHHABBHHHHHHHAHHHHHHBHHHHHHAHAHHHHBHBAAHHHBHBHHHABBAHHHBHHBHABH-HHHHHHBHABHBABHHHHAHHHBHB

*000975_00102032 AAAH-HHAAHH-AH--HHH-HAAHHHHHHHHHA-AHHA-HAAAHAHHAHAHHABH-HAAHAHHAHHHHHAHHHHHH-AAHHH-H-HHHAHH-H-AHAHHHAHAHAH-HHH-HHAH-AAA-AHHHHHHAA-HHH-HBA-A-AHH-

*000422_00410318 HHAAHAHAAHHBAHAAHHHHHHHAHBABAAHHHHBHHAHHAAAHHBHHHHHHHHHHHAAHHAHHHHHAHHBH-HHA-HHAHAAHAHBHHHHHAHHBHAHHAHHHHAAHHHBHBHABHHAHHAHHHHHHAHHHBHBHHHAHHHHB

*010066_00026537 B-AHBHBHHHBAAHHBHBHHHBHBAHBHHHBHABHBHHHHHHHBBBBHHHBHBHHHHHHHHHHBHHHAHHHAHHHHAHBABABHHHHAHBAHBBHHHHHHHHHBHHAHHBHHHHHABHAHHAHHAHBHHAHHHAHHHHAHAHB-

*000479_00369836 A-HHAHHAHAH-HAHAHHHAAAHHH-HHHAHHHHHAAAAHHHHHHHHHHAHAHHAAHHHHHHHAAHABBH-AAHHH-H-HHB-AHHAHAHHAA-HAHHHHHHHHHHAHAHHHHHHAHAHHAHHAHHAAAHHHAAA-HAHAAHAA

*001195_00005919 HHAAHHBHHAHHHABBHABHHAHHAHAHBABBAABHHHAHHHHHHHAHHHHHHHABHHBHBAHAABHHAHHHAAHBHBHHHHHHBHHHAHHAAHHBBHHHHHAAHB-BHHHHHAHHHHHAHHHHHBHHBBHAHH-HHHHHHHAH

*004624_00011356 AHHAAHAHA-AHHH--HAAHHAHHAAHHHAHAH-HHHAAHBH-AHAHHAA-HAABHHHHAAHHHHHHHHAHAHHAAAHHAHAHHHHAHHH-HHAAHHHHBAHHHHAAHHAAHHHHH-HAAHHHHHHAHH-HHHHHHAHAHAAHA

*001329_00279543 HHHAHHHAHBH-AHHHHHHHHHHHHHHBHBHAHHHHHBHAHAB-HHHHAHAAHHHHHAHHHAHHAHHAHHHHBHAHBHAHHA-HAHHBBAHHBHHHHBHHHHAHBAAAHHHHBHHHAABAHHBHAHHBHHBBHHBABBABHAHB

*002923_00010163 HHAHHHBHHAHHHABBHABAHAHHAAAHBABBAABHHHAHHHHHHHAHHHHHHHABHHBHBAHAABHHAHHHAAHHHBHHHHHHBHHHAHHAAHHBBHBHHHAAHBBHHHHHHAHHHBHAHHHHHBHHHBHAHHAHHHHHHHAH

*002923_00033955 HHAHHHBHHAHBHABBHABAHAHHAAAHBABBAABHHHAHHHHHHHAHHHHHHHABHHBHBAHAABHHAHHHAAHHHBHHHHHHBHHHAHHAAHHBBHBHHHAAHBBHHHHHHAHHHHHAHHHHHBHHHBHAHHAHHHHHHHAH

*001024_00002370 HAHHHHHHAHHBHBHHHHHHAHBHHAHAHAABHBHHHBBHHHAAHBHHAHHAAHHHHHHHBAHHHHHAHBHHHAHHAHHHHHBBHAHHHHHHHABAHAAHAHHHBAAHHAHHHHHBHBHBHHHA-HAAHHBHBHAHAHBAHAHB

*001973_00176751 HBBAHABHHAHABHHBHA-HBHHBB-AHHHHHHHHBAAHHHH-HHHHAHA-AHA-HHBA-HHHAHHHAHBHHABHH-HAAHAA-HB-HHBAHHBHHH--HBHHH--BH-HHHHHH-HB-HHBHH-BAAH-AHH-H-HBHHAHHH

*000707_00165233 HBBBABBBBBBBHHHBBHHHHHABHHBBHHBHHAHHHHHHHHHHHHHHBHBHBHHBHHHHHBHHHHHH--HBBHHH-HHAH-HHHHHBABHBHHHHHBHHBHHHHBBBHHHHHBABBBBHHBBBBBHH-BBHA-BHHHHBHHHH

*000478_00155481 H-HH-HBHB-HHBHBHHBB-H-H-B-HBHHHBBHBHAHHHBHHHHHHHBB-HH-H-HH-HAHBHBHH--BHB-HHH-BBHHB-H--BHHBHHBHH-HHBHBHBHHBBHBHHHHBH-HHHHBHA--BHBHBBBHBHBH-BHB-H-

*003109_00012945 H-HHHAA--HH--A--HHA---A-A-H-H-AA--AHHHHH-AHHAHHB-HHA-HH-HHBAHHHA-HHAHAHAHHHH-HAHA--H-HHHAA-AAAH-HHHAHHHBH-AHAHBAHH---AAHAAHA--HH--HHHHAH--AHHHA-

*000222_00019242 HAHHHHHHHHHBHBHHHHHHAHBHHAHAHAABHBHHHBBHHHAAHBHHAHHAAHHHHHHBBAHHHHAAHBHHHAHHAHHHHHBBHAHHHHHHHABAHAAHAAHHBAAHHAHHHHHBHBHBAHHABHAAHHBHHHAHAHHAHAHH

*003002_00048537 AHBHBABHBBBBHHHHHHHBHABHHHAHBHHHHHBHHHHHHABHHHHHAAHHHAAHBBHBHHHHHHH--HHBBHBBBHBHHB-HHBHHHBHHAHHHHHBBAHHHHBBHBHHAHBB-HHAAHHHHBHHBB-HHHHBBA-HB-HHH

*000329_00210386 HHHHHHHAHAHAHHHHHHHHAHHHHHHAHBBHAHBAHHHHHBHHHHHHHHHHHHBBHHHHHABHHBHHAHBAAHBBHHBHAABHHHAHAHABBHBBHABHHBAHHAHAHHHABHHAHHHHBBHAHAAHBHHBHHHBBAHBAHHH

*000009_00672567 ABHBBHHHAHBAAHBHHHHHHHHHAHHHBBBHHAHAHHHBAHHHHHAHHHHHBHABHBHHHABAABHHABBHHHHBHHHHAAHBHHAAAHHAAHHHHHBHBBHHHHABHBHHBHAHHHHHBHHHBAHHHHHHBHHABHBHAHHH

*000045_00713516 HHHHBHAHAAHAHHHHHBHHAHHHAHHAHBBHHHBAHHHHHBHHHHHHHHHHBAABHHHHHABHHBHHAHBHAHHBHHHHAABHAHAHAHABHHBBHABHHBAHHHAAHHHABHHAABHHBBHHHAAHBHHBHHHHB-HHAHHH

*001525_00041969 AHAHHHBHHAHHHABBHABAHAHHAAAHBABBAABHHHAHHHHHHHAHHHBHHHABHHBHBAHAABHHAHHHAAHHHBHHHHHHBHHHAHHAAHHBBHBHHHAAHBBHHHHHHAHHHHHAHHHHHBHHHBHAHAAHHHHHHHAH

*000045_00813215 HHHHBHAHAAHAHHHHHBHHAHHHAHHAHBBHHHBAHHHHHBHHHHHHHHHHBAHBHHHHHABHHBHHAHBHAHHBHHHHAABHAHAHAHABHHBBHABHHBAHHHAAHHHABHHAABHHBBHAHAAHBHHBHHHHBAHHAHHH

*000238_00153216 HHBHBHBHHHHHHAHAHHBHHAHHHHHHHHHHHHHBHAHBAABAHHBHAHHHHHBBHHHHHBHHHABHHBBBHHHHABHABAAHHHABHBHAHBHAHBBHHHBHHABABHHHBHAHHHHHHAHHHBBHHHHHBHHAHBHBABHB

*000668_00202057 HHAHHHBHHAHHHABBHABAHAHHAAAHBABBAABHHHAHHHHHHHAHHHHHHHABHHBHBAHAABHHAHHHAAHHBBHHHHHHBHHHAHHAAHHBBHBHHHAAHBBHHHHHHAHBBHHAHHHHHBHHHBHAHHAHHHHHHHAH

*001195_00139921 HHAHHHBHHAHHHABBHABAHAHHAAAHBABBAABHHHAHHHHHHHAHHHHHHHABHHBHBAHAABHHAHHHAAHHHBHHHHHHBHHHAHHAAHHBBHBHHHAAHBBHHHHHHAHHBHAAHHHHHBHHBBHAHHAHHHHHHHAH

*003127_00032219 HHHHHHHAHAHAHHHHHHHHAHHHHHHAHBBHAHBAHHHHHBHHHHHHHHHHBHBBHHHHHABHHBHHAHBHAHBBHHBAAABHAHAHAHABBHBBHABHHBAHHAAAHHHABHHAHHHHBBHAHAAHBHHBHHHHBAHHAHHH

*000009_00220000 HBHBBHHHAHBAAHBHHHHHHHHHAHAHBHBHHAHAHHHBAHBHHHAHHHHHBHABHBHHHABAABHHABBHHHHBBHBHAAHBHHAA-HHAAHHHHHHHBHHHHHHBHBHHBHABHHHHBHHHBAHHBHHHBHHAHABHAHHH

*008964_00007175 HAHHHHHHAHH-BBBHHHHHAABHHAHAHAABHBHHHBBHHHAAHBHHAHHAAHHHHHHHBAHHHHHAHBHHHAHHAHHHHHBBHAHHHHHHHABAHAAHAHHHBAAHHAHHHHHBHHHBHHHABHAAHHBHBHAHAHBAHAHB

*000316_00417272 HHHABAHAHAHHAHHHAAAAHHHH--HAHA-HAAHHAHHAHABAHHHHHAHHHHHHAHHAAHHAAAHAAH-AAHHHAH-HAAHH-HHHHHHAH-HHH-HHHAHHHHAHAHHHHHHA-AH-HHAA-A-AAAHAHAAHH-HAAHAH

*001064_00067642 HHHHHHHHHAHHHHHHHAHHAHHHHHHAHBBHAHBAHHBHHBBHHHHHBHHBHHBBHHHHHAHHHBHHAHBAAHBBHHHAAABHHHHHAHABBHHBHABHABAHAHHAHHHABHHAHHAHBBHAHAAHBHABHHHBB-HBHHHH

*000480_00007762 HAHAAHHHHHBHABHHHHAHAHAHHHHAHAABBBHAAHBHHHHAHAHAAHHAHBAHHHHABAHHAHHABHHAHAHHAAHHHHHBHABHHHHHHABHHHBHAHHHAHHHHHHABHHHHHHBHHHABHHHHHAHBHHHAHBHBAAB

*000746_00098074 AHAHHHBHHAHHHABBHABAHAHHAAAHBABBAABHHHAHHHHHHHAHHHBHHHABHHBHBAHAABHHAHHHAAHHHBHHHHHHBHHHAHHAAHHBBHBHHHAAHBBHHHHHHAHHBHHAHHHHHBHHHBHAHAAHHHHHHHAH

*001024_00047522 HAHHHHHHAHHBHBHHHHHHAHBHHAHAHAABHBHHHBBHHHAAHBHHAHHAAHHHHHHHBAHHHHHAHBHHHAHHAHHHHBBBHAHHHHHHHABAHAAHAHHHBAAHHAHHHHHBHBHBAHHABHAAHHBHBHAHAHBAHAHB

*001338_00010913 HAHHHHHHAHHBHBHHHHAHAHBHHAHAHAABBBHHHBBHHHAAHBHHAHHAAHHHHHHHBAHHAHHAHBHHHAHHAHHHHHBBHAHHHHHHHABAHAAHAHHHHAAHHAHHHHHBHBHBAHHABHAAHHHHBHAHAHBAHAHB

*000288_00023238 HHHHHHHAHAHAHHHHHHHHAHHHHHHAHBBHAHBAHHHHHBHHHHHHHHHHBHBBHHHHHABHHBHHAHBHAHBBHHBAAABHAHAHAHABBHBBHABHHBAHHAAAHHHABHHAHHHHBBHAHAAHBHHBHHHBBAHHAHHH

*000467_00275725 HHHHBHAHAAHABHHHHBHHAHHHAHHAHBBHHHBAHHHHHBHHHHHHHHHHBAHBHHHHHABHHBHHAHBHAHHBHHHHAABHAHAHAHABHHBBHABHHBAHHHAAHHHABHHAABHHBBHAHAAHBHHBHHHHBAHHAHHH

*000639_00183795 HHHHHHHAAAHAHHHHHHHHAHHHHHHAHBBHAHBAHHHHHBHHHHHHHHHHBHBBHHHHHABHHBHHAHBHAHBBHHBAAABHAHAHAHABBHBBHABHHBAHHAAAHHHABHHAHHHHBBHAHAAHBHHBHHHHBAHHAHHH

*001000_00295361 HHHHHHHAHAHAHHHHHHHHAHHHHHHAHBBHAHBAHHHHHBHHHHHHBHHHHHBBHHHHHABHHBHHAHBAAHBBHHBHAABHHHAHAHABBHHBHABHHBAHAAHAHHHABHHAHHHHBBHAHAAHBHABHHHBBAHBAHHH

*001834_00203182 HAHHHHHAH-HAAHHHAHHHA-HHHHHAHBBHAHBAHHHHHB-HHHHHHHHHBHBBHHHHHABHHBHHAABH-HBBHHBAAABHHHAHAHABHHBBHABHHBAHHAAAHHHABHHAAHAHB-HAHAHH-HH-HHHBBAHHAHHH

*000238_00218628 HHBHBHBHHHHHHA-AHHHHHAHHHHHHHHHHHHHBHAHBAABAHHBHAHHHHHBBHHHHHBHHHABHHBBBHHHHABHABAAHHHABHBHAHBHAHBBHHHBHHABABHHHBHAHHHHHHAHHHBBHHHHBBAHAHBHBABHB

*001064_00032894 HHHHHHHHAAHHHHHHHAHHAHHHHHHAHBBHAHBAHHBHHBBHHHHHBHHBHHBBHHHHHAHHHBHHAHBAAHBB-HHAAABHAHHHAHABBHHBHABHABAHAHHAHHHABHHAHHHHBHHAHAAHBHABHHHBBAHBHHHH

*001834_00201285 HHHHHHHABAHAHHHHHHHHAHHHHHHAHBBHAHBAHHAHHBHHHHHHHHHHBHBBHHHHHABHHBHH-HBHAHBBHHBAAABHAHAHAHABBHBBHABHHBAHHHAAHHHABHHAHHHHBBHAHAAHBHHBHHHBBAHHAHHH

*000189_00699303 BBHAHHHHAABHHHBBBHAHHABHHHHHBHBHHBHHABBBBHAHHBBAAAAABAHBHHAHHBHHHHHAHHHHHBBHHHHHAAHAHAHHHHAHHHAHBHAHHBHHBHAHAAHHHAHAHHHHHHHHHHHHHHBHHAHAHHHBHBBA

*000222_00019608 HAHHHHHHHHHBHBHHHHHHAHBHHAHAHAABHBHHHBBHHHAAHBHHAHHAAHHHHHHBBAHHHHAAHBHHHAHHAHHHHHBBBAHHHHHHAABAHAAHAAHHBAAHHAHHHHHBHBHBABHABHAAHHBHHHAHAHHAHAHH

*000222_00486132 HAHHHHHHAHHBHBHHHHHHAHBHHAHAHAABHBHHHBBHHHAAHBHHAHHAAHHBHHHHBAHHHHHAHBHHHAHHAHHHHHBBHAHHHHHHHABAHAAHAHHHBAAHHAHHHHHBHBHBAHHABHAAHHBHBHAHABBAHAHB

*001525_00079464 AHAHHHBHHAHHHABBHABAHAHHAAAHBABBAABHHHAHHHHHHHAHHBBHHHABHHBHBAHAABHHAHHHAAHHHBHHHHHHBHHHAHHAAHHBBHBHHHAAHBBHHHHHHAHHBHHAHHHHHBHHHBHAHAAHHHHHHHAH

*001800_00118441 HAHHHHHHHHHBHBHHHHBHAHBHAAHAHAABHBHHHBBHHHAAHBHHAHHAAHHHHHHBBAHHHHAAHBHHHAHHAHHHHHBBBAHHHHHHAABAHAAHAAHBBAAHHAHHHHHBHBHHHHHABHAAHHBHHHAHAHHAHAHH

*000045_00499355 HHHHBHAHAAHAHHHHHBHHAHHHAHHAHBBHHHBAHHHHHBHHHHHHHHHHBAABHHHHHABHABHHAHBHAHHBHHHHAABHAHAHAHABHHHBHABHHBAAHHABHHHABHHAABHHHBHAHAAHBHHBHHHHBAHHAHHA

*000045_00628144 HHHHBHAHAAHAHHHHHBHHAHHHAHHAHBBHHHBAHHHHHBHHHHHHHHHHBAABHHHHHABHHBHHAHBHAHHBHHHHAABHABAHAHABHHBBHABHHBAAHHABHHHABHHAHBHHBBHAHAAHBHHBHHHHBAHHAHHH

*000068_00261314 HHHHHHHHHAHAHHHHHHHHAHHHHHHAHBBHAHBAHHBHHBBHHHHHBHHHHHBBHHHHHABHHBHHAHBAAHBBHHHAAABHHHAHAHABBBHBHABHHBAHAAHAHHHABHHAHHHHBBHAHAAHBHABHHHBBAHHAHHH

*000238_00194558 HHBHBHBHHHHHHAHAHHBHHAHHHHHHHHHHHHHBHAHBAABAHHBHAHHHHHBBHHHHHBHHHABHHBBBHHHHABHABAAHHHABHBHAHBHAHBBHHHBHHABABHHHBHAHBHHHHAHHHBBHHHHBBHHAHBHBABHB

*000392_00384600 HHAHHHHAHHHHHBBHBHBHHHBHABBHBBHHBHBHHAHAAHBHHBHHHHHHBHABABHAHHHAAHBAHAAHHHAHHHBBHHHHHAHBHHHAHBHHABBHHBAHBHHAHHHHHHHBBHHHHBAHHHHAHHBBBAAHAHHAHHHH

*000505_00066359 HHHHHHHBBHHHHBABHHBHAHBHHHHHHHBHAHHHHBHBBHBHHHHAABABHHBBAHAHHHBBHAHAAHBHHAAHHHHHHHHHHBAHBHHHHHHAHABBHHAAHAHHAABHHABAHAHBHHAAHHAAHHHHHHAHHHBHABHH

*000505_00126265 HHHHHHHBBHHHHBABHHBHAHBHHHHHHHBHAHHHHBHBBHBHHHHAABABHHBBAHAHHHBBHAHAAHBHHAAHHHHHHHHHHBAHBHHHHHHAHABBHHAAHAHHAABHHABAHAHBHHAAHHAAHHHHHHAHHHBHABHH

*000551_00285936 HHHHHHHHAAHHHHHHHHHHAHHHAHHAHBBHAHBAHHBHHBBHHHHHBHHBHHBBHHHHHAHHHBHHAHBAAHBBAHHAAABHHHHHAHABBHHBHABHABAHAHAAHHHABHHAHHHHHBHAHAAHBHABHHHBBAHBHHHH

*000621_00251073 HHHHHHHAAAHAHHHHHHHHAHHHHHHAHBBHAHBAHHHHHBHHHHHHHHHHBHBBHHHHHABHHBHHAHBHAHBBBHBAAABHAHAHAHABBHBBHABHHBAHHAAAHHHABHHAHBHHBBHAHAAHBHHBHHHHBHHHAHHH

*000854_00183434 HHBHBHBHHHHHHAHAHHBHHAHHHHHHHHHHHHHBHAHBAABAHHBHAHHHHHBBHHHHHHHHHABHHBBBHHHHABHABAAHHBABHBHAHBHAHBBHHHBHHABABHHHBHAHBHHBHAHHHBBHHHHHBHHAHBHBABHB

*001331_00028307 HHHHHHHHHAHABHHHHHHHAHHHHHHAHBBHAHBAHHBHHBBHHHHHBHHBHHBBHHHHHAHHHBHHAHBAAHBBHHHAAABHHHHHAHABBHHBHABHABAHAAHAHHHABHHAHHHHBBHAHAAHBHABHHHBBAHBHHHH

*001349_00141928 HHHHHHHAHAHAHHHHHHHHAHHHHHHAHBBHAHBAHHHHHBHHHHHHHHHHBHBBHHHHHABHHBHHAHBHAHBBHHBAAABHAHAHAHABBHBBHABHHBAHHAAAHHHABHHAHHAHBBHAHAAHBHHBHHHBBAHHAHHH

*001637_00073276 HHAAHAHHHAHHHAHBHABBHAHHAAAHBABHAABHHBAHHHHHHHAHAHHBAHABHHBAHAHAHHAHAHHHAAHBHBHHHHHBBBHHAHHAAHHHBHHHHHAHHBBBHHHHHAHHHHAAHHHHBBHHBBHHHHAHHHHHHHAH

*004426_00021201 HHHHHHHAAAHAHHHHHHHHAHHHHHHAHBBHAHBAHHHHHBHHHHHHHHHHBHBBHHHHHABHHBHHAHBHAHBBHHBAAABHAHAHAHABBHBBHABHHBAHHAAAHHHABHHAHBHHBBHAHAAHBHHBHHHHBAHHAHHH

*001937_00034320 BHH-HBHHA-H--H-HBAHBHHHH-BB-AHBHBHAHHHBAHB-BHHBHHHBBBH-HHBHHHHHHHHH-AHHABHHHBHAHHH-B-H-HHBHAHHHBHAHBHBBHHB--AHHHHHHABBHHB-B--HH-H-HBBBA-B-BHHHBB

*001670_00172588 HHABHHHAHHHBAHAAHB-HH-HBBBAHAAHHAHBAH-HHAAAAHHHHHHHHAHHHHAHHHHHAAHHABHHHBHHAAHHHAH-H-HAHHHHAAAAHHHAHHBHHAA-HAHBHHHAAHH-HHAHBHHBHABAHHAHHHHA-AHHB

*000583_00217386 HHHHHHAAAAHAHHHHHBHHAHHHAHHAHBBHHHBAHHHHHBHHHHHHHHHHBABBHHHHHABHHBHHAHBHAHHBBHHHAABHAHAHAHABHHBBHABHHBAHHA-AHHHABHHAABHHBBHAHAAHBHHBHHHHBAHHAHHH

*000854_00179912 HHBHBHBHHHHAHAHAHHBHHAHHHHHHHHHHHHHBHAHBAABAHHBHAHHHHHBBHHHHHHHHHABHHBBBHHHHABHABHAHHBABHBHAHBHAHBBHHHBHHABABHHHBHAHBHHBHAHHHBBHH-HHBHHAHBHBABHB

*002213_00093893 HHBABHHHHHBBHAHHHHBHHHAHHAHHHHHHHHABHAHBAHBAHHBHAHHHHHBBHAAHHHHHHAHHBHBBBHHHABHABHAAHBHBHBHAHBHHHBBHHHBHHABABHHHBHAHBHHHHAHHHHBHHAHHBABAH-HHHBHB

*000037_00128852 HBHHHHABAHBHHHHHHHHAHHBAHHHHHHHHHHHAHAHHHBHHAHHHHHABHHHBBHAAHHHHBHABHBBABAHBHHHBHAHBHAAAHBHBHHAAHHAABHAHHHBAHBBAHAHHHHHHBHBHAHAABHHHHABHHHAAAHHH

*000037_00156225 HBHHHHABAHBHHHHHHHHAHHBAHHHHHHHHHBHAHAHHHBHHAHHHHHABHHHBBHAAHHHHBHABHBBABAHBHHHBHAHBHAAAHBHBHHAAHHAABHAHHHBAHBHAHAHHHHHHBHBHAHAABHHHHABHHHAAAHHH

*000100_00009348 AHHBHHHHAHBHAHAHBHBHHHHHABBBBHHHAHHHHHABHHAAHBHHABAAHHAHBBABHHHHBHBHBHAABAHAAHAHHHHBHHHHHHAAAHHAHAHHHAHBHBHHHABAHHHHHAHHHHBHHBAHHBHHHHHABHHHHAHH

*004515_00065714 HHBHBHBHHHHHHA-AHHBHHAHHHAHHHHHHHHHBHAHBAABAHHBHAHHHHHBBHHHHHBHHHABHHBBBHHHHABHABA-HHHABHBHAHBHHHBBHHHBHHABABHHHBHAHBHHHHAHHHBBHHAHBBHHAHBHBABHB

*000392_00211830 HHAHHHBAHHHHHBBHBHBHHHBHABBHBBHHBHBHHAHAAABHHBHHHHHHBHABABHHHHHAAHBAHAAHHHAHHHBBHHHHHAHBHHHAHBHHABBHHBAHBHHAHHAHHHHBHHHHHBAHHHHAHHBBBAAHAHHAHHHH

*000825_00088578 HHBHHBBHHHHBBHHHHABHHHAHHAHHHBHHHHABHAAHBHBAHHBHAHHHHHABHAAHHHHHBHAHHHBBBHHHAHHHBHBAHHHBHBHAHBHHHBHHHHBAHABHBHHBBHABHHBHHAHHHBBBHAHHBABHBBHHHBHH

*002112_00017360 HHAAHAHHHAHHHAHBHABBHAHHAAAHBABHAABHHBAHHHHHHHAHAHHBAHABHHBAHAHAHHAHAHHHAAHBHBHHHHHBBBHHAHHAAHHHBHHHHHAAHBBBHHHHHAHHHHAAHHHHHBHHBBHAHHAHHHHHHHAH

*000757_00058349 HAHHHHHHHHH-HBBHHHHHAHBHAAHAHAABHBHHHBBHBHAAHBHHAHHAAHHHHHHBBAHHHHAAHBHHHAHHAHHHHHBBBAHHHHHHAABAHAAHAAHBBAAHHAHHHHHBHBHBHHHABHAAHHBHHHAHAHHAHAHH

*001978_00049623 ABAHHHHHHHBHHH-HBHBHHHHHBHBHABHHHHHBBH-HBABHHHHBAHHHHBHAHHHHHBAHBHBA-AHHBABA-HAHHHBAHHHAHHHBABHBAHBHHBHHAA-BHHBHAHABHHHAHAHHBHBHAH-AHHBHA-HHHHHA

*003746_00054268 BHHBHHHHHBHBHB-HAHHHHBHBHHHHBAHHHHHBHBBHHBBBHHHBHBAHBHBBHBHHBHHHBHHBABABB-HBBHHHHHHBBHHBAABHBHHBHHH-BBHH-B-HBH-HB---HHBHBBHBHHHHBB-HHHHHH-BHBHBH

*000551_00234903 HHHHHHHHHAH-HHHHHHHHAHHHAAHAHBBHAHBAHHBHHBBHHHHHBHHBHHBBHHHHHAHHHBHHAHBAAHBB-HHAAABHAHHHAHABBHHBHABHABAHAAHAHHHABHHAHHHHHBHAHAAH-BABHHHBBAHBHHHH

*000055_00063093 HBHBHHHBHHBHBBBHHHHHHHBAAHHHHHHHHHBHHABHHBHHAHHHHHABHBBBHBAHHHABHAABHHBHBABHHAHBBAHBHAAAHBHBHHHAHBAABBHHHHHAHHBAHAHHHAHHHHHHHHAHBHHHHAHHHHAHAAHB

*001197_00127862 HBABBHHHAHBHAHBHHHHHBHHHAHAHHHBHHAHAHHBBAHBHHHAHBHHHBHABHBHHHAHHABHHABBHHHHBBHBHAAHBHHHAAHHAAHHHHHHHBHHAHHHBHBHBBAABHHHHBHHBBAHHBHHHBHHAHABHHHBH

*000025_00541633 ABHHHHAHHHHHAHHHBHHHHHABBABHABHHHHHBHHAHBABHBHHBAHHHHBHAHBHBHHABHHHHHHHHHHHAAHAHHHBAHBHAHHHBABHBAHBHABHHHAHBHAHHAHAHHAHAHAHHBHHAAHHAHHBAA-HHHABA

*001918_00096871 A-HH-H-HH-H-AH--BH-BBBABHABH-HHHAB-H-HHHHH-H-HBHHHHBHHHAHHH-HHHABHAHB-HHHBHB-H-AB--BHHA--HHH--H-HH-H-BBHAHHBHHBH-A-AHA-AHB-BB-BBBHAHB-H-H-B--HBB

*003196_00028691 -BAB-HHHHBH-BB-HBHHBH-AH--H-B-HHH--HHH-HBHB-HHHHHHA-HBBHB--AHHHB-HB-HA-A-BAH-BBHAA-H-HHBB-BBH-HH-BHHABHHH-HBBHA--HHHHA-HHH-H-BHBHBBHBB--HA-HHHH-

*000966_00096089 BHHHAAAHHHHHBHHHHHHHABHHAHHABHBBAH-AHBBHBBBHHABHBHHHAHHBHBHBBHHHHHHH-HHBAAHBBAHHAAHHHHHA-HHHBHHBBAAHABHBAHHHBHHHHHH-AHAHHHBAHBHAHHABHHBBHAHBHHHH

*001753_00144247 HHBHBHBHHHHHHAHAHHBHHAHHHHHHHHHHHHHBHAHBAA-AHHBHAHHHHHBBHHHHHBHHHABH-BBBHHHH-BHABAAHHHABHBHAHBHAHBBHHHBHHABABHHHBHAHBHHHHAH-HBBHABHBBHHAHBHBABHB

*000329_00110715 HHHHHHHAHAHAHHHHHHHHAHHHHHHAHBBHABBAHHHHHBHHHHHHHHHHBHBBHHHHHABHHBHHAHBAAHBBHHBAAABHAHAHAHABBHBBHABHHBAHHAAAHHHABHHAHHHHBBHAHAAHBHHBHHHBBAHBAHHH

*000454_00261787 BHHHAAAHHHHHBHHHHHHBABHHAHHABHBHAHBAHBBBBBBHHABHBHAHAHHBHBHBHHHHHHHHHHHBAAHHHAHAAAHHBHHAHHHHBHHBHAAHABHBAHHHBBHAHHHAAHAHHHHAHBHHHHABHHBBHAHBHHHH

*000600_00405132 HHBHBHBHHHBHHAHAHHBHHAHHHHHHHHHHHHHBHAHBAABAHHBHAHHHHHBBHHHHHHHHHABHHBBBHHHHABHABAAHBBABHBHAHBHAHBBHHHBHHABABHHHBHAHHHHBHAHHBBBHHHHHBHHAHBHBABHB

*000761_00006561 HHHHHHHBBAHHHBABHHBHAHBHHHHHHHBHAHHHHBHBBHBHHHHAAHABHHBBHHAHHHBBHAHAAHBHHAAHAHHBHHHHHBAHBHHHHHHAHABBHHAAHAHHAABHHABAHAHBBHHAHHAAHBHHHHAHHHBHABHH

*000827_00006700 HHAAHAHHHAHHHAHBHABBHAHHAAAHBABBAABHHBAHHHHHHHAHAHHBHHABHHBABAHAHHHHAHHHAAHBHBHHHHHBBBHHAHHAAHHHBHHHHHAAHBBBHHHHHAHHHHAAHHHHBBHHBBHAHHAHHHHHHHAH

*002112_00004072 HHAAHAHHHAHHHAHBHABBHAHHAAAHBABHAABHHBAHHHHHHHAHAHHBAHABHHBAHAHAHHAHAHHHAAHBHBHHHHHBBBHHAHHAAHHHBHHHHHAAHBBBHHHHHAHHHHAAHHHHABHHBBHAHHAHHHHHHHAH

*002664_00048419 HHBHBHHHHHHHHAHAHHBHHAHHHHHHHHHHHHHBHAHBAABAHHBHAHHHHHBBHHHHHBHHHABHABBBHHHHABHABAAHHHABABHAHBHAHBBHHHBHHABABHHHBHAHBHHHHAHHABBHHHHBBHHAHBHBABHB

*008093_00001408 HHAAHAHHHAHHHAHBHABBHAHHAAAHBABHAABHHBAHHHHHHHAHAHHBAHABHHBAHAHAHHAHAHHHAAHBHBHHHHHBBBHHAHHAAHHHBHHHHHAAHBBBHHHHHAHHHHAAHHHHHBHHBBHAHHAHHAHHHHAH

*008660_00003497 HHHHBHAAAAHAHHHHHBHHAHHHAHHAHBBHHHBAHHHHHBHHHHHHAHHHBABBHHHHHABHHBHHAHBHAHHBHHHHAABHAHAHAHABHHBBHABHHBAHHAAAHHHABHHAHBHHBBHAAAAHBHHBHHHHBAHHAHHH

*000025_00398393 ABHHHHAHHHHHAHHHBHHHHHABHHBHABHHHHHBHHAABABHBHHBAHHHHBHAHBHBHHHBHHHHHHHHHHHAAHAHHHBAHBHAHHHBABHBAHBHABHHHAHBHAHHABAHAAHAHAHHBHHAAHHAHHBAAAHHHABA

*000247_00358595 ABAABHHHAHBHAHBHHAHHBHAHAHAHHHBHHAHAHHBBAHBHHAHHBHHHBHABHBHHHAHHABHHABHAHHHBHHBHAHHBHHHAAHHAAHHAHHHHBABAHBHBHBHBBAABHHHHBHHBBHHHBHHHBHHHHHHHHHBH

*001375_00270905 HHHHHHHA-AH-HHHHHHHHAHHHHHHAHBBHAHBAHHHHHBHHHHHHBHHHHHBBHHHHHABHHBHHAHBAAHBB-HBAAABHBHAHAHABBHBBHABHHBAHAAHAHHHABHHAHHHBHBHAHAAHBHABHHHBBAHBAHHH

*002440_00080488 HAHAAHHHHHHHABHHHHAHAHAHHHHAHAABBBHAAHBHHHHAAHHAAHHAHBAHHHHABAHHAHHA-HHAHAHHAAAHHAABHAHA-AHHHABHHHBHAHHHAHAHHHHAHHH-HBHBHHHA-HHHHHAHBHHHABBHHAAB

*000505_00182586 HHHHHHHBBHH-HBABHHBHAHBHHHHHHHBHAHHHHBHBBHBHHHHAABABHHBBAHAHHHBBHAHAAHBHHAAHHHHHHHHHABAHBHHHHHHAHABBHHAAHAAHAABHHABAHAHBHHAAHHAAHHHHHHAHHHBHABHH

*000694_00133360 HBHHHHABAHBHHHHHHHHAHHBAHHHHHHHHHHHAHAHHABHHAHHHHHABHHHBBHAAHHHHBHABHBBABAHBHHHBHAHBHAAAHBHBHHAAHHAABHAHHHBAHBBAAAHHHHHHBHBHAHAABHHHHABHHHAAAHHH

*004991_00027310 HBHBHHHBHHBHBBBHHHHHHHBAAHHHHHHHHHBHHABHHBHHAHHHHHABHBBBHBAHHHABHAABHHBHBABBHAHHBAHBHAAAHBHBHAHAHBAABBHHHHHAAHBAHAHHHHHHHHHHHHAHBHHHHAHHHHAHAAHB

*005655_00043081 HBHHHBHAHHAHHBHAHBBAABHBBHHHBHHHAAHHHAHHHHHABAABAAHBHHHHAHBHHHBHHAHBAAHHBAAHHHHAHABBHAHAHBHAAHHHAHBHAAHHHHAHHAHAHHHHHHBBHHAAHHHBHHHHBHHBHBHHHHBH

*006989_00006855 HBHHAAHHBBHHHBHHAAHHHHHHHHBBAHBHHBHHHAHHHHABHABHHHHHHBHHABBAHHAHHAHAHABBBHHHHAHAHHHHHHHHHHHABAHHAHHBAHAHHBHHHBBABHHHHHABHBAHHHBHHBHHBABBHABBHAAB

*000375_00397508 ABAHBHHHAHBHAHBHHHHHBHHHAHAHHHBHHAHAHHBBAHBHHAAHBHHHBHABHBHHHAHHABHHABBAHHHBHHBHAHHBAHHAAHHAAHHAHHHHBABAHBHBHBHBBAABHHHHBHHBBAHHBHHHBHHHHHHHHHBH

*000517_00234724 -BAHHBHAHAAAHH-HHHBAHAH--HAHAAAHHAHHHAAAHHHA-HAHAAAAHHAAAHHHHAHHHHHHAHHA-AHHAHHHHHA-AAHHHHAHHHAHHHHAAHAHAHAAHHAHHHAAH--HAAHAAHHBA-HHHAH-AHHHHA-A

*001301_00199459 HAHHHHHHABHBHBHHHHHHAHBHBHHAHAABHBHHHBBHHH-AHBHHAHHAAHHBHHHHBAHHHHHAHBHHHAHHAHHHHHBBAAHHHBHHHABAHAAHAHHHBA-HHAHHHHHB-BABAHHABHAAHHBHBHAAAHBAHAHB

*000622_00355347 HBHBHH-H-HHBHBHHBHHAHHHAHHHBBBHHHBHAHAHHBHHBABHBBB-HHHHHHHA-HBBHHHAHHHHAHAHH-HHBBAHHHAA--HBHHHA-HBAAHBAHHBHAHHHAAAHBAHHHHBHHAHAAHHHBBABBHHAAAHHH

*000239_00025826 HHBABHBHHHBBHAHAHHBHHHAHHHHHHHHHHHABHAHBAHBAHHBHAHHHHHBBHAAHHHHHHABHHHBBBHHHABHHBHAAHBABHBHAHBHHHBBHHHBHHABABHHHBHAHBHHHHAHHHBBHHHHHBABAHBHHABHB

*001682_00024252 BHHHAAAHHAHHBHHHHHHHABHHAHHABHBHAHBAHBBBBBBHHABHBHAHAHHBHBHBHHHHHHHHAHHBAAHHHAHAAAHHBHHAHHHHBHHBHAAHABABAHHHBHHAHHHAAHAHHBHAHHHHHHABHHBBHAHBHHHH

*001826_00159492 BHBABAHHBHHHHHHHHHAHAHHAABHBAHAHBAHAHHHHHHBBAAAHAHHAAHHAHHHAHABABHHBBHHHHHABABHHHHABHHHHHHAHHHHBBABABHHBHHAHHBBAHHHHHBAHBHHAHHHAHBHHHHHAHHAHHHBB

*003481_00064358 BHHHAAAHHHHHBHHHHHHBABHHAHHABHBHAHBAHBBBBBBHHABHBHAHAHHBHBHBHHHHHHHHHHHBAAHHHAHHAAHHBHHAHHHHBHHBHAAHABHBAAHHBBHAHHHAAHAHHHHAHBHAHHABHHBBHAHBHHHH

*000217_00599231 HBBBHHHHHHHBHBBHHAHHBHAHHHHHBBHHHHHBHHABHHHBHABBHBHHHBHHBHHHHHABBHHHHBBHBBAHAABHHBBBHAHHAHBBBABBHHHABBHHHAAHBHBHHHHA-AHHHHBBHHBHHAHHHHHHBAHBHHBH

*000603_00057210 HBHHAAHHBBHHHBHHAAHHHHHHHHBBAHBHHBHHHAHHHHABHABHHHHHHBHHABBAHHAHHAHAHABBBHHHHAHAHHHHBHHHHHHABHHHAHHBAHAHHB-HHBBABHAHHHABHBAHHHBHHBHHBABBHABHHAAB

*001821_00121716 HBHBHHHBHHB-BBBHHHHHHHBAAHHHHHBHHHBHHAHHHBHHAHHHHHABHBBBHBAHHHABHAABHH-HBABBHAHHB--B-AAHBBHBH-HAHBAABBHHHHHAAHBAHAHHHHHHHAHHHHAHBHHHHAHHHHAHAAHB

*002259_00230014 ABAHHHHHHHBAHHHHBHHHHHHHBHBHABHHHHHBBHAHBABHHHHBAHHHHBHAHHHHHBAHHHBAHHHHHABAAHAHHHBAHHHAHHHBABHBAHBHABHHAAHBHHHHAHABHHHAHAHHBHBHAAAAHHBAAAHAHHBA

*000022_00048645 HHBABHBHHABBBHHHHHBHHHAHHAHHHBBHHHABHAAHBHBAHHBHAHHHHHHBHAAHHHHHBAHHHHBBBHHH-BHABHBAHBHBHBHAHBHHHBHHHHBHHABABHHHBHABHHHHHAHHHBBHHAHHBABAHBHHHBHB

*003088_00106791 HHBHHBHHHHHBBHHHHHHBHHHHHAHHABHHHHHBHAAABHBAHHBHAHAHHHABHAAHHHHHBHAHHHBBBHHA-HHHBHBAHBHBHBHAHBHHABHHHHBAHABHHHHBBBABHHBHHAHHHBBBAAHHBABHBBHHHBHH

*000003_01349293 HAAHHHBABHHHHBBHBHHHHHBAHBBHBBAHBHBHHAHAAAHHABAHHHHHBHAHHBHHHHHAAHBAHAAHHHAHHABHHHAHBAHBHHHAHBHHHBBHHBAHBH-AHHAHHHABBHHHHBHHHHHAHH-BHAAHAHHAHAHH

*000037_00686996 HBHHHHABHHBHHHBHHHHAHHBAHHHHHHHHHHHAHAHHHBHHAHHHHHABHHHBBHAAHHHHBHABHBBABAHBHHHBHAHBHAAAHBHBHHAAHHAABHAHHHBAHBBAHAHHAHHHBHBHAHAABHHHHABHHBAAAHHB

*000076_00220097 AHHHBHBHHAHHBHHBHHHAAHHHHHAHBHBAAHHHHHAHHBHBHAHAABHHAABBAAAHAHBHHHAAHBAHHAHAHBHHHHHHHBHHBHHAHHHBHHHBHHAAHAHHHHBHHABABHHHAHAAHAAAAHHHBAHHHBBHHBHH

*000162_00197655 HAHAAHHBAHHBHBHHHHAHAHHHHAHAHAABBBHHABBHHHAAHBHHAHHAHHHHHHHHBAHHAHHAABHAHAHHAHHHHHBBHAHHHHHHHABAHAAHAHHHAAAHHHHAHHHBHBHBHHHABHHAHHAHBHAHAABAHAHB

*000465_00232420 AHAHHHHAAHBHHHHHBHBHHHHHBHBHAHHHHHHBBHAHBABHHHHBAHHHBBHAHHHHHBAHHHBHHAHHHABAAHAAHHBAHHHABBHBABHBAHBHHBBHAAHBHHHHAHABHHHAHAHHBHBHAHAAHHHHAAHAHHHA

*000480_00262667 HAHAAHHHHHBHABHHHHAHAHAHHHHAAAABBBHAAHBHHHHAHAHAAHHAHBHHHHHABAHAAHAABAHAHAHHAAHHHHHBHABHHHHHHABHHHBHAHHHAHHHHBHABHHAHHHBHHHABHHHHHAHBHHHAHBHBAAB

*000676_00173086 HBHHAHHHBHHHHBHHAAHHHHBHHHBHAHBHHBHHHAHHHHHBHABHHHHHHBHHABBAHHABHAHAHHBBBHHHBAHAHBHHHHHHHHHABAHAAHHBAHAHBHHHHBBABHAHHBHBHBAHAHBHHHBHBAHBHABBHAAB

*001240_00162140 HBAHHHBABHHHHBHHBHHHAHBAHBBBHBAHBHHHHAHAHHAHABAHHHHHHHAAHBHBHBHAHHHAHAAHHHHHHABHHHAHHAAHHHBAHBHHHBBHHBAABHHHBHAHHHHBBHHHHBHAAHHAHHBBHHAHAHHAAAHA

*001257_00111107 HAABAHHHHAHBAHHHHBABBAAHHHHHBHAHHBAHHHHBHHAHHAHBHAHBABHHHHHHHBHAHHAHHHBHHAHHHABAHHBBHBHHHHBHHHABHHBABABABHHBBAHHAAHAHHHHAHHBHHAHHBBAHAHHAHHHHHHB

*002939_00033717 HAHHHHHHHHHBHBHHHHBHAHBHAAHAHAABHBHHHBBHHHAAHBHHAHHAAHHHHHHBBAHHHHAAHBHHHAHHAHHHHABBHAHHHHHHAABAHAAHAAHBBAAHHAHHHHHBHBHBAHHABHAAHHBHHBAHABHAHAHH

*002988_00061698 ABBBHHHHHHHBHBBHHAHHHHAHHHHHBBHHHHHBHHABHBHBHABBHBHHHBHHBHHHHHAHBHHHHBBHBBAHAABHBBBBHAHHAHBHBABBHHHABBHHHAHHBHBHHHHAAAHHHHBHBHBHHAHHHHBHB-HBHABH

*002292_00012679 BHHHAAAHAHB-BHHHHHHBABHHABHHBHBBAHBAHBBHBBHHHA-HBHHHAHABHBHBBHHHHHHHHHHB-AHHHA-HAABH-HHHHHHABBHBBAAHABHBAHAHHH-HHHHA-HAHHHHAHBHAHH-BHABBH-HBHHHH

*000002_00406165 HHHAAHHHHHBHABHHHHAHAHAHBHHHAAHHBBHHAABHHBHHHAHAAHHAHBHHHHHAHAHAAHAABAHAHAHHAAHHHHHBHABHHHHHHABHHHBHAHAHAHHHHBHABHHAHHHBHHHABHHHHHAABABHAHBHBAAB

*000171_00253750 HHHHBHHBBAHHHBABHHBAAHBHHHHHHHBHAHHHHBHBBHBHHHHAABABAHBBAHAHHHBBHAAAHHHHHAHHAHHHHHHHABAHBHHHHHHAHABBHHAAHAHAAABHHABAHHHBHHAAHHAAHAHHHHAHHHHHABHH

*000596_00386661 HHBHBABBAHHHHAHAHHBHHAHAHHAHHHHHHHHBHAHBAABAAHBHAHHHBHBBHHHHHBHHHABHHBHBHHHHAHHHBAAHHHABHBHAHBHAHBBHHHBHHABAHBHHBHAHHHAHBAHHHBHHHHHBBHHAHBHBABHB

*000825_00017599 HHBHBBBHHHHBBHHHHABHHHAHHAHHHBHHHHABHAAHBHBAHHBHAHHHHHABHAAHHHHHBHAHHHBBBHHHABHHBHBAHBHBHBHAHBHHHBHHHHBAHABHBHHBBHABHHBHHAHHHBBBHAHHBABHBBHHHBHH

*001390_00037553 HHAHHHBABHHHHBBHBHHHAHBAHBBBBBAHBHHHHAHAAAHHABAHHHHHBHAHHBHHHBHAAHBAHAAHHHAHHABHHHAHHAHBHHBAHBHHHBBHHBAHBHHAHHAHHHHBHHHHHBHAHHHAHHBBHAAHAHHAHAHH

*001860_00049915 HHBHHBBHHHHBBHHHHABHHHAHHAHHHBHHHHABHAAHBHBAHHBHAHAHHHABHAAHHHHHBHAHHHBBBHHHAHHHBHBAHBHBHBHAHBHHHBHHHHBAHABHBHHBBHABBHBHHAHHHBBBHAHHBABHBBHHHBHH

*003882_00076270 HHBABHBHHHBHHAHAHHBHHAAHHHHHHHHHHHHBHAHBAHBAHHBHAHHHHHBBHAAHHHHHHABHAHBBBHHHABHABAAHHBABHBHAHBHAHBBHHHBHHABABHHHBHAHBHHBHAHHHBBHHHHHBHHAHBHHABHB

*000805_00013959 HBHHHHABAHBHHHHHHHHAHHBAHHHHHHHHHHHAHAHHHBHHAHAHHHABHHHBBHAAHHHHBHABHBBABAHBHHHBHAHBBAAAHBHBHBAAHHHABHAHHH-AHBHAAAHHAHHHBHBHAHAABHHHHABHHAAAAHHH

*001711_00165452 HHHHHHHBBAH-HBABHHBHAHBHHAHHHHBHAHHHHBHBBHBHHHHAABABHHBBAHAHHHBBHAHA-HBHHAAHHHHHHHHHHBA--HHHHHHAHA-BHHAAHA-BAABHHABAAAH-HAAAHHAAHHHHHHAHHHBHABHH

*000424_00421952 ABHBAHHHHHHHHHAHHBAHHBHHBHHAHAAHAAAHHHAAABHAHHBHAHAHHHHHBHHHHAABHHHAHABHBHBHHBHHBHHHHAAHHBHHHHBBHBHHBHHHBBABHAABBHABHHHBHBBHHBHAHHBHHHHAHHBAABHH

*001693_00106349 ABABBHHHAHBHAHBHHAHHBHHHAHAHHHBHHAHAHHBBAHBHHAAHBHHHBHABHBHHHAHHABHHABBHHHHBHHBHAAHBHHHAAHHAAHHHHHHHBAHAHHHBHBHBBAABHHHHBHHBBAHHBHHHBHHAHABHHHBH

*000101_00662738 HAHAABHHHHH--B-HHHAHAAAHB-HAHAABB-HAAB-HHHAAHHHH-HHAHH-HHHHABAHHAHHABB-AHAHHAAHHHHBBHAHHHAHHHABHHABHAHHHAA-HHHHAHH--ABHBHHHABHHHHHAHBHHHHHB-BAAB

*001336_00181906 HHBHBABBAHHHHAHABHBHHAHAHHAHHHHHHHHBHAHBAABAAHBHAHHHBHBBHHHHHBHHHABHHBHBHHHHAHHHBAAHHHABHBHAHBHAHBBHHHBHHABAHBHHBHAHHHAHBAHHHBHHHHHBBHHAH-HBABHB

*000651_00055953 HBHHHBAHHAHHHHHAHHBAABHBBHBHHBABHABHAAHAHHHHBHAHAAHHHHHHAHBHBABHHAHH-AHHHAAHHHHHHABBHAHHHBHAAHAHABHHHAHHHH-HBAHABHAHABBHBHAAHHHBHBHBHHHBHBHHHHBH

*000212_00035391 HBHHAAHHBBHHHBHHAHHBBHHHHHBBAHBHHBHHAAHHHHAHHABHHHHHHBHHABBAHHAHHAHAHABHBHHHHAHAHHHHBAHHHHHABAHHAHHBHHAHHBHHHBBABHAAHBABHBABHHBHHBHHHHBBHHBBHAAB

*000480_00258748 HAHAAHHHHHBHABHHHHAHAHAHHHHAAAABBBHAAHBHHHHAHAHAAHHAHBHHHHHABAHAAHAABAHAHAHHAAHHHHHBHABHHHHHHABHHHBHAHHHAHHHHBHABHHAHBHBHHHABHHHHHAHBHHHAHBHBAAB

*000822_00018070 HBAHHHBABHHHHBHHBHHHAHHAHBBBHBAHBBHHHAHAHHAHABAHHBBHHHAAHBABHBHAAHHAHAAAHHHHBAHHHHAHHAHHHHBAHBHHHBBHHBHABHHHBHAHHHBBHHHHHBHAAHHAHHBBHHHHAHHAHAHA

*001100_00133913 HBHBHHHBHHBHBHBHHHHHHHBAAHHHHHHHHHBHHABHHBHHAHHHHHABHBHBBBAHHHABBAABHHBABABBHHHBBAHBHAAAHHHBHHHAHBAABBHHHHHAHHBAHAHHHAHHHHBHAHAAHHHHHABHHHAHAAHB

*001120_00138488 HBAABAHAHBHBAHAHHHABBHHHHHABAAAHHBBHBHHBAAHHHHHBHHHHBHBAHHAHHABBHBHABHBHHHAHAHBAHHABHBBHHHHHAHHBHAHHHHAAHAHHHBHHBHHHHHAHHHHBHHBBHHHHBHHHHBAABHHB

*001120_00211988 HBAABAHAHBHBAHAHHHABBHHHHHABAAAHHBBHBHHBAAHHHHHBHHHHBHBAHHAHHABBHBHABHBHHHAHAHBAHHABHBBHHHHHAHHBHAHHHHHAHAHHHBHHBHHBHHAHHHHBHHBBHHHHBHHHHBAABHHB

*008651_00012008 HAHAAHHHHHBHABHHHHAHAHAHBHHAAAABBBHAAHBHHHHAHAHAAHHAHBHHHHHABAHAAHAABAHAHAHHAAHHHHHBHABHHHHHHABHHHBHAHHHAHHHHBHABHHAHHHBHHHABHHHHHAHBHHHAHBHBAAB

*000498_00174907 ABAHHHAHHHHHAH-HBHHHHHHBBABHABHHHHHBHHAHBA-HBHHBAHHHHBBAHBHBHHAHHHBHHHHHHHBH-HAHHBBAHBHAHHHBABBBAHBHABHH-A-BAHHHAHA-AHHAHAHHBHBAAHHAHH-AA-HHHHBA

*000454_00343213 -HHHH-A-A-H-AHBAH-HAAAAHA-H--HHAHHHH-AAH---HHHAHHHAHHHH-HAHHHHHHAHB---A--HA-AHHHAAH--H-AA-H-HHAHA-H-AAAHA-ABAH-A-HA---AA----AAHA-B-HAHHHHAHAHHHA

*001821_00122705 HBHBHHHBHHBHBBBHHHHHHHBAAHHHHHHHHHBHHAHHHBBHAHHHHHABHBBBHBAHHHABHAABHHBHBABB-AHHBAHB-AAABBHBHHHAHBAABBHHHHHAAHBAHAHHHAHHHHHHHHAHBHHHHAHHH-AHAAHB

*002968_00052693 HAHAAHHHHHBHABAHHHAHAHAHHHHAHAABBBHAAHBHAHHAHAHAAHHAHBAHHHHABAHHAHHABHHAHAHHAAHHHBHBHABHHHHHHABHHHBHAHHHAB-HHHHAHHHHHBHBHHHA-HHHH-AHBHAHABBHBAAB

*000638_00137985 HHHHHAHBBHHHHBABHHBHAHBHHHHHHHBHAHHHHBHBBHBHHHHAAHABHHBHHHAAHHBBHAHAAHBBHAAHAHHBHHHHHBAHBHHHHHHAHABBHHAAHAHHAABHHABAHAABBHHAHHAAHHHHHBAHHHBBABHH

*001448_00123569 HHHHAAAHHAHHHHHHHHHHABHHAHHABBBHAHBAHBBHBBBHHAHHBAABAHHBHHBBHAHHHBHHAHBHAABHHHHHAABHBHHAAHAHBHHBHAHHABAHAHHHHHHAHHAAAHAHHBHAHAHHHHABHHHBBAHHHHHH

*001957_00050548 BHAHHAHHHHBHHHAHAAHBBHHHHHHBHHHHBHBBAHHBHAHAHAHBHHBBHHHHHBHHHAAHAHAHHHBABHHBAAHBHBHBHAHHABBBBAHHHAHABHHAHHAHHABHHHHHHAABABHBAABHHHHAHAABHHHHHHBH

*000681_00242878 ABHHHHBHHAAHHABBHABAAAHHAHAHBABBAABHHHAHHHHHHHAHHBBAHHABHHBHBAHAABHHAHHAAAHHHBHBHHHHBHHHAHHAAHHBBHBHHHAAHBBHHHAHHAHHHHHAHHHAHBHHHBHAHAAHHBHHHHAH

*000037_00797938 HHHHHHABHHB-HHBHHHHHHHBHHHHHHHHHHHHAHABHHBHHAHHHHHABHHHBBHAAHHHHBHABHBBABAHB-H-BHAHBHAAAHBHBHAAAHHAABHAHHH-AHBBAHAHBAAHHBHBH-HAABHHHHABAHHAAAHHB

*000854_00183348 -HBBBH-H--HH-A-AHHBHHAH-H-HHHHHHH-HBH-HB-ABAHHBHAHHHH---HHHHHHAHHABHHBBBH-HB-BAAB-AHH-ABB-HAH-H-H-BHHABHHA-A-HAHBHAH-AHBHAHH-BBHHA-HBAHAH-HB-BHB

*006388_00026908 HHHHAHHAHAHAHHAHHHHHAHHHHHHAHBBHAHBAHHHHHBHHHHHHHHHHBHBBHHHHHABHHBHHAHBHAHBB-HBAAABH-HABAHABB-BBHABAHBAHHAAAHHHABHBAHABHBBHAHAAHBHHBHHHHBAHHAHHH

*000219_00264353 ABHBBH-HAHBAAHBHHHHHBHHHAHAHHHBHHAHAHHHB-HBHHHAHBHHHBHABHBHHHABHABHH-B-HHHHBHHBHAAHBHBHAAHHAABHHHHHBBHAHHHHHHBBBBAABHHHBBHHBBAHHBHBHBHAAH-BHHHBH

*000761_00080827 HHHHHHHBBHHHHBABAHBHAHBHHHHHHHBHAHHHHBHBBHBHHHHAABABHHBBAHAHHHBBHAHAAHBHHAAHHHHHHBHHBBAHBHHHHHHAHABBHHAAHAHHAABHHABAHA-BBHAAHHAAHHHHHHAAHHBHABHH

*000761_00137525 HHHHHHHBBHHHHBABHHBHAHBHHBHHHHBHA-HHHBHBBHBHHHHAABABHHBBAHAHHHBBHAHAAHBHHAAHHHHHHHHHHBAHBHHHHHHAHABBHHAAHAAHAABHHABAHAHBBHAAHHAAHHHHHAAHHHBHABHB

*000831_00100028 HHHHHHHAHAHAHHBHHHAHAHHHHHHAHBBHAHBAHHAH-BHHHHHHBHHHHHBBHHHHHABHHBHHAHBAAHBHHHBAAABHBHAHAHABBHBBHABHHBAHAAHAHHHABHHAHHAHBBHAHAAHBHABHHHBBAHBAHHH

*000055_00189092 HBHBHHHBHHBBBBBHHHHHHHBAAHHHHHHHHHBHHABHHBHHAHHHHHABHBBBHBAHHHABHAABHHBHBABBHAHBBAHBHAAAHBHBHHHAHBAABBHHHHHAHHBAHAHHHAHHHHHHHHAHBHHHHABHHBAHAAHB

*000075_00513497 AHHBHHBHAHBHAHAHBHBHHHHHABBBBHHBAHHHHHABHHAHHBHHABAAHHAHBBAHBHHHBHBHBHAAHAAAAHAHHHHBHAHHHHAAAHHHBAHHAAHHHBHHHABAHHHHBAHHHHBHHBAHABHHHHBABHHHHAHH

*000075_00515412 AHHBHHBHAHBHAHAHBHBHHHHHABBBBHHBAHHHHHABHHAHHBHHABAAHHAHBBAHBHHHBHBHBHAAHAAAAHAHHHHBHAHHHHAAAHHHBAHHAAHHHBHHHABAHHHHHAHAHHBHHBAHABHHHHBABHHHHAHH

*000269_00238416 AHHBHAHHAHHHAHHHBHBBHHHHABBBBHHHHHHHHBABHAAAHBHAHBAAHHABHBHBHHHHBHBHBHAABAHAHAAHHHHBHBAHHHAAAAHHHAHHHAABHBHHHABAHHHHHAAHHHHHHBAHABHHHHHABHHHAAHH

*000272_00209996 HBBBHBHABHABAHHAHAHHHHHBHHHHAHAHBBHHHAHHBHHBBAHHHBHAHBHHHHHHHAHHAHBBHHHHHHHHHAAHHHHBABHBBHHHHABHABHHHAHHAAHABHBHHBHAHABHHAHHHHHBHBABAABHBBHBHAAB

*000283_00398853 HBHHHBHAHHAHHBHAHBBAABHBBHBHBHHHAAHHHAHHHHHABAHBAAHBHHHHAHBHHHBHHAHBAAHHBAAHHHHAHABBAAHAHBHAAHHHAHBHAAHHHHAHHAHAHHHHHBBBBHAAHHHBHHHHBHHBHBHHHHBH

*000480_00300205 HAHAAHHHHHBHABHHHHAHAHAHBHHAAAABBBHAAHBHHHHAHAHAAHHAHBHHHHHABAHAAHAABAHAHAHHAAHHHHHBHABHHHHHHABHHHBHAHHHAHHHHBHABHHAHBHBHHHABHHHHHAHBHHHAHBHBAAB

*000725_00188168 HBHBHHHHHHHBHBBHBAHHHHHHHBBHBBHHHHHBBHABHBHBHABBHBHHHBHBBHHHHHAHBHHAHBHHBBAHAABHBHBBHHHHAHBHBABHHHBABBHHHABHBHBHHHHAHHHHAHBHHHBBHAHHHBBHBAHBHABH

*000753_00326601 HBAHHHBABBHHHBHHBHHHHHHAHBHBBBAHBBHHAAHAHHAHABABHBBHHHAAHBABHBBAAHHHHAHAAHHHBAHHHHAHHAHHHHBHHHHHHBBAHBHAHHHHBHHHHHHBBHHHBBHHAHHAHHHBBHBBAHHAHAAH

*000824_00054369 HBAABAHAHBHBAHABHHHBBHHHHHABHAAHHBBHBHHBAAHHHHHBAHBHBHBAHHAHHABBHBHABHBHAHAHAHBAHHABHBBHHHHHAHHBHAHHHHHAHHHHHBHHBHHBHHAHHHHBHHBBHHHHBHHHHBAABHHB

*000824_00080866 HBAABAHAHBHBAHABHHHBBHHHHHABHAAHHBBHBHHBAAHHHHHBAHBHBHBAHHAHHABBHBHABHBHAHAHAHBAHHABHBBHHHHHAHHBHAHHHHHAHHHHHBHHBHHBHHAHHHHBHHBBHHHHBHHHHBAABHHB

*001098_00131853 AHHHBAAHAAHAHHHHHHHHHHHHAHHABBBHHAHAHHHBHHHHHHAHHHHHBAABHHHHHABAABHHABBHAHHBHAHHAABBAHAHAHHAAHHBHHBHBBHHHHABHBHABHAHAHAHBBHHBAAHBHHHHHHABABHAHHH

*001386_00063504 HBHHHBHAHHAHHBHAHBBAABHBBHHHBHHHAAHHHAHHHHHABAABAAHBHHHHAHBHHHBHHHHBAAHHBAAHHHHAHABBHAHAHBHAAHHHAABHAAHHHAAHHAHAHHHHHBBBHHAABHHBHHHHBHHBHBHHHHBH

*005969_00014010 BBHHHBAHHAAHHHHAHHBAABHBBHBHHBAHHAHHHAHAHHHHBAAHAAHBHHHHAHBHHABAHAHHAAHHBAAHHHHHHABBAAHHHBHAHHAHAHHHHAHHHHAAHAHAHHHHABBBHHAAHHHBHBHBBHHBHBAHHHBH

*002161_00068480 ABHHAHHHHBHHHHAHHBABHHHHBHHAHAAHAAAHBHAAABHAHHBHAHAHHHHHBHHHHAABHHHAHABHBHBHABHHBHHAHAAHHBHHHHBBHBHHBHHHBB-BBAABBHHBAHHBHHBHHBHHHHBHHHHAHHBAABHH

*002747_00004918 ABHAHHHAA-AB-HAHHHHHAHAHHHHHAH-HHAAHHHAHAHAHHHHHHHHAHHHHAHHAHAAAHAHH-AHAAHHAAHHAH-AHBAAA-HAHBBAHHHHBAAAHHAAHHHAAAHA-HHAAHHHAAHBHAHAHHAHAHAHHAAHA

*003481_00065206 BHHHAAAHHHHHBHHHHHHBABHHABHABHBHAHBAHBBBBBBHHABHBHAHAHHBHBHBHHHHHHHHHHHBAAHHHAHHAA-HBHHAHBHHBHHBHAAHABHBABHHBBHAHHHAAHAHHHHA-BHAHHABHHBBHAHBHHHH

*000022_00120600 HBBABHBHHHBB-HHHHHBHHHAHHAHHHBBHHHABHAAHBHBAHHBHAHHHHHHBHAAHHHHBBAHHBHB-BHHHABHABHBAHBHBHBHAHBHHHBHHAHBHHA-ABHHHBHABBHHHHAHHHBBHHAHHBABAHBHHHBHB

*000022_00563452 HHBABHBHHHBBBHHHHHBHHHAHHAHHHBBHHHABHAAHBHBAHHBHAHHHHHABHAAHHHHBBAHHHHBBBHHHABHABHBAHBHBHBHAHBHHHBHHHHBAHABHBHHBBHABHHHHHAHHHBBHHAHHBABAHBHHHBHB

*000234_00224993 HHHHAAAHHAHHHHHHHHHHABHHAHHABBBHAHBAHBBHBBBHHAHHBAABAHHBHBBBHAHHHHHHAHHBAABHHHHAAABHBHHAAHHHBHHBHAAHABAHAHHHBHHAHHAAAHAHHBHAHHHHHHABHHBBHAHHHHHH

*001860_00113046 HHBHHBBHHHHBBHHHHABBHHAHHAHHHBHHHHABHAAHBHBAHHBHAHAHHHABHAAHHHHHBHAHHHBBBHHHAHHHBHBAHBHBHBHAHBHHHBHHHHBAHABHBHHBBBABBHBHHAHHHBBBHAHHBABHBBHHHBHH

*001190_00018793 BBHHHBHAHHAHHHHAHBBAABHBBHBHBHAHAAHHHAHHHHHABAABAAHBHHHHAHBHHHBAHAHBAAHHBAAHHHHAHABBHAHAHHHAAHHHAHBBAAHHHHAHHAHAHHHHHBBBBHAAHHHBHHHHBHHBH-HHHHBH

*001386_00165869 HBHHHBHAHHAHABHAHBBHABHBBHHHBHHHAAHHHAHHHHBABAABAAHBHHHHAHBHHHBHHHBBAAHHBHAHHHHAHABBHAHAHBHAAHHHAABHAAHHHAAHBAHAHHHHHHBBHHAABHHBHHHHBHHBH-HHHHBH

*000130_00318949 ABHBHHHHBHABHBBHBHHHHHHHHBBHBBHHHHHBBHABHBHBHABBHBHHHBHBBHHHHHAHBHHAHBHHBBAHAABHBHBBHHHAAHHHBABHHHBAHBHHHABHBHBHHHHAHHHHAABHHHBBHAHHHBBHBAHHHABH

*000424_00153786 ABHBAHHHHHHHHHAHHBAHHBHHBHHAHAAHAAAHHHAAABHAHHBHAHAHHHHHBHHHHAABHHHAHABHBHBHHBHHBHHHBAAHHBHHHHBBHBHHBHHHBBABHAABBHABHHHBHBBHHBHAHHBHHHHAHBBAABHH

*000424_00422442 ABHBAHHHHHHBHHAHHBAHHBHHBHHAHAAHAAAHHHAAABHAHHBHAHAHHHHHBHHHHAABHHHAHABHBHBHHBHHBHHHHAAHHBHHHBBBHBHHBHHHBBABHAABBHABHHHBHBBHHBHAHHBHHHHAHHBAABHH

*001207_00164200 ABBABABABHBHHAHAAHHBAHBHHHAHHAHHHHBHAHHHHAHHHHHHAAHHHAAHHHHBHHHHHHHHHHABBHBBBABHHBHHHBBHABHHAAHHHHBBAHHHHBBHHHHABBBBHHAAAHHABHHBHBHAHHBBAHHBBHHH

*001749_00110181 ABHHBAAHAAHAHHHHHHHHHHHHAHHABBBHHAHABHHBHHBHHHAHHHHHBAABHHHHHABAABHHABBHAHHBHAHHAAHBHHAHAHHAAHHBHHBHBBHHHHABHBHABHAHAHAHBBHHBAAHBHHHHHHABABHAHHH

*003483_00027342 ABHHAHHHHBHHHHAHHBABHHHHBHHAHAAHAAAHBHAAABHAHHBHAHAHHHHHBHHHHAABHHHAHABHBHBHABHHBHHAHAAHHBHHHHBBHBHHBHHHBBABBAABBHHBHHHBHBBHHBHHHHBHHHHAHHBAABHH

*000003_00222132 HHAHHHBAHH-HHBBHBHHHHHBHHBBHBBBHBABHHAAAAABHHBHHHHHABHABABHHHHHAAHBAHAAHAHAHHHBHHHHHAAHBHHHAHBHHH-BHHBAHBH-AHHAHHHHBBHHHHBAHAHHAHABBBAAHAHHAHHHH

*006676_00005292 AHBHHBHHAHAHBHBHBHHHHBHHAAHAHHHHAHHBBBAAHH-HHABHHAAHBHAHAHAAHBBHHHAHAHBHHBHA-AHHBHBHHBHBHHBAHBHHHBHHHHHAAAHBAHHBABABHHBHHHAHBHHBH-AHBHHHB-HHHBHA

*000002_00495805 HHHAAHHHHHB-ABHHHHAHAHAHBHHHAAHHBBHHHABHHBHHHAHAAHHAHBHHHHHAHAHAAHAABAHAHAHHAAHHHHHBHABHHHHHHABHHHBHAHAHAHHHABHABHHAHBHBHHHABHHHHBAABABHAHBHBAAB

*002684_00082660 BBHHHBHAHHAHHHHAHBBAABHBBHBHBHAHAAHHHAHHHHHABAABAAHBHHHHAHBHHHBAHAHBAAHHBAAHHHHAHABBBAHAHBHAAHHHAHBHAAHHHH-AHAHAHHHHHHBBHHAA-HHBHHHHBHHBHBHHHHBH

*000040_00387150 AHHAHHAHHHAHHBHHHBBHABBHHBAABAHBAAAHAHHHHHHAHHHBAAHHBBHHABAHBBBHHHHBAHAHHBHBBHAHHABHHHHAAHHAHHAHAABHHAHHHAAHBHHAHBHAHHHBHHAAHHHHBHHHBHHBHBBHHAHH

*000040_00603861 AHHAHHAHHHAHHBHHHBBHABBHHBAABAHBAAAHAHHHHHHAHHHBHAHHBBHHABAHBBBHHHHBAHAHHBHBBHAHHABHHHHAAHHAHHAHAABHHAHHHAAHBHHAHBBHAHHBHHAAHHHHBHHHBHHBHBBHHAHH

*000382_00319998 AHAHABBHAHHHAAHHHHBBHHABHHBBHAHAHHHAHBHHHHHBBBHHAHAHHHHBHHAAAHHHHHABBHBHHHAHHAHHHHBHHHHHBHHBHAAAABHHAHAHHAHHHHHAAHHHBABBABAHHAAAAHHHBBHABHAAHHHA

*000892_00155186 HBHHHHAHHHHHAHHBBBHHHBABHHBHABHHHHHBHHAABHBHBHHBAHHHHBHAHBBBHAHBHHHHHHHHHHHAHHAHHABAABHAHHHBHHHBAHBHABBHHAHHAAHAABAHHAHAHAHHBHHAAHHAHHBAAAHBHABA

*000937_00017677 BBHHHBHHHAAHHHHAHHBAABHBBHBHHBAHAAHHHAHAHHHHBAHHAAHBHHHHAHBHHABAHAHBAAHHBAAHHHHHHABBAAHAHBHAAHHHAHHHHAHHHHAAHAHAHHHHHBBBBHAAHHHBHBHBBHHBHBAHHHBH

*002032_00174733 HBHHAAHHBBHHHBHHAAHHHHHHHHBBAHBHHBHHHAHHAHABHABHHHHHHBHBABBAHHAHHAHAHABBBHHHHABHHHHHHHHHHHHABAHHAHHBAHAHHBAHHBBABHAHHBABHBAHHHBHHBHHBABBHABHHAAB

*002684_00118127 BBHHHBHAHHAHHHHAHBBAABHBBHHHBHAHAAHHHAHHHHHABAABAAHBHHHHAHBHHHBAHAHBAAHHBAAHHHHAHABBHAHAHBHAAHBHAHBHAAHHHHAAHAHAHHHHHBBBHHAAHHHBHHHHBHHBHBHHHHBH

*003341_00087067 HBAHHHBABBHHHBHHBHHHHHHAHBHBBBAHBBHHAAHAHHABABABHBBHHHAAHBABHBBAAHHHHAHAAHHHBAHHHHAHHAHHHHBHHHHHHBBAHBHAHHHHBHAHHHHBHHHHBBHHAHHAHHHBBHBBAHHAHAAH

*003662_00002276 HBHBHAHBHBBHHBAHHAHHBHAABAHBHBHHHHHBHHHBHAHBAHHBBBBHHHBHHHHAHAABHHHHHBBHHHAHHHHBHBHBHABHABBBBAHBHHHABBHHHHAAHABHHHHHHAABHHHBAABHHAHHHAHHHAHHBHBH

*004369_00035386 HBHBHAHBHBBHHBAHHAHHBHAABAHBHBHHHHHBHHHBHAHBAHHBBBBHHHBHHHHAHAABHHHHHBBHHHAHHHHBHBHBHABHABBBBAHBHHHABBHHHHAAHABHHHHHHAABHBHBAABHHHHHHAHHHAHHBHBH

*005135_00007639 HBHBHBHAAHHHABHBHBHBHHHHAHABHAAHHBHAHAHAAHHHHHHHHHHBHBBBHBBBHHAHHHAAHHBHBABHBAHHBHHBHAHABAHHHHBAHHABHBHHAHBHHHBABABHHABHHHHHHHAHHBABHHHHHHABHAHB

*000108_00173831 BBHAHBHBAABHHHBBBHHHHABAHBHHBABHHBHHABBBBHAHHHBA-AAABABBHHAHHBHHHAHA-HAHHBBHBHHHAA-ABAHHHHAHHAAHHHAHHBHHBHAHAAHHHAH-HHHHHHHHBHHB-HBHHAH-HHHHHBHA

*000209_00021768 HHBHABHABHABAHHHHHHBHHHBHHABHHHABBHHHBHHBHHBBAHBHBHAHBHHBHHHAABHAHHHHHHHHHHHHAAHHHBHABHBBAAHHAHHABHBBAHAHAHABBHBBHHAHABHHABHHHHBHHHBHHBHBBHHHAAB

*000621_00297050 HHHHHHBAAAHAHHAHHHHHAHHHHHHAHBBHAABAHHBHHBHHHHHHHHHHBHBBHHHHHABHHBHHAHBHAHBBAHBAAABHHHAHAHABBHBBHABHHBAHHAAAHHHABHHAABHHBBHAAAAHBBHBHHHHBAHHAHHH

*001125_00079470 BHABHAHHHHBHHHAHAAHBBHHHHHHBHHHHHHBBAHHBHAHAHAHBHHBBHHHHHBHHHAAHAHAHHHBABHABAAHBHBHBHAHBABBBBAHHHAHABHHAHHAHHABHHHHHHHABABHBAABHBAHHHAABHHHHHHBH

*001583_00157953 HHBAHAAHHHAHAAHHABHHHHAHHBAHAAHAHABBHHBAHHHHHHHBHHBABHAHBABHAHBHHHBHHAHHHBBHBBHHHABBHAHAAHHHHHBHHHBHHHHHHHBHBAHBBHAHAHAABHBHAHHBHBHHHHBABAHHHHAB

*002127_00104679 HHHHBHAHAAHAHHBHHBHHAHHHAHHABBBHHHBAHHHBHBHHHHAHHHHHBAABHHHHHABAABHHAHBHAHHBHHHHAABBAHAHAHABAHHBHABHBBAAHHABHHHABHAAAHHHHBHABAAHBHHHHHHHBAHHHHHA

*000370_00233665 AHAAHHBHHHH-ABHAHHHHBHAAHHAHAHBHHHHAHBBHAHHHAHHBBHBHHHABHBBHHAHAHBHHAHHHHHBBAHBBHHHBAHHAAHHAAHAAAHAABAHHHHBHHHHBHAABBHHBBHHBHHAABHHHHAHBHBHHHABH

*002549_00042426 BBHHHBHHHAAHHH-AHHBHABHBBHBHHBAHAAHHHAHAHHHHBAAHAAHBHHHHAHBHHABAHAHBAAHHBAAHHHHHHABBAAHAHBHAAHHHAHHHHAHHHHAAHAHAHHHHHBBBHHAAHHHBHBHBBHHBHBAAHHBH

*000003_01206319 HHAHHHBABHHB-BBHBHAHHHBAHBBHBBAHBHBHHAHAAAAHABAHHHHHBHAHHBHHHHHA-HB--A-HHHAHHHBHHAAHHAHBHHHAHBHHH-BHHBAHBHBAHHAHHHHBBHHHHBHHHBHAA-BBHAA-A-HAHAHH

*000122_00403393 ABAABHBHAHBHABHHHAHHBHAHHHAHHHBBHHHABHBHAHHHHAHHBHHHBHABHBHHBAHHABHHABHAHHHBHHBBAHHBHHHAAHBAHHHAHHAHBABAAHHBHBHBHAABBHHHBHHBBHAHBHAHBHBHHHHHHHBH

*000130_00428930 ABHBHHHHBHABHBBHBHHHHHHHHBBHBBHHHHHBBHABHBHBHABBHBHHHBHBBHHHHHAHBHHAHBHHBBAHAABHBHBBHHHHAHBHBABHHHBAHBHHHABHBHBHHHHAHAHHAABHHHBBHAHHHBBHBAHHHABH

*000130_00462292 ABHBHHHHBHABHBBHBHHHHHHHHBBHBBHHHHHBBHABHBHBHABBHBHHHBHBBHHHHHAHBHHAHBHHBBAHAABHBHBBHHHHAHBHBABHHHBAHBHHHABHBHBHHHHAHAHHAABHHHBBHAHHHBBHBAHHHABH

*000274_00415703 ABHBHHBHHHABHBBHBHHHHHHBHBHHBHHHHAHHBHABHBHHBHBHABHHABHBBHBHHBHHHHHAHBHABBAHHAHHBHHBHHHAHHBHBHHBHHBAHBHAHABBBHBAHHAABHHHHABHHHBBHAHHHBBHBAHHHABA

*000681_00247340 ABHHHHBHHAAHHABBHABAAAHHAHAHBABBAABHHHAHHHHHHHAHHBBAHHABHHBHBAHAABHHAHHAAAHHABHBHHHHBHHHAHHAAHHBBHBHHHAAHBBHHHAHHAHHBHHAHHHAHBHHHBHAHAAHHBHHHHAH

*000900_00133924 ABAAHHBHHHBHABHHHHHHBHAAHHAHAHBHHHHAHHBHAHHHAHHBBHBHHHABHBBHHAHAHBHHABHHAHBBHHBBHHHBAHHAAHBAAHAAAHAHBAHAHHHHHHHBBAABBHBBBHHBHHAAHHHHBHHBHHHHHABH

*006689_00024524 HHBHHBHBBBHHHHHBHBBHBBHBBBBBBHBHHBBHHBHHBHBHHBHHBHBBHHHBHBHHHBHHHHHHBHHBBHHHBHHHBHHHBBHBHBHBHHBBHBHHBHHHBBBBHHHBHHBHBBHBHHBBBBHHBBBHBHHBHBHHBBBH

*000068_00078918 HHHHHHHHHAHAAHHHHHHHAHHHHHHAHBBHA-BAHHBHHBBHHHHHBHHHHHBBHHHHHAHAHBHAAHBAAHBBAH-AAABH-AHHAHABB-HBHABHABAAAAHAHHAABHHAHHHHBBHAHAAHBHABHHHBBAH-AHHH

*000329_00395003 HBHHHHHAHAHAHH-HHBHHAAHBHHHAHBBHAHBAHH-HHBBHHHHHHHHHHHB-HHHHHABHHBHHAHBAAHBBHHBAAABHAHAHAHABBHBBHABHBBAHHAHAHHHABHHAHHB-BBHAHAAHBBHBHHHBBAHBAHHH

*000144_00364419 HHBAHAAHHHAHAAHHABHHHAAHHBAHAAHAHABBHHBAHHHHHHHBHHBABHAHBAHHAHBHHHBHHAHHABBHBBHHHHBBHAHAAHAHHHBHHABHHHHHHHBHBAHBBHHHAHAABHBHAHHBH-HHHHBABAHHHHAB

*000306_00437567 BHHAAAAHHHBHBHHHHHHBABHHAAHHBHBBHHBAHBBHBBHBHABHBHHHAHABHBHBBBHBHHHH-HHBAAHHAAHHAAHHBHHHHHHABABBBAAHABHBAHBHHHHHHHHAAHAHHHHAHBHAHHABHHBBHHHBHHHH

*002781_00019515 HHBAHAAHHAAHAAHHABHHHHAHHBAHAAHAHHBBHHBAHHHHHHHBHHBABHAHBABHAHBHHHBHHAHHHBBH-BHHHABBHAHAAHHHHHHHHHBHHHHHHHBHBAHBBHAHAHAABHBHAHHBHBHAAHBABAHHHHAB

*006939_00002717 HHBAHAAHHHA-AAHHABHHHHAHHBAHAAHAHABBHHBAHHHHHHHBHHBABHAHBABHAHBHHHBHAAHHHBBHBBHHHABBHAHAAHAHHHBHHHBHHHHHHHBHBAHBBHHHHHAABHBHAHHBHBHHHHBABAHHHHAB

*000040_00284820 AHHAHHAHHHAHHBHHHBBAABBHHBAABAHBAAAHAHHHHHHAHHHBAAHHBBHHABAHBBBHHHHBAHAHHBHBBHAHHABHHHHAAHHAHHAHAABHHAHHHAAHBHHAHBHAAHHBHHAAHHHHBHHHBHHBHBHHHAHH

*000090_00572643 HBHHHHABHHBAHHBHHHHHHBBAHHHHHHHHHHHAHABHHBHHAHHHHHABHHHBBBAHHHHHBHABABBABAHBHHHBBAHBAAAAHBHBHHAAHHAABHAHHHBAHBBAHAHHAAHHHHBHAHAABHHHHABHHHAAAHHB

*000351_00153961 AHHBHAHHAHHHAHHHBHBBHHHHABBBBHHHAHHHHBABHAAAHBHAHBAAHHABBBHBHHHHBHBHBHAABAHAHHAHHHHBHBAHHHAAAHHAHAHHHAHBHBBHHABAHHHHHAAHHHBHHBAHABHHHHHABHHHAAHH

*000783_00187868 HBHHHBHAHHAHHBHAHBBHABHBBHHHBHHHAAHHHAHHHHAABAABAAHBHBHHAHBHHHBHHHBBAAHHBHAHHHHAHABBHAHAHBHAAHHHAABHAAHHHAAHBHHAHHHHHBBBHHAABHBBHHHHBHHBHBHHHHBH

*001134_00119292 HBAHHHBABHHHHBBHBHHHAHBAHBBBHBAHBBHHHAHAAHAHABAHHBHHHHAAHBHBHBHAAHHABAAHHHHHHABHHHAHHAHBHHBAHBHHHBBHHBAABHHHBHAHHHHBBHHHHBHHAHHAHABBHHAHAHHAHAHH

*001148_00228355 AHAHAHBHAHHAHAHHHHBBHHABAHBBBAHAHHAAHBHHHHHBBBHHAHAHHHHBAAAAAHHHHHABBHBAHHAHHAHAHHBHHHHHBHHHHAAAABHHAHAHHAHHHHBAAHHHHAHBABAHHAHAHHHHBBHHBHAAHHHA

*001710_00020473 AHHHHHHAAHBHHHHHBBBBHHHABHBHAHHHAHAHBAAHBABHAHHBAHHHBBBHHHHHHBAHHABHHAHHHAHAAHAAABBHHBHABBHHHBHBAHBHHBHHAAHBBHHHAHABHHHABAHHBHBHAHAAHHHHHAHHHHHA

*002146_00047536 BBHHHBHHHAAHHHHAHHBAABHBBHBHHBAHAAHHHAHAHHHHBAAHAAHBHHHHAHBHHABAHAHBAAHHBAAHHHHHHABBAAHAHBHAAHHHAHHHHAHHHHAAHAHAHHHHHBBBBHAAHHHBHBHBBHHBHBAHHHBH

*002369_00059449 AHBHHHHHHHHBBHHHHHHBHHAHAAHHABHHHHABHAAAHHBHHHBHAHAHHHABAAAHHHHHHHAHHHBHBHHAAHHHBHBAHBHBHBHAHBHHHBHHHBBAAABHHBHBHBABHHBHHAAHBBBBAAAHBABHBBHHHBHH

*003220_00029126 HBHBHAHBHBBHHBAHHAHHBHAABAHBHBHHHHHBHHHBHAHBAHHBBBBHHHBHHHHAHAABHHHHHBBHHHAHHHHBHBHBHABHABBBBAHBHHHABBHHHHAAHABHHHHHHAABHBHBAABHHAHHHAHHHAHHBHBH

*003374_00130529 HBHBHAHBHBBHHBHHHAHHBHAABAHBHBHHHHHBHHHBHAHBAHHBBBBHHHBHHHHAHAABHHHHHBBHHHAHBHHBHBHBHABHABBBBAHBHHHABBHHHHAAHABHHHHHHAABHBHBAABHHAHHHAHHHAHHBHBH

*005720_00027504 HBHBHBHBAHHHHBBBHBHBHHHHAHABHAHHHHHHHAHAAHAHHHHHHHABHBBBHBBBHAAHHAAHHBBHBABHHAHHBHHBHAHABHHHHHBAHBABHBHHAHBHHHBAHABBHABHHHHHHHAHHBABHHHBHHABHAHB

*002155_00001960 ABAAHHBHHHHHABHAHHHHBHAAHHAHAHBHHHHAHHBHAHHHAHHBBHBHHHA-HBBHHAHAHBHHAHHHAHBBAHBBHHHBAHHAAHBAAHAAAHAABAHHHHHHHHHBBAABBBHBBHHBHHAABHHHHHHBHHHAHABH

*000009_01161840 A-HH-HHHAHH-AH-HHHH-HHHHABHABBBHAAHABHHBHHBHAHAH-HHHBHABHHHHHABAABHH--BHHAH-AAAHAAHB-HAA-HHAAHHHHHBHBBHHHAABHBAHBHAAHHHHBBHABAAH-HHHBHHABABHAHHH

*000392_00223846 HHAHHHBAHHHHBBBHBHBHHHBHABBHBBHHBHBHHAHAAABHHBHHHHHHBHABABHHHHHAAHBA-AAHHHABHHBBH-BHHAHB-HBAHBHHABBHHBAHBH-AHHAHHHHBBHHHHBAHHHHAHHBBBAAHAHHAHHHH

*000002_00294494 HHHAAHHHHHB-ABHHHHAHAHAHBAHHAAHHBBHAAABHHBHHHAHAAHHAHBHHHHHAHAHAAHAABAHAHAHHAAHHHAHBHABHHHHHHABHHHBHAHAHAH-HHBHABHHAHBHBHHHABHHHHHAABHBHAHBHBAAB

*000854_00181988 HHBHBHBBHAHH-AHAHHBHHAHHHHHHHHHHHHHBHAHBAABAHHBHAHHBHHBBHHHHHHHHHABHHBBBHHHHABHABAAH-BABHBHAHBHABBBBHHBHHABABHHHBHAHHHHBHABHHBBBHBHHBBHHHBHBABHB

*000090_00185327 HBHBHHABHHBBHHBHHH-HHHBAHHHHHHHHHHHA-ABHHBHHAHHHHHABHHHBBBAHHHAHBAABHBBABAHHHHHBBAHBHAAAHBHBHHHAHHAABHAHHHBAHBBAHAHBAAHHHABHAHAHB-HHHABHHHAAAHHB

*000428_00320334 BHBHBAHHBHHBHHHHHHAHAHHAABHHAHAHBAHAHHHHBHHBAAAHAHHAAAHAHHHHHABABHHBHBHHAHABABHHHHABAHHHHAAHHHHBBABABHHBHHAHHBBAHHHBHBAHBHHAHHBABBHHHAHAHHAHHHBH

*000541_00121519 BHHAAHHAHBAAAABHBBHBHHHBHBAHHHAAABHHABBAHHHHBHBHHHHHBABHHAABAHHHBHAAHHHHBAAHABHHAAHBHHHBBHAHBABBHAAHBHHHHAHAHHHHHHHAHHHHHHHHHHABHHHHBABHHBAHHAHB

*000580_00319269 BHBHBAHHBHHHHHHHHHAHAHHAAHHAABAHBHHAHHHHBHHBAAAHAHHAAAHAHHHHHABHBHHBHBHHAHABABHHHHABAHHHHAAHHAHBBABABHHBHHAHHBBAHHHBHBAHBHHAHHBABBHHHAAAHHAHHHBH

*000736_00045658 HHHBBHABBHHHHHAHHBAHBBAHBHHAHAAHAAAHHHAHHBHAHHBHAHAHHHHHBHHHHAAHHHAAHHBHBHBHHBHHBHHBHAHAHBHHHBBBHBHHBHHHBBABHAHBBHABAHHBBBBBHBHAHHBHHHHAHHBAHBHH

*000877_00095445 BHHHHAHABBHHAABHBBHBHHBHHHAHHAAAHHABHBHHHHHHHHBHHAHBBAHHHAHBAHHABHHABAHHBAHHABBHAAAHBHHBBHAHBAHBBAAABHBBHAHAHHHHHHAAHAAHHHHHHHABHHHHBAHHHHAHHAHH

*000949_00155785 HHAABAHAABHBAHAHHHHBBHHHHHABAAHHHHBHBHHBAAHHHHHHHHHHBHBHHAAHHABHHBBABHBHHBAAAHBAHHABABBHHHHHAHHBHAHHAHHAHAHHHBBHBHABHHAHHAHBHHBHAHHHBHBHHBAHBHHB

*001125_00237516 BHAHHAHHHHBHHHAHAAHBBHHHHHHBHHHHBHBBAHHBHAHAHAHBHHBBHHHHHBHHHAAHAHAHHHBABHHBAAHBHBHBHAHBABBBBAHHHAHABHHAHHAHHABHHHHHHAABHBHBAABABAHAHAABHHHHHHBH

*001144_00219448 HHBAHAAHHHAHAAHHABHHHHAHHBAHAAHAHABBHHBAHHHHHHHBHHBABHAHBABHAHBHHHBHHAHHHBBHBBHHHABBHAHAAHAHHHBHHHBHHHHHHHBHBAHBBHAHAHAABHBHAHHBHBHHHHBABAHHHHAB

*001159_00266738 HHABHAHAAHHBAHHAHBHBBHHHHBAHAAHHAHBABHHBAAHHHBHHHHHHHHBHHAHHHABHABBABHBHHBHAAHHAHHAHABHHHHHHAAHBBAHBHBHHAAHHHBBHBHABHHAHHAHBHHBHAHABBHHHHBAHHBHH

*001214_00219152 HHAAHAHAABHBAHAHHHHBBHHHHHABAAHHAHBHBHHBAAHHHBHHHHHHBHBHHAAHHABHABBABHBHHBHAAHBAHHAHABBHHHHHAHHBHAHHABHHHAHHHBHHBHABHHAHHAHBHHBHAHHHBHBBHBAHBHHB

*006518_00007406 BHHHHAHABBHHAABHBBHBHHBHHHAHHAAAAHABHBHHHHHHHHBHHAHBBAHBHAHBAHHABHHAHAHHBAHHABBHAAAHHHHBBHAHBAHBBAAABHBBHAHAHHHHHHAAHAAHHHHHHHABHHHHBAHHHHAHHAHH

*006718_00039351 BHHHHAHABBHHAABHBBHBHHBHHHAHHAAAHHABHBHHHHHHHHBHHAHBBAHHHAHBAHHABHHABAHHBAHHABBHAAAHBHHBBHAHBAHBBAAABHBBHAHAHHHHHHAAHAAHHHHHHHABHHHHBAHHHHAHHAHH

*000236_00444030 HBHHHBHAHHAHHBHAHBBHABHBBHHHBHHBAAHHHHHHHHHABAABAABBBBHAAHHHBBBHHHHBAAHHBHABHHHHHABBHAHAHBHAAHHHAABHAAHHHA-HBHHAHHHHHHBBHHAAHHBBHHHHBHHBHBHHHABH

*000280_00056800 HBHHHHABAHBHHHHHHHHAHHBAHHHBHAHHHHHAHAHHHBHHAHAHHHABBAHBBHAAHHHHBHABHBBABAHBHHHBHAHB-AAAHBHBHHAAHHHABHAHHHBAHBBAAABHHBHHBHBHAHAABHHHHABHHHAAAHHH

*002559_00006106 BBHHHBHHHAAHHHHAHHBAABHBBHBHHBAHAAHHHAHAHHHHBAAHAAHBHHHHAHBHHABAHAHBAAHHBAAHHHHHHABBAAHAHBHAAHHHAHHHHAHHHH-AHAHAHHHBHBBBBHAAHHHBHBHBBHHBHBAHHHBH

*003690_00020419 HAHABAHHBHBHHBHBHHHHAHBAHBHBHHHHHAHHHHHHHHBBAAHHHBBAAHHHHHHAHABABHHBHHBHAAHB-BHAHHABAAHAHHAHAHAHAABHHHBBHHAAHBHHBHHHHBAHBAHAHBHAHBHHABHAHHAHHHBB

*000130_00232517 ABHBHHHHBHA-HBBHBHHHHHHHHBBHBBHHHHHBBHABHBHBHABBHBHHBBHBBHHHHHAHBAHAHBHHBBAH-ABHBHBBHHHAAHHHBABHHHBAHBHHHABHBHBHHHHAHAHHHABHHHBBHAHHHBBHBAHBHABH

*000150_00199509 ABHBHHBHHAABHHHBHABAHAHHAHAHBABBAABHAHAHHHHHHHABHBBAHHHBAHBHBHHAAHHHAHHAAABHHHHHBHAHHAHHAHHAHHBBBHBHHHHAHBBHHHAHHAHHBHHAHHAHHBHHHBHAHAAHHBAHHAAB

*000274_00241771 ABHBHHBHHHABHBBHBHHHHHHBHBHHBHHHHAHHBHABHBHHBABHABHAABHBBHBHHBHHHHHAHBHABBAAHAHHBHHBHHHAHHBHBHHBHHBAHBHAHABBHHHAHHAABHHHHABHHHBBAHHHHBBHBAHHHABA

*000274_00435537 ABHBHHBHHHABHBBHBHHHHHHBHBHHBHHHBAHHBHABHBHHBABHABHHABHBBHBHHBHHHHHAHBHABBAHHAHHBHHBHHHAHHBHBHHBHHBAHBHAHABBBHBAHHAABHHHHHBHHHBBHAHHHBBHBAHHHABA

*000872_00132485 ABHHBHAHAAHAAHHHHHHHHHHHAHHABBBHHAHABHHBHHHHHHAHHHHHBAABAHHHHABAABHHABBHHHHBAAHHAAHBAHAHAHHAAHHBHHBHBBHHHHABHBHABHAHAHHHBBAHBAAHBHHHBHHABABHAHHH

*000002_00195613 HHHAAHHHHHBHAB-HHHAHAHAHBHHAAAHHBBHAAABHHBHAHAHAAHHAHBHHHHHAHAHAAHAABABAHAHH-AHHHHHBAABHHHHHHABHHABHAH-HAHHHHBHABHHAHBHHHHHABHHHHHAABHBHAHBHBAAB

*002452_00082918 HBHBHBHAAHH-ABHBHBHBHHHHAHABHAAHHBAAHAHAAHHHHHHHHHHBHBBBHBBBHHAHHHAABBBHBABH-AHHBHHBHAHA-AHHHHBAHHABHBHHAHBHHHBABABHHABHHHHHHHAHABAHHHHHHHABHAH-

*000318_00011739 HHAAHHHBBBAHHBABHHABAHBHHHHHHHHHAHAHHBHBBHBHHAHAHHABHHBHHHAAHABAHABHHHBBHAAHAHHBAAHHHHAHBHAHHHHAHAHHHHAAHAHHAA-AHABHHHHHBBHAHBAHHHHHHBAHHABBABBH

*001322_00352415 HHHHBHBBBAHHHBABHHBAAHHHHHHHBHBAAHHHHHHBHHBBHAHAABHBAHBBAHAHAHBBHAAABHAHHAHAHHHHHHHH-BHHBHHHHHHHHAHBHHAAHABHAABHHABABAHBAHAAHHAAAHHHHHAAHHBHHBHH

*001500_00083020 HHHBHAHHHHBH-HAHAAHBBHHHHHHBHHHHHHBBAHHBHAHAHAHBBHBBHHHHHBHHHAAHAHAHHBBABHABHHHBHBHBHAHBABBBBAHHHAHABHHAHHAHHABHHHHHHAABABHBAABHBAHHHABBHBHHHHBH

*002144_00068188 HHAAHHHBBBAHHBABHHABAHBHBHHHHHHHAHAHHBHBBHBHHHHAHHABHHBHHHAAHABAHABHHHBBHAAH-HHBAHHHHHAHBHAHHHHAHAHBHHHAHAHHAAHAHABHHHHBBBHAHBABHHHHHBABHABHABBH

*000032_00811851 HBHBHBHBABH-HBBBHBHBHHHHAHAHHAHHHHHHHAHAAHAHHHHHHHABHBBBHBBBHAAHHAAHHBBHBABHAAHHBHHBHHHABHHHHHBAHBABHBHHAHBHHHBAHABBHABHHHHHHHAHHBABHHHBH-ABHAHB

*000130_00376651 ABHBHHHHBHABHBBHBHHHHHHHHBBHBBHHHHHBBHABHBHBHABBHBHHHBHBBHHHHHAHBHHAABHHBBAHAABHBHBBHHHAAHHHBABHHHBAHBHHHABHBHBHHHHAHHHHAABH-HBB--HHHBBBBAHHHABH

*007302_00008208 ABBABABABHHHHAHAAHHBAHBHBHAHHABHHHBHAH-HHAHHHHHHAAHHHAAHHHHBHHHHHHHHAHA-BABBBAHHHBHHHBBHABHHAAHHHHBBAHHHHBBHHHHABBBBBHAAAHHABHHBBHHAHHB-AHHBBHHH

*000100_00264152 AHHBHHBHAHBHAHAHBHBHHHHHABBBBHHHAHHHHHABHHAAHBHHABAAHHAHBBABHHHHBHBHBHAABAAAAHAHHHHBHAHHHHAAAHHAHAHHAAHBHBHHHABAHHHHBAHAHHBHHBAHABHHHHBABHHHHAHH

*000114_00095893 HBHBHBHBAAHHHBBBHBHBHHHHAHABHAHHHHHAHAHAAHAHHHHHHHHBHBBBHBBBHAAHHAAHHBBHBABHHAHHBHHBHAHABHHHHHBAHBABHBHHAHBHHHBAHABBHABHHHHHHHAHHBABHHHBHHABHAHB

*000154_00363800 AHHBAHBBAHHAHBAABHBHBHBHABBBBHABAAHHHHABHBHHHHHHAAAABHHHHBHHBAAABHBBHHHAHAAHAHHHHHHBHAHHHHHAAHHHBHHBHHHHHHHHBBBHBHHBHAHAHHHBBBAAHBHHHBHAHHHBHAHH

*000203_00040671 HAHABAHHBHBHHBHBHHHHAHBAHBHBHHHHHAHHHHHHHHBBAAHHHBBAAHHHHHHAHABABHHBHHBHAAHBABHAHHABAAHAHHAHAHAHAABHHHBBHHAAHBHHBHHHHBAHHAHAHBHABBHHABHAHHAHHHBB

*000280_00355390 HBHHHHABAHBHHHHHHHHAHHHAHHHBHAHHHHHAHAHHHBHHAHAHHHABBAHBBHAAHHHHBHABHBBABAHBHHHBHAHBBAAAHBHBHHAAHHHABHAHHHBAHBHAAABHABHHBHBHAHAABHHHHABBHHAAAHHH

*000346_00289729 HAHHHHHHBBHBABHHBBHBHHBABHBHAAHBHBHBBHAAHBHHABHAHAHHHHHHHHHHBBBBHABHAAHBHAHHHAHHAABHHHHBBAABHHBHBBBHAABABHHHHBHBHBAAHHHHHHHHHHHHHHBHHHBBHBBBHBHH

*000365_00122470 AHHBBABBAHHHHAAAHHBHHAHAHHAHHHHHHHHBHAHBAABAAHBHAHHHBHBBHHHHHBHBHABHHHHBHHAHAHHHBAAHBHABAHHAABHAHHBHHHBHHABAHBHHHHAABHAHBAHHHBHHHHHBBHAAHBHHABHB

*000368_00311155 AHHBAHBHAAAAHAAHHHBBHHHBAHBBBAHAHHAAHHHHHHBBBHHHAHAHBHHBAHAAAAHHHHHBBBBHHHAHHHHAHHBHHHHAHHHHHAAAABAHABABBHAHBHHHHHHHHAHBAHAHHAHAHHBHBBAHBHHHHHHA

*000474_00159938 AHHHHHABHHHABHHAHBBAABHBBBBHHBABHABAHAHABHHHBHHHAAHHBHHHHHHHBAHHHAHHAAHHHAHBHHHHHHBBHAHHHBHAAHAHAHHHHHHHAHAHBBHHBHABAHHHBHHAHBABBBABAHBBHBAHHAHH

*000693_00252144 AHHHHHHAAHBHHHHHBBBBHHHABHBHAHHHAHAHBAAHBABHAHHBAHHHBBBHHHHHHBAHHABHHAHHHAHAAHAAABBHHBHABBHHHBHBAHBHHBHHAAHBBHHHAHABBHHABAHHBHBHAHAAHHHHHAHHHHHA

*000736_00342398 AHHBBHABBHHHBHAHHBAHBBAHBHHAHAAHAAAHHHAHHBHAHHBHAHAHHHHHBHHHHAAHHHAAHHBHBHBHHBHHBHHBHAHAHBHHHBBBHBHHBHHHBBABHAHBBHABHHHBBBBBHBHAHHBHHHHAHHBAHBHH

*000800_00014550 AHHHHHHAAHBHHHHHBBBBHHHABHBAAHHHAHAHBAAHBHBHAHHHAHHHBBBHHHHHHBAHHHBHHAHHHAHAABAAABBHHBHABHHHHBHBHHBHHBHHAABBBHHBAHABHHHABAHHBHBBAHAAHAHHHAHHHHHA

*000837_00085588 HBHHAAHHBBHHABHHABABBHHHHHBBAHBHHBHHAAHHHBAHHABHHHBBHBHHHBHAHHHAHAHAHABHBHHHBAHAHHHHBAHHHHHABAHHAHHBHHAHHBHHHBBABHAAHBABBBHBHHBHHBHHHABBHHHHHAAB

*000898_00107482 HBAABAHAHBHBAHAHHHABBHHHHHABAAAHHBBHBHABAAHHHHHBAHHHBHBAHHAHHABBHBHABHBHHHAHAHBAHHABHBBHHHHHABHBHAHHHHHAHAHHHBHHBHHBHHAHHHHBHHBBHHHHBHAHHBAABHHB

*000923_00124960 AHAAHHBHHHHHABHAHHHHBHAAAHAAAHBHHHHAHBBHAHHHAHHBBHBHHAABHBBAHAHAHBHHAHHHHHBBAHBBHHHBAHHAAHHAAHAAAHHABAHHHHHHHHHBBAABBHHBBHHBHHAABHHHHHHBHHHHHABB

*001100_00083119 HBHBHHHBHHBBBHBHHHHHHHBAAHHHHHHHHHBHHABHHBHHAHHHHHABHBHBBBAHHHABBAABHHBABABBHHHBBAHBHAAAHBHBHBHAHBAABBHHHHHAHHBAHAHHHAHHHHHHAHAABHHHHABBHHAHAAHB

*001667_00144534 HAHABAHHBHBHHBHBHHHHAHBAHBHBHHHHHAHHHHHHHHBBAAHHHBBAAHHHHHHAHABABHHBAHBHAAHBABHAHHABAAHAHHAHAHAHAABHHHBBHHAAHBHHBHHHHBAHBAHAHBHAHBHHABHAHHHHHHBB

*001808_00156940 HBHBHAHBHBBHHBAHHAHHBHAHBAHBBBHHHHHBHHHBHAHBHHHBBBBHHHBHBHHAHAABHHHHHBBHHHAHHAHHHBHBHABHABBBBAHBHHHABBHHHHAAHABHHHHHHAABHBBBAABHHAHHHAHHHAHHBHBH

*002801_00109499 BBAHHHBHHHBAHAHBHBAAABHBAABHHHBHAHABHHAAHHHHBBBAAABHBAHAHAHHHBHBHHHHHHHAHHHHAHBABHHHHAHHHBAHBBAAHHAHHHAHAHAHABHHHAHHHHHHHAHAAHBBHHABHAAHHHAHHHBB

*012064_00001310 HAHABAHHBHBHHBHBHHHHAHBAHBHBHHHHHAHHHHHHHHBBAAHHHBBAAHHHHHHAHABABHHBHHBHAAHBABHHHHABAAHAHHAHAHAHAABHHHBBHHAAHBHHBHHHHBAHBAHAHBHABBHHABHAHHAHHHBB

*001096_00063185 ABHAHHAHBHHHHAHAAHHHAAAHBHAHAABHHHBBHBBAHAHBHHHHAHHHHHBHHHHAHHHABBBHHHHHAHBHBBHHHABBHHBHAHAHBABHHHBBABHHHHBAHAHHBHBBAH-AHBHAAAAHHBHHHBHHHHBBBHHB

*001103_00144766 HHAHHHBAHAHHHBBHBHBHHHBHABBHBAHHBHBHAHHAAH-HHBHHHHHBBHABABHAHHHAAHBA-AABHHAHHHBBHH-HHAHBHHHAHBHHABBHHBAHBHHAAHAHHHHBBHBHHBAH-HHAH-BBBAAHA-HAHHHH

*000037_00034358 HBHHBHABAHBHHHHHHHHAHHBAHBHHHHHHHHBAHAHHHBHHAHHHHHABHHHBBHA-HHHHBHABHBBABAHB-HHBH-HBHA---BHBHBAAHHAABHAHHHBAHBBAHAHBABHHBHBBAHAABHHHBABHHHAA-HHB

*001197_00141346 A-ABBHHHAHBHAHBHHHHHBHHHABAHHHBHHAHAHHBBAABHHHAHBHHHBBABHBHHHAHHABHHABBHHHBBAHBHAAHB-HHAAHHAAHH-HHHHBH-AHB-BHBABBA-BHHHHBHHBB-HHBAHHBHH-HABHHHBH

*000175_00066951 HBHHABAHBHAHAH-BHBAABHHAHAAHAHHBHHAHBA-HBHAHHHHAAHHHHHHAHAHHBHHAHAHB-HAHBAAHBBHAHA-BHBBAHHHAHHHAAHHHHHAHHH-BAHBHAHHAAHBHABHHAHHHHAHAHBAHHBHAHAHB

*001804_00001557 HHBAHAAHHHABAAHHABAHHHAHHBAHAAHAHHBBBHBABHHHHHHBHHBABBAHBABHAHBHHHBHHAHHHBBH-BHHHABBHAHAAHHHHHHHHHBHHHHHHH-HBAHBBHAHHHAABHBHAHHBHBHHAHBABAHHHHAB

*000040_00759070 AHHAHHAHHHAHHBHHHBBHABBHHBAHBAHBAAAHAHHHBHAAHHHBHAHHBBHHABAHBBBHHHHBAH-HHBHBBHAHBABHHHHAAHHAHHAHAABHHAHHHA-HBHHAHBBHAHBBHHAAHHHHBHHHBAHBH-BHHAHH

*004296_00032076 HBHHHBHAHHA-HBHAHBBHABHBBHHHBHHHAAHHHHHHHHBABAABAAHBBBH-AHBBHHBHHHHBAAHHBHAHHHHHHABBHAHAHBHAAHHHAABHAAHHHAAHBHHAHHHHBHBBHHAABHBBHHHHBH-BHBHAHHBH

*002732_00054547 HHBBHHBHBBHHBHHHBBHBABHBHHHBHHBHAHBHH-BBAHHHHBBABHBH-HHBHHBBHHHHHBBBBBHABHHHABBBHHBH-HHHBHHBHAAHBHAHHHBHAHAHABBHBHHHBBHHBHHABHBBHBAHHAAHBHH-HHHB

*000104_00484133 HHHHHHHAAHBAHHHHBBBBHHHABHBHAHHHAHAHBAAHBABHAHHBAHHHBBBHHHHHHBAHHHBHHAAHHAHAAHAAHBBAHBHABBHHHBHBAHBHHBHHAAHBBHHHAHABHHHABAHHBHBHAHAAHHHHAAHHHHHA

*000228_00511223 BHHHHBHAHHBABAHBBHHAAHHHAAAHHAAHHBHAAHHHHBAHHHHBBAHAHBAHBHHBHHBHABAHBHHAHHBHHHABHAAHHBHHHBBHAAHHAAHHHBHAHBBBHBHHHHHAHHBBABBAHHAHHBHBHAHAHHHAHHHB

*000336_00450739 HHBABAHHBHBHHHHHHHAHAHHAABHBAHAHBAHAHHHHHHBBAAHHAHHAAHHAHHHAHABABHHBBHHHAHABABHHHHABAAHHHHAHHBHBBABAHHHBHHAHHBBAHHHBHBAHBHHAHBHABBHHHHHAHHAHHHBB

*000347_00006061 BHHHHAHABBHHAABHBBHBHHBHHHAHHAAAAHABHBHHHHHHHHBHHAHBBAHBHAHBAHHABHHAHAHHBAHHABBHAAAHHHHBBHAHBAHBBAAABHBBHAHAHHHHHHAAHAAHHAHHHHABHHHHBAHHHHAHHAHH

*000354_00230445 HHHHHAHBBHHHHBABHHBHAHBHHHHHHHBHAHHHHBHBBHBHHHHAAHABHHBHHHAAHABBHAHHAHBBHAAHAHHBHHHHHBAHBHAHHHHAHABBHHAAAAHHAABHHABAHAABBBHAHHAAHHAHHBAHHHBBABHH

*000362_00327774 BHBHBAHHBBHHHAHHHHAHAHHAAHHAABABBHHHHHHHBBHBAAAHAHHAAAHAHHHHBHBHBHHBABHHAHABABHHHHABAHHHHAAHHHHBBABABHHBHHAHHBBAHHHBHBAHBHHAHHBABHHHHAAHHHAHHHBH

*000382_00119908 HHAHAHBHAHHAHAAHHHBBHHABAHBBBAHAHHAAHBHHHHHBBBHHAHAHHHHBAAAAAHHHHHABBHBAHHAHHAHAHHBHHHHHBHHHHAAAABHHAHAHHAHHHHBAAHHHHAHBABAHHAAAHHHHBBHHBHAAHHHA

*001148_00101249 HHAHAHBHAHHAHAAHHHBBHHABAHBBBAHAHHAAHBHHHHHBBBHHAHAHHHHBAAAAAHHHHHABBHBAHHAHHAHHHBBHHHHHBHHHHAAAABHHAHAHHAHHHHBAAHHHBAHBHBAHHAHAHHHHBBHHBBAAHHHA

*001214_00203886 HHAAHAHAABHBAHAHHHHBBHHHHBABAAHHAHBHBHHBAAHHHBHHHHHHBHBHHAAHHABHABBABHBHHBHAAHBAHHAHABBHHHHHAHHBHAHHABHHHAHHHBBHBHABHHAHHAHBHHBHAHHHBHBHHBAHBHHB

*001224_00204352 HHHBHAHHABBHHHAHAAHBBHAHBAHBHBHHHHHBAHHBHAHAHHHBBHBBHHBHHBHHHAAHHHHHHBBABHABHHHBHBHBHAHHABBBBAHHHAHABBHAHHAHHABHHHHHHAABHBHBAABHBAHHHAHBHHHAHHBH

*001357_00207663 HHHHHBABHAHHHHHAHBBAABHBBHBHHBABHABHAAHAHHHHBHAHAAHHHHHHAHBHBABHHAHHAAHHHAHHHHHHHABBHAHHHBHAAHAHABHHHAHHHHAHBAHHBHABAHBHBHAAHBABHBHBAHHBHBAHHHHA

*001941_00067952 HHAAHHHBBBAHHBABHHAHAHBHHHHHHHHHAHAHHBHBBHBHHAHAHHABHHBHHHAAHABAHABHHHHBHAAHAHABAAHHHHAHBHAHHHHAHAHBHHAAHAHHAAHAHABHHHHBBBHAHBABHHHHHBAHHHBBABBH

*002030_00080608 HHHHAAAHHAHHHHHHHHHHAHHHAHHABBBHAHBAHBBHBBBHHAHHBAABAHBBHHBBHAHHHBHHAHBHAABHHHHAAABHBHHAAHAHBBHBHAHHABAHAHHHHHHABHHAAHAHHBHAHAHHHBABHHHBBAHBHHHH

*002228_00045777 HHHHHAHHHAHHAAHHABAHHHAHHBAHAAHAHHBBBHHAHHHHHHHBHHBABBAABHBHAHBBHHBHHAHBHBABBBHHHABBHAHAAHHHHHHHAHBHHHHHHHBHBAABBHAHHHAAHABHAHHBBBHHAHBABAHHHHAH

*000365_00203131 AHHBBABBAHHHHAAAHHBHHAHAHHAHHHHHHHHBHAHBAABAAHBHAHHHBHBBHHHHHBHBHABHBHHBHHAH-HHHBAAHBHABAHHAABHAHHBHHHBHHABAHBHHHHAAHHAHBAHHHBHHHHHBBHAAHBHBABHB

*000693_00289597 AHHHHHHAAHBHHHHHBBBBHHHABHBHAHHHAHAHBAAHBABHAHHBAHHHBBBHHHHHHBAHHABHHAHHHAHAAHAAABBHABHABBHHHBHBAHBHHBHHAAABBHHHHHABHHHABAHHBHBHA-AAHHHHHAHHHHHA

*001534_00015401 BBHABBHHAAHHAHAHHHHHHHHHHAHBAHHHHHHAHBHHHBHBHHAAHHAHHAABBAAAHBHHHBHHHBBHBHBB-HBBHABBBHHHHBHBBBAAHHBAHHAHHHBABBHHBAHBHBBHBBBHAHHABAHHHAHBHAHHHHHH

*003170_00046751 HAHBAHBAAHHAHBHHHHHBBHBHB-HBHHHBBBBHAAHBAAAHHAAHHAHHBBHBBBHHBHHHAABHHABHAAHHBAHBHBHAHAHHBAAAHHHHHHHHBHHBHHHHHHAHAHHBBBHAHHBBAHHHHHHHBAHHAHBBHAHH

*000095_00131692 ABHBHHHABHABHBHHBHHAHHHAAHABBBHHHBHHAAHHBHABABHBBBBAHHHAAHHBHBBAAHHH-AHAAHHHBAHHBHAHAAHBHHBHH-HBHBHAHBHAHBHHHHAHHHHBHHHHHBAHAHHHHHHBBHBBAHHAHAAH

*000052_00067969 ABHBHHHHHHHBHBBHHAHHHHHHHBBHBBHHHAHBBHABHBHBHABBHBHHHBHBBHHHHHAHBHHHBBHHBBAHAABHBHBBHHHHAHBHBABBHHBABBHHHABHBHBHHHHAHAHHHABHHHBBHAHHHBBABAHBHABH

*000122_00068532 ABAABHHHAHBHABBHHAHHBAAHHHAHHHBBHAHAHHBBAHHHHAHHBHHHBHABHBHHBAHHABHHABHAHHHBHHBBAHHBHHHAAHHAAHHAHHAHBABAABHBHBHBBAABHHHHBHHBBHAHBHAHBHHHHHHHHHBH

*000274_00451576 ABHBHHBHHHABHBBHBHHHHHHBHBHHBHHHBAHHBHABHBHHBABHABHHABHBBHBHHBHHHHHAHBHABBAHHAHHBHHBHHHAHHBHBHHBHHBAHBHAHABBBHBAHHAABHHHHABHHHBBHAHHHBBHBAHHHABA

*000424_00153700 ABHBAHHHHHHHHHAHHBAHHBHHBHHAHAAHAAAHHHAAABHAHHBHAHAHHHHHBHHHHAABHHHAHABHBHBHHBHHBHHHHAAHHBHBHABBHBHHBHHHBBABHAABBHABAHHBHBBHHBHAHHBHHHBAHABAABHH

*001385_00056442 ABHBHHBHBHABHHHBBHHAHAHAAHAHBBHHABHHAAHHBAHBAHHBHBBAHHHAAHHBHBBAAHHHAAHAAHBHBHHHBHAHHAHHHHBHHAHBHBBAHBHAHBHHHHHHHHHHBHHHHHAHAHAHHHHABHBHABAAHAAB

*001853_00136235 ABHBHHHHBHABHBBHHHHHHHHHHBBHBBHHBAHBBHABHBHBHABBABHHABHBBHBHHHHHBHHAHBHABBAHHABHBHBBHHHAHHHHBAHHHHBAHBHAHABHBHBHHHAAHHHHHABHHHBBHAHHHBBHBAHHHABA

*000788_00273976 HHABHAHA-HH--HAAHBHBBHHHH-AHAAHHHHBABHHBAAHHHBHHHHHHHHBHHAHHHABHABBA-HBHHBHAAHHAAHAHABHHHHHHHAHBBAHBHBHHAAHHBBBHBHABBHAHHAHBHHBHAAABBHHHHBABHBHH

*000307_00075855 BBHABBAHAHHHAHAHHHHAHHHHHAHBAHHHHHHAHBHHHBABHHAAHHAHHAABBAAAHBHHHBHBHBBHBHBB-HBBHABBBHHHHBHBBHAAHHBAHHAHHH-ABBHHBAHBHHBHBBBHAHHABAHHHAH-H-AHHHHH

*000365_00342822 AHHBBABBAHHHHAAAHHBHHAHAHHAHHHHHHBHBHAHBAABAAHBHAHHHBHBBHHHHHBHBHABHHHHBHHAB-HHBB-AHBHAB-HHAABHAHHBHHHBHHABAHBHHHHA-HHAHBAHHHBHHHHHBBHAAHBHBABHB

*003355_00030243 HBHBAHHHHHHAHHAHHBAHHBHHB-HAHAAHAAAHHH-AABBAHHBHAHAHHHHABHHHHAABHHHA-ABHBHBHABAHBHHHHAAHHBHHHHBBHBHHBHHHBB-BHAABBHABHHHBHBBHHBHAHHBHHHHAHHBAABHB

*000316_00025877 HHABHAHAAHHBAHAAHBHBBHHHHBAHAAHHAHBABHHBAAHHHBHHHHHHBHBHHAHHHABHABBA-HBHHBHAAHHAHHAHABBHHHHHAAHBHAHHHBHHAAHHHBBHBHABHHAHHAHBHHBHAHABBHHHHBAHBBHH

*001214_00060036 HHABHAHAABHBAHAHHHHBBHHHHBABAAHHABBHBHHBAAHHHBHHHHHHBHBHHAAHHABHABBABHBHHBHA-HBAHHAHABBHHHHHAHHBHAHHABHHHAHHHBBHBHABHHAHHAHBHHBHAHHHBHBHHBAHBHHB

*003728_00046364 HBHBHAHBHBBHHBAHHAHHBHAABAHBBBHHHHHBHHHBHAHBAHHBBBBHHHBHHHHAHAABHHHH-BBHHHAHBHHBHBHBHABHABBBBAHBHHHABBHHHH-AHABHHHHHHAABHBHBAABHHAHHHAHHHAHHBHBH

*000055_00430177 HBHBHHHBHHBBBBBHHHHHHHBAAHHHHHHHHHBHHABHHBHHAHHHHHABHBBBHBAHHHABHAABHHBABABBHAHBBAHBBAAAHBHBHAHAHBAABBHHHHHAHHBAHAHHHAHHHHHHBHAHBHHHHABHHAAHAAHB

*000114_00254454 HBHBHHHBAAHHHBBBHBHBHHHHAHABHAHHHBHAHAHAAHAHHHHHHHHBHBBBHBBBHAAHHAAHBBBHBABHHAHHBHHBHAHABHHHHHBAHBABHBHHAHBHHHHAHABBHABHHHHHAHAHHBABHHHBHHABHAHB

*000156_00165380 HAAAABHHHHHHBHBAAAHAHHHHHHHBHHHHHHHHBBBHBBHBHHAABHAAHBHHABBHHAAHAAHBAHABHHHHHAHBABBHHHHHHHHBBABAAAHBHHAAAHAHBHHAHHHHHBBHAAAHAAHHHHAHHHHBBABHHBHH

*000158_00200352 HABABAHHBHBHHBHBHHHHAHBAHBHBHHHHHAHHHHHHHHBBAAHHHBBAAHHHBHHAHABABHHBHHBHAAHBABHHHHABAAHAHHAHAHHHAABHHHBBHHAAHBHHBHHHHBAHHABAHBHABBHHABHAHHAHHHBB

*000325_00306468 AHBAHABAHBBAHAHAAHABAHHHHHAHHHHHHHBHHHHHHHHHHHHBAAHAHHAABHHHHHHHBAHHHHABBHBBBABHHBHHHHHHABAHAHHHHHBBAHAHABBHHHBABBHBHHAAHHHABAHBBBAAHABBHHABHHHA

*000349_00177636 HAHBAHBAAHHAHBHHHHHBBBBABBHBHHHBBBBHAAHBAAAHAAAHHAHHBBHBBHHHBHHHAABHHABHAAHHBAHHHBHAHAHHBAAAHHHHHHHHBHHBHHHHHHAHAHHBHBHAHABBAHHHHHHHBAHHAHBHHAHH

*000351_00104802 AHHBHAHHAHHHAHHHBHBBHHHHABBBBHHHAHHHHBABHAAAHBHAHBAAHHABBBHBHHHHBHBHBAAABAHAHHAHHHHBBBAHHHAAAHHAHAHHHAHBHBHHHABAHHHHHAAHHHBHHBAHABHHHHAABHHHAAHH

*000479_00080342 BBHAHHABAHBHHHHHHHHAHHHAHHHBAAHHHHHAHHHHHBHHAHAHHHABBAHBBHAAHHHHBHHBHBBHBAHBHHHBHAHBBAAAHBHBBHAAHHHABAAHHHBAHBHAAABHABHHBHBHAHAABHHHHABBHHAAAHHH

*000684_00053208 AHHBBHABBHHHHHAHHBAHBBAHBHHAHAAHAAAHHHAHHBHAHHBHAHAHHHHHBHHHHAAHHHAAHBBHBHBHABHHBHHBHAHAHBHHHBBBHBHHBHHHBBABHAHBBHABHHHBBBBBBBHAHHBHHHHAHHBHHBHH

*000693_00112023 AHHHHHHAAHBHHHHHBBBBHHHABHBHAHHHAHAHBAAHBABHAHHBAHHHBBBHHHHHHBAHHABHHAHHHAHAAHAAABBHHBHABBHHHBHBAHBHHBHHAAABBHHHAHABAHHABAHHBHBHAHAAHHHHHAHHHHHA

*000751_00371268 HBHBHAHBABBHHHAHAAHBBHAABAHBHBHHHHHBHHHBHAHHAHHBBBBBHHBHHHHHHAABHHHHHBBABHABHHHBHBHBHAHHABBBBAHBHAHABBHHHHAAHABHHHHHHAAHHBHBAABHBAHHHAHHHHHHHHBH

*000927_00070387 HBHBHAHBHBBHHBAHHAHHBHAABHHBBBHHHHHBHHHBHAHBAHHBBBBHHHBHBHHAHAABHHHHHBBHHHAHHHHBHBHBHABHABBBBAHBHHHABBHHHHAAHABHHHHHHAABHBBBAABHHAHHHAHHHAHHBHBH

*000963_00207357 HBAABAHAABHBAHAHHHABBHHHHHABAAAHHBBHBHHBAAHHHHHBHHHHBHBAHHAHHABBHBBABHBHHHAAAHBAHHABABBHHHHHAHHBHAHHAHHAHAHHHBHHBHHBHHAHHHHBHHBBAHHHBHHHHBAHBHHB

*001017_00038064 BBHHHBHAHHAHHHHAHHBAABHBBHBHHBAHAAHHHAHAHHAABAABAAHBHHHHAHBHHHBAHAHBAAHHBAAHHHHAHABBHAHAHBHAAHHHAHBHAAHHHHAAHAHAHHHHHBBBBHAAHHHBHHHBBHHBHBHHHHBH

*001199_00021037 BBHABBAHAHHHAHAHHHHHHHHHHAHBAHHHHHHAHBHHHBHBHHAAHHAHHAABBAAAHBHHHBHHHBBHBHBBAHBBHABBBHHHHBHBBHAAHHBAHHAHHHBABBHHBAHBABBHHHBHAHHABAHBHAHBHAAHHHHH

*001853_00138784 HBHBHHHHBHABHBBHBHHHHHHHHBBHBBHHBAHBBHABHBHBHABBABHHABHBBHBHHHHHBHHAHBHABBAHHABHBHBBHHHAHHHHBAHHHHBAHBHAHABHBHBHHHAAHHHHHABHHHBBHAHHHBBHBAHHHABA

*001889_00136129 HBHHAAHHBBHHABHHABABBHHHHHBBAHBHHBHHAAHHHBAHHABHHHBBHBHHHBHAHHHAHAHAHABHBHHHHAHAHHHHBAHHHHHABAHHAHHBHHAHHBAHHBBABHAAHBABBBHBHHBHHBHHHABBHHHBHAAB

*001925_00081475 HAHABAHHBHBHHBHBHHHHAHBAHBHBHHHHHAHHHHHHHHBBAAHHHBBAAHHHHHHAHABABHHBHHBHAAHBABHAHHABAAHAHHAHAHAHAABHHHBBHHAAHBHHBHHHHBAHBAHAHBHABBHHABHAHHAHHHBB

*002752_00081130 AHAHAHBHAHHAHAAHHHBBHHABAHBBBAHAHHAAHBHHHHHBBBHHAHAHHHHBAAAAAHHHHHABBHBAHHAHHAHAHHBHHHHHBHHHHAAAABHHAHAHHAHHHHBAAHHHBHHBABAHHAAAHHHHBBHHBHAAHHHA

*003743_00049587 AHAHAHBHAHHAHAAHHHBBHHABAHBBBAHAHHAAHBHHHHHBBBHHAHAHHHHBAAAAAHHHHHABBHBAHHAHHAHHHHBHHHHHBHHHHAAAABHHAHAHHAHHHHBAAHHHBAHBABAHHAAAHHHHBBHHBHAAHHHA

*005622_00054732 BBHABBAHAHHHAHAHHHHHHHHHHAHBAHHHHHHAHBHHHBHBHHAAHHAHHAABBAAAHBHHHBHHHBBHBHBBHHBBHABBBHHHHBHBBHAAHHBAHHAHHHBABBHHBAHBABBHHBBHAHHABAHBHAHBHAAHHHHH

*000013_01078797 ABHAHHHHBHHHAHHAAHHAHHHHHBHBBHHHHBBAHHBAAAHBHBHBHHHHHHHAHAHAHHHBBHHAABHHBABHBHHAHHBHHABHHHAHHABBBABBHHAHBB-AAAHHBHABHHHHHHHHAAABHBBHBHHBHHBBBAHB

*000059_00360264 ABBABABABHHBHAHHAHHBABBHBHAHHHHHHHBHAHHHHAHHHHHHAABBAAAHHHHBHHHBAHHH-HHBBHBBBAHHHHHHHBBHABHHAAHHHBHBHHHAHBBHHHBABBHBBBAAAHHABHHHBHHAHHBHHHHBBBHB

*000079_00596621 HBHHBHHBHHBBBH-B-HBHHBBHBBHBBBBHHHBHBBBHBHHB-B-HHHHBBHH-BBBBBHHHHBHBBHBBBHHHHBBHHBBB--HBH-HHH-HBHHHBHHBHHH-BHBHHBHBBB-BHHHHHHBHBHB-BHBB-HHHBHHBB

*000254_00352101 HBHBHAHHHBHB-BBHHAHHBHAHHHHBBBHHHHHBHHABHHHBHABBBBHHHBHHBHHAHHABBHHH-BBHBBAH-ABHHBBB-AHHAHBBBABBHHHABBBHHABHBABHHHHHHAAHHHBBHHBHH-BHHAHHHAHBHHBH

*000258_00519861 BHHABBAHAHHH-HAAHHHHHHHHHAHBAHHHHHHABBHHHBHBHH-ABHHHHAABBAAHHBAHHHHHHBBHBBBHAHBHHABBBBHHHBHBBHAAHHBAHHAHHBBABBHHBAHBHBBHBBBHAHHAHAHBHAAHHAAHHHHH

*000313_00307732 HHAHHHBAHHHAABBHBHBHHHBHABBHBHHABHBHAHHAAH-HHBHHHHHBHHABHBHAAHHAAHBAAAAHHBAH-HBBHHHHHAHBHHHAHBHHABBHHBAHBHHAAAAHHHABBHHHHBHHHHHAHHBBBAAHABHAHHBH

*003070_00070736 HHAAHHHBBBAHHBABHHABAHBHHHHHHHHHAHHHHBHBBHBHHAHAHHABHHBHHHAAHABAHABHHHBBHAAA-HHBAAHHHHAH-HAHHHHAHAHBHHAAHAHHAAHAHABABHHBBBHAHBABHHHHHBAHHHBHABBH

*000039_00323093 AHBHHBHHHHHBBHHHBHHBHHAHA-HHABHHHHABBAAAHH-HHHBHAAAHHHABAAAHHHHHHHAHAHBHBHHH-HHHBHBAHBHBHBHAHBHHHBHHHBHAAABHHBABHBABHHBHHAAHBBBBAAAHBABHBBHHHBHH

*000164_00126512 HHHHHAHHHAHHAHHHAHAHHHABHBHHAAHAHHBBHHHHHHHBBHBHHHBABBAABHBBAHBBHHBHHAABHBABHHHHBHBHHAHAABHAHHAHAAHHHHHHAHHHBAHBBAAHHHAAHABHAHHBBBAHHHBABABHHHAH

*000277_00705399 HHAAHHHBBBAHHBABHHABAHBHHHHHHHHHAHAHHBHBBHBHHAHAHHABHHBHHHAAHABAHABHHHBBHAAHAHHBAAHHHHAHBHAHHHHAHAHBHHAAHABHAAHAHABHHHHBBBHAHBABHHHHHBAHHHBHABBH

*000354_00380764 HHHHHAHBBHHHHBABHHBHAHBHHHHHHHBHAHAHHBHBBHBHHHHAAHABHHBHHHAAHABBHAHHAHBBHAAHAHHBHHHHHBAHBHAHHHHAHABBHHAAAAHHAAHHHABAHHABBBHAHHAAHBAHHBABHHBBABHH

*000610_00251186 HHAAHAHBBBAHHBABHHABAHBHHBHHHHHHAHAHHBHBBHBHHHHAHHABHHBHHHAAHABABABHHHBBHAAHAHHBAAHHHHAHBHAHHHHAHAHBHHAAHAHHAAHHHABHHHAHBBHAHBAHHHHHHBAHHHBBABBH

*000691_00282129 HHHHHAHHHAAHAAHHABAHHHAHHBAHAAHAHHBBBHHAHHHHHHHBHHBABBAABHBHAHBBHHBHHAHBHBABBBHHHABBHAHAAHHHHHHHAHBHHHHHHHBHBAABBHAHHHAAHABHAHHBBBHHAHBABAHHHHAH

*000788_00244388 HHABHAHAAHHBAHAAHBHBBHHHHBAHAAHHAHBABHHBAAHHHBHHHHHHHHBHHAHHHABHABBABHBHHBHAAHHAAHAHABHHHHHHAAHBBAHBHBHHAAHHHBBHBHABHHAHHAHBHHBHAHABBHHHHBAHHBHH

*000809_00248300 HHHHHAHHHAHHAAHHABAHHHAHHBAHAAHAHHBBBHHAHHHHHHHBHHBABBAABHBHAHBBHHBHHAHBHBABBBHHHABBHAHAAHHHHHHHAHBHHHHHHHBHBAABBHAHAHAAHABHAHHBBBHHAHBABAHHHHAH

*000878_00100287 HHABHAHAAHHBAHAAHBHBBHHHHBAHAAHHAHBABHHBAAHHHHHHHHHHBHBHHAHHHABHABBABHBHHBHAAHHAHHAHABBHHHHHAAHBHAHHHBHHAAHHHBBHBHABHHAHHAHBHHBHAHABBHHAHBAHBBHH

*001127_00138509 HHHHAHAHHHABHBHABAHBHHHHHAHBHAHAHABHHABHBHAHHAHHAAABAAHHBBBAAHHHHABBHABAHBAHAHHAHAHHHAABBBAHHAHHBBHHABHHHHHBBAHHHHHHHBHAHHBHHHABHBHAHBHBHHHHAHHA

*001292_00047241 HHAAHHHBBBAHHBABHHABAHBHHHHHHHHHAHAHHBHBBHBHHAHAHHABHHBHHHAAHABAHABHAHBBHAAHAHHBAAHHHHAHBHAHHHHAHAHBHHAAHAHHAAHAHABHHHHBBBHAHBHBHHHHHBAHHHBBABBH

*001929_00167374 HHAABAHAABHBAHAHHHHBBHHHHHABAAAHHBBHBHHBAAHHHHHHHHHHBHBHHHAHHABBHBBABHBHHBAAAHBAHHABABBHHHHHAHHBHAHHAHHAHAHHHBHHBHABBHAHHHHBHHBBAAHHBHHHHBAHBHHB

*002838_00069633 HHHHAHAHHHABHBHABAHBHHHHHHHBHAHAHABHHABHBHAHHAHHAAABAAHHBHBAAHHHHABBAABAHBAHAHHHHAHHHAABBBAHHAHHBBHHABHHHHHBBAAHHHHHHBHAHHBHHBABHBHHHBHBHHAHAHHA

*008163_00001931 BHHAAHBAHBAAAABHBBHBHHHBHBAHHAAAABHHHBBAHHHHBHBHHAHHBABHHAABAHHHBHHAHAHHBAAHABBHAAHHHHHBBHHHBAHBHAAHBHAHHAHAHHHHHHAAHAAHHHHHHHABHHHHBHHHHBAHHAHB

*000003_00796342 HBAHHHBABHH-HBBHBHHHHHBAHBBHBBAHBHBHHABAAAHHABAHHHHHBHABABHHHHHAAHBAHAAHHHAHHHBHHBHHAAHBHHHAHBHHHBBHHBAHBHHAHHAHHHHBBHHHHBAHHHAAHHBBBAAHAAAAHAHH

*000040_00905948 AHHAHHAHHHAHHBHHHBBHABBHHBAHBAHBAAAHAHHHHHHAHHHBHAHHBBHHABAHBBBHHHHBAHAHHBHBBHAHBHBHBHHAABHAH-AHAABHHAHHHAAHBHHABBBHAHHBHHAAHHHHBHHHBHHBHBBBHAHH

*000147_00246459 AHHAHHAHHHAHABAHABBHAHBHHBAHBAHHAAHBAHHHAHHAHHHBHAHHBBHHABAHHBBHHBHBHHAAHBHB-HABBHBHBHHAHHHAHBAHAABHHAHHHAAHBHHABBBHAHHBHHAAHHHHBHHHBHHBHBBHHAHH

*000156_00166124 HAAAABHHHHHHBHBAAAHAHHHHHHHBHHHHHHHHBBBHBBHBHHAABHAAHBHHABBHHAAHAAHBAHABHHHHAAHBABBHHHHHHHHBBABAAAHBHHAAAHAHBHHAHHHHHHBHAAAHAAHHAHAHHHH-BABHHBHH

*000370_00340578 AHAAHHBHHHH-ABHAHHHHBHAAHHAHAHBHHHHAHBBHAHHHAHABBHBHHHABHBBHHAHAHBHHAHHHAHBBAHBBHHHBAHHAAHHAAHAAAHAABAHHHHHHHHHBBAABBBHBBHHBAHAABAHHHHHBHHHHHABH

*000814_00433766 BBHABBAHAHHHAH-HHHHHHHHHHAHBAHHHHHHAHBHHHBHBHHAAHHAHHHABBAAAHBHHHBHHHBBHBHBBAHBBHABBBHHHHBHBBHAAHHBAHHAHHHBABBHHBAHBABBHBBBHAHHABAHBHAHBHAAHHHHH

*001015_00175519 HBHHAAHHBBHHHBHHABHBBHHHHHBBAHBHHBHHAAHHHBAHHABHHHBBHBHHABHAHHAAHAHAAABHBHHHBAHAHHHHBAHHHHHAHAHHAHHBHHAHHBBHHBHABHAAHHABBBABHHBHHBHHHABBH-HBHAAB

*000183_00090689 ABHBHHBHBHABBHHBBHHAHAHAAHAHBHHHABHHAAHHBAHBAHHBHBHAHHHAAHHBHBBAAHHHAAHAAHBH-HHHBHAHBAHHHHBHHAHBHBBAHBHAHB-HHHHHHHHHBHHHHHAAAHAHHHHABHBHABAAHAAB

*000282_00440858 ABHHHHBHHHABHBBHBHHHHHHHHBHHBHHHBAHHHHABHBHBHABBABHHABHBBHBHHBHHHHHA-BHABBAHHAHHBHABBHHAHHBHBHHBHHBAHBHAHA-BBHBAHHAABBHHHABHHHBBHAHHHBBHBAHHHABA

*006710_00005589 ABAAHHBHAHB-ABHHHAHHBHAHH-AHHHBBHHHABHBHAHHHHHHHBHHHHHABHBHHBAHHABHHABHAHHBBHHBBAHHBAHHAAHBAAHHAAHAHBHBAABHBHBHBHAABHHAHBHHBBHAABHAHBHBHHHHHHHBH

*009582_00002422 ABHBHHBHHHABHBBHBHHHHHHBHBHHBHHHBAHHBHABHBHHBABHABHHABHBBHBHHBHHHHHAHB-ABBAHHAHHBHBBHHHAHHBHBHHBHHBAHBHAHA-BBHBAHHAABHHHHABHHHBBHAHHHBBHBAHHHABA

*000274_00268832 ABHBHHBHHHABHBBHBHHHHHHBHBHHBHHHHAHHBHABHBHHBABHABHAABHBBHBHHBHHHHHAHBHABBAAHAHHBHHBHHHAHHBHBHHBHHBAHBHAHABBHHBAHHAABHHHHABHHHBBAAHHHBBHBAHHHABA

*000383_00398917 ABBABABABHHHHAHAAHABABBHBHAHHHHHHHBHAHHHHAHHHHHHAAHBAAAHHHHBHHHHAHHHAHABBHBBBAHHHHHHABBHABHHAAHHHBBBAHHHHBBHHHBABBBBBHAAHHHABHHHBHHAHHBHHAHHBBHH

*000386_00050194 ABHHHAAHHHAHAHHBBBHHHHABHHBHABHHHHHBHHAABHBHBHHBAHAHHBHAHBBBHAHHHHHHHHHHBHHAHHAHHABAABHAHHHBHBHBAHBHABBHHAHHAAHAABAHHHAAHAHHBHHAAHHAHHBAAAHBHABA

*000396_00210320 ABHBHHBHHAABHHHBHABAHAHAAHAHBABBAABHAHAHHAHHAHABHBBAHHHHAHBHBHHAAHHHAHHAAABHHHHHBAAHHAHHAHHAHHBBBHBHHHHAHHBHHHAHHHHHHHHAHHAHABHHHBHAHAAHHBAAHAAB

*001014_00298892 ABAABHHHAHBHABBHHAHHBAAHHHAHHHBBHAHAHHBBAHHHHAHHBHHHBHABHBHHBAHHABHHABHAHHHBAHBBAAHBHHHAAHHAHHHAHHHHBABAABHBHBHBBAABHHHBBHHBBHHHBAAHBHHHHHHHHHBH

*002603_00063993 ABHHHAAHHHAHAHHBBBHHHBABHHBHABHHHHHBHHAABHBHBHHBAHHHHBHAHBBBHAHHHHHHHHHHBHHAHHAHHABAABHAHHHBHHHBAHBHABBHHAHHAAHAABAHHAAAHAHHBHHAAHHAHHBAAAHBHABA

*000556_00158667 AAHHHHHH-BHB-BHHBBHBHHBA-HBHAAABHBHBBHAAHBHHABHAHAHHHHHHHH-HBBBBH-BHAAHBHAHHHAHHAABH-HHBBAABHHBHBBBHAABABHBHHBHBHBA-HHHHHHHHHHHHH--BH-B-H-BB-BHH

*003017_00051059 BHHHBHAAAAHAHH-HHBHHAAHHA-HAHBBHHBBAHHHHAB-HHHHHHHHHBAH-HHHHHABHHBHHABBHAHHB-HHHAABH-HAHAHABHABBHABHABAHHAAAHHHABHHAABBBBBHAHAAHB-BBHHHHBAHHAHHH

*000822_00245151 HBAHHHBABHH-HBHHBHHHAHHAH-BBHBAHBBHHHAAAHHAHABAHHBBHHHHAHBABHBBAAHHH-AAAAHHH-AHHHAAHHAHHHHBHHBHHHBBHHBAABH-HBHAHHHBBBHHHHBHAAHHAHBBBHHH-ABHAHAHA

*000076_00137106 A-HHBHBHHAH-BHHBHHHAABHHH-AHBHBAAHHHHHAHBBABHAHHABHHAABBAAAHAHBHHHAH-BAHHAHAHBHHHHAHABHBHHHAHHBBHHHBHHAAHAHHHHBHHHB-BHHBAHAAHAAAAHHHBABHHBBHBBHH

*001417_00063255 AHHHHHABHBHABHAAHBBAABHBBBBHHBABHABHHHAHBB-HBHHHAAHHBHHHHHBHBAHHHAHHAAHHHAHB-HHHHAHBHAHHHBHAHHAHBHHHHHHHAHAHBBHHBHABAH-HBHHAHBAHBBABA-BBHBAHHAHH

*002684_00118041 BBHHHBHA-HAHHHHAHBBAABHBBHBHBHAHAAHHHAHHHHHABAABAAHBHHHHAHBHHHBAHAHBAA-HBAAH-HHAHABBHAHAHBHAAHHHAHBHAAHHHH-AHAHAHHHHHBBBBHAABHHBBHHHBHHBHBHHHHBH

*004408_00062091 HBABHHBABBHH-B-HBHHAHHHAHBHHBBAHBBHHAAHABHABABABHBBHHHAAHHABHBBAAHHHHAHAAHHHBAHHHH-HAAHHHHBHHBHHHBBAHBHAHB-HBHAHHBHHHHHHHBHHAHHAHHHBBHHBAAHAHAAH

*004563_00006896 AHHHHHHAAHBHHHHHBBBBHHHABHBHAHHHABAHBAAHBABHAHHBAHHHBBBHHHAHHBAHHHBHHAHHHAHAAHAAAB-AHBHABBHAH-HBAHBHHBHHAAHBBHHHAHABHHHABAAH-HBHAHAAHHH-HAHHHHHA

*000110_00081956 HHHAHHHHHHHHAHHAHHHAHHAAABAAAHBHBHHHHBBHAAHHAHHBHHBHHAABHBBAHAHAHHHHAHAHHHBBAHBBHHHBAHHAHHHAABAAABHABHHHHHHHAHHBBBABBHHBBHHHHHAABBHBAHBBH-HHHABB

*000362_00305143 BHBHBAHHBBHHHAHHHHAHAHHAAHHAABABBBHHHHHHBBHBAAAHAHHAAAHAHHHHBHBHBHHBHBHHAHAB-BHHHHABAHHHHAAHHHHBBABABHHBHHAHHBBAHHHBHBAHBHHAHHBABBHHHAAAHHAHHHBH

*000711_00120761 HHABABHBAHAABA-HHHHBBAHHBAHHAHHABAHHBHAHBHHAHHHHBHHAHBHBHHHAAHHHHAHBBBAAHHAHAAHHBHBHHHBAHHAHHAAAHHAHHHABBHABHAHHAHHHHABBHHHBHAHHHBBABHAHBHHHBHHA

*000893_00160156 BHBABAHHBHBHBHHHHHAHAHHAABHBAHAHHAAAHHHHHHBBAAHHABHAAHHAHHHAHABABHHBBHHHAAABABHHHHABAAHHHHAHAHHBHABAHHHBHH-HHBBAAHHHHBAHBHHAHBHABBHHHHHAHHAHHHBB

*001201_00055452 HHHHHAAHHHA-AAHHABAHHHAHHBAHAAHAHHBBBHBAHHHHHHHBHHBABBAHBABHAHBHHHBHHAHBHBBBBBHHHABBHAHAAHHHHHHHHHBHHHHHHABHBAHBBHAHAHAABABHAHHBHBHHAHBABAHHHHAH

*001250_00159596 BHHHHAHABBBHAABHBBHBHABHHHAHHAAAHHABHBHHHHHHHHBHHAHBBAHHHAHBAHHABHHABAAHBAHHABBHAAAHBHHBBHAHBAHBBAAABHBBHA-AHHHHHHHAHHAHHHHHHHABHHHHBAHHHHAHHAHA

*002081_00067862 HHABHAHAAHHBABAAHBHBBHHHHBAHAAHHAHBAHHHHAAHHHBHHHHHHHHBHHAHHHABHABBABHBHHBHAAHHAAHAH-BHHHHHHAAABBAHBHBHHAAHHHBBHBHABHHAHHAHBHHBHABABBHHHHBAHHBHH

*003021_00034898 HHAAHBHBBBAHHBABHHABAHBHHHHHHAHHAHAHHBHBBHBHHAHAHHAHHHBBHHAAHABAHABHHHHBHAAHAAAHBABHHHAHBAHHH-HAHAHBHHAAHAHHAAHAHABHHHHBBBHAHBHBHHHHHBAHHHHBABHH

*000287_00090262 HBHBHBHAAHHHABHBHBHBHHHHAHABBAAHBBHAHAHAAHHHHHHHHHHBHBBBHBBHHHABHHAAHB-HBABH-AHHBHHBHAHABAHHHHBAAHABHBHHAHBHHHBABABHAHBHHHHHHHAHHBABHHHHHAABHAHB

*000603_00156998 HBHHAAHHBBHHHBHHAAHHHHHHH-BBAHBHHBHHHAHHBHABHABHHHHHHBHHABBAHHAHHAHA-ABBBHHHHABAHHHHHHHHHAHABAHHAHHBAHAHHBAHHBBABHAHBBABHBAHHHBHHBHHBABBHABBHAAB

*000832_00025228 AHHH-HHAAHHHABHHHBBBHHHHBHABHAAHBBHAHAHAHHHHHHHAHBHBHHBBHHBHHHABHHAHHBHHBABHBHHBBH-HAAAABAHBHBBHAHHBHBHHHHBBHBHHBABHHAHHBAHHHHHBHBHBHHAABBABAAHB

*001098_00196997 AHHHBAAHAAHAHHHHHHHHHHHHAHHABBBHHAHABHHBHHBHHHAHHHHHBAABHHHHHABAABHH-BBHAHHBBAHHAAHBAHAHAHHAA-HBHABHBBHHHHABHBBABHAHAHAHHBHHBAAHBBHHHHHABABHAHHA

*004518_00013687 BBHABBAHAHHBAHAHHHHHHHHHHAHBAHHHHHHAHBHHHBHBHHHAHHAHHAABBAAAHBHHHBHHHBBHBHBBAHBBHABBBBHHHBHBBHAAHHBAHHAHHH-ABBHHBAHBABBHBBBHAHHABAHHHAHBH-AHHHHH

*000346_00009676 AAHHHHHHBBHBABHHBBHBHHBABHBHAAHBHBHBBHAAHBHHABHAHAHHHHHHHHHHBBBBHABH-ABBHAHHHAHHAABHHHHBBAABHHBHBBBHAABABH-HHBHBHBAAHHHHHHHHHHHHHHBHHBBBH-BBHBHH

*000020_01342362 HHBBHHHABBHHHHHHBBAHHHHHHHHHAABHHHBHHHHBAAHAHBBHBABHBHHBHHBBHHAAHBBBBBAABHAHABBHAABHHHAHHHHHHAAHBHAHHABHAAHAABBHHHAAAHHHBHHHBHHHHBAHHAHHBAHBAHHB

*000091_00138618 HAHABAHHBHBHHBHBHHHHAHBAHBHBHHHHHAHHHHHHHHBBAAHHHBBAAHHHBHHAHABABHHBHHBHAAHBABHAHHABAAHAHHAHAHHHAABHHHBBHHAAHBHHBHHHHBAHBABAHBHABBHHABHAHHAHHHBB

*000091_00145515 HAHABAHHBHBHHBHBHHHHAHBAHBHBHHHHHAHHHHHHHHBBAAHHHBBAAHHHBHHAHABABHHBHHBHAAHBABHAHHABAAHAHHAHAHHHAABHHHBBHHAAHBHHBHHHHBAHBABAHBHABBHHABHAHHAHHHBB

*000091_00819967 HAHABAHHBHBHHBHBHHHHAHBAHBHBHHHHHAHHHHHHHHBBAAHHHBBAAHHHBHHAHABABHHBHHBHAAHBABHAHHABAAHAHHAHAHAHAABHHHBBHHAAHBHHBHHHHBAHBABAHBHAHBHHABHAHHAHHHBB

*000154_00318411 AHHBAHBBAHHAHBAABHBHBHBHABBBBHABAAHHHHABHBBHHHHHAAAABHHHHBHHBAAABHBBHHHAHAAHAHHHHHHBHAHHHHHAAHHHBHHBHHHHHHAHBBBHBHHBHAHAHHHBBBAAHBHHHBHAHHHBHAHH

*000154_00370146 AHHBAHBBAHHAHBAABHBHBHBHABBBBHABAAHHHHABHBBHHHHHAAAABHHHHBHHBAAABHBBAHHAHAAHAHHHHHHBHAHHHHHAAHHHBHHBHHHHHHHHBBBHBHHBHAHAHHHHBBAAHBHHHBHAHBHBHAHH

*000154_00616253 AHHBAHBBAHHAHBAABHBHBHBHABBBBHABAAHHHHABHBBHHHHHAAAAHHHHHBAHBAAABHBBHHAAHAAHAHHHBHHBHAHHHHHAAHHHBHHHHHHHHHHHBHBHBHHBBAHAHHHBBBAAHBHHHBHAHHHBHAHH

*000164_00240075 HAHHHAHHHAHHAHHHAHAHHHABHBHHAAHAHHBBHHHHHHHBBHBHHHBABBAABHBBAHBBHHBHHAABHBABHHHHBHBHHAHAABHAHHAHAAHHHHHHAHHHBAABBAAHHHAAHHBHAHHBBBAHHHBABABHHHAH

*000177_00169443 HAABAHHHHAHBAHAHHBABBAAAHHHHBHAAHBAHHHHBHAAHAAHBHHHBABABHHHHABHHHHAHHHBHBAHHHABAHHBBHBHHHHBBHHABHHBHBABABHHBBAHHAAHAHHHHAHHBHHAHHBBAHAHHAHHHHHHB

*000214_00032429 BAHHAHAHHHAHHHHABAABAHBHHHHAHHHAHHHABABHBHHHHABHAHHHAAAHBABAAHHHHHBBAABAHBAHABHAHAHBHHAHBBAHAHHHBBHAABHHBHHHHAAHHHHBABHHHHBAHBHBHBBHHBHHHHHHAHAA

*000216_00150177 HBHBHHHBHHBBBHBHHHHHHHBAABHHHHHHHHBHHABHHBHHAHHHHHABHBHBBBAHHHABBAABHHBABABBHBABBAHBHAAAHBHBHHHAHBAABBAHHHHAHBBAHAHHHHHHHHBHAHAABHHHHABHHHAHAHHB

*000228_00391051 BAHHHBHAHHBABAHBBHHAAHHHAAAHHAAHHBHAAHHBHBAHHHHBBAHAHBAHBHHBHHBHABAHBHHAHHBHHHABHAAHHHHHHBBHAAHHAAHHHBHAHBBBHBHHHHHAHHBBABBAHBAHHBHBHAHAHHHAHHHB

*000230_00007039 HABABAHHBHBHHHHBHHAHAHHAABHBHHHHHAHHHHHHHHBBAAHHHBBAAHHHBHHAHABABHHBHHHHAAHBABHAHHABAAHAHHAHAHHHAABAHHBBHHAAHBHHBHHHHBAHBHBAHBHABBHHABHAHHAHHHBB

*000233_00244982 BBHHHBHAHHAHHHHAHHBAABHBBHBHHBAHAAHHHAHAHHHABAABAAHBHHHHAHBHHHBAHAHBAAHHBAAHHHHAHABBHAHAHBHAAAHHAHBHAAHHHHAAHAHAHHHHHBBBBHAAHHHBHHHBBHHBHBAHHHBH

*000344_00335375 BBHAHHABAABHHBBHBBHHAABHABHHBABHBBHHABBBBBAHHAHAAAAABABBHHAHHBHHHHHAHHHHBBBHHHAHAHHABAHHHHAHHHAHHHAHHHHHBBAHAAHHHABAAHHHHHHAHHHHHHBHHABAHHHHHBHA

*000349_00248858 HAHBAHBAAHHAHBHHHHHBBBBABBHBHHHBBBBHAAHBAAAHAAAHHAHHBBHBBHHHBHHHAABHHABHAAHHBAHBHBHAHAHHBAAAHHHHHHHHBHHBHHHHHHAHAHHBHHHAHABBAHHAHHHHBAHHAHBHHAHH

*000368_00311069 AHHBAHBHAAAAHAAHHHBBHHHBAHBBBAHAHHAAHHHHHHBBBBHHAHAHBHHBAHAAAAHHHHHBBBBHHHAHHHHAHHBHHHHAHHHHHAAAABAHABABBHAHBHHHHHHHBAHBAHAHHAHAHHBHBBAHBHHHHHHA

*000372_00195952 HBHBHAHBABBHHHAHAAHBBHAABAHBHBHHHHHBHHHBHAHHAHHBBBBBHHBHHHHHHAABHHHHHBBABHHBHHHBHBHBAAHHABBBBAHBHAHABBHHHHAAHABHHHHHHAABHBHBAABHBAHHHAHHHHHHHHBH

*000382_00385661 AHAHABBHAHHAHAAHHHBBHHABHHBBHAHAHHAAHBHHHHHBBBHHAHAHHHHBAAAAAHHHHHABBHBAHHAHHAHAHHBHBHHHBHHHHAAAABHHAHAHHAHHHHHAAHHHHABBABAHBAAAHHHHBBHHBHAAHHHA

*000506_00059929 AHHBAHBBAHHAHBAABHBHHHBHABBBBHABHABHHHABHBBHHHHHAAAABHHHHBHHBAAABHBBHHHAHAHBAHHHHAHBHAHHHHHAAHHHBHHBHHHHHHHHBBBHBHHBHAHAHHHHBBAABBHHHHAAHAHBHAHH

*000778_00077631 BAHBAHHBHHAAAHHHBHABABBHBHHAHHHAHBHABABHBAHAHHBHAHHHAHAHBABAAHHHHHHBAAHAHHBHHBHHBAHBHBAHBBAHAHHHBBHAHBHHBHHHHAHHHBHBHBHHHBBABHHHHHBHBBHHHHHHABAA

*000943_00008597 AHHHABAHHHHHBHAHHBBBBHHHHBHAHHHABHBHBAHHHHBHHHBHAHHABAHHHHBAAHBBHBBBBHAAHHBAHHAHHBHABAHHAAHAHHBHBAHHBHBAHHBHHHHHBBABHBBABHHBHHHAABABHAHHBHBHHHHH

*001030_00089329 HBHHAAHHBBHHABHHABABBHHHHHBBAHBHHBHHAAHHHBAHHABHHHBBHBHHHBHAHHHAHAHAHABHAHHHHAHAHHHHBAHHHHHABAHHAHBBHHAHABHHHBBABHAAHBABBBHBHHBHHBAHHABHHHHBHAAB

*001083_00050118 BAHBAHHBHHAHAHHHBHABABBHBHHAHHBAHBHABABHBAHAHHBHAHHHAHAHBABAAHHHHHHBAAHAHHHHHBHHBAHBABAHBBAHAHHHBBHAHBHHBHHHHAHHHBHBHBHHHBBABHHHHHBHBBHHHHHHABAA

*001083_00122031 BAHBAHHBHHAHAHHHBHABABBHBHHAHHHAHBHABABHBAHAHHBHAHHHAHAHBABAAHHHHHHBAAHAHHHHHBHHBAHBABAHBBAHAAHHBBHAHBHHBHHHHAHHHBHBHBHHHBBABHHHHHBHBBHHHHHHABAA

*001236_00132775 HAHABAHHBHBHHBHBHHHHAHBAHBHBHHHHHAHHHHHHHHBBAAHHHBBAAHHHBHHAHABABHHBHHBHAAHBABHAHHABAAHAHHAHAHAHAABHHHBBHHAAHBHHBHHHHBAHBHBAHBHABBHHABHAHHAHHHBB

*001317_00003443 HAHABAHHBHBHHBHBHHHHAHBAHBHBHHHHHAHHHHHHHHBBAAHHHBBAAHHHHHHAHABABHHBHHBHAAHBABHAHHABAAHAHHAHAHAHAABHHHBBHHAAHBHHBHHHHBAHBAHAHBHABBHHABHAHAAHHHBB

*001404_00057176 BAHBAHHBHHAHAHHHBHABABBHBHHAHHHAHBHABABHBAHAHHBHABHHAHHHBABAAHHHHHHBAAHAHHBHHBAHBAHBABHHBBAHAHHABBHAHBHBBHBHHHHHHBHBHHHHHBBABHHHHHBHBBHHHHHHHBAA

*001580_00012543 HBHHAAHHBBHHABHHABABBHHHHHBBAHBHHBHHAAHHHBAHHABHHHBBHBHHHBHAHHHAHAHAHABHBHHHHAHAHHHHBAHHHHHAHAHHAHHBHHAHABHHHBBABHAAABABBBHBHHBHHBAHHABBHHHBHAAB

*002004_00053324 BBHABBAHAHHHAHAHHHHHHHHHHAHBAHHHHHHAHBHHHBHBHHAAHHAHHAABBAAAHBHHHBHHHBBHBHBBAHBBHABBBHHHHBHBBHAAHHBAHHAHHHBABBHHBAHBHBBHBBBHAHHABAHBHAHBHAAHHHHH

*002379_00040654 AHHBAHBHAAAAHAHHBHBBHHHBAHBBBAHAHHAHBHHHHHBBBHHHAHAHBHHBHHAAAAHHHHBBBBBHHHAHHHBAHABHHHHAHHHHAAAAABAHABABBHHHBHHHHHHHBAHBHHHHHAHAHHBABBAHBHHHHHHA

*002509_00166613 BAHBAHHBHHAHAHHHBHABABBHBHHAHHHAHBHABABHBAHAHHBHAHHHAAAHBABAAHHHHHBBAABAHHAHHBHAHAHBAHAHBBAHAHHHBBHAABHHBHHHHAHHHBHBHHHHHBHAHHHHHHBHBBHHHHHHABAA

*002570_00120626 HAHBAHBAAHHAHBHHHHHBBBBHBBHBHHHBBBBHAAHBAAAHHAAHHAHHBBHBBBHHBHHHAABHHABHAAHHBAHBHBHAHAHHBAAAHHHHHHHHBHHBHHHHHHAHAHHBHBHAHABBAHHHHHHHBAHHAHBBHAHH

*003448_00058553 AHHBAHBHAAHAHAAHHHBBHHHBAHBBBAHAHHAAHHHHHHHBBBHHAHAHHHHBAAAAAAHHHHHBBBBAHHAHBHHAHHBHHHHHHHHHHAAAABAHABABBHHHBHHAHHHHHAHBABAHHAHAHHBHBBHABHAHHHHA

*003889_00012608 BBHABBAHAHHHAHAHHHHHHHHHHAHBAHHHHHHAHBHHHBHBHHAAHHAHHAABBAAAHBHHHBHHHBBHBHBBAHBBHABBBHHHHBHBBHAAHHBAHHAHHHBABBHHBAHBHBBHBBBHAHHABAHBHAHBHAAHHHHH

*004196_00020628 HBHBHBHBAHBHBBBBHBHBHHBAAHAHHHHHHHHHHAHAABHHAHHHHHABHBBBHBHBHAAHHAABHHBHBABHHAHHBAHBHAHABBHHHHBAHBAHBBHHAHHAHHBAHAHBHABHHHHHHHABHHABHAHHHAAHHAHB

*006299_00031395 AHHHAHBHAAHABAAHHHBBHHHBAHBBBAHAHHAAHHHHHHHBBBHHAHAHHHHBAAAAAHHHHHHBBBBAHHAHAHHAHHBHHHHHHHHHHAAAABAHABABBHHHBHHAHHHHBAHBABAHBAHAHHBHBBHHBHAHHHHA

*007550_00006280 HBHBHBHBAHBHBBBBHBHBHHBHAHAHHAHHHHHHHAHAAHAHHHHHHHABHBBBHBBBHAAHHAABHHBHBABHHAHHBAHBHAHABHHHAHBAHBABHBHHAHBHHHBAHABBHABHHHHHHHAHHHABHHHBHHABHAHB

*011981_00000147 HBHBHAHBABBHABAHHAHHBHAABAHBHBHHHHHBHHHBHAHBAHHBBBBHHHBAHHHAHAABHHHHHBBHHHAHHHHBHBHBHABHABBBBAHBHHHABBHHHHAAHABHHHHHHAABHBHBAABHHAHHHAHHHAHHBHBH

*000383_00099313 ABBABABABHHHHAHAAHHBABBHBHAHHHAHHHBHAHHHHAHHHHHHAAHBAAAHHHHBHHHHAHHHAHABBHBB-AAHHHHHABBHABHHAAHHHBBBAHHHHBBHHHBABBBBHHAAAHHABHHHBHHAHHBHHHHBBBHH

*000415_00141189 ABHAHHHHBHH-AAHAAHHAHHAHBHABBHHHHHBAHHBAAAHBHBHHAHHBHHHAHAHAHHHABBHHABHHBHBHBHHAHABHHHBHHHAHHABHBHBBAHAHHBBAAAHHBHHBHHHAHBHHAAABHBHHBBHBHHBHBAHB

*000866_00056381 ABHBHHBABHHBHBHHBHHAHHHAA-ABBBHHBBHHAAHHBHHBABHBBBBAHHHAAHHBHBBAAHHHAAHAAHHHBAHHBHAHHAHBHHBHHAHBHBHAHBHAHBHHBHAHHHHBBHHHHBAHAHHHHHHHBHBBAHHAHAAH

*003355_00010026 ABHBAHHHHBHHHH-HBBAHHBHHBHHAHAAHAAAHHHAAABBAHHBHAHAHHHHHBHHHHAABHHHABABBBHBHHBBHBHHHHAAHHBHHHHBBHBHHBHHHBBABHAABBHABBHHBHBBHHBHAHHBHHHHAHHBAABHH

*000066_00619935 AHHHBH-H-AHA-BABHHBAAHHHHAHHBHBAA-HHHBHHHHBBHAHAABHBAHB-AHAHAABAHAAAHBAHHAHAHHHHHHHH-BHHBHHHH-AHHHHBHHAAH--HAHBHHABABAHBAHAAHAAAAHHHBAH-H-BHHBHH

*001014_00038338 ABAABHHHA-BB-B-HHAHHBAAHHHAHHHBBHAHAHHBBAHAHHAHHBHHHBHA-HBHHBAHHABHHABHAHHHBHHB-AHHB-HHAAHHAA-HAHHHHBABAHBABHBHBBAABHAHHBAHB-HHHB-HHBHHAH-HHHHBH

*000156_00033937 HAAAABHHHHH--HBAAAHAHHHHHHHBHHHHHHHHBBBHBBHBHHAABHAAHBHHABBHHAAHAAHBAHABHHHHHAHBABBHAHHHHHHBBABAAAHBHHAAAHAHBHHAHHHHBBBHAAAH-AHH-HAHHHH-BABHHBHH

*000603_00261358 HBHHAAHH-BHA-B-HAAHHHHHHHHBBAHBHHBHHHAHH-HABHABHAHHAHBHHABBAHHAHHAHAHABBBHHHHAHAHAHHHHHHHHHABAHHAHHBAHAHHBHHHBBABHABHBABHBAH-HBHHBHHBABBHABBHAAB

*002984_00011167 BBHHHBHHBAAH-HHAHHBAABHBBHBHHBAHHAHHHAAAHHHHBAAHAAHBHHHAHHBBHABAHAHBAHHH-AAH-HAHHABB-AHAHBHAHHHHAHHHHAHHHHAAHAHAHHHHAHBBBHAA-HHBHBABBHBBHBAHHHBH

*003561_00029157 ABAHHHHHH-BHHHHHBHHHHHBBBABHABHHHHHBHHAHBABHHHABAHHHHB-AHHHBHHAHBHBA-HBHHABAAHAHHHBAAHHAHHHBABHBAHBHABBHAAHBHHHHAHABBHHAHAHH-HBHA-AAHBBAA-HAHHBA

*001837_00037713 H-HHHBABAAHHHHHAHBBAABHBBHBHHBABHABHAAHAHHHHBHAHAAHHHBHHAHBHBABHHAHHAAHHAAHHHHHHHABBHAHHHBHAAHAHABHHHAAHHHAHBAHABHABAHBHBHAAHBHBH-HBAHHBH-AHHHHH

*010495_00005478 BBHHHBHHHAABAHHAHHBAABHBBHBBHBAHAAHHHAHAHHHHBAAHAAHBHHHAAHBHHABAHAHBAAHHBAAHBHHHHABBAAHHHBHAHHHHAHHHHAHHHHAAHAHAHHH-HBBBBHAAHHHBH-HBBHBBH-AHHHBH

*000020_00348055 HHBBHHAABBHHHHHHBBAHHHAHHHHHAABHHHBHHHHBAAHAHBBHBHBHBHHBHHBBHHAAHBBBBBAABHAHABBHAABHHHAHHHHHHAAHBHAHHABHAAHAABBHBHAAHHHHBHHHBHHHHBAHHAHHBHHBAHHB

*000178_00008539 B-BHBAHHBHHBBHHHHHAHAHHAABHBAHAHBAHAHHHHBHHBAAAHAHHAAHHAHHHHHABABHHBHBHHAHABABHHAAABAHHHHAAHHHHBBABABHHBHHAHHBBAHHHBHBAHBHHAHHBABBHHHAHAHHAHHHBH

*000258_00544052 BHHABBHHAHHHAHAAHHHHHHHHHAHBAHHHHHHABBHHHBHBHHAABHHHHAABBAAHHBAHHHHHHBBHBBBHAHBBHABBBBHHHBHBBHAAHHBAHHAHHBBABBHHBAHBABBHBBBHAHHAHAHBHAHHHAAHHHHH

*000310_00109362 HHABHAHAAHHBABAAHBHBBHHHHBAHAAHHAHBABHHBAAHHHBHHHHHHHHBHHAHHHABHABBABHBHHBHAAHHAAHAHABHHHHHHAAABBAHBHBHHAAHHHBBHBHABHHAHHAHBHHBHAHABBHHHHBAHHHHH

*000322_00348898 HHBHHBHHHHAHBHBHBHHBHHAHAAHHABHHHHHBBAAAHHBHHHBHAAAHHHABAAAHHHHHHHAHAHBHBHHAAHHHBHBHHBHBHHBAHBHHHBHHHBHAAABHAHHBABABHHBHHAAHBBBBAAAHBABHBBHHHBHH

*000541_00121433 BHHAAHHAHBAAAABHBBHBHHHBHBAHHHAAABHHABBAHHBHBHBHHHHHBABHHAABAHHHBHAAHHHHBAAHABHHAAHBAHHBBHAHBABBHAAHBHHHHAAAHHHHHHHAHHHHHHHHHHABHHHHBABHHBAHHAHB

*000707_00260145 HHBHHBHHHHAHBHBHBHHHHBHHAAHAHBHHAHHBBHAAHHBHHABHHAAHBHAHAHAAHBBHHHAHAHBHHBHAAAHHBHBHHBHBHHBAHBHHHBHHHBHAAAHBAHHBABABHHBHHAAHBHHBAAAHBHHHBBHBHBHA

*000714_00150886 HHHHAHAHHHAHHHHABAABAHHHHHHBHAHAHABHHABHBHAHHAHHAHHBAAHHBABAAHHHHHBBHABAHBAHAHHAHAHBAHAHBBAHHAHHBBHAABHHHHHBBAAHHHHBHBHAHHBAHBABHBHHHBHBHHAHAHHA

*000877_00047124 BHHHHAHABBBHAABHBBHBHHBHHHAHHAAAHHABHBHHHHHHHHBHHAHBBAHHHAHBAHHABHHABAHHBAHHABBHAAAHBHHBBBAHBAHBBAAABHBBHAHAHHHHHHAAHAAHHHHHHHABHHHHBAHHHAAHHAHH

*001202_00218172 HHHBABBHAHAAHAHHHHHBHAHBHABHHAHABHHHBHHHBHBHBHHHAHAAHBHBHHHAAHHBHABBBBBAHHAHAABABHBHHHBAHHAHHAAAHHAHHHABBHAHBAHHAHHABHBBHHHHHAHHHBBABHHHBHHHBHHA

*005789_00000848 HHHAABHHHHHHBHBAAAHAHHHHHHABHHHHAHHHBBBHBBHBHHAABBAHHBHHAHBHHAAHAAHBAHABHHHHAABBABBHHHAHHHHBBAHAAAHBHHAAAHAHBHHAHHHHHBBHAHAHAAHHHHAHHHABBHBHABHH

*009221_00003642 BHHBHHAABBBHAABHABABBABHHHAHHHAAHHABBBHHHHHHHHBHHAHBBAHHHAHBAHHABHAABAAHBAHHABBHHAAHBHHBBHAHBAHHBHAAHHBBHHHHHHHHAHAHAHAHHHHBBHABHHHABHHHAHHHHAHH

*000032_00543121 HBHBHBHBABHHHBBBHBHBHHHHAHAHHAHHHHHHHAHAAHAHHHHHHHABHBBBHBBBHAAHHAABHBBHBABH-AHHBHHBHAHABHHHAHBAHBABHBHHAHBHHHBAHABBAABHHHHHHHAHHBABHHHBHHABHAHB

*000038_00976340 BBHABBAHAHHHAHBAHHHHHHHHHAHBAHHHHHHABBHHHBHBHHAABHHHHAABBAAAHBHHHHHHHBBHBHBBAHBBHABB-BHHHBHBBHAAHHBAHHAHHBBABBHHBAHBAHBHBBBHAHHAHAHBHAHHHAAHHHHH

*000453_00354114 BBHABBAHAHH-AHAHHHHHHHHHHAHBAHHHHHHAHBHHHBHBHHAAHHAHHAABBAAAHBHHHBHHHBBHBHBBAHBBHABBBHHHHBHBBHAAHHBAHHAHHHBABBHHBAHBAHBHBBBHAHHABAHBHAHBHAAHHHHH

*000510_00381093 HAHAABHHHHHHBHBAAAHAHHHHHHABHHHHAHHHBBBHBBHBHHAABHAAHBHHABBHHAAHAAHBAHABHHHH-AHHABBHHHHHHHHBBABAAAHBHHAAAHAHBHHAHHHHHBBHAAAAAAHHHHAHHHHBBABHHBHH

*000601_00084692 HBABHHBABBHBHBHHBHHAHHHAAHHBBBHHBBHHAAHHBHABABHBHBBHHHHAAHABHBBAAHHHHAHAAHHHBAHHBHAH-AHBHHBHHHHBHBHAHBHAHBHHBHAHHHHBHHHHBBAHAHHHHHBBBHBBAHHAHAAH

*000753_00292029 HBAHHHBABBHHHB-HBHHHHAHAHBHBBBAHBBHHAAHAHHAHABABHBBHHHAAHBABHBBAAHHAAAHAAHHHBAHHHHAHHAHHHHBHHHHHHBBAHBBAHHHHBHAHHHHBHHHHBBHHAHHAHHHBBHBBAHHAAAAH

*002703_00086940 HBHHAAHHBHHBABHHHBABAAAHBHBBAAHHHBHHAAHHHBBHHABHBHHHHBHHHBHAHAHAHHHAHAHHHHHHAAHHHBHHHABHHHBHHABHAHBBHBAHAB-HHBAABHAAABABHHHAHHHHHHAHBABHHHBHBAAB

*003975_00067523 BAHAHHAAAHB-BHHBBHHHHHHHHABAHAAHBHHBHHABHHABHHBAAAHHHHAABHHBHHHHHHBHHAHAAHHHHBHAHAHBHHBAAHBBBHHHABHHBBAHBHABABABHABHAHHHHHBHHAHAHHBBBHAHHHAHBAHB

*000421_00339806 ABHBHHBHBHABHBHHBHHAHAHAAAABBBHHHBHHAHHHBHABABHBBBBAHHHAAHHBHBBAAHHH-AHAAHBH-AHHBHAHBAHBHABHHAHBHBHAHBHAHBHHHHAHHHHBHHHHHHAHAHHHHHHHBHBBAHAAHAAH

*000511_00123030 ABHBHHBHBHABHBHHBHHAHAHAAAABBBHHHBHHAAHHBHABABHBBBBAHHHAAHHBHBBAAHHHAAHAAHBHBAHHBHAHHAHHHABHHAHBHBHAHBHAHB-HHHAHHHH-HHHHHHAHAHHHHHHHBHBBAHAAHAAH

*006342_00005274 AAHHHHHHBBHBABHHBBHHHHBAB-BHHAHBBBHBBHAAHBHBABHAHHHHHHHHHHHHBBBBHAHHAAHHHABHAAHHA-BHHHHBBAABHHBHBBBHAABABBHHHBABHBAHHAHHHHHHHHHHHHBHHABBHABBHBHH

*000023_00480946 ABAAHHBHAHBHABHHHHHHBHAAHHAHAHBBHHAABHBHAHHHAHHBBHHHHHABHBBHBAHAHBHHABHHAHBBHHBBHHHBAHHAAHBAAHHAAHAHBABAHHHHHHHBBAABBHHBHHHBBHAABHHHBHHBHHHHHABH

*000080_00527836 ABBABABABHBAHAHAAHHBAHBHHHAHHAHHHHBHAHHHHAHHHHHHAAHHHHAABHHHHHHHHHHHHHABBHBBBABHHBHHHBBHABAHAAHHHHBBAHHHHBBHHHHABBBBHHAAAHBABHHBBBHAHABBAHHBBHHH

*000622_00221798 ABHBHHBABHABHBHHBHHAHHHAAHABBBHHHBHHAAHHBHABABHBBBBAHHHAAHHBHBBAAHHHAAHAAHHHBAHHBHAHHAHBHHBHHAHBHBHAHBHAHBBHHHAHHHHBHHHHHBAHAHHHHHHBBHBBAHHAHAAH

*000866_00168643 ABHBHHBABHHBHBHHBHHAHHHAAHABBBHHBBHHAAHHBHABABHBBBBAHHHAAHHBHBBAAHHHAAHAAHHHBAHHBHAHHAHBHHBHHAHBHBHAHBHAHBHHBHAHHHHBHHHHHBAHAHHHHHHBBHBBAHHAHAAH

*001007_00080945 ABHAHHABBHHHHHHAAHHAHHHHHBHBBBHHHBBAHHHAAAHBHBHBHHHHHHHAHAHAHHHBBHHAABHHBABHBHHABHHAHHBHHHHHHHBBBABBHHAHBHBHAAHHBHABAAHHHHHHAAABHBBHBHBBHHBBBABB

*001385_00055747 ABHBHHBHBHABHHHBBHHAHAHAAHAHBBHHABHHAAHHBAHBAHHBHBBAHHHAAHHBHBBAAHHHAAHAAHBHBHHHBHAHHAHHHHBHHAHBHBBAHBHAHBBHHHAHHHHHBHHHHHAHAHAHHHHABHBHABAAHAAB

*003489_00022220 ABBBABHABHABAHHAHAHHHHHBHHABAHHHBBHHHAHHBHHBBAHHHBHAHBHHBHHHHAHHAHBBHHHHHHHHHAAHHHABABHBBHHHHABHABHHBAHAAAHABHBBHBHAHABHHABHHHHBHBABHHBHBBHHHAAB

*002112_00003986 HHAAHAHHHAHHHA-BHABBHAHHAAAHBABHAABHHBABHHHHHHAHAHHBAHABHHBAHAHAHHAH-HHHAAHB-BAHHHBB-BHAAHHAA-HHBAHBHH-AHB-BHHH-HAH-HH-AABHH-BHH--HAHAAHH-H-BHAH

*000915_00048163 HHBBBABHAHHH-A-AHABHHAHHB-AHHHHHAHHBHAHB-A-HHHBHAHHBBHBBHHHHHBHHHABH-BBBHHHHAHHHB-AHHHABHBHAH-HAHBBHHHBHHA-ABAHHBHAA-HAHBAAH-BHH-AHBBHHAHBHBABA-

*000025_00923463 ABAHHHAH-HHHAH-HBHHHHHABB-BAABHHAHHBH-AHBABHBHHBAHHHHB-AHBHBHHAHHHBAHH-HHHBA-H-HHH-A--HAHHHBABBBAHBHABHHHA-B-AHHAHAHAHAABAHH-HHAAHHHHABAA-HAHABA

*000616_00054455 HHABHAHBAAAAHHAAHHBBBAH-HBAHHABBA-HHAHHH-HBABBHAAHHHBAAHHHHABBHHHAHH-H-HAHAHAHAHHAAHBHHHAAAAABHHHABAAHBHHHBHHBHHHH--HH-HHAHBHBAH-A-HHHAHH-HHHBAB

*000446_00141272 AHHAHHAHBHAH-BHHH-BHA-BBHBAHBAHBAAAHAHHHAHHAHHHBHAHHBBHHABAHBBBHHBHB-H-HHBHBBHAHBHBHBHHAAHHAHBAHAABHHAHHHA-HBHHABBBHAHHBHHAA-HHHB-HHBHHBHBBHHAHH

*001023_00039884 A-HBHHHHAHBHAHHHBHBHHBH-ABBBBHHHAHHHHBABHHAAHBHAHBAAHHABBBHBHHHHBHBH-HAABAHAHHAHHH-BBBAHHHAAABAAHAHHHABBHBBHHABAHHHAHAHHHHBHHBAHA-HHHHHAB-HHAAH-

*000021_00981124 BHHHHAHABBHHAABHBBHBHHBHHHAHHAAAA-ABHBHH-H-HHHBHHAHBBAHBHAHBAHHABHHAHAHHBAHHABBHAAAHHHHBBHAHBAHBBAAABHBBHAHAHHHHHHAAHHAHHAHHHHABAHHHBAHHHBAHHAHH

*002611_00006570 HAHBAHBAAHH-HBHHHHHBBBBAB-HBHHHBBBBHAAHHAH-HAAAAHAHHBBHBBHHHBHHHAABH-ABHAAHHBAHBHBHAHAHHBAAAHHHHHHHHBHHBHHHHHHAHAHHBBBHAHABBAHHAHHHHBAHHAHBHHAHB

*003048_00034244 HBHAHABHB-HB-B-HHBHHB----HHHHHBBA-BHH-BBBHHABHBH-AHBHHBHBBH-HBHHHBBBB-HBBABB-H-BH--H-BHBHHHHHHB-HBHHHBH-HH--B--BHBB-BB-HHB-H-BB--B-BHHB-B-HHBHH-

*000541_00274744 BHHAAHHAHBAAAABHBBHBHBHBHBAHHAAAABHHABBAHHBHBHBHHHHHBABHHAABAHHHBHAAAHHHBAAH-BHHAAHHHHHBBHAHBAHBHAAHBHHHHAAAHHHHHHHAHAHHHHHHAHABHHHHBHBHHBAHHAHB

*000809_00233374 HHHHHAHHHAHAAAHHABAHHHAHHBAHAAHAHHBBBHHAHHHHHHHBHHBABBAABHBHAHBBHHBHBAHBHBAHBBHHHABBBAHAAHHHHHHHAHBHHHHHHHBHBAABBHA-HHAAHABHAHHBBBHHAHBABAHHHHAH

*000216_00252148 HBHBHHHBHBBHBHBHHHHHHHBAAHHHHHHBBHBHHABHHBHHAHHHHHABHHHBBBA-HHABBAABHHBABABB-HHBBAHBHAAABBHBHHHAHBAABBAHHHHAHBBAHAHBHHHHHHBHAHAABHHHHABHHHAHAHHB

*000453_00241472 BBHABBAHAHH-AHAHHHHHHHHAHAHBAHHHHHHAHBHHHBHBHHAAHHAHHAABBAAAHBHHHBHHHBBHBHBBAHBHHABBBHHHHBHBBHAAHHBAHHAHHHBABBHHBAH-ABBHBBBHAHHABAHBHAHBHAAHHHHH

*000493_00135817 AHHBAHBBAHHAHBAAHHBHHHBHABBBBHABHABHHHABHBBHHHHHAAAABHHHHBHHBAAABHBB-AHAHAHBAHHHHAHBHAHHHHHAAHHHBHHBHHHHHH-HBBBHBHABBHAAHHHHHBAABBHHHHHAHHHBHAHA

*000456_00287476 ABHHHHHHBHABABBHBHHHHHHHHBHHBBHHBAHHBHABHBABHABBABHHABHBBHBHHBHHHHHA-BHABBAHAABHBHHBHAHHHABHBHHHHHBAHBHHHA-BBHBAHHAA-HHHHABHHHBBHAHHHBBHBAHHHABA

*000745_00275882 ABHBHHBABBHB-BHHBHHAHHHAAHABBBHHBBHHAAHHBHABABHBBBBAHHHAAHHBHBBAAHHHAAHAAHHH-AHHBHAHHHHBHHBHHAHBHBHAHBHAHBHHBHAHHHHBHHHHHBAHAHHHHHHBBHBBA-HAHAAH

*002045_00062007 ABAHAHHHABHHBHAHHBABHHHHBHHAHAHAAAAHBHAAAB-AHHBHAHAHHHHHBHHHAAABHHHAAABHBHBHABHHBHHABAAHHBHHHHBBHBHHBHHHBB-BBAABBHH-HHHHHBBHHBHHHABHHHHAHHBHABHA

*000000_00580982 HAHHHHHBBBHBABHBBBHBHHHHHHBHAAABHBHHBHAAHBHHHBHHAAHAHBBAAHHHBBBBAABBAAHBHAHHHAHHHABHHHHHHHAHHHBAHBBBAABAHAAHHBHBHHAAHHHHHHAHHHBBHHHHHHBBHBBHHBHA

*000000_00814989 HAHHBHHHBBHBABHBBBHBHHHHHHBHAAABHBHHBHAAHBHHHBHHAAHAHHBAAHHHBBBBAABBAAHBHAHHHAHHHABHHHHHHAAHHHBAHBBBAABAHAAHHBHBHHAAHHHHHHAHHHBBHHHHHHBBHBBHHBHA

*000056_00208657 HBHHHBHHAHAHBHBHBHBHHBHHAABHHHHBAHHBBHAAHBHHHABAHAAHBHAHAHHABBBHHHAHAHBHHBHAAAHHBHBHHHHBHHBAABHHHHHHHHHAAAHBHHHBABABHHBAHHAHBHHBAAAHBHABBBHHHBHA

*000058_00154385 BBHABBABAHHHAHAHHHHAHHHHHAHBAHHHHHHAHBHHHBHBHHAAHHAHHAABBAAAHBHHBBHHHBBHBHBBHHBBHABBBAHHHBHBBHAAHHBAHHAHHHBABBHHHABHHBBHBBBHAHHABAHHHAHBHAAHHHHH

*000156_00008757 HAAAABHHHHHHBHBAAAHAHHHHHHABHHHHAHHHBBBHBBHBHHAABHAAHBHHABBHHAAHAAHBAHABHHHHHAHBABBHHHHHHHHBBABAAAHBHHAAAHAHBHHAHHHHHBBHAAAHAAHHHHAHHHHBBABHHBHH

*000252_00561505 HBHHAAHHBBHHABHHABABBHAHHHBBAHBHHBHHAAHHHBAHHABHBHBBHBHHHBHAHHHAHAHAHAHHAHHHHAHHHHHHBHHHHBBHBABHAHBBHHAHABHHHBAABHAAHBABBHHBHHBHHHAHBABHHHBBHAAB

*000257_00397691 AHABHAHHAHHBBAHHBHBBHHHAAHHBHHHHHBHHBBHBHAAAABHAHBAHHHABHBHBHAABAHBHHHAABABABAAHHHHHHBAHHBBAAAHHAAHHHAABHBHHHAHHHHHHHHAHHHHHHHAHABHHHHAAHAHHAAAA

*000287_00060442 HBHBABHAAHHHABHBHBHBHHHHAHABBAAHBBHAHAHAAHHHHHHHHHHBHBBBHBBHHHAHBHAABBBHBABHHAHHBHHBHAHABAHHHHBAAHABHBHHAHBHHHHABABBHABHHHHHHHAHHBABHHHHHHABHAHB

*000291_00080892 BAAHAHAHBAHHAAAHHHAABAHHAHAHHHHAHBHHABHBHAHAHAABHHHHABABHBHBABHHHBHBHAHHBHAHAAHHHBBHAAHHHBHBHAAHHABHBHHAHBBHHABHHAHAAHHBHHHBAAAHHBHHBAHBHHHHHHHB

*000374_00393367 AHHBHHABHHHABHAAHBBAHHHBHBBHHBABHABHHHAHBBBHBHHHAAAABHHHHHHHBAAHHABHAAHAHABBHHHHHAHBHAHHHBHAAHAHBHBHHHHHAHAHBBHHBHABAHHABHHHHBAHBBAHAHHBHBAHHAHH

*000470_00198234 AHHHAHHBHHHHBHAHHHABHHBHHHAHAAHAHHBAAAHHBHAAHHBHBHABHBAABHBHAHBBAHBAAHBABHHHHHAAHBHABHHAAAHHHHBBAHHAHAHAAHHAHHAAHBHHBHHABHBHHHHAHAAHHHHHBABHHAHB

*000490_00091109 BAHBAHAHHHAHHHHHBHABABBHBHHAHHHAHBHABABHBHHAHHBHAHHHAAAHBABAAHHHHHBBAABAHHAHABHAHAHBAHAHBBAHAHHHBBHAABHHBHHHHAHHHBHBABHHHBBAHHHHHHBHHBHHHHHHABAA

*000601_00076120 HBABHHBABBHBHBHHBHHAHHHAAHHBBBHHBBHHAAHHBHABABHBBBBHHHHAAHABHBBAAHHHHAHAAHHHBAHHBHAHHAHBHHBHHHHBHBHAHBHAHBHHBHAHHHHBBHHHBBAHAHHHHHHBBHBBAHHAHAAH

*000657_00147422 HBAHBHHHHAHAHHHBHAABHBBABHAABHHBHHHBBHHBHAHHAHAAHBHBHAAHBHHHBAHAABAAAHBHBBHHBHAAHAAHHBAHHBAHHBHBHAHBHBHBAABHHHHABHHAHHHHHHBHHHHHHHAHBAHHHBHAAHHB

*000778_00185881 BAHBAHHBHHAHAHHHBHABABBHBHHAHHHAHBHABABHBAHAHHBHABHHAHHHBABAAHHHHHHBAAHAHHBHBBHHBAHBABAHBBAHAHHHBBHAHBHHBHHHHAHHHBHBHBHHHBBABHHHHHBHBBHHHHHHABAA

*000839_00057166 BAHBAHHBHHAHAHHHBHABABBHBHHAHHHAHBHABABHBAHAHHBHAHHHAAAHBABAAHHHHHBBAABAHHAHHBHHHAHBAHAHBBAHAHHHBBHAABHHBHHHHAHHHBHBHBHHHBBAHHHHHHBHBBHHHHHHABAA

*000963_00193653 HBAABAHAABHBAHAHHHABBHHHHHABAAAHHBBHBHHBAAHHHHHBHHHHBHBAHHAHHABBHBBABHBHHHAAAHBAHHABABBHHHHHAHHBHAHHAHHAHAHHHBHHBHHBHHAHHHHBHHBBAHHHBHBAHBAHBHHB

*001190_00270336 BBHHHBHAHHAHHHHAHBBAABHBBHBHBHAHAAHHHAHHHHHABAABAAHBHHHHAHBHHHBAHAHBAAHHBAAAAHHAHABBHAHAABHAAHHHAHBHAAHHHHAAHAHAHHHHABBBBHAAHHHBHHHHBHHBHBHHHHBH

*001248_00073216 HBHHABHAHHAHHHHAHBBAABABBHBHBHAHAAHHHAHHHHHABAABAAHBHHHHAHBHHHBAHAHBAAHHBAAHHHHAHABBHAHAABHAAHHHAHBHAAHHHHAHHAHAHHAAHBBBBHAAHHHBHHHHBHHBHBHHHHBH

*001404_00074401 BAHBAHHBHHAHAHHHBHABABBHBHHAHHHAHBHABABHBAHAHHBHABHHAHHHBABAAHHHHHHBAAHAHHBHHBAHBAHBABHHBBAHAHHABBHAHBHBBHBHHHHHHBHBHBHHHBBABHHHHHBHBBHHHHHHHBAA

*001536_00164233 HBHHHBHBAHBHBBBBHBHHHHBAAHAHHAHHHHHHHHHAAHAHAHHHHHABHBBBHBHBHAAHHAABHHBHBABHBAHHBAHBBAHABBHHHHBAHBABHBHHAHBHHHBAHAHBHABHHHHHHHABHHABHHHBHAABHAHB

*004052_00018414 BBHABBAHAHHHAHAHHHHHHHHHHAHBAHHHHHHAHBHHHBHBHHAAHHAHHAABBAAAHBHHHBHHHBBHBHBBAHBBHABBBHHHHBHBBHAAHHBAHHAHHHBABBHHBAHBABBHBBBHAHHABAHBHAHBHAAHHHHH

*000346_00081567 AAHHHHHHBBHBABHHBBHBHHBABABHAAHBHBHBBHAAHBHHABHAHAHHHHHHHHHHBBBBHABHAAHBHAHH-AHHAABHBHHBBAABHHBHBBBHAABABHHHHBHBHBAAHHHHHHHHHHHHHHBHHHBBHBBBHBHH

*000792_00173315 AAHHHHHHBBHBABHHBBHBHHBABHBHAAHBBBHBBHAAHBHBABHAHAHHHHHHHHHHBBBBHAHHAAHBHABHHAHHAHBHHHHBBAABHHBHBBBHAABABB-HHBHBHBAHHAHHHHHHHHHHHHBHHABBHHBBHBHH

*001529_00215192 AHHHABAHAHHHBHHABHBBBHHHHBHAAHHABHBHBAHBHHBHHHBHAHHABAHHHHBAAHHBHBBBHBAABHHHHHAHHBBHBHHBHAHAHBBHBAHBBBHAAHHHHHHHBAABHBBABHHBBHHBAHAHHHAHBHBHHHHH

*004939_00012510 ABBABABABHHHHAAHAHHBABBHBHAHHHHHHHBHAHHHBAHBHHHHAABBAAAHHHHBHHHBAHHHAH-BBHBBBAHHHHHHABBHABHHAAHHHBHBHHHAHBBHHHBABBHABHAAAHHABHHHBHHAHHBHHHHHBBHB

*004969_00005826 BAHH-HHHABHB-BHHHHABABBHH-HAHAABBB-HHBBHHHAAABHHAHHAABHHHHHHBAHHAHHA-BHHHAH--HB-H--B-A---HBHBABAHAAHAHHHHAAHHAHHHH--BBHBAHHABHA-HHHH--AHA-BA-AHB

*003254_00088305 HBHBHAHB-BBH-B-HHAHHBHAABAHBBBHHHHBBHHHB-AHBAHHBBBBHHHBAHHHAHAABHHHH-BBHHHAH-HHBH-HB-ABHABBBB-HBHHHABBHHHHAAHABHHHHHHAABHBHBAABHBAHHH-HHH-HHBHBH

*000045_00090374 HHHHBHAH-AHAAHAHHBBBAAHHAHHAHBBHHABAHHHBAB-HHHAHHHHHBAABHHHHHABHABHHAABHAHHBHHHHA-BBAHAHAHABAHHBHABHBBAABH-BHHHABHHAHHHHBBHABAAHB-HHHBHHB-HHAHHH

*000167_00574207 HHHBHAHHABB-HH-HAAHBBHAABAHBHBHHHHHBHHHBAAHAAHHBBHBBHHBHHBHHHAAHHHHH-BBABHAB-HHBHBHBHAHHABBBBAHBHAHABBHAHHAAHABHHHHHHAABHBHBAABHBAHHHHHHHHHAHHBH

*003481_00062489 BHHHAAAHABHABHHAHHHBABHHAHHABBBHAHBAHBBBBB-HHABHBHAHAHHBHBHBHHHHHHHHHHHBAAHHAAHHAAHHBHHAHHHHB-HBHAAAABHBABAHBBHAHHBAAHAHHBHH-BHAHHABHHBBH-HBHHHH

*000465_00330109 AHAHHHHAAHHHHHAHBHBHHHBHBHBHAHAHAAHBBH-HBA-HHHHBAHHHBBAAHHHHHBABHHBHAA-HBABHAHAAHH-AHBHABBHBA-HBHHBHHBHHAAABHHHHAHABAAHAHAHHBHBHAHAAHHHAAAHAHHHA

*001107_00096283 BBHHAHHHBAHHHBHHAABHHHBHHHBHAHHHHBHABAHHHH-BHABHHHHAHBHHABBAHHABHAHABHABBHHHAAHHBBHHHBHHHBHAB-HAAHBBAAAHBH-HHBHABHAHBBHBHBAHAHBHHA-HHAHBB-BBHHAB

*009349_00003107 HBHBHABBHBBHHBAHHAHHBHAABAHBHBHHHHHBHHHB-AHBAHHBBBBHHHBHHHHAHAABHHHH-BBHHHAHBHHBHBHB-ABHABBBB-HBHHHABBHHHHAAHABHHHHHAAABHBHBAABHHAHHHAHBH-HHBHBH

*000096_00637523 HHHAAHBHHBHHBHAHHBABHHHHHHAHAHHAHHBAAABHHHABHHBHBHABHBAAHHBHAHBBAHHAHHBABHHBHHAAHBHABHHAAAHHHHHBHHHAAABAHHHAHHAAHBH-BBHABBHHAHHABAHHHHHBB-BHHAHB

*000258_00542589 BHHABBAHAHHH-HAAHHHHHHHHHAHBAHAHHHHABBHHHBHBHHAABHHHHAABBAAHHBAHHHHHHBBHBBBHAHBBHABBBBHHHBHBBHAAHHBAHHAHHB-ABBHHBAHBHBBABBBHAHHAHAHBHAHHHAAHHHHH

*000504_00261660 HHABHHHAHHHBHHAAHBHBBHHBBBAHAAHHAHBAHHHHAAAHBBHHHHHAHHBHBAHHHABHABBABHHHHBHA-HHAAHAHHHAHHHHHAAABBHABHBHHAAHHAB-HBHAHHBHHHABBHHBHABABHHHHHBABABHH

*001783_00167769 HHAAABHHBHHABHHAAAHAHHHHHHBHAAHHHHHHBB-B-BHHHAAHBHAAHBHBABBHHHAHHAHBAHABBHHHHAHHBHBHHHHHHBHBBABAAHBBHAAAHBAHBHHAHHHHHBBHAAAHAABHBHHHHHHBBABHHHAH

*000154_00616339 AHHBAHBBAHHAABAABHBHBHBHABBBBHABAAHHHHABHB-HHHHHAAAAHHHHHBAHBAAABHBB-HAAHAAHAHHHBHHBHAHHHHHAAHHHBHHHHHHHHHHHBHBHBHHBBAHAHHHB-BAAHBHHHBHAHAHBHAHH

*000877_00182784 BBHHHAHABBHHAABHBBHBHHBHHHAAHAAAHHABHBHHHHHHBHBHHAHBBAHHHAHBABHABHHABAHHBAHH-BBHAAAHBHBB-HAHBAHBBAAABHBBHAHAHHHHHH-AHAAHHHHHHHABHHHHBAHHHHAHBAHH

*000020_00184448 HHBBHHAABBHHHHHHBBAHHHAHHHHHAABHHHBHHHHBAAHAHBBHBHBHBHHBHHBBHHAAHBBBBBAABHAHABBHAABHHHAHHHHHHAAHBHAHHABHAAHAABBHBHAAHHHHBHHHBHHHHBAHHAHHBAHBAHHB

*000062_00076331 HHHAHAAABHHHAAHHABHHHAAHHBAHAAHAHHBBHHBAHHHHHHABHHBHBHAHBHHHAHBHHBBAHHHHAHBHBBAHHHBBHAHAAHAHHABAHABHHBHHHBBHBAHBBHHHHHAABHBHAHHBHBHHHHBABABBHHAB

*000062_00451118 HHHAHAAABHHHAAHHABHHHAAHHBAHAAHAHABBHHBAHHHHHHABHHBHBHAHBHHHAHBHHBBAHHHHAHBHBBAHHHBBAAHAAHAHHABHHABHHBHHHHBHBAHBBHHHAHAABHBHAHHBHBHHHHBABAHBHHAB

*000067_00391618 HHBHHBHHHHAHBHBHBHHBHBHHAAHHABHHAHHBBAAAHHBHHABHAAAHHHABAAAHHHBHHHAHAHBHBHHAAAHHBHBHHBHBHHBAHBHHHBHHHBHAAABBAHHBABABHHBHHHAHBHHBAAAHBAHHBBHHHBHH

*000082_00038306 HHHBHBBHHAHBBHAHAHHAHHBBBAHHHAABBBBBHHAHBBHHBHHHBABHBHBAHHHBBBBHABAHBHBAAHBBBBHBBHHHHHHHHHHHHAHAHHHAHBHBHAAHHABAHHHAHABBBBHHHHBHBHHHAHBHHAHHHAHH

*000127_00072224 BHHAHHBHHBHBAHAAAHAAHHAAABAHHHHHBBHHHBBBAHAHAHHBHHBHHAAHHBHAHAAHHAHAHHABHHBHAHBHBHHHAHHHHHHAAHAHABHABBHBHHAHAAHHBBABBHHBBHHHBHAABBHBAHHHHHHBHHBB

*000257_00462624 HHABHAHHAHHHBAHHBHBBHHHAABHBHHHHHBHHBBABHAAAABHAHBAHHHABHBHBHHABAHBHHHAABABAHAAHHHHHHHAHABHAAAHHAAHHHAABHBBHHAHAHHHHHHAHHHHHHBAHABHHHHAAHAHHAAAA

*000477_00132307 HHABHHHAHHHBHHAAHBHBBHHBBBAHAAHHAHBAHHHHAAAABBHHHHHAHHBHHAHHHHBAABBABHHHHBHHAHHHAHAHAHAHHHHAAAABBHABHBHHAAHHAHBHBHAAHBHHHAHBHHBHABABHHHHHBABABHH

*000503_00271905 HHAABAHAABHBAHAHHHHBBAHHHHABAAAHHBBHBHHBAAHHHHHHHHHHBHBHHAAHHABBHBBABHBHHBAAAHBAHHABABBHHHHHAHHBHAHHAHHAHAHHHBHHBHABHHAHHAHBHHBHAAHHBHBHHBAHBHHB

*000610_00276937 HHAAHAHBBBAHHBABHHABAHBHHBHHHHHHAHAHHBHBBHBHHHHAHHABHHBHHHAAHABABABHHHBBHAAHAHHHAAHHAHAHBAAHHBHAHAHBHHAAHAHHAAHHHABHHHABBBHAHBAHHHHHHBAHHHHBABBH

*001054_00204618 HHHHAHAHHHABHBHABAHBHHHHHAHBHAHAHABHHABHBHAHHAHHAAABAAHHBBBAAHHHHABBHABAHBAHAHHAHAHHHHABBBAHHAHHBBHHABHHHHHBBAAHHHHHABHAHHBHHBABHBHHHBHBHHAHAHHA

*001215_00023602 HHBBHHAABBHHHHHHBBAHHHAHHHHHAABHHHBHHHHBAAHAHBBHHHBHBHHBHHBBHHAAHBBBBBAABHAHABBHAABHHHAHHHHHHAAHBHAHHABHAAAAABBHBHAAAHHHBHHHBHHHHBAHHAHHBHHBAHHB

*002172_00077408 HHHHABAHAHHBAHHBHHHHBHBHAHHABHBHHABHHAHABHAHHAHHHAHAAABHBHHHHBBHHABBBHBHAHAHAHHBAHHHHAHBHBHHHAHAHBHHBHAHHHHBBAABAAHHHBBAAABBHABHHHHHAHHBHAAAHHBA

*002732_00029104 HHBBHHAABBHHHHHHBBAHHHAHHHHHAABHHHBHHHHBAAHAHBBHBHBHBHHBHHBBHHAAHBBBBBAABHAHABBHAABHHHAHHHHHHAAHBHAHHABHAAHAABBHBHAAAHHHBHHHBHHHHBAHHAHHBHHBAHHB

*003056_00030030 BHHAHHBHHBHHAHAAAHAAHHAAABAHHHHHBBHHHBBBAHAHAHHBHHBHHAAHHBHAHAAHHAHAHHABHHBBAHBHBHHHAHHHHHHAAHAHABHABBHBHHAHAAHHBBABBHHBHHHHBHAABBHBABHHHHHBHHBB

*003872_00027435 HHHHAHAHHHABHBHABAHBHHHHHAHBHAHAHABHHABHBHAHHAHHAAABAAHHBBBAAHHHHABBHABAHBAHAHHAHAHHHAABBBAHHAHHBBHHABHHHHHBBAAHHHHHABHAHHBHHHABHBHHHBHBHHAHAHHA

*007910_00002447 BHHAAHHAHBAAAABHBBBBHHHBHBAHHHHAABHHABBHHBHHBHBHAHHHBABHHAABAHHHBHAAHHHHBAAHABHHAAHBHHHBBHABBABBHAAHBHHHHAHAHHHHHHHAHAHHHHHHHHAHHHHHBABABBAHHAHB

*000032_00659865 HBHBHBBBAHHHHBBHHBHBHHHHA-AHHAHHHHHHHAHAAHAHHHHHHHABHBBBHBBBHAAHHAAHABBHBABHHAHHBHHBHAHABHHHHABAHBABHBBHABBHHHBAHABBHABHHHHHBHAHHBABHHHBHHABHAHB

*000135_00611696 AHHHHBABHHHA-HHAHBBAABHBBBBHHBABHABAHAHABHHHBHHHAAHHBHHHHHHHBAHHHAHHAAHHHAHBBHHHHABBHAHHHBHAAHAHABHHHHHHAHAHBBHHBHABAHBHHHHAHBABBBABAHBBHBAHHAHH

*000832_00150109 AHHHHHHAAHHHABHHHBBBHHHHBHHBHAAHBBHAHAHAHHHHHHHAHBHBHHBBHHBHHHABHHAHBBAHBABHHHHBBHHHAAAABAHBHBBHAHHBHBHHHBBBHBHHBABHHAHHBAHH-HHBHBHBHBAABBABAAHB

*001196_00184199 BBHHHBHHHAAAHHHAHHBAABHBBHHHHBAHAAHHHAHABHHHBAABAAHBHHHHAHBHHHBAHAHBAAAHBAAHHHHAHABBAAHAHBHAAHHHAHBHAAHHHHAAHAHAHHHHHBBBBAAAHHHBHHHBBHHBH-AHHHBH

*005192_00018607 HBHHAAHHBBHHABHHABABBHHHHABBAHBHHBHHAAAHHBAHHABHHHBBHBAHHBHAHHHAHAHAHA-HBHHHBAHAHHAHBAHHHHHABAHHAHHBHHAHHBHHHBBABHAAHHABBBHBHHBHHBHHHABBHHHBHAAB

*005623_00010867 AHHHHHHAAHHABHHHBHBBHAHHBABAAAHHAHAHBAAHBHBHHHHHAHHHBHBAHHHHHBABHHBHAAHHHAHAABAAABBHHBHABHHHHBBHHHHHHBHHAAHBBHABAAABHHHABAHHBHBBAHHABAH-HAHHHHHA

*000386_00062025 ABHHHAHHHHAHAHHBBBHHHBABHHBHABHHBHHBHHAABHBHBHHBAHAHHBHAHBBBHAHHHHHH-HHHBHHABHAHHABAABH-HHHBHHHBAHBHABBHHAHHAAHAABAHAAAAHHHHBHHAAHHAHHBAAAHBHABA

*004558_00027644 ABHBHHBHBHABHHHBBHHAHAHAAHAHBBHHABHHAAHHBAHBAHHBHBBAHHHAAHHBHBBAAHHH-AHAAHBHBAHHBHAHHAHHHHBHBAHBHBBAHBHAHB-HHHAHHHHHBHHHHHAHAHAHHHHABHBHABAAHAAB

*000013_00910363 ABHAHHAHBHHHAHHAAHHAHHHHHBHBBHHHHBBAHHBAAAHBHBHBHHHHHHHAHAHAHHHBBHHAABHHBABHBHHAHHBHHHBHHHAHHABBBABBHHAHBBBAAAHHBHABAHHAHHHHAAABHBBHBHHBHHBBBAHB

*000023_00386410 ABAAHHBHAHBHABHHHHHHBHAAHHAHAHBBHHAAHHBHAHHHAHHBBHHHHHABHBBHBAHAHBHHABHHAHBBHHBBHHHBAHHAAHBAAHAAAHAHBABAHHBHHHHBBAABBHHBBHHBHHAABHHHBHHBHHHHHABH

*000386_00045115 ABHHHAAHHHAHAHHBBBHHHBABHHBHABHHHHHBHHAABHBHBHHBAHAHHBHAHBBBHAHHHHHHHHHHBHHAHHAHHABAABHAHHHBHHHBAHBHABBHHAHHAAHAABAHAAAAHAHHBHHAAHHAHHBAAAHBHABA

*000659_00269734 ABBBABHABHABAHHAHAHHHHHBHHABAHHHBBHHHAHHBHHBBAHHHBHAHBHHBHHHHAHHAHBBHHHHHHHHHAAHHHHBABHBBHHHHABHABHHBAHAAAAABHBBHBHAHABHHABHHHHBHBABHHBHBBHBHAAB

*000866_00060273 ABHBHHBABHHBHBHHBHHAHHHAAHABBBHHBBHHAAHHBHABABHBBBBAHHHAAHHBHBBAAHHHAAHAAHHHBAHHBHAHHAHBHHBHHAHBHBHAHBHAHBBHBHAHHHHBHHHHHHAHAHHHHHHBBABBAHHAHAAH

*000897_00322394 ABHBHHBABHABHBHHBHHAHHHAAHABBBHHHBHHAAHHBHABABHBBBBAHHHAAHHBHBBAAHHHAAHAAHHHBAHHBHAHHAHBHHBHHAHBHBHAHBHAHBHHHHAHHHHBBHHHHBAHAHHHHHHBBHBBAAHAHAAH

*001796_00030749 ABHAHHABBHHHHHHAAHHAHHHHHBHBBBHHHBBAHHHAAAHBHBHBHHHHHHHAHAHAHHHBBHHAABHHBABHBHHABHHAHABHHHHHHHBBBABBHHAHBBBHAAHHBHABHAHHHHHHAAABHBBHBHBBHHBBBABB

*002174_00089651 ABHBHHBHBHABBHHBBHHAHAHAAHAHBBHHABHHAAHHBAHBAHHBHBBAHHHAAHHBHBBAAHHHAAHAAHBHBHHHBHAHBAHHHHBHHAHBHBBAHBHAHBHHHHAHHHHHBHHHHHAHAHAHHHHABHBHABAAHAAB

*002829_00010749 ABHAHHABBHHHHHHAAHHAHHHHHBHBBBHHHBBAHHHAAAHBHBHBHHHHHHHAHAHAHHHBBHHHABHHBABHBHHABHHAHABHHHAHHHBBBABBHHAHBBBHAAHHBHABHAHHHHHHAAABHBBHBHBBHHBBBABB

*007214_00009319 ABBABABAHHBAHAHAAHHBAHBHHHAHHHHHHHBHHAHHHHHHHHHBAAHAHHAABHHHHHHHHAHHHHABBHBBBABHHBHHHBBHABAHAHHHHHBBAHAHHBBHHHBABBHBBHAHAHBABAHBBBHAHABBHHABHHHA

*011143_00000051 ABHBHHBHHAABHHHBHABAHAHAAHAHBABBAABHAHAHHAHHAHABHBBAHHHBAHBHBHHAAHHHAHHAAABHHHHHBHAHBAHHAHHAHHBBBHBHHHHAHBBHHHAHHHHHHHHAHHAHABHHHBHAHAAHHBAAHAAB

*000193_00236943 ABHBBHABHHHH-HAHHBAHBBAHBHHAHAAHAAAHHHAAABHAHHBHAHAHHHHHBHHHHAAHHHHAHHBHBHBHBBHHBAHHBAAHHBHHHBBBHBHHBHHHBBABHAABBHABHHHBBBBBHBHAHHBHHHHAHHBAABHH

*000299_00202728 BBBABHBHH-B-HA-AHA-HHHAHHHHHHHHAHAABHABB-HBAHHBHAHAHHABBHAABHHHHHABH-H-BBHHHABBABAAHBBABHBHAH-HAHBBHHABHHA-A-HHHBHAH-H--H-HH-BBHHHHHBAHAHBHHABH-

*000375_00113936 ABABBHHHAHB-AHBHHA-HBHHAA-AAHHBHHAHAHHBBAHBHHAAH-AHHBHABHBHHHAHHABHH-B-AHHHBHHBHAA-BHHHAA-HAA-HAHHHHBA-AH--BHBHBBAABHHHBBHHBBAHHBHHHB-HHH-BHHHB-

*000157_00103114 HBHHBHAHAAHAHHH-HHHHHHH-AHHABBBHHAHAH-HB-BHHHHAHHHHHBAABAHHHHABAABHHA-BH-HHBAHHHAABBAHAHAHABABHBHHBHBBAAH--BHHHABHAHAHAHB-HHBAAHB-HHH-BAB-BHAHHA

*001363_00145869 HAHHHAAH-HHAAH-HHHAHAHAHA-HAAHAHAAAHHAHAAHAAHHAHHAAAHHHHAAHHAHHHHAHB-HAHAAAHHAHAHA-HH-HHHAAHA-HAAAH-AA-AAAAHAHHAHHA-AHAHHHAAHAHAHHAHAAHAHAHAAHAH

*000822_00265746 HBAHHHBABHHAABHHBHHHAHHAHBBBHBAHBBHHHA-AHH-HABAHBBBHHHAABBABHBBAAHHH-AAAAHHH-AHHHH-HH-HHBBBHHBHHHBBHHBBA-HHHBHAHHHBBBHHHHBHAAHHAH-BBHHAHAHHAHAHA

*000132_00410994 BHBBHAAABBBAAABHHBHBBABHHHAHHAAAHHABHBHHHHHHHHBHHAHBBAHHHAHBAHHABHHA-AAHBAHHABBHAAAH-HHBBHAHBAHHBAAABHBBHABHHHHHHHAHHAAHHHHB-HAB-HHHBHH-HHHHHAHH

*000178_00456644 BHBHBAHHBHHHHHHHHHAHAHHAABHBAHAHBA-AHHHHHHBBAAAHAHHAAHHAHHHAHABABHHBBHHHAHABABHAH-ABAHHA-HAHHHHBBABABHHBHHAHHBBAHHB--BAHBHHAHHHABBHHHHBAHAAHHHBB

*000541_00225645 BHHAAHHAHBA-AABHBBHBHHHBHBAHHAAAABHHABBA-HHHBHBAHHHHBABHHAABAHHHBHAA-B-HBAAHABHHAAHBHHHBBHAHBAHBHAAHBHHHHABAHHHHHHHAHHHHHHHHHHABAHHHBABHH-AHHAHB

*001250_00178039 BHHHHAHABBB-AABHBBHBHABHH-AHHAAAHHABHBHHAHHHHHBHHAHBBAHHHAHBAHHA-HHA-AAHBAHH-BBHAAAHBHHBBHAHBAHBBAAABHBBAHHAHHHHHHHABHAHHHHHHHABHHHHBAAHHAAHHAHH

*000037_00800847 HBHHHHABH-BHHHBHHHHHHHBAHAHHH-HBHHHAHABHABBHAHHHHAABHHHBBHAAHHHHBHAB-BBABAHBAHHBHA-BHAAAHBABH-AAHHAABHAHHHBAHBBAHAHHAH-HBHBHAHAABHHHHABHHHAAAHHB

*001214_00055944 HAABHAHAABHB-HAHHHHBBHHHABABAAHHAHBHBAHBAAHBHBHHHHHHBHBHHAAHHABHABBABABHHBHA-HBAHHAHABBHHHHHHHHBHAHHABHHHAHHHBBHBHABBHAHHAHB-HBH-HHHBHBBH-AHBHH-

*003088_00002576 HABAHBHHHHHBBHHHHHHBHHAHHAHHABHHHHABHAAABHBAHHBHAHAHBHABHAAHHHHHBHABHH-BBHHAAHA-BHBAHBHBHBHAHBHHHBHHHH-AH--HHHHBBBABHHBAHAHHBBBBA-AHBABHBBAHHBHB

*000580_00112429 BHBHBAHHBBHBHHHHHHAHAHHAA-HAABABBHHAHHHHBBHBAAAHAHHAAAH-HHHHBABHBHHBHBHHAHABABHHHHABAHHHHAAHHHHBBABABHHBHH-HHBBAHHHBHBAHBHHAHHBABBHHHAAAHHAHHHBB

*006485_00005948 BHHAAAAHHHBHHHABHHHBABBAAHAHHHBBBHBAHBBHBBHHAHBBBHHHAHABHBHBBBHBHHHHBBHBAHHBBAAHA-HHBHBH-HHABHHBHAAHABHBAHHHHHBHHHBHABAHHHH-HBHAHHAHHHBBHHHHBHBB

*000490_00114245 BAHBAHABHHAHHHHHBHABABBHBHHHHHHAHBHABABHBHHAHHBHAHHHAAAHBABAAHHHHHBBAABAHHAH-BHHHAHBAHAH-BAHA-HHBBHAABHHBHBHHAHHHB-BABHHHBBAAHHHHHBHHBHHHHHHABAA

*000020_00184780 HHBBHHAABBHBHHHHBBAHHHAHHHHHAABHHHBHHHHBAAHAHBBHBHBHBHHBHHBBHHAAHBBBBBAABHAHABBHAA-HAHAHHHHHHAAHBHAHHABHAAHAABBHBHAAHHHHBHHHBHHHHBAHHAHHBHHBAHHB

*000024_00399170 BHHAHHBAHBHHAHHAHHHAHHAAAHAAAHHHBHHHHBBBAAHHAHHBHHBHHAABHBHAHAAAHHHAAHAHHHBBAHBBBHHH-HHAHHHAAHAHABHABHHHHHAHAHHBBBABHHHBBHHHBHAABBHBABHBHHHBHABB

*000258_00090553 BHHABBAHAHHAAHAAHHHHHHHHHAHBAHHHHHHABBHHHBHBHHAABHHHHAABBAAHHBAHHHAHHBBHBBBHAHBBHABBBBHHHBHBBBAAHHBAHHAHHB-ABBHHBAHBABBHBBBHAHHAHHHBHAHHHHAHHHHH

*000503_00246870 HHAABAHAABHBAHAHHHHBBAHHHBABAAAHHBBHBAHBAAHHHHHHHHHHBHBHHAAHHABBHBBHBHBHHBAHAHBAHHABABBHHHHHABHBHAHHAHHAHA-HHBHHBHABHHAHHAHBHHBHAHHHBHBHHBAHBHHB

*000596_00248736 HHBHBABBAHHHHAHAHHBHHAHAHAAHHHHHHAHBHAHBAA-AAHBHAHHHBHBBHHHHHBHHHABABBBBHHHHAHAHBAAHBHABBBHAHBHAHBBHHHBHHABAHHHHBHAHHHAHBAHHABHHHBHBBHHAHBHBABHB

*007910_00004575 BHHAAHHAHBAAAABHBBBBHHHBHBAHHHHAABHHABBHHBHHBHBHAHHHBABHHAABAHHHBHAAHHHHBAAHABHHAAHB-HHBBHABBABBHAAHBHHHHAHAHHHHHHHAHHAHHHHHHHAHHHHHBABABBAHHAHB

*000464_00433724 HABABAHHBHBHHHHBHH-HAHBAABHBHHHHHAHHHHHHHHBBAAHHHBBAAAHHBHHAHABABHHBHHHHAAHBABHAHHABHAHAHHAHAHHHAABAHHBBHHAABBHHBHHBHB-HBHBAHBHABBHHABHAHHAHHHBB

*001892_00008881 AHAHAHBHAHHAHAAHHHBBHHABAHBBBAHAHHAAHBHHHHHBBBHHAHAHHHBBAAAAAHHHHHABBHBAHHAHHAHAHHBHBHHHBHHHHAAAABHHAHAHHA-HHHBAAHHHBAHBHBAH-AAAHHHHBBHHBHAAHHHA

*003610_00023651 HAHABAHHBHBHHBHBHHHHAHBAHBHBHHHHHAHHH-HHHHBBAAHHHBBAABHHHHHAHABABHHBHHBHAAHBABHAHH-BAAHHHAAHAHAHAABHHHBBHHAAHBHHBHHAHBAHBAHAHBHABBHHABHAHHAAHHBB

*002020_00018186 ABBABABAHHBAHA-AAHHBAHBHHHAHHHHHHABHHAHHHHHHHHHHAAHHHHAABHHHHHHHHAHHAHABBHBB-ABHHBHHHBBHABAHAHHHHHBBAHAHHBBHBHBABBHBBHAAAHBABHHBBBHAH-BBHHABBHHH

*000036_00142794 AHHBHHBBAHBHHHAHBHBHBHBAABBBBHHBAAHHHHABHBHHAHHHAAAAHHAHBBAHBHAHBHBBBHAAHAAAAHHHBHHBHAHHHHHAAHHHBHHHHHHHHBHHBABHBHHBBHHAHHBBBBAAABHHHBHABHHHHAHH

*000073_00371648 HBHBHBHBAHBHBBBBHBHBHHBAAHAHHHHHHHHHHAHAABAHAHHHHHABHBBBHBHBHAAHHAABHHBHBABHHAHHBAHBHAHABBHHHHBAHBAHBBHHAHBHHHBAHAHBHABHHHHHHHABHHABHHHBHAABHAHB

*000091_00051396 HABABAHHBHBHHBHBHHHHAHBAHBHBHHHHHAHHHHHHHHBBAAHHHBBAAHHHBHHAHABABHHBHHBHAAHBABHAHHABAAHABHAHAHHHAABHHHBBHHAAHBHHBHHHHBAHBABAHBHABBHHABHAHHAHHHBB

*000146_00039399 BBHABBABAHHHAHAHHHHAHHHHHAHBAHHHHHHAHBHHHBHBHHAAHHAHHAABBAAAHBHHBBHHHBBHBHBBAHBBHABBBAHHHBHBBHAAHHBAHHAHHHBABBHHHAHHABBHBBBHAHHABAHHHAHBHAAHHHHH

*000228_00056537 BAHHHBHAHHBABAHBBHHAAHHHAAAHHAAHHBHAAHHBHBAHHHHBBAHAHBAHBHHBHHBHABAHBHHAHHBHAHABHAAHHHHHHBBHAAHHAAHHHBHAHBBBHBHHHHHAHABBABBAHBAHHBHBHAHAHHHAHHHB

*000602_00239360 BAAHHAHHHHBHHAAHAAABBHHHHHABHHHHBHBBAHHBBAHAHAHBHHBBHHHHHBHHHAHHAHAHBHBABHHBAAAHHHHBAAHBHBHBBAHHHAHABHHAHHAHHABHHHHHHAABABHBAABHBBHABAABHHHHHHBB

*000635_00357588 BBAHABHHBAHHHBHHAAHHHHBHHHBHAHHBHBAHBHHHHBHHHAAHHHHAHBHHABBABHABHHHAAHABBHHHHABHHHBHHBHHHBHBBAHAAHBBHAAHBHAHBBHABHAHHBBBHBAHAHBHHHBHHAHBBABBHHAB

*000648_00248133 BBHAHHBBAAHAHHBHBBBHABBHHBHBBABHBBHAABHBHBAAHAHHAAAHHAHBHHAHHAHHHAHABHHHHBBAHBABHHHABAHHAHHHBHAAHHHAAHABHBHHHAHHAAHHBHHHHHHAHHHAAHHAHAHHBHBHHBHA

*000654_00067438 BBBAHHBBHHHABHBAHBBBAHHHHBHBBABHBBHAABBHHBAAHAHHHAAHHAHBHHAHHHHHHHHABHHHHBHAHBABHBHAHHABAHHHHBHAHHHAAHHBAHHABAHHHAHHBHBHHHHAHHAAAHAABAAHBHBHABHH

*000695_00172920 AHHBHHBBAHBHHHAHBHBHBHBHABBBBHHBAAHHHHABHBHHHBHHAHAAHHAHBBAHBHAHBHBHBHAAHAAAAHBBBHHBHAHHHHHAAHHHBAHHHHHHHBHHHABHBHHBBAHAHHBBBBAAABHHHBHABHHHHAHH

*000855_00091215 AHHBHBBHHHHBBHAHAHHAHHBBBAHHHAABBBBBHHAHBBHHBHHHBABHBHBAHHHBBBBBABAHBHBAAHBBBBHBBHHHAHHHHAHHHAHAHHHAHBHBHAAHHHBAHHHAHABBBBHHHHBHBHHHAHHHHAHHHAHH

*000855_00106858 AHHBHBBHHHHBBHAHAHHAHHBBBAHHHAABBBBBHHAHBBHHBHHHBABHBHBAHHHBBBBBABAHBHBAAHBBBBHBBHHHAHHHHAHHHAHAHHHAHBHBHAAHHHBAHHHAHABBBBHHHHBHBHHHAHHHHAHHHAHH

*001142_00121662 HAABAHHHHAHBAHAHHBHHBAAHABAHHHAAHBHHHBHHHAHAHAHBHHHHABABHHHHABHHHHHBBAHHBHHAAAHAHHBBAHAHHBBBHAAHHABHBABABHHHHAHHAAHAHHHHAHHBAAAHABBHHAHBHHHHAHBB

*001193_00187380 HAHBAHBAAHHAHBHHHHHBBBBHBBHBHHHBBBBHAAHBAAAHHAAHHAHHBBHBBBHHBHHHAABHHABHAAAHBAHBHBHAHAHHBAAAHHHHHHBHBHHBHHHHHHAHAHHBBBHAHHBBAHHHHHHHBAHHAHBBHAHH

*001398_00165404 BBHABBAHAHHHAHAAHHHHHHHHHAHBAHHHHHHABBHHHBHBHHAABHHHHAABBAAAHBAHHHHHHBBHBBBHAHBBHABBBBHHHBHBBHAAHHBAHHAHHBBABBHHBAHBHBBHBBBHAHHAHAHBHAHHHAAHHHHH

*001499_00068621 AHBHHHAHBBBHHHHBHHAABHHAHAAHAHHHHHHBBAHBHAAAABHHAHBHHHHHHHBBHAHAHABBAHAHBAAHHBBHHABHAHHAAHHHHHHHAHAHBAHHAHHBABHHBAAAABHHHBHBHBAHHHAHBAHHBAAHHAAH

*001989_00199089 HBHHAAHHBBHHABHHABABBHHHHHBBAHBHHBHHAAHHHBAHHABHBHBBHBHHHBHAHHHAHAHAHABHAHHHHAHAHHHHBAHHHHHABAHHAHBBHHAHABHHHBBABHAAHBABBBHBHHBHHBAHHABBHHHBHAAB

*002055_00069198 BBHABBABAHBHAHAHHHHAHHHAHHHBAAHHHHHAHHHHHBHHAHAHHHABBAHBBAAAHBHHBHHHHBBHBABBHHHBHABBBAHHHBHBHHAAHHBABAAHHHBAHBHAAABHAHBHBHHHAHAABHHHHAHBHAAAHHHH

*002205_00025761 AHBAHAHHHBHHHABAAHABAHHHHHAHHHAHHBHHBHBHHBHHHAHBAHAAHHAABBAHHHBBBHHBHBAHBHHBBHBHHHHHHHHHABAHHAAHHHBAAHABHBBHHHBAHBABHHAAAHBAHAHBBBHAHAHBHHABHHHA

*002245_00113261 HBHBHAHBABBHHHAHAAHBBHAABAHBHBHHHHHBHHHBHAHBAHHBBBBBHHBHHHHAHAABHHHHHBBABHABHHHBHBHBHAHHABBBBAHBHAHABBHHHHAAHABHHHHHHAABHBHBAABHBAHHHAHHHHHHHHBH

*002356_00124231 AHHBAHBBAHHAHBAABHBHBHBHABBBBHABAAHHHHABHBBHHHHHAAAAHHHHHBAHBAAABHBBHHAAHAAHAHHHBHHBHAHHBHHAABHHBHHHHHHHHHHHBHBHBHHBBAHAHHHBBBAAHBHHHBHAHHHBHAHH

*003845_00002815 BAHAHHAAAHBABHHBBHHHHHHHHABAHAAHBHHBHHHBBHABHBBAAAHHHHAABHHBHHHHHHBHHAHAAHHHHBHAHAHBHHBHAHBBBHHHABHHBBAHBHABABABHABHHAHHHHBHHAHAHHBBBBAHHHHABAHB

*004196_00025515 HBHBHBHBAHBHBBBBHBHBHHBAAHAHHHHHHHHHHAHAABHHAHHHHHABHBBBHBHBHAAHHAABHHBHBABHHAHHBAHBBAHABBHHHHBAHBAHBBHHAHHAHHBAHAHBHABHHHHHHHABHHABHAHBHAHBHAHB

*005668_00017677 AHHBHHABHHHABBAAHBBAHHHBHBBHHBABHABHHHAHBBBHBHHHAAAABHHHHHHHBAAHHHBHAAHAHABBHHHHHAHBHAHHHBHAAHAHBHBBHHHHAHAHBBHHBHABAAHAHHHHHBAHBBAHAHHBHBAHHAHH

*006988_00011054 AHHBABBHAAAAHAHHBHHBHHHBHABBBAHAHHAHBHHHHHBBBHHHAHAHBHHBHHAAAAHHHHBBBBBHHHAHHHBAHABHHHHAHHHHAAAAABAHABABBHHHBHHHHHHHBABBHHHHHAHAHHBABBAHBBHHHHHA

*000023_00331417 ABAAHHBHAHBHABHHHHHHBHAAHBAHAHBHHHAAHHBHAHBHAHHBBHHHHHABHBBHHAHAHBHHABHHAHBB-HBBHHHBAHHAAHBAAHAAAHAHBABAHHAHHHHBBAABBHHBBHHBHHAABHHHBHHBHHHHHABH

*000193_00157554 ABHBBHABHHHHHHAHHBAHBBAHBHHAHAAHAAAHHHAAABHAHHBHAHAHHHHHBHHHHAAHHHAAHHBBBHBHHBHHBAHHHAAHHBHHHBBBHBHHBHHHBBABHAHBBHABAHHBBBBBHBHAHHBHHHHAH-BAABHH

*000840_00013632 ABBBHBHABHABAHHAHAHHBHHBHBBHAHAHBBHHHAHHBHHBBAHHHBHAHBHHHHHHHAHHAHBBBHHHHHBHBAAHHHHBAHHBBBAHHABHABHHHAHHAA-ABHBHHBHAHABHHAHHHHHHHBABAABHBBHBHAAB

*000866_00060359 ABHBHHBABHHBHBHHBHHAHHHAAHABBBHHBBHHAAHHBHABABHBBBBAHHHAAHHBHBBAAHHHAAHAAHHHBAHHBHAH-AHBHHBHHAHBHBHAHBHAHBHHBHAHHHHBBHHHHBAHAHHHHHHBBHBBAHHAHAAH

*001860_00052053 HH-HH-BHHAH-BH-HH-BHHHAHAAHHABHHH-ABHAAH--BAHHBHAHAHAAA-HAAHHHHH-HAHHH-BBHHH-HHAB-BA-BHBA-AAH--HHBHAHH-AHA-HBH-BBHAB-HBHHA-H--BB--HHB-B-BBH-ABA-

*000147_00350754 AHHAHHAHHHAA-BAH--BHAHBH-BAHBAHHAAHBAH-H-HBAHHHBBAHHBBHHABAHHBBH-BHB-H-AHBHH-H-BBH-H-HHAHHHAH-AHAABHHAHHHA-HBHHABBB-AAH-HHAABHHBB-HABHH-HBBHHAHH

*003963_00000342 AHHHBHAHAAH-BHHHHHHHHHH-A-HABBBHHAHAHBHBAH-HHHAHHHHHBAA-HHHBHABAABHHAB-BAHAB-HHHAABBA-AHAHHBA-HBHHBHBBAHHH-BAB-ABHAH-H-HB-BHBAAHBHHHHAHAB-BHAHHH

*000370_00278377 AHAAHHBHHHH-ABHABHHHBHAAHAAHAHBHH-HAHB-HAHHHAHHB-HBHHHABHBBHHAHAHBHB-HHHHHBB-HBBHHHBAHHAAHHAABAAAHAABA-HAH-HHHHBBAABB-HBBH-BHHAABAHHHHHBH-HBHABH

*000510_00299308 HAH-ABHHHBH--HBAAAHAHHHHHHABHHHHAHHHBBBH-BHBHHAABBAAHBH-ABBHHAAHAAHB-HABHHHHHAHBA-BHAHHHHHHBBABAAAHBHHAAAHAHBHHAHHAHHHBHAAAH-AHH-HAHHAH-BABHHBH-

*000315_00208503 AHHHHHHAAHHBHHH-BHBBHAH-BHBAAAHHAHAHBAAHBHBHAHHHAHHHBBBHHHHHHBAHHHBA-AHHHAHA-BAA-BBHHBHABHHHH-BBHHBHHBBHAAHBBHHBAHAB-HHABAHHB-BBABAABAHHH-HHHHHA

*001223_00092740 HHHHAHAHHHABHBAABAHBHHHHHHHBHAHAHABHHABHBHAHHAHHAAABAAHHBHBAAHHHHABBBABAHBA--HHAHAAHHAA--BAHHAHHBBHHABHHHHHBBAAHHHHAAHHAHHBHHBABHBHHHBHBHHAHAHHA

*000736_00421639 AHHBB-ABBHHHHH-HHBAHBBAHBHHAHAAHAAAHHHAHABHAAHBHAHAHHHHHBHHHHAAHHHAAHHBHBHBH-BHHBBHBHAHAHBHHHBBBHBHHBHHHBB-BHAHBBHABAHHBBBHBABHAHH-HHHHAHHBAABHA

*000332_00218203 HHHAHAAHBHHHHAHHABHHHAAHHHAHAAHAHABBHBBAHHHBHHABHHBHBHHHBHHAAHBHHBBA-HHHAHBHBBAHHHBBHAHAAHAHHABAHABHHBHHHBBABAHHBHBHHHAAHBBHAHHBHBHHHHB-BABBHHAB

*000803_00319826 HHHHAHAHHHA-BBHABAHBHHHHHAHBHAHAHABHHABHBHAHHAHHAAABAAHHBBBAAHHHHABBHABAHBAHAHHHHAHHHAABBBAHHAHHBBHHABHHHHHBBAAHHHHH-BHAHHBHHBABHBBHHBHBHHAHAHHA

*003648_00025303 BHHHAHAHHHBBBHAHHBBBHHHHHBHHHBHABHBHBABHAHHHHHBHAHBABAHHHBBAAHBBHBHBBHAAHHBHAHAHHHHA-ABHAAAAHHBHBAHHBHBABH-HHHHHBBABHHHHBHHHAHHAABBBHHHBBHBHBHAB

*005460_00000183 HHBHHBHHHHAHBHBHBHHHHBHHAAHAHBHHABHBBHAAHHBHHABHHAAHBHAHAHAAHBBHHHAHAHBHHBHAAAHHBHBHHBHBHHBAHBHHHBHHHBHAAAHBAHHBABABHH-BHAAHBHHBAAAHBAHHBBH-HBHA

*008584_00008844 HHAAABHHHHHHBHHAAAHAHHHHHHHBHAHHHHHHBBBBBBHHHAAHBHAAHBHHABBHHHAHHAHBAHABBHHB-AHBABBH-HHHHBHBBABAAAHBHHAAAHAHBHHAHHHHABBHAAABAAHHHHAHHHHBBABHHBHH

*000464_00402361 HABABAHHBHBHHHHBHHHHAHBAHBHBHHHHHAHHHHHH-HBBAHHHHBBAAHHHBHHAHABABHHBHHBHAAHBABHAHBABAAHAHHAHAAHHAABAHHBBHH-AHBHHBHHHHBAHBHBA-BHABBHHABHAHHABHHBB

*011087_00000602 HAHBAAHHAAAAHHHAHHHHBAHBBBHHHABBAHHBAHHHAHBABBHAHHHHBAABHHBABHHHHAAH-HABAHAH-HAHHAAHHHBHAAAAABHHBAHAAHBHHH-HHBHHHHAAHHAHHAHBHBAHHBHHHHAHHHHABBAB

*000044_00224406 HBHBABHAAHHHABHBHBHBHHHHHBABBAAABBHAHAHAAHHHHHHHHHHBHHBBHBBHAHABHHAA-BBHBABHHAHHBHHBAAHABAH-HHBAAHHBHBHHA-BHHHBABABHHHBHHHHHAHAHHBHBHHAHHHABHAHB

*001169_00160124 ABHBAHAHHHHHBHAHHBAHHBHHBBHABAAHAAAHH-AAABHAHHBHAHAHHHHHBHHHHA-BHHHAHABHBHBH-BHHBHHHHHAHHBHBHBBBHBHHBHHHBB-BHAABBHABAHHBHBBBHBHAHHBHHHHABBBAABHH

*000020_01170664 HHBBHHAABBHHHHHHBBAHHHAHHHHHAABHHHBHHHHBAAHAHBBHBHBHBHHBHHBBHHAAHBBBBBAABHAHABBHAABHAHAHHHHHHAAHBHAHHABHAAHAABHHBHAAAHHHBHHHBHHHHBAHHAHABHHBAHHB

*000064_00660591 HHHBABBHAHAAHAHHHHHBHAHBHABHHAHABHHHBHHHBHBHBHHHAHAAHBHBHHHAAHHBHABBBBBAHHAHAABABHBHHHBAHHAHHAAAHHAHHHABBHAHBAHHAHHABHBBHHHHHAHHHBBABHAHBBHHBHHA

*000067_00605820 HHBHHBHHHHAHBHHHBHHBHBAHAAHHABHHHHHBBAAAHHBHHABHAAAHHHABAAAHHHBHHHAHAHBHBBHAAAHHBHBHHBHBHHBAHBHHHBHHHBHAAABBAHHBABABHHBHHAAHBHHBAAAHBAHHBBHHHBHH

*000079_00778622 HHABHAHBAAAAHHAAHHBBBAHHHBAHHABBAHHHAHHHAHBAHBHAAHHHBAAHHHHABBHHHAHHAHAHAHAHAHAHHAAHBHHHAAAAABHAHABAABBHHHBHHBHHHHAAHHAHHAHBHBAHHHHHHHAAHHHHHBAB

*000275_00153075 BHHAHHBHHBHHAHAAAHAAHHAAABAHAHHHBBHHHBBBAAHHAHHBHHBHHAABHBHAHAAHHHHAHHAHHHBBAHBHBHHHAHHAHHHAAHAHABHABHHBHHAHAAHBBBABHHHBBHHHBHAAHBHBABHHHHHBHABB

*000310_00069000 HHABHAHAAHHBAHAAHBHBBHHHHBAHAAHHAHBABHHBAAAHHBHHHHHHHHBHHAHHHABHABBABHBHHBHAAHHAAHAHABHHHHHHAAABBAHBHBHHAAHHHBBHBHABHHAHHAHBBHBHAHABBHHAHBHHHBHH

*000319_00045392 HHBHABHABHABAHHAHAHHHHHBHHABAHHHBBHHHHHHBHHBBAHBHBHAHBHHBHHHHAHHAHBBHHHHHHHHBAAHHHHBABBBBAAHHABHABHHBAHAAAHABHBBHHHAHABHHABHHHHBHBABHHBHBBHBBAAB

*000434_00275339 HHHHABAHAHHBAHHBHHHHHHBHAHHABHBHHABHHAHABHAHHAHHHAHAAABHBHHHHBBHHABBBHBHAHAHAHHBAHHHHAHBHBHHBAHAHBHAHBAHHHHBBAABAAHHABBAAABHHABHHHHHAHHBHAAAHHBA

*000455_00006569 HHAAABHHHHHBBHHAAAHAHHHHHHHBAAHHHHHHBBBBBBHHHAAHBHAAHBHHABBHHHAHHAHBAHABBHHBHAHBABBHHHHHHBHBBABAAAHBHHAAAHAHBHHAHHHHHBBHAAAHAAHHBHAHHHHBBABHHBHH

*000518_00170542 HHHBABBHAHAAHAHHHHHBBAHBBAHHHAHABAHHBHHHBHBHBHHHAHAAHBHBHHHAAHHHBAHBBBBAHHAHAABABHBHHHBAHHAHHAAAHHAHHHABBHABHAHHAHHABHBBHHHBHAHHHBBABHAHBHHHHHHA

*000542_00059547 HHHHHHAABHBHBHAHHBBBHHHHABHHHBHABHBHHHBHBHHAHHBHHHBABAHHHBBAAABBABHHBHAAHHBAABAHBHHHHABHAAHAHHBHBAHHBHBABABHHBHHBBABHHHHHHHHHHHAABHBHHHHBHBHBAAB

*000542_00162149 HHHHHHHABHBHBHAHHBBBHHHHABHHHBHABHBHHHBHBHHAHHBHHHBABAHHHBBAAABBABHHBHAAHHBAABAHBHHHHABHAAHAHHBHBAHHBHBABABHHBHHBBABHHHHHHHHHHHAABBBHHHHBHBHBAAB

*000688_00261291 HHHHAAABHHABHBHHBAHHHHHHHHHHHAHAHABHHABHBHAHHHHHAAABAABHBBBAABHHHABBHABHHBAHAHHHHAAHHAHBBBAHHAHABBHAHBHHHHHBBAHHAHHHABAAAABHHHBBHHHHABHBHHAHHHBA

*000912_00217577 HHHHAAABHHABHBHHBAHHHHHHHHHHHAHAHABHHABHBHAHHHHHAAABAABHBBBAABHHHABBHABHHBAHAHHHHAAHHHHBBBAHHAHABBHAHBHHHHHBBAAHAHHHABAAAABHHHBBHHHHABHBHHAHHHBA

*001739_00222225 HHHHAAAHHHABHBHABAHBHHHHHHHHHAHAHABHHABHBHAHHAHHAAABAABHBBBAAHHHHABBHABHHBAHAHHAHAHHHAABBBAHHAHHBBHHHBHHHHHBBAAHHHHHABAAHABHHHBBHBHAHBHBHHAHAHHA

*001965_00148091 HHHHAHAHHHABHBHABAHBHHHHHAHBHAHAHABHHABHBHAHHAHHAAABAAHHBBBAAHHHHABBBABAHBAHAHHAHAHHHAABBBAHHAHHBBHHABHHHHHBBAAHHHHHAHHAHHBHHBABHBHHHBHBHHAHAHHA

*002119_00030345 HHHHAAABHHABHBHABAHHHHHHHHHHHAHAHABHHABHBHAHHHHHAAABAABHBBBAAHHHHABBHABHHBAHAHHHHAAHHAHBBBAHHAHABBHAHBHHHHHBBAAHAHHHABAAAABHHHBBHHHAHBHBHHAHHHHA

*002171_00046925 HHHHAHAHHHABBBHABAHBHHHHHAHBHAHAHABHHABHBHAHHAHHAAABAAHHBBBAAHHHHABBHABAHBAHAHHHHAHHHAABBBAHHAHHBBHHABHHHHHBBAHHHHHHABHAHHBHHBABHBHHHBHBHAAHAHHA

*002552_00026881 HHBHHBHHHHAHBHBHBHHBHHAHAAHHABHHHHHBBAAAHHBHHABHAAAHHHABAAAHHHBHHHAHAHBHBBHAAAHHBHBHHBHBHHBAHBHHHBHHHBHAAABBAHHBABABHHBHHAAHBHHBAAAHBAHHBBHHHBHH

*004613_00021772 BHHAHHBHHBHHAHAAAHAAHHAAABAHHHHHBBHHHBBBAHAHAHHBHHBHHAAHHBHAHAAHHAHAHHABHHBBAHBHBHHHAHHHHHHAAHAHABHABBHBHHAHAAHHBBABBHHBBHHHBHAABBHBABHHHHHBHHBB

*005334_00003366 BHHBHAAABBBHAABHABABBABHHHAHHHAAHHABBBHHHHHHHHBHHAHBBAHHHAHBAHHABHAABAAHBAHHABBHHAAHBHHBBHAHBHHHBHAAHHBBHHHHHHHHAHAHAAAHHHHBBHABHHHABHHHAAHHHAHH

*006619_00006399 HHHHAHAHHHABHBHABAHBHHHHHAHBHAHAHABHHABHBHAHHAHHAAABAAHHBBBAAHHHHABBHABAHBAHAHHAHAHHHAABBBAHHAHHBBHHABHHHHHBBAAHHHHHABHAHHBHHBABHBHHHBHBHHAHAHHA

*000030_00532901 HAHBAHBAAHHHHHHHHHHBBHBABHABHAHAHBBHAAHHBHAAAAHAHHHBHBHHBHBHAHBHAABHHHBAAHBHBAHAH-HABHHABAHAHBHHAHHBHAHHHHHHHHAHHBHBBHHABABBABHAHHHABHAABHBHHAHH

*000039_00080448 AHBHHBHHHHABBHBHBHHBHHAHA-HHABHHHHABBAAAHHBHHHBHAAAHHHABAAAHHHHHHHAHAHBHBHHAAHHHBHBAHBHBHHHAHBHHHBHHHBHAAABHHBHBHBABHHBHHAAHBBBBAAAHBABHBBHAHBHH

*000098_00635102 AHHHHHHAAHBHHHHHBBBBHHHABBBHAHHHABAHBAAHBABHAHHBAHHHBBBHHHHHHBAHHABH-AHHHAHAAHAAABBHHBHABBHHHBHBAHBHHBHHAAHBBHHHHHABHHBABAHHBABHAHAAHAAHHAHHHHHA

*000198_00400512 BBHABBAHAHHHAHAHAHAHHHHHHAHBAHHHHHHAHBHHHBHBHHAAHHAHHAABBAAAHBHHHBHH-BBHBHBBAHBBHABBBHHHHBHBBHAAHHBAHHAHHHBABBHHBAHBAHBHBBBHAHHABAHBHAHBHAAHHHHH

*000213_00348635 HBHAHBHHHAHHHHHAHHBAABHBBHBHHBABHABHAAHABHBHBHAHAAHHHHAHAHBHBABHHAHHAABHHAHH-HBHHABBHAHHHBHAAAAHAHHHHAHHHHAHBAHABHAHHBBHBHAAHBHBHBHBABHBHBAHHHBH

*000236_00264729 HBHHHBHAHHAHHBHAHBBHABHBBHHHBHHBAAHHHHHHHHBABAABAABBBBHAAHHHBBBHHHHBAAHHBHAB-HHHHABBHAHABBHAAHHHAABHAAHHHAAHBHHAHHHHHHBBHHAAAHBBBHHHBHHBHBHHHABH

*000497_00494736 BBHBBHHHHABAHAHBHBAAAHABAABHHBBHHBABHHAHHHHBBHBAAABHAHHAHAHHHHHBHAHHABHAHHHH-HBABHBHHAHHHBAHBBHAHHAHBHABAHAHABHHAHHHHHHHBAHAAHBBHHABHAHHHHAAHBHB

*000901_00266636 HBABHHBABBHHHBHHBHHHHHHAHBHBBBAHBBHHAAHABHABABABHBBHHHAAHBABHBBAAHHHHAHAAHHHBAHHHHAHHAHAHHBHHBHHHBBAHBBHHHHHBHAHHHHBBH-HBBHHAHHAHBHBBHBBAHHAHAAH

*001321_00051948 AHHBAHBBAHBHHHAHBHBHBHBHABBBBHHBAAHHHHABHBHHHBHHAHAAHHAHBBAHBHAHBHBHBHAAHAAAAHHHBHHBHAHHHHHAAHHHBAHHHHHHHBAHBABHBHHBBHHAHHBBBBAAABHHHBHAB-HHHAHH

*001838_00091672 AHAHAHBHA-HAHAAHHHBBHHABABBBBAHAHAAAHBHHHHHBBBHHAHAHHHHBAAAAAHHHHHABBHBAHHAHHAHAHHBHHHHHBHHHHAAAABHHAHAHHAHHHHBAAHHHBAHBABAHHAAAHHHHBBHHBHAAHHHA

*003068_00048406 AHHBHABBAAHHHAAAHHBHBAHAHHAHHABHHHHHHAHHAABAAHHHAHHHBABBHHHAHBHBHABHHHAHAHAHAHAHHAAHBHAHAHHAABBAHABHHBBHHH-AHBHHHHAAHHAHBAHBHBAHHHHBBHAAHBHHABHB

*004891_00008015 AHHBHBBHHHHBBHAHAHHAHHBBBAHHHAABBBBBHHAHBBHHBHHHBABHBHBAHHHBBBBBABAH-HBAAHBBBBHBBHHHAHHHHAHHHAHAHHHAHBHBHAAHHHBAHHHABABBBBHHHHBHBHHHAHHHHAHHHAHH

*007469_00010082 HBHHHBHHAHAABHBHBHBHHBHHAABHHHHBAHHBBHAAHBHHHABAHAAHBHAHAHHABBBHHHAH-HBHHBHAAAHHBHBHHBHBHHBAABHHHHHHHHHAAAHBHHHBABABHHBAHHHHBHHBAAAHBHABBBHBHBHA

*000666_00373570 ABHAHHABBHHHHHHAAHHAHHHHHBHBBBHHHBBAHHHAAAHBHBHBBHHHHHHAHAHAHHHBBHHAABHHBABH-HHABHHAHABHHHHHHHBBBABBHHAHBBBHAAHHBHABAHHHHHHHAAABHBBHBHBBH-BBBABB

*002020_00133311 ABBABABAHHB--AAAAHHBAHBHHAAHHHBHHHBHHAHHHAHHHHHHAAHHHHAHBHHHHHHHHAHHHHABBHBBBABHHBHHHBBHABAHAHAHHHBBAHAHHBBHHHBABBHBHHAAAHBABHHBBBHAHABBHHABBHHH

*000422_00225038 ABHAHHABBHHHHHHAAHHAHHHHHBHBBBHHHBBAHHHAAAHBHBHBHHHHHHHAHAHAHHHBBHHAABHHBABHBHHABHHAAABHHHHHHHBBBABBHHAHBBBHAAHHBHABHAHHHHHHAAABHBBHBHBBHHBBBABB

*000459_00403854 ABHBHHBHBHABHHHBBHHAHAHAAHAHBBBHABHHAAHHBAHBAHHBHBBAHHHAAHHBHBBAAHHHAAHAAHBHBHHHBHAHBAHHHHBAHAHBHBBAHBHAHBHHHHAHHHHHHHHHHHAHAHAHHBHABHBHABAAHAAB

*000540_00053076 AAHBHABBAHHHHAAAHHBHBAHAHHAHHABHHHHHHAHBAABAAHBHAHHABABBHHHAHBHBHABHHHHHHHAHAHHHHAAHBHABAHHAABHAHABHHBBHHHBAHBHHHHAAHHAHBAHBHBAHHHHBBHAAHBHHABHB

*000624_00067530 ABHBABHAAHHHABHHHBBBHHHHHHABHAAHBBHAHAHAAHHHHHHAHHHBHHBBHBBHHHABHHAHHBBHBABHHHHHBHHBAAHABAHBHBBHAHHBHBHHHBBBHBBABABAHABHBHHHHHAHHBHBHBAHHHABHAHB

*000624_00205777 ABHBABHAAHHAABHHHBBBHHHHHHABHAAHBBHAHAHAAHHHHHHAHHHBHHBBHBBHHHABHHAHHBBHBABHHHHHBHHBAAHABAHBHBBHAHHBHBHHHBBBHBBABABHHABHBHHHHHAHHBHBHBAHHHABHAHB

*000789_00241085 ABHAHHAHBHHHHAHAAHHHHHAHBHABBABHHHBAHHBAAAHBHBHHAHHBHHHHHAHAHHHABBBHAHHAAHBHBHHAHABBHHBHHHAHBABHHHBBABAHHBBAHABHBHBBAHHAHBHHAAABHBHHBHHHHHBBBHHB

*000954_00114188 ABHAHHAHBHHHHAHAAHHHAAAHBHAHHABHHHBBHHBAHAHBHHHHAHHHHHBHHAHAHHHABBBHHHHHAHBHBBHAHABBAHBHAHAHBABHHHBBABHHHBBAHABHBHBBAHHAHBHAAAAHHBHHHBHBHABBBHHB

*000986_00171288 ABHAHHABBHHHAHHAAHHAHHHHHBHBBBHHHBBAHHHAAAHBHBHBHHHHHHHAHAHAHHHBBHHAABHHBABHBHHABHHAHABHHHHHHHBBBABBHHAHBBBHAAHHBHABAAHHHHHHAAABHBBHBHBHHHBBBABB

*001617_00003121 ABBABABABHHBHAAHAHHBABBHHHAHHHHBHHBHABHHHAHBHHHHAABBAAAHHHHBBHHBAHHHAHHBBHBBBAHHHHHHABBBABHHAAHHHBHBHHHAHBBHHHBABBHHHBAAAHHABHHHHHHAHHBHHHHBBBHB

*003429_00061283 ABHAHHABBHHHHHHAAHHAHHHHHBHBBBHHHBBAHHHAAAHBHBHBHHHHHHHAHAHAHHHBBHHAABHHBABHBHHABHHAHABHHHHHHHBBBABBHHAHBBBHAAHHBHABAHAHHHHHAAABHBBHBHBBHHBBBABB

*004560_00044690 ABHAHHABBHHHAHHAAHHAHHHHHBHBBBHHHBBAHHHAAAHBHBHBHHHHHHHAHAHAHHHBBHHAABHHBABHBHHABHHAHABHHHHHHHBBBABBHHAHBBBHAAHHBHABHAHHHHHHAAABHBBHBHBBHHBBBABB

*000025_00023594 -BHHHH-H-HH--H-BBAHHHHAB-BBHABHHH-BBHHAABH-HBHBBA-HHHBHAHBHBHHHBHHHH---HAHH--HAHH----BHA-AH-A-H-A-BBAB-HH--BAAHHABAHAAAAH-BHBAHAABHAHHBA--H-----

*002183_00134127 B-HHH-AHHAAAHHHABHBAABHBBHBHHBAHBAHHHAAA-H-HBAAHAAHBHHHHAHBHHABAHAHHAAHABAAHHHHHBABB-AHHHBHAAHAHAHHHHAHHHBAAHAHAHHH--HBBBHAABHHB--HBHHBBH-AHHHBH

*000139_00007581 ABBABABABHH-HAHAAHHBABBHB-AHHHHHHH-HAHHHHAHHHHHHAAHBAAA-HHHBHHH-AHHHAHABBHBBBAHBHA-HABBHABHHA-HHHBBBAHHHHBBHHHBABBBB-HAAAHHA-HHHB-HAHHBHH-HBBBHH

*000217_00055522 A-BBBHHAH-HB-BBHHAHHHHAHHHHHBBHHHHHBHAABHBHBHABB-BHHHBHABHHHHHABBHHHHBBHBBAHAABA-BBBHAH-AHBHBABBHHHABB-HHA-HBHBHHHHAAABHHHBHBHBH-AHHHHBHBAHBHABH

*000855_00106944 AHHBHBBHAHH-BHAHAHHAHHBBB-HHHAABBBBBHHAHBBHHBHHHBABHBHBAHHHBBBBBABABBHBAAHBB-BHBBHHH-HHHHAHHHAHAHHHAHBHBHA-HHHBAHHHABHBBBBHHAHBHBHHHAHHHHAHHHAH-

*003384_00047899 HBHHHBABHAHHHHHAHBBAABHBBHBHHBABHABHAAHAHHHHBHAHAAHHHHHAAHBHBABHHAH--AHHHAHHBHHHHABBHAHH-BHAA-AHABHHHAHHHAAHBAHABHABAHBHBHAAHBHBB-HBABHBH-AHHHHH

*000076_00031394 AHHHBBHHHAHB-HHBHHHAABHHA-AHBHBAAHHHHHAHABHBHAHAABHHAABBAAAHAHBHAHAHBBA-HAHABBBHH-HHHBHHHHHAHHBBHHHBBHAAHAAHHHBHHABAHHBHAHAAHAAAAHHHBAHAHBHHHBHH

*000479_00298143 BBHHHHABABBH-HHHHHHAHHHAHHHBAABHHBHAH-HHBB-HAHAHHHABBAHBBHAAHHHHBHHBHBBHBAHBHHBBHAHBHAAAHBHBH-AAHBHABHAHHHBAHBHAAABHABHHBHBHAHAABHHHHABBHHAAAHHH

*002370_00020959 ABHAHHHBBHHHHHHAAHHAHAHBH-HBBBHHHBBAHHHAAAHBHBHBHHHHHHH-HAHAHHHBBHH--BHHBABHBHHABBHAHABHHHAHH-BBBABBHHAHBBBHAAHHBHABHHAHHHHHAAABHBBHBHBBHHBBBABB

*000096_00334248 HHHAAHBBHHHHBHAHHBABHHHHHBAHAHHAHHBAAAHHHHABHHBHBHABHBAAHHBHAHBBAHHAABBABHHBBHAHHBHABHH-AAHHHAHBHHHAAABAHHHAHHAAHHHABHHABBHHAHHABAHHHHHBBBBHHAHB

*000333_00027166 BHBHAABHBHAAHBBHHBHHHHBBHHBHHBBBHABBHAAAHHHHBHAHHAHHAHHAHBABBHHAHBAHHHHHBHAABHHAAHHAABBAHABBH-ABAHHHBAHHHBBAAHHAHBAHBHAHHHHHBBHAHHHABAHAHHHHBHHH

*000771_00178976 HHHHAHAHHHABHBAABAHBHHHHHAHBHAHAHABHHABHBHAHHAHHAAABAAHHBBBAAHHHHABBHABAHBAHAHHAHAHHHAABBBAHHAHHBBHHABHHHH-BBAHHHHHHABHAHHBHHBABHBHHHBHBHHAHAHHA

*001071_00026334 HHHAHHHHHAHHAHHBAHAHHHABBBBHAAHAAHBBHHHHHHABBHHHBHHAHBAABHBBAHBBHHBHHAABHBABHHHHBBBHHAHHABHAHHAHHAHHHHHHAHAHHAHBBAAHHHHABABHAHHB-BAAHABABABBHHHH

*001594_00204379 HHABHAHAAHHBAHAAHBHBBHHBHBAHAAHHAHBABHHBAAHHHBHHHHHHHHBHHAHHHABHABBABHBHHBHAAHHAAHAHABHHHHHHAAABBAHBHBHHAAHHHBBHBHABHA-HHAHBHHBHAHABBHHHHBAHHBHA

*007343_00001843 HHBAHAAHHHA-AAHHABAHHHABHBAHAAHAHHBBBABAHBHHHHHBHHBABBAHBABHAHBHHHBHHABHHBBBBBHHHHBBHAHAAHHHHHHHHHBHHHHHHHBHBAHBBHAHHHAABHBHAHHBBBHHAABABAHHHHAH

*011802_00001592 HHBBHHAABBHHHHHHBBAHHHAHHHHHAABHHHBHHHHBAAHAHBBHBHBHBHHBHHBBHHAAHBBBBBAABHAHABBHAABHHHAHHHHHHAAHBHAHHABHAABAABBHBHAAAHHHBHHHBHHHHBAHHAHHB-HBAHHB

*000427_00462000 HBHHAABHBBHHABHHABABBHHBHHBBAHBHHBHHAAHHBBAHHABHHHBBHBHBHBHAHHHAHAHA-ABHBHHHHAHAHHHHBAHHHHHABAHHAHHBHHAHHB-HHBHABHAAHBABBBHBAHBHHBHHHABBHHHBHAAB

*000071_00001350 AHBAHABAHBBAHABAAHABAHHHHHAHHHHHHHHHBHBHHBHHHHHBAAAAHHAABHHHHHHHBAHHAHABBHBBBABHHBHHHHHHABAHHHHHHHBBAHAHABBHHHBABBHBBHAHAHBABAHBBBHAHABBHHAHHHHA

*000112_00615160 AHHHBBBHHAHHBHHBHHHAABHBHBABBHBAAHHHHHAHHBHBBAHAABHHAABBAAAHAHBHHHAHHBAHHHHAHBHHHBHBHBHHHHHAHHBBHHHBBHAABAAHHHBHHABABHBHAHAAHAHAAHBHBAHHHBBHHBHH

*000177_00553664 HAABAHHHHAHBAHAHHBABBAAAHHHHBHAAHBAHHHHBHHAHAAHBHAHBABABHHHHABHAHHAHHHBHBAHHHABAHHBBHBHHHHBBHHABHHBABABABHHBBAHHAAHAHHHHABHBHHAHHBBAHAHHAHHHHHHB

*000292_00509720 BBAHBHBHHHBAHAHBHBAAABHBAABHHHBHABABHHAAHHHBBHBAAABHBAHAHAHHHBHBHHHHHHHAHHHHAHBHBHBHHAHHHBAHBBAAHHAHHHABAHAHABHHHAHHBHHHHAHAAHBBHHABHAAHHHAAHHBB

*000470_00198465 AHHHAHBBHHHHBHAHHHABHHBHHHAHAAHAHHBAAAHHBHAAHHBHBHABHBAABHBHAHBBAHBAAHBABHHHHHAAHBHABHHAAAHHHABBAHHAHAHAAHHAHHAAHBHHBHHABHBHHHHAHAAHHHHHBABHHAHB

*000553_00012112 BAAHHAHHHHBHHAAHAAHBBHHHHHABHHHHBHBBAHHBHAHAHAHBHHBBHHHHHBHHHAAHAHAHBHBABHHBAAABHBHBAAHBABHBBAHHHAHABHHAHHAHHABHHHHHHAABHBHBAABHBBHABAABHHHHHHBB

*000656_00264910 HAHBAHBAAHHAHBHHHHHBBBBABBHBHAHBBBBHAAHHAHAHAAAAHAHHBBHBBHBHBAHHAABBHABHAAHHBAHHHBHAHAHHBAAAHHHHHHHHHAHHHHHHHHAHAHHBBBHAHABBAHHAHHHABHHHAHBHHAHH

*000657_00110646 HBAHBHHHHAHAHHHBHAABHBBABHAABHHBHHHBBHHBHAHHAHAAHBHBHAABBHHHBAHAABAAAHBHBBHHBHAAHAAHHBAHHBAHHBHBHAHBHBBBAABHHHHABHHAHHHHHHBHHHHHHHAHBAHHHBHAAHHB

*000700_00301713 BAHHAHAHHHAHHHHABAABAHHHHHHBHHHAHABHHABHBHAHHAHHAHHHAAHBBABAAHHHHHBBHABAABAHAHHAHAHBAHAHBBAHHAHHBBHAABHHHHHBBAAHHHHBABHAHHBAHBHBABHHHBHBHBAHAHHA

*000742_00220143 AHHHAHHAAHHHABHHHBBBHHHHBHABHAAHBBHAHAHAHHHHHAHAHHHBHHBBHHBHHHABHHAHHBBHBABHHHHBBHHHAAAABAHBHBBHAHHBHBHHHBBBHBBHBABHHAHHBAHHAHHBHBHBHBAAHBABAAHB

*000747_00269820 AHHBAHBBAHBHHHAHBHBHBHBAABBBBHHBAAHHHHABHBHHAHHHAAAAHHAHBBAHBHAABHBBBHAAHAAAAHHHBHHBHAHHHHHAAHHHBHHHHHHHHBHHBABHBHHBBAHAHHBHBBAAABHHHBHAHHHHHAHH

*000855_00202154 AHHBHBBHHHHBBHAHAHHAHHBBBAHHHAABBBBBHHAHBBHHBHHHBABHBHBAHHHBBBBBABAHBHBAAHBBBBHBBHHHAHHHHAHHHAHAHHHAHBHBHAAHHHBAHHHABABBBBHHHHBHBHHHAHHHHAHHHAHH

*000983_00069609 BBHHBHAHBHHABBHBHBHHAAHBAHHHBABAABHHHHAHHBHHBBAAAABBHAHAAABAAHHBHBHHHBHABHHHABHAHHHBHHABHHABBBHAHAHAHHABHHAHABAHHHHBHHHHBHAAHBHHHHHHBHAHHHAHABBH

*000984_00080072 BAAHHAHHHHBHHAAHAAHBBHHHHHABHHHHBHBBAHHBHAHAHAHBHHBBHHHHHBHHHAAHAHAHBHBAHHHBAAABHBHBAAHBABHBBAHHHAHABHHAHHAHHABHHHHHHAABABHBAABHBBHABAABHHHHHHBB

*001011_00059561 HBHHAAHHBBHHABHHABABBHHHHHBBAHBHHBHHAAHHHBAHHABHBHBBHBHHHBHAHHHAHAHAHABHAHHHHAHAHHHHBAHHHHHABAHHAHBBHHAHABHHHBBABHAAHBABBBHBHHBHHBAHHABBHBHBHAAB

*001011_00263297 HBHHAAHHBBHHABHHABABBHHHHHBBAHBHHBHHAAHHHBAHHABHBHBBHBHHHBHAHHHAHAHABABHAHHHHAHAHHHHBAHHHHHABAHHAHBBHHAHABHHHBBAHHAAHBABBBHBBHBHHBAHHABBHHHBHAAB

*001216_00148503 BBHABHHBHAHAHHHBHAABHBBABHAABHHBHHBBBHHBHHHHAHAAHBHBHAAHBHAHBAHAABAAABHHHBHHBHAAHAAHHBAHABAHHBHBHAHHHBBBAABHHHHABHHAHHHHHHBHHHHHHHAHBAHHHBHAAHHB

*001342_00062794 BAAHHHAHHHBHHAAHAAABBHHHHAABHHHHBBBBAHHBBAHAHAHBHHHBHHHBHBHHHBHHABAHBHBABHHHAAAHHBHBAAHBBBHBBAHHHHHABHHAHHAHHABHHAHHHHHBAHHBAABHHBHABAABHBHHHHBB

*001380_00123036 HBHHHBHBAAAHBHBBBAHHBBHHAABHHAHBAHHBBHAHHBHHHAHAHAAHBHAAHHHHBBBHHHAHAHHHHBHAAAHHBHBHHBHBHHBAABHHAHHAHHHAAAABHHHBHBABHHBAHHHBBHHBHAAHBHABHBHHHBHA

*001496_00094699 BBAHBHHHHHBAHAHBHBAAABHBAABHHHBHABABHHAAHHHBBBBAAABHBAHAHAHHHBHBHHHHHHHAHHHHAHBABHBHHAHHHBAHBBAAHHAHHHABAHAHABHHHAHHBHHHHAHAAHBBHHABHAAHHHAHHHBB

*001580_00250376 HBHHAAHHBBHAABBHABABBHHHHHBBAHBHHBHHAAHHHBAHHABHHHBBHBHHHBHAHHHAHAHAHABHBHHHHAHAHHHHBAHHHHHABAHHAHHBHHAHABAHHBBABHAAHBABBBHBHHBHHBAHHABBHHHBHAAB

*001633_00210549 BBHABAHHAAHHHHBBBAAHBBBHBAAHBHBBHHHBABBHHHABHHHAHAAAHAABHHAABBBAHHHABBHHHBBHHHAHAAAABHHAHHAHHBHHHAAHHBBHAHAHAHHHBHHAHHAHHHHBAHHHHHAHBAHAHHHBHBHH

*001728_00168749 AHBBHBAABBHHHHHHBBAHHHAHHHHHAABHHHBHHHHBAAHAHBBHBHBHBHHBHHBBHHAAHBBBBBAABHAHABBHAAHHHHAHHHHHHAAHBHAHHAHHAAHAABBHBHAAHHBHBHHHBBHHHBAHHAHBBHHBAHHB

*001892_00010602 AHAHAHBHAAHAHAAHHHBBHHABAHBBBAHAHHAAHBHHHHHBBBHHAHAHHHHBAAAAAHHHHHABBHBAHHAHHAHAHHBHHHHHBHHHHAAAABHHAHAHHABHHHBAAHHHBAHBABAHHAAAHHHHBBHHBAAAHHHA

*002017_00055088 HAHHHBHBBBHBABHBBBHBHBHHHHHHAHABABHHBHAAHBHHHHHHAAHAHBBAAHHHBBBBAABBAAHBHAHHHAHHHABHBHHHHAAHHHBAHBBBAABAHAAHHBHBBHAAHHBHHHAHHHBBHHHHHHBHHBBHHBHH

*002031_00153619 BAAHAHAHHAHHHAHAHHAABAHHHAAHHHHAHBBHABBBHAHAHAABHHHBAHABHBHHABHBABHBHAHHBHHHAAHHABBHAAHBBBHBHAHHHABHBHHAHHHHHABHHAHHAHHBHAHBAAAAHBHHBAHBHHHHHHBB

*002236_00021575 AHHHBBBHHAHHBHHBHHHAABHBHBABBHBAAHHHHHAHHBHBBAHAABHHAABBAAAHAHBHHHAHHBAHHHHAHBHHHBHBHBHHHHBAHHBBHBHBBHAABAAHHHBHHABABHBHHHAAHHHAAHBHBAHHHBBHHBHH

*003006_00016013 AHHBAHBBAHHAHBAAHBBHHHBHABBBHHABHABHHHABHBBHHHHHAAAABHHAHBHHBAAABHBBAAHAHAHBAHHHHAABAAHHHHHAABHHBHHBHHHHHHAHBBBHBHABBAHABHHHHBAAHBHHHHHHHHHHHAHH

*005075_00021799 HAHABAHHBHBHHBABHHHHAHBAHBHBHHHHHAHHHHHHHHBBAAHHHBBAAHHHHHHAHABABHHBAHBHAAHBABHAHAABAAHAHHAHAHAHAABHHHBBHHAAHBHHBHHAHBAHBAHAHBHABBHHABHAHHAHHHBB

*005668_00012288 AHHBHHABHHHABBAAHBBAHHHBHBBHHBABHABHHHAHBBBHBHHHAAAABHHHHHHHBAAHHHBHAAHAHABBHHHHHAHBHAHHHBHAAHAHBHBBHHHHAHAHBBHHBHABAAHABHHHHBAHBBAHAHHBHBAHHAHH

*000670_00165143 ABHHHHHAABHAHHHHBHBBHAHABHBAAAHHABAHBAAHBHBHHHHHAHHHBHBHAHHHHBABHHBAAAHHHAHAABAAABBHHBHABHHHHBBHHHHHHBHHAAHBBHABAAABHHHABAAH-HBBAHAHHAHHHAHHHHBH

*002106_00013640 ABHAHHABBHHHHHHAAHHAHHHHHBHBBBHHHBBAHHHAAAHBHBHBHHHHHHHAHAHAHHHBBHHAABHHBABHBHAABHHAHABHHHHHHHBBBABBHHAHBBBHAAHHBHABAHAHHHHHAAABHBBHBHBBHHBBBAB-

*002271_00011401 ABHAHHABBHHH-HHAAHHAHHHHHBHBBBHHHBBAHHHAAAHBHBHBHHHHHHHAHAHAHHHBBHHHABHHBABHBHHABHAAHABHHHAHHHBBBABBHHAHBBBHAAHHBHABHAHHHHHHAAABHBBHBHBBHHBBBABB

*000062_00072532 HHHAHAAABHHHAAHHABHHHAAHHBAHAAHAHABBHHBAHHHHHHABHHBHBHAHBHHHAHBHHBBAHHHHAHBHBBAHHHBBHAHAAHAHHABAHABHHBHHHBBHBAHBBHHHAHAABHBHAHHBHBHHHHBABABBHHAB

*000142_00420438 ABHBBBHABHABABHAHBHHHBHBHBBHAHAHBHHBHAHABHABBAHHHBBAHBHHHHHHHAHBAHBBHHBAAHHHHHABHBHBHBHBBBHHHABHAHHHHAHHAHHABHBAHHHAHABHHAHHHHHHHBABAAHBBBHBHAAH

*000202_00467228 BHHBHAAABBBHAABHABABBABHHHAHHAAAHHABBBHHHHHHHHBHHAHBBAHHHAHBAHHABHHABAAHBAHHABBHHAAHBHHBBHAHBAHHBAAAHHBBHHHHHHHHAHAHAAAHHHHBBHABHHHABHHHAHHHHAHH

*000285_00293364 BHHBHAAABBBHHABHABABBABHHHAHHHAAHHABBBHHHHHHHHBHHAHBBAHHBHBBAHHHBHAABAHHBAHHABBHHAAHBHHBBHAHBAHABHAAHHBBHHHHBBHHAHAHHHAHHHBBBHABHHHABHHHAHHBHAHH

*000332_00179148 HHHAHAAHBHHHBAHHABHHHAAHHHAHAAHAHABBHBBAHHHBHHABHHBHBHHHBHHAAHBHHBBAHHHHAHBHBBAHHHBBHAHAAHAHHABAHABHHBHHHBBABAHHBHBBHHAAHBBHAHHBHBHHHHBHBABBHHAB

*000340_00070845 BHHAHHAAAHBABAHBBHHHHHHHHABAHAAHBHHBHHHBHHABHBBAAAHHHHAABHHBHHHHHHBHHAHAAHHHHBHAHAHBHHBAAHBBBHAHABHHBBABBHAHABABHABHHAHHHBBHHAHAHHBHBHAHHHAABAHB

*000357_00070949 HHHHAHAHHHABHBHABAHBHHHHHAHBHAHAHABHHABHBHAHHAHHHAABAAHHBBBAAHHHHABBAABAHBAHAHHAHAHHHAABBBAHHAHHBBHHABHHHHHBBAAHHHHHABHAHHBHHBABHBHHHBHBHBAHAHHA

*000415_00078145 ABHAHHAHBHHHAAAAAHHAHHAHBBABBHHHHHBAHHBAAAHBHBHHAHHBHHHAHAHAHHHABBHHABHHBHBHBHHAHABHHHBHHHAHHABBBHBBAHAHHBBAAAHHBHHBHHHAHBHHAAABHBHHBHHBHHBBBAHB

*000468_00187934 BHHBHAAABBBHAABHABABBABHHHAHHHAAHHABBBHHHHHHHHBHHAHBBAHHHABBAHHABHAABAHHBAHHABBHHAAHBHHBBHAHBAHHBHAAHHBBHHHHBHHHAHAHHHAHHHHBBHABHHHABHHBABHHHAHH

*000592_00235638 HHHBABBAHAHBBAHHHAHAHHHBBAHHHAHBHBHBAAABBBHHBHHHHABABHBABHHBBBBAABAHBHBAAHBHHHABBHHHHHHHAHHHHAAAHHHAHBHBHHAHHHBAHHHABABBABBHAHHHHHHHHHHAHAHHHAAH

*000631_00106852 ABBHBBBHHAHHBHHBHAHAABHBHBABBABAAHHHHHAHHBHBBAHAABHHAABBAAHHAHBHAHAHHBAHHHHAHBHHHBHBHBHHHHBAHHHBHBHBBHAABHAHHHBHHHHAHHBHAHAAHAHAAHBHBAHHHBHAHBHH

*000716_00042653 ABHHHHHAAHHAHHHHBHBBHAHHBBBAAAHHABAHBAABBHBHHAHHAHHHBHBHAHHAHBAHBHBHAAHHHAHAAHAAABBHHBHHBHHHHBBHBHHHHBHHAAABBHABAAABHHAABHAHBHBBAHAHHAHHHAHHHHHH

*000859_00055016 ABHBHHBHBHABHBHHBHHAHAHAAAABBBHHHBHHAAHHBHABABHBHBBAHHHAAHHBHBBAAHHHAAHAAHBHBAHHBHAHHAHHHABHBAHBHBBAHBHAHBHHHHAHHHHBHHHHHHAHAHAHHHHABHBBAHAAHAAH

*000928_00034402 HHHBABBAHABHBAHHBAHAHHHBBAHHHAHBHBHHAAABBBHHBHHHHABABHBABHHBBBBAABAHBHBAAHBHHHABBHHHHHHHAHHHHAAAHHHAHBHHHHAHAHBAAHHAHHBBABBHHHAHHHHHHAHAHAHHHAAH

*001045_00043403 HHAAHAHBBBAHHBABHHABAHBHHBHHHHHHAHAHHBHBBHBHHHHAHHABHHBHHHAAHABABABHHHBBHAAHAHHBAAAHHHAHBHAHHHHAHAHBHHAAHAHHAAHHHABAAHABBBHAHBAHHHHHHBAHHHBBABBH

*001371_00042383 ABHAHHABBAHHAHHAAHHAHHHHHBHBBHHHHBBAHHBAAAHBHBHBHHHHHHHAHAHAHHHBBHHAABHHBABHBHHAHHHHAABHHHAHHABBBABBHHAHBBBHAAHHBHABAHHHHHHHAAABHBBHBHBBHHBBBAHB

*001374_00115275 BHHABBAHAAHHHHAAHHHHHHHHHAHBAHABHAHABBHBHBAHHHAAHHHHHHAHBAAHBBAHHHAHBBBHBBBHAHBBHABHBBHHHHABBBAHAHBAHHAHHBHHBBHHBAABAHBHBHBHAHHAHAHBBAHHAHAHHHHH

*002355_00066871 HHHHHHHHHAHHAHHBAHAHHHABBBBHAAHAAHBBHHHHHHABBHHHBHBAHBAABHBBAHBBHHBHHAABHBABHHHHBHBHHAHAABHAHHAHAAHHHHHHAHAHHAHBBAAHHHHABABHAHHBBBAAHABABABHHHHH

*002733_00057316 ABHBHHBHBHABHHHBBHHAHAHAAHAHBBBBABHHAAHHBAHBAHHBHBBAHHHAAHHBBBBAAHHHAAHAAHBHBHHHBHAHBAHHHHBAHAHBHBBAHBHAHBHHHHAHHHHHHHHHHHAHAHAHHBHABHBHABAAHHAB

*003303_00092283 HHHHAHAHHHAAHHHABAAHAHHHHHHBHAHAHABHHABHBHAHHAHHAHABAAHHBABAAHHHHHBBBABAHBAHAHHAHAHBAHAHBBAHHAHHBBHAABHHHHHBBAAHHHHBABHAHHBAHBABHBHHHBHBHHAHAHHA

*003723_00009795 HHHAAHBHHBHBBHAHHBABHHHHHHAHAHHAHHBAAABHHHABBHBHBHABHBAAHHBHAHBBAHHAHHBABHHBHHAAHBHABHHAAAHHHHHBHHHAAABAHHHAHHAAHBHAHBHABBHHAHHABAHHHHHBBBBHHAHB

*004015_00059732 ABHBHHHHBHABBBBHBHBHHHHHHBHHBBHHBAHHBHABHBHBHABBABHHABHBBHBHHHHHHHHAHBHABBAHAABHBBHBHHHAHHHHBAHHHHBAHBHAHABBBHBAHHAAHHHHHABHHHBBHAHHHBBABAHAHABA

*005334_00037458 BHHBHAAABBBBAABHABABBABHHHAHHAAAHHABBBHHHHHHHHBHHAHBBAHHHAHBAHHABHAABAAHBAHHABBHHAAHBHHBBHAHBAHHBHAAHHBBHHHHHHHHAHAHAHAHHHHBBHABHHHABHHHAHHHHAHH

*000079_00755099 HHABHAHBAAAAHHAAHHBBBAHHHBAHHABBAHHHAHHHAHBAHBHAAHHHBAAHHHHABBHHHAHHAHAHAHAHAHAHHAAHBHHHAAAAABHAHABAABBHHH-HHBHHHHAAHHAHHAHBBBAHAHHHHHAAHHHHHBAB

*000136_00421566 AHHBAHBBAHBHHBAABHBHBHBAABBBBHHBAAHHHHABHBHHAHHHAAAAHHHHBBAHBHAABHBB-HAAHAAHAHHHBHHBAAHHHHHAAAHHBHHHHHHHHHHHBHBHBHHBBAHAHHBBBBAAHBHHHBHAHBHHHAHH

*000140_00198226 HBBBBBHABHABAHHAHHHHHBHBHBBHAHAHBBHHHAHABHHBBAHHHBBAHBHHHHHHHAHHAHBBHHHHAHHHAAAHHBHBABHB-BHHHABHAHHHHAHHAAHABHBAHBHAHABHHAHHHHHHHBABAABBBBHBHAAB

*000348_00175185 AHBAHABHHBHB-ABAAHABAHHHHHAHHHAHHBHHBHBHHBHHHAHBAHAAHHAABBAHHHBBBHHBHBAHBHHBBHBHHHHHHHHBABAHHHAHHHBAAHABHBBHHHBAHBABHBAAHHBAHAHBHBHAHAHBHHABHHHA

*000408_00275443 AHHBAHBBAHHAHBHAHBBHHHBAABBHHHABHABHHHABHBBHHHHHAAAABHHAHHHHBAAABHBHAAHAHAHBAHHHHAHBHABHBHHAAHHHBHHBHHHHHHAHBBBHBHABBA-ABHHHHBAHBBHHAHHBHAHHBAHH

*000567_00064291 HAHHHHHHBBHAABHBHBABHBHHHHHHAHABABHHBHAAHBHHHHAHHAHAHBBAAHHHBBBBAABBAHHBHAHHHAHABABHBHHHHAAHHHBAHHBBAABAAAAHABHABHAAHHHBHHAHHHBBHHAHAHHAB-HHHHHH

*000684_00210965 AHHBBHABBHH-AHAHHBAHBBABBBHAHAAHHAAHHHAHHBAAHHBHAHAHHHHHBHHHHAAHHHAAHBBHBHBHHBHHBHHBHAHAHBHHHBBBHBHHBHHHBBABHAHBBHABHAHBBBBBBBHAHHBHHHHAHHBHHBHA

*000742_00223922 AHHHAHHAAHHHABHHHBBBHHHHBBABHAAHBBHAHAHAHHHHHAHAHHHBHHBBHHBHHHABHHAHHBBHBABHHHHBB-HHAAAABAHBHBBHAHHBHBHHHBBBHBBHBABHHAHHBAHHHHHBHBHBHBAAHBABAAHB

*000776_00231095 AHAHABBHAHHAHAAHHBBBHHABHHBBHAHAHHAAHBHBHHHBBBHHAHAHHHHBAAAAAHHHHBABBHBAHHAHHAHHHHBHHHHHBHHHH-AAABHHABHHAAHHHHHAAHHHBABBABAHBAAAHHAHBBHHBHAAHHHA

*001234_00070082 BAAHAHAHHAHHHAHAHHAABAHHHAAHHHHAHBBHAHBBHAHAHAABHHHBAHABHBHHABHBABHHHAHHBHHH-AHHABBHAAHBBBHBHAHHHABHBHHAHBBHHABHHAHHAHHBHAHBAAAAHBHHBAHBHBHHHHBB

*002205_00018934 AHBAHABHHBHHHABAAHABAHHHHHAHHHAHHBHHBHBHHBHHHAHBAHAAHHAABBAHHHBBBHHBHBAHBHHBBHBHHHHH-HHHABAHHHAHHHBAAHABHBBHHHBAHBABBHAAAHBAHAHBBBHAHAHBHHABHHHA

*003249_00065723 AHHBHABBAHHHHAAAHHBHBAHAHAAHHABHHHHHHAHHAABAAHHHAHHHBABBHHHAHBHBHABHAHAAAHAHAHAHHAAH-HAHAHHAABHAHABHHBBHHHBAHBHHHHAABHAHBAHBHBAHHHHHBHAAHBHHABHB

*007680_00009559 AHHBHHBH-HBHABAHBHBHHAHHABBBBHHBAHHHHHABHHAAHBHHABAAHHABBBAHBHHHBHBHBHAAHAAAAHAHHHHBHAHHHHAAAHHHBAHHAAAHHBHHHABAHHHHBAHAHHBHHBAHABHHHHBABAHHHABB

*000168_00452204 HHHHAHABHHABHHHABAHBHHHHHBHBHAHAHABHHABHBHAHHAHHAHABAAHABHBAAHHHAHBBAABAHBAHAHHAHAHH--AHBBAHHAHBBBHAABHHHHHBBAAHHHHHHBHAHHBHHBABHBHHHBHBHBAHAHHA

*000428_00428757 BHBHBAHBBHHHAHHHHHAHAHHAABHBAHAHBAHAHHHHBHBBAAAHAHHAAAHAHHHHHABABHHBHBHHAHABABHHHBABAHHHHAAHHHHBBABABHABHH-HHBBAHHHBHBAHBHHAHHBABBHHHAHAH-AHHHBB

*000477_00254828 HHABHHHAHHHBAHAAHBHBBHHBBBAHAAHHAHBAHHHH-AAABBHHHHHAHHBHHAHHHHBAABBABHAHHBHAAHHHAHAHAHAHHHHAAAABBHABHBHHAA-HAHBHBHAAHBHHHAHBHHBHABABHHHHHBABAHHH

*000745_00356480 ABHBHHBABBHBHBHHBHHAHHHAAHABBBHHBBHHAAHHBHABABHBBBBAHHHAAHHBHBBAAHHHAAHAAHHH-AHHBHAHBAHBHHBHHAHBHBHAHBHAHBHHBHAHHHHBBHHHHBAHAHHHH-HBBHBBAHHAHAAH

*001008_00245483 ABHBHHBHBHA-BHHBBHAAHAHAAHAHBBHHABHHAAHHBA-BAHHBHBBAHHHAAHHBHBBAAHHHAAHAAHBHBAHHBHAHAAHHHHBHBAHBHBBAHBHAHBHHHHAHHHHHHHHHHHAHAHAAHHHABHBHABAAHAAH

*001594_00048874 HHABHAHAAHHBAHAAHBHBBHHHH-AHAAHHAHBABHHBAAHHHBHHHHHHHHBHHAHHHABHABBABHBAHBHAAHHAAAAHABHHHHHHAAABBAHBHBHHAAAHHBBHBHABHHAHHAHBHHBHAHABBBHHH-AHHBHH

*001602_00051090 ABHBBHABHHHHHHAHHBAHBBAHBHHAHAAHAAAHHHAAABHAHHBHAHAHHHHHBHHHHAAHHHAAHBBBBHBHHBHHB-HHHAAAHBHHHBBBHBHHBHHHBBABHAHBBHABAHHBBBBBHBHAHHBHHHHAH-BAABHH

*002792_00009497 HHAAHHHBBBAHHBABHHABAHBHAHHHHHHHAHAHHBHBBHBHHAHAHHABHHBHHHAAHABAHABHAHBBHAAH-HHBAABHHBAHBHAHHHHAHAHBHHAAHAAHAAHAHABHHHHBBBHA-BHBHHHHHBAHHBBHABBH

*003443_00047974 HHBBHHAABBHHHHHHBBAHHHAHHHHHAABHH-BHHHHBAAHAHBBHBHBHBHHBHHBBHHAAHBBBBBAABHAHABBHAABHHHAHBHHHHAAHBHAHHABHAAHAABBHBHA-AHHHBHHHBHHHHBAHHAHHBAHBAHHB

*005324_00007514 HHBHAHHABHABAHBHHAHBHHHBHAABAHHABBHHHBHHBHHBBAHBHBHAHBAABHHHAABHAHBHHHHHHHHH-AAHHHHBABHBBAAHH-HHABHBBAHAHAHABHHBBHHAHABHHABHHHHBHHABHHBHBBHBHAAB

*001914_00137127 HAHBAAHHAAAAHH-AHHHBBAHBHBHHHABBAHHBAHHHAHBABBHAHHHHBAAHHHBABHHHHAAHAHABAHAH-HAHHAAH-HBHAAAAABHHBABAAHBHHHBHHBHHHHAAHHAHHAHBHBAHHHHHHHAHHHHABBAB

*002506_00059315 AHBHHHAHBBB-AHABHHAABHHAHAAHAHHHHHHBBAHBHAAHABHHAHBBHHHHHHHBHHHAHABBAH-HBAAH-BBHHABHAHHAAAHHHHHHAHHHHAHHAHBBABBHBAAAABHHHBHBHHHHHAAABHBBBHHBHAAH

*000850_00170907 HHHBAHBBA-H-HHAAHBBHHHBHABBBBHABHABHHHABHBBHHHHHAAAABHHHHBHHBAAABHBBAAHAHAHBAHHHHAHBBAHHHAHAAHHHBHHB-HHHHH-HBBBHBHABBAHAHHHHHBAABBBHHHHAHBHHHAHH

*001214_00218762 HHAAHAHAABHBAH-HHHHBBHH-HBABAAHHAHBHBHHBAAHHHBHHHHHHB-BHHAAHHABHABBABHBHABHAAHBAHHAHABBHHHHAAHHBHAH-ABHHHAHHHBBHBHABHHAHHAHBBHBHAHHHBHBBHBAHBHHB

*004372_00019306 HBHBHAHBHBBHHBAHHAHHBHAABAHBHBHHHHABHHHBHAABAHHBBBBHHHBHHHHAHAABAHHHABBHHHAHAHHBHB-B-ABHABBBBAHBHHHABBHHHH-AHABHHHHHHAABHBHBAABHAAHHHAH-H-HHBHBH

*005960_00029760 HBHHHHAABBBHBHAHH-BBHHHHABHHHHHABHBHHHBH-BHAHHHHHHHABAHHHBHHAABBABHABHAAHHBA-BAABHHHHABAHAHHHH-HBHHHBHBABABBBBHHBBAB-HHHHHHHBHHAABBBHHHHBBBHBAAB

*001134_00177664 A-AHHHBABHBHHBBHBHAHAHBAHBBBHBAHB-BHHAHAAHAHABAHHHHHBHA-HBHBHBHAAHHAHAAHHHHHBABHHHAHAAHBHHBAHBHHHBB-HBAHBB-HBHAHHHHBBHHAHBHA-HHAHHBBH-AHA-HABAHH

*000138_00125878 ABBHHHABAHB--HBHHHHAHHBAHAHHAHHHHHHAHAHHHB-HAHHHHHABHHBBBHAAHHHHBHABHBBABAHBHHHBHAHBBAAAHBHBHHAAHHHABHAHH-BAHBBAAAHA-BHABHBH-HAABBHHHAB-H-AAAHHH

*005839_00006899 BHHA-BBBHHHHBHAHHHABHH-HHHAHAHHAHBBAAAHHBHAABHBHBHABHBAAHHBHABBBAHHAHBBABHHH-HAHH-BABHH--ABHHAHBAHHAAABAHHAAHHAAHHH-BHBABBH-HHHAHAHHHHHHBHBHHAHB

*000830_00175710 HAHABAHH-HBB-BABHHHHAHBAHBHBHBHHHAHHHHHHHHBBAAHHHBBAAAHHHHHAHABABHH--HBHAAHB-BHAHHABAAHAHHAHAHAHAABHHHBBHB-HHBHHBHHH-BAHB-HAHBHAB-BHABHAHBAHHHBB

*001015_00128215 HBHHAAHHB-B-HBAHABHBBHHHH-BBAHBHHBHHAAHHHB-HHABHHHBBHBH-ABHAHHAABAHAHA-HBHHHHAHAHHBHBAHHH-HAB-HHABHBHH-HHBHHHBBABHAAHH-BBBABHHBHHBBHHABBHHHB-AAB

*000556_00037571 A-HH-HHHBBHBABHABHHBHA-ABHBAAAHB---BBHAAHBHHABHAHAHHHHHHHH--BBBBHABH--HBHAH--AHHA-BHHHB--AABHHBHBBBHAABABHHHHBHBHBA--HHHHHHHHHHHHHBHAHB-HBBBABHA

*002958_00060961 HBAHHH-ABHHABB-BBHHHAHB-HBBBHBAHB-HHHA--AHAAABAH-HHHBBA-HBHBHBHAAHHAHAAHAHHH-ABHHHAHAAHBHHBAHBBHHBB-HB-HBH--BHAHHHHBHHHHH-HABHHAB--BHAA-A-HAHHH-

*000219_00086243 A--BBHBA-HB--H-AHHHB--HHAAAHHHBHHAHAHHHBAHBHAHAHBHHHBHABHBHHH-HHABHHABBHHHHBAHBHA-HB--H-ABHAAHHHBAA-BB-AH--BHBHBBAA-HHHHBHHBBAHHB-HBBHHAH-B--HBA

*000101_00497385 -AHAA-HHAHH-ABHH-H-HAHAHH-HAAA-BBBAHA--H-HAAHH-B-BHAHHABHHHAB-HHAHHABB-AHAHH-AHHH--BA-HHAHHHHABAHABHAAHHAAAB-H-AHHHH-BHBB-HA-HHH--AHBH--A-BHHAAB

*001162_00106981 HHHBHHHAAAHHHH-HHHHHAHHH--A-HB-HAABAHB-H-BBHHAHH-HABH-B--H-BBAHHHBH--HBHAHBB--HAAA-H-HHBA-ABBHHBHAHHABAHAH-AHHAABHH--HBBH-AA-AAHBBABHHH-BAA--HH-

*000009_00143922 ABBBB--HA-B--H-HHH-HH-HH--AH-HBHAAHABHAB-H-HHHAHHHHHBAA-ABH-HABAABHA-B-HHB-B-H-H---B--AA-AHAHBHHBHH-BHHHBH-BBB-HBAA-HHHHB-AH-AHH-BHHBHHAH-B-AHA-

*000033_00809478 AAHHAAHHHHAABBHAHBHBHHHBBHHHHABBAHHBAHHBHHBABHHAHBAHBAAHHABHBHBAHAHHAAABHAABHHABBAAAHABHHHAHAHHHHHHHHABAAHBHHHHABBAHHHAHBHHHBBHBBHAHHHHAHHHHBBAB

*000035_00685630 AAHHHAHHHHAABBHAHBHBHHHBBHHHHABBAHABAHHBHHBABHHAHBAHBAAHHABHBHBAHAHHAAABHAABHHABBAAAHABHHHAHAHHHHHHHHABAAHBHHHHABBHHHHAHBAHHBBHBBHAHHHHAHHHHBBAB

*000034_00298767 AHHHABAHAHHHBHHABHBBBHHHHBHAAHHABHBHBAHBHHBHHHBHAHHABAHHHHBAAHHBHBBBHBAABHHAHHAHHBBHBHHBHAHAHBBHBAHBBBHAAHHHHHHHBAABABBABHHBBHHBAHAHHAAHBHBHHHHH

*000036_00435213 AHHBAHBBAHBHHHAHBHBHBHBAABBBBHHBAAHHHHABHBHHABHHAAAAHHAHBBAHBHAHBHBHBHAAHAAAAHHHBHHBHAHHHHHAAHHHBHHHHHHHHBHHBABHBHHBBAHAHHBBBBAAABHHHBHABHHHHAHH

*000072_00600970 HBAHHBHBAAAHHABBHHHHBBHHHHBHAHHHAHHBHBABHBBAHAAAHAAHBHAAHHHHHBHAHHAAHHHHHBHAAHHHBABHHAHHAHBBABBHHHHAHHHAAHABBHBBHBAHHHBAHHHBHHHBAAAHAHABHBHBHBBA

*000103_00015853 HAHBAHBAAHHAHBHHHHHBBBBHBBHBHHHBBBBHAAHBAAAHHAAHHAHHBBHBBBHHBHHHAABHHABHAAHBBAHBHBHAHAHHBAAAHHHHHHBHBHHBHHAHHHAHAHHBBBHAHHBBAHHHBHHHBAHHAHBBHAHH

*000146_00079732 BBHABBABAHHAAHAHHHHAHHHHHAHBAHHHHHHAHBHHHBHBHHAAHHAHHAABBAAAHBHHBBHHBBBBBHBBAHBBHABBBHHHHBHBBHAAHHBAHHAHHHBABBHHHAHHABBHBBBHAHHABAHHHAHBHAAHHHHH

*000165_00007000 HAHBAAAHHAAAHHHAHHHBBHHBBHHHHABBAHHBAHHHAHBABBHAHBHHBAAHHABHBHBHHAAHAHABAHAHHHABHAAHBHBHHAAHABHHHAHHHABHHHBHBBHAHHAAHBAHBAHBBBHHHHHHAHHHHHHABBAB

*000165_00193698 HAHBAHHHHAAAHHHAHHHBBHHBBHHHHABBAHHBAHHBAHBABBHAHBHHBAAHHABHBHBHHAAHAAABAHABHHABHAAHBHBHHHAHABHHHAHHHABHHHBHBBHAHHAHHBAHBAHBBBHHBHHHAHHHHHHABBAB

*000214_00342996 BAHHAHAHHHAHHHHABAABAHBHHHHAHHHAHHBABABHBHAHHABHAHHHAAAHBABAAHHHHHBBAABAABAHAHHAHAHBAHAHBBAHAHHHBBHAABHHBHHBHAAHHHHBHHHHHHBAHBHBHBBHHBHBHHAHAHAA

*000290_00329801 BBHABHHHAABHHHBBBAAHHHBHBAAHBHBBHAHBABBHBHABHBBAHAAABAABHHAABBBAHHHHBHHHHBBHHHHHAAHAHAHAHHAHHBAHBAAHHBBHBHAHAHHHHHHAHHHHHHHHAHHAHHBHBAHAHHHBHBBA

*000311_00054267 AHBAHABHHBHHBABAAHABAHHHHHAHHHAHHBHHBHBHHBHHHAHBAHAAHHAABBAHHHBBBHHBHBAHBBHBBHBHHHHHHHHBAHAHBHAHHHBAAHABHBHHHHBAHBABBHAAAHBAHAHHHBHAHAABHHABHHHA

*000380_00037904 HBAHHBHBAAAHHABBHHHHBBHHHHBHAHHHAHHBHBABHBBAHAAAHAAHBHAAHHHHHBHAHHAAHHHHHBHAAHHHBABHHAHHAHBBABBHHHHAHHHAAHABBHBBHBAHHHBAHHHBHHHBAAAHAHABHBHBHBBA

*000380_00120264 HBAHHBHBAAAHHABBHHHHBBHHHHBHAHHHAHHBHBABHBBAHAAAHAAHBHAAHHHHHBHAHHAAHHHHHBHAAHHHBABHHAHHAHBBABBHHHHAHHHAAHABBHBBHBAHHHBAHHHBHHHBAAAHAHABHBHBHBBA

*000425_00188497 BBHABAHHAABHHHBBBAAHBHBHBAAHBHBBHAHBABBHHHABHBHAHAAAHAABHHAABBBAHHHHBHHHHBBHHHHHAABABHHAHHAHHBAHBAAHHBBHHHAHAHHBBHHAHHAHHHHBAHHHHHHHBAHAHAHBHBHH

*000553_00011842 BAAHHAHHHHBHHAAHAAHBBHHHHHABHHHHBHBBAHHBHAHAHAHBHHBBHHHHHBHHHAAHAHAHBHBABHHBAAABHBHBAAHBABHBBAHHHAHABHHAHHAHHABHHHHHHAABABHBAABHBBHABAABHHHHHHBB

*000606_00545407 BBHABAHHAABHHHBBBAAHBHBHBAAHBHBBHAHBAHBHBHABHBHAHAAAHAABHHAABBBAHHHHBHHHHBBHBHHHAAHAHHHAHHAHHBAHBAAHHBBHHAAHAHHBBHHAHHAHHHHBAHHHHHHHBAHAHHHBHBHA

*000608_00131173 HBHHHHAABHBHBHAHHBBBHHHHABHHHBHABHBHHHBHBBHAHHBHHHHABAHHHBBAAABBABHHBHAAHHBAABAHBHHHHABHHAHHHHBHBAHHBHBABABHBBHHBBABAHHHHHHHHHHAABBBHHHHBHBHBAAB

*000724_00235034 BBHABAHHAAHHHHBBBAAHBHBHBAAHBHBBHHHBABBHHHABHBHAHAAAHAABHHAABBBAHHHABHHHHBBHHHAHAAHABHHAHHAHHBAHBAAHHBBHAHAHAHHBBHHAHHAHHHHBAHHHHHAHBAHAHHHBHBHH

*000770_00001491 AHHHABAHAHHABHHABHBBBHHHHBHAAHHABHBHBAHBHHBAHHBHAHHABAHHHHBAAHHBHBBBHBAABHHAHHAHHBBHHHHBHAHAABBHBAHHBBBAAHHHHHHHBAHBABBABHHBBHHBAHHHHAAHBHBHHHHH

*000776_00060674 AHAHABBHAHHAHAAHHBBBHHABHHBBHAHAHHAAHBHBHHHBBBHHAHAHHHHBAAAAAHHHHBABBHBAHHAHHAHHHHBHHHHHBHHHHAAAABHHABHHAAHHHHHAAHHHBABBABAHBAAAHHAHBBHHBHAAHHHA

*000786_00314793 BBHAHHABAABHHBBHBBHHAABAABHHBHBHBBAAABBBHBAHAAHHAAAABABBHHAHHHHHHAHAHHHHBBBHHHAHAHHABAHHHHAHBBAHHHAHAHHHBBAHAAHHAABHHHHHHHHAHHHHHHBHHABAHHBHHBHA

*000910_00004794 BBHABHHHAABHHHBBBAAHHABHBHAHBHBBHAHBABBHBHABHBBAAAAABAABHHAABBBAHHHABHHHHBBHHHHHAAHAHAHAHBAHHHAHBAAHHBBHBHAHAHHHHHHAHHHHHHHHAHHHHHBHHAHAHHHBHBBA

*000910_00141953 BBHABHHHAABHHHBBBAAHHABHBHAHBHBBHAHBABBHBHABHBBAAAAABAABHHAABBBAHHHABHHHHBBHHHHHAAHAHAHAHHAHHHAHBAAHHBBHBHAHAHHHHHHAHHHHHHHHAHHHHHBHBAHAHHHBHBBA

*000984_00215261 BAAHHAHHHHBHHAAHAAHBBHHHHHABHHHHBHBBAHHBHAHAHAHBHHBBHHHHHBHHHAAHAHAHBHBABHHBAAABHBHBAAHBABHBBAHHHAHABHHAHHAHHABHHHHHHAABABHBAABHBBHABAABHHHHHHBB

*001091_00071219 AHHHABHHAHHABHHABHBBBHHHHBHAAHHABHBHBAHBHHBAHHBHAHHABAHHHHBAAHHBHBBBHBAABHHAHHAHHBBHHHHBHAHAABBHBAHHBBBAAHHHHHHHBAHBABBABHHBBHHBAHAHHAAHBHBHHHHH

*001095_00074963 AHHAHHAHBHHHHAHHABHHHAAHBHAHAAHAHHBBHBBAHHHBHHABHHHHBHBHHHHAAHHHHBBAHHHHAHBHBBAHHHBBHAHAAAAHBABHHABBABHHHBBABAHHBBBBHHAABBHHAAAHHBHHHHHHBABBHHAB

*001303_00045845 BBAHBHBHHHBAHAHBHBAAABHBAABHHHBHAHABHHAAHHHBBBBAAABHBAHAHAHHHBHBHHHHHAHAHHHHAHBABHBHHAHHABAHHBAAHHHAHHABAHAHABHHHHHHBHHHHAHAAHBHHHABAAAHHHAAHHBB

*001364_00195587 HAHHHBHHBBHBABHBBBABHBHHHHHHAHABABHHBHAAHBHHHHHHAAHAHBBAAHHHBBBBAABBAHABHAHHHAHAHABHBHHHHAAHHHBAHBBBAABAAAAHHBHHBHAAHHBHHHAHHHBBHHAHHHHAHBBHHHHH

*001499_00102296 AHBHHHAHBBBHHHHBHHAABHHAHHAHAHHHHHHBBAHBHAAAABHHAHBHHHHHHHBBHAHAHABBAHAHBAAHHBBHHABHAHHAAHHHHHHHAHAHBAHHAHHBABBHBAAAABHHHBHBABAHHHAHBAHHBAABHAAH

*001541_00042650 AHHHABAHAHHABHHABHBBBHHHHBHAAHHABHBHBAHBHHBHHHBHAHHABAHHHHBAAHHBHBBBHBAABHHAAHHHHBBHBHHBHAHAHBBHBAHBBBHAAHHHHHHHBAABHBBABHHBBHHBAHAHHAAHBHBHHHHH

*001914_00074549 HAHBAAHHAAAAHHHAHHHBBAHBHBHHHABBAHHBAHHHAHBABBHAHHHHBAAHHHBABHHHHAAHAHABAHAHAHAHHAAHHHBHAAAAABHHBABAAHBHHHBHHBHHHHAAHHAHHAHBHBAHHAHHHHAHHHHABBAB

*002062_00164020 AHHHABAHAHHABHHABHBBBHHHHBHAAHHABHBHBAHBHHBAHHBHAHHABAHHHHBAAHHBHBBBHBAABHHAHHAHHBBHHHHBHAHAABBHBAHHBBBAAHHHHHHHBAHBHBBABHHBBHHBAHAHHAAHBHBHHHHH

*002359_00005541 HBHABAHHAAHHHHBBBAAHBBBHBAAHBHBBHHHBABBHHHABHBHAHAAAHAABHHAABBBAHHHABHHHHBBHHHAHAAAABHHAHHAHHBHHBAAHHBBHAHAHAHHHBHHAHHAHHHHBAHHHHHAHBAHAHHHBHBHH

*003587_00015020 AHHHABHHAHHHBHHABHBBBHHHHBHAAHHABHBHBAHBHHBHHHBHAHHABAHHHHBAAHHBHBBBHBAABHHAHHAHHBBABHHBAAHAHBBHBAHBBBHAAHBHHHHHBAABHBBABHHBBHHBHHAHHAAHBHBHHHHH

*003879_00038335 HBHHHBHBAAAHBHBBBAHHBBHHAABHHHHBAHHBBHAHHBHHHAHAHAAHBHAAHHHHBBBHHHAHAHHHHBHAAAHHBHBHHBHBHHBAABHHAHHAHHHAAAABHHHBHBABHHBAHHHBBHHBAAAHBHABHBHBHBHA

*005137_00003781 AHAHABBHAHHAHAAHHBBBHHABHHBBHAHAHHAAHBHBHHHBBBHHAHAHHHHBAAAAAHHHHBABBHBAHHAHHAHHHHBHHHHHBHHHHAAAABHHABAHAAHHHHHAAHHHBABBABHHBAAAHHAHBBHHBHAAHHHA

*006569_00008618 AHHHABAHAHHABHHABHBBBHHHHBHAAHHABHBHBAHBHHBAHHBHAHHABAHHHHBAAHHBHBBBHBAABHHAHHAHHBBHHHHBHAHAABBHBAHHBBBAAHHHHHHHBAHBHBBABHHBBHHBAHAHHAAHBHBHHHHH

*006988_00011140 AHHBABBHAAAAHAHHBHHBHHHBHABBBAHAHHAHBHHHHHBBBHHHAHAHBHHBHHAAAAHHHHBBBBBHHHAHHHBAHABHBHHAHHHHAAAAABAHABABBHHHBHHHHHHHBABBHHHHHAHAHHBABBABBBHHHHHA

*010018_00005103 HBHHHBHBAAAHBHBBBAHHBBHHAABHHAHBAHHBBHAHHBHHHAAAHAAHBHAAHHHHBBBHHHAHAHHHHBHAAAHHBHBHHBHBHHBAHBHHHHHAHHHAAAABHHHBHBABHHBAHHHBBHHBAAHHBAABHBHBHBHA

*010846_00004034 AHBAHHHAAHHHABHHHBBBHHBHBHHBHAAHBBHHHABHHHHBHHHAHBHBHHBHBBBHHHABHHBHHBHHBAHBHBABBHAHHAAAHHHBHBBHAAHBHBBHHBHBHHAHHABHHHHHBABHHHHBBBHBBBAAHBABAAHB

*000173_00354356 ABHHHHBHHAAHHABBHABAAAHHAHAABABBAABAHHAHHHHHHHABHBBAHHABAHBHBAHAABHHAHBAAAHH-BHBHBHHBHHHAHHAAHHBBHBHHHAAHBBHHHAHHAHHHHHAHHAAHBHHHBHAHAAHHBAHHHAB

*002130_00044287 BHBHAABHBHAAHBBHHBAHHHBBHHBHHBBBHABBHAAAHHHHBHAHHAHHHHHAHBABBHHAHBAHHHHHBHAA-HHAAHHAABBAHHBBHHABAHHHBAHHHBBAAHHAHBAHBAAHHHHHBBHAAHBABAHAHHHHBHHH

*000001_00030203 AHHHAHHAAHHAHHHHBHBBBAHHHBHAAAHAHHHHBAABBHBAHHBHAHHABAB-AHBAAHHB-BBHHHAABHHHHHAHHBBHHHHBHAHAABBHBAHHHBBHAAHHHHAHAAHBHHHABHABBHHBAHAHHAAABHBHHHHA

*000201_00183592 HBHBHAHBHBBBAB-HHAHHBHAHHHHBBBHHHHHBHHABHHBBHAHBBBBHHBHHBHHAHHABBHHHHBBHBBAHAAHHHBABBAHHABBBBAHBHHHABBHHBHAABABHHHHHHAAHHHBH-ABHHABHHABHHAHBHHBH

*000291_00677697 BAAHAHAHBAHH-AAAHHAABAHHHHAHHHHAHBHHABHBHAHAHAABHHHHABABHBHBABHBABHBHAHHBHAHAABHABBHAAHHBBHBHAHHHABHBHHAHBHHHABHHAHAAHHBHAHBAAAHHBHHBAH-HHHHHHHB

*000344_00036312 BBHAHHABAABHHBBHBBHHAABHABHHBABHBBHHABBBBBAHHAHAAAAABABBHHAHHBHHHHHABHBHBBBHHHAHAHHABAHHHHAHB-AHHHAHHHHHBB-HAAHHHABAAHHHHHHABHHHHHBHHABAHHHHHBHA

*001278_00140004 AHBHHBHHHAA-HBHHABBHHHBBAAHHBHHAHBHHAHBBAHAABABAHHHHBAHBABAAABAHABBAHHHHBHHH-HBBHHAABBHHBHAHBHHHHHHBHAHHAAHBHHBBHBHHHHBHHBAHAHAHHAAHAHBHABHHHBAH

*002226_00072666 HBBHHHHHAHAHBHBHBHBHHBHHAABAHHHHAHHBBHAA-BHHHABAHAAHBHAHAHHAHBBHHHABAHBHHBHA-AHHBHBAHBHBHHBAABHHHHHHHHHAAAHBHHHBABABHHBAHHAHBHHBAAAHBHABBBHBHBHA

*003673_00022889 HBHHAAHHBBHHABBHABABBHHHHHBBAHBHHBHHAAHHHBAHHABHBHBBHBBHHBHAHHHAHAHAHABHAHHHHAHAHHHH-AHHHHHABAHHAHBBHHAHABHHHBBABHAAHBABBBHBHHBHHBAHHABBH-HBHAAB

*001175_00173057 HHHHAHAHHAABHBHABAHBHHHHHAHBHAHAHA-HHABHBHAHHAHHAAABAAHHBBBAAHHHHABBHABAHBAHAHHAAAHHHAAB-BAHHAHHBBHHABHHHHABBAAHHHHA-BHAHHHHHBABHBHHHBHBHHAHAHHA

*000258_00336344 BAHABBAHAHHBAH-AHHHHHHHHH-HBAHHHHAHA-BHHHBHBHHAABHHHHAABBAAHHBAHHHHH-BBHBBBHAHBBHABBBBHAHBHBHHAAHHBAHHAHHBBABBHHBAABABBHBBBAAHHAHAHBHAHHHAHHHHHH

*000013_01013544 ABHAHHAHBHHAAH-AAHHAHHHHHBHBBHHHHBBAHHBAAAHBHBHBHHHHHHHAHAHAHHHBBHHAABHHBABHBHHAHHBHHABHHHAHH-BBBABBHHAHBBBAAAAHBHA-AAHAHHHH-AABH-BHBHHBHHBBBAHB

*000016_00765785 -HHAAAAHHHB-BHABHHHBABHAAAAHHHBBHHBAHBBHBBHHAHBBBHHHAHABHBHBBBABHHHHHHHBAAHH-AAH-AHH-HHHHHHABHHBBAAAABHBAABHHHBHHHHA-BABHHHAHBHAHHAHHBBBHBHBHHHH

*000228_00375302 BAHHHBHAHHB-BAHBBHAAAHHHAAAHHAAHHBHAAHHBBB-HHHHBBAHAHBAHBHHBHHBHABAHBHHAHHBHAHABHA-HHHHHH-BHAAHHAAHHHBHAHBBBHBHHHHHAHA-BABBAABAHHBHBHAAAHHH-BHHB

*000150_00237437 ABHBHHBHHAA-HHHBHABAB-HHABAHBABBAABHAHAH-HBHHHABHBBAHHA-AHBHBHHAAHHHAHHAAABHAHHHBHAHHAHHAHHAHHB-BHBHHHAAHBBHHHAHHAHHBHBAHHAH-BHHHBHAHAABHBAHHAA-

*010625_00003706 HHHHHAHBBHAHHBABHHHHAHBHH-HH-HBHAHAHHBHBBHBHHHHAHHABHHB-HHAAHABHBABH-HBBHAAHAHBBHHBHHBAHBHAHHHHAHAHBHHAAHA-HAABHHABA-HABBBHABBAABHHHHBA-HBBBABHH

*000694_00154173 HBHBHH-BAHBHBH-HHHHAHHBABHHHHHHHHBHAHA-HBBHHAHHHHHABHHHBBHAAHHHHBHAB-BBABAHBBHHBHAHB-AAAHBBBHHAAHHAABHAHHH-ABBBAAAHHABHHBHBB-HAAB-BHHABHHHAAAHHH

*006718_00032439 HHHHHAHABBH-AABHBBHBHBBHHHAHHAAAHHABHBHHHHB-HHBHHAHBBAHHHAHBABHABHH--ABHBAHH-B-HAAA-BHHBBHAHBAHBBAAABHBBHA-AHHHHHHA-HAAHHHHHHHABHBHBBAHHH-A-HAHH

*000080_00002610 ABBABABAHHBAHAHAAHHBAHBHHHAHHAHHHBBHHAHHHAHHHHHHAAHHHHAABHHHHHHHHAHHHHABBHBBBABHHBHHHBBHABAHAAHHHHBBAHAHHBBHHHHABBBBHHAAABBABHHBBBHAHABBAHABBHHH

*000188_00319712 AABHAHHHABHHABHBHBHHBHBHAHAHHHHHAHHABHHBHBBHHHHAHAAHBHHHHHAHHBBBAHAHBBHBAHHBHBHBHHABBBBHABHBHAHBABHBBAABHHABHHHHABHHHAHBHAHBBAAHBBHBHHBBBHHHBAHH

*000422_00154136 ABHAHHABBHHHHHHAAHHAHHHHHBHBBBHHHBBAHHHAAAHBHBHBHHHHHHHAHAHAHHHBBHHAABBHBABHBHHABHBABABHHHHHHHBBBABBHHAHBBBHAAHHBHABHHHHHHHHAAABHBBHBHBBHBBBBABB

*000790_00225529 ABHBHHBHBAABHHHBBHBAHAHAAHAHBBBBABBHAHAHHAHBAHHBHBBAHHHHAHHBBBBAAHHHAAHAAHBHHHBHBAAHBAHHAHBAHHHBBHBAHHHAHBBHHHAHHHHHHHHAHHAHABHHHBHABHHHHHAAHAAB

*001841_00077424 ABHHHHHAAHHAHHHHBHHBHAHHBBBAAAHHABAHBAABBHBHHAHHAHHHBHBHAHHAHBABBHBHAAHHHAHAABAAABBHHBHHBHHHHBBHBHHHHBHHAAABBHABAAABHHHABHAHBHBBAHAHHAHHBAHHHHHH

*001998_00014771 ABHHHAAHHHAHAHHBBBHHHBABHHBHABHHHHHBHHAABHBHBHHBAAAHHBHAHBBBHAHHHHHHHHAHBHHAHHAHHABAABHAHHHBHHHBAHBHABBHHAAHAAHAABAHAAAAHAHHBHHAAHHAHHBAAHBBHABA

*005125_00016143 AAHHHHHHBBHBABHHBBHBHHBABHBHAAHBBBHBBHAAHBHBABHAHAHHHHHHHHHHBBBBHAHHAAHBHABHBAHHAABHBHHBBAABHBBHBBBHAABABHHHHBHBHBAAHAHHHHHHHHHHHHBHHABBHHBBHBHH

*000070_00256380 HHBHHBAHBBHHBBABHBAABBBHHAAHAHHBBHABBABHBHABHBHAAHBBHHHHHAHHBHHHHABBAHAHBAAHHBBAHAHHHBBAHHHABHHHAHHHHHAHHHHBAHHHHHHAABBHHBHBHHHHHAHABBHBBBHHBAHB

*000086_00426301 HHHHHHHHHAHHAHABAHAHHHABBBBHAAHAAHHBHHHHHHABBHHHBHBAHBAABHBBAHBBHHBHHAABHBABHHHHBHBHHAHAABHAHHAHAAHHHHHHAHAHHAHBBAAHBHHABABHAHHBBBAAHABABABHHHHH

*000089_00317029 HHBAHAHHHBHBBAHHAHABAHHHHHAAHHABHBHHBHBHHBHHHAABAHAAHHAAHBAHBHBBBHHBHBAHHHHBBBBBHHHHHHHBAHAHBHAHHHBAAHABHBHHHBBAHBABHBAAHHHAHAHHBBHAHAABHHABHHHA

*000423_00422158 BHBHAABHBHAAHBBHHBHHHHBBHHBHHBBBHABBHAAAHHHHBHAHHAAHAHHAHBABBHHAHBAHHHHHBHAABHHHAHHAABBAHABBHHABAHHHBAHHHBBAAHHAHBAHBAAHHHHHBBHAAHHABAHAHHHHBHHH

*000759_00029863 HHHHAHHHHHBBBHAHHBBBHHHHHBHHHBHABHBHBABHHHHAHHBHAHBABAHHHBBAAHBBHBHBBHAAHHBAHHAHBHHAHABHAAAAHHBHBAHHBHBABHBHHHHHBBABAHHHBHHHHHBAABBBHHHBBHBHBAAB

*000793_00137658 HHHHABAHAHHHBHAHHBBBBHHHHBHAAHHABHBHBAHHHHBHHHBHAHHABAHHHHBAAHHBABBBHBAAHHBAHHAHHBBABHHBAAHAHBBHBAHBBBHAHHBHHHHHBBABABBABHHBBHHHAHHHHAAHBABHHHHH

*001403_00132984 HHBBHHAABBHBHHHHBBAHHHHHHHHHAABHHHBHHHHBAAHAHBBHBABHBHHBHHBBHHAAHBBBBBAABBAAABBHAHBHHHAHHHHHHAAHBHAHHABHAAHAABBHBHAAHHHHBHHHBHHHABAHHAHHBHHBABHB

*001420_00044983 BHHAAHHAABAHAHBHBBBHHBHHHBAHHHHAHBHHABBHHBBBHABBAHHABABHHAAHAABHHHHAHHHHBBAHHBHHHABHABHBBHAHHABBAHAHHHHHBAHAHBHBHHHHHHHHHBHHAHAHHHBBBABABBAHHABB

*002472_00044994 HHAAHAHBBBAHHBABHHABAABHHBHHHHHHAHAHHBHBBHBHHHHAHHABHHBHHHAAHABABABHHHBBHAAHAHHBAAHHBHAHBHAHHHHAHAHBHHAAHAAHAAHHHABAHHABBBHAHBAHHHHHHBAHHHBBABBH

*002792_00087911 HHAAHHHBBBABABABHHABAHBAHHHHHHHHAHAHHBHBBHBHHAHAHHABHHBHHHAAHABAHABHHHBBHAAHAHHBAAHHHHAHBAAHHHHAHAHBHHAAHAHHAAHAHABHHHABBBHAHBHBHHHHHBAHHBBBABBH

*008832_00005288 BHHAHHAAAHBABAHBBHHHHHHHHABAHAAHBBHBHHHBHHABHBBAAAHHHHAABHHBHHHHHHBHHAHAAHHHHBHAHAHBHHBAAHBBBHAHABHHBBABBHAHABABHABHAAHHHBBHHAHAHHBHBHAHHHAHBAHB

*015215_00000473 HHHHHHHHHAHHAHHBAHAHHHABBBBHAAHAAHBBHHHHHHABBHHHBHBAHBAABHBBAHBBHHBHHAABHBABHHHHBHBHAAHAABHAHHAHAAHHHHHHAHAHHAHBBAAHHHHABABHAHHBBBAAHABABABHHHHH

*000000_00580896 HAHHHHHBBBHBABHBBBHBHHHHHHBBAAABHBHHBHAAHBHHHBHHAAHAHBBAAHHHBBBBAABBAAHBHAHH-AHHHABHAHHHHAAHHHBAHBBBAABAHAAHHBHBHHAAHHHHHHAHHHBBHHHHHHBBHBBHHBHA

*000072_00624799 HBAHHBHBAAAHBABBHHHHBBHHHHBHAHHHAHHBHBABHBBAHAAAHAAHBHAAHHHHHBHAHHAAHHHHHBHAAHHHBABHHAHHAHBBABBHHHHAHHHAAH-BBHBBHBAHHHBAHHHBHHHBAAAHAHABHBHBHBBA

*000292_00213662 BBAHBHBHHHBAHAHBHBAAABHBAABHHHBHABABHHAAHHHBBBBAAABHBAHAHAHHHBHBHHHHHHHAHHHHAHBABHBHHAHHHBAHB-AAHHAHHHABAHAHABHHHAHHBHHHHAHAAHBBHHABHAAHHHAAHHBB

*000405_00374498 BAAHAHAHBAHHAAAAHHAABAHHHHAHHHHAHBBHABHBHAHAHAABHHHHABABHBHBABHBABHBHAHHBHHHAAHHABBHAAHHBBHBHAHHHABHBHHAHBHHHABHHAHAAHHBHAHB-AAHHBHHBAHBHBHHHHHB

*000612_00251804 BAHBAHAHHHAHHHHABAABABBHBHHAHHHAHBHABABHBH-AHHBHAHHHAAAHBABAAHHHHHBBAABAHHAHABHAHAHBAHAHBBAHAHHHBBHAABHHBBHHHAHHHBHBHBHHHBBAHHHHHHBHHBHBHHHHABAA

*000855_00015405 AHHBHBBHHHHBBHAHAHHAHHBBBAHHHAABBBBBHHAHBBAHBHHHBABHBHBAHHHBBBBHABAH-HBAAHBBBBHHBHHHAHHHHAHHHAHAHHHAHBHBHAAHHABAHHHABABBBBHHHHBHBHHHAHHHHAAHHAHA

*000867_00250533 HAHBAAAHHAAAHHHAHHHBBHHBBHHHHABBAHHBAHHHAHBABBHAHBHHBAAHHHBHBHBHHAAHAHABAHAHAHABHAAHBHBHHAAHA-HHHAHHHABHHHBHBBHAHHAAHBAHBAHBBBHHHHHHAHAHHHHABBAB

*001087_00208459 BBAHBHBHHHBAHAHBHBAAABHBAABHHHBHABABHHAAHHHBBBBAAABHBAHAHAHHHBHBHHHHHHHAHHHHAHBABHBHHAHHHBAHBBAAHHAHHHABAH-HABHHHAHHBHHHHAHAAHBBHHABHAAHHHAAHHBB

*001541_00007183 AHHHABAHAHHHBHHABHBBBHHHHBHAAHHABHBHBAHBHHBHHHBHAHHABAHHHHBAAHHBHBBBHBAABHHAHHAHHBBHBHHBHAHAHBBHBAHBBBHAAHHHHHHHBAABABBABHHBBHHBAHAHHAAHB-BHHHHH

*003729_00062348 BBHABAHHAABBHHBBB-AHBHBHBAAHBHBBHAHBAHBHBHABHBHAHAAABAABHHAABBBAHHHHBHHHHBBHHHHHAAHAHHHAHHAHHBAHBAAHHBBHBHAHAHHBBHHAHHAHHHHBAHHHHHBHBAHAHHHHHBHA

*000024_00792183 HHHAHHBAHBH-AHHAHHHAHHAAABAAHHHHBHHHHBBBAAHHAHHBAHBHHA-BHBBAHAAAHHHAAHAHHHBBAHBBHHHHAHHAHHHAAHAHABHABHHHHHAHAHHBBBABBHHBBHHHBHAABBHBABHBHHHBHABB

*000347_00063996 BHHHHAHABBHBAABHBBHBHHBHHHAHHAAAAHABHBBHHHHHHHBHHAHBBAHBHAHBAHHABHHA-AHHBAHHABBHAAAHHHHBBHAHBAHBBAAABHBBHA-AHHHHHHAAHAABHAHHAHABHHHHBAHHHAAHHAHH

*000529_00182247 BHBHBAHHBBHHHAHHHHAHAHHAAHHAABABBHHHHHHHBBHBAAAHAHHAHAAAHHHHBHBHBHHBHBHAAHAB-BHHHBABAHHHHAAHBAHBBABABHHBHHAHHBBAHHH-HBAHBAHAHHBABBHAHAAAHHAHHHBH

*004973_00017256 HHBAHAHHHBHBBABHAHABAHHHHHAAHHABHBHHBHBHHBHHHAABAHAAHHAAHBAHBHBBBHHB-BAHHHHBBBBHHHHHHHHBAHAHBHAHHHBAAHABHBHHHBBAHBABHBAAHHHAHAHHBBHAHAABH-AHHHHA

*000969_00157255 BBHABBAHAHHBAHAAHHHHHHHHHAHBAHHHHHHABBHHHBHBHHAABHHHHAABBAAAHBAHHHHHHBBABBBHAHBBHABBBBHHHBHBBHAAHHBAHHAHHB-ABBHHBA--AHBHBBBHAHHAHAHBHAAHHAAHHHHH

*001478_00192770 AB-HAHHHA-BAHAAHHHBBBBABAHBBBAHAHHAAHBHHHHHBBBHHAHABHHHBAAAAAHHHHHABBHBAHHAH-HHAHHBHHHHHBHHHHAAAABHHABAHBABHBHBAHHHHH-HBHBABBAHAHHHHBBHHBHAAHHHA

*001736_00161075 HBHHAAHHBBHHABHHABABBAAHHAHBAHBHHBHHAAHHHBAHHABHBHBBHBHHHBHAHAHAHAHA-AHBAHHHHAHHH-BHHAHHHBBHBABHAHBBHHAHABHHHBAABHAAHBABHHHBHHBHHH-HBABHHBBHHAAB

*000178_00008625 BHBHBAHHBHH-AHBAHHAHAHHAA-HBAHAHBAHAHABHBHHBAAAHAHHAAHAAHHHHHABABHHB-BHHAHABABHHHHAB-HHHHAAHHAHBBABABHBBHHAHHBBAHHHBHBAHBHHAHHBABBHHHAHAHHAHHHBH

*000336_00441640 BHBABAHHBHB-HHHHHHAHAHHAABHBAAAHBAHAHHHHAHBBAAHHAHHAAHHAAHHAHABABHHBBHHHAHAB-BHHHAABAAHHHHAHH-HBBABAHHHBHH-HHBBAHHBHHBAHBAHABBHABBHHHAHAHHAHHHBB

*001501_00086602 BHBHBAHHBBHHHAHHHHAHAHBAAHHAABABBHHHHHHHBBHBAAAHAHHAHAAAHHHHBHBHBHHBHBHAAHABABHHHHABAHHHHAAHBHHBBABAHHABHH-HHBBAHHHB-BAHBHHA-HBABBHAHAAAH-AHHHBH

*000614_00242005 AHBAHAHHH-HHBABAAHABAHHHH-AHHHAHHBHHBHBHHBHHHAABAHAAHHA-HBAHHHBBBHHBHBAHHBHBBHBBHHHHHBHBAHAHB-AHHHBAAHABHBHHHBBAHBABHBAAAHHABHHHB-HAHAABHHABHHHA

*002908_00074035 HBHHAAHHBBH--BHAABABBAHHHHBBAHBHHBHHAAHHHBAHHAHHHHBBHBHBHBHAHHAAHAHA-ABHBHHHBAHHH-BHBAHHAHHABAHHAHHBHHAHHBHHHBBABHAAHHABBBABBHBHHBHHHABBHHHBHAA-

*005326_00043297 BBHABBAHABHAAH-HHHHAH-HHHAHBAHHHHHHAHBHH-BBBHHAAHHAHHAA-BAAAHBHHHBHHHBB-BHBB-HBBHABB-HHHHBHBBHAAHHBAHHAHHHBABBHHBAHBABBHBBBHAHHABABHHAHBHAAHHHHH

*001945_00054359 AAHHHHHHBBH-ABHHBBHBHHBABHBHAAHBBBABBHAAHBBBABHAHAHHHHHHHHHBBBBBHAHHAAHBHABH-AHBA-BHHHHH-AABHHBHBBBHAABHBHHHHBHBHB--HAHHHHHBHHHHBHBHB-BBH-BBHBHH

*000152_00488578 ABHAHHAHBHH--A-AAHHAHHAHB-ABBHHHHHBAHHBA-AHBHBHHAHHBHHHHHAHAHHHABBHHABAHAHBH-HHAHABB-HB-HHAHB-BHBHBBABAHHBBAHAHHBHHBAHHAHBAHAAAHH-HHBBHBHHBBBAHB

*000805_00041050 HBHAHHABAAB-AHHBHHHAHHBAH-HHHHHHH-HAHAHHBBAHAHAHHHABBHHBBAAAHHHH-AA-HBBABAHBHHHBHAAB-AA-HBHBHHAAHHHA-HAHHA-AHBBAAAHHABHHB-BA-HAABHHHHABHH-AA-HHH

*000040_00575345 ABHA-HAHHHA-HBAHHBBHABBHHBAABAHB-A-HAHHHHHHABHHBHAHHBBHHABABBBBHHBHB-HAHBBH--H--H--HAHH---HABHAHAABHHAHHHAAHBHHAHBB-AHBBHHA-HHHHBHHHBHHBHBBBBAHB

*000307_00479106 BBHABBAHAHHH-HAAHH-AH-HHH-HBAHHHHBHAH-HHHBHBHHAAHHAHHAABBAAA-BHHHBH-HB-ABBBB-H-BHABB-HHAHBHBBHAAHHBAHHAHHHBABBAHBAHB-HBHBBBHAHHA---HHAHBH-AHHH-B

*009742_00002625 HBHHH-AAHHHA-HHHBH-BBBAHABHHAAHHBHBHBBAB-A-AHHBHHHHHBHHBHHBHHHA-HBBHHBAABHAH-HHBA-BHBBA-HHHHH--HHAABB-HHAAB-AHBHBHA-AB-HHBHB-HHBAHAHHAHABAHBAH-B

*000825_00179017 HHBHHBBH-BB-BHBHHABHHBAHH-HHHBHHBHAB-AAB-HBAHHBHAHA-HBABHAAHHAHHBHAHHHBBBHHAAHHHB-BAHBHBHBBAH-HHH-HHHH-AHA-HBHHBBHA-BHBH---H-B-B---HBABABBH-HBH-

*000010_00256959 AHBAHHHAAHHHABHHHBBBHHBHBHHBHAAHBBHHHABHHHHBHHHAHBHBHHBHBBBHHHABHHBHHBHHBAHBHBHBBHAHHAAAHAHBHBBHAAHBHBBHHBHBHHAHHABHHAHHBABHHHHBBBHBBBAAHBABAAHB

*000010_00479212 AHBAHHHAAHHHABHHHBBBHHBHBHHBHAAHBBHAHABHHHHBHHHAHBHBHHBHBBBHHHABHHHHHBHHBAHBHBHBBHAHHAAAHAHBHBBHAAHBHBBHHBHBHHAHHABHHAHHBABHHHHBBBHBBBAAHBABAAHB

*000010_00584698 AHBAHHHAAHHHABHHHBBBHHBHBHHBHAAHBBHAHABHHHHBHHHAHBHBHHBHBBBHHHABHHHHHBHHBAHBHBHBBHAHHAAAHAHBHBBHAAHBHBBHHBHBHHAHHABHHAHHBABHHHHBBBHBBBAAHBABAAHB

*000072_00542472 HBAHHBHBAAAHHABBHHHHBBHHHHBHAAHHAHHBHBABHBBAHAAAHAAHBHAAHHHHHBBAHHAAAHHHHBHAAHHHBABHHHHHAHBHABBHHHHAHHHAAHABBHBBHBAAHHBAHHHBHHHBAAAHHHABHBHBHBBA

*000121_00042157 HAHBAHHAAHHAABHHHHHBBBBABHABHAHBBBBHAAHHHHAHAAAAHHHBBBHBBHBHBHBHAABHHABAAABHBAHHHBHABAHHBAAAHBHHHHHHHAHHHHHHHHAHHBBBBHHAHABBAHHAHHHABHAHHHBHHAHH

*000198_00449581 BBHABBAHAHHHAHAHHHHHHHHHHAHBAHHHHHHAHBHHHBHBHHAAHHAHHAABBAAAHBHHHBHHBBBABHBBAHBBHABBBHHHHBHBBAAAHHBAHHAHHHBABBHHBAABAHBHBBBHAHHABAHBHAHBHAAHHHHB

*000257_00304834 AHABHAHHAHHBBAHHBHBBHHHAABHBHHHHHBHHBBHBHAAAABHAHBAHHHABHBHBHAABAHBHHHAABABAHAAHHHHHHBAHHBBAAAHHAAHHHAABBBHHHAHHHHHHHAAHHHHHHBAHABBHHHAAHAHHAAAA

*000319_00420413 AHBBABHABHABAHHAHAHHHHHBHHABAHHHBBHHHHHHBHHBBAHBHBHAHBHHBHHHHAHHAHBBHHHHHHHHHAAHHHHBABBBBHAHHABHABHHBAHAAAAABHBBHBHAHABHHABHBHHBHBABHHBHBBHBBAAB

*000400_00232396 HBHHAABHBBHHABHHABABBHAHBHBBAHHHHBHHAAHHHBAHHABHBHBHHBHHHBHAHAHAHAHAHAHHAHHHHAHHHHHHBABHHBBHHABHAHBBHHAHABHHHBAABHAABBABBHHBHHBHHHAHBABHHHBBBAAB

*000408_00066711 AHHBAHBBAHHAHBAAHBBHHHBHABBHHHABHABHHHABHBBHHHHHAAAABHHAHHHHBAAABHBHAAHAHAHBAHHHHAABAABHHHHAABHHBHBBHHHHHHAHBBBHBHABBAHABHHHHBAHBBHHAHHBHHHHBAHH

*000435_00188728 BBHABAHHAAHHHHBBBAAHBHBHBAAHBHBBHHHBABBHHHABHBHAHAAAHAABHHAABBBAHHHABHHHHBBHHHAHAAHABHHAHBAHHBAHBAAHHBBHAHAHAHHBBHHAHHAHHHHBAHHHHHAHBAHAHHHBHBHH

*000490_00268271 BAHBAHABHHAHHHHHBHABABBHBHHAHHHAHBHABABHBHHAHHBHAHHHAAAHBABAAHHHHHBBAABAHHAHABHAHHHBAHAHBBAHAHHHBBHAABHHBHAHHAHHHBHBABAHHBBAHHHBHHBHBBHHHHHHABAA

*000492_00112357 HBAHHBBHAAAHHAHBHHHHBBHHBHBHAHHHABBBHBABHBBAHAAHHHHHHAAABBHHHBHHHBAHHHHHBHHHAHBABABHHAHHAHBBABBHHHBAHHHHAHABBHHBHHAHBHBABHBBHHHBHAAHAAABHBHHHBBA

*000493_00210950 AHHBAHBBAHHABBAAHHBHHHBHABBBBHABHABHHHABHBBHHHHHAAAABHHHHBHHBAAABHBBHAHAHAHBAHHHHAHBBAHHHHHAAHHHBHHBHHHHHBBHBBBHBHABBAHAHHHHBBAABBHHHHHAHHHBHAHA

*000545_00097280 BBHHBHAHBHHAHBHBHBHHAAHBAHHHBABAABHHHHAHHBHHBBHAAABBBAHAAABHABHBHBHHHBHABHHHABHAHHHBHAABHHABBBHAHHHAHHABHHAHABAHHHHBHHHHBHAAHBHHHHHHBHAAHHAAABBB

*000575_00044431 AHHHABAHAHHABHHABHBBBHHHHBHAAHHABHBHBAHBHHBAHHBHAHHABAHHHHBAAHHBHBBBHBAABHHAHHAHHBBHHHHBHAHAABBHBAHHBBBAAHHHHHHHBAHBABBABHHBBHHBAHAHHAAHBHBHHHHH

*000585_00079278 AHHBHBBHHHHBBBAHAHHAHHBBBAHHHAABBBBBHHAHBBHHBHHHBABHBHBAHHHBBBBBABAHBABAAHBBBBHBBBHHAHHHHAHHHAHAHHHAHBHBHAAHHHBAHHHAHABBBBHHHHBHBHHHAHHHHAHHHAHH

*000586_00130566 HBHBHBHBAHBHBBBBHBABHHBHAHAHHAHHHHHHHAHAAHAHHHHHHHABHBBBHBBBHAAHHAABHHBHBABHAAHHBAHBBAHABHHHAHBAHBABHBHHAHBHHHBAHAHBHABHHAHHHHABHHABHHHBHAABHAHB

*000602_00194665 BAAHHAHHHHBHHAAHAAABBHHHHHABHHHHBHBBAHHBBAHAHAHBHHBBHHHHHBHHHAAHAHAHBHBABHHBAAAHHBHBAAHBABHBBAHHHAHABHHAHHAHHABHHHHHHAABABHBAABHBBHABAABHHHHHHBB

*000607_00003474 AHBAHABHHBHHBABAAHABAHHHHHAHHHAHHBHHBHBHHBHHHAHBAHAAHHAAHBAHHHBBBHHBHBAHBBHBBHBBHHHHHHHBAHAHBHAHHHBAAHABHBHHHBBAHBABBHAAAHHAHAHHBBHAHAABHHABHHHA

*000720_00039146 AHHBBABBAHHHHAAAHHBHBAHAHHAHHABHHHHHHAHBAABAAHBHAHHABABBHHHAHBHBHABHHAAHHHAHAHHHHAAHBHABAHHAABHAHHBHHBBHHHBAHBHHHHAABHAHBAHBHBAHHHHBBHAAHBHHABHB

*000742_00117661 AHHHABHAAHHBABHHHBBBHHHHHHABHAAHBBHAHAHAAHHHHAHAHHHBHHBBHHBHHHABHHAHHBBHBABHHHHBBHHHAAAABAHBHBBHAHHBHBHHHBBBHBBABABHHABHBHHHHHHBHBHBHBAAHBABAAHB

*000786_00314595 BBHAHHABAABHBBBHBBHHAABAABHHBHBHBBAAABBBHBAHAAHHAAAABABBHHAHHHHHHAHAHHHHBBBHHHAHAHHABAHHHHAHHAAHHHAHAHHHBBAHAAHHAABHAHHHHHHAHHHHHHBHHABAHHBHHBHA

*000873_00106935 HAHBAAAHHAAAHHHAHHHBBAHBBBHHHABBAHHBAHHHAHBABBHAHBHHBAAHHHBABHBHHAAHAHABAHAHAHABHAAHBHBHHAAAABHHHHHAHHBHHHBHHBHHHHAAAHAHHAHBBBHHHHHHAHAHHHHABBAB

*000909_00045849 HAABAHHHHAHBAHAHHBABBAAHABHHHHAAHBHHHBHBHAAHHAHBHHHHABABHHHHABHHHHHBBAHHBHHAAAHAHBBBAHAHHBBBHAAHHHBHBABABHHHBAHHAAHAHHHHAHHBAAAHABBHHAHBHHHHAHBB

*001099_00087957 AHHBHABBAAHHHAAAHHBHBAHAHAAHHABHHHHHHAHHAABAAHHHAHHHBABBHHHAHBHBHABHAHAHAHAHAHAHHAHHBHHHAHHAABBAHABHHBBHHHBAHBHHHHAABHAHBAHBHBAHHHBBBHAAHBHHABHB

*001174_00013265 HBHHHBHBAAAHBHBBBAHHBBHHAABHHAHBAHHBBHAHHBHHHAAAHAAHBHAAHHHHBBBHHHAHAHHHHBHAAAHHBHBHHBHBHHBAABHHHHHAHHHAAAABHHHBHBABHHBAHHHBBHHBAAAHBHABHBHBHBHA

*001174_00155674 HBHHHBHBAAAHBHBBBAHHBBHHAABHHAHBAHHBBHAHHBHHHAAAHAAHBHAAHHHHBBBHHHAHAHHBHBHAAAHHBHBHHBHBHHBAABHHHHHAHHHAAAABHHHBHBABHHBAHHHBBHHBAAHHBHABHBHBHBHA

*001203_00543107 BBAHBHBHHHBAHAHBHBAAABHBAABHHHBHABABHHAAHHHBBBBAAABHBAHAHAHHHBHBHHHHHHHAHHHHAHBABHBHHAHHHBAHHBAAHHAHHHABAHAHABHHHAHHBHHHHAHAAHBBHHABHAAHHBAAHHBB

*001392_00039727 AHHBAHABAHHAHBAAHBBHHHBHABBHHBABHABHHHABHBBHHHHHAAAABHHAHHHHBAAABHBHAAHAHAHBAHHHHAHBHABHHHHAABHHBHBBHHHHAHAHBBBHBHABHAHABHHHHBAHBBAHAHHBHHHHBAHH

*001446_00009270 HAHBAAAHHAAAHHHAHHHBBAHBBBHHHABBAHHBAHHHAHBABBHAHBHHBAAHHHBHBHBHHAAHAHABAHAHAHABHAAHBHBHHAAAABHHHAHAHHBHHHBHHBHHHHAAABHHHAHBBBHHHHHHAHAHHHHABBAB

*001529_00222254 AHHHABAHAHHHBHHABHBBBHHHHBHAAHHABHBHBAHBHHBHHHBHAHHABAHHHHBAAHHBHBBBHBAABHHAHHAHHBBHBHHBHAHAHBBHBAHBBBHAAHBHHHHHBAABHBBABHHBBHHBAHAHHAAHBBBHHHHH

*001869_00024794 HBAHAHHAHBHHHHHHBBABHHHHBHHHAAHAAAAABAAAABHAHHBHHHAHAAHHBHBHAAHBHHHAHABHBHBHABHHBHHAAHHHBHHHBBBHHBHHBAHHBBABBAABBHHBHHHHABBHABHBHABHHHAHHHBHHBAA

*002226_00001703 HBBHHBHHAHAHBHBHBHBHHBHHAABAHHHBAHHBBHAAHBHHHABAHAAHBHAHAHHABBBHHHAHAHBHHBHAAAHHBHBHHBHBHHBAABHHHHHHHHHAAAHBHHHBABABHHBAHHAHBHHBAAAHBHABBBHBHBHA

*002265_00133029 BBHABAHHAABHHHBBBAAHHHBHBAAHBHBBHAHBABBHBHABHBHAHAAABAABHHAABBBAHHHHBHHHHBBHHHHHAAHAHAHAHHAHHBAHBAAHHBBHBHAHAHHBBHHAHHAHHHHHAHHHHHBHBAHAHHHBHBHA

*005032_00018209 HBHHHBHBAAAHBHBBBABHBBHHAABHHHHBAHHBBHAHHBHHHAHAHAAHBHAAAHHHBBBHHHAHAHHHHBHAAAHHBHBHHBHBHHBAABHHHHHHHHHAAHABHHHBABABHHBAHHABBHHBAAAHBHABHBHBHBHA

*005592_00057712 BBAHBHBHHHBAHAHBHBAAAHHBAABHHABHABABHHAAHHHBBBBAAABHBAHAAAHHHBHBHHHHHHHAHHHHAHBABHBHHAHHHBABBBHAHHHAHHABAHABHBHHHHHHBHHHHAAAAHBHHHHBHAAHHBAAHHBB

*000631_00040605 ABBHBBBHHAHHBHHBHAHAABHBHBABBABAAHHHHHAHHBHBBAHAABHHAABBAAHHAHBHAHAHHBAHHHHAHBHHHBHBHBHHHHBAHHHBHBHBBHAABHAHHHBHHHHABHBHAHAA-AHAAHBHBAHHHBHAHBHH

*000047_00010363 BHHAHHAAAHBABAHHBHHHHHHHHABAHAAHBHHBAHHBHHABHBBAAAAHHHAABHHBHHHHHHBHHAHAAHHHHBHAHAHBHHBAAHBBBHAHABHHBBABBHAHAHHBHABHAAHHHBBHHAHAHHBHBHAAH-AABAHB

*000332_00084970 HHHAHAAHBHHHHAHHABHHHAAHHHAHAAHAHABBHBBAHHHBHHABHHBHBHHHBHHAAHBHHBBAHHHHAHBABBAHHHBBHAHA-HAHHABAHABHHBHHHBBABAHHBHBBAHAABBBHAHHBHBHHHHBHBABBHHAB

*000771_00183281 HHHHAHAHHHABHBHABAHBHHHHHAHBHAHAHABHHABHBHAHHAHHAAABAAHHBBBAAHHHHABB-ABAHBAHAHAAHAHHHAABBBAHHAHHBBHHABHHHHBBBAAHHHHHAHHAHHBHHBABHBHHHBHBHHAHABHA

*001071_00024309 HHHHHHHHHAHBAHHBAHAHHHABBBBHAAHAAHBBHHHHHHABBHHHBHBAHBAABHBBAHBBHHBH-AABHBABHHHHBHBHHAHAABHAHHAHAAHHHHHHAHAHHAHBBAAHHHHABABHAHHBBBAAHABABABHHHHH

*001223_00156772 HHHHAHAHHHABHBHABAHBHHHHHHHBHAHAHABHHABHBHAHHAHHAAABAAHHBBBAAHHHHABBBABAHBAHAHHAHAHHHAABBBAAHAHHBBHHABHHHHBBBAAHHHHHABHAHHBHHBABHBHHHBHBH-AHAHHA

*002051_00120592 HHHAHAABHHHBHBHHBAHHHHHHHHHHHAHAHABHHABHBHAHHHHHAAABAABABBBAABHHHABBHABHHBAHAH-HHHAHHAHBBBAHHAHABBHAHBHHHHHBBAAHAHHHABAAAABAAHBBHHHHABHBHHAHHHBA

*007636_00006593 HHHHHHHHHAHHAHHBAHAHHHABBBBHAAHAAHBBHHHHHHABBHHHBHBAHBAABHBBAHBBHHBH-AABHBABAHHHBHBHHAHAABHAHHAHAHHHHHHHAHAHHAHBBAAAHHBABABHHHHBHBAAHABABABHHHHB

*000538_00241307 AHBAHABAHBBAHHBAAHABAHHHHHHHHHHHHHHHBHBHHBHHHHHBAAAAHHAABHHHHHHBBHHBHBAHBHBBBHBHHBHHHHHHABAAHHAHHHBHAHABABBHHHBABBABBBAAAHBABAHBH-AAHABBH-AHHHHA

*000567_00036151 -AHHHHHHBBHHABHBHBABHBHHH-HHAHABABHHBHAAHBHHHHAHHAHAABBAAHHHBBBBAABBAHHBHAHHAAHABABHBHHHHAAHHBBAHHBBAABAAA-HABHABHAAHHHBHHHHHHBBHHAHAHHABBHHHHHH

*000967_00223997 BAAHAHAHHAHAAAAAHHAABAHHHAAHHHHAHBBH-BHBHAHAHAABHHHHHBABHBBBABHBABHBHAHHBHHH-AHHABBHAHHBBBHBHAHHHABHBHHAHBAHHABHHAHHABHBHHHBAAAHHBHHBAHBHHHHBHHB

*000984_00204914 BAAHHAHHHHBAHAAHAAHBBHHHAHABHHHHBHBBAHHBHABAHAHBHHBBHHHHHBHHHAAHAHAHBHBABHHBAAABHBHBAAHBABHBBAHHHAHABHHAHHAHHABHHHHHHA-BHBHBAABHB-HABAABHHHHHHBB

*006624_00008401 AHHBABBHAHAABAHHHHHBHAHBHABHHAHABBHHBHHHBHBHBHHHAHAAHBHBHHHHAHHBHABB-BBAHHAH-ABABHBHAHBAHHAHHAAAHHAHHHABBHAHBAHHAHHAHBBBHHHAAAHHHBBABHAHBHHHBHHA

*000023_00386496 ABAAHHBHAHB-ABHHHHHHBHAAAHAHAHBBHHAAHHBHAHHHAHHBBHHHHHABHBBHBAHAHBHHABHHAHBBHHBBHHHBAHHAAHBAAHAAAHAHBABAHHHHBHHBBAABBBHBBHHBHHAAB-HHBHHBH-HHHABH

*000025_00196906 ABHHHHAHBHHAAHBH-HHHHHABA-BHABHHHHHBHHAABABHBHHBAHHHHBHAHBHBHHHBHHHBHBHHHHHAAHAHHHBAABHAHHHBABHBAABAABHHHAABAAHHABAAAAHABAHHBHHAAHHAH-BAAAHHHABA

*000632_00292308 ABHBHHBHBAABHH-BBABAHAHAAHAHBBBBAHBHAHAHHAHBAHHBHBBAHHHAAHBBBHHAAHHHAAHAAHBHAHHHBAAHBAHHAHHAHHBBBHBHHHHAHBBHHHAHHHHHBHHAHHAHABHHH-HAHAHHH-AAHAAB

*000047_00259538 BHHAHHAAAHBABAHHBHHHHHHHHABAHAAHBHHBAHHBHHABHBBAAHAHHHAABBHBHHHBHHBH-AHAAHHHABHAHAHBHHBAAHBBBHAHABHHBBHBBH-HAHHBHABHAHHHHBBHBAHAHHBHBHAAH-HABAHB

*001202_00071754 HHHBABBHAHAAHAHHHHHBHAHBHABHHAHABAHHBHHHBHBHBHHHAHAAHBHBHHHAAHHBHABBBBBAHHAAAABABHBHHHBA-HAHHAAAAHAHHHABBHAHBAHHAHHABHBHHHHHBAHHHB-ABHAHB-HHBHHA

*007905_00005206 HAHAHHBHHHHAAHBAHHHAHHAAA-AAAHBHBHHHHBBHAABHAHHBHHBHHAA-HBBAHAHAHHHAAHAHHHBBAHBBHHHBAAHAHHHAA-AAABHABHHHHHHHAHHBBBABBHHBBHHH-HAABBHBAHHBHHHBHABB

*000141_00087427 HBHAAABHBHH-ABH-HBAHAAAHBHHBAAAHBBHHAAHHHBAHHABHHHHHHBHHHHHAHAHAAHHAHAHHHHHH-AHHHHBH-ABHHHBHHABHABBBABAHABHBHBHABHAA-BABHHHAHHHHHAAHBABHA-BHBAAB

*000447_00159322 HBHHAAHHBBHHABHAABABBHAHHHBBAHBHHBHHAAHHHBAHHABHBHBBHBHHHBHAHHHAHAHABA-HAHHHHAHAHHHH-AHHHHBABAHHAHBBHAAHA-BHHBBABHAAHBA-BBHBHHBHHAAHH-BHH-HBHAAB

*001197_00006651 ABABBHHHAHB-AHBHHAHHBHHHAAAHHHBHH-HAH--BAH-HHHAHBAHHBAABHBHHHAHBABHH-B--HHHBHH-HAAHBBBHAAHHAAAHHHAHHBAHAHHHBABHBBAAB-HH-BHBBBAHHBHBHBHHAHAB--HBB

*000087_00387320 BBHHH-AH-AAHBH-AHHBAABHBBABHHBAHBAHHHAHAHHHHBAAHAAHBHHHHAHBHHABAHAHH-ABHBAAHAHAHAA-B-AHHH-HAAHAHAHHHHA-HHH-AHAHAHHH-ABBBBHAAHHHBH-BBH-HBH-AB-HBB

*008807_00002173 HHHB-BBHHAHBBHAHAHHAHH-BBAHHHAAB---BHHAHBBHH-HHHBABHBHBAHHH-B-BHABAH--BAHHB--BHBB-AHAHB--HAHHAHAHHHAHBHBHAAHHABAHH--BABBHBHBHHBHB-HH-HBBHAHHBAHH

*000805_00131985 --HHHBABA-B--HHHHHHABHBAH-HBHHHHHHHAAAHHHBHHAHAHHHABB-H-BH-AHHHHBHAB-B--BAHBHH-B-AHBBAAAHBBBHBA-HHHABHAHHH-AHBBAAAHA-BBBBHBHAHA-BHHHH-B-H-AAABHH

*000991_00290005 AHBBAHHABBA-HH--HHHHAAA-HAAHH-AHHAHHHA-AAA-AHHAHHH-AAHH-AAHHHAHHAAHH-A-AAAAHHHHAAHHA-AHHBHAHHHA-AAHAAHHHA-AHAH-A-B-AABAHA-HHAHAHA-H-HHHBAHAAAHBH

*000045_00659062 -HHHBHAHAAHAHH-HHBAHABHAA-HAHBBHHHBAHH-A-B-AHHHHHHHHBAA-A-HHHABHHBHH-H-HAHHB-HAAA-BH-BAHAHABH-BBHABHHBAAHH--BH-ABHA-HB--BBHAHAAH--BBHABHBAHHAHHA

*009350_00002031 HHAABAHAABHBAH-HAHBBB-H-H-AB-AHHHABH-HHB-AHHHH-BBHHHBHB-HAAHHABHBBBABH-HHBHA-HB-H--B-B-HH-HHA-HB-AHHAH-HB---HBBHBH-B-AAHHAHB-HBH--HHB-BHH-A-BHHB

*000131_00037243 ABHHABHAAHHHABHHHBBBHHHHHHABHAAHBBHAHAHAAHHHHAHAHHHBHHBBHBBHHHABHHAHHBBHBABHHHHBBAHHAAAABAHBHBBHAHHBHBHHHBBBHBBABABHBABHBHHHHHHBHHHBHBAHHBABAAHB

*000531_00024271 AABHAHHHABHHABHBHBHHBHBHAHAHHBHHAHBABHHBHBBAHHHAHAAHBHHHHHAHHBBBAHAHBBHBAHHBHBHBHHHBBBBHABHBHAHBABHBBAABHHABHHHHABHHHHHBHAHBBAAHBBHBHHBBBHHHBAHH

*001480_00008481 ABHHHAAHHHAHAHHBBBHHHBABHHBHABHHHHHBHHAABHBHBHHBAAAHHBHAHBBBHAHHHHAHHHAHBHHAHHAAHABAABHAHHBBHHABAHBHABBHHHHHAAHAABAHAAAAHHHHBHHAAHHAHHBAAHBBHABA

*001830_00034815 ABHAHHABBHHHHHHAHHBAHHHHHBHBBBHHHBHAHHHAAAHBHBHBHAHHHAHAHAHAHHHBBHHHABHHBABBBHHHBHHAHABHHHAHHHBBBABBHHAHBBBHAAHHBHABHAHAHHHHAAABBBBHBABBHHBBBABB

*006399_00014728 ABHHBBBHHAHHBHHBHAHAABHBHBABBABAAHHHHHAHHBHBBAHAABHHAABBAAAHAHBHAHAHHBAHHHHAHBHHHBHBHBHHHHBAHHHBHBHBBHAABAAHHHBHHAHAHHBHAHAAHAHAAHBHBAHHHBBHHBHH

*010223_00000859 ABHBHHBHBHABHBHHBHHAHAHAAAABBBHHHBHHAAHHBHABABHBHBBAHHHAAHHBHBBAAHHHAAHAAHBHBAHHBHAHHAHHHABHBAHBHBBAHBHAHBBHHHAHHHHBBHHHHHAHAHAHHHHABHBBAHAAHAAH

*000008_00267111 BHHBBHAHBHHABBHBHBHHHAABBHHHBABAABHAHHAHHBHABBAAAABBHHHAHBBAAHHBHBHHHBBHBHHHABHABHHBBHABHHHBHBHBAAHAHHABBHAHABAHHHHBHHHHBHHHHHHHHABHBHAAHHAHABBH

*000067_00192838 HHBHHBHHHHAHBHBHBHHBHBHHAAHAABHHAHHBBHAAHHBHHABHHAAHBHABAHAAHHBHHHAHAHBBHBHAAAHHBHBHHBHBHHBAHBHHHBHHHBHAAABBAHHBABABHHBHHAAHBHHBAAAHBAHABBHHHBHA

*000097_00226706 HHHBAAHHAAAAHHAAHHHBBAHBHBAHHABBAHHBAHHHAHBABBHAHHHHBAAHHHBABBHHHAAHAHABAHAHAHAHHAAHBHBHAAAAABHHBABAAHBHHHBHHBHHHHAAHHAHHAHBHBAHHHHHHHAHHHHHBBAB

*000285_00288163 BHHBHAAABBBHHABHABABBHBHHHAHHHAAHHABBBHHHHHHHHBHHAHBBAHHBHBBAHHHBHAABAHHBAHHABBHHAAHBHHBBHAHBAHABHAAHHBBHHHHBBHHAHAHAHAHHHBBBHABHHHABHHHAAHBHAHA

*000308_00116029 HHHHAABHBHAAHBBHHBHHHHBBHHBHABBBHABBHAAAHHHHBHAHHAAHAHHAHBABBHHHBHAHHHAHBHAABBHAAHBAABBAHHBBHHABAHHHHHBHHBBAAHHAHBAHBHAAHHHHBBHAAHHABAHAHHHHBHHH

*000403_00239589 BHHAAHHAABAHAHBHBBBHHBHHHBAHHHHAHBHHABBHHBBBHABBAHAABABHHAAHAABHHHHAHHHHBBAHHBHHHABHABHBBHAHHABBAHAHHHHHBAHAHBHBHHHHHAHHHBHHAHAHHHBBBHBABBAHHABB

*000461_00018171 HHBBBAHAHHAHHHBHHAHHHHBHHHBAHBBHBHBAAHHHHHBHHHBHBHBBHHABHBBAHHBBBABABHAHHBABBHHBHHHHHAHBHHHABHHBABBAAHABBHHHAAAHHHABBAAHABHHHBHHBHHBBHAABHHAHHBB

*000483_00144617 HHAAHHHHBBAHABABHBABAHBHHAHHHABHAHAHHBHBBHBHHAHAHHAAHHBBHHAAHABAHABHAHHBHAAHAAAHBABHBHAHBAHHHHHAHAHBHAAAHAHHAAHAHABHHHHBBBHAHBHBHHHHABAHHHHBAHHH

*000921_00040828 HHBHHBAHBBHHBBABHBAABBBHHAAHAHHBBHABBABHBHABHBHAAHBBHHHHHAHHBHHAHABBAHAHBAAHHBBAHABHHBBAHHHABHHHAHHHHHAHHHHBAHHHHHHAAHBHHBHBHHHHHAHABBHBBBHHBAHB

*001038_00012733 HHABBHHHHBHABAAHHHBHHHHBBBAABHAHHHHAHABHBHABBABAAAHHBBHHAHAHHBBAHAHHAAHHBHHHHHHBHBAHBBAAHAHHABHABHBHAHBABHBHHHHHHBAHHBHAHBAHAAAHHHBAHHHHHBHHAABB

*001080_00172264 HHHAAHHAABAAAABHBBBHHBHHHBAHHHHAABBHABBHHBHHHABBAHHABABHHAAHAHHHHHAAHHHHBHAHABHHHAHBABHBBHAHBABBHHAHHHHHBAHAHHHBHHHAHAHHHBHHAHAHHHBHBABABBAHHABB

*002231_00030249 HHBBHHAABBHBHHHHBBAHHHHHHHHHAABHHHBHHHHBAAHAHBBHBABHBHHBHHBBHHAAHBBBBBAABBAAABBHAHHHHHAHHHHHAAAHBHAHHABHAAHAABBHBHAAHHHHBHHHBHBHABAHHAHHBHHBABHB

*003576_00000799 BHHBHHHHHBBAHABHHBHAHHABHHBAHBBBHBHHHHAHHBBBBHBAAABAABHAHHHHBHHHHABHBHHAHABHAHBABHBHBAHHBBABBBHABHAHBHABAAAHAHHHAHHBHHHHBHHHBHBBHHABHAHHBHAAHBHH

*004529_00007502 BHHAHHAAAHBABAHHBHHHHHHHHABAHAAHBHHBAHHBHHABHBBAAHAHHHAABBHBHHHBHHBHHAHAAHHHHBHAHAHBHHBAAHBBBHAHABHHBBHBBHAHAHHBHABHAAHHHBBHBAHAHHBHBHAAHHAABAHB

*005462_00039709 BHHBHAAABBBHAABHABABBABHHHAHHAAAHHABBBHHHHHHHHBHHAHBBAHHHAHBAHHABHAABAAHBAHHABBHHAAHAHHBBHAHBAHHBAAAHHBBHHHHHHHHAHAHHAAHHHHBBHABHHHABHAHAAHHHAHH

*011517_00002321 HHHBHABBAAHHHAAAHHBHBAHAHBAHHABHHHHHAAHHAABAABHHAHHHBABBHHHAHBHBHABHHHAHAHAHABAHHAAHBHAHAHHAABBAHABHHBBHHHBAHBHHHHAAHHAHBAHBHBAHHHHBBHAAHBHHABHH

*000177_00466318 HAABAHHHHAHBAHAHHBABBAAAHHHHBHAAHBAHHHHBHHAHAAHBHAHBABABHHHHABHAHHAHHHBHBAHHBABAHHBB-BHHHHBBHHABHHBABABABHABBAHHAAHAHHAHAHHBHHAHHBBAHAHHAHHHHHHB

*000187_00358775 BBHBBHHHHABAHAHBHBAAABABAABHHBBHHBABHHAHHHHBBHBAAABHAHHAHAHHHHHBHAHHABHAHHHHAHBABHBHHAHHHBAHBBHAHHAHBHABAHAHABHHAAHHHHHHBAHAAHBBHHABHAHHH-AAHBHB

*000215_00209045 AHHBHHABAHHABBAAHBBHHHBBABBHHBABHABHHHABHBBHBHHHAAAABHHHHHHHBAAHHHBHAAHAHAHB-HHHHAHBHAHHBHHAABAHBHBBHHHHAHAHBBBHBHABAAHABHHHHBAHBBAHAHHBHBHHHAHH

*000251_00135570 HAHBAAAHHAAAHHHAHHHBBHHBBHHHHABBAHHBAHHHAHBABBHAHBHHBAAHHABHBHBHHAAHAHABAHAH-HABHAAHBHBHHAAHABHHHAHHHABHHHBHBBHAHHAAHBAHBAHBBBHHHHHHAHAHHHHABBAB

*000380_00034348 HBAHHBHBAAABHABBHHHHBBHHHHBHAHHHAHHBHBABHBBAHAAAHAAHBHAAHHHHHBHAHHAA-HHHHBHAAHHHBABHHAHHAHBBABBHHHHAHHHAAHABBHBBHBAHHHBAHHHBHHHBAAAHAHABHBHBHBBA

*000527_00074206 HBAHABHAHBH-HBHHBHABHHHHBBHHAAAHAHAHBAAAABHHHHBHHHBHAAHHBHBHHAHHBHBABABHBHBHAHHHBAHAAHHHBHHHBBBHHBHHBAHHBBABBAABHBHHHHBHABBHABHBHHBBHHAHHHBHHBAA

*000135_00028244 HHHHABABHHHAHHHAHBBAABHBBBBHHBABHABAAAHAHHHHBHAHAAHHHHAHHHHHBAHHHAHHAAHHHAHBHHAHHABBHAHHH-HAAHAHABHHHHHHAHAABHHHHHABAHBABAHAABABBBABAHB-HBAAHAHH

*000572_00206868 HHAAHHHBBBAHHB-BHHABAHBHHBHHHHHHAHAHHBHBBHBHHAHAHHABHHBHHHAAHABAHABHAHBBHAAHAAHBAABHBHAHBAHHHHHAHAHBHHAAHA-HAAHAHABAHHHBBBHAHBHBHHHHHBAHHABBABBH

*000711_00214412 HHABABBBAHAA-AHHHHHBBAHHBAHHAHHABAHHBHAHBHHAHHHABHBAHBHBHHHAAHHHHAHBBBAAHHABAAHABHBHHHBAHHAHHAAAHHAHHHABBH-BHAHHAHHHBABBHHHBHHHHBBBABHABBHHHBHHA

*000992_00266994 HHABHAHBAAAAAHAAHHBBBAHBHBAHHABBAHHHAHHHAHBABBHAAHHHBAAHHHBABBHHHAHHAHAHAHAHAHAHHAAHBHHHAAAAABHHHABAAHBHHH-HHBHHHHAABHAHHAHBBBAHH-HHHHAHHHHHHBAB

*001054_00205447 HHHHAHAHHHABABBABAHBHHHHHHHBHAHAHABHHAHHBHAHHAHHAAABAAHHBBBAAHHHBABBAABAHBAH-HHAHAHHBAABBBAHHAHHBBHHABHHHHHBBAHHHHHHABHAHHBHABABHBHHHBHBH-AHAHHA

*002655_00027452 HHAAHHHBBBAHBBABHHABAHBHHHHHHHHHAHAHHBABBHBHHAHAHHABHHBHHHAAHABAHABHHHBBHAAHAHHBAAHHHHAHBHAHH-HAHAHBHHAAHAHHAAHAHABHHHHBBBHA-BABAHBHHBAHHHBBABBB

*000034_00262466 AHHHABAHAHHHBHHABHBBBHHHHBHAAHHABHBHBAHBAHBHHHBHAHHABAHHHHBAAHHBHBBH-BAABHHAHHAHHBBHHHHBHABAH-BHBAHBBBHAAAHHBHHHBAABABBABHHBBHHBHHAHHAAHB-BHHHHH

*000606_00339364 BBHABAHHAABHBHBBBAAHBHBHBAAHBHBBHAHBAHBHBHABHBHAHAAAHAABHHAABBBAHHHHBHHHHBBBAHHHAAHAHHHA-HAHH-AHBAAHHBBHHHAHAHHBBHHABHAHHHHB-HHHHHHHBAHAHHHBHBHA

*000346_00439891 AAHHHHHHBBHB-BHHBBHBHHBABHBHAAHBHBHBBHAAHBHHABHAHAHHHHHAHHHHBBBBHABAAA-BHAHHBAHHAABHHHHBBAABH-BHBBBHAABABHBHHBHBHBAAHHHHHHHH-HHHHBBHHABBHBBBHBHH

*002609_00147268 AAHBHABBAHHA-AAAHHBHBAHAHHAHHABHHHHHHAHBAABAAHBHAHHAHA-BHHHAHBHBHABHHAHHHHAH-HAHHAAHBHABAHHAABHAHABHHB-HHBBAHBHHHHAABHAHBAHHABAHHHHBBHAAHBHHABHB

*001657_00065233 -HABBHHHHBH-BAAHHHBHHHHBBBAABHAHHHHAHABHBHABBABAAAHHBBHHAHAHHBBAHAHHAAHHBHHHBHHHHBAHBBAAHAHHABHABHBHAHBABHBHHHHHHBAHHBHAHBA-AAAHHHBAH-HHHBHH-ABB

*000031_00071162 HBHBHHBBHHBH-BBBHHHHHHBAAHAHHH-HBHBHHAHHHBBHAHHHHHABHBBBHBABHAABHAAB-HBHBABB-AAHBAHBHAHABBHHHHBAHBAABBHHHH-AAHBAHAHBBAHHHHHHHHABBHHBHAHBHAAHHAHB

*000031_00918886 HBHBHBHBHHBH-B-BHBHBHHBAABAHHHHHHHHHHAHAABHHAHHHHHABHBBBHBABHAAHHAABHHBHBABHHAAHBAHBBAHABBHHHHBAHBAHBBHHAB-AHHBAHAHB-HBHHHHHHHABHAABHAHBH-ABHAHB

*000214_00160648 BAHHAHAHHHAA-HHABAABAHBHHHHAHHHAHHBABABHBHAHHABHAHHHAAAHBABAAHHHHHBB-ABAHBAHABHHHAHBAHABBBAHAHHHBBHAABHHBHAHHAAHHHHBABHHHHBA-BHBHB-HHBHHH-HHAHAA

*002578_00014930 HBAHHHBABBHAHBHHBHHHHHHAHBHBBBAHBBHHAAHAHHABABABHBBHHHAAHBABHBBAAHA-HAHAAHHHBAHHHBABHAHHHHBHHAHHHBBAHBHAH-HHBHAHHHHBHHBHBBH-AABAHHHBBHB-A-HAHAAH

*000097_00164061 HHHBAAHHAAA-HHAAHHHBBAHBH-AHHABBAHHBAHHHAHBABBHAHHHHBAAHHHBABBHHHAAH-HABAHAHAHAAHAAH--BHAAAAABHHHABAAHBHHH-HHBHHHHAAHHAHHAHBBBAHHAHHHHAHHHHHBBAB

*000318_00011295 HHAAHHHBBBA-HBABHHABAHBHH-HHBHHHAHAHHBBBBHBHHAHABHABHHBHHHAAHABAHABHHHBBHAAHAHHBA-BHH-AHBHAHHHHAHAHBHHAAHAHHAAHAHABHHHHBBBHAHBAH-HHHHBA-HHBBABBA

*000362_00040275 BHBHBAHHBBHHHABHHHAHAHHAA-HAABABBHHHHHHHBBABAAAHAHHAHA-AHHHHBHBHBHBB-BHHAHAB-BAHHBABAHHHHAAHH-HBBABABHHBHHAHHBBAHHHBHBAHBHHAHHBABBHHHAAAH-HHHHBA

*001929_00166110 HHAABAHAABHBAHAHHHBBBHHHHAABAAAHBBBHBAHBAA-HHHHHHHHHBHBHHHAHHABBHBBA-HBHHBAA-HBAHHABABBHAHHHAHHBHAHHAHHAHA-HHBHHBHAB-HABHHHBHHBBAHHHB-AHHBAHBHHB

*003273_00062418 HAHABAHHBHBBHB-BHHHHAHBAHBHBHHHHHAHHHHHHAHBBAAHHHBBAAHHHHHBAHABABHHBHB-HAAHBABHAHAABAAHAHHAHABAHAABHHHBBHHA-HBHHBHHHHBAHBAHA-BHABBHHAB--H-AHHHBB

*003622_00055447 BBHABAHHAAH-HHBBBAAHBHBHB-AHBHBBHHHHABBH-HABHBHAHAAAHAABHHAABBBAHHHA-AHHHBBHAHAHA--ABHHAHHAHHBAHBAHHHBBHAH-HAHHBBHHAHHAHHBHBAHHHHHAHBAHAHBHBHBHH

*000352_00001724 BBHBHBBHHAHB-HAHAHHAHHBB-AHAHAABBBBBHHAHBB-HBHHHBABHBHBAHHHBBBBHABABBH-AAHBB-BHBBHHHAAHHA-HHHAH-HHHAHBHBHAAHHABAHHHABABBHBHHHHBHBHHHAHBHHAHHHAHH

*001342_00133864 BAAHHHAHHHB--AAHAAAHBHHHHAABHHHHBBBBAHHBBAAAHAHBBHHBHHHBHBHHHBHHABAH-HB-BHHH-AAHHBHBAAH-BBHBB-HHHAHABHHAHHAHHABBHAHA-HHBAHHBAABHH-HABAABHBHHHHBB

*002399_00033758 BBHHHBHHHAAB-HBAHHBAABHBB-BAHBAHAAHHHAHAAHHHBAAHAAHBHHHHAHBHHABAHAHBAABHBAAH-H-HHABBAAHAH-HAAAHHAHHHHA-HH-AAHAHAHHHH-BBBHHAAHHHBHBHBBBHBH-AHHHBH

*000037_00434076 H-HHAHABAHBABHBHHHHAHH-AHHHHHHHH-BAAHAHAHBHHAHHHHHABHHH-BHAAHHHHBHABHBBABAH-AHH-HA-BBAA--BHBHAAAHHAABHAHHHBAHBBAHA--AAHHBHBHAHAABAHHAABHHBAAAHHA

*001027_00089281 B-HHABHHHAAHHHHAAHBAA-H-BA-HABAHAAHHHAHA-HHHBAABAAHBAHHHAH-HHA-AHAHBAAHHBAAHAHHHHA-BHAHAHBHAAHHHAABAAAAHHAAAHAHAHHHHAB-BHHAAHHHBHHHBBHA-H-AHAHBH

*000040_00377737 A-HAHAAHAHA--B-HHBBHA-BAH-AABAABAAAHAHHHAHHAHHHB-AHABBH-ABAHBBBH-HHB-HAHHBHB-HAHHA-HBHHAAHHAHHAHAABHHAHHAAAHBHHAHBH-AHHBHBAAHHHHBHHHBHABH-BHHAHH

*000087_00578017 BBHHHBHHHAA--HHAHABAABHB-HBHHBA-AAHHHABABHHHBAAHAAHBHHHHAHBHHABAHAHB-A-HBAAHAHHHH-BB-AHAHBHAA-AHAAHHHAHHHH-AHAHAHHHHABBBBHA-HBHBB-HBBHHBH-AHAHBH

*000492_00231727 HBAHHBBHAAAHHA-BHHHHB-HHBHBHAHHHA-BBHBABAB-AHAAHHHHHHAAAH-HHHBHHHBAH-HHHBHHHAHBABABH-AHHAHBBA-BHHHBAHHHAAHABBHHBHHAA-H-ABHB-HHHBAAAHAAA-H-HBHBBA

*000198_00399354 -BHABBAHAHHH-HAHHHBHH-H-H-HBAHHBHHHAHBHHHBHBHHAAHHAHHA-BBAAABBHAHBHH-B-HBHBB-HBB-A-BBHHHHBHBBAAAHHB-HHAHHHBABBHHBAHBA-B-BBBHAHHABA-BHA--H-AHHHHB

*000261_00317794 H-HAAHHAHBAAAABHBBBHHBHBHBAHHHHA--BHABBHHBHHBHBHAHHHBABHBA-BAHHHHHAA-HH-BHAH-B-HA-HB-HHB-HABBABBHAAHHHHHBAHABHHBHH---AHHHHH-AHAH-HBHB-B-BBAHHAB-

*007845_00003956 HHBBHBBAH-HB-H-HAAHAHHBBB-A-BAHBBBHBAH-H-BHHBHHHBABABHB-HHHBBBBHABAH--BAAHBB-BHBBH-HAHHHAHHHH--AHHHAHBHBH--HHABAHHHA-A-BHBHHHHBB-HHHAHBHHAH-AAHH

*002521_00066912 HBBAHAAHHHAHAABHA-AHHHA-H-AHAAHAAABBH-BA-HBHHHHBHHBABAAHBABH-HBHHHBH--BHBBBH-BHHHA-B-AHAAHHHH-BHHHBHHHBHH-BHBA-BBHAA-H-ABHBH-HHBA--HHHBA--H-HH-B

*000033_00910422 AAHHHAHHHHAABBHAHBHBHHHBBHHHHABBAHABAHHBHHBABHHAHBAHBAAHHABHBHBAHAHHAAABHAABHHABBAAAHABHHHAHAHHHHHHHHABAAHBHHHHABBAHHHAHBAHHBBHBBHAHHHHAHHHABBAB

*000011_00353725 AHHBABHHAHAAHAHHBBHBHHHBHABBBAHABHAHBHHHBHBBBHHHAHAHBBHBAHHAAAHHHHBBBBBHHHAHHABAHABHHHHAHHHHAAAAABAHABABBHHHBHHHHHHAHABBHHAHHAHAHHBABBAHBBHHHHHA

*000031_00714320 HBHBHBHBHHBHBBBBHBHBHHBAAAAAHHHHHHHHHAHAABHHAHHHHHABHBBBHBABHAAHHAABHHBHBABBHAAHBAHBHAHABBHHHHBAHBAHBBHHAHHAHHBAHAHBHHBHHHHHHHABBHABHAHBHAABHAHB

*000109_00185991 AHHBABBHAHAAHAHHBBHBHHHBHABBBAHABHAHBHHHBHBHBHHHAHAHBBHBHHHAAHHBHABBBBBHHHAHHABABABHHHHAHHAHAAAAABAHABABBHHHBHHHHHHAHHBBHHHHHAHAHBBABHAHBBHHHHHA

*000120_00437969 AHHHAHBBAHHHBHAHHHABHHBHHHAHAABAHHBAAAHHBHAAHHBABHHBHBAABHBHAHBHAABAAHBAAHHHBHHAHBHAHHHAAAHHHHHBAHHHHAHAABAAHHAABBHHBHHABABHHBHAHAAHHHHHBHBHHABB

*000121_00453942 HAHBAHBAAHHAHBHHHHHBBBBABHABHAHBHBBHAAHHHHAHAAAAHHHBBBHBBHBHBHBHAABHHABAAABHBAHHHBHAHAHABAAAHBHHHHHBHAHHHHHHHHAHHBBBHBHABABBAHHAHHHABHAHHHBHHAHH

*000132_00265694 BABBHAAABBBHAABHHBABBABHHHAHHAAAHHABBBHHHHHHHHBHHAHBBAHHHAHBAHHABHHABAAHBAHHABBHAAAHBHHBBHAHBAHHBAAABHBBHAHHHHHHHHAHAAAHHHHBBHABHHBHBHHHAHHHHAHH

*000215_00407206 AHHBHHABAHHABBAAHBBHHHBBABBHHBABHABHHHABHBBHBHHHAAAABHHHHHHHBAAHHHBHAAHAHAHBAHHHHAHBHAHHHBHAABAHBHBBHHHHAHAHBBBHBHABAAHABHHHHBAHBBAHAHHBHBHHHAHH

*000311_00266799 AHBAHABHHBHHHABAAHABAHHHHHAHHHAHHBHHBHBHHBHHHAHBAHAAHHAABBAHHHBBBHHBHBAHBBHBBHBHHAHHHHHBAHAHBHAHHHBAAHABHBHHHHBAHBABHBAAAHBAHAHBBBHAHAABHHABHHHA

*000328_00065869 AHHHABAHAHHHBHHAHBBBBHHHHBHAAHHABHBHBAHHHHBHHHBHAHHABAHBHHBAAHHBHBBBHBAAHHHAHHAHHBBABHHBAAHAHBBHBAHBBBHAAHBHHHHHBHABABBABHHBBHHBAHAHHAAHBBBHHHHH

*000358_00017395 BAHAAHAAAABABHHBBHHHHHBHHABAHAAABHHBHHBBHHHBHBBAAHHHHHAABHHBABHHHHBHHAHAAAHHBBHAHAHBHHBAAHHBBHHHAHHBBHAHBAABABABHAHHAAHHHHBHHAHAHHBBHHAHHHAHBAHB

*000363_00114956 AHBHHHAHBBBHHHABHHAABHBAHAABAHHHHHABBAHBHAAHABHHAHBBHHHHHAHBHHHAHABBAHAHBAAHHBBAHABHABBAHHHHHHHHAHHHHAHHAHHBABBHBAAAABHHHBHBHHHHHAAABHHBBBHBBAHH

*000412_00094661 AHBAHHHAAHHHABHHHBBBHHBHBHHBHHAHBBAHHABHHHHBHHAAHBHBHHBHBBBHHHABHHBHHBHHBAHBABABBHAHHAAAHHHBHBBHAAHBHBBHHBHBHHAHHABHHHHHBABHHBHBBBHBBBAAHBABAAHH

*000516_00045065 HAHHHBHHBBHBABHBBBABHBHHHHHHAHABABHHBHAAHBHHHHAHAAHAHBBAAHHHBBBBAABBAHHBHAHHHAHAHABHBHHHHAAHHHBAHHBBAABAAAAHABHABHAAHHBHHHAHHHBBHHAHHHHABBBHHHHH

*001095_00118099 AHHAHAAHBHHHHAAHABHHHAAHBHAHAAHAHHBBHBBAHHHBHHABHHHHBHHHHHHAAHHHHBBAHHHHAHBHBBAHHHBBHAHAAAAHBABHHABBABHHHBBABAHHBBBBAHAABBHHAAAHHBHHHHHHBABBHHAB

*001541_00044160 AHHHABAHAHHHBHHABHBBBHHHHBHAAHHABHBHBAHBHHBHHHBHAHHABAHHHHBAAHHBHBBBHBAABHHAHHAHHBBHBHHBHAHAHBBHBAHBBBHAAHHHHHHHBAABABBABHHBBHHBAHAHHAABBABHHHHH

*001592_00076119 AHHHABAHAHHHBHBABHBBBHHHHBHAAHHABHBHBAHBHHBHHHBHAHHABAHHHHBAAHHBHBBBHBAABHHAHHAHHBBABHHBAAHAHBBHBAHBBBHAAHBHHHHHBAABHBBABHHBBHHBAHHHHAAHBHBHHHHH

*002900_00105503 BBHABHHBHAHAHHHBHAABHBBABHAABHBBHHBBBHHBHHAHAHAAHBHBHAAHBHAHBHHAABAAABHHHBBHBHAAHAAHHBAHHBAHHBHBHAHHHBBHAABHHHHABHHAHHHHHHBHHHHAHHAHBAHHHBHAABAB

*003044_00046615 AHHBHHBHBAABHHHBBHHAHAHAAHAHBBBBABHHAHAHHAHBAHHBHBBAHHHAAHHBBBBAAHHHAAHAAHBHHHBHBHAHBAHHAHBAHHHBHBBAHBHAHBBHHHAHHHHHBHHAHHAHAHHHHBHABHBHABAAHAAB

*003698_00045753 BBAHBHBHHHBAHAHBHBAAABHBAABHHABHABABHHAAHHHBBBBAAABHBAHAAAHHHBHBHHHHHHHAHHHHAHBABHBHBAHHHBABBBHAHHHAHHABAHAHABHHHHHHHHHHHAAAAHBHHHABHAAHHHAAHHBB

*003883_00010689 BBAHBHHHHHBAHHHBHBHHAHHBAABHHABHABABHHAAHHHBBBBAAABBBAHAAAHHHBHBHHHHHHHABHHHAHBABHBHHAHHHBABBBHAHHHAHHABAHAHABHHHHHHHHHHBAAAABBBHHABBAAHHHAAHHBB

*003946_00076881 BBAHBHBHHHBAHAHBHBAAABHBAABHHHBHABABHHAAHHHBBBBAAABHBAHAHAHHHBHBHHHHAHHAHHHHAHBABHBHHAHHHBAHBBAAHHAHHHABAHAHABHHHAHHBHHHHAHAAHBBHHABHAAHHHAAHHBB

*012460_00001709 HBHHHBHBAAAHBHBBBAHHBBHHAABHHAHBAHHBBHAHHBHHHAAAHAAHBHAAHHHHBBBHHHAHAHHHHBHAAAHHBHBHHBHBHHBAABHHHHHAHHHAAAABHHHBHBABBHBAHHHBBHHBAAAHBHABHBHBHBHA

*001559_00082024 ABHBHHBHBHABHHHBBHHAHAHAAHABBBHHABHHAAHHBAHBABHBHBBAHHHAAHHBHBBAAHHHAAHAAHBHBAHHBHAHAAHHHABHBAHBHBBAHBHAHB-HHHAHHHHHBHHHHHAHAHAHHBHABHBBAHAAHAAH

*000136_00201769 HHHBAHBBAHBBHBAABHBHBHBAABBBBHHBAAHHHHABHBHHAHHHAAAAHHHHBBAHBHAABHBBBHAAHAAHAHHHBHHBAAHHHHHAAHHHBHHAHHHHHA-HBHBHBHHBBAHAHHBBBBAAHBHHHBHAHHHBHAHH

*000473_00086967 HHBAHAHHHBHBBABHAHABAHHHHHHAHHABHHHABHBHHBABHAABAHHAHHAAHBAHBHBBBHHB-BAHHHHBBBBBHHHHHHHHHHAHHHAHBHBAAHABHBHHHBBAHBABABAAHHHAHABHBBHAHAABHHABHHHA

*001783_00114226 HHAAABHHAHH-BHHAAAHAHHHHHBBHAAHHHHHHBBBBHBHHHAAHBHAAHBHBABBHHHAHHAHBAHABBHHBAAHHBHBHHHHHHBHBBABAAHBBHAAABBAHBHHAHHHHBBBHAAAHAABHBBHHHHHBBABHHHHH

*014526_00000184 HHABHAHBAAAAHAAAHHBBBAHHHBAHHABBAHHHAHHHAHBAHBHAAHHHBAHHHHHABBHHHABHAHAHAHAHAHAHHAAH-HAHAAAAABBAHABAHBBHHHBHHBHHHHAAHHAHBAHBHBAHHBHHHHAAHBHHABHH

*000011_00123945 AHHBABBHAHAAHAHHBBHBHHHBHABBBAHABHAHBHHHBHBHBHHHAHAHBBHBAHHAAAHHHHBBBBBHHHAHHABAHABHHHAAHHHHAAAAABAHAB-BBH-HBHHHHHHAHABBHHAHHAHAHHBABBAHBBHHHHHH

*000031_00538050 HBHBHBHBHHBHBB-BHBHBHHBAAHAHHHAHHHHHHAHAHBHHAHHHHHABHBBBHBABHAAHHAAB-HBHBABBHAAHBABBHAHABBHHHHBAHBAHBBHHAHBAHHBAHAHBHABHHHHHHHABBHABHAHBHAAHHAHB

*000050_00243316 BAABABBBHBAAHAHHHAHBHABABAHABHHHBABAHHAH-HHAAAHHBHBAHHBBHBHAHHBHHHHBHHAABHABAHAHHHHHHHBAHHHHBAHAHHHAHAHHBHHBAAAHAAHHHA-HHHAHBBHHBHBABBAHHHHBBBBA

*000056_00694795 HBHHHBHBAAAHBHBBBABHBHHHAABHHHHBAHHBBHAHHBHHHABAHAAHBHAHAHHABBHHHHAHAHBHHBHA-AHHBBBHHBHBHHBAABHHHHHHHHHAAAHBHHHBABABHHBAHHAB-HHBAAAHBHABBBHBHBHA

*000108_00308258 BBHAHHABAHHHHHBBBHHHHABAHBHHBABHHBHHA-BBBBAHHHBAAAAABABBAHAHABHHHAHAAHHAHBBHAHHAAAHABAHAHHAHHAAHHHAAHBHHBBAHAAHHHAHAAHHHHHAH-HAHHBBHHAHAHHHBHBHA

*000778_00159346 BAHBAHHBHHAAAHHHBHABABBHBAHAHHHAHBHAHABHBAHAHHBHABHHAHAHBABAAHHHHHHBAAHAHHBH-BHHBAHBABAHBBAHAHHHBBHAHBHHBBAHHAHHHBHBHBBHHBBHBH-HHHBHBBHBHHHHABAA

*001226_00204605 HBHHAAHHBBHBABHHABABBHAHHABBAHBHHBHHAAHHHBAHHABHBHBBHBHHHBHAHHHAHAHAHAHHAHHH-AHAHA-HBAHHHHBABAHHAHBBHHAHABBHHBBABHAAHBABBBHBHHBHHHAHHABHHHHBHAAB

*003239_00001991 HBAHHBBHAAAHHAHBAHHHBBHHHHBHAHHHABBBHBABHBBAHAAHHHAHBAAAHBHHHBHHHBAHHHHHBHHAAHBHBA-HHAHHAHBBABBHHHBAHHHAAHABBHBBHHAHHHBAHHHB-HHBAAAHAHABHBHBHBBA

*000035_00283007 AABHHAHHBHAABBHAHBHBHHHBB-HHHABBAHAHAHHBHHBABHHAHBAHBAA-HABHBHBAHAHHAAABHAAB-HABBAAAHABHHHABAHHHHHHHHABAHHBHHHHHBBHHHHAHBAHHBBHBBHHHHHHAHHBHBBAB

*000386_00168789 ABHHHAAHH-AHAHHBBBHHHBABBHBHABHHHHHBHHAABHBHBHHBAHAHHBHAHBBBHAHHHHHHAHHHBHHAHHAHHABAABHAHHBBHAHBAHBHABBHHA-HAAHAABAHAHAAHAHHBHHAAHHAHHBAA-BBHABA

*001617_00033587 ABBABABABHHBBAAHAHHBABBHHHAHHHHBHHBHAHHHHAHBHHHHAABBAAAHHHHBBHHBAHHH-HHBBHBBBAHHHBBHABBB-BHHAAHHHBHBHHHAHBBHHHBABBB-BBAAAHHABHHHHHHAHHBHHHHBBBHB

*003235_00149138 BBABHHHHHABAHAHHHBAAABABAABBHBBHHBHBHHAHHHABBHBAAABHAHHAHAHAHHHBHABHABHAHHHH-HBABHBHHAHHHBAHBBBAHHAHBHABAH-HABHHAHAHA-HHBAHAHHBHHBABHAHHHHAAHBHH

*003483_00018385 ABHHAHHHBBHBHHAHHBABHAHHBHHAHAAAAAAHBHAAABBAHHBHAHAHHHHBBHHHHAABHHHAAABHBHBH-BHHBHHABAAHHBHHHBBBHBHHBHHHBB-BBAABBHHB-HHBHBBHHBHHHBBHHHHAHHBAABHA

*000082_00112843 HHHBHBHHBAHBBHAHAHHAHHBBBAHHHAABBBBBHAAHBBHHBHHHBABHBHBAHHHBBBBHABAH-HBAAHBB-BHBBHHHAHHHHHHHH-HAHHHAHBHBHAAHHABAHHBABABBBBHHBBBHBHHHAHBHHAHHHAHH

*000649_00348298 HAHHAHABHHABHB-ABAHBHHHHHHHBHAHAHABHHABHBHAHHHHHAAABAAHHBBBAAHHHHABB-ABAHBAHAHHAHAHHHAAB-BAHHAHHBBHHABBHHHHBBAAHHHHBABHAHHBHBBABHBBHHBH-HHAHAHHA

*000659_00028871 AHBBABHABHABAHHAHAHHHHHBHAABAHHHBBHHHAHHBHHBBAHBHBHAHBHABHHHHAHHAHBB-HHHHHHHBA-HHHHB-BBB-HAHHABHABHHBAHAAAHABHBBHBHAHABHHABHHHHBBBAHHHBHBBHBBAAB

*003331_00049374 BBHABBABAHBBAHAHHHHAHHH-H-HBAAHHHHHAHHHHHBBHAHAHHHABBAHBBAAAHBHHBHHHHBBHBABBHHHBHABBHAHHHBHBBHAAHHBABAAHHH-AHBHAAABH-BBHBHBAAHAABHHHHAHBHAAAHHHH

*003671_00045217 -BHHAABHB-H-BBHHAAHHHHHHHABBAHBHABHHHAHHAHABHABHHHHHHBHHABBAHHAHHAHAHABBBHHH-AHAHHAH-HHHHHHABABHAAHBAHAHHBAHBBBABHAHHBABHBAHHHBHHBAHBABBHABBHAAB

*000768_00131508 HAABBHBAHHHHHBHHBHBHHH-HABBHBHBABHBHAHHAAHBHHBBHBHHBHHABHBHAAHHHBABA-AAHHBAA-HBBH-HHHAHB-HHAHBHHABBHHBABBHHHAAAHHBA-BAHHABHHHHHAHHBBBAAHABHAHHBH

*000137_00180229 HBBBHHAA-HB--H-HHBBBHHHHABHHHHHABABHHAHHBBHHHHHHHHHABAHHHBHHAABBABHA-HAAHHBAAHAHBHAHBABHHAHBHHBHBHHHBHBHBABBBBBHBBABAAHHHAHHHHHAA-BBHHHBBHBHBAAB

*000021_00953227 -HHHHAHABBHHAA-HBBHBHHBHH-AHHAAAAHABHBHHHHHHHHBHAAHBBABBBAHBAHHABHHA-AHHBAHHABBHAA-AHHHBBHABBAHBBAAABHBBHAHAHH-HHHAABHAHHAHH-AABBHHHB-HHHHAHHAHH

*000181_00368932 HHHHABAHABHB-HHBHHHHBHBAA-HABHBHAABHHAHAAHAHHAHHHAHAHHHABHHHHBBHHABBBHBHAHAH-HHBHH-HHAHBHBHHBAHAHBHBBHAHHH-BBAHBAAHHABBAAABBBABHA-HHAHHBH-AAHHBA

*000356_00044512 H-ABAHHHHAHBAHAHHBABBAAAHHHHBHAAHBAHHHBB-HAHHAHBHHHHABABHHBHABBHHHAH-H-HBAHHAABAHB-B-BHHHHBBH-ABHHBHBABABHBB-AHHAAHAAHHHABHBHAAHABBHHABHHHHHHHHB

*000066_00145235 HAHHBHBABABHHBABHHBAAHHHAHHHBHBAAHHHH-HBHHBBHAHAABBBA-BBAAAHAHBHHAAAHBAHHAHAHH-HHHHH-BH-BHHAHHHHHAHBHHAAH-HHAABHHABABAHBAHAAHHAAABHHBHB-H-BHHBHA

*000850_00061863 AHHBAHBBAHHAHBA-HBBHHHBHABBBBHABHABHHHABBB-HHHBHAAAABHHHHBHHBAAABHBBAABAHAHB-HHHHAHBBAHHHHHAAHHHBHHBHHHHHH-HBBBHBHABBA-AHHHH-BAABBHHH-B-H-HHHA-H

*006266_00025924 -HHHHH-HBAH-AH-BHHHHA-H-H-HABBBHAHBAHHBHHBBHHHHHBHH-AHB-HH-HHA-H-BHA--BAAHBB-HH-AABHHHA---A-B-H-H-BAHB-BAAH-HHAABHAAA---B--A-AABB-ABH---B-HBA-HH

*000334_00096598 BBHABHHHAAB-HHBBBH-HHABHBBAHBHBHH-HBABBHBHABHBBAAAAABAH-HHAAHBBAHHHA-HAH-BBH-HHHAAHAHAHAHHAHH-AABHAHHBBHB--HAAHHHHHAHHHBHBHHAHBHHB-HHAHAH-HBHBBA

*000660_00060512 H--AABBHH-AHBHHAAAHAH-HHHHHBAAHHBHHHBBBBBBHHHAAHBHAABBHH-BBHHHAHHAHB-H-BBHHBHAHBA-BHHHHHHBHBBAB-AAHBAHAAAH-HBHHAHAHH-BBHA--B-AHHB-AHHHBBB-BHHBHH

*000015_00825160 ABHBBHHHHHABHBBHBHHHAHHBHBHHBHHAHAHHBHHBHBHHBABAABAHABHHBHBHABBHHBHAHHHABBAAHAHHBHHBHHHAHHBHBHHBAHBHHBBAAABBHHBAHHAAHHHAHABAHHHBAAAHABHHBAAHHABA

*000033_00887457 AAHHHAHHHHAABBHAHBHBHHHBBHHHHABBAHABAHHBHHBABHHAHBAHBAAHHABHBHBAHAHHAAABHAABHHABBAAAHABHHHAHAHHHHHHHHABAAHBHHHHABBAHHHAHBAHHBBHBBHAHHHHAHHHABBAB

*000084_00367259 ABBABABABHHBHAAHAAHHAHBHHHAHHHHBHHBHAHHHBAHBHHHHAABBAAAHBHHBBHHHAHHHAHHBBHBBBAHHAHHHAHBBABHHAAAHHBHBHHHAHBAHHHBABBHABHAHAHBABHHABHBABHBHHHHHBBHB

*000393_00293176 AAHBAHHHHHAABHHAHBHBBHHBBHHHHABBAHHBAHHBAHBABHHAHBAHBAAHHABHBHBHHAAHAAABAAABHHABHAAHHHBHHHAHABHHHHHHHABHHHBHHBHAHBAHHBAHBAHBBBHBBHHHAHHAHHHABBAB

*000452_00009386 AABHABHHABHHAHHBHBHHBHBHAHHHHHBHHAHABAHAHBHAHHBHHAAABHHHHAAHHBBBAAAHBBHBAHHBABHBHHHHBAHHABHBBAHBABBBBHAHHHABBAABAHHHHABBHHHBBAAHBHHBHHHBHAHHHHBH

*000472_00134202 ABBABABABHHBHAAHAAHHHHBHHHABHHHBHHBAAHHHBAHBHHHHAABBAAAHHHHBBHAHAHHHAHHBBHBBBAHHAHHHAHBBHBHBHAAHHBHBHHHABBHHHHBABBHAHHAHAHHHBHAABABABHBHHHHBBBHB

*000913_00170831 ABHBBHHHHHABHBBHBHHHAHHBHBHHBHHAHAHHBHHBHBHHBABAABAAABHHBHBHABHHHBHAHHHABBAAHAHHBHHBHHHAHHBHBHHBAHBHHBBAAHBBHHBAHHAAHHHAHABAHHBBAAAHABHHBAAHHABA

*005072_00000688 HABHHAHHBHAABBHAHBHBHHHBBHHHHABBAHAHAHHBHHBABHHAHBAHBAAHHABHBHBAHAHHAAABHAABHHABBAAAHABHHHABAHHBHHHHHABAHHBHHHHHBBHHHHAHBAHHBBHBBHHHHBHABHBHBBAB

*000008_01012708 BHHBBHHHBHHABBBHHBHHHAABBBHHHABHABHAHHAHABHABBAAAABBHBHAHBBAHHHBHBHHHBBHBHHHHBAABHHBBHABHHHBHBHBAAHAHAABBHAHHBHHHHBBAHHHBHHHHHHHHABABHAAHHAHABBH

*000051_00469872 HHHBAHBBHBAAHHHHHHHAAAHABBHABHAHAABHHHAHBBBAAAHHBHBAHHBBAHHHHHBHHHAHHAAHBHAHAHAAAHAABHHAHHHHBABABHHABAHHHHABAABHAAHHBAHHHHAABBHAHHHAHBHBHHBAHHHA

*000070_00471336 HHBHHBAHBBHHBBABHBAABBBHHAAHAHHBBHABBABHBHABHBHAAHBBHHHHHAHHBHHHHABBAHAHBAAHABBAHABHHBBHBHHABHHHAHHHHHAHHHABHHBHHHHAAHBHHBHBHHHHHAHABBHBBBHHBAHB

*000093_00130914 HHHAHHAAAHBABAHHHHHHHHHBHHBAHAAHHHBBAAHBBHABBBBAAHAHHHAABBHBHHHBABBAHHHAAHHHHBHAHAHBHHBAAHBBBHAHABBHBHHBBHAHHHHBHABHHAHHHBBHHHAAHBBHBAAAHHAABHHH

*000111_00557495 HHHHHAHHHAHHAHHBAHAHHHABBBBHAAHAAHBBHHHHHHABBHHHBHBABBAABHBBAHBBHHBHHAABHBABHHHHBHBHHAHAABHAHHAHAAHHHHHHAHAHHAHBBAAHHHAABABHAHHBHBAAHABABABHHHAH

*000118_00114776 HHBHBABAHHAHHHHBHAHAHHBHHHBAHBBHBHBAHHHHHHBHHHBHBHBBHHABHBBAHHBBBABABHAHHBABBAHBHHHHBAHBHHHABHHBABBAAHABBHHHAAAHHHABHAAHABHHHBHHBHBBBHAABBHHHHBH

*000118_00648813 HHBBBABAHHAHHHBBHAHHHHBHHHBAHBBHBHBAHHHHBHBHHHBHBHBBHHABHBBAHHBBBABABHAHHBABBHHBHHHHHHHBHHHABHHBABBAAHABBHHHAAAHHHABHAAHABHHHBHHBHBBBHAABBHHHHBB

*000246_00062827 BHABABBBHBAABAHAHAHBHABABAHAHHHHBAHABAAHHHHAAAHHBHBAHBBBHBHAHHHHHHHBHHAABHABAHAAHHHHHHBAHHAHBAHAHHAHHAHHBHHBAAHHAAHHHABHHHHHHBAHBBBABBAHHHHHBBHA

*000249_00035723 BHHBHBHHBBBHHABHHBHABHAHHHBAHBBBHHHABHAHHBHBHHBHAABAABHAHHHHBHHHHABHHHHAHABHAHBABHBHHAHHBBABBBHABHHHBAABAAAHAHHHAHHBHHBBBHHBBHBBHHABHAHHBAAAHBHH

*000270_00133471 HHAAHHAHBBAHABABHBABAHHHHAHHHABHAHAHHHHBBHBHHAHHHHAAHBBBHHAAHABAHABHHHHBHAAHAAAHBABHBAAHBAHHHHHAHAHBHAHAAAHAAAHAHABAAHHBBBHAHBHBHHAHAHAHHHHHAHHH

*000308_00172603 HHHHAABHBHAAHBBHHBHHHHBBHHBHABBBHABBHAAAHHHHBHAHHAAHAHHAHBABBHHHBHAHHHAHBHAABBHAAHBAABBAHHBBHHABAHHHHHBHHBBAAHHAHBAHBAAAHHHHBBHAAHHABAHAHHHHBHHH

*000385_00339652 HHBBHHHAHBHBHHAABBHBBHHBBBHHAAHHABBHHHABAAHABBBHBHHAHHBHHAHHHHBAABBBBHHHHBHAAHHHAHAHAHAHHHHHAAAHBHAHBBBHAAHHAHBHBHAAHBHHBAHBHHBHABABHHBHHHAHABAH

*000417_00012649 BHBAHAHHHBHBHABHAHAHAHHHHHHAHHABHHHABHBHHBABHAAHAHAAHAAAHBAHBHBBBHHBHBAAHHABHBBBHHHHHHBHAHAHBHAHBHBAAHABHBAHHBBAHBABHBAHHHHAHHBHBBHABAABHHAHBHHH

*000512_00083078 BHABABBBHBAAHAHAHAHBHABABAHABHHHBABABAAHHHHAAAHHBHBAHBBBHBHAHHBHHHHBHHAABHABAHAHHHHHHHBAHHHHBAHAHHHHHAHHBHHBAAAHAAHHHABHHHHHBBAHHHBABBAHHHHBBBHA

*001453_00101869 HHAAHHHBBBAHHBABHHABAABBHAHHHHHHAHAHHBHBBHBHHAHAHHABHHHHHHAAHABAHABHBHBBHAAHAHHBAAHHHHAHBHAHHAHAHAHBHHAABABHAAHAHABHHHHBBBHAABABHHHHHBAHHHBBABBH

*002268_00111382 BHHHBHAHBHHABBHBHBHHHAABBHHHBABAABHAHHAHHBHABBAAAABBHHHAHBBAAHHBHBHHHBBHBHHHABHAHHHBBBABHHHBHBHBAAHAHHABBHAHABAHHHHBAHHHBHHHHBHHHABHBHAAHHAHABBH

*003666_00005819 BHBHBAHHHBHBHAHHAHAHAHHAAHHAHBABBHHHHHBHHBHBAAAHAHHAHAAAHBAHBHBBBHHBHBAAHHABHBHBHHHHAAHHAHAHBHABBHBAHHABHHBHHBBAHBHBHBAHBHHAHHBABBHABAAHHHABHHBH

*003828_00039958 HHBHHBAHBBHHBBABHBAABBBHHAAHAHHBBHABBABHBHABHBHAAHBBHHHHHAHHBHHHHABBAHAHBAAHHBBAHAHHHBBAHHHABHHHAHHHHHAHHHABAHHHHHHAABBHHBHBHHHAHAHABBHBBBHHBAHB

*006452_00004634 BHBHAABHBHAAHBBHHBBHHHBBHHBHABBBHABBHAAAHHHHBHAHHAAHAHHAHBABBHHHHBAHHHHHBHAABHHAAHHAABBAHHBBHHABAHHHBHBHHBBAAHHAHBAHBAAAHHHHBBHAAHHABAHAHHHHBHHH

*003598_00065012 AHHHABAHAHHABHHABHBBBHHHHBHAAHHABHBHBAHBHHBAHHBHAHHABAHHHHBAAHHBHBBBHBAABHHABHAHHBBHHHHBHAHAA-BHBAHHBBBAAHBHHHHHBAHBABBABHHBBHHBAHAHHAAHBHBHHHHH

*006480_00000838 AHBAHABAHBBAHABAAHABAHHHHHAHHHHHHHHHBHBHHBHHHHHBAAAAHHAABHHHHHHBBAHH-BAHBHBBBHBHHBHHHHHHABAHHHHHHHBBAHAHABBHHHBABBHBBBAAAHBABAHBBBAAHABBHHABHHHA

*007326_00005211 AHBHHHAABBBBHHHBHHAABHHAHAAHAHHHHHHBBAHBHAAAABHHAHBHHHHHHHBBHAHAHABBAHAHBAAHABBHHABHAHAHAHHHH-HHHHAHBAHHAHHBABBHBAAAABBHHBHBHBAHHHAHBAHHBHAHAAAH

*000140_00311546 ABBBBBHABHABAHHAHHHHHBHBHBBHAHAHBBHHHAHABHHBBAHHHBBAHBHHHHHHHAHHAHBBHHHAAHHHHAAHHBABABHBBBHHHABHAHHHHAHHAA-ABHBAHBH-HABHHAHHHHHHABABAABBBBHBHAAB

*000459_00437137 ABHBHHBHBHABHHHBBHHAHAHAAHAHBBBHABHHAAHHBAHBAHHBHBBAHHH-AHABHBBAAHHBAAHAAHBH-HHHBHAHBAHHHHBAHAHBHBBAHBHAHBHHAHAHHHBHBHHHHHAHAHAHHBHABHBHABAAHAAB

*001143_00093134 ABBHBBBHHAHBBHHBHAHAABHBHBABBABAAHHHHHAHAHHBBAHAABHHAABBAAHHAHBHAHAHHBAHHHHA-BHHHBHBHBHHHHBAHHHBHBHBBHAABHAHHHBHHHHABHBHAHAAHAHAAHBHBAHHH-HAHBHB

*002733_00064205 ABHBHHBHBHABHHABBHHAHAHAAAAHBBBBABHHAAHHBAHBAHHBHBBAHHHAAHHBBBBAAHHHAAHAAHBH-HHHBH-HBAHHHHBAHAHBHBBAHBHAHBHHHHAHHHHHBHHHHHAHAHAHHBHABHBHABAAHAAB

*000463_00263592 HBAHHBHBAAAHHHBBHAHHBBHHHABHAAHHAHHBHHABHBHAHAHAHAAHBHAAHAHHHBBAHHAAHHHBHBHA-AHHBABH-BHBAHBHABBHHHHAHHHAAHABBHHBHBABHHBAHHHBBHHBAAAHBHABH-HBHBHA

*000474_00127838 AHHHHHABHHHABHHAHBBAABHBBBBHHBABHABAHAHABHHHBHHHAAHHBHHHHHBBBAHHHAHH-AHHHAHBBHHHBABBHAH--BHAABAHAHHBHHHHAHAHBBHHBHABAHHHBHHAHBABBBABAHBBHBAHHAHB

*001010_00209395 HBAHAHHAHBHHHHAHHBABHHHHBHHAAAHAAAAHBAAAABHAHHBHAHAHHHHHBHHBAAHBHHHABABABHBHABHHBHHABAAHHBHHHHBBHBHHBAHHBB-BBAABBHHBH-ABABBHHBHBHABHHH-HHHBAAHHH

*002102_00005902 AHHBAHABAHHAHBAAHBBHHHBHABBHHBABHABHHHABABBHHHHHAAAABHHAHHHHBAAABHBH-AHAHAHBAHHHHAHBHABHHHHAA-HHBHBBHHHHAHAHBBBHBHABAAHABHHHHBAHBBAHAHHBH-HHBAHH

*004008_00061238 AHBBHBAABBHBHHHHBBAHHHAHHBHHAABHHHBHHHHBAAHAHBBHBHBHBHHBHHBBHHAAHBBBBB-ABHAHABBHAABHAHAHHHHHHAAHBHAHHABHAA-AABBHHHAA-HBHBHHHBBHHHBAHHAHHBHHBAHHB

*000111_00245144 HHHHHAHHHAHHAHHBAHAHHHABBBBHAAHAAHBBHHHHAHHBBHBHBHBABBA-BHBBAHBBHHBHHAABHBABAHHHBHBH-AHAABHAHHAHAAHHHHHHHH-HHAABBAAHHHAAHABHAHHBBBAHHHBAB-BHHHAA

*001931_00056952 HHHBABBHAHA-HABHHHHBBAHBB-HHHAHABAHHBHHHBHBHBHHHAHAAHBHBHHHAAHHHHABBBBBAHHAHAABABHBHHHBAHHAHH-AAHHAHHHABBHABBAHHAHHABHBBHHHB-AHHBBBABHABBHHHBHHA

*000233_00687359 BBHHHBHAHHAH-HBAHHBAABHBBBBHHBAHAAHHHAHA-H-ABAABAAHBHHHAAHBHHHBAHAHBAAHHBAAHHHHAHABBBAHAHBHAABHHAABHAAHHHH-AHAHAHHHHHBBBBHAAHHHBHHHBBHBBH-AHHHBH

*001284_00240375 --HHAAHAHHH--B-AHH-HHBHBB-HHHH-BBBBHA--BAA-BAAAHHAAHHHHBBHHHHAHHAABH-AB--AHH-AABBB-AAAHB-HAAH-AAHBHHHH-HHH-AAA-HAHAB-HAHH-BHABHB--HHH--HA-HB-AHA

*000011_00782031 AHHBABBHAAAA-AHHBBHBHHHBHABBBAHAHHAHBHHBHHBBBHHHAHAHBHH-AHHAAAHH-HBBBBBHHHAH-HBAH-BHHAHAHHHHA-AAABAHABABBHHHBHHHHHHHBABBHHAHHAHAH-BABBAHBBHHHHHA

*001559_00071251 -BHBHHBHB-ABHHABBHBAHAHAAHABBBHHABHHAAHHBAHBABHBBBBAHHHAAHHBHBBAAHHH-AHAAHBHBAHHBHAHHAHHH-BHBAHBHBBAHBHAHB-HHHAHHHAH-HHHHHAH-HAHHHBABHBBA-AHHAAH

*000282_00455541 ABHAHHBHAHABHBBHBHHHH-HAHBHBBH-HBAHHBH-BHBHBHABBABHHABHBBHBHHBHHHHHAABHH-BAHAAHHBHHB-HHAHHBABHHBHBBAHBHAHA-BBHBAHHAABHHHHAB--HBBHAHHHBBHBAHBHABA

*001110_00094606 ABHHAAHH-BHBABAHABABAHAHB-BBAAHHHBHHAAHA-BAHHABHBHHHHBBAHBHAHAHAHHHA-AH-BHHHAAAHHHHHHABHHHBHHABHAHBBHHAHAB-HHBAABHAAHHHBHHHAHHHHHBAHB-BBH-BHBAAB

*001214_00218676 HHAAHAHAABHB-HAHH-HBBHHHB-ABAAHHABBHBHABAAHBHBHHHHHHBHBHHAAHHABHABBABH-HABHAAHBAHHAHABBHABHHAHHBHAH-ABHHHA-HBBBHBH-BHBAHHAHBHHBHAHHHBHBHH-AHBHHB

*001186_00359301 HBHHAAHH-BHHABHHABABB-HHBHBBAHBHHBAHAAHHHBAHHABHBHBBHBHHHBHAHHHAHAHAHABAAHHHHAHAHH-H-AHHH-HABAHHAHBBHHAHABHHHBBABHAAABABBBHB-HBHHBAHH-B-H-HBHAAB

*000878_00091865 HHABHAHAAHHBAH-ABBHBBBHHHBAHAAHHAHBABAHBAA-HHBHHBHHHBHB-HAHHHABHABBABH-HHBHA-HHAHB-HABBHHAHHAAHBHAHHHBHHAA-HHBBHBH-B-HAHHAHBHHBHAH-BBHABHBAHHBHB

*000096_00264828 A-HAAHBBH-HHBHAHHBABHHBBHHAHAHHAHHBAAAAHAH-HHH-ABHABHBAAHBBHAHBBAHHA-H-ABHHB-HAAH-HABHHAAAHHH-HBHBHAAABAH--AAH-AHHH-HHHABBHHAHHABABHHHH-BBBHHAHB

*000966_00085063 BBHH-AAHAHH-BHHHHHHBA-HBABHHBABBAHBAHBBHBBBHHABHBHHBAHHBBBABBHHHBHBH-HHBAAHH-AHHAA-HBBHAHHAHBHBBBAAHABHBA--HBHHHHHHA--AABHH-ABBAHH-BHHBBH-HBHHHH

*008224_00002299 H-AA-AAA-BH-AH-HAH-BBHHHH-ABAAHHA-BHB--B-AHHHBHHHHHHBHBHHAAHHABHABBA-H---BHA-HBA---H-BBBHH-HA-HBH-HHABHHH--H-BBHBHA--HA-H-HBABBH-A-HBHB-A-A--HHB

*000033_00770291 AAHHAAHHHHAABBHAHBHBHHHBBHHHHABBAHHBAHHBHHBABHHAHBAHBAAHHABHBHBAHAHHAAABHAABBHABBAAAHABHHHAHAHHHHHHHHABAAHBHHHHABBAHHHAHBAHHBBHBBHAHHHHAHHHABBAB

*000066_00047948 H-HBBHBHBAHA-B-BHBBAAHHHH-HHBHBAAAHHHHBBBHBBHAHAABHBAHB-AHAHAHBHHAA-HBAHHAHAHHHHHH-H-BHHB-HAHBAABAHBHHAAHAHH-ABHHAB--H--A-AA--AAAAHHBH-HH-BHHBHH

*000011_00121688 AHHBABBHAHAAHAHHBBHBHHHBHABBBAHABHAHBHHHBHBHBHHHAHAHBBHBAHHAAAHHHHBBBBBHHHAHHABAHABHHHHAHHHHHAAAABAHABABBAHHBHHHHHHABABBHHAHHAHAHHBABBAHBBHHHHHA

*000011_00154744 AHHBAHBHAAAAHAHHBBHBHHHBHABBBAHABHAHBHHHBHBHBHHHAHAHBBHBAHHAAAHHHHBBBBBHHHAHHABAHABHHHHAHHHHAAAAABAHABABBHHHBHHHHHHABABBHHAHHAHAHHBABBAHBBHHHHHA

*000012_00321072 BAHBABBAHABABAHBBHHAAHHBAAHBHAHBHBHAAHHBHBAHBHBHHABABHAHBHHBBHBAABAHBHBAHHBHHHABHAAHHHAHHBHHAAHHHHHHHBHHHBABHHHHHHHABABBABBAHBAHHBHHHAHAHHHAAHAH

*000077_00017244 BAHBAHHBHHAAAHHHBHABABBHBHHAHHHAHBHABABHHAHAHBBHABHHABHHBABAAHHHHHHBAAHAHHBHHBAHBABBABHHBBAHAHHABBHABBHBBHBHHHHHHBHBHAHHHBBABHHHHHBABBHHHHHBHBAA

*000103_00302946 HAHBAABAAHHAHBHHHHHBBBBHBBHBHHHBBBBHAAHBAAAHHAAHHAHHBBHBBBHHBHHHAABHHABHAAHBBAHBHBHAHAHHBAAAHAHHHHBHBHHBHHBHHHAHAHHBBBAHHHBBAHHHBHHHBAHHAHBBHAHB

*000163_00145507 HBAAABHAHBHHHBHHBHAHHHHHBBHHAAAHAHAHBAAAABHHHBBBHBBBAAHHBHBHHAHHBHBABABHBHBHAHHHHHHAAHHHBHHBBBBHHBHHBAHHBBABBAHBHBHHHHBHABBHABHBHHBBHHAHHHBHHBAA

*000210_00128733 BAAHHHAHHABHHAHAAAAABAHHHHABHHHABBBBAHHBHAHAHAABHHHBHHABHBHHABHHABHHBHBHBHHHAAAHHBHBAAHBBBHBBAHHHABHBHHAHBAHHABHHAHHAHHBAAHBAABHHBHHBAHBHBHHHHBB

*000210_00450105 BAAHHHAHHABHHAHAAAAABAHHHAABHHHABBBBAHHBHAHAHAABHHHBHHABHBHHABHBABHHBHBHBHHHAAAHHBHBAAHBBBHBHAHHHABHBHHAHBAHHABHHAHHAHHBAAHBAAHHHBHHBAHBHBHHHHBB

*000220_00340919 AHHBAHBHAHHHBHAHAHHBHHBHHHAHAABAHHBHAHHHBHAAHHBAHHHBABHHBHBHAABHAABAHHBAAHHHBHBAHBHABHHAAAHAHBHBAHHHHAHHABHAHHAABBHBBAHABABHHBHAHAAAHHHHBABHHAHB

*000245_00368453 BBBAHHBBAAHAHHBHHBBHABBHHBHBBABHBBHAABBBHBAAHAHHHAAHHAHBHHAHHAHHHAHABHHHHBHAHBAHHBHAHAHHAHHABAHAHHHAAHABABHABAHHAAHHBBHHHAHAHHHAAHAAHAAHBHBHHBHH

*000339_00030205 AHHAABAHABHBAHHBHHHHBHBHAHHAHHBHHABAHAHAHHAHHABHHAAAHHHHBHAHHBBBHAHHBBBBAHABAHHBHHHHHAHBABHBBAHAABBBBHAHHHHBBAABAAHHHABHHABBBAHHHHHHAHABHAHAHHBH

*000475_00312784 AHHBAHABAHHABBAAHBBHHHBHABBHHBABHABHHHABHBBHHHHHAAAABHHAHHHHBAAHBHBHAAHAHAHBAHHHHAHBHABHHHHAABHHBHBBHHHHAHAHBBBHBHABAAHABHHHHBAHBBAHAHHBHBHHBAHH

*000607_00278297 AHBAHAHHHBHHBABAAHABAHHHHHAHHHAHHBHHBHBHABHHHAABAHAAHHAAHBAHHHBBBHHBHBAAHBHBBHBBHHHHHHHBAHAHBHAHHHBAAHABHBHHHBBAHBABHBAAAHHAAAHHBBHAHAABHHABHHHA

*001065_00090473 AHHAHAAHBHHHHAHAABHHHAAHBHAHAAHAHHBBHBBAHHHBHHABAHHHBHBHHHHAAHHAHBBHHHHHAHBHBBAHHHBBHAHAAAAHBABHHABBABHHHBBABAHHBBBBAHAAHBHHAAAHHBHHHHHHBABBHHAB

*001312_00189844 AHHAHAAHBHHHHAHAABHHHAAHBHAHAAHAHHBBHBBAHHHBHHABHHHHBHBHHHHAAHHAHBBHHHHHAHBHBBAHHHBBHAHAAAAHBABHHABBABHHHBBABAHHBBBBAHAABBHHAAAHHBHHHHHHBABBHHAB

*001320_00074737 HBAHABHAHBHHHHHHBBABHHHHBHHHAAHHAAAABAAAABHAHHBHHHAHAAHHBHBHHAHBBHBAHABHBHBHAHHHBHHAAHHHBHHHBBBHHBHHBAHHBBABBAABBHHBHHBHABBHABHBHABBHHAHHHBHHBAA

*001327_00014752 AHHBBHABBHHHHHAAHBAHBBAHAHHAHAAHHAHBHBAHHHAAHHBHAHAHHBHHBHAHHAAHHAAAHBBHBHBBHBHABHHBHAHAHBHHHBABHHBAHHHBBBAHHAHBBHABHHHBBBBBBBHABABHHHAHBAHHHBHH

*001553_00261695 BBHBBHHHHABAHAHBHBAAABABAABHHBBHHBABHAAHHHHBBHBAAABHAHHAHAHHHHHBHAHHABHAHHHAAHBABHBHBAHHHBAHBBHAHHAHBHABAHAHAHHHAHHHHHHHBAHAAHBBHAABHAHHHHAAHBHB

*002100_00014622 BBHABHHHAABHAHBBBAAHHHBHBAAHBHBBHAHBABBHBHABHBBAAAAABAABHHAABBBAHHHABHHHHBBHBHHHAAHAHAHAHHAHHHAHBAAHHBBHBHAHAHHHHHHAHHHHHHHHAHHHHHBHBAHAHBHBHBBA

*002699_00004360 BBAHBHAHBHBAHHHBHBHHAHHBAHBHBABAABAHHHAAHHHBBBBAAABBBAHAAABHABHBHHHHHHHABHHHABHAHHBHHAAHHHABBBHAHHHAHHABHHAHABAHHHHBHHHHHAAAABHBHHHBBHAHHHAHABBB

*002888_00058051 BBHABAHHAABHBHBBBAAHBHBHBAAHBHBBHAHBAHBHBHABHBHAHAAABAABHHAABBBAHHHHBHHHHBBHHHHHAAHAHHHAHHAHHBAHBAAAHBBHBHAHAHHBBHHAHHAHHHHBAHHHHBHHBAHAHHHBHBHA

*003804_00019300 AHAHHBHHAHHBBBAHAHHAHHBBBAHHHAABBBBBHHAHBBBHBHAHBABBBHBAHHBBBBBBABAHBABAAHBBBBHBBHHHAHHHHAAHHAHAAHHAHBHBHAAHHHBAHHHHHHBHBBHHHHBHBHHHAHHHHHHHHABH

*004757_00062852 BBHABAHHAABHHHBBBAAHBHBHBAAHBHBBHAHBAHBHBHABHBHAHAAABAABHHAABBBAHHHHBHHHHBBHAHHHAAHAHHHAHHAHHBAHBAAHHBBHBHAHAHHBBHHAHHAHHHHBAHHHHHBHBAHAHBHBHBHA

*006615_00023142 AHHBHBBHHHHBBBAHAHHAHHBBBAHHHAABBBBBHHAHBBHHBHHHBABHBHBAHHHBBBBBABAHBABAAHBBBBHBBBHHAHHHHAHHHAHAHHHAHBHBHAAHHHBAHHHABABBBBHHHHBHBHHHAHBHHAHHHAHH

*000005_00815196 HHBBHHHAHBHBHHAABBHBBHHBBBHHAAHHABBHHHHBAAHABBBHBHHAHHB-HAHBHHHAABBBBHHHHBHAAHHHAHAHAHAHHHHHAAAHBHAHBBBHAAHHAHBHBHAAHBHHBHHBHHBHABABHHBHBHABABAH

*000020_01293917 HHBBHHAABBHAHHBHBBAHHHHBHHHHAABHHHBHHHHBAAHAHBBHBABHBHHBHHBBHHAAHBBBBBAABHAHABBHAABHBHAHHHHHHAAHBHAHHABHAA-AABBHBHAAAHHHBBHHBHHHHBAHHAHHBHHBAHHB

*000473_00181725 HHBAHAHHHBHBBABHAHABAHHHHHHAHHABHHHHBHBHHBABHAABAHAAHHAAHBAHBHBBBHHBHBAHHHHBBBBBHHHHAHHHAHAHBHAHBHBAAHABHB-HHBBAHBABHHAAHHHAHABHBBHAHAABHHABHHHA

*000558_00052198 BHHBHHHHHBBAHABHHBHAHHABHBBAHBBBHHHHHHAHHBHBBHBAAABAABHAHHHHBHHHBABHHHHAHABH-HBABHBHHAHHBBABBBHABHAABAABAAAHAHHHAHHBHHHBBHHHBHBBHHABHAHHBAAAHBHH

*000823_00160303 HHBHHHAHBBBBHBABHHAABBBAHAAHAHHHHHABBAHBHHABABHAAHBBHHHHHAHBHHHAHABBAHAHBAAHHBBAHABHABBAHHHHBHHHAHHHHAHHAHHBABHHBAHAABHHHBHBHHHHHAAABHHBB-HBBAHH

*001481_00096660 HHHBHABBAAHHBAAAHHBHBAHAHHAHHABHHHHHHAHHAABAAHHHAHHHBABBHHHAHBHBHABHAHAHAHAHAHAHHAAHBAAHAHHAABBAHABHHBBHHHBAHBHHHHAABHAHBAHBHBAHAHHBBHAAH-HHABHB

*001656_00065782 BHHBAHHBHHHAHHHHBHHHAHBHBHHAAHHAHHHABABHAAHAHBHHABHHABHHBABAAHHHABABAAHAHHBH-BAHBABBHBHBHBAHAHAABHAABBABBHBHHHHHHBHBHHAHHBBABAHHHHBABBHHHHHBHBAA

*001720_00048197 BHBA-AHHBHBHHHHHHHAHAHAAABHBAHAHHAAAHHHHHHBBAAHHABHAAHHAHHHAHABABHHBBAHHAAABABHHHAABAAHAAHAHAHHBHABAHHHBHHAHHBBAAHAAHBAHBHHAHBHABBHHHBHAHHAHHHBB

*000181_00507063 HHHAABAHABHBAHHBHHHHBHBHAHHABHBHHABHHAHAHHAHHABHHAHAHBHBBHHHHBBHHAHBBHBBAHAHAHHHHH-HAAHBHBHHBAHAABBBBHAHHAHBBAABAAHHABBAHABBHAHH-HHHAHHBHAAAAHBA

*000510_00020356 HAHAABBHHHHHBHBAAAHAHBHHHAABHHHHAAHHBBBHBBHBHHAABBAAHBHHABBHHAAHAAHBAHABHHHH-AHBABBHHHHHHHHBBABAAAHBHHAAAHAHBHBAHHHHBBBHAAAHAAHHHHAHHHH-BABHHBHH

*000884_00083733 AHHHABAHAHHABH-ABHBBBHHHHBHAAHHABHBHBAHBBHBHHHBHAHHABAHHHHBAAHHBHBBBHBAABHHAHHAHHBHHBHHBHAHAHBBHBAHBBB-AAHAHHHHHBAABHBBABHHBBBHBHHAHHAAHBHBBHHHH

*000193_00412031 ABHBAHAB-BHHHHAHHBAHHBHHB-HAHAAHAAAHHAAAABHAHHBHAHAHHHHHBAHHHAABHHHAAABHBABH-BHHBAHHHAAHHBHHHBHBHBHHBHBHBBABHAABBHABAHHBHBBHABHAHHBHHHHAHHBAABHB

*000540_00126373 AAHBHABBAHH-HAAAHHBHBAHAHBAHHABHHHHHHAHBAABAAHBHAHHABABBHHHAHBHBHABH-HHHBHAH-HHHHAAHBHABAHHAABHABABHHBBHHHBAHBHHHHAABHAHBAHHBBAHHHHBBHAAHBHHABHB

*001054_00205533 HHHHAHAHHHABHBHABAHBHHHHHAHBHAHAHA-HHABHBHAHAAHHAAABAAHHBBBAAHHHHABBAABAHBAA-HHAHAAHHAAB-BAHHAHHBBHHABHHHHABBAAHHHAAHBHAHHBHHBABHBHHHBHBHHAHAHHA

*001188_00099095 HHABABBBA-AABAHHHHHBBAHABAHHAHHABAHHBHAH-HHAHHHABHBAHBHBHHHAAHHHHAHBBBAAHHABAABHBHBHHHBAHHAHHAAAHHAHHHABBH-BHAHHAHHHBABBHHHBHHHHBBBABHAHBBHHBHHA

*000034_00013100 AHHHABAHAHHHBHHABHBBBHHHHBHAAHHABHBHBA-BHHBHHHBHAHHABAHHHHBAAHHBHBBBHBAABHHAAHAHHBBHBHHBHAHAHBBHBAHBBBHAAH-HHHHHBAAB-BBABHHBBHHBA-AHHA--BABHHHHH

*000067_00230894 HBBHHBHHHHAHBHBHBHHBHBHHA-HAABHHAHHBBHAAAHBHHABHHAAHBHABAHAAHBBHHHAHAHBHBBHAAAHBBHBHHBHBHHBAHBHHHBHHHBHAAABBAH-BABABHHB-HAAH-HHBAAAHBAHHB-HHHBH-

*001018_00144459 A-BABABAHHB-HA-AAHHHAABHHHAHHHHHHBBHHAHHHHHHHHHHAAHAHHAABHHHHHHHHAHHBHA-BHBB-ABHHBBH-BBHABAAA-AHHHBBAAAHHBBHHHBABBHBBHAAAHBABHHBBBHAHABBHHABBHHA

*003293_00042950 BBHABBAHAHHHAHAHBHHBHHHHHABBAHHHHAHAHBHHHBHBHHAAHHAHHAA-BAAAHBBH-BHH-BBHBHBBAH-BHABBBHHHHBHBBHAAHHBAHHAHHHBABBHHBAHBABBHBBBHAHHABAHBHA-BHAAHHHH-

*000776_00236827 AHAHABBHAHHAHAAHHBBBHHABH-BBHAHAHHAAHBHBHHHBBBHHAHAHHHH-AAAAAHHHHBAB-HBAHHAHBAHHHHBH-HHHBHHHH-AAABHHABHHAA-HBHHAAHHHBHBBABAHBAAAH-AHBBHABAAH-HHA

*000318_00008953 HHAAHHHBBBA-HBABHH-BAHBHHHHHHHBHAHAHHBHBBHBHHAHABHABAHBHHAAA-ABAHHBH-HBBHAAHAHHBAAHHHHAHBHAHHHHAHAH-HHAAHA-HAAHAHABH-HHBBB-ABBAHAHAHHBAHH-BBABBH

*001292_00297194 H-AAHHHBBBA-AB-BHHABAHBH-AHHHHHHAHAHHBHBBHBHHAHAHHABHHBAHHAAHABAHABHBH-BHAAH-HHHAAAH-HAHBHAHHAHAHAHBHHAAHAAHAAAAHABH-AHBBBHHHBHBHHHHH-ABH-BBABBH

*001918_00120209 AHH-HABBA-HHHA-AHHBHBHHAA-AHAABHHHHHHAHHAABAAHHHAHHHBABBHHHAHBHBAABHHHAHAHAH-HAAHAAHBHAHABHAABBAHABAHB-HHHBAHBHHHHA-HH-HBAHBBHAHH-BBBHAAH-BHABHB

*000424_00421866 ABHBAHHHHHHAHHAHHBAAHBHHB-HAHAAHAAAHHHAA-BBAHHBHAHAHHAHBBHHHHAABA-HAAABHBHBHHB-HB-HB-AAHHBHHH-BBH-HHBHAHBB-BHAABBHAB-HHBHB-HABHAHHBHHHHABABAABHH

*006296_00000908 HHAAABAHHAHHBHHAAA-AHHHHH-BBAAHHHHHHBBBB-BHHHAAHBHAAHBHB-BBHHHAHHAHBAH-BBHHBHABBHH-HHHHHHBHBB-BAA-BBHAAAB-ABBHHAHHBHHBBHAAAHAAHHBHBHHH-BB-BHHBHH

*000480_00084386 HAHAAHHH-ABHAB-HHHAHA-AAHHHAHAABBBHAAHBBHHAAHAHAAHHAABAHHHH-BAHHAHHABHHA-AHHAA-HHHHBBABHHAHHH-B-HHBHAHHHAH-HHBAABBHABB-BHHHABHHHHA-ABHHAA-BHBAAB

*001978_00110211 AHAHHHHHBBB-HH-H-ABHH-HABHBHABHHAHHBBHAHBABHHHHBAHHHABHAHHHHHBAHHHBAHA-BHABAAAAHH-BAH-HABAHBA-ABABBHHB-HAA-BBH-HAHABHBHAHAHHBHBHAA-AHHBHAAHAHHHA

*002984_00011253 BBHHHBHHH-ABAH--HABAA-HBBHBHHBAHAAHHHAAAHHHHBAAHAAHHHBHAAABHHABAHAH-AA-HBAAHAHAHHA-BAAHAHBHAA-AHAAHHHAHHHH-AHAHAHHHHBBBBBHA--HHBHBBBBHHBH-AHHHBH

*000940_00071464 HAHBAAHHH-AAHHBAHHHBBHHBBHH-HABBAHHBA-HBAHBABBHAHBHHBAABHABHBHBHHAAH-AABAHAB-HABHA-H-HBHHHAHABHHHHHHHA-HHB-HBH-AHBA-HBAHBAHBBBHHB-HHAHHBHHH-BBA-

*000493_00216990 -HHBAHBBAHH-ABAAHH-AHHB-A-B-BHABHABHHHABHBBHHHBHAAAABHHHHBHHBAAABHBB-AAAHAHB-HHHHA-BHAHHHHHAA-HABHHBHH-HH-HHBBBHBHABBAH-HHHH-BAAB-AHHHHAH-HBBAHA

*001048_00230953 HHABBHBA-HHBHBBHBBBHHBBHABBHBHHAB-BHAH-A-HBHHBHHHHHBHH-BHBHAAHHABABA-AA-HBAH-H-BHHBHBAHBHHHAH-HHABBHHBABBHHA-AAHHHABHHHHABH-HHHAHHBBBA--ABHAHHB-

*001445_00112389 BBH-BBAH-HH-AHAABHHAH-HHHAHB-HAHBHHAH-AHHBHBHHAABHAHHAABBAAAHBHHHBHHABB-BHBBAHBBHA-B-HHHHBHBBBAAHHBAHHAHH--ABBBHHAHAAB-BBBB-AHHAB-HHHAH-H-AHHHHH

*000289_00542679 ABHAHHABBHHHHHHAHHBAHHHHHBHBBBHHHBHAHHHAAAHBHBHBBAHHHAHAHAHAHBHBBHHHABHHBABBBHHABHHAHABHHHAHHHBBBABBHHAHBBBHAAHBBHABHAHAHHHHHAABBBBHBABBHHHBBABB

*000326_00188542 ABHBBAHHHHABHBBHBHHHHHHBHBHHBHHAHAHHBHHBHBHHBAHAABAHABHHBHBHABBHHBHHHHHABBAAHAHBBHHBHHHAHHBHBHBBHHBHHBBAAABBBBBAHHAAHHAAHABHHHHBAHAHABHABAAHHABA

*000452_00008526 AABHABHHABHHAHHBHBHHBHBHAHHHHHBHHAHABAHAHBHAHHBHHAAABHHHHAAHHBBBAAAHBBHBAHHHABHBHHHHBAHHABHBBAHBABBBBHAHHHABBAABAHHHHABBHAHBBAAHBBHBHHHBHAHHHHBH

*000472_00169225 ABBABABABHHBHAAHAAHHHHBHHHABHHHBHHBHAHHHBAHBHHHHAABBAAAHHHHBBHAHAHHHAHHBBHBBBAHAAHHHAHBBHBHBAAAHHBHBHHHABBHHHHBABBHAHBAHAHHHBHAABHBABHBHHHHBBBHB

*001480_00094481 ABHHHAAHHHAHAHHBBBHHHBABHHBHABHHHHHBHHAABHBHBHHBAAAHHBHAHBBBHAHHHHAHBHAHBHHAHHAAHABAABBABHBBHHABAHBHABBHHHHHAAHAABAHHHAAHHHBBHHAHHHAHHBAAHBBBABA

*001996_00102074 ABHBHHBHBAABHHHBBHHAHAHAAHAHBBBBABHHAHAHHAHBAHHBHBBAHHHAAHHBBBBAAHHHAAHAAHBHHHBHBAAHBAHHAHBAHHHBHBBAHBHAHBHHHHAHHHHHBHHAHHAHABHHHBHABHBHABAAHAAB

*000007_00690643 BHABAHABHHBAHHHHBHHHAHBHBHAAAHHAAHHABBHHAAHAHBHHABBHABHHBABAAHHBHHABAAHAHABHHBAHBHBBABHHHBHAAHHABHAABBABBHBHHHHAHHHBHAHHHBBABAHHHHBAHHBHBHHBHHHA

*000024_00206138 BHHAHHBAHBHHAHAAAHHAHHAAABAAAHHHBBHHHBBBAAHHAHHBHHBHHAABHBHAHAAAHHHAAHAHHHBBAHBBBHHHAHHAHHHAAHAHABHABHHBHHAHAHHBBBABHHHBBHHHBHAABBHBABHBHHHBHABB

*000051_00405387 HHHBAHBBHBAAHHHHHHHAAAHABBHABHAHAABHHHAHBBBAAAHHBHBAHHBBAHHHHHBHHHAHHAAHBHAHAHAAAAAABHHAHHHHBABABHHABAHHHHABAABHAAHHBAHHHHAABBHAHHHAHBHBHHBAHHHA

*000085_00209525 HHHHHHAABHBHBHAHHBBBHHHHABHHHBHABHBHBHBHBHHAHHBHAHBABAHHHBBAAABBABHHBHAAHHBAABAHBHHHHABHAAHAHHBHBAHHBHBABABHHBHHBBABAHHHHHHHHHBAABBBHHHBBHHHBAAB

*000132_00225376 BHBBHAAABBBBAABHHBABBABHHHAHHAAAHHABBBHHBHBHHHBHHAHBBAHHHAHBAHHABHHABAAHBAHHABBHAAAHBHHBBHAHBAHHBAAABHBBHAHHHHHHHHAHHAAHHHHBBHABHHHHBHHHAHHHHABH

*000246_00030300 BHABABBBHBAABAHAHAHBHABABAHAHHHHBAHABAAHHHHAAAHHBHBAHBBBHBHAHHHHHHHBHHAABHABAHAAHHHHHHBAHHAHBAHAHHAHHAHHBHHBAAHHAAHHHABHHHHHHBAHBBBABBAHHHHBBBHA

*000308_00291841 HHBHAABHBHAAHBBHHBHHHHBBHHBHABBBHABBHAAAHHHHBHAHHAAHAHHAHBABBHHHBHAHHHHHBHAABBHAAABAABBAHHBBHHABAHHHHHBHHBBAAHHAHBAHBAAHHHHHBBHAAHHABAHAHBHHBHHH

*000317_00251321 HHABABBAHBHBBHAAAHHHAAHHHAHHHHHBABBHHHBBHBHBHHHAHAAHHHABHHHHBAHHABHABHBAABAAAAHHHHBBHHHABHHAHBAAABHHHBHHAHHHHAHAHAHBBHBBAAHABHAHABAAHAAHHAAHHHBH

*000317_00277268 HHABABBAHBHBBHAAAHHHAAHHHAHHHHHBABBHHABBHBHBHHHAHAAHHHABHHHHBAHHABHABHBAABAAAAHHHHBBHHHABHHAHBAAABHHHBHHAHHHHHHAHAHBBHBBAAHABHAHABAAHAAHHAAHHHBH

*000355_00050768 HHHHAAABHHABHBHHBAHHHHHHHHHHHAHAHABHHABHBAAHHHHHAAABAABHBBBHABHHHABBHABHABAHAHHBHAAHAAHBBBAHHAHABBHAHBHHHHBBBAAHAHHHHBAAAABHHHBBHHHHABHBHHAAHHBA

*000417_00171033 HHBAHAHHHBHBHABHAHABAHHHHHHAHHABHHHABHBHHBABHAAHAHAAHAAHHBAHBHBBBHBBHBAAHHABHBBBHHHHBHBHAHAHBHAHBHBAAHABHBHHHBBAHBABHBAHHHHAHHBHBBHABAABHHAHBHHH

*000449_00101874 BHHBAHABHHBAHHHHBHHHAHBHBHAAAHHAAHHABAHHAAHAHBHHABBHABHHBABAAHHHABABAAHAHABHHBAHBHBBABHHHBHAAHHABHAABBABBHBHHHHAHBHBHAHHHBBABAHHHHBAHHHHBHHBHHHA

*000461_00312987 HHBBBABAHHAHHHBHHAHHHHBHHHBAHBBHBHBHAHHHAHBHHHBHBHBBHHABHBBAHHHBBABABAAHHBABBHHBHHHHHAHBHHAABHHBABBAAHABBHHHAAAHHHABHAAHABHHHBHHBHBBBHAABBHHHHBB

*000473_00165683 BHBAHAHHHBHBBABHAHABAHHHHAHAHHABHHHHBHBHHBAHHAABAHAAHHAAHBAHBHBBBHHBHBAHHHHBBBHBHHHHAHHHAHAHBHAHBHBAAHABHBHHHBBAHBABHBAAHHHAHABHBBAAHAABHHABHHHA

*000630_00187922 HHHBBABAHHHHHBBHBHBHHBBHABBHBHBABHBHAHHAAHBHHHBHBHBBHHABHBHAAHHHBABAHAAHHBAHBHHHHHHHHAHBHHAAHBHHABBAAHABBHHHAAAHHBABBAAHABHHHBHHHHBBBAAAHBHAHHBH

*000731_00071525 BHHBAHBBHBAAHAHHHHHAAAHABBHABHAHAABHHHAHBBBAAAHHHABAHHBBAHAHHHBHHHAHHAAABHAHAHAAAHAABHHHHHHHBABABHHABAHHHHABAABHAAHBBAHHHHAAHBHAHHHAHBHHHHBAHHHA

*000732_00075557 HHAHAHBHHBHABHHHHHABHAHHHBAAAHHAHHBAAABHHHABHBBHBHABHBAAHHHHAHBBAHHAHHBHBHHBHHAHHBHABBHAAAHHHHHHHHHAAABABAHAHHAAHBAABBHABBHHHHHABABHHHABBBBHHABB

*001080_00206533 BHHAAHHAABAAAABHBBBHHBHHHBAHHHHAABBHABBHHBHHHABBAHHABABBHAAHAHHHHHAAHHHHBHAHABHHHAHBHBHBBHAHBABBHHAHHHAHBABAHHHBHHHAHAHHHBHHAHAHHHBHBABABBAHHABB

*001465_00029060 BHABAHABHHBAHHHHBHHHAHBHBHAAAHHAAHHABBHHAAHAHBHHABBHABHHBABAAHHBAHABAAHAHABHHBAHBHBBABHHHBHAAHHABHAABBABBHBHHHHAHHHBHAHHHBBABAHHHHBAHHHHBHHBHHHA

*000073_00557675 HBHBHBHBAHBHBBBBHBHBHHBAAHAHHHHHHHHHHAHAABAHAHHHHHABHBBBHBHBHAAHHAABAHBHBABHAAHHBAHBHAHABBHHHHBAHBAHBBAHAHBHHHBAHAHBAABHHHHHHHABHBABHBH-HAABHAHB

*000718_00114779 AHBAHABAHBBAHABAAHABAHHHHHAHHHHHHHHHBHBHHBHHHHHBAAAAHHAABHHHHHHBBHHBHBAHBHBBBHBHHBHHHHHHABAHHBAHHHBHAHABAB-HHHBAHBABBBAAAHBABAHBBBAAHABBHHABHHHA

*000938_00275578 BBHABAHHAABBBHBBBAAHBHBHBAAHBHBBHAHBAHBHBHABHBHAHAAABAABHHAABBBAHHHHBHHHHBBHHHHHAAHAHHHAHHAHHBAHBAAHHBBHBHAHAHHBBHHAHHAHHHHBAHHHHABHBAH-HHHBHBHA

*001145_00114686 HAHHHBHBBBHBABHBBBHBHBHBHHHHAHABABHHBHAAHBHBHHHHAAHAHBBAAHHHBBBBAABBAAHBHAHHHAHHHABHBHHHHAAHHHBAHBBBAABAHAAHHBHHBHAAHHBHBHAHHHBBHAHHHHBAHBBHHBH-

*001246_00000658 HBAAABHAHBHAHBHHBBAHHHHHBBHHAAAHAHAHBAAAABHHHBBHHBBHAAHHBHBHHAHHBHBABABHBHBHAHHHHHHAAHHHBHHHBBBHHBHHBAHHBB-BBAABBBHHHHBHABBHABHBHHBBHHAHHHBHHBAA

*002873_00060907 AHBAHABAHBHAHABAAHABAHHHHHAHHHAHHBHHBHBH-BHHHHHBAHAAHHAABBAHHHHBBHHBHBAHBHBBBHBHHBHHHHHHAHAHHHAHHHBHAHABABBHHHBAHBABBBAAAHBAAAHBBBAAHAHBHHABHHHA

*003582_00052277 AHHHABAHAHHHBHAABHBBBHHHHBHAAHHABHBHBAHBHHBHHHBHAHHABAHHHHBAAHHBHBBBHBAABHHAHHAHHBBABHHBAAHAH-BHBAHBBBHAAHBHHHHHBAABABBABHHHBHHBAHAHHAABBHBHHHHH

*004753_00024863 BBAHBHBHHHBABAHBHBAAABHBAABHHHBHABABHHAAHHHBBBBAAABHBAHAAAHHHBHBHHHHAHHAHHHHAHBAB-BHHAHHHBABBBAAHHHAHHABAHAHABHHHHHHBHHHHAAAAHBHHHABHAAHHHAHHHBB

*000070_00364719 HHBHHBAHBBHHBB-BHBAABBBHHAAHAHHBBHABBABHBHABHBHAAHBBHHHHBAHHBHHHHABBAHAHBAAHBB-AHAHHBBBAHHHABHHHAHHHHHAHHHHBAHHHHAHAAHBHHBHBAHHHHAHABBHBBBHHBAHB

*000310_00038821 HHABHAHAAHHBAHAAHBHBBHHHHBAHAAHHAHBABAHBAAHHHBHHHHHHHHBHHAHHHABHABBABHBHHBHAAHHAABAHABHHHAHHAAABBAHBHBAHAA-HHBBHBHABHHAHHHHBBHBHAHABBH-BHBAAHBHH

*000637_00193371 HHABBHHHHBHA-AAHBHHHAHHBBBAABHAHHHHAHABHHHABBHBAAAHHBBAHHHAAHHBHHAHAHAHHBHHH-HAHHBAHBBAAAAHHABHABHBHAHHABHBHHAHAHBAHHBHABBHAAAAHHHHAABHHHBHHAABB

*006763_00002348 BHHAHHBHH-HHAHAAAHAAHHAAABAHAHHHBBHHHBBBAAAHAHHBHHBHHAAHHBHAHAAHHHHAAHABHHBBAHBHBHBHAHHAHHHAAHAHABHABBHBHH-HAAHBBBABBHHBBHHHBHAABBHBABHHHBHHHABB

*000752_00005903 HAHHAHAHHHABHBHABAHBHHHHH-HBHAHAHABHHABHBHAHHAHHAAABAAHHBBBAAHHHHABB-ABAHBAHAHHAHAHHHAABBBAHHAHHBBHHABAHHHBBBAAHHHHHABHAHABHBBABHBHHHBHBH-AHAHHA

*001134_00086983 HBAHHABABBH-HBBHBHHHAHBAHBBBHBAHBBHHB-HA-H-H-BAHABHHHHAAHBABHBHAAHH--AAB--HH-ABHHHAH--HBHHBAHBAHHBBBHB-AB-HHBHAH-HHB--HBH-HA-HHAH-BBHHA-A-HAHAHA

*000008_01085352 BHHBHHAHBHHABBBHHBHHHAABB-HHHABHABHAHHAHABHABBAAAABBHBH-HBBAHHHBHBHHHBBHBHHHABAHBH-BBHABHBHBHBHBAAHAHAABBH-HHBHHHHBBAHBHBHHHHHHHHABABHAAHHAHABBH

*000227_00278302 HHHBABBHABAABA-AHHHBBAHBBAHHHAHABAHHBHHH-HHHBHHHHHAAHBHBHHHAAHHHBAHBBBBAHHAH-ABABHBHHHBAHHAHHAAAHHAHHHABBHABHAAHAHHABHBBHAHB-AHHBBBABHAHBHHHBHHA

*001080_00198862 BHHAAHHAABAAAABHBBBHHBHHHBAHHHHAABBHABBHHBBHHABBAHHABABHHAAHAHHHHHAA-HHHBHAH-HHHHABBHBHBBHAHBABBHHAHHHAHBAHABHHBHHHAHAHHHBHHAHAH-HBHBABAB-AHHABB

*000215_00281382 AHHBHHABAHHA-BAAHBBHHHBBABBHHBABHABHHHAB-B-HBHHHAAAABHAHHHHHBAAHHHBHAAH-HAHBAHHHHA-BAAHHHHHAABAHBBBBHHHHAAAHBBBHBHABHAHABHHHHBAHBBAHAHHBHBHHHAHH

*000506_00188678 A-HBAHBBAHH-BB-ABHBHHHBHABBBBHABHABHHHABHBBHHHHHAAAABHHHHBHHBAAABHBBHABAHAHBAHHHH-HBHAHHHHAAAHHBBBHBHHHHHH-HBBBHBHHBBAAAHHHH-BAABBAHHHHAHAHBHAHA

*001321_00078664 ABHBAHBBAHB-HHAHBABHBHBHABBBBHHBAAHHHHABBBHHHBHHAHAAHHABBBAHBHAH-HBH-HAAHAAA-HHHBHHBHAHHHHHAAAHHB-H-HHHHHBAHBABHBHHBBAHAHHBBBBAAABHHHBHABBHHHAHH

*000720_00048282 AHHBBABBAHHBHAAAHHBHB-HAHBAHHABHHHHHHAHBAABAAHBHAHHABABBHHHAHBHBHABHHHHAHHAHAHHHHAAHBHABAHH-ABHAHHBHBBBHHH-AHBAHHHAABHAHB-HB-BAHHHHBBBA-HBH-ABHB

*000910_00168431 BBHABAHHAABHAHBBBAAHHHBHBHAHBHBBHAHBABBHBHABHBBAAAAABAABHHAABBBAHHHA-H-HHBBH-HHHAA-A-AHAHHAHHBAHBAAHHBBHBHAHAHHHHHHAHHHHHHHH-HHHH-BHBABAHHHHHBBA

*000033_00810690 AAHHAAHHHHAABBHAHBHBHHABBHHHHABBAH-BAHHBHHBA-HHAHBAHBAAHHABHBHBAHAHHAAABHAA--HA-BAAAHA-A-HAHAHHHHHHHHABAAH-HHHHABBAAHHAHBAHHBBHBHHAHHHHAH-HABBAB

*000074_00469048 HAABAHHH-AHBAHAHHBABBAAAH-HHBHAAHBAHHHHBBAAHAAHBHHHHABABHHHHABHHHHAHBHBBBAHA-ABAHB-B-BHHHHBBH-ABHHBHBABABH-HBAHHAAHAHHHHAHHBHAAHABBHH-BHHAHHHHB-

*003273_00069873 -AHABAHHB--HABBBHHHHAHBA-BHBHHHHHABHHAHHHBBBAAHHHBBAAHBHHHHAHABABHHBHHB-AAHHABHAHAABAAH-AHAHAHAHAABHHH-BHAAAHBHHBHHHHBAHBAHA-BHA-B-HABHAHHAHHHBB

*000056_00400718 HBHHHBAHAHAH-H-HBABHHBHAAABHHHABAHHBBHAAHBHHHABABAAHBBAHAHHABBBHHHAHAHBHHBHAAAHHBH-HHBH-AHBAA-HHHHHBHHHAA--BHHHBAB-BBH-AHHAHBHHBAAAHBHA-B-HBHBHA

*000291_00041159 B-AHAHAHB-H-AA-BBHAABHHHA-AHHHHAHBHHAB-BHAAAHAABHHHHABABHBHBABHHBBHB-AHHBHAH-AHHHBBH-AHHHBBBHAAHHABHBHHAHBHHBA-HHAHAAHABHAHBAAAHHBHBBAHBHHH-HHHB

*000090_00466833 HBHHHHABHHB-HHBHHH-AAHBAH-AHHHHHH-HAHA-A-BHHAHHHHHABHHHBBBAHHHHHBHAB-BBABAHBAHHBB--BHAAAHBHBH-AAHHAABHAHHBBAHBBAHAHAA-HBHHBHAHAABBAHHAB-H-AAAHAB

*000624_00308853 ABHBABHAAAHHABHHHBBBHHHHHHABHAAHBBHAHAHAAHHHHAHAHHHBHHBBHBBHHHABHHAHHBBHBABHHHHBBHHHAAAABAHBHBBHAHHBHBHHHBBBHBBABABHAABHBHBHHHHBHBHBHBAHHBABAAHB

*002792_00087997 HHAAHHHBBBA-HB-BHHABAHBHH-HHAHHHAHAHHBH-BH-HHAHAHHABHHBHHHAAHABAHABH-HBBHAAHAHHHAABHAHAHBAAHHHA-HAHBH-AAH--HAAHAHABBAHHBBBHABBHBB-HHH--HH-BBABBA

*000352_00236841 HAHBHBBH-AHBBHAHAHHABHB-B-HH-AABBBBBHHAABB-HBHHHBABHBHBAHHHBBBBHABAH-H-AAABB-BHBBHHHAHHHAHHHH-HAHHHAHBHBHA-HAABAAHH-BABBHBHH-HBH-AHHAH--HAHHHAHH

*000861_00150312 AHHBAHBBA-BH-HAHBHBHBBHHA-BBBHHBAAHHHBABHBHHHBBAAHAA-HAHBBAHBHAHBBBHBHAAHAAAAHHHB-ABHAHHHHHAA-HH-AHHHHHHHB-HHABAHHHHBABAHHBB-BAH-BH-HH--B-HAHAH-

*002818_00060796 ABHBHHBHBAA-HBH-BHHAHAHAAAAB-BHHH-HHAAHHBHABABHBHBBAHHHAAAHBHBBAAHHH-A--AHBHBAHHBHAH--HHHABBB-BBHBBAHB-AHB-HHHAHHBHHHHHHHH-HAHAHH-AABH-BA-AAHAAH

*000000_01352675 HAHHBHHHBBHBABHBBBHBHHBAHHBHAAABHBHBBHHAHBHHABHHAAHAHHHAAHHHBBBBHABBAABBHAHHBAHHAABHHHHHBAAHHHBAHBBBAABABAAHHBHBHHAAHHHHHHAHHHBBHHBHHHBBHBBAHBHH

*000000_01416024 HAHHBHHHBBHBABHBBBHBHHBAHABHAAABHBHBBHAAHBHHABHHAAHAHHHAAHHHBBBBHABBAAHBHAHHHAHHAABHHHHHBAABHHBAHBBBAABABAAHHBHBHHAAHHHHHHAHHHBBHHBHHHBBHBBHHBHH

*000155_00631368 AHBBHBAABBHHHHHHBBAHHHAAHHHHAABHHHBHHHHBAAHAABBBBHBHBHHBHHBBHHAAHBBBBBAABHAHABBHAABHHHAHAHHHHAAHBHAHHABHAAHAABBHBHAAAHBHBHHHBBHHHBAHHAHHBHHBAHHH

*000180_00599847 AHBBHBAABBHHHHHHBBAHHHAAHHHHAABHHHBHHHHBAAHAABBBBHBHBHHBHHBBHHAAHBBBBBAABAAHABBHAABHHHAHHAHHHAAHBHAHHABHAAHAABHHBHAAAHBHBHHHBBHHHBAHHAHHBHHBAHHH

*000223_00143945 AHBBHHAABBHHAHHHBBAHHHAAHHHHAAHHHHBHHHHBAAHAABBBAHBHBHABHHBBHHAAHBBBABAABAAHABBHHABHHHAHHHAHHAAHHHAHHABHAAHHABBHBAAAABHHHHHHBBAHHBAHHAHHBHABAHHH

*000231_00455765 BAHHAHAAHAHABHHBBHAHHHBBHABAHAAABHHHHHBHHHHHBBBAAHHHBHAABHBHABHHHHBHHABAAAHBHBBHHBHBHHBAAHHHBHHHAHHBBHAHBAABAHABHAHAHAHAAHBHHAHABHBBHBAHHHAABAHB

*000413_00463923 HAHBBHAHABBHBAAAHHAHBHHBAHAHHAABHAHBAAABHAAHBHHAAAAHHBAHBHAHBHAHHBAHBBBHBHBBABBABHABHHHAHHBHHHHHAHBAHHBBBHHHHAHBHHABAHHHBBBBBHHABHBHHHHHBHHHHHBH

*000492_00307724 HBAHHBBHAAAHHAHBHHHHBBHHBHBHAHHHABBBHBABHBBAHAAHHHHHHAAABBHHHBHHHBAHHHHHBHHHAHBABABHHAHHAHBBABBHHHBAHHHAAHABBHHBHHAHBHBABHBBHHHBHAAHAAABHBABHBBA

*000563_00180711 AHABHABHAHHBBAHHBHBBHHHAABABHHHHHBHHBBHBAAAHAHHAHBAHHHABHBHBHAHBAHBHHHHABABAHAAHAHHHHBAHHBBAAAHHAAHAHAABBHHHHAHHHABHHAAHHHHHHBAHABBHHHAAHABHAAAA

*000578_00300180 BBBHAHBHHHBHBHBAHBBBAHHBABHBBAHBBBHAHBBHHHHABBHAHAHHHHBBHHHBBHHHAHHHBHAAHBHHHBABBHBABHABAAHHHHBAHBAAAHHHAHHABHHHAHHHHHHHHHHAHHAAHBAAHBAABABAABBB

*000614_00049193 AHBAHAHHHBHBBABAAHABAHHHHHAHHHABHBHHBHBHHBHHHAABAHAAHHAAHBAHBHBBBHHBBBAHHBHBBBBBHHHHAHHBAHAHHHAHHHBAAHABHBHHHBBAHBABHHAAAHHAHAHHBBHAHAABHHABHHHA

*000811_00208876 AHHHBBBHHAHHBHHBHHHAABHBHBABBABAAHHHHHAHHBHBBAHAABHHAABBAAAHAHBHAHAHBBAHHHHAHBHHHBHBHBHHHHBAHHBBHBHBBHAABAAHHHBHHABABHBHAHAAHAHAAHBHBAHHHBBHHBHH

*000826_00015483 BBBHHHBHHHBABHBAHBBBAHHBABHBBAHBBBHAHBBHHHHABHHAHAHHHHBBHHHBBAHHAHHHBHAAHBHHHBABBHBABHABAAHHHHBAHBAAAHHHAHHABHHHHHHHBHHHHHHAHHAAHHAABBAABABAABBB

*001291_00041924 HBAHAHHAHBHHHHHHBBABHHHHBHHHAAHAAAAABAAAABHAHHBHAHAHAAHHBHBBAAHBHHHAHABHBHBHABHHBHHAAAHHBHHHBBBBHBHHBAHHBBABBAABBHHBHHHHABBHABHBHABHHHAHHHBHHBHA

*001488_00002062 HAHHBHAHBBHBABABBBHBHHBAHHBHAAABHBHBBHAAHBBHABHHAAHHHHHAAHHHBBBBHABHAAABHAHHBAHHAABHHHHHBAABHHBAHBBBHABHBAHHHBHBHHAAAHHHHHAHHHBBHHBHHHBBHBBHHBHH

*002042_00101907 BBAHBHBHHHBAHAHBHBHHAHHBAABHHABHABABHHAAHHHBBBBAAABBBAHAAAHHHBHBHHHHAHHABHHHAHBABHBHHAHHHBABBBHAHHHAHHABAHAHABHHHHHHBHHHHAAAABBBHHABBAAHHHAHHHBB

*002243_00100840 BBBAHHBHHHBAHHBAHBBBAHHBABHBBABBBBHAHBBHHHHABHHAHAHHHHBBBHHBHAHHHHHHBHAAHBHHHBABBBBABHABAAHHHHBAHBAAAHHHAHHABHHHAHHHHHHHHHBAHHAAHHAABHAABHBAABBB

*002451_00037918 AHHHABAHAHHABHAABHBBBHHHHBHAAHHABHBHBAHBHHBAHHBHAHHABAHAHHBAAHHBHBBBHBAABHHAHHAHHBBHHHHBHAHAABBHBAHHBBBAAHBHHHHHBAHBABBABHHBBHHBAHAHHAAHBHBHHHHH

*003445_00009258 BBHBHAAABBBHHABHAHAHBABHHHAHHHAAHHABBBHHHHHHHHBHHAHBBAHABHBBAHHHBHAABAHHBAHHABBHHAAHBHHBBHAHBAHABHAAHHBBHHHHBBHHAHAAAAAHHHBBBHABHHHAHHBAABHBHAHH

*003647_00020742 HAHBAABAHHHAHBHHHBHBBBBHBBHHHHHBBBBHAAHBAAABHAAHHAHHBBHBBBHHBHHHAABHHABHAAHBBAHBBBHAHAHBBAAAHHHAHHBHBHHBHHHHAAAHAHABHHAHHHBBAHHHBHHHBAHHAHBBHAHH

*004470_00008973 AHAHHBHHAHHBBBAHAHHHBHBBBAHHHAABBBBBHHAHBHHHBHAHBABBBHBAHBBHBBBBABAHBABAAHBBBBHBBAHHAHHAHAAHHAHAAHHHHBHBHAAHHHBAHHHHHABHBBHBHHBHBHHHAHHHHHHHHABH

*005267_00008341 AHHHABAHAHHHBHHABHBBBHHHHBHAAHHABHBHBAHBHHBHHHBHAHHABAHHHHBAAHHBHBBBHBAABHHAAHAHHBBABHHBAAHAHBBHBAHBBBHAAHBHHHHHBAABABBABHHBBHHBAHAHHAAHBHBHHHHH

*009002_00006828 HAHBAAAHHAAAHHHAHHHBBAHBBBHHHABBAHHBAHHHAHBABBHAHBHHBAAHHHBHBHBHHAAHAHABAHAHAHABHAAHBHBHHAAAABHHHAHAHABHHHBHHBHHHHAAABAHHAHBBBHHHHAHAHAHHHHABBAB

*000034_00339810 AHHBABAHAHHHBHHABHBBBHHHHBHAAHHABHBHBAHBAHBHHHBHAAHABAHAHHBAAHHBHBBBABAABHHAHHAHHBBHBHHBHAHAHBBHBAHBBBHAAHAHHHHHBAABHHBABHHBBHHBAHAHHHAABHBHHHHH

*000226_00465376 ABBABAHHHAHBAAHHHBABABHHBHBHHHBBBAAAAAHAAHBHHAAHBHAAABAHBHHBBHBBHAAHHAHAHHHABHHBBHAHABBHHBHHAHHAAHHHHHHHHA-BHBHHAHBAHHAAAHBAHHHAABHBAHHHBHHHBHBH

*001626_00092240 ABHBHHBHBHA-HBHHBHHAHAHAAAABBBHHHBHHAAHBBHABABHBHBBAHHAAAHHBHBBAAHHHAAHAAHBHBAHHBHAHAAHHHABHBAHBHBBAHBHAHBBHHHAHHHHHHHAHHHAHAHAHHHHABHBBAAAAHAAH

*000933_00012027 HHHHHHHHHAHH-H-BAHAHHHABBBBHAAHA-HBBAA-HHHABBHHHBH-AHBA-BHBBAHBBHHBHHAA-HBAB-HHHBHBHBAHA-BHAH-AHAAH-HHHHAHABHA-BBAAHH-H-BABHAHHBBBAAHABAB-BHHH-H

*000006_00760735 HHHHAABHBBAABHBHHBHHHHBBHHBHABBBHHHBHAAAHHBHBHAHHAAHAHHAHBHBBAHHBHAHHHAHBHAABBHAAHBAABBA-HBBHHABAHHAHHBHHBBAAHHAHBAHBAAAHHHHBHHAAHHABAHAHHHHBAHH

*000403_00382265 BHHAAHHAABAHAHBHBBBHHBHHHBABHHHAHBHHABBHHBBBHABBAHAABABHHAAHAABHHHHAHB-HBBAHBBHHHAHHABHBBHAHHABHAHAHHHHHBAAAHBHBHHHHHAHHHBHHAHAHHHBBBHBABBAHHABB

*001365_00093868 HHAAAHHABBHHABAHBHAHAHHHHBAHAAAAHHAHBAAAABHHHBHBHBBBAAHHBHBHAAHHHHBA-HBHBHHHAHHHHHAAAHHHHHHBBBBHBBHHBAHHHBHBBAABBBAHHHBHABBAAHHBHHHBBHAHHABHHHAA

*000484_00366185 HAHHHHHHBBHBABHHBBHBHHBABHBHAAHBHBHBBHAAHBHHABHAHAHHHHBAHHHHBBBBHABAAAHBHAHHBAHHAABHBHHHBAABHABABBBHAABABHBHHB-BHBAAHHAHHHHHHAHHHHBHHHBBH-BBHBHA

*000539_00269072 BAHBAHAHHHAHHHHABAABABBHBHHAHHHAHBHABABHBHHAHABHAHHHAAAHBABAAHHHHHBBAABAHHAHABHAHAHBAHAHBBAHAHBHBBHAABAHBHHHHAAHHBHBABHHHBBAHBHHH-BHHBHHH-HHAHAA

*004844_00001998 AHHBHHABAHH-BBAAHBBAHHHBHBBBHBABHABHHHABHBBBBHHHAAAABHHBHHHHBAAHHHBHAAHAHABBAHHHHABBHAHHHBHAABAHBHBBHHHHAHAHBBHHBHABAAHABHHHHBAHBBAHAHH-HBHHHAHH

*000294_00273473 AAHBHABBAHHA-AAAHHBHBAHAHHAHHABHHAHHHAHBAABAAHBHAHHABABBHHHAHBHBHABHHHAHHHAHAHAHHA-HBHABAHHAABHAHABHHBBHHHBAHBHHHHAA-HAHBAHBABAHHHHBBHAAHBHHABHB

*000538_00151671 AABAHABAHBBA-ABAAHABAHHHHHAAHHHHBHHHBHBHHBHHHHHBAAAAHHAABHHHHHHBBHHBBBAHBHBBBHBHHBAHHHHHABAHHHHHHHBHAHAHAB-HHHBABBHBBHAAAHBABAHBBBAAHAB-HHABHHHA

*001195_00094665 H--B-HBHBAHHHA-BHABAHA-HA-AHBABB--BHBBAHHHHH-HAHHBHHHHABBHB-BAHA-BHHAHHHA-H--B--B-HBBHB--H----HBBHB-HHAAHBBBHHBHBA--BHB-BH--BBHB-BHAHHA-HHHB-H--

*000215_00263833 AHHBHHABA-H-BBAAHBBHHHBBABBHHBABHABHHHABHB-HBHHHAAAABHHBHHHHBAAHHHBAAAHAHAHBAHHHHAHBHAHHHAHAA-AHBHBBHHHHAHAHBBBHBHABAAHABHHHHBAHBBAHAHHBHBBHHAHH

*002100_00013349 BBHABHHHAAB-HHBBBAAHHHBHBAAHBHBBHAHBABBHBHABHBBAAAAABAABHHAABBBAHHHA-HHHHBBHHHHHAABABAHAHHAHHBAHBAAHHBBHBHAHAHHHHHH-HBHHHHHHAHHHHHBHBAHAH-HBHBBA

*006398_00006953 HAHHAHAHHHB-BHAHHBBBHHHAHBHHHBHABHBHBABHHHHAHHBHAHBABAHHHBBAAHBBHBHB-HAAHHBA-HAHBHAAHABHAAAAHHBHBAHHBHBABHBHHHHHBBABHHHHBHHHHHBAABBBHHABB-BHBAAB

*001128_00068338 ABHHHHAAAHHAHHHHBHHBHAHHBBBAAABHABAHBAABBHBHHABHAHHHBHBHAHBAHBABBHBH-AHHHAHAABAAABBH-BHBBHHHH-BHBHHHHBHHAAHHBHHBAAABAHHABAAH-HBBA-AHHAHHBAHHHHHH

*002490_00122891 ABHBHHBABHABHBHHB-HAHHHAA-ABBBHHHBHHA-HABH-BABHB-BBAHHHAAHHBHBBAAHHHAAAAAHBHBAHHBAAHAAHBHHBHHAHBHBHAHBAAHBHHHHAHHHHBBHHHHBAHAHHHHHHBBABBAHHABAAH

*000783_00187588 HBBHHBHAHHABHB-ABBBHABH---HHBHHHAAHHH-HH-H-ABA-BAAHBHBHAAHBHHHBHAHB--AHA-HAHHHHAHABB-AHAHBHAA-HHAABBAAHHHA-H-HHAHHBHABBB-HA-B-BBA-HHBAH-B-HBHHBH

*006615_00023056 HHHBHBBHHHHBBBAHAHHAHHBBBAHHHAABBBBBHAAHBBHHBHHHBABHBHBAHHHBBBBBABAHBABAAHBB-BHBBAAHAHHH-AHHHAHAHHHAHBHHHAAHHHBAHHHAHABBBBHHBHBH-B-HAHHHH-HHHAHB

*000464_00447401 HABABAHHBHBHBHBBHHAHAHBAABHBHHHHHABHHHHHHHBB-AHHHBBAAHHHBHHAHABABHHBHBHHAAHB-BH-BBABAAH-BHAHABHHAABAHHBBHHAAHBHHBHB-HBAHBHB-HBHABBHHABHAHHAHHHBB

*000006_00561661 HHHHAAHHBHA-HHBHHB-HHHBBH-BHABBBHAHBHAAAAH-HBHAHHAAHABHAHBHBBAHHBHAHHHAHBHAAHBHAAHBAABBAHHBBHHABAHHAHA-HHBBAAHHAHBAAAHAAH-HHBHHAAHHABAHAH-HHBHHA

*002186_00114597 AHHBHHABAHHABBAAHBBAHHHBHBBHHBABHABHHHABHBBHBHHHAAAABHHHHHHHBAAHHHBA-AAAHABBAH-HH-HBBAHHHBHAABAHBHBBHHHH-B-HBBHHBHABAAHAB-HH-BAHBBAHAHHBH-HHHAHH

*006806_00010603 HABHHBHHH-ABBHBHBHHHHBH-AAHAHBHHAHHBBHAAHHBHHABH-AAHBBAHAHAAHHBHAHAA-HBHHBHAAAHHBBBHHBHBHHBAHBHHHBHHHBHAAA-BAHBBABABAHBHHAAHBHHBAA-HBHH-B-HBHBHA

*000261_00441437 BAHAAHHAHBAA-A-HBBBBHHHBHBAHHHHAABHHABBHHBHHBHBHAHHHBABHHAABAHHHBHAA-H-HBAAHABHHAA-BABHBBHABH-BBHAAHBHHHBAHAHHHBHHHAHA--HHHHHHAHHHBHBABABBAHHABB

*000480_00060299 -AHAAHHH-HB-AB-HAHAHA-AHH-HAHA--BBHAAHBH-HHAHAHAAHHAABA-HH-ABAHBAHHA-HAAHABHAAHHHB-BBABHHBHHH-BHH-B-AHHHA--HAHHABHH--BHBHHBA-HHHH--HBB-AA-B-BAAB

*000040_00379341 AHHAH-AHHHAHHBH--BBHABBBB-AABAABAAAHAHHHAH-AHHHBAAHHBBB-ABAHBBBH-HHBAHAAHBHHBHAHH-BHBHHAAHHAHHAHAABHHAHHHAAHBHHAHBHAA--BHHAAAHHHB-BHBHHBH-HBHAHH

*001516_00241160 ABABHHBH--A-HBHHBHBAHAAAA-ABBBHHHBHHA-HHBH-BABHBBBBAAHHAAHHBHBBAAHHHAAAAAHBH-AHHBHAHHAHHH-BHB-HBHBBAHBHAHB-HHHAHHHHBBHHHHH-H-HAHHHHAB-BBAAAAHAAH

*000015_00907479 ABHBBHHHHHABHBBHBHHHAHHBHBHHBHHAHAHHBHHBHBHHBABAABAHABHHBHBHABBHHBHAHHHABBAAHAHBBHHBHHHAHHBHBHHBAHBHHBBAAABBBHBAHHAAHHHAHABAHHHBAAAHABHHBAAHHABA

*000160_00310522 ABHHHBHHHHHBHHHHHABAABHHHBHBBBHHABHAAHHAAAHHHBHHBAHHHABHHAHHHBHHBAHHHBBHBABHBHHABHHAAHBAHAAHBBBBHABBAHAHBBBHAAHBBBAHBHBAHHHAHAAAHHBHHAABHABBBHHB

*000393_00251696 AAHBAAHHHHAAHHHAHBHBBHHBBHHHHABBAHHBAHHBAHBABHBAHBAHBAAHHABHBHBHHAAHAAABAAABHHABHAAHBHBHHHAHABHHHHHHHABHHHBHHBHAHBAHHBAHBAHBBBHBBHHHAHHAHHHABBAB

*000452_00328759 AABHABAHABHHAHHBHHHHBHBHAHHHHHBHHABABAHAHBHAHABHHAAABHHHHAAHHBBBAAAHBBBBAHHBAHHBHHHHBAHHABHBBAHBABBBBHAHHHHBBAABAHHHHABBHAHBBAAHBHHBHHHBHAHHHHBH

*000531_00250501 AABHAHHHABHHAHHBHBHHBHBHAHAHHBBHAHBABHHHHBBAHHBAHAAHBHHHHHABHBBBAAAHBBHBAHHBHBHBHHHBBHBHABHBHAHBABBBBAAHHHABHHHHABAHHAHBHAHBBAAHBBHBHHBBBHHHBAHH

*000636_00212492 ABHAHHHHBHHBHHBHHABAABHHHBHBBBHHABHAAHHAAABHHBHHBAHHHABHHAHHHBHHBAHHABHHBABHBHHABHHAAHBHHAAHBBBBHABBAHAHBBBHAAHBBBAHHAHAHHHAHAAHHHBHHAHBHABBBHBB

*000663_00123574 ABHHABHHHHHBBHHHHABAABHHHHHBBBHAABHHAHBAAAHHHBHHBAHHHAHHHHHHABHHBBBHHHBABABHBHBHBHAAABBAHAAHBBHBHABBHHAHBHBHAAHBBBAHHHBHBBHAHAAAHHBAAHABHAHBBHHH

*001128_00131562 ABHHHHAAAHHAHHHHBHHBHAHHBBBAAABHABAHBAABBHBHHABHAHHHBHBHAHBAHBABBHBHAAHHHAHAABAAABBHHBHBBHHHHBBHBHHHHBHHAAHHBHABAAABHHHABAAHBHBBAHAHHAHHBAHHHHHH

*001179_00010971 ABBABAHHHAHBAAHHHBABABHHBBBHHHBBBAAAAAHAAHBHHAAHBHAAABAABHHBBHBBHAAHHAHHHHHABHHBBHAHABBHHBHHHHHAAHHHHHHHHAABHBHHAHBAHHAAAHBAHHHAABHBAHHHBHHHBHBH

*001438_00055320 ABHHABAAAAHAHHHHBHBBBAHHHBHAAAHAHHHHBAABHHBAHHBHAHHABABHAHBAAHHBBBBHBBAABHHAHHAHHBBHHHHBHAHAABBHBAHHBBBAAAHHHHAHHAHBHHBABHABBHHBHHAHHHAABHBHHHHH

*002875_00008143 ABHHHHHAAHHAHHHHBHHBHAHHBBBAAABHABAHBAABBHBHHABHAHHHBHBHAHHAHBABBHBHAAHHHAHAABAAABBHHBHBBHHHHBBHBHHHHBHHAAHBBHABAAABHHHABAAHBHBBAHAHHAHHBAHHHHHA

*000007_00897244 BHABAHABHHBAHHHHBHHHAHBHBHAAAHHAAHHABBHHAAHAHBHHABBHABHHBABAAHHBAHABAAHAHABHHBAHBHBBABHHHBHAAHHABHAABBABBHBHHHHAHHHBHAHHHBBABAHHHHBAHHBHBHHBHHHA

*000029_00523925 HHBBHHAABBHBHHHHBBABHHHHBHHHAABHHHBHHHHBAAHAHBBHBABHBHHBHABBHHAAHBBBBBAABBAAABBHAHAHAHAHHHHHAAAHBHAHHABHAAHHAHBHBHAAHHHHBHHHBHBHAHAHHAHHBAHBABHB

*000118_00099216 HHBBBABAHHAHHHHBHAHAHHBHHHBAHBBHBHBAHHHHHHBHHHBHBHBBHHABHBBAHHBBBABABHAHHBABBAHBHHHHBAHBHHHABHHBABBAAHABBHHHAAAHHHABBAAHABHHHBHHBHBBBHAABBHHHHBH

*000118_00217503 HHBBBABAHHAHHHABHAHAHHBHHHBAHBBHBHBAHHHHHHBHHHBHBHBBHHABHBBAHHBBBABABHAHHBABBAHBHHHHHAHBHHHABHHBABBAAHABBHHHAAAHHHABBAAHABHHHBHHBHBBBHAABBHHHHBH

*000249_00413785 BHBBHBHHBBBHBABHHBHABHAHHHBAHBBBHHHABHAHHBHHHHBHAHBAABHAHHHHBHHHHABHHHHABABHAABABHBHHAHHBBHBBBHABHBHBAABAAAHAHHHAHHBHHBBBHHBBHBBHHABHAHHBAAAHBHH

*000416_00043643 HHHBBHAHABBBBAAAHHHHBHHBAHAHHAABHABBAAABHAABBHHAAHAHHHAHBBAHBHABABAHBBBHHHBHABBABHABHHHAHHBHHHHBAHBAHHHBBBHHHAHBHHHHAHHHBBBBBHHAHHBHHHHHBAHBHHBH

*000423_00077725 BHBHAABHBHAAHBBHHBHHHHBBHHBHABBBHABBHAAABHHHBHAHHAAHAHBAHBABBHHHHBAHHHHHBHAABHHAAHHAABBAHHBBHAABAHHHBHBHHBBAAHHAHBAHBAAAHHHHBBHAAHHABAHAHHHHBHHH

*000448_00092579 HHBAHAHHHBHBBABHAHABAHHHHHHAHHABHHHABHBHHBABHAABAHAAHAAAHBAHBHBBBHBBHBAHHHHBHBBBHHHHHHHHAHAHBHAHBHBAAHABHBHHHBBAHBABHBAAHHHABHBHBBHABAABHHABHHHH

*000449_00103824 BHHBAHABHHBAHHHHBHHHAHBHBHAAAHHAAHHABAHHAAHAHBHHABBHABHHBABAAHHHABABAAHAHABHHBAHBHBBABHHHBHAAHHABHAABBABBHBHHHHAHBHBAAHHHBBABAHHHHBAHHHHBHHBHHHA

*000476_00153849 HHABABBBAHAABAHHHHHBBABHBAHHAHHABAHABHAHBHHAHHHABHBAHBHBHHHAAHHHHAHBBHAAHHABAAAABHBHHHBAHHAHBAAAHHAHHHHBBAHBHAHHAHHHHABBHHHBHHHHBBBABHAHBAHBBHHA

*000512_00114234 BHABABBBHBAABAHAHAHBHABABAHAHHHHBABABAAHHHHAAAHHBHBAHBBBHBHAHHBHHHHBHHAABHABAHAAHHHHHHBAHHHHBAHAHHHHHAHHBHHBAAAHAAHHHABHHHHHBBAHBHBABBAHHHHBBBHA

*000530_00158690 HHHBBHAHABBHBAAAHHAHBHHBAHAHHAABHAHBAAABHAAHBHHAAAAHHBAHBHAHBHAHHBAHBBBHBHBBABBABHABHHHAHHBHHHHHAHBAHHBBBBHHHAHBHHHBHHHHBBBBBHHABHBAHHHHBHHBHHBH

*000630_00189058 HHHBBABAHHHHHBBHBHBHHBBHABBHBHBABHBHAHHAAHBHHHBHBHBBHHABHBHAAHHHBABAHAAHHBAHBHHBHHHHHAHBHHAAHBHHABBAAHABBHHHAAAHHBABHAAHABHHHBHHHHBBBAAAHBHAHHBA

*000998_00054056 HHABABBBAHAABAHHHHHBBABABAHHAHHABAHABHAHBHHAAAHHBHBAHBHBHHHAAHHHHHHBHHAAHHABAHAAHHHHHHBAHHHHBAHAHHAHHHHBBAHBHAHBAAHHHABBHHHBHBAHBBBABBAHBHHBBBHA

*001082_00184200 HHABBHHHHBHABAAHBHHHAHHBBBAABHAHHHHAHABHHHABBHBAAAHHBBHHHHAAHHBHHAHAHAHHBHHHHHAHHBAHBBAAAABHABHABHBHAHBABHBHHAHAHBAHHBHABBHAAAAHHHBAAHHHHBHHAABB

*001164_00132177 BHABABBBHBAABAHAHAHBHHBABAHAHHHHBAHABAAHHHHAAAHHBHBAHBBBHBHAHHHHHHHBHHAABHHHAHAAHHHHHHBAHHAHBAHAHHAHHAHHBHBBAAHHAAHHBABHHHHHBBAHBBBABBAHHBHBBBHA

*001416_00056397 BHBHHAHHHBHBHAHHAHABAHHHAHHAHBABBHHHBHBHHBABHAAHAHHAHAAAHBAHBHBBBHHBHBAAHHABHBHBHHHHHABHAHAHBHAHBHBAHHABHBHHHBBAHBABHBAHBHHAHHBABBHABAABBHABBHHH

*001526_00143224 HHHBBHAHABBBBAAAHHHHBHHBAHAHHAABHABBAAABHAABBHHAAHAHHBAHBBAHBHAHABAHBBBHHHBHABBABHABBHHAHHBHHHHBAHBAHHHBBBBHHAHBHHHHHHHHBBBBBHHAHHBHHHHHBAHHHHBH

*001896_00157570 HHHBHAHBAAHHHAAAHHBHBAHAHBAHHABBHHHHAAHHAABAABHHAHHHBABBHHHABBHBHABHHHAHAHAHABAHHAAHBHAHAHHAABBAHABHHBBHHHBAHBHHHHAAHHAHBAHBHBAHHHHBBBAAHBHHABHB

*001911_00038823 HHHAHHAAAHBHBAHHHHHHHHHBHHHAHAAHHHBBAABBBHABBHBAAHAHHHAABBHHHHHBABBAHBHAHHBHHBHABAABHHBAAHBBBHAHABBHBHHBBBAHHHHBHAHHAHHHHBBHBHAAHBBHBAAAHHAABHBH

*003915_00005980 BHBHAHBHHHHHBHBAABBBAHHBABHBBAHBHHHAHBBHHHHABBHAHAHHHHBBHHHBBHHBAHHHBHAAHBHHHBABHHBABHABAAHHAHBHBBAAAHHHAHHABHHHAHHBBHHHHHHAHHAAHBAHABAABABAABBB

*000109_00125504 AHHBABBHAHAAHAHHBBHBHHHBHABBBAHABBAHBHHHBHBHBHHHAHAHHBHBHHHAAHHBHABBBBBHHHAHAABABABHHHHA-HAHAAAAABAHABABBHHHBHHHHHHABHBBHHHHHAHAHBBABHAHBBHHHHHA

*000425_00221266 BBHABAHHAABBHHBBBAAABHBHBAAHBHBBHAHBABBHHHABHBHAAAAAHAABHHAABBBAHHHHBHHHHBBHHHBHAAHABHHAHHAHHBAHBAAHHBBHHHAHAHBBBHHAHHAHHHHB-HHHHHHHBABAHHHBHBHA

*000550_00194372 HBBHHHBHHHBHBHBAHBBBAHHBABHBBAHBHBHAHBBHHHHABHHAHAHHHHBBHHHBBHHHAHHHBHAAHBHHHBABBHBABHABAAHHHHBAHBAAAHHHH-AABHHHAHHAHHBHHHHAHHAAHBAABBAABABAABBB

*001002_00242869 BBHABAHHAAB-HHBBBAAHHHBHBAAHBHBBHAHBABBHBHABHBHAHAAABAABHHAABBBAHHHHBHHHHBBHHBHHAAHAHAHAHHAHHBAHBAAHHBBHBHAHAHHBBHHAHHAHHHHHAHHHAHBHBAHAHBHBHBHA

*001337_00193817 AHBAHABAHBHAHABAAHABAHHHHHAHHHAHHBHHBHBHHBHHHHHBAHAAHHAABBAHHHHBBHHBHBAHBHBBBHBHH-AHHHHHABAHHAAHHHBHAHABABBHHHBAHBABBBAAAABAHAHBBBHAHAHBHHABHHHA

*002296_00094036 AHHHABAHAHHBBHHAHBBBBBHBHBHAAHHABHBHBAHHHHBHHHBHAHHABAAHHHBAAHHBHBBBABAAHHBA-HAHHBBABHHBAAHAHBBHBAHBBBHAHHBHHHHHBBABABBABHHBHHHHAAHHHAAHBHBHHHHH

*002320_00122466 HAHABAHHBABHHBHBHHAHAHBAHBHBAHHHHAHHHHBHHHBBAAHHHBBAAHHHHHHAHABABHHBBHBHAAHBABHAHHABAAHAHHAHAHABAABHHHBBHH-ABBHHBBHBHBAHBAHAHBHABBHHABBAHHAHHHBB

*000080_00274263 ABBABABABHBA-AHAAHHBAHBHHHAHHAHHAHBHAAHHHAAHHHHHAAHHHHAABHHHHHHHHHHHAHABBHBBBABHHBBHHBHH-BAHAAHHHHBBAHAHHBBHHHHABBBBBHAAABBABHHBBBHAHABBAHABBHHH

*000792_00025961 AAHHHHHHBBHBABAHBBHBHHBABHBHAAHBBBHBBHAAHBBBABHAHAHHHHHHHHHHBBBBHAHHAAHBHABH-AHHAABHBHHBBAABHHBHBBBHAABABBHHHBABHBAHAAHHHHHHHHHHHBBHHABBH-BBHBHH

*000228_00545746 BHHHHBAAHHBABA-BBHHAAAHHA-AHHAAHHBHAAHHHHBAHHHHB-AHAHBAABHHBHHBHABAH-H-AH-BH-HABH-AH-BHHHBBHAAHHAAHAHBAAHBBBHBBHHHHA-H-BABBA--AHHB-BHAHAH-HAHHBB

*000860_00020320 HHHHAAABHHABHBBABAHBHHHHHHHHHAHAHABHHABHBHAHHAHHAAABAABHBBBAAHHHHABB-ABHHBAHAHHAHA-HBAABBBABHAHHBBHHHBAHHHHBBAAHHHHHHBAAHABHHHBBHBHAHBHBHBAHAHHA

*000933_00199914 HHHHAHHHHAHHAHHBAHAHHHABBBBHAAHAAHBBHHHHHHABBHHHBHBAHBAABHBBAHBBHHBHHAABHBA--HHABABHHAAAABHAHHAHAAHHHHHHAHAHHAHBBAAHHHHABABHAHHBBBAAAABABABHHHHH

*001166_00070228 HHHHHHHHHAHBAHBBAHAHHBABBBBHAAHAAABBHHHHHHABBHHHBHBAHBAABHBBAHBBHHBHHAABHBAB-HHHBHBHBAHAABHAHHAHAAHHHHHHAHAHHAHBBAAHHHHABABHAHHBBB-AHABABABHHHHH

*000608_00136891 HBHHHHAABBBHBHAHHBBBHHHAABHHHBHABHBHHHBH-BHAHHBHHHHABAHHHBBAAABBABHHBHAAHHBAABAHBH-HHABHHAHHHHBABAHHBHBABA-HBBHHBBABAHHHHHHHHBHAABBBHAHHBHBHBAAB

*000169_00026430 BBBH-BHAHHB-BHHBBHHHAAABAHHHHAHAABBAAABHHBHBBBHBBHAAABHBBHHBAHBHHHBBBHBAHABHBHABH--HBHHABBBHHAHHHAHHHHHAHHHBHBHHHHHABABHABBHHHHAABHBAABAHAHAHHAH

*000424_00491804 ABH-AH-HAHA--H-H-BAH-BHHB-HAAAAHAAAHH-AA-B---HBHAHAHHHHBBHHB-AABHHH-H-BHBHBHBB-BBBHH-AAHHBHHHHBBABHHBHHH-B-BBAAB-H-B-H-BHBBH-BB-BB-HH-H-H-B--BHH

*000006_00772572 HHHHAABHBHAAHHBHHBHHH-BBHHBHABBBHHHBHAAAHHBHBHAHHAAHAHHAHBHBBAHHBHAHHHAHBAAA-BHAABBAABBAHHBBHHABAHHAHHBHHB-AAHHAHBA-BAAAHHHBBHHAAHHABAHAHHHHBHHA

*000011_00122911 HHHBABBHAHAABAHHBBHBHHHBH-BBBAHABHAHBHHH-HBHBHHHAHAHBBHBAHAAAAHHHHBBBBBHHHAH-ABAHABH-HHAHHHHAAAAABAHABABBHHHBHHHHHHABABBHHAHHAHAHHBABBAHBBHHHHHA

*000164_00591259 HHHHHAHHHAHAAH-HAHHHHAAHHB-AAAHAHHBBBAAHHHHBHHBBHHBABBAABHBHAHBBHHBHAAABHBABBHBHHABHBAHAABHBHHAHAAHHHHHHHHAHBAABBAAHHHAABABHAHHB-BHHAHBABAHHHHA-

*002206_00132457 HHHBABBHAHA-BAHBHHHBBAHHBAHHAAHABAHHBHAH-HHHHHHHBHAABBHBHHHAAHHAHAHBBBAAHHAHAAHABHBH-HBAHHABHAAAHBAHHHABBH-BHAAHAHHAHABBHBHBHAHHHBBABHAHBHHABHHA

*000051_00183131 HHHBAHBBHBAAHAAHHHHAA-HABBHABHAHAABHHHAHBBBHAAHHBABAHHBBAHHHHHBHHHAHHAAHBHAHAHAAA-AABHHAHHHHBABABHHABAHHHHHBAABHAAHBBAHHHHA--BHAHH-AHBHAH-BAHHHA

*001837_00066213 HAHHHBABHAHBHHH-HBBAABHBBHBHHBABHABHAAHAHHHHBHAHAAHHHHH-AHBHBABHBAHB-AHAHAHH-HAHHABB-AHBBBHAAA-AABHHHAHHHHAHBAHABHAB-HBHBHAAHBHBHBHBAAHBHBAHBHHH

*000171_00252850 --ABBHH-BAHHBBABHH-AA-BBH-HHBHBHAHHHH-HBB-BHHHHA-BABHHB-AHA-HAB-HAHAABH-H-HH--HHHH-H-BA-BHAAH-BA-AB-HHAAHA-HAABHHA----HBHH---HA--B-BHBAH--B-ABHB

*002792_00009411 HHAAHHHBBBAHABABBHABAHBHHHHHBHHHAHAHHBHBBHBHHAHAHHABHHBBHHAAHABAAABH-HBBBAAHAHHBA--H-HAHBHAHHHHAHAHBHHAAHA-HAAHAHAB-HHBBBBHAHBHBHHHHHBA-H-BBABBH

*000016_00806801 BHHAAAAHHHBH-HHBHHHBABHAAAAHHHBBHABAH-BHBBHHAABBBHHHAHABHBHBBBHBHHHH-HHBAAHHAAAHAAHHBHHHHHAABBBBBAAHABBBAH-HHHBHBHH--BAHHHHABBHA--AHHBBBHBHBHHHB

*000000_00286731 HAHHHHHBBBHBABHBBBHBHHHHHHBHAAABHBHHBHAAHBHHABHHAAHAHBBAAHHABBBBAABBAAHBHAHAAAHAHABHBHHHAAAHHHBAHBBBAABAHAAHHBHBBHAAHHHHHHAHHHBBHHHHHHBHHBBHHBHA

*000019_00067912 AHABHAHHAHHBBAHHBHBBHHHAABABHHHHHBHHBBHBAAAHAHHAABHHHHABHHHHHAHBAHBAHBHABABHHAAHAHHHABAHHBBAHAHHAABAHHABHHHHHAAHHABHBHABHAHHABABABHHAHAAHABHAAHA

*000120_00439247 AHHHAHBBABHHBHAHHHABHHBHHHAHAABAHHBAAAHHBHHAHHBABHHBHBAABHBHAHBHAABAAHBAAHHHBHHAHBHABHHAAAHHHHHBAHHBHAHAABHAHHAABBHHBAHAHABHABHAHAAHHHHHBABHHABB

*000471_00463347 BBAHBHHHHHBAHHHBHBHHAHHBAHBHBABAABAHHHAAHHHBBBBAAABBBAHAAABHABHBHHHHHHHABHHHABHABHBHAAAHHHABBBHAHHHAHHABAHAHABHHHHHBAHHHBAAAABHBHHABBHAHHHAAHHBB

*000481_00375960 HAHBABHAHABABAHHBHHAAHHBHAHBHAHBHBHAAAABHBHHBHBHHABABHHHBHHBBHBAAHAHBHBAAHBHHHABBHAHHHAHABHHAAHAHHHHHBHHBBAHAHBHHHHABABBABBABHAHHBBHHAHAHAHHAHAH

*000548_00191018 BHHBHBHHBBBHBAHBABHABHABBBBHHBBBBHHABHABBBBHBHBHHHBHAHAHHHHHBHBHHABBHHHABABHAAHHBHBHHAHBHBAHBBBHHHBHBHABAHAHHHHHAHHBHBBBBHHBBHBBHHABHAHBBHAAHBHH

*000565_00155638 BBBAHHBHHHBABHBAHBBBAHHBABHBBAHBBBHAHBBHHHHABHHAHAHHHHBBHHHBBAHHAHHHBHAAHBHHHBABBHBABHABAAHHHHBAHBAAAHHHAHHABHHHAHHHBHHHHHHAHHAAHHAABBAABHBAABBB

*000709_00085607 HAHBABBAHABABAHHBHHAAHHBHAHBHAHBHBHAAAABHBHHBHBHHABABHHHBHHBBHBAAHAHBHBAAHBHHHAHBHAHHHAHABHHHAAAHHHHHBHHBBAHAHHAHHHABABBABBAHHAHHBBHHAHAHAHAAHAH

*000728_00460670 AHAHHBHHAHHBBBAHAHHHBHBBBAHHHAABBBBBHHAHBHBHBHAHBABBBHBAHBBHBBBBABAHBABAAHBBBBHBBAHHAHHAHAAHHAHAAHHHHBHBHAAHHHBAHHHHHABHBBHBHHBHBHHHAHHHHHHHHABH

*000773_00173034 HBHHHBHBAAAHBHHBHAHHBBHHAABHAAHBAHHBBHAHHBHAHAAAHAAHBHAAHHHHBBBAHHAHAHHBHBHAAAHHBABHHBHBAHBHABHHHHHAHHHAAAABBHHBHBABHHBAHHHBBHHBAAAHBHABHBHBHBHA

*000851_00231107 HAAAHHHHBBAHABHBHBABAHHHBAHAHABHAHAHHHHBBHHHHAAHHHAAHBBBHHAAHHBAHABHHHHBHAAHAAAHBABHBAAHBAHHHHHAHAHBAAAAAAHAAAHAHAAAHHHBBBHAHBHBHAAHAHAHHHHAAHHH

*001020_00031574 AHHHAHBBAHHHBHAHHHABHHBHHHAHAABAHHBAAAHHBHAAHHBABHHBABAABHBHAABHAABAAHBAAHHHBHBAHBHABHHAAAHHHHHBAHHHHAHAABHAHHAABBHHBAHAHABHHBHAHAAHHHHHBABHHAHB

*001577_00099168 AHAHHBHHAHHBBBAHAHHHBHBBBAHHHAABBBBBHHAHBHBHBHAHBABBBHBAHBBHBBBBABAHBABAAHBBBBHBBAHHAHHAHAAHHAHAAHHHHBHBHAAHHHBAHHHHHABHBBHBHHBHBHHHAHHHHHHHHABH

*001623_00023972 BBBHAHBHHHBBBHBAHBBBAHHBABHBBAHBBHHAHBBHHHHABBHAHAHHHHBBHHHBBHHHAHHHBHAAHBHHHBABHHBABHABAAHHAHBAHBAAAHHHAHHABHHHAHHHBHHHHHHABHAAHBAAHBAABAHAABBB

*001959_00074464 AHBBHHAABBHBHHHHBBAAHHAAHHHHAAHHHHBHBHHBAAHAABBBAHBHBHHBHHBBHHAAHBBBABAABAAHABBHHABHHHAHAHAHHAAHHHAHHABHAAHHABBHBAAAHBHHHHHHBBAHHBAHHAHHBHABAHHH

*002434_00097229 AHHHAHBBAHHHBHAHHHABHHBHHHAHAABAHHBAAAHHBHAAHHBABHHBHBAABHBHAHBHAABAAHBAAHHHBHBAHBHABHHAAAHHHHHBAHHHHAHAABHAHHAABBHHBAHABABHHBHAHAAHHHHHBABHHABB

*007057_00006923 BHHBHBHHBBBHBAHBABHABHABBHBHHBBBHHHABHAHBBBHBHBHHHBAAHHHHHHHBHHHHABHHHHABABHAAHHBHBHHAHBBBAHBBBABHBHBHABAHAHAHHHAHHBHHBBBHHBBHBBHHABHAHBBAAAHBBH

*000760_00108310 AABHABHHABHAAHBBHBHHBHBHAHAHHHBHHAHABAHAHB-AHHBAHAAHBHHHHAAHHBBBAAAHBBHBAHHBABHBHHHHBAHHAHHBBAHBABBBBHAHHHABBHABAHHHHABBHAHBBAAHBHHBHHHBHAHHHHBH

*002456_00050604 ABHHABHHHHHBBHHHHABAABHHHHHBBBHAABHHAHBAAAHHHBHHBABHHAHHHHHHABHHBBBHHHBABABHBHBHBHAAABBAHAAHBBHBHABBHHAHBH-HAAHBBBAHHHBHBBHAHAAAHHBAAHABHAHBBHHH

*007290_00002629 ABBBHHBHBHABHBHHBHHAAAHAAAABBBHHHBHHAAHHBHABABHBHBBAHHHAAHHBHBBAAHHHAAHAAHBH-AHHBHAHHAHHHABHBAHBHBBAHBHAHBHHHHAHHHHBBHHAAHAHAHAHHHHABHBBAAAAHAAH

*001940_00051414 AHAHABBHA-HAAAAHHHBBHBABA-BBBAHAAHHAHHHHAHH-BBHHAB-HHHHBAAAAAHHHHHAB-HBAHHAHAAHAHHBHHH-HBHHHH-AAABHHAHAHHAAHAHBAAHHH--HBABA-AAAABHHHB-HHBBA--HHA

*000881_00159631 H-HABAHB-HBH-BHBAH-HAHBAH-HB-HHHH-HHH-HH-HBBAA-H-BBA-HHBBHHAHABABHH----H-AHBABHAH-ABAAHAHAAHAHAHAABHHB-BBHAAHBHHBHH--BAHB-HAHBH-BBHHABHAH-A-BHB-

*000620_00284752 BHHBAHBBHBAAHAHHHHHAAABABBHABHAHAABHHHAHHBBAAAHHBABAHHBBAHAHHHHHHHAHHAAABHAHAHAAAHAABHHAAHHHBABABHHABAHHHHABAABHAAH-HHHHHHAABBHAHHHAABHHHHBHHBHA

*000935_00251371 HHBBBABAHHAHHBBHBAHHHHBHHHBAHBBHBHBHAHHHAHBHHHBHBHBBHHABHBBAHHHBBABA-AAHHBABBHHBHHHHHAHBHHAABHHHABBAAHABBHHHAAAHHHABBAAHABHHHBHHBHBBBHAABBHHHHBB

*001164_00231319 BHABABBBHBAABAHAHAHHHABABAHAHHHHBAHABAAHHHHAAAHHBHBAHBBBHBHAHHHHHHHB-HAABHABAHAAHHHHHHBAHHAHBAHAHHAHHAHHBHHBAAHHAAHHBABHHHHHBBAHHBBABBAHHBHBBBHA

*001306_00152811 HHBBBABAHHAHHBBHBAHHHHBHHHBAHBBHBHBHAHHHAHBHHHBHBHBBHHABHBBAHHHBBABA-AAHHBABBHHBHHHHHAHBHHAAHBHHABBAAHABBHHHAAAHHHABHAAHABHHABHHBHBBBHAABBHHHHBB

*001863_00045328 HHHHAHAHABHBAHHBBHHAHHBHAHHABHBHBABHHABABHAHHAHHHAHAAABHBHHBHBBHHABBBHBHAHAHAHHBAHHHBAHBBBHHBAHABBHHHBAHHHHBBAABAAHH-BHAAABHHHBBAHHHAHHBHAAAHHBA

*003390_00014239 BHHAAHAAABAHAHHHBBHBHBHHHHABHHHAHBHHHBHHHHBBHABBAHAHBABHBAAHAABHAHBA-BHABBAHHHHHHABHHBHBBHAHHABHHBBHHHAHBABHHBHBHHHHAAAABBBHAHHHHHBBBHBABBABHAHB

*000112_00353111 AHHHBBBHHAHHBHHBHHHAABHBABABBHBAAHHHHHAHHBHBBAHAABHHAABBAAAHAHBHHHAAHBAHHHHAHBHHHHHB-BHAHHAAHHBBHHHBBHAABAAHHHBHHABAHHBHAHAABAHAA-BHBAAHHBBHBBHH

*000189_00194217 BBHAHHAHAAB-AHBBBHAHHABHBHAHBHBHHBHBABBHBHABHBBAAAAABAHBBHAAHBBHHHHABHHHHBBHAHHHAAAAHAHAHHAAHHAHBBAHHBBHBHAHAAHHHHBAHHAHHHHHAHHHHBBHHABAHHBBHB-A

*000095_00488616 ABHBHHBABHA-ABAHBHHAHHHAAHABBBHHHBHHAAAHBHABABHBBBBAHHHAAHHBHBBAAHHH-AHAAHHHBAHHBHAH-AHBHHBHHAHBHBHAABBAHBAHHHAHHHHBHHAHHBAHAHHHAHHBBHBBAAHAHAAA

*000332_00183525 HAHAHAAHBAHHHAHHABHHHAAHHAAHAAHAHABBHBBAHHHBHHABHHBHBHHHBHHAAHBHHBBAHHHHAHBHBBAAHHBBHAHAAHAHHABAHABHHBAAHHBABAHHBHB-AAAABBBHAAHBHBHHHHBHBAHBHHA-

*001001_00304860 BHAAABHHBHH--HAHAAHAHBH--HBHAAHBHBAHBHBHAB-HHAAHHHHAHBHBABBABAAHHHHH-H-BBHHB-ABHBHBHHBHHHBHBBAHAAHBBHAAABH-ABHHABHH-HBBBAAAH-HBHBH-HHAHBB-B-BHHH

*000208_00125479 HHHAABAHABH-AHHBHHAHBHBHA-HABHBHHABHHAHABH-HHABHHAHAHHBHBHHHABBBHAHB-BBBAHABAHHBHBHH--HBHBHHBAHAABBBBH-HH--BBAHBAAHHABBAH-BBBAHHBHHHA-H-H-A-HHB-

*000670_00002263 -BHHHHHAA-HA-H-HBH-BHAH-BBBAAAHAABAHBAAH-HBHAHBHAHHHBHBAAHHAHBABHHBH-A-HHAHA-BAAAB-H-BHABHHHHBBHHAHHHBHHAA-BBHABAAA-HHHABAAH-HBBAHAHHAHBH-HBHHHB

*001958_00069806 BHBHBAHHBBHBHAHHHHAHAHHAAHHAABABBHHHHHBHHBABAAAHAHHAHAA-HBAHBHBHBHHB-BAAHHABABHBHHAHAHHHAHAHBHABBHBAHHABHHBHBBBAHBHB-BAHBHHAHHBABBHAHAAAHHAHHHBH

*000718_00114031 AHBAHABAHBBAHABAAHABAHHHHHAHHHHHHHHHBHBHHBHHHHHBAAAAAHAABHHHHHHBBHHB-BAHBHBB-HBHHBHHHHHHABAHHBAHHHBHAH-BABBHAHBAHBABBBAAAHBABAHBBBAAHA-BHHABHHHA

*001341_00157323 AHHBHABBAAH-HAAAHHBHBAHAHHAHHABHHHHHHABHAABAAHHHAHHHBABBHHHAHBHBHABH-HABAHAHAH-HHAAHBHAHAHHAABBAHABHHBBHHHBAHBHHHHAABHAHBAHBABAHB-HBBHAAHBHHABHB

*001875_00019572 HBHH-AHHBBHHABHHABABBHHHHHBBAHBHBBHHAAHBHBAHBABHBHBBHBHHHBH-HHHAHAHABABHHHHB-AHAHBHHBAHBBHHABAHHAHBBHHAHABHHHBBABHA-BBABBBHBHHBHHBAHBABBHHHHHAAB

*002689_00030312 BBHABHHHAABH-HBBBAAHHHBHBHAHBHBBHAHBABBBBHABHBBAAAAABAABHHAABBBAHHHABHHHHBBHHHBHAAHAAAHAHHAHHAAHBAAHHBBHBA-HAHHHHHHAHHHHHHHHAHHHH-BHBAHAH-HBHBBA

*004145_00010448 HBHBHBHBA-BHBBBBHBHBHHBAAHAHHHHHHHHHHABAABHHAHHHBHABHBB-HBABHAAHHAABHHBHBABHBAAHBA-BHAHABBHHH-BAHBAHBBHHAHAAHHBAHAHBHHBHHHHHBHABHHABHAHBHAABHAHB

*002216_00138458 HBHHAAHHBBHH-BHBABABBHAHBABBAHHHHBHHAAHHHBAHHABHBBBHHBHHHBHAHAHAHAHABAHHAHHH-AAHHHHHBABHHBBHHABHAHBBHHAHABAHHBAABHAAHBABBHHBHHBHHH-HBABHH-BBBAAB

*000608_00133148 HBHAHHAA-H-HBH-HH-BBHHHHA-HHHB-ABHBHHH-H-B-AHHBHHHHABAHHHBBAAABBABHHBHAAHHBAABAHBHHH-ABHHAHHH-BHBAHHBHBABABHBBBHBBA-HA--HHHH-HHAA-BBHAAHB-BBBAAB

*000859_00133680 HBAHHBHBAAA-HABBHAH-BBHH-HBHAAHHAHHBHBABBB-AHAAAHAAHBH-AHHHHHBBAHHAA-H-HBBHAAH-HBA-BHHHBAHBHABBHHHHAAHHAAH-B-HHBHBA-HHBAHHHBB-HBA-AHHH-BH-H-HBBA

*003696_00018388 --BAH-BAHBB-AABAAAABAHHHH-AHHHHHBHBHBH---HHHHHHB-AHAHHA-BHHHHH-ABAHH-A-BBH-B-ABHABHH--HBABAHA-HHHHBBAH-HA-BHHHBABBHBBHAAA---BAHBB--AH---HB--H-HA

*000027_00766615 BHAHAHBHABHABHA-AHABHAHHHHAAAHHAHHBAAABHAHABHHBHBHABHBA-HHHHAABBAHHAHHBHBHHBBHAAHBHABBHAAAHHHBHBHHHAAABAHH-AHHAAHBHA-BHABBHHAHHABAHHHHHBB-BHHAHB

*000125_00749788 HAHABAHHBHB-HBBBHAAHAHB-H-HBHH-HH-HHH-BHHHBBAAHHHBBAAH-BHHHAHABABHB-HBBBAAHB-BBAHH-B-AH-HHAHAHAHAABHHHBBBH-AABHHBHH-HBA-BA-ABBHA-BHHABH-HHAHHHB-

*016022_00000778 HHBBHBHHBBBBBHBABBHHH-H-H-HHHBBHBBBHHHBBHH-HHBBHBHBBB-BBHHBBHHHHHBBBBBAH-HHAHB-HHHBB-BHHHBHBBHAHBABHBHBBH-BAHBBBBB---HHH-BA--H--BB-HHAH-BBBBHHHB

*000024_00061356 BHHAHHBAHBHAAHAAAHHAHHAAABAAAHHHBBHHHBBBAA-HAHHBHHBHHAABHBHAHAAHHHHAAHAHHHBBAHBBBBHHAHHAHHHAAAA-ABHABHHBHH-HAHHBBBABBHHBBHHH-HAA-BHBABH-HHH-HABB

*000012_00881531 BAHBABHAHABABAHBBHHAABBHAAHBHAHBHBHAAHHBBBAHHHBBHABABHAHBHHBBHBAABAHBHBAHHB-BHABH-AHHHAH-BHHAAHHHHHHHBHAHBBBHHHHHHH--ABBABBA-BAHHBHHHAH-HHHA-HHB

*000274_00298783 ABHBBHBHHHABBBBHBHHHHHHBHBHHBHHHHABHBHABHBHHBABHABHAABHBBHBBHBHHHHHA-BBABBAA-AHBB--BHHH--HBHBHHBHHBAHBHAHABBHHBAHH--BHHBHABBHHBBA-HHHBBHBAHBHABA

*001057_00151252 HHHAHAHHHAHHAHHHAHAAH-A--BHHAAHAHABBBABBHHBHHHBBAHBAB-AABHBHAHBBHHBHHAHBHBAB-HAHHHBHHAHAA-HHH-AHAABHHHAHHHBHBAABBAA-HHAABABHAHHBBBBHAHBAB-HHHHAH

*000424_00421265 A-HBAHHHHHHHBHAAHBAHHBHHBAHAHAAAAAAHHBAAABHAHHBHAHAHHHH-BHHHHAABHHH--ABBBHBH-BBHBHBHHAAHHBHBH-BBHBHABHBHBBABAAABBHAB-HHBHBBH-BHAHH-HHHHAH-BAAB-B

*000927_00052536 HBHBHAHB--BHHBAHHAHHBHAABAHBBBHHHAABHHHBHHHBAAHBBBBHHHBABHHAHAAB-HHH-BBHHHAH-HHBHB-BHABHA--BBAHBHHHABBAHAAAAHABHHHHAHAABHBBBAABHHAHAHAHHH-HB-HBH

*000462_00038225 ABHAHHAHBHHH-AHAAHHHHAAHBAABB-BHHHBHHHBAAA-BHBAHAHHBHHHHHAHAHHHABBBHAHH--HBH-HBAHABB-ABHAHAHBABHHHBBABBHHBBAHABHBHBBAH-AHBHAAAAB-BHHBHHHH-BBBHH-

*000289_00425797 ABHAHHABBHHHHHHAHHBAHHHHHBHBBBHHHBHAHHHAAAHBHBHBBAHHHAHAHAHAHBHBBHHHABHHBABBBHHHBHHAHABHHHAHHHBBBABBHHAHBBBHAAHBBHABAAHAHHHHHAABBBBHBABBHABBBABB

*000472_00032841 ABBABABABHHBHAAHAAHHHHBHHHABHHHBHHBAAHHHBAHBHHHHAABBAAAHHHHBBHAHAHHHAHHBBHBBBAHAAHHHAHBBHBHBAAAHHBHBHHHABBHHHHBABBHABBAHAHHHBHAABHBABHBHHHHBBBHB

*000948_00064647 ABBABABABHHBHAAHAAHHHHBHHHABHHHBHABAAHHHBAHBHHHHAABBAAAHHHHBBHAHAHHHAHHBBHBBBAHHAHHHAHBBHBHBAAAHHBHBHHHABBHHHHBABBHABHAHAHHHBHAABHBABHBHHBHBBBHB

*001011_00202159 ABHHAAHHBBHBABAHABABBHHHBHBBAHBHHBHHAAHHHBAHHABHBHBBHBHHHBHAHHHAHAHABABHAHHHHAHAHHHHBAHHHHHABAHHAHBBHHAHABBHHBBABHAAAHABBBHBHHBHHBAHHABBHAABHAAB

*001428_00025770 ABHBBHHHHHABHBBHBHHHHHHBHBHHBHHAHAHHBHHBHBAHBABAABAAABHBBHBHABHHHBHAHHHABBAABAHHBHHBHHHAHHBHBHHBAHBHHBBAAABBHHBAHHAAHHHAHABHHHBBAAAHABBHBAAHHABA

*000778_00137212 BAHBAHHBBHAA-HHHBHABABBHBHHAHHHAH-HABABHBA-AHHBHABHHAHAHBABAAHHH-HHBAABAHHBH-BHHB--BABAHBBAHAHBHBBH-HBHHBH-HHAHHHBHBHBHHHBBA-AHH-HBHBBBBH-HHABAA

*000007_00947615 BHABAHABHHBAHHHHBHHHAHBHBHAAAHHAAHHABBHHAAHAHBHHABBHABHHBABAAHHBAHABAAHAHABHHBAHBHBBABHHHBHAAHHABHAABBABBHBHHHHAHHHBAAHHHBBABAHHHHBAHHBHBHHBHHHA

*000093_00056447 HHHAHHAAAHBABAHHHHHHHHHBHHBAHAAHHHBBAAHBBHABBBBAAHAHHHAABBHBHHHBAHBAHHHAAHHHHBHAHAHBHHBAAHBBBHAHABBHBHHBBHAHAHHBHABHAAHHHBBHBHAAHBBHBAAAHHAABHHB

*000111_00432205 HHHHHAHHHAHHAHHBAHAHHHABBBBHAAHAAHBBHHHHHHHBBHBHBHBABBAABHBBAHBBHHBBHAABHBABHHHHBHBHHAHAABHAHHAHAAHHHHHHAHAHHAHBBAABHHAABABHAHHBBBAAHABABABHHHAH

*000208_00300039 HHHAABAHABHBAHHBHHHHBHBHAHHABHBHHABAHAHAHHAHHABHHAHAHHHHBHHHHBBBHAHBBBBBAHABAHHBHHHHHAHBHBHBBAHAABBBBHAHHHHBBAABAAHHABBHHABBBAHHBHHHAHABHAAAHHBB

*000249_00358692 BHBBHBHHBBBHBABHHBHABHAHHHBAHBBBHHHABHAHHBHHHHBHAHBAABHAHHHHBHHHHABHHHHABABHAABABHBHHAHHBBABBBHABHBHBAABAAAHAHHHAHHBHHBBBHHBBHBBHHABHAHHBAAAHBHH

*000461_00189249 HHBBBABAHHAAHHBHHAHHHHBHHHBAHBBHBHBAAHHHHHBHHHBHBHBBHHABHBBAHHBBBABABHAHHBABBHHBHHBHHAHBHHAABHHBABBAAHABBHHHAAAHHHABHAAHABHHHBHHBHBBBHAABBHHHHBB

*000507_00161065 BHHBHBBHBBBBBAHBABHABHABBBBHHBBBBHHABHABBBBHBHBHHHBBAHAHHHHHBHBHHABBHHHABABHAAHHBBBHHAHBHBAHBBBHHHBHBHABAHAHHHHHHHHBHHBBBHHBBHBBHHABHAHBBHHHHBHH

*000521_00421390 HHHBBHAHABBBBAAAHHHHBHHBAHAHHAABHABBAAABHAABBHHAAHAHHBAHBHAHBHAHABAHBBBHHHBBABBABHABHHHAHHBHHHHBAHBAHHHBBBHHHAHBHHHHAHHHBBBBBHHABABHHHHHBHHBHHBH

*000650_00141665 HHABABBAHBHBBHAAAHHHAAHHBAHHHHHBABBHHABBHBHBHHHAHAAHHHABHBHHBAHHABHABHHAABAAAAHHBHBBHHBAHHHAHBAAABHHHBHHAHHHHHHAHAHBBHBBAAHABHAHABAAHAAHHAHHBHBH

*000768_00134062 HHABBHBAHAHHHBBHBHBHHBBHABBHBHBABHBHAHHAAHBHHBBHBHHBHHABHBHAAHHABABAHAAHHBAHBHBBBHHHHAHBHHHAHBHHABBHHBABBHHHAAAHHBABBAHHABHHHHHAHHBBBAAHABHAHHBH

*000885_00102261 HHABABBAHBHBBHAAAHHHAAHHHAHHAHHBAHBHHABBHBHBHHHAHAAHHHABHHHHBAHHABHABHBAABAAAAHHHHBBHHHABHHAHBAAABHHHBHHAHHHHAHAHAHBBHBBAAHABHAHABAAHAAHHAAHHHBA

*001328_00031991 HHABHAHBAAAAHAAAHHBBBAHAHBAHHABBAHHHAAHHAHBAABHHAHHHBAHBHHHABBHBHABHAHAHAHAHAHAHHAAHBHAHAHAAABBAHABAHBBHHHBHHBHHHHAAHHAHBAHBHBAHHHHHHBAAHBHHABHH

*001527_00015666 BHBHHAHHHBHBHAHHAHABAHHHAHHAHBABBHHABHBHHBABHAAHAHAAHAAAHBAHBHBBBHHBHBAAHHABHBBBHHHHHHBHAHAHBHAHBHBAHHABHBHHHBBAHBABHHAHBHHAHHBABBHABAABBHABBHHH

*001569_00011390 BHHAAHAAABAHAHHABBHBHBHHHHABHHHAHBHHHBHHHHBBHABBAHAHBABHBAAHAABHAHBABBHABBAHHHHHHABHABHBBHAHHABHHBBHHHAHBAHAHBHBHHHHHAHABBBHAHHHHHBBBHBABBABHAHB

*001752_00193921 HHHBAHBHAHHHBHAHHHHBHHBHHHAHAABAHHBAAAHHBHAAHHBABHHBABAABHBHAABHAABAHHBAAHHHBHBAHBHABHHAAAHAHHHBAHHHHAHAABHAHHAABBHBBAHABABHHBHAHAAHHHHHBABHHAHB

*001792_00208593 BHHAAHAAABAHAHBHBBHBHBHHHHABHHHAHBHHHBHHHBBBHABBAHAABABHBAAHAABHAHBAHBHABBAHHHHHHABHABHBBHAHHABHHBHHHHHHBAHAHBHBHHHHAAHABBBHAHHHHHBBBHBABBABHAHB

*001909_00036931 BHHBAHBBHBAAHAHHHHHAAABABBHABHAHAABHHHAHHBBAAAHHBABAHHBBAHAHHHHHHHAHHAAABHAHAHAAAHAABHHAHHHHBABABHHABAHHHHABAABHAAHBBAHHHHAABBHAHHHAHBHHHHBHHBHA

*002086_00021803 HHABABBBAHAABAHHHHHBBABABAHHAHHABAHABHAHBHHAAAHHBHBAHBHBHHHAAHHHHAHBHHAAHHABAAAHHHHHHHBAHHAHBAHAHHAHHHHBBAHBHAHHAAHHBABBHHHBHBAHBBBABHAHBHHBBBHA

*002227_00025656 HHABABBAHBHBBAAAAHHBAAHHHABHAHHHAHBHHABBHBHBHHBAHAAHHHHBHAHHHAHHABHABHBAAHAHAAHHHABBHHHABHHAHHAAABHHHBHHAAHHHHHAHHHBBABBAAHABAAHHBAAHAAHBHAHHHBA

*003360_00018441 HHBBHHAABBHBHHHHBBAAHHAAHHHHAAHHHHBHBHHBAAHAABBBAHBHBHHBHHBBHHAAHBBBABAABAAHABBHHABHHHAHAHAHHAAHHHAHHABHAAHHABBHBAAAABHHHHHHBBAHHBAHHAHHBHABAHHH

*010054_00004238 BHHAAAAAABAHAHHHBBHBHBHHHHABHHHAHBHHHBHHHHBBHABBAHAHBABHBAAHAABHAHBAHBHABBAHHHHHHABHABHBBHAHHABHHBBHHHAHHAHAHBHBHHHHAAAABBBHAHHHHHBBBHBABBABHAHB

*000223_00207323 AHBBHHAABBHHHH-HBBAHHHAAHBHHAAHHHHBHHHHBAAHAABBBAHBHBHHBHHBBHHAAHBBBABAABAAHABBHHABHAHAHHHAHHAAHHHAHHABHAAHHABBHBAAAHBHHHHHHBBAHBBAHHAHHBHABAHHB

*000232_00216182 HAABBHHHHBHABAAHBHBHHHHBBBAABHAHHHHAHABHBHABBHBAAAHHBBHBAHAAHBBHHAHHAAHHBHHHBHHHHBAHBBAAHABHABHABHBHAHBABHBHHAHHHBAHHBHABBAH-AAHHHBAHHHHHBHHAABB

*000337_00120938 HBBHBHBHBAHHHAHHHHABHAHHHHBAAAHABHAAAABHABBHHBABBHAAABABBHHBAHBHHAHHHABHBHHABHHBBHAHHBBABBHHHHHAHHHHHHHHHAABHBBAAHBABHHAAHBHHHHAA-HBAABABAHHBHAH

*000481_00113000 HAHBABBAHABABAHHBHHAAHHBHAHBHAHBHBHAAAABHBHHBHBHHABABHHHBHHBBHBAAHAHBHBAAHBH-HABBHAHHHAHABHHAAHAHHHHHBHHBBAHAHBHHHHABHBBABBAHHAHHBBHHAHAHAHAAHAH

*001623_00135821 BBBHAHBHHHB-BHHAHBBBAHHBABHBBAHBBHHAHBBHHHHABBHAHAHHHHBBHHHBBHHHAHHHBHAAHBHHHBABBHBABHABAAHHAHBAHBAAAHBHAHHABHHHAHBHHHAHHHHAHHAAHHAAHBAABABAABBB

*001666_00141721 -AAHAHAHAAB-HAHAHHAABAHBHAAHHHHABBBBAHBBHAHAHAABHHHBA-A-HBHHABHBABHH-A-ABHHH-AHHH-BH-AHBBBHBH-HHHABHBH-AHB-HHABHHA-HAHHBHAHBAAAAHBHHBAHBHBH-HHB-

*000232_00019621 ABABBHHHA-H-BAAAHHBHHHHBHBAABBAHAHHAHABHBHABBABAAAHHBBHHAHAAHBBHHAHH-A-HBHHH-HH-HBAH-BAAHA-HA-HABHBHAHBAB--HHHHHHBAHHBHABBHH-AAH--BAHHBHH-HHAABB

*000558_00154331 BHHBBHHHHBBAHABHHBHAHBABHBBAHBBBHHHABHAHHBHBBHBAAABAABH-HHHHBHHHHABHBHHAHABH-HBHBHBHHAHHBBABHBHABHAHBAABAAAHAHHHAHHBHHHBBHHHBHBBHBABHAHBBAAAHBHH

*002575_00035786 HHHAAHAAABAHAHHHBBHBHBHHHAABHHHAHBHHHBHHHHBBHABBAHAABABHBAAHAABHAHBA-BHABBAH-HHHHABHABHBBHAHHABHHBHHHHHHBAHAHBHBHHHBAAHAHBBHAHHHHHBBBHBABBABHAHB

*002862_00090463 HHHHAHAHHHBBBHAHHBBBHHHHHBHHHBHABHBHBABHHHHAHHBHAHBABAHBHBBAAHBBHBHBBHAAHHBABHAHBHHAAABHAAAAHHBHBAHHBHBABH-HHHHHBBABAHHHBHHHHHBAABBBHAHBB-BHBAAB

*000233_00421644 BBHAHBHAHHAAHHHAHHBAABHBBBBHHBAHAAHHAAHAHHHABAABAAHBHHHHAHBHHHBAHAHBAABHBAAH-HHAHABBAAHAH-HAA-HHAHBBAAHHHHAAHAHAHHAABBBBBHAAHHHBHHHBBHHBHBAHHHBH

*000643_00283949 A-HHBH-HHAHH-HABHAHAABHBABABBABAAHHHAHAHHBHBBAHAABHHAABBAAAHAHBAAHAAHBAHHHHAHBHHABHBHBHAHHBAHHHBHBHBBHAABAAHHH-HHAHAHHBHAHAAHAHAAHBHBAHHHBHAHBHH

*001736_00068737 HBHHAAHHBBHAABHHABABBHAHHHBBAHBHHBHHAAAHABAHHABHBHBBHBHHHBHAHAHAHAH-BA-HAHHH-AAHHHHHBAHHHBBHBABHAHBBHHAHABHHHBHABHAAHBABBHHBAHBHHHAHBABHHABBHAAB

*001892_00134223 AHAHAHBHAAH-AAAHHBBBHHABA-BBBAHAHHAAHBHHAHHBBBHHAHAHHHHBAAAAAHHHHHABBBBAHHAHHAAAHHBHHHHHBBHHHAAAABHHAHAHHABHHHBAHHHHBABBABAHHAAA-HHHBBHHBBAAHHHA

*003640_00028100 BBHHHBHHAAAHBHHAHHBAABHBBHBBHBAHAAHHHAHAHHHHBAAHAAHBHBHHAHBHHABAHAHB-AHHBAAHHHHHHABBAAHAHBHAAAHHAAHHHAHHAHAAAAHAHHAHAHBBBHAAHHHBABBBB--BHBAHHHBA

*006539_00003629 HBHHHBAHAAHHAAHAHABAABHBBHBAABABHABHAAHAHHHABHAHAAAHHHHHAHBHBABHHAHHAAHHHAAHAHHHHABBAAHAHBHAAHAHABHAHAHHHAAHBAHABHAAAHBHHHAAAHHBH-HBHHA-H-AHAHBH

*000244_00591564 ABHAHHABBHHHHHAAHHBAHHHHHBHBBBHHHBHAHHHAAA-BHBHBHAHHHAHAHAHAHHHBBHHHABHHBABBBHBABBHAHABHHHAHH-BBBABBHHAHBB-HAAHHBHABAHHAHHHH-AABBBBHBABBHHBBBABB

*000776_00010844 ABAHABBHAHHAHAAHHBBBHHABHHBBHAHAHBAAHBHBHHHBBBHHAHAHHHHBAAA-AHHHHBABBHBAHHAHBAHAH-BHHHBH-HHHHAAAABHHABHHAAHHHHHAAHB-HHBBABABBAAAHHAHBBHHBHAABHHA

*000313_00319450 HHAHHHBA-AH-BBB-B-BHHHBHA-BHBHHABHBHAHHAAHBHHBHAHHABHHABHBHAAAHAAHBA-AAHHBAH-HBBHH-H-AHBHAHAHBHHABBHHBAHBA-AAAAHHAA-HHHHHBH-A-HAH-BBBAAHABH-HHBH

*001711_00165547 -HHHHHHBBAH-HB-BHHBHAABHH-HHHHBHA-HHBB-B-ABHHHHAABAB-BBBAHAHHABBHAHAHH-AHAAHBHHHHHBH-BAHBHBHHHHAHABBHHAABA-BAA-HHABABABBB-AA-HAAHHHHHHAHH-BBABA-

*001924_00093650 HBHABABABHHBAAAHAAHHHHBHHHAHBHHBHHBAAABHBH-BHHHHAABBAAAHHHABBHAHHHHH-HHHHHHBAAHAAHAHAHBBHB-BAAAHHBABHH-ABBAHAHBHBBHABHAHAHHH-BAABHBABHHHHHHHBBBB

*004024_00007990 BBHHBHBHHBBAHAHBHBAAABHBAABHHHBHHBABHHAAAHHBBBBA-ABHBAH-HAHHHHHBHAHH-HHABHHHAHBABHBHAAHHHBAHBBAAHAAHBH-BAHAHABHHAAHHBHHHBAHAAHBBHHABH-AHHBAAHHHB

*007856_00010172 HBHHAAHHBBH-ABHHAB-BBHHHHHBBABBHHBHHA-HHHBAHHABHBABBHBA-HBHAHHHAHAHAHABHAAHHAAHAHHHHBAHHHHHABAAHAHBBHHAHABHHHBBABHAAHBABBBABHHBHHBAHHAB-BHHBHAAB

*000157_00415735 A-B--A-H-AHAAH-HHBHHBH-AA-HABBBHHHBAHHHBHB-HHH-HHHBHBAA-HHHAAABAABHA-HB-AHHB-H-HAAB-----A-ABABH-A-BHBBBAHHABHHAA-HAH-BABBBHHB-AHBHHHHH----HHAHHB

*001328_00160453 HHABHAHBAAAAHA-AHHBBBAHAHBAHHABBAHHHAAHHAH-AABHHAHHHBAHBHHHABBHBHABHAH-HAHAHAHAHHAAHBHAH-HAAABBAHABAHBBHHHBHHBHHHHAAAHAHBAHBBBAHHHHHHBA-H-HHABHB

*010935_00000953 HBAHHHBABBHBHBHHBHBHHHHAH-HBBBABBBHHAAHAAAAHABABHBBHHHAAHBABHBBAAHHH-A-AAHHHBAHHH-AHHAHHHHBBHBHHABBAHBBAAHHABHAHHHHBBH-HBBHH-HHAHHHBBHBBAAHAHAA-

*000866_00181765 ABHBHHBABHH--BHHBHBAHAHAA-ABBBHHBBHHAAHHBHABABHBBBBAHHBAAHHBHBBAAHHH-AHAAAHH-AHHBH-HHAHB-HBBHAHBHBHAABHAHB-HBHAHHHHBBHHHHBAHAHHHHHABBHBBABHAHAAH

*000127_00153598 BHHAHHBHABABAHAAAHAAHHAAABAHHHAHBBHHHBBBAH-HAHHBHHBHHAAAHBHAHAAHHAHAAHABHHBB-HBHB-HHAHHHHHHAAHAHABHABB-BHHAHAAHHBBA-BHHBBHHBBHAAB-HBABH-H-HBHHBB

*001436_00014275 BHHABBAHAAH-BH-ABHHHHHHHH-HBAHAHHHHAB-HBHBA-HHAAHHHHHBAHBAAHHBAHHHA-BBBHBBBHHHBBHABHBBHHAHABHBAHAHBAHHAHHB-HBBHABAABAHBHBHBHAAHAAAABBA-AAAAHHHHH

*000221_00416002 BAHHHAHABBH--ABHBBHBHHBHHHAAHAAAHHABHBHHHHHHHHBHHAHBBAB-HAHBABHABHHA-A-HBAH--BBHA-AHBHBBBBAHBAHBBAAABHBBHAHAHHHHHHAAAAAHHHHBBHABHAHHBAHHH-AHBAHB

*000703_00085867 HBHHAAHHB-HHAB-HABABBHHHBHBBAABHHBBHAAHHHBAHHABHHHBBHB-BHBHAHHHAHAAAAABHBHHHBABAHHBH-AHHH-HAB-HHAHHBHHAHH-BHHBBABHA-ABABBBHBHHBHH-BHHABBHBHBHAAB

*000747_00144690 AHHBAHBBAHBBHHAHBHBHBHBAABBBBHHBAAHBHHAB-BHHAHHHAAAAHHHHBBAHBAAABHBB-HAAHAAHAHBABHHBHAHHHHHAA-HHBHHHHHHHHB-HBABHBHBBBAH-HHBBBBAAH-HHHBH-H-HBHA-A

*003471_00054113 BBHABBAHABHA-HABHHHHHHHHH-HBAHBHHHHAHBHBAB-BHHAAHHAHHAAB-AAAHBHHHBHHHBBHBHBBAHBBHABBBBHHHBHBBHAABHBAHHAHHHBABBHHBAHBA-BABBBHAHH-BAHBHAB-H-A-HHHA

*000019_00967569 AHABHABHAHHBBAHHBHBBHHHAABABHHHHHBHHBBHBAAAHAHHAHBAHHHABHBHBHAHBAHBHHHHABABAHAAHAHHHHBAHHBBAAAHHAAHAHAABBHHHHAHHHABHBAAHHAHHHBAHABBHHHAAHABHAAAA

*000109_00187456 AHHBABBHAHAAHABHBBHBHHHBHABBBAHABHAHBHHHBHBHBHHHAHAHBBHBHHHAAHHBHABBBBBHHHAHHABABABHHHHAHHAHAAAAABAHABABBHAHBHHHHHHAHHBBHAHHHAHAHBBABAAHBBHHHHHA

*000367_00359875 HBHHHAHHBHAHHHBHBBHHHBBBHHBBABBBHHHBHHAAHHBHBHHHHAAHABHAABBBBAHHBHAHHHAHBHAAHBAAAABAABBAHHBBHHABABHAHBBHHHBAAHHAHBAHHAAAHHAHBHHAAHHABAHAHHHBBHHA

*000391_00167464 AHBBHHAABBHBHHHHBBAAHHAAHHHHAAHHHHBHBHHBAAHAABHBAHBHBHHBHHBBHHAAHBBBABAABAAHABBHHABHHHAHAHAHHAAHHHAHBABHAAHBABBHBAAAAHHHHHHHBBAHHBAHHAHHBHABAHHH

*000437_00058406 BAHAAHAAAAHABHHBBHAHHHBBHABAHAAABHHBHHBBHHHBBBBAAHHHHHAABHBHABHHHHBHHAHAAAHHHBBAHBHBHHBAABHHBHHHAHHBBHAHBAABABABHAHAAHHAHHBHHAHAHHBBHHAHHHAABAHB

*000483_00394529 HBAAHHAHBBABABABHBABAHBHHAHHHABHAHAHHBHBBHBHHAHAHHAAHHBBHHAAHABAHABHHHHBHAAHAAAHBABHBHAHBAHHHHHAHAHBHAAAAAHHAAHAHABHAHHHBBHAHBHBHHAHABAHHBHBAHHH

*000562_00355442 HAHBAABAAHHAHBHHHBHBBBBHBBHHHHHBBBBHAAHBAAABHAAHHAHHBBHBBBHHBHHHAABHHABHAAHBBAHBBBHAHAHBBAAAHHHAHHBHBHHBHHHHAAAHAHABHBAHHHBBAHHHBHHHBAHHAHBBHAHH

*000578_00333185 BBBHAHBHHHBHBHBAHBBBAHHBABHBBAHBBBHAHBBHHHHABBHAHAHHHHBBHHHBBHHHAHHHBHAAHBHHHBABBHBABHABAAHHAHBAHBAAAHHHAHHABHHHAHHHBHHHHHHAHHAAHBAAHBAABABAABBB

*000615_00244802 AHABHABHAHHBBAHHBHBBAHBAABABHAHBHBHHBBHAHAAHAHHAABHHHHHHHAHHBAHHAHBAHHHABABAHAAHAHHHHBAHHBBAHHHHAABAHHABHAHHHAAHHAHHHHABHAHAABABABHHAHAAHABHAAHA

*000780_00172361 AHHHABHHHAABHBHHAHBAAHBBHHBHBHHAHBHHAHBHAAAHBAHABAHHHAHBABAAABHHHBBHHHHHBHBHBHBHHHAAHBBHBHAHBHHBHHHBHAHAAHBBHAHBBBAHHHBHBBAAAHAAHAAHAHABABHHBBAH

*000826_00062035 BBBAHHBHHHBABHBAHBBBAHHBABHBBAHBBBHAHBBHHHHABHHAHAHHHHBBHHHBBAHHAHHHBHAAHBHHHBABBHBABHABAAHHHHBAHBAAAHHHAHHABHHHAHHHBHHHHHHAHHAAHHAABBAABABAABBB

*001297_00203688 AHAHHBHHAHHBBBAHAHHHBHBBBAHHHAABBBBBHHAHBHBHBHAHBABBBHBAHBBHBBBBABAHBABAAHBBBBHBBAHHAHHAHAAHHAHAAHHHHBHBHAAHHHBAHHHHHABHBBHBHHBHBHHHAHHHHBHHHABH

*001372_00034420 AHBBHHAABBHBHHHHBHAAHHAAHHHHAAHHHHBHBHHBAAAAABHBAHBHBHHBHHBBHHAAHBBBAHAABAAHABBHHABAAHAHAHAHHAAHAHAHBAHHAAHBABBHBAAAABHHHHHHHBAHHHAHHAHHBHABAHHH

*002721_00012222 AHHHAHBBAHHHBHAHHHABHHBHHHAHAABAHHBAAAHHBHAAHHBABHHBABAABHBHAABHAABAAHBAAHHHBHBAHBHABHHAAAHHHHHBAHHHHAHAABHAHHAABBHHBHHABABHHBHAHAAHHHHHBABHHABB

*002933_00095025 HAAAHHHHBBAHABABHBABHBHHBAHAAABHAAAHBHHBBHHHHAAHHHAAHBBBHHAAHHBHHABBHHHBAAAHHAAHBABHBHAHBAHHHHHAAABBAAAAAAHAAHHABAAAHHHBBHHHHBHBHAAHAHHHHHHHAHHH

*003204_00008493 AHAHHBHHAHHBBBAHAHHHBHBBBAHHHAABBBBBHHAHBHBHBHAHBABBBHBAHBBHBBBBABAHBABAAHBBBBHBBAHHAHHAHAAHHAHAAHHHHBHBHAAHHHBAHHHHHABHBBHBBHBHBHHHAHHHHHHHHABH

*003552_00057277 AHHBBHABABBHHAAAHBAHBHHBAAAHHAABHAHBAAABHHHABHHAAAAHHBAHBHAHBAAHHHHHBBBHBHBBABHABHABHHHAHBHHHBABAHBAHHBBBBHHHAHBHHHBHHHBBBBBBHHABHBHHHHHBHHHHHBH

*003931_00018651 AHABHABHAHHBBAHHBHBBAHBAABABHAHBHBHHBBHAHAAHAHHAABHHHHHHHAHHBHHHAHBAHHHABABAHAAHAHBHHBAHHBBAHHAHAABAHHABHAHHHHAHHAHHHHABHAHAABABABHHAHAAHABHAAHA

*004002_00042429 AHHBBAABHBBHHHAAHBAHBBHHAHHAHAAHHAHBHHAHHHAAHHBHAHAHHBAHBHAHHAAHHHAABBBHBHBBHBHABHABHAHAHBHHHBABHHBAHABBBBAHHAHBBHABAHABBBBBBBHAHABHHHHHBHHHHBHH

*000150_00434739 ABHBHHBHHAABBHBBHABAAAHHAHAHBABBAABHAHAHAHAHHHABHBBAHHABAHBHBHHAAHHHAHHAAABHHHHHBHAHBAHHAHHAHBBBBHBHHHAAHBBHHHAHHAHHBHHAHHAA-BHHBBHAHAABHBAHHAAB

*000734_00194196 AAHHAAHHHHAABHHAHBHBHHHBBHHHHABBAHHBAHHBAHBABHHAHBAHBAAHHABHBHBABAAHAAABAAABAHABHAAABABHHHAHABHHHHHHHABHHHBHHBHABBAHHBAHBAHH-BHBBHHHHHHAHHHABBAB

*000293_00085623 ABBHBBBHHAH-BHAHHAHAHBHBH-ABBABAABHHH--B-HHBBAHAABHHAABBAAHBAHBHAHAH-B-BHHHABBHHA-HBBBHHHHBAH-HBHBH-BHAABHAHHHBHAHHABHBHAHA-HAHAA-BHBAHHHBHHHBHH

*000047_00006814 BHHAHHAAAHBABAHHBHHHHHHHHABAHAAHBHABAHHBHHABHBBAAAAHHHAABHHBHHHHHHBHHAHAAHHH-BHAHAABHHBAHHBBBAAHABHHBBABBAAHAHHBHABAAAHHHBBHHAHAHHBHBHAAHAAABAHB

*000190_00417033 BHBHAHBHHHHHBHBAABBBAHHBABHBBAHBHHHAHBBHHHHABBHAHAHHHHBBHHHBBHHBAHHH-HAAHBHHHBABHHBABHABAAHHAHBHBBAAAHHHAHAABHHHAHHBBHHHHHHAAHAAHBAHABAABABAABBB

*000320_00460658 HHHAHHAAAHBABAHHHHBHHHHBHHBAHAAHBHHBAAHBBHABBBBAAHAHHHAABBHBHHHBHHBH-AHAAHHHHBHAHAHBHHBAABBBBHAHABBHBBHHBHAHAHHBHABHAHHHHBBHBHAAHBBHBAAAHHAABHHB

*000486_00031836 HHHBBHAHHBBHBAAAHHAHBHHBAHAHHHABHAHBAAABHAAABHHAAAAHHBAHBHAHBHAHHHAH-BBHBHBBABBABHABHHHAHHBHHBAHAHBAHHBBBBHHHAHBHHHBAHHBBBBBBHHABHBAHHHHBHHBHHBH

*002828_00019921 HHBBBABAHHAHHBBHHAHHHHBHHHBAHBBHBHBHAHHHAHBHHHBHBHBBHHABHBBAHHHBBABABAAHHBABBHHBHHHHHAHBHHAABHHBABBAAHABBH-HAAAHHHABBHAHABHHBBHHBHBBBHAABBHHHHBB

*003225_00055877 HHBBHHAABBHBAHHHBBABHHHHBHHHAABHHHBHHHHBAAHAHBBHBABHBHHBHABBHHAAHBBBBBAABBAAABBHAHHHAHAHHHHHA-AHBHAHHABHAAHHABBHBHAAAHHHBHHHBHBHABAHHAHHBHHBABHB

*000706_00028819 HAHBAABAHHHABB-AHBHBBBBHBBHHHHHBBBHHAABBAAABHAAHHAAHBBHBHBHHBHHHAABHHABHAAHBHABBBBHAHAHBBHAHHBAAHHBHHHHBBHHHAAHHAHABBBAHHHHBAHHHBHBHBAHHA-HHHAHH

*001047_00348773 AHHHABAHAHHBBHBABHBBBHHHH-HAAHHABBBHBAHHHHBHHHBHAHHABAHHHHBAAHHBABBBHBAABHHAHHAHHBBABHHBAAHAHBBHBAHBBBHAHH-HHHHHBAABABBABHHBBHHBABAHHAAHBBBHHHHH

*004545_00009710 BBHABHHHAABHHHBBBAAHHABHBHAHBHBBHAHBABBHBHABHBBAAAAABAABHHAABBBAHHHABHHHHBBHBHHHAAHAHAAAHBAHHAAHBAAHHBBHBH-AAHHHHHHAHHHHHHHHAAHHHBBHH-HAHBHBHBBA

*000493_00232859 HHHBAHBBAHH-ABAABHBHHHBHA-BBBHABHABHHBAB-BBHHHHHAAAABAHHHBHBBAAABHHBHA-AHAHB-HHHAAHBAAHHHHHAA-HHBHHBHABHHH-HBBB-BHAB-A-AHHHHHBAABBHHHAHAHBH-HAH-

*001129_00222239 ABHBHH-H-HABHBH--AAAHAHAAAABBBHHHBHHAAHH-H-BABHBABBAHHH-HHABHBBAAHHBA-H-AH-H-AHHBH-HHAHHHABHB--BHBBAHBHAHB-BHHAHHHH-BBH-HBA-AHH-AHHABAB-A----AA-

*000962_00113979 H-HH-BHBAAAH-HBBBAB--B-HAABHHHHBA---BHAHHBHH-AHAHAAHBHAAAHAAB-BHH-AH--HHHBH--AB----AAB---H-AA-HAHH-HHHHAAAABHHHBAB--HHB-HHA-BHHBAAAH-HA-HBH--BH-

*000261_00408411 BHHAAHHAHBAAAABHBBBBHHHBHBAHHHHAA-BHABBHHBAHBHBHAHHHBAB-HAABAHHHHHAABHHHBHAHABHHAAHBBHHBBHABBABBHAAHHHHHBA-AHHHBHHHABAAHHHHHHHAHHHBHBABABBAAHABB

*000555_00175737 HHHHAAABHHABHBHABAHBHHHHBHHHHAHAHABHHABHBH-HHHHHAAABAABHBBBAAHHHHABBBABHHBAHAHHAHABHHAABBBAHHAAHBBHHHBHHHHABBAAHAHHBABAAHABHHHBBHBHAH-HBH-ABAHHA

*001432_00004177 HHHBBHAHABBHBAAAHHAHBHHBAHAHHAABHAHBAAABHAAHBHHAAAAHHBAHBHAHBHAHHBAHBBBHBHBB-BBABHABHHHAHHBHHHHHAHBAHHBBBB-HHAHBHHHBHHHHBBBBBHHABHBAHHH-BAHBHHBH

*000067_00425762 HBBHHBHHHAA-BH-HBHHBHBHHAAHBABHHHHHBBAAA-HBHHABHAAAHHHABAAAHHHBHHHAHAHBHBBHAAAAHBHBBBBHBHHBABBHHHBHHHBHAAABBAHHBABABHHBHHAAHBHHBA-AHBAAHBBHHHBHH

*001563_00167245 AHHHABAHAHHHBHAHHBBBBHHHHBHAAHHABHBHBA-HHABHHHBHAHHABAHHHHBAAHHBABBBHBAAHHBA-HAHBBBABHHBAAHAHBBHBAHBBB-AHHBHHHHHBBABABBABHHB-HHAAHHHHAAHBABHHHBH

*000364_00215237 HHHBABBHAHA-BAHAHBHBHAH-H-BHBA-ABHAHBAHHBHBABHHH-HAHHBABHHHAABHBHABB-BBAHHAH-A-ABHBH-HHAHHAHAAAAAHAHAHAHBA-HBAHHAHH--H-BHHHHHAHAB-BABAAHBBHHHHHA

*000004_01423078 AHBAHHHHHHAAHBHAHBBHABBHA-AH-AHBAAAHAAAAAHHAHHHBAAHHBBHHABAHBBBHHHH-AHAHBBABHHAHHABA-HAAHAHAAHAHAABHHAHHHAAHBHHAHBHABH-BHHAAAHAHBHHHBHHBH-BAAAHH

*000208_00390104 HBHAABAHABH-AHHBHHHHBHBHAHHAHHBHHABAHAHAHHAHHABHHAHAHHH-BHAHHBBBHAHB-BBBAHABAHH-HHBHHAHBABHBBAHAAHBBBHAHHH-BBAABAAHHAABHHABBBAHHBH-HAHABHAHAHHBB

*000439_00117145 BBHHHBHAHHA-HHHAHHBAABHBBABBHBAHAAHHHAB-HHAABAABAAHBHHHHAHBHHHBAHAHBAABHBAAHAHHAHABBHAHHHBHAAHHHAHBHAAAHHHAA-AHABHHHHBBBB-AAHHHBA-HBBHABH-AHHHBB

*001000_00213717 BHHHHAHAH-B--HHHBHHHAHHB--HAHBBHAHBAHBHBHBAHHHBHBHHBAHBBHHAHHABAABH--H-A-HBB-HBAAABH--AHAHABBHBBHABHHBAHAABAHHBABHHABHHHBBHAHAAH---BHAHBBAHBA-HA

*000154_00562039 AHHBAHBBABH-HBAABBBHBBBHA-BBBHAB-AHHHHH-HB-HHHHHAAHAHHHHHBAHBAAABHBB---AHAAHAH-HB-BBHA-BHHHAAAHHBHHBBHHHBBHHBHBHBHHB-AHAAAHB--HAHBAHHB--HBHB-AHH

*000993_00023031 HBAAHABBBBA-HB-BHH-BAHBHH-HHHHHH--AHHBBBBBBHHBHAHHABHBBHHHA-HABABABHBABBHAAH-HAB---HHH-H-HAHHBH-HAHBHHAAHAHHAAHHHAB-BHABBBB-BBAHHHHHHBA-HHBBABBH

*007564_00001213 --HHBBBH-A--BH--H-HAA-HBH-ABBHBAAHHHHHAHABAB-AHAABHHA--BAAAHAHBH-HA--B-HHHH--BHHHAHBHBHHHHHAHH-BHHHBBH-ABA-HHHBH-ABA-AB-AHAA-ABAAHBHBA-HHBBB-B-H

*000094_00077391 HHHBABBAH-HB-HAHHAHAHBHBBAHH-AHBHBHBAAAHBB-HBHHHBABHBHBABHHBBBBAABAHBHBAAHBHBHABBBAHHHHHAHHHHAHAHHHAHBHBHH-HHABAHHHA-ABBABBHAHBH-BHHAHHAHAHAHAAH

*001835_00096947 -BHABAHHAAB--HBBBAAHB-BHBAAHBBBBHAHBAHBHBHABHBHAHAAAHAA-HHAABBBAHHHHBH-HHBBHBHAHAAHAHHHAHHAHHBAHBAAHHBBHBHABAHHBBHH-HH-HHHHBAHHHH-BHBAHAHBHBHBHA

*000013_00443988 -BHAHHAHBHH-AAHAAHHAHHHHB-ABBHHHHHBAHHBAAABBHBHBHHHAHHAAHAHAHHHBBHHHABHHBABH-HHAHBBH-BBHHB--HABBBABBHHAHBB-AAA-HBHBBAAHAHHHHAAABHBBHBHHBHABBBAHB

*000000_01023120 H-HHBHHHBBHB-B-BBB-BHHHHH-BAAAABHBHHBH-AHBHHHBHHAAHAHBBAAHHHBBBBAABB-AHBHAHH-AAHAA-AHHHHB-AHH-BAHBB-AABAHAAHBBHBHHA--BHBHHAHBHBBH--HHHB-H-B-HBHH

*003374_00130443 HBHBH-HBH-BAABAAHA-H-HAAB-HBHBHHH-HBH--BBABBAH-BBBBH-HBHHHHAHAABHHH----AHHAH-HABHBAB-ABHABBBBAHBHHHABBHHHHAAHABHHHHAAAABHBHBAABHHAHAHAH-H-HH-HBH

*000013_00404421 ABBAHHAHBHH--ABAAHHAH-HHB-HBBHHHHB-AHHBA-AABHBHBHHHBHH-AHAHAHHHBBHAA--HHBABHBHAAHHBHBHBAHAA-HABBB-BBHH-HBB-AAAHHBHH-H-HAHHHHAAAB--BHB-B-HAB-BAHB

*002460_00007690 B-AHBH-HA-BAHAHBHBAAABH-AABHHHBH-BABHAAA--HBBBBAAABHBAHAH-HHHBHBHHHH-H---H-H-HBABBBHHAAHBBAHBBAAHHAAHHABAH-HAB-HHHHBHHHHHA-AA-BHHHABHAAHHHAAHHB-

*000014_00337661 ABHHHAAHHHAHAHHBBBHHHBABHHBHABHBHHHBHHAABHBHBHHBAAAHHBHAHBBBBAHHHHAHHHAHBHHAHHAAHABAABBAHHBBHHABAHBAABBHHHHHAAHAABAHAAAAHHHHBHHAAHHABHHAAAHBBABA

*000160_00300275 ABHHHBHHHHHBHHHHBABAABHHHBHBBBHHABHAAHHAAAHHHBHHBAHHHABHHAHHHBHHBAHHHBBHBABHBHHABAHAAHBAHAAHBBHBHABBAHAHBBBHAAHBBBAHHABAHHHAAAAAHHBHHAABHABBBHHB

*000199_00348824 ABHAHHABBHHHHHHAHHBAABHHHBHBBBHHHBHAHHHAAAHBHBHBBAHHHAHAHAHAHBHBBHHHABHHBABBBHHABHHAHABHHHAHHHBBBABBHHAHBBBHAAHBBHABHAHAHHHAHAABBBBHBAHBHHBBBABB

*000289_00207891 ABHAHHABBHHHHHHAHHBAHHHHHBHBBBHHHBHAHHHAAABBHBHBBAHHHAHAHAHAHBHBBHHHABHHBABBBHHABHHAHABHHHAHHHBBBABBHHAHBBBHAAHHBHABAAHAHHHHHAABBBBHBABBHABBBABB

*001179_00246724 ABBABAHHHAHBAAHHHBABHBHHBHBHHHBHBAHAAAHAAHBHHAAHBHAAABAHBHHBHHBBHAAHBAHHHHAABHHBBHAHHBBHHBHHABHAAHHHHHHHHAABHBHAAHBAHHAAAHBHAHHAABHBAHBHBBHABHBB

*005584_00036262 ABHHHAHHHHAHAHBBBBHHHBHBHHBBABHBHHHBHHAABHBHBHHBAAAHABHAHBBBBAHHBHAHHHAHBHHAHBAAAABAHBBAHHBBHHABAHHAABBHHHBAAHHAABAHHAAAHHHHBHHAAHHABAHAAHHBBHHA

*004924_00032776 BHHHABAHAHHB-H-B-HHH--BHA-HABHBHBABHB-HA-H-HHAHH-AHA-ABHB-HHHBBHHAB--HBHAH-H-HBBAHHB-AHHH-BHBAHAHBHHBHAHH--B-A-BAAH-ABB-AABB-ABB-BBBAHH-H-A-HHBA

*001446_00070646 HAHBAAAHAAAAHHAAAHHBBAHBBBHHHABBA-HBAHHHAHBABBHAHBHABHAHHHBHBHBHHAAHAH-BAHAH-HABHA-HBHBHHAAAABHHHAHAHABHHHBHHBHHHHAA-BAHHAHB-BHHB-HHAH--H-HABBAB

*003480_00108863 AHHHABAHAHHB-HHABBBBB-HHH-HAAHHABABHBAHBHHBHHHBHAHHABAHHHHBAAHHBHBBB-BAABHAA-HAHHBBABHHBAHHAH-BHB-HBBBHAAH-HHHHBBHAB-HBABHHBBHHBAHAHHAAAB-BHHHHA

*006136_00007858 AHHHHHABHAHABHHAHBBAABHBBBBAHBABHABHHAAABH-HBHHHAAHHBHHBHHHHBAHH-AHH-AAHHAHBAHHHHA-B-AHHHBHAAHAHBHHHBH-HAB-ABBHHBHAB-AAHBHHAABABBBABAHB-H-AHHAHB

*000277_00595891 AHAAHAH-BBA-HBABHHABAHBHHHHHHHHHAHAHHBBBBHBHHAHAHHABHH--HHAAHABAHABBAHB-HAAH-HHBAAHHAHAHBHAAH-HAHAHBHBAAHAHHAAHABABHH-HBBBHAAB-BHHHBHBAHH-HBABBB

*000050_00778103 BHABABBBHBAAHAHHHAHAHABABHHABHAHBABAHHAHHHBAAAHHBABAHHBBABHHHHBHHHHBHHAABHABAHAAAHAAHHBAHHHHBAHAHHHAHAHHBHHBAAAHAAHBBABHHHAHBBHHBHBAHBHHHHHHBBBA

*000093_00260643 HHHAHHAAAHBABAHHHHHHHHHBHHBAHAAHHHBBAAHBBHABBBBAAHAHHHAABBHBHHHBABBAAHHAHHHHHBHAHAHBBHBAAHBBBHAHABBHBHHBBHAHHHHBBABHAAHHHBBHBHAAHBBHBAAAHHAABHHH

*000179_00170605 HHABABBAHBHBBHAAAHHHAAHHHABHAHHHAHBHHABBHBHBHHBAHAAHHHABHAHHHAHHABHABHBAABAHAAHHHHBBBHHABHHAHHAAABHHHBHHAHHHHHHAHAHBBABBAAHABAAHHBAAHAAHBAAHHHBA

*000246_00305751 BHABABBBHBAABAHAAAHBHABABAHHAHHHBAHABAAHHHHAAAHHBHBAHBBBHBHAHHHHHHHBHHAABHABAHAHHHHHHHBAHHAHBAHAHHAHHAHBBAHBAAHHAAHHBABHHHHHHBAHBBBABBAHHHHBBBHA

*000413_00539874 HHHBBHAHABBHBAAAHHAHBHHBABAHHAABHAHBAAABHAAHBHHAAAAHHBAHBHAHBHAHHBAHBBBHBHBBABBABHABHHHAHHBHHBHHAHBAHHBBBBHHHAHBHHHBAHHHBBBBBHHABHBHHHHHBHHBHHBH

*000712_00163155 HHHBBHAHABBHBAAAHHAHBHHBAHAAHAABHAHBAAABHAAHBHHAAAAHHBAHBHAHBHAHHBAHBBBHBHBBABBABHABHHHAHHBHHHHHAHBAHHBBBBHHHAHBHHHBAHHHBBBBBHHABHBAHHHHBHHBHHBH

*001432_00004541 HHHBBHAHABBHBAAAHHAHBHHBAHAHHAABHAHBAAABHAAHBHHAAAAHHBAHBHAHBHAHHBAHBBBHBHBBABBABHABHHHAHHBHHHHHAHBAHHBBBBAHHAHBHHHBAHHHBBBBBHHABHBAHHHHBHHBHHBH

*001597_00171555 HHHBAABBHBAAAHHHHHHAAAHHHBHABHAHAABHHBAHBBBAHAHABHBAHHBBAHABHHBBHHAHHAHHBHAHAHAAABAABHHHHHBHBABABHHABAHHHHABAABHAAHHHAHHHHAABBHAHHHAHBHBHBBAHHHA

*001712_00023168 BHHAAHAAABAHAHBHBBHBHBHHHHABHHHAHBHHHBHHHBBBHABBAHAABABHBAAHAABHAHBAHBHABBAHHHHHHABHABHBBHAHHABHHBHHHHHHBABAHBHBHHHHAAHABBBHAHHHHHBBBHBABBABHAHB

*001982_00008714 HHABABBAAHHHHAAHHBBBAHABHABHHHHAHHAABHBBHHBBBBHHHHAHHHHBHAAAAHHHHBABBHBAHHAHAAAHHABHHHHABHHHHHAAABHBABHHAAHHBHHAAHAHBABBAHAABAAAHHAHBHHABHAAHHHA

*003166_00075269 BHHAAHHAABAHAHBHBBBBHBHHHBABHHHAHBHHHBBHHBBBHABBAHAABABHBAAHAABHAHBAHBHHBBAHHHHHHABHABHBBHAHHABHHBHHHHHHBAHAHBHBAHHHHHHHBBBHAHAHHHBBBHBABBABHABB

*009166_00002974 HHHBBHAHABBHBAAAHHAHBHHBAHAHHHABHAHBAAABHAAABHHAAAAHHBAHBHAHBHAHHHAHBBBHBHBBABBABHABHHHAHHBHHHAHAHBAHHBBBBHHHAHBHHHBAHHBBBBBBHHABHBAHHHHBHHBHHBH

*000143_00694976 HAHBABBAHABABAHHBHHAAHHBHAHBHAHBHBHAAAABHBHHBHBHHABABHAHBHHBBHBAABAHBH-AAHBHHHABBHAHHHAHABHHAAHAHHHHHBHHBBAHAHHHHHHABHBBABBAHHAHHBBHHAHAHAHAAHAA

*000165_00117008 HAHBAAHHHAAAHHAAHHHBBHHBBHHHHABBAHHBAHHHAHBABBHAHBHHBAAHHABHBHBHHAAHAHABAHABBHABHAAHBHBHHAAHABHHHAHHHABHHH-ABBHAHHAAHBAHBAHBBBHHBAHHAHBHHAHABBAB

*000677_00068565 AHHHABHHHAABBHHHAABAAHHBHHBBBHHAHB-HAHBAAAHHBHHABABHHAHBHBHHABHHHBBHHHBHBHBBBHBHBHAAABBABHAHBHHBHHBBHHHHAHBHAAHBBBAHHHBHBBHAAHAAHAAHAHABAAHHBHAH

*001189_00096458 AHBAHABHHBHHBABAAHABAHHHHBAHHHAHHBHHBHBHHBHHHAHBAHAAHHAABBAHHHBBBHHBABAHBBHBBHBBHHHHHHHBAHAHBHAHHHBAAHABHBHHHBBAHBABBBAAAHBAHAHHBBHAHAABH-ABHHHA

*001562_00023542 BBBHHAHHHBHBHAHHAHABAHHHABHAHBABBHHHBHBHHBABHAAHAHHAHAAAHBAHBHBBBHHBHBAAHHABHBBBHH-HHHBHAHAHBHAHBHBAHHABHBHHHBBAHBABHBAHBHHAHHBABBHABAABBHABBHHH

*002463_00070212 HAHBAABAAHHAHBHHHBHBBBBHBBHBHHHBBBBHAAHBAAAHHAAHHAHHBBHBBBAHBHHHAABHHABHAAHBBAHBBBHAHAHHBAAAHHHAHHBHBHHBHHHHAAAHAHHBBBAHHHBBAHHHBHHHBAH-AHBBHAHH

*002464_00031631 BAABAHHHHAHBAHAHHBABBAAAAHHHBHAAHBAHHHHBAAAHAAHBHHHHABABHHHHABHHHHHBBABHBAHAAABAHBBBAHHHHHBBHAAHHHBBBABABHHHBAHHAAHA-HHHAHHBAAAHABBHHABHHHHBHHBB

*003360_00017060 AHBBHHAABBHBHHHHBBAAHHAAHHAHAAHHHHBHBHHBAAHAABBBAHBHBHHBHHBBHHAAHBBBABAABAAHABBHH-BHHHAHAHAHHAAHHHAHHABHAAHHABBHBAAAABHHHHHHBBAHHBAHHAHHBHABAHHH

*000289_00220084 ABHA-HABBHHHHHBAHHBAHHHHHBHBBBHHHBHAHHHAAAHBHBHBBAHHHAHAHAHAHBHBBHHHABHHBABBBHHABH-AHABHHHAHHHBBBABBHHAHBBBHAAHHBHABAAHAHHHHHAABBBBHBABBHHBBBABB

*000005_00566093 HHBBHHHAHBHBHHAABBHBBHHBBBHHAAHHABBHHAHBAAHABBBHBHHAHHBHHAHBHHBAABBBBHHHHBHA-HHHAHAHAHAHHHHHAAAHBHAHBBBHAAAHAHBHBHAAABHHBAHBHHBHABABHHBHHHABABA-

*000175_00211530 HHBHHBAHBBHAHBABHBAABBBHHAAHAHHBBHABBABHBHABHBHAAHBBHHHHHAHHBHHAHABB-HAHBAAHHBBAHABHHBBAHHHABHHHAHHHHHAHHH-BAHBHBHHAABBHHBHBAHHHHAAABBHBBBHHBAHB

*000533_00046994 BHHAAHAAABAAAH-HBBHBHBHHHHABHHHAHBHHHBHHHHBBHABBAHAHBABHBAAHAABHAHBAHBBABBAHHHHHHABHABHBBHAHHABHHBHHHHAHBA-AHBHBHHHHAHHABBBHAHHHHHBBBABABBABHAHB

*000552_00006969 BHHAAHAAABAHAHH-BBHBHBHBAHABHHHAHBHHHBHHHHBBHABBAHAHBABHBAAHAABHAHBA-BHABBAHHHHHHABHABHBBHAHHABHHBBHHHAHBAHAHBHBHHHHAAHABBBHAHHHHHBBBHBABBHBHAHB

*002445_00039864 HHHBBHAHABBH-AAAHHAHBHHBAHAHHHABHAHBAAABHAAABHHAAAAHHBAHBHAHBHAHHHAHBBBHBHBBABBAB-ABHHHAHHBHHHAHAHBAHHBBBBBHHAHBHHHBHHHBBBBBBHHABHBAHHHHBHHBHHBH

*000019_00537184 AHABHABHAHH-BAHHBHBBHHHAABABHHHHHBHHBBBBAAAHAHHAABHHHHABHHHHHAHBAHBHHHHABABAAAAHAHHHBBAHHBBAAAHHAABAHHABBH-HHAAHHABHBHAHHAHHHBAHABBHAHAAH-BHAAHA

*001451_00047919 BBHABAHHAABHHHBBBAAHBHBHBAAABHBBHAHBAHBHBHABHBHAHAAAHAABHHAABBBAHHHBBHHHHBBHAHHHAABAHHHAHHAHHBAABAAHHBBHBH-HAHHBBHHAHHAHHAHBAHHHH-BHBAHAH-HBHBHA

*001788_00025193 HAAHAHHHBAHBAAAHHH-ABAHHAHAHHHAAHBHHHBHBBAHAHAABHHHHABABHHHBABHHHBHBBAHHBHAAAAHAHABH-AAHHBBBHAAHHABHBAHAHHBHBA-BHAHAHAHBHAHBAAAHABHHBAHBHAHHAHHB

*003426_00059005 BBHA-AHHAABHHHBBBAAHBHBHBAAHBHBBHAHBAHBA-HABHBAAHAAABAABHHAABBBAHHHH-HHHHBBHAHHHAAHAAHHAHHAHHBAHBAAHHBBABAAHAHHBBHAAHHAHHHHHHHHHHHBHBAHAHAABHBHA

*001179_00183772 ABBABAHHHAHBAAHHHBABABHHBHBHHHBHBAAAAAHA-HBHHAAHHHAAABAHBHHBHHBBHAAHHABHHHAABHHBBBAHABBBHBHHA-BAAHHHHHHHHA-BHBHHAHBAHHAAAHBAAHHAA-HBAHHHBHHABHBH

*002634_00058488 ABHHABAHAHHABHHABHBBBHBHHBHAAHHABHBHBAHBHHBAHHBHAHHABAHHHHB-ABHBHBBBBBAABHH-BH-HHBBHHHHBBAHAABBHBAHHBBBAAHHHHHHHBABBHBBABHHBBHHBAHHHBAA-BHBHHHHB

*000076_00303088 AHHHBHBHB-HHBHHBHHBAABH-HHAHBHBAAAHHAH---BBBHAHA-BHHAABBAAAHAHB-HHAAHBA-HAHA-B--BH-HH-HABHHAH-HBHHHBHH-ABA-HHBBHH-BA-AH-AHAAHAAA--HHBAH-BBBAHBH-

*001209_00120385 BBHHHBHH-AA--H-AHABAABHBB-BHHBAHAAHHHAHAHHB-BAABAAHBHAHHAHBHHABAHAHB-AA-BAA--HHHHABB-AHAHBHAAHHHAHBHAABHHH-AHAAAHHHH-BBBBHAAAHHBHHHBBHHBH-AHAHBH

*001785_00117624 HAAHHH-HBBHHABHBHB-BHBHHHBHHAHABABHHBHAA-BHHHHAHHAHAHB--AHHHBB-BAABB-HBBBAHHHA-ABABHBHHHHAAHHBBAHHBBAA-AAA-HABHABHAA-HHBHHAH--BBHHAAHHBAB-BHHHHA

*001489_00017502 HHHHHHAHBHBBBHAHHBBBHHH-HBHHHBHABHBHBABHAHHAHHBHAHBABABHHBBAAHBBABHHBHAAHHBAABAHBHHH-ABHAAHAHHBHBAHHBHBABABHAHHHBBABAHHHHHHHHHBAABBBH-HBB-BHBAAB

*005084_00004064 HHHHABHHHHA-BHBABAABAHHHH-HBHAHABHBHHABHBH-HHAHHAHHBAAHHBABAAHBHHHBBHABAABAHAHHAHABBAHAHBBAHHAHHBBHAABAHHHABBAAHHHHBABHAHHBABBABHBHHHBHBH-AHAHHA

*001438_00145989 AHHHAHAA-HHAHHAHBHBBBAHHHBHAAAHAHHHHBAAB-HBAHHBHAHBABABHAHBAAHHBBBBBHHAABHHA-HAHHBBHHHHBHAHAABBHBAHHHBBAAA-HHHAHAAHBAHBAHHABBHHB-HAHHAAABHBHBHHH

*000575_00148226 AHHHABAHAHH-BH-ABHBBBBHHHBHAAHHABHBHBAABBHBAHHBHAHHABAHHHHBAAHHB-BBB-BAABHHHHHAHHBBHHBHBHHHAABBHBAHHBBBAABBHHHHHBAH-ABBABHHBBHHBABAHHAAHBHBHHHHH

*000147_00405173 AHHABHAA-BA-AB-HABBHAHBHABAHBAHHA-HBAHHBAHBAHHBBBAHHBBHHABAHHBBHHBHHHH-AHBHBHH-BBHBHBHHAHHHAABAHAABBH--BHA-HBHHABBBH-HBBHHAAHHHH--HHBH-BHBB--AH-

*000886_00240944 -BHBHHBHBHABHB-HBHHAH-HAA-ABBBHHABAHAAHH-AABABHBHBBAHHH-AHHBHBBAAHH--AHAAHBHBAHHBH---AHHHABHBAHBHBBAHBHAHB-HHHAHHH--BHAHHAAHAHAHH--ABBBBAHA-HAAH

*010107_00036791 BBHAB-AHAHHHAHABHHHHHAHHHAA-AH-HHBHAHBHHBB--HHAAHAABH---BAAAHBHHHBHH-BBHBHBBAHBBBABBBHHHHBHBBHA-HABAHHAH-HBABBHHHABBA-BHBBB-AHHAB-HBHA-BA-AHHHHA

*000134_00117976 BHBBHAAABBBAAA-HHBHBHAB-H-AHHAAAHHABHBHHAHHHHHBHHAHBBAHBHAHBABHABHHABA-HBAHBABBHA-AHBHHBBHHABAHHBAAABHBBHABAHHHHHHAHHAAHHHHABHABHHHHBHAAHHABHAH-

*000065_00197374 AHAHHBHHABHBBBAHAHHABHBBB-HHHAABBBBBH-AHBHBHBHAHBABBHHBAHBBHBBBBABAH-ABAAHBBBBHBBHHB-HHHHAAHHAHAAHHBHHHBHAAHHHBAHHHHBA-HBBHB-HBHBAHHAHHHH-HHHABH

*001287_00098345 HBAHHBBHAAAHAAHBHHHHBBHHHHBHAHHHABBBHBABHBBAHAAHAHAHBAA-HBHHHBHH-BAH-HHHBHHAAHBHBABH-AHHAABBABBHHHBAHHBAAHAB-HBBHHAHBHBAHHHBAHHBAAAHAHABH-H-HBBA

*000008_00012562 BBHHBHHHBHBHBBHBBBBBBBHBBBHBBHBHHBBHBHHBHBBHBBHHHHBBHBBHHBBHHBBBBBBHBBBBBBHHHBBHHBBBBBHBHHBBBBHBHBBBHBHBBHHBHBBHBHHBHBHHHHHHHBBBHHBHBBHHHBHHHBBB

*000988_00281375 ---AABHB--AHAB--HHABAHBBHHHHHAHHAAAHH--BBH-HHAHAHHAHH-B--HAAHABAHABH--A-HAAHA-AHB--H-HAHBAHBH-AAHAHBAHAAHA--AA-ABAHHHHABBBH-HBHB--HHHBAHA-HBABHH

*002059_00124418 H-AHHBBH-AAHHAHBHHHBBBHHBHBHAHHHABBBH--BABBAHAAHHHHHHAAABBHHHBHHHBAHHHHHBHHHAHBABABH-AHHAHBBABBBHHBAHHHAA--BBHHBHBAHBHBABHB-HBHBHA-HAAABHBHAABBA

*002268_00125638 BAHHBHAHBHH-BBHBHBHHHAABBH-HBABAABHAHH-HABHABBAAAABBHHHAHBBAAHHBBBHH-B-HBHHHABHAHHBBBHABHHHBHBHBAAHAHHABBH-HABAHHHHB-AHHBHBHHBHH-A-HBHAAHAAHABBH

*000049_00718460 BBHABAHHHAHHBHBBBAABBBBHBAAHBBBBBHHBABBHHHAHHHHAHAAAHAABHHAABBBAHHHABBHHHBBHHHAAAAAABBAAHHAHHBHHHAAHHBBHAHAHAHHHBHHAHHABHHHBABHAHHAHBAHAHHHHABHH

*000065_00885223 AHAHHBHHAHHBBBAHAHHABHBBBAHHHAABBBBBHHAHBBBHBHAHBABBBHBAHHBBBBBBABAHBABAAHBBBBHBBHHHAHHHHAAHHAHAAHHAHBHBHAAHHHBAHHHHHABHBBHBHHBHBAHHAHHHHHHHHABH

*000077_00173906 BAHBAHHBHHAAAHHHBHABABBHBHHAHHHAHBHABABHHABAHBBHABHHABHHBABAAHHHAHHBAAHAHHBHBBAHBABBABHHBBAHAHAABHHABBHBBHBHHHHHHBHBHAHHHBBABAHHHHBABBHHHHHBHBAA

*000099_00581167 HBBABHBHHAHHAAHHHBABHAHHBHBAHHBABHAAAAHAAHBHHAABBHAAABAHBHHBAABHHAAHHAHHHHAABHHBBHAHABBHBBHHAHHAAHHHHHHHHAABHBBAAHBHBHHAAHBHHHHAABHBAABHBHHABHBH

*000099_00596928 HBBABHBHHAHHAAHHHBABHAHHBHBAHHBABHAAAAHAAHBHHAABBHAAABAHBHHBAABHHAAHHAHHHHAABHHBBHAHABBHBBHHAHHAAHHHHHHHHAABHBBAAHBABHHAHHBHHHHAABHBAABHBHHABHBH

*000143_00049080 BAHBABBAHABABAHBBHHAAHHBAAHBHAHBHBHAAHABHBAHBHBHHABABHAHBHHBBHBAABAHBHBAAHBHHHABHHAHHHAHHBHHAAHHHHHHHBHHBBABAHHHHHHABABBABBAAHAHHBBHHAHAHHHAAHAH

*000210_00380425 BAAHHHAHHABHAAHAAAAABAHHHAABHHHABBBBAHHBHAHAHAABHHHBHHABHBHHABHBABHHBHBHBHHHAAAHHBHBAAHBBBHBBAHHHABHBHHAHBAHHABHHAHHAHHBAAHBAAHHHBHHBAHBBBHAHHBB

*000218_00271711 BBBHHBHAAHBABHHBBBHAAHHBAAAHAAHHABBAAHHHHBABBBABBAAAABHHBHHHHHBHABAHBHHAHABAHHABHAAHBBHHHBBHAAHHAABHHHHAHHHBHBHHAHHAHABBABBAHHAHABHBHAHAAHHHHHHH

*000231_00065298 BAHHAHAAHAHABHHBBHAHHHBBHABAHAAABHHHHHBHHHHHBBBAAHHHBHAABHBHABHHHHBHHABAAAHBHBBAHBHBHHBAABHHBHHHAHHBBHAHBAABABABHAHAAAHAAHBHHAHABHHBHBAHHHAABAHB

*000240_00135198 HAAHHHHHBBAHABHBHBABHBHHHHHAAHABAAHHBHHBHHHHHAAHHAAAHBBHAHHHBBBBHABBAHHBAAAHHAHABABBBHAHHAAHHHHAAABBAAHAAAAHABHABAAAHHHBHHAHHBHBHHAHAHHABBHAAHHH

*000248_00011679 AHABHABHAHHBBAHHBHBBHHHAABABHHHHHBHBBBHBHAAAAHHAHBAHHHABHBHBHAHBAHBHHHHABABABAAHAHHHHBAHHBBAAAHHAAHAHAABBHBHHAHHHABHHAAHHHHHHBAHABBHHHAAHABHAAAA

*000391_00407598 AHBBHHAABBHBHHHHBBAAHHAAHHHHAAHHHHBHBHHBAAHAABHBAHBHBHHBHHBBHHAAHBBBABAABAAHABBHHABHAHAHAHAHHAAHHHAHBABHAAHBABBHBAAAABHHHHHHHBAHHBAHHAHHBHABAHHH

*000429_00007872 AHBBHHAABBBBHHHBBHAABHHAHAAHAAHHHHHHBAHBAAAAABHBAHBHHHHHHHBBHHHAHHBBAHAABAAHABBHHABHAHAAAHAHHHAHAHAHBAHHAHHBABHHBAAAABHHHBHBABAHHHAHHAHHBHABAAHH

*000560_00014968 AHHHABHHHAABHBHHAHBAAHBBHHBHBHHAHBHHAHBHAAAHBAHABAHHHAHBABAAABHHABBHHHHHBHBHBHBHHHAAHBBHBHAHBHHBHHHBHAHAAHBBHAHBBBAHHHBHBBAAAHAAHAAHAHAAABHHBBAH

*000599_00032819 HBBHBHBHBAHHAAHHHHABHAHHHHBAAHBABHAAAAHHABBHHAABBHAAABAHBHHBAABHHAAHHAHHBHHABHHBBHAHHBBABBHHAHHAHHHHHHHHHAABHBBAAHBABHHAAHBHHHHAABHBAABABAHABHHH

*000690_00278818 BHHBHHHHBBBHBAHBABHABHABBHBHHBBBBHHABHABBBBHBHBHHHBAAHHHHHHHBHHHHABHHHHABABHAAHHBHBHHAHBBBAHBBBABHBHBHABAHAHAHHHAHHBHHBBBHHBBHBBHHABHAABBAAAHBBH

*001329_00292327 HBBAAHHAHBABAHHHHBHBHHHHHHABHBHAHBHHHBHHBHBBHAHBAHAAHBHHBAAHAABHAHHAHBHHBBAHHAAHHABHABHBBAAHBABAHBBBBHAHBAHAHBHBBHHHHABAHHBHAHHBHHBBHHBABBABHAHB

*001394_00221787 HAAAHHHHBBAHABABHBABHBHHBHHAAABHAAAHBHHBBHHHHAAHHHAAHBBBHHAAHHBHHABBHHHBAAAHHAAABABHHHAHBAHHHHHAAABBAAAAAAHAAHHABAAAHHHBBHHHHBHBHAAHAHHHHBHAAHHH

*001793_00015413 AHABHABHAHHBBAHHBHBBAHBAABABHAHBHBHHBBHAHAAHAHHAABHHHHHHHAHHBHHHAHBAHHHABABAHAAHAHBHHBAHHBBAHHAHAABAHHABHAHHHHAHHAHHBHABHAHAABABABHHAHAAHABHAAHA

*001793_00186859 AHABHABHAHHBBAHHBHBBAHBAABABHAHBHBHHBBHAHAAHAHHAABHHHHHHHAHHBHHHAHBAHHHABABAHAAHAHBHHBAHHBBAHHAHAABAHHABHAHHHHAHHAHHBHABHAHAABABABHHAHAAHABHAAHA

*003344_00030807 HAHBAABAHHHABBHAHBHBBBBHBBHHHHHBBBHHAAHBAAABHAAHHAAHBBHBHBHHBHHHAABHHABHAAHBBABBBBHAHAHBBHAHHHAAHHBHHHHBBHHHAAAHAHABBBAHHHHBAHHHBHBHBAHBAHHBHAHH

*000014_00864396 ABHHHAHHBHAH-HBHBBHHHBHBHHBBABHBHHHBHHAAHHBHBHHHAAAHABHAHBBBBAHHBHAHBHAHBHHABBAAAABAABBAHHBBHHABAHHAABBHHHBAAHHAABAHHAAAHHHHBHHAAHHABAHAAHHBBHHA

*001862_00036367 ABHHHAAHHHAAAHHBBBHHHBABHHBHABHHHHHBHHAABH-HBHHBAAAHHBHAHBBBHAHHHHHHAHAHBHHABHAAHABAABHAHHBBHHABAHBAABBHHAAHAAHAABAHAHAAHHHHBHHAAHBAHHBAABBBHABA

*002761_00033251 AAHBAHBHAHHHHHAHAHHBHHBHBHABAABAHBBHAAHHBHAAHABAHHHBABHHBHBHAHBHAABAHHBAAHBHBABAHBHABHHA-AHAHBHHAHHHHAHAAHHAHHAHHBHBBAHABABHHBHAHAAABHAHBABHHAHB

*000450_00061953 HHBH-BBHBHHBBH-HHABBHHAHA-HHABHHH--BHAAHBHBAHHBHAHAHHHA-AAAHAHHHBHAHA-BBBHH--HH-B-BAHBA--BHAH-HAHBHAHHBAAABHBHHBBBABBHBHHABHBBBB-AHHB-B-BBH--BHH

*000977_00210752 HBA-HAHAH-AB-HAAHB-BBBHBBB-H-AHHAHBAAH-BAA-ABBHH-HHAHHB-HAHHHHBAABBA-H-HHBHAAHHHAHAHAHAHHAHAAAABBHABHBBHAAHHAHBHBHA-HHA-HAHBHH-HA---HHB-H-ABABHH

*002107_00142097 HBHB-AHBHBBH-BABHAHBBH-AB-HBHBHHBBHBBHBBHABB-HHBBBBHHHBHHHH-B-ABHHHH-BBHHHAH-BHBH-BBBA-B-BBBB-HBHHHABBHHHH-AHABHHH--H--BBBHB-ABHH-HBBAHAHAHHBBBH

*002733_00055203 ABHB-HBH-HABHHHBBHHABABAAHAHBBBBA--HAAHHBAHB-HHBHBBAHHHAAHH-B-BAABHH--HAAHB--HH-B--HBAB--H-AHAHBHBBAHBHAHBHHHHAHHH--HHBHHHA-AHAHHBHABHBHABAA-AAB

*001779_00042100 ABHBHHBHBHA--B-HBHHAHAHAA-ABBBHHHBHHAAHH---BABHBHBBAHHAAAHHBHBBAAHHHA-BAAHBHBAHHBHAHHAHHHABBBAHBHBBAHB-AHBAHHHAHHHHBBAHHHHAH-HAHB-HAB-BBAHAAHAAH

*000047_00860106 BHHAHHAAAHBABAHHHHHHHHHHHABAHAAHBAHBAAHBBHABHBBAAHAHHHAABBH-HHHBHHBHHAHAAHHAABHAHAHBHHBAAHBBBHAHABBHBBHBBHAHHHHBHABAAAHAHBBHBHAAHBBHBHAAHHAABHHB

*000587_00075706 BHABABBBHHAABAHHAAHBBABABAHHAHHHBAHABHAHHHHAAAHHBHBAHBHBHBHAHHHHHHHBHHAAHHABAHAAHH-HHHBAHHAHBAHAHHAHBHHBBABBHAHBAAHHBABBHHHBHBAHBBBABBAHBHHHBBHA

*000731_00073851 BHHBAHBBBBAAHAHHHHHAAABABBHABHAHAABHHHAHBBBAAAHHBABAHHBBAHAHHHBHHHAHHAAABHAHAHAAAHAABHHAHHHHBABABHH-BAHHHHABAABHAAHBHAHHHHAABBHAHHHAHBHHHHBAHHHA

*001454_00184605 HHHBBHAHABBHBAAAHHAHBHHBAHAHHHABHAHBAAABHAAABHHAAAAHHBAHBHAHBHAHHHAHBBBHBHBBABBABH-BAHHAHHBHHHAHAHBAHHBBBBBHHAHBHHHBHHHBBBBBBHHABHBAHHHHBHHBHHBH

*001939_00071300 BHABAHABHHB-HHAHBHHHAHBHBAAAAHHAAHHABHHHAAHAHBHHABBHABHHBABAAHHBABABAAHAHABHHBHHBBBBABHHHBHAAHHABHAABBABBHBHHHHAHHHBAAHHHBBABAHHHBBAHHHHBHHBHHHA

*002227_00051358 HHABABBAHBHB-AAAAHHBAAHHHABHAHHHAHBHHABBHBHBHHBAHAAHHHHBAAHHHAHHABHABHBAAHAHAAHHHHBBHHHABHHAHAAAABHHHBHHAAHHHHHAHHHBBABBAAAABAAHHBAAHAAHBHAHHHBA

*000012_00050453 BAHBABBABABABAHBBHAAAHHBAAHBHAHBHBHAAAABHBAHBHBHHABABHAHBHHBBHBAABAHBHBAHHBH-HABHA-HHHAHHHHHAAHHHHHBHBAHHBABHHHHHHHABABBABBAHHAHHBBHHAHAHHHHAHAH

*000737_00537173 AHHHABAHHHHHBHAHHBBBBHBHHBHAAHHABHBHBAHHHHBHHHBHAHHABAHAHHBAABBBHBBBBBAAHHBA-HABHBBABAHB-ABAHBBHBAHHBHHAHHBHHHHHBBABABBABHHBHHHAABHBBAHHBHBHHHHB

*001111_00224995 AHBBHHAABBHBHHHHBBAAHHAAHHHHAAHHHHBHBHHBAAHAABBBAHBHBHHBHHBBHHAAHBBB-BAABAAHABBHHABHHHAHAHAHHAAHHHAHHA-HAAHHABBHBAAAABAHHHHHBBAHHBAHHAHABHABAHHH

*005638_00012096 AHAB-ABHAHHBBAHHBHBBHHHAABABHHHHHBHHBBHBAAAHAHHAHBAHHHABHBHBHAHBAHBHHHHABABAHAAHAH-HHBAHHBBAAAHHAAHAHAABBHHHHAHHHABHBAAHHAHHHBAHABBHHHAAHABHAAAA

*010335_00000372 HBHHHBHAHHABHBHAHBBAABHBBHHHBHBHAAHHHAHBHHHABAABAABBHHHHAHBHHBBHHAHB-ABHBAAHAHHAHABBBAHAHBHAAHHHABBHAAHHBAAHHAHAHHHBBBBBHHAA-BHBBHHHBHHBHBHBBHBB

*010944_00002208 AHHHAHBBAHHHBHAHHHABHHBHHHAHAABAHHBAAAHHBHAAHHBABHHBHBAABHBHAHBHAABAAHBAAHHH-HHAHBAABHHAAAHHHAHBAHHHHAHAAB-AHHAABBHABAHABABHHBHAHAAHHHHHBABHHABB

*000034_00338036 AHHH-BAHAHHHBHHABHBBBHBHHBHAAHHABHBHBAHBHHBHHHBHAHHABAHHHHBAAHHBHBBBBBAABHHABHABHBBHBBBBBAHAHBBHBAHBBBHAAHHHHHHHBA-BHBBABBHBBHHBAHAHBAAHBHBHHHHH

*000050_00037311 BHABABBBBBA-AAHAHAHBHABA--HABHHHBABAHHAHBH-AAAHHBHBAHHBBHBHAHHBHHHB-AHAABHAB-HAHH-AH-HBAHHHHB-HAHHHAHAHHBHBBHAAHAAHHBABHHAHH-BAH-HBABBAHH-HHBBHA

*000579_00251796 BHBHBAHHHBHB-AHAAHABAHHHAHHAHBABBHHHHABHBB-BHAAHAHH-HAAAHBAHBHB-BHHBH-AAHHAB-BHBHB-H-AHHAHABHHABBHBAH-ABHHBH-BBAHBHBHB-BBHHAHHBAHBBABAAHHHA-HHBH

*002756_00095392 AHBH-BHHAAABBBHHABBAH-BB--HHBHHAABHHAHBBAHAABABAHHHHBAH-ABAAABAHABBAHBHHBHH-HHBBHH-AHBH--HAHBHHHHHHBHAHHAAHBHHBBHBH-BHBHHBAA-HAHH-AHAHBBABHH-BAH

*001434_00198681 ABHHAAAHBBHHABHHABABBHA-HHBBAH-HHBHHAAHHHBA-BABH-HBBHB--ABHAHHHAHAHAHABHBHHHAAHAHHHH-AHHHBHABAHHAHBBHH-HAB-BHBBABHA-HBA-BBHBAABHHBAHH-B-H-HBHAAB

*000270_00174985 HHAAHHAHBBAHAB-BHBABAHHHHAHHHABHAHAHHHHBBHBHHAHHHHAAABBBHHAAHABAHABHHHHBHAAHAAAHBABHBAAHBAH-HHHAHAHBHAAAAABAAAHAHABA-HHBBBHAHBHBHHAHAHAHHAHHAHHA

*000550_00164664 BBBHAHBHHHBH-HBAHBBBAHHBABHBBAHBBBHAHBBHBHHABHHAHAHHHHB-HHHBBHHHAHHHBHAAHBHHHB-BBHBABHABAAHHHHBAHBAAAHHHAHAABHHHAHHAHHHHHHHA-HAABBAABBAABABAABBB

*000840_00185860 AB-BB-AA-H---H-AHAHHHBHBB-B-AH-HBBHHAAAA---BBAAHBBHAHBH-HHHHBAHHAHB--HHH-HHH-AABHBHB-BH-B-HAHABAABHH-AHHAA-ABHBHHBB---BHH--A-HHH--ABAA-HB-HHHA-B

*000841_00217699 HBABHHBABBH-AB-HBHBHAH-AH-HBBBAHBBHHHAAABHAHABAHHBBHH-AAHBABHBBAAHHH-A-AAHHHBAHHH-AH-AHAH-BHHBBHHBBAHHHAHA-HBHAAHHBBBH--B-HAAHHAHHHBHABBA-HAHAHH

*001579_00128599 BBHHAAHHBBHH-BHHABABBHH-A-BBAHBBHBHHAAHH-BAHHABHBHBBHBH-HBHAH-HAHAHA-ABHAHHH-AHAH--HBAHHHHHABAHHABBBHHAHAB-HHBBABHAABBABBBHB-HBHHBAHH-BAH-HBHAA-

*000141_00522119 BBHHAAHHB-HAABAHHBAHAAAHBABBAAAHHBBHAA-HHBHBHABHHHHHHBHHHBHAHAHAAHHA-AAHHHHHAAAHHHHBHABHHHBHHABHAHBBABAHAB-HHBAABHAAHB-BHHBABHHH-HAHBABAAHBHBAAB

*000873_00097332 HAHBAAAH-AAAHHBAHHHBBAHBBBHHHABBAHHBABHHAHBABBHAHBHHBAAHHHBABHBHHAAHAH-BAHAHAHABHAAHBBBHHAAAABHHHHHAHHBHHHBHBBHBHHA-AHAHHAHBBBHHHHHHA--BH-HABBAB

*001133_00275980 AHAAABBBAHH-HAAHHHBBHBABAHBBHAHAHHAAHBHHHHABBBHHAHAHHHHBAAAAAHHHHHABBHBAHHAH-AHAH-BHBHHHBBHHH-AAABHHAHAHHA-HHHBAAHHHBABBABAHHAAABHHHBBA-BHAAHHBA

*001553_00166458 BBHBBHHHHABA-AHBHBAAABABAABHHBBHH-ABHBAHHHBBBHBAAABHAHAAHAHHHHHBHAHH-BHAHHHHAHBABHBHHAHAHBAHBBBAHHAHBHABAA-HABHHAHHH--HHBAHAAHBBHHABHAHBHHAAHBHB

*000199_00362425 ABHAHHABBHHHHHHAHHBAABHHHBHBBBHHHBHAHHHAAAHBHBHBBAHHHAHAHAHAHBHBBHHHABHHBABBBHHABBHAHABHHHAHHHBBBABBHHAHBBBHAAHBBHABAHHAHHHAHAABBBBHBAHBHHBBBABB

*000410_00128980 ABHHHHAAAHHAHHHHBHHBBAHHBBBAAAHHAHABBAABBHBHHABHAHHHBHBHAHBAHBABBBBHAAHHHAHAABAAABBHAHHBBAHHHBBHBHHHHBBHAAHHBHHBAAABHHHABAABBHBBAHAHHAHHBAHHHHHH

*002641_00078067 AAHBAHBAAHHHHHHHAHHBBHBHBHABAABAHBBHAAHHBHAAHAHAHHHBABHABHBHAHBHAABAHHBAAHBHBABAHBHABHHAHAHAHBHHAHHBHAHHAHHAHHAHHBHBBHHABABBABHAHHAABHAHBABHHAHB

*002745_00036973 BBABAHHHBHH--BHAAAHHH-BHAHBHAHBHHBHABHHHAB-HHAAHHHHAHBHBABBAHHABHAHAAHABBHHHHABHHABHBBHHHBHBBAHAAHBBAA-HBH-HBBHABHAHABHBHB-HAHBHBABHHAA-BABBHHAB

*001039_00238783 HHHAHAAHB-HA-AHHAB-HHAAHH--HAAHAHABBHBBABHHBHHABHHHHB-BHHHHAAHHHHBB--H-HAHBHBB-HHH-B-AHAAAAHHABAHABHABHHHB-ABABHBBBBHHAABBHAAAABH-HHHHABB-B-HHAB

*001368_00035841 AABBHHAHBBBAAH-BHHAABHH-HAAHAHHHHBHBBAHB-AAHABHHAHBBHHHHHHHBHHHABABB-HAH-AAH-BBHH-BA-HHAAHHHH--HAHH-HA-HAH-BABBHBAAAABAAHBHBHA-HHA-ABA--BHHBHAA-

*001266_00036367 -AHHHH-B-AH-BB-BHABHAHB-H-HHHBBHAB-AHBHBBHBHHHHA--ABHBBBAHAHHHBBHAH--H-HHAAHBHHHHBHH--A-B-HHHHBAH-BBBBAAHA-BAABHAAB-H-HBHBAAHH-A-AHBH-A-H-BBA-BA

*000228_00025339 -ABHHBHA--B--AHBB-HAAHHHA-AHHAAHHBHAAHHB-BABAHHBBAHAA-AHBHHBHHBH-BAH-H-AAB-H-HA-H--HAHHHABBHA-H-AAHHHBBAHB-BHBHHAHH--ABBABBAB-AHH-HBHAHAB-H-BHH-

*001273_00027781 A-HBHBBB--BBAH-BBHBHBAH-ABBBBHHBA-HHAH-B-BAHHBHH-HAAHBAHBBAHBHHHBHBH-H-AHA-A-A-HB--B-AH-HHAAA-AHBAHBHAHHHB--HA-AHHHHBAH-HABB-BAHABHHHH-AB-HHHAH-

*000185_00565920 HHHHHHAHABBBBAAAHHHBBHHBAHAHHAABHABBAAABBAABBHHAAHAHHHAABBAHBHABABAABBHHHHBHABBABAABHHHAHHBHHHHBAHBAHHHBBBHHHAHBHHHHHHHHBBBBBHHAHHBHHAAHBAHBHHBH

*000419_00150085 HHABABBAAHHHHAAHHHBBAHAHHABHHHHAHHAABHBBHHHBHBBHHAAAHHHBAAHAAABHABAABHBAAHAHAAHHHHBHHHHABHHHHHAAABHBABHHAAHHBHHAAHHBBABBAHHABAAAHBAHBHHABHAHHHBA

*000494_00221255 BHHBAHHBHHBAHHHHBHHHAHBHBHHAAHHAAHHABABHAAHAHBHHABBHABHHBABAAHHHABABAAHAHHBHHBAHBABBABHBHBHAAHHABHAABBABBHBHHHHAHBHBHAHHHBBABAHHHHBABHHHBAHBHBAA

*000502_00262031 BHHBAHHBHBAAHABHHHHAAABABBHABHAHAABHHHAHHBBAAAHHBABAHHBBAHHHHHHHHHAHHAAABHABAHAAAHAABHHAAHHHBABABHHABAHHHHABAAHHAABBBAHHHHAABBHABHHAHBAHHHBHHHHA

*000645_00099300 BHABABBBHBAABAHHAAHBBABABAHHAHHHBAHABHAHHHHAAAHHBHBAHBBBHBHAHHHHHHHBHHAAHHABAHAAHHHHHHBAHHAHBAHAHHAHHAHBBAHBAAHBAAHHBABHHHHBHBAHBBBABBAHBHHHBBHA

*000645_00288156 BHABABBBHBAABAHHAAHBBABABAHHAHHHBAHABHAHHHHAAAHHBHBAHBHBHBHAHHHHHHHBHHAAHHABAHAAHHHHHHBAHHAHBAHAHHAHHHHBBAHBAAHBAAHHBABBHHHBHBAHBBBABBAHBHHBBBHA

*000848_00059859 HHHBABBAHABHBAHHBHHAHHHBHAHHHAHBHBHAAAABBBHHBHBHHABABHHABHHBBBBAAHAHBHBAAHBHBHABBHAHHHHHABHHHAAAHHHAHBHHBBAHAHBAAHHABABBABBHHHAHHBBHHAHAHAHAHAAH

*001097_00092262 BHBHAHBHHHHHBHBAABBBAHHBABHBBAHBBHHABBBHHHHABBHAHAHHHHBBHHHBBHHBAHHHBHAAHBHHBBABHHBABAAHAAHHAHBHBBAAAHHHAHHABHHHAHHHBHHHHHHAHHAAHBAAABAABABAABBB

*002101_00033592 HHAHAHBHHBHABAAHBHABHHHBBBAAHHHAHHBAAHBHAHABBBBABHABHBAAHHHHAHBBAHHAHAHHBHHHHHAHHBAABBAAAABHHHHHHBHAAABABAHAHAHAHBAHBBHABBHHHHHAHHBHHHABHBBHAAHB

*004620_00044403 HHHBABBAHABHBAHHBHHAHAHBHAHHHAHBHBHAAAABBBHHBHHHHABABHHABHHBBBBAABAHBHBAAHBHAHABBHAHHBHHABHHHAAAHHHAHBHHBBAHAHBAAHHABABBABBHHHAHHHBHHAHAHAHHHAAH

*000143_00109664 BAHBABBABABABAHBBHHAAHHBHAHBHAHBHBHAAAABHBHHBHBHHABABHAHBHHBBHBAABAH-HBAAHBHBHABBHAHHHAHHBHHAAHAHHHHHBHHBBAHAHHHHHHABABBABBAHHAHHBBHHAHAHHHAAHAH

*000550_00038151 BBBHAHBHHHB-BHBAHBBBAHHBABHBHAHBBBHAHBBHHHAABBHAHAHHHHBBHHHBBHHHAHHHBHAAHBHHHBABBHBABHABAAHHAHBAHBAAAHHHAHHABHHHAHHABHHHHHHAHHAAHBAAHBAABABAABBB

*000663_00189695 HBHHABHHAHHBBHHHHABAABHHHHHBBBHAABHHAHBAAAHHHBHHBAHHHAHHHHHHABHHBBBHBHBABABH-HBHBHAAABBAHAAHBBHBHABBHHAHBHBHAAHBBBABHABHBBHAHAAAHHBAAHABHAHBBHHH

*001519_00036631 BBHABAHHHAHABHBBBAABBBBHBAAHBHBBBHABAHBHHHAHHHHAHAAAHAABHHAABBBAHHHABBHHHBBHHHAAHAAABBAAAHAHHBHHHAAHHBBHAAHHAHHHBHHAHHABHHH-AHHAHHAHBAHAHBHBABHH

*002933_00091667 HAAAHHHHBBAHABABHBABHBHHBAHAAABHAAAHBHHBBHHHHAAHHHAAHBBBHHAAHHBHHABBHHHBAAAH-AAHBABHBHAHBAHHHHHAAABBAAAAAAHAAHHABAAAHHHBBHHHHBHBHAAHAHHHHHHAAHHH

*000165_00319020 HAHBAAHHHAAAHHHAHHBBBHHBBAHHHABBABHBAAHBAHBABBHAHBHHBAAHHABHBHBHHAAHAAABAHAB-HABHAAHBHBHHHAHABHHHAHHHABHBHBHBBHAHBAHHBAHBAHBBBHHBHHHAHHHH-HABBAB

*000663_00200344 ABHHABHHHHH-BHHHHABAABHHHHHBBBHAABHHAABAAAHHHBHHBAHHHAHHHHHHABHHBBBHHHBABABHBHBHBAAAABBAHAAHBBHBHABBHHAHBH-HAAHBBBAHHABHBBHAHAAAHHBAAAABHAHBBHHH

*000914_00087191 ABHH-BAHAHHHBHAABHBBBHHHHBHAAHHABBBBBAHBHHBHHHBHAHHABAHHHHBAAHHBHBBBHBAABHH-BHABHBBHBHBBBABAHBBHBAHBBBHAAHHHHHHHBAABABBABHHBBHHBAHAHHAAHBHBHHHHH

*000400_00416946 HBHHAABAB-HH-BHHHBABBHAHBHBBAHHHAB-HA-HHHBAH-ABHBHBHHBAAHBHAHAHHHAHAHAAHAHHHHAAHH-AHBAB-ABBHHABHAHBBHHAHABHAHBAABHA-BBABHHH-HHBHBHAHBABHHABHBAA-

*002122_00111722 BBHHHBHHHAAHHH-AH-BAABB-B-BH-BAHAAHHHABA-HHHBAAHAAHBHHHHAHBHHA-AHAHB-AHHBAAH-HBAHABB-AAABBHAAHHAAHBH-AHHHB-A-AHAHHH-HBB-B-AAAHHBHHHBB-HBHBA-AHB-

*000383_00134404 ABBABABA-HH-AAHAAHHBABBHBHAHHHHHHHBHABHHHABHHHHHAAHBA-A-AAHBHHHHAHH-AAABBHBBBA-AHH-A-BBHABHHAAHH-BBBAH-HHB-HBHBABB-B-HA-A-HA-H--BHHAHHB-H-HBBBHH

*000419_00168638 HHABABBAAHHAHAAHHHBHAHAHHABHHHHAHHAABHBBHHHBHBBHHAAAHHHBAAHAAABHABAABHBAAHAH-AHHHHBHHHHABHHHHAAAABHBABHHAHAHBHHAAHHBBHBBAHAABHAAHBAHBHHABHA-HHBA

*001806_00088826 HHHHAAAB-HABHBHHBAHHAHHHA-HHHAHAHABHAABABHAHHHHHAAABAABHHBBHABHHHABBAABHABAHAHHHHAAHBAHBBBAHHAHABBHAHBHHHHBBBAAHAHHHABAAAABHHHBBAHHHABHBHHHAHHBA

*001939_00110967 BHABAHABHBBAHHHHBHHHAHBHB-AAAHHAAHHABHHHAAHAHBHHABBHABHHBABAABHBABABAAHAHABHABAHBHBB-BHHHBHAAHHABHAABBABBHBHHHHAHHHBAAHHHBBABAHHHHBAHHHHBHHBHHHA

*000000_01441139 HAHHBHHHBBHBABHBBBHBHHBAH-BHAAABHBHBBHAAABHHABBHAAHAHHHAAHHHBBBBHABBAAHBHAHHHAHHAABHHBHHBAABH-BAHBBBAA-ABAAHHBHBHHAAHHHHHHHHAHBBHHBHHHBBHBBAHBHH

*000034_00013186 AHHHABAHA-HBBHAABHBBBHHHH-HAAHHABHBHBAHBHHBHHHBHAHHABAHHHHBAAHHBHBBBHBAABHHAHHAHHBBHBHHBHAHAHBBHBAHBBBHAAHHHHHHHBAABABBABHBBBHHBABAHHAABB-BBHHHA

*001207_00158686 ABBABABABHBHHAAAAAHBAHBHHHAH-AAHHHBHAHHHBA-HHB-HAAHHHAAAHHHBHHHAHHHB-HABBHBBBABHHBHBHBBAABHHA-HHHABBAHHHHB-AHHBABBBB-HAAAHH-BHHBB--AHHB-A-HBBHH-

*002320_00054549 AABABAAHB-BHBBHBHHBBAABAH-HBHHBHHAAHHHHHHHBBAAH-HBBAAHH-HHHAHA-ABHHB-HBHAAHBA--ABB-BAAHAHHAHAHAHAABHHHBBHHAAHBHHBHHH-BAHB-H--BHABBHHABHAHHAHBHBB

*000294_00229071 AAHBHAB-AHH--AAAAA-HBAH-HHAHHABHHBHHHAHBAA-AAHBHABHABAB-BHHAHBHBHAB--H-ABBAH-HAHHA-H-AA-AHHAA-HAHABHHB-HH--AHBHHHHA-BHAHBHHB-BAHHH-BBBAAH-HHABHB

*000215_00023780 AHHBAHABA-HABBAAHBBHHHBHABBHHBABAABAHHABHBBHHHHHAAAABBHAHHHHBAAHBHBHAAHAHAHBAHHHHAHBHAHHHHHAA-AHBHBBHHHHAHAHBBBHBHABAABABHHABBAH--AHAAH-HBHHHAHH

*001648_00108742 AHHBHHABAAHABBAAHBBAHBHBHBBHHBABHABHHHABBBBHBHHHAAAABHHHHHHHBAAH-HBHAAHAAABBAHHHHA-BHAHHHBHAABAHBHBBHHHHAHAHBBHHBH--A-HABAHHHBAHBBAHAAHBHBHHAAHH

*002528_00169032 AHHBBAHBHBBHHH-AHBAHBBHHA-HAHAAHHAHBH-AHHH-AHHHHAAAHABAHBHAHHAAHHHAABBBHBHBB-BHABA-B-AHHH-HHHBA-AHBAHABBBBAHHAHBHHAB-H-BHBBBBBHABHBHH-A-BBH-HBHH

*004917_00038097 HBHBHAHBHB-H-B-H-AAHBHAAB-ABB-AHHHHB---BHAABAHHBBBBHHHB-HBHAHAABHHHABBBHHHAHHHHHHBAB--BHA-BBB-HBHHHABB-HH--AHA-BHHHBAA-BABHBAABHA-HHHA-BH-HHBB-H

*000140_00070373 HBBBBBHABHABABBAHHHHHBHBH-BHAHAHBBHHAAHABHBBBAHHHBBAHBHHHHHHHAHHAHBBHHBAAHHHHAAHHB-BABHBBBHHH-B--HHHHAHHAABABHBAHBHAAABBHAHHHHHH--ABAABBBBHBHAAB

*000006_01075342 H-HHAABH-HA-ABBHHBHBHHBBAABAABBHHABBHA-AHAHHBBAHHAAHAHH-HBHBBH-BBHAB-HAHBHAABBHA-HBAABBAHHBBH--BAHHHHHBHBBBAAHHAHB---HAAHHHHBBHAAHHAB-H-H-HHBHHH

*001478_00022019 A-A-AHBH-AH--AABHHBBHBA-A-BBBAHAAHAAHHBH-HHBBBHHAHAHHHH--AAAAHHHHHA--HBAHHAHABHAHH-H-HHABHH-HAAAABH-ABAHAABHBHBAHHH--A--A-AA-AHABH-HB-BHB-A-HHHA

*000099_00557265 HBBABHHHHAHHAAHHHBABHAHHBHBAHHBABHAAAAHAAHBHHAABBHAAABAHBHHBAABHHAAHHAHHHHAABHHBBHAHABBHBBHHAHHAAHHHHHHHHAABHBBAAHBABHHAAHBHHHHAABHBAABABHHABHBH

*000099_00565124 HBBABHBHHAHHAAHHHBABHAHHBHBAHHBABHAAAAHAAHBHHAABBHAAABAHBHHBAABHHAAHHAHHHHAABHHBBHAHABBHBBHHAHHAAHHHHHHHHAABHBBAAHBABHHAAHBHHHHAHBHBAABHBBHABHBH

*000143_00712611 HAHBABBAHABABAHHBHHAAHHBHAHBHAHBHBHAAAABHBHHBHBHHABABHAHBHHBBHBAABAHBHBAAHBHHHABBHAHAHAHABHHAAHAHHHHHBHHBBAHAHHHHHHABABBABBAHHAHHBBHHAHAHAHAAHAH

*000163_00583842 HBAAABHAHBHHABAHBHAHAHHHBBHHAAAAAHAHBAAAABHHHBBBHHBBAAHHBHBHAAHHHHBABABHBHBHAHAHHHHAAHHHHHHBBBBHHBHHBAHHBBABBAHBBBAHHHBHABBAAHHBHHBBHHAHHHBHHBAA

*000218_00070204 BBBHHBHAAHBABHHBBBHAAHHBAAAHAAHHABBAAHHHHBABBBABBAAAABHHBHHHHHBHABAHBHHAHABAHHABHAAHBBHHHBBHAAHHAABHHBHAHHHBHBHHAHHAHHBBABBAHHAHABHBHAHAAHHAHHHH

*000218_00076794 BBBHHBHAAHBABHHBBBHAAHHBAAAHAAHHABBAAHHHHBABBBABBAAAABHHBHHHHHBHABAHBHHAHABAHHABHAAHBBHHHBBHAAHHAABHHBHAHHHBHBHHAHHAHHBBABBAHHAHABHBHAHHAHAAHHHH

*000248_00200058 AHABHABHAHHBBAHHBHBBHHHAABHBHHHHHBHBBBHBHAAAAHHAHBAHAHABHBHBHAHBAHBHHHAABABAHAAHAHHHHBAHHBBAAAHHAAHHHAABBBBHHAHHHABHBAAHHHHHABAHABBHHHAAHAHHAAAA

*000339_00133155 AHHAABAHABHBAHHBHHHHBHBHABHAHHBHHABAHAHAHHAHHABHHAAABHHHBHAHHBBBHAHHBBBBAHABAHHBHBHHHAHBABHBBAHAABBBBHAHHHHBBAABAAHHAABHHABBBAAHBHHHHHABHAHAHHBH

*000599_00184417 HBBHBHBHBAHHAAHHHBABHAHHHHBAAHBABHAAAAHAABBHHAABBHAAABAHBHHBAABHHAAHHAHHBHHABHHBBHAHHBBABBHHAHHAHHHHHHHHHAABHBBAAHBABHHAAHBHHHHAABHBAABHBAHABHHH

*000645_00007336 BBABABBBHBAABAHAAAHBHABABAHHAHHHBAHABAAHHHHAAAHHBHBAHBBBHBHAHHHHHHHBHHAAHHABAHAAHHHHHHBAHHAHBAHAHHAHHAHBBAHBAAHBAAHHBABHHHHHHBAHBBBABBAHHHHBBBHA

*000920_00197928 AHBBHHAABBHBHHHHBHAAHHAAHHHHAAHHHHBHBHHBAAAAABHBAHBHBHHBHHBBHHAAHBBBABAABAAHABBHHABHAHAHAHAHHAAHAHAHBABHAAHBABBHBAAAHBHHHHHHHBAHHBAHHAHHBBABAHHH

*000389_00338373 AABHABHHABHHAHHBHBHHBHBHAHAHHHBHHAHABAHAHBBAHHBAHAAABHHHHAAHHBBBAAAHBBHBAHHBABHBHHHBBAHHABHBBAHBABBBBHAHHH-BBHABAHHHHABBHAHBBAAHBBHBHHHBHAHHHABH

*000481_00183894 AAHBABBAHABABAHHBHHAAHHBH-HBHAHBHBHAAAABABHHBHBHHABABHHHBHHBBHBAAHAHBHBAAHBHHHABBHAHHBAHABHHAAHAHHHHHBHHBBAHAHHHHHHABABBABBABHAHHBBHHAHAHAHAAHAH

*000775_00066264 AABHABHHABHHAHHBHBHHBHBHAHAHHBBHAHHABHHAHBBAHHBAHAAABHHHHAAHHBBBAAAHBBHBAHHBABHBHHHB-AHHABHBBAHBABBBBHAHHHABHHABAHHHHABBHAHBBAAHBBHBHHBBHAHHHABH

*001137_00043707 HHAAAHHBBBA--B-BHHABAHBHH-HHAHHHAHAHHBHBBHBHHAHAHHABAHBHHHAAHABAHABBHBBBHAAHAAABAABHHHAHBAHHH-HAHAHBHHAABA-HAAHAHABHAHHBBBHA-BHBHHHHHBA-HHBBABBH

*001278_00125693 AHBHHBHHHAA-ABHHAB-AHBBBABHABHHAHBHHAHBBAHAABABAHHHHBAHBABAAABAHABBA-HHHBHHH-HBBHHAAHBHBBHAHBHBHHHHBHA-H---BHHBBHBHHBHBHHBAHAAAHH-AHAHBAABHHHBAH

*002745_00036887 BBAHAHHHBAH--BH-AA-HHBB-HBBHAHHHHBHABHHHABHHHAAHHHHAHBBHABBAHHABHAHAAHA-BHHH-ABHBABHHBHBHBHBBAHAAHBBAAAHBHBHBBHABHAHHBHBHBAAAABHHABHH-H-BABBHHAB

*003227_00002830 ABHBHHBHHHABHBBABHHHHHHBHBHHBHHHBAHHBH-B-BHBBABBABHHABHBBABHHBHHBHH-HBA-BBAH-AHHBBHB-HHABHBHBBHBHHBAHBHAHA-BBHBAHHAAB-AHHABHHBBBAAHHHBBHB-HABAB-

*000645_00242586 BHABABBBHBA-BAHHAAHBBABABAHHAHHHBAHABHAHHHHAAAHHBHBAHBHBHBHAHHHHHHHBHHAAHHABAHAAHHHHHHBHHHAHBAHAHHAHHHHBBABBAAHBAAHHBABBHAHBHBAHBBBABBAHBHHBBBHA

*000810_00234625 HHHHAAABAHHBAHHBBHBAHHHHAHHABHBAHABHHABABAAHHAHHAAHAAABHBBHHABBHHABBHHBHAHAH-HHBAHAHHAHBHBHHBAHABBHAHBHHHHBBBAABAAHHHBAAAABHHHBBHHHHABHBHAAAHHBA

*001886_00061238 HHAHHHBHHBH-BAAABAHHAHHBBBAABHHHHAHAHABHHAABBHBAHAHHBBAAHHAAHHBHHAHAHAHHBHHHHHAHHBAHHBAAAABHHHHHBBBAAHBABABAHAHAHBAHHBHABBHAAAAHHHBHAHABHBHHAABB

*002389_00111082 HHHHAAABHHABABHHBAHHHHHHHBHHHAHAHABHHABHBHAHHHHHAAABAABHBBBAABHHHABBHABHBBAHAHAHHAAHHAHBBBAHHAHABBHAHBHHBH-BBAAHAHHHABAAAABHBHBBHHHHABHBHBAHAHBA

*007160_00000954 HHAHAHBHHBHABAAHBHHHHHHBBBAAHHHAHABAAHBHAHABBBBABAHBHBAAHHHHAHBBAHHHHAHHBHHHAHAHABAABBAAAABHHHHHHBHAAHBABA-AAAHABBAHBBHABBHHHHHHHHBHHHABHBBHAAHB

*000191_00235881 BBHBHHHHBHBAHABHHBAAABABAABHHBBHHBHHHHAHHHHBBHBAAABAABHAHAHHHHHBHABHAHHAHABH-HBAB-BHHAHHHBABBBHABHAHBHABAAAHABHHAHHBHHHBBHHABHBBHAABHAHHBAAAHBHB

*000826_00247608 BBBAHHBHHHBABHBAHBBBAHHBABHBBAHBBBHAHBBHHH-ABHHAHAHHHHBBHHHBBAHHAHHHBHAAHBHHBBABBHBABHABAAHHHHBAHBAAAHHHAHHABHHHAHHHBHHHHHHAHHAA-HAABBAABABAABBB

*001284_00117143 HAHBAABAHHHAHBHAHBHBBBBHBBHHHHHBBBBHAAHB-AABHAAHHAAHBBHBBBHHBHHHAABHAABHAAHB-AHBBBHAHAHBBHAAHAAAHHBHHHABHHBHAAAHAHABHBAHHHBBAHHHBHHHBAHHAHHBHAHH

*002337_00089857 HBAHAHHABBHBHH-HBBABHHHHB-HHAAHAAAAABAAAABHAHHBHAHAHAAHHBHBBAAHBHHHAHABHBHBHABHHBBHAAAHHBHHHBBBBHBHBBAHHBBABBAABBHHBHHHHABBHABHBHABHHHHHHHBHHBHA

*009260_00003173 AHHHABHHHAABHBHHAHBAAHHBHHBHBHHAHBHHAHBHAAAHBAHABABHHAHBABHAABHHHBBHHHHHBHBHBABHBHAAABBHHHAHBHHBHHBBHAHAAHBBAAHBBBAHHHBHBBAA-HAAHAAHAHABA-HHBBAH

*003744_00043875 B-HHHHHHHAH-AHBBAHAHH-A-B-BHAA-A-HBBH-HHHH-BBHHH-ABAHBAABHBB-HBB-HBH-A--HBAB-HHABHBH-AHAABHAAHAHAAHBH--HA-AHAAHBBA-HHH-ABABHAHHB---AHABAB-BB-HHH

*000006_00433063 HHHHAAHHBAAAHHBHHBHHHHB-HABHABBBHHHBH-AAHHBHBHHHHAAHAHHAABHBBAHHBHAH-HAABHAA-BHAAHBAABBAHHBBHHABAHHAHHBAHB-AAHBAHBA-AAA-HBAHBHHAA-HABAHAHBH-BHH-

*000359_00345240 -HAHAAABAHA-HBBHBAHHHABHHBHHHAHAHA-HHABHBHAHHHHHAAABAABBBBBAAHHBHABBHAB-HBAHAHHHHA-H-AHBBBAHHAHABBHAHB-HH-HBBA-HAHHAABAAAABHHABBHHHAH-HBH-AHHHHA

*000529_00330936 B-BHBAHHBBH-BAHHHHAAAHHAAHHAABABBHHHHBHH-BHBAAAHAHAAHA-AHHHHBHBHBHHBHBAAAHAB-BHHHH-BAH--AAABBHHBBABABH-BHHAHHBBAHHH-HBAHBAHAHHBABBHAHAAAHHABHHB-

*000383_00423007 ABB-BABA-HHB-AHAABHBA-BHBHAHHHHHABBBA-BHHAHHHHHH-AHBAAABHHBBHBHHAHHHAHABBHBB-AHHHAAHABBBABBHAAHHHBBBAHHHH--HHHBABBB--HAAAHHABAHHBHHAHHB-HAHBBBHH

*000038_00782736 BB-ABBAH-HH-AH-AHHHHAHH---HBAHAHH-HABBHHHBHBBHAABHHH-AABBAAAHBHHBHH-BBBHBHBHAHB-H--BBBHHHBHBBBAAHHBAHHAHHBBABBAHBAA--B-BBBBH-HHAHAHBHAH-HAA-AHH-

*010952_00000823 HHABABBBAHA-BAHHHHHBBABABAHHAHHABAHABHAHBHAAAAHHBHBAHBHBHHHAAHHHHABB-HAAHHABAAAAHHHHBHBAHHAHHAHAHHAHHHHBBAHBHAHHAAHHHA-BHHHBHBAHBBBABHAHBBHBBBHA

*000091_00852092 BAHABAHHBHBH-BBBHHHAAHBAHBHBABHHHAHHHHAHHHBBAAHHHBBAAHH-BHHAHABABHHB-BBHAAHBABHAHHABAAHAHHAAAHAHAABHHHBBHHAAHBHHBHHHHBAHBABAABHABBHHABHAHBA-HHBB

*000400_00381909 HBHHAABHBBHAABHHHBABBHAHBHBBAHHHHBHHAAHH-BAHHABHBHBHHBHHHBHAHAHAHAHAAA-AAHHHHAAHHAHHBABHHBBHHABHAHBBHHAHAB-HHBAABHAABBABBHABHHBHHHAHBAB-HHBBBAAB

*001469_00254288 HBHHAAHHBBHHABAHABABBHHAHABBAHBHHBHHAAAHHBAHHABHBHBBHBHHABHAHHHAHAHAHABBAHHH-AAABAHH-AHHHHHAB-H-AHBBHHAHABHHHBBABHAAHHABBBHBHHBHBBAHHABBHAHBHAAB

*002310_00046438 AHHHAHBBAHHHBHAHHHABHHBHHHAHAABAHHBAAAAHBHAAHHBABHHBABAABHBHAABHAABAAHBAAHHHBHBAHBAA-HHAAAHHHHHBAHHHHAHAABHAHHAABBHHHHHABAB-HBHAH-AHHHBBB-BHHABB

*004318_00029266 AHHHAHBBAHH-BHAAHHABHHBHH-AHAABAHHBAAAHHBHAAHHBABHHBHBAABHBHAHBHAABA-HBAAHHHBHHAHBHABHHAAAHHHHHBAHHHHAAAABBAHHAABBHHB-HABABHBBHAHAAHHHHHBABHHABB

*000484_00037692 HAHHBHHH-BH--B-BBBHHHHBABABHAAABABHBBHAAHB-HABHHAAHHHHHAHHHHBBBBBABHAA-B-AHHHAHHAABHHAHHBAABHHBAHBBHAABABA-HABBBHBAA-HHHHHHH-HBBH-BHHBBBH-BHHBH-

*001449_00120639 BAHBAHHBHHHAAHHHBHHHAHBHB-HAH-HAHHHABABHAAHAHBHHAHBHABH-BABAAHHHAHAB-AHAHH-H-BAHBA-BABHBHBAHA-AABHHABBABBBBBBHHHHB--BABHHBBA-AHHHHBABBH-HHH-HBAA

*000194_00190297 ABHAH-AHBHHAHA-AAHHHAAAHBHAHAABHHHBBH-BAHAABHHAHA-HHHHBBHHBAHHHABBBH-HHHAHBH-BBAHA-BAHHHA-AHB-B-HBBBABHHHB-AHABHBHBB-HAAHBBAAAAHABHHHBHHH-BBBHB-

*002297_00036872 AAHHHHHH-BHHABHBHBABH-HAA-HAHHABABHHBHBA-B-HHHAHHAAAHBBHAHHHBBBBHABB-BHBAAAH-AAAB-BH-HHHHAAHHHHAAABBAABAAA-HABHABHAA-BHBHHAH-HBBH-AHAHAAB-HHHHHH

*000681_00244964 -BH-HHBA---B-A-BHABAAAHHA-AABABBA-BH---HHH-HHHAHHBBABAA-H-BHBAHAABHH-H-AAAHH-B-BH-HHB-HHAAHA--H-BHBHHH-AB--H-HAHHAH-BH-AA-HAHBH-A--AHAABH-H--A--

*001644_00026945 AHHHABAHAHHA-H-ABHBBBHHHHBHAAHHABHBHBAHBHHBAHHBHAHHABAHHHHBAAHHBHBBBBBAABHHA-HAHHBBHAHHBHAHAABBHBAHHBBBAAH-HHHBHBAHBABBAB-HBBHHB-HABHAABBHBHHHBH

*000046_00452826 AABHABHHABHHAHHBHBHHBHBHAHAHHBBHAHHABHHAHBBAHHBAHAAABHHHHAAHHBBBAAAHBBHBAHHBABHBHHHBBABHABHBBAHBABBBBAAHHHABHHAHAHHHHHBBHAHBBAAHHBHBHHBBHAHHBABH

*000381_00506573 ABHAHHABBHHHHHHAHABAABHHHBHBBBHHHBHAHHHAAAHBHBHBBAHHHABHHAHAHBHHBAHHABHHBABBBHBABHHAAABHHAAHHHBBHABBHHAHBBBHAAHBBHABAHHAHHHAHAABBHBHHAHBHABBBABB

*000406_00092587 ABBABAHHHAHBAAAHHBABHBHHBBBAHHBHBAAAAAHAAHBHHAAHBHAAABAHBHHBHABBHAAHHAHHHHAABHHBBHAHABBHHBHHAHHAAHHHHHHHHAABHBHAAHBAHHAAAHBHHHHAABHBAABHBHHABHBH

*000410_00181187 ABHHHHAAAHHAHHHHBHHBBAHHBBBAAAHHAHABBAABBHBHHABHAHHHBABBAHBAHHABBBBHAAHHHAHAABAAABBHHHHBBAHAHBBHBHHHHBBHAAHHBHABAAABHHHAHAABBHBBAHAHHAHHBAHHHHHH

*000899_00082730 ABHAHHABBHHHHHHAHABAABHHHBHBBBHHHBHAHHHAAAHBHBHBBAHHHAHAHAHAHBHBBAHHABHHBABBBHHABHHAHABHHAAHHHBBBABBHHAHBHBHAAHBBHABAAHAHHHAHAABBHBHHAHBHABBBABB

*000011_00049376 A-BBABBHAHA--AHHBBHBHHHBHABBBAHABHAHBAHHBH-HBHHHAHAHBBHBAHHAAAHHHHB-BBBAHHAH-ABAHA-HHHHAHHHAA-AAABAHABABBHHHBHHHHAHABABBHAAHHAHAB-BABHAHBBHHHHHA

*000254_00317739 HBBBAAHHBBH-HBBHHAHHBHAHHHHBBBHHAA-BAHAB-HHBAABBBBHHHBHABHHAHHABBHHH-BBHBBAAAAHAH--B-AHA-HBBBABBHHHABBHHHAHHBABHHHAAAAAHHABBHHBHHAHHAAHHHAHBAHBH

*000576_00203536 BBBAHHBHHHBA-HBAHB-BAHHBA-HBBABBBBHAHB-HHHAABHHAHAHHHHBBBHHBBAHHHHHHBHAAHBHH-BABBBBA--ABAAHHHHBAHBAAABHHAABABHHHAHH-HHHHHHHAHHAAHHAABHAABHBAABBB

*001001_00178483 BAAAABHHBHHH-HHHAAHAHBHABHBHAAHBH-AHBHBHA-HHHAAHHHHAHBH-ABBABHAHHHHHAHAB-HHBHABHB-BH-BHHHBHBBABAAHBBHAAABHAABHAABHHABBBBAAAAAHBHBABHHHH-BABBHHHH

*001274_00212638 BBAHBHBHHABA-AHBHBAAABHBA-BHHABHABABHHAAAHHBBBBAAABHBAHAHAHHHBHBHHHH-H-AHHHH-HBABHBH-AHHHBAHB-AAHHAHHHABAAAHABBHHABBHHHHHAHAAHBBHHABHAAHH-AAHHBB

*000079_00443546 HHABAAHBAAAAHHAAHHBBBAAHHBAHHABBAHHHAHHHAHBAHBHAAHHHBAAHHHHABBHHHABHAAAHAHAHAHAAHAAHBHAAAAAAABBAHABAABBHHHBHHBHHHHAAHHAHBAHBHBAHHHHHHHAAHBHHHBAB

*000126_00467580 HHHHHHAAABBBBAAHHHHBBHHBAHAAHAAHHABBAAHBBAABBHHAAHAHHHAABBHHHHHBABAABBHHHHBHHBBABAABHHHAAHBBHHABABBABHHBBBHHHHHBHAHHHHHHHBBBBHAAHBBHHAAHBAAHHHBH

*000129_00322035 BHHBAHBBHBAAHAHHHHHAAABABBHABHAHHABHHHAHHBBAAAHHBABAHHBBAHHHHHHHAHAHHAAABHABAHAAAHAABHHAAHHHBABABHHABAHHHHABAAAHAABBBAHHHHAABBHABHHAHBHHHHBHHBHA

*000494_00075376 BHHBAHHBHHHAHHHHBHHHAHBHBHHAAHHAAHHABABHAAHAHBHHABBHABHHBABAAHHHABABAAHAHHBHHBAHBABBABHBHBAAAHAABHAABBABBHBHHHHAHBHBHAHHHBBABAHHHHBABHHHBAHBHBAA

*000494_00210943 BHHBAHHBHHBAHHHHBHHHAHBHBHHAAHHAAHHABABHAAHAHBHHABBHABHHBABAAHHHABABAAHAHHBHHBAHBABBABHBABHAAAHABHAABBABBHBHHHHAHBHBHAHHHBBABAHHHHBABHHHBHHBHBAA

*000548_00225934 BHHBHBBHBBBHBAHBABHABHABBBBHHBBBBHHABHABBBBHBHBHHHBHAHAHHHHHBHBHHABBHHHABABHAAHHBHBHHAHBHBAHBBBHHHBHBHABAHAHHHHHAHHBBBBBBHHBBHBBHHABHAHBBBAAHBHH

*000584_00241550 HHAHHHBHHBHABAAABAHHHHHBBBAABHHAHAHAHABHHAABBBBAHAHHHBAAHHAAAHBHHAHAHAHHBHHHHHAHHBAHBBAAAABHHHHHBBBAAHBABABAHAHAHBAHBHHABBHHAHAHHHBHAHABHBHHAABB

*001170_00016049 BHBAAAAAABAAAHHHBBHBHBHHHHABHHHAHBHHHBHHHHBBHABBAHAHBABHBAAHAABHAHBAHBHABBAHHHHHHABHABHBBHAHHABHHBBBHHAHBAHAHBHBHHHHHAAABBBHABHHHHBBBHBABBAHHAHB

*001225_00154749 HHBAAAAAABAHAHHHBBBBHBHHHHABHHHAHBHHHBHHHHBBHABBAHAHBABHBAAHAABHAHBAHBHABBAHHHHHHABHABHBBHAHHABHHBBBHHAHBAHAHBHBHHHAHAAABBHHAHHHHABBBHBABHABHAHB

*001323_00050197 BHHBAHABHHBAHHHHBHHHAHBHBHHAAHHAAHHABAHHAAHAHBHHABBHABHHBABAAHHHABABAAHAHABHHBAHBABBABHBHBHAAHHABHAABBABBHBHHHHAHBHBHAHHHBBABAHHHHBABHHHBAHBHBAA

*001598_00022150 BHHAAHHAABAHAHBHBBBBHBHHHBABHHHAHBHHHBHHHBBBHABBAHAABABHBAAHAABHAHBAHBHHBBAHBHHHHABHABHBBHAHHABHHBHHHHHHBABAHBHBHHHHHAHABBBHAHAHHHBBBHBABBABHABB

*002400_00039266 BHHABBAHAAHHBHAABBHHAHHBHAHBAHAHHHHABBHBHBAHBAAAHHBHHBAHBAAHHBHHBAAHBHBHBBBHHHBHBABHBBHHHHAHBBHHAAHAHHAHABHHBBBABHABABBHBHBAAAHAHAABBAHHAHAHHHHH

*002445_00055436 HHHBBHAHABBHBAAAHHAHBHHBAHAHHHABHAHBAAABHAAABHHAAAAHHBAHBHAHBHAHHHAHBBBHBHBBABBABHABHHHAHHBHHAAHAHBAHHBBBBAHHAHBHHHBHHHBBHBBBHHABHBAHHAHBBHBHHBH

*004976_00028071 HHBBHHAABBBBHHBHBHAAHHHAHHHHAAHHHHBHBHBBAAAAABHBAHBHBHHBHHBBHHAAHBBBAHAABAAHABBHHABAAHAHAAAHHAAHAHAHBAHHAHHBABBHBAAAAHHHHHHHHBAHHHAHHAHBBHABAHHB

*005797_00009136 HHAHAHBHHBHABAAHBAHHHHHBBBAAHHHAHABAAHBHAAABBBBABAHBHBAAHHHHAHBBAAHAHAHHBHHHHHAHHBAHBBAAAABHHHHHHBHAAHBABABAAAHABBAHHBHABBHHHHHHHHBHAHABHBHHAAHB

*000223_00308309 AHBBHHAABBHBBHHHBBAAHHAAHHHHAAHHHHBHHHHBAAHAABBBAHBHBHHBHHBBBHAAHBBBABAABAAHABBHHABHHHAHHAAHHAAHHHAHHABHAAHHABBHBAAAABHHHHHHBBAHHBAHHAHAB-ABAHHH

*000429_00237506 AHBBHHAABBBBHHHBBHAABHHAHAAHAAHHHAHHBAHBAAAAABHBAHBHHHHHHHBBHHHAHABBAHAABAAHABBHHABHAHAAAHAHHHAHAHAHBAHHAHHBABBHBAAAAHHHHBHBHBAHHHAHHAHHB-ABAAHH

*000857_00096252 AHHHAHBBAHH-BHAHHHABHHBHHHAHAABAHHBAAAHHBHAAHHBABHHBABAABHBHAABHAABAAHBAAHHHBHBHHBHABHHAAAHHHHHBAHHHHAHAABHAHHAABBHBBAHABABHABHAHAAHHHHHBABHHABB

*001569_00014449 BAHAAHAAABHA-HHHBBBBHBHHHHABHHHAHBHHHBHHHHBBHABBAHAHBABHBAAHAABHAHBAHBHABBAHBHHHHABHABHBBHAHHABHHBBHHHAHBAAAHBABHHHHAAHABBBHAHHHHHBBBHBABBABHAHB

*001111_00285590 AHBBHHAABBH-HHAHBBAAHHAAH-AHAAHHHHBHBHHBAAHAABBBAHBHBHHBHHBBHHAAHBBBABAABAAHABBHHABHHHAHAHAHHAAHHHAHHABHAAHHABBHBAAAABHHHHHHBHAHHBAHHAHBBAABAHHH

*000064_00431717 HHHBABBH--A--AHHABBBH-HBA-BHHAHABHHHB-HHBHBHBHHH-HAAHBB-HHHAAHHBHABBBBBAHHAHAABABHBH-BB-HHAHHAAAAHAHAHABBBAHBAHHAHH-BH-BHH-HBAHHABBABBA-BHHHBHH-

*000066_00478099 H-HHBHBHA-AHABABAH-AABHHB-HHBHBAAHHBHB-BBHB-BAHAABHBAHBBAHAHABBHHAAA-B-ABAHA-HHHHHHH-BHBB-HHHHHHHBHBHH-AHA-H-ABHHABABAHBABAAH-AAABAHB-HHHH-BHBHH

*000134_00290847 BAHHHAHA--BA-A-HHB-BHAB-H-AHHAAAHAABH-HHHBBHHH-HBAHBB-BBHAHBAHHABHH--A-ABAHH-B-HAA-B-AHBBAABH--BBAAABH-BA--A-HHHHAAHH-AHB-HBHHA-H-HHB-----HHBAH-

*002640_00001606 BBBHAHBAHHB-BHBAABBBAHHBABHBBAHBBHHAHBBHHHHABBHAHAHHHHBBHHHBBHHHAHHH-HAAHBHHBBABH-BABH-B-AHHAHBHBBAAAHHHAH-ABHHHAHH-BH-HHHHA-HAAH-AAHBAABABAABBB

*000316_00014946 HHABHAHA-A-BAHAABBABB-HHA-AHAABHA-BABBABAAHHHBHHHHHHBH-BHAHHH-BHABBA-H-HHBHA-HHAHH-HABBHHHH-AAHBHAHAHB-HAAHHHBBBBHA--HAHB-HB-BBH-HABB---H-A--BH-

*000111_00462468 HHHHHAHHHAHBAHHBAHAHHHABBBBHAAHAAHBBHHHHBHABBHBHBHBABBAABHBBAHBBHHBH-AABHBABAHAHBHBHHAHAABHAHHAHAAHHHHHHAH-HHAHBBAAHHHAABABHAHHBBBAAHABABABHHHAH

*000522_00239240 HHABABBAAHH-HAAHHBBBAHABHABHHHHAHHAABHBBHHHBBBHHHHAAHHHBAAAAAAHHHBABBHBAHHAHAAHHHHBHHHHABHHHHHAAABHBABHHAAHHBHHAAHAHBAHBAHAABAAAHBAHBAHAB-AAHHHA

*000679_00034024 BHHAAHHAABAHAH-HBBBBHBHBHHABHHHAHBHHHBHHHBBBHABBAHAABABHBAAHAABHAHBAABHHBBAH-HAHHABHABHBBHAHHABHHBHHHBHHBAHAHBHBHHHHHAHAHBBHAHAHHHBBBHBABBABHABB

*000165_00430006 HAHBAAHHHAAABHBAHHHBBHHBBAHHHABBAHHBAHABAHBABBHAHBHHBAA-HABHBHBHHAAHAAA-AHABHHABHAAHBH-HHHAHABHHHAHHHABHHHBHBBHAHBAAABAHHAHBBBHHBHHHAHBHHAHABBAB

*000012_00876561 BAHBABHAHABABAHBBHHAAHBHAAHBHAHBHBBAAHHBHBAHBHBBHABABHAHBHBBBHBAABAHBHBAHHBHAHABH-AHHHABBBBH-AHHHHHHHBHAHBBBHHHHHHBABHBBABBAHBAHHBHHHAH-HHHAAHHB

*001408_00146209 AHHBBA-BHBBHHHAAHBAHBBHHAHHAHAAHHAHBHHAHHHAAHHHHAAAHABAHBHAHHAAHHHAA-BBHBHBB-BHABHABAAHAHBHHHBABAHBAHABBBBAHHAHHHHABAHABBBBBBBHABHBHHBHABHHHHBHH

*004656_00030687 ABHHHAAHHHA-AHABBBHHHBABHBBHABHHHHHBHHAABHBHBHHBAAAHHBHAHBBBHAHHHHHH-HAHBHHA-HAAHABA-BHAHHBBHAHBAHBHABBHHABHAAHAABAAAAAAHAHHBHHAABHAHHBAAHBBHABA

*000030_00872604 HAHBAHBAA-HHHHAHH-ABBHBABAABHAHAHBBHAAHHBHAAAAAAHHH-BBHHBHHHAHBHAABH-ABAAABH-AHAHBHABHH-BAAAABHHAAHBHAHHHH-HHHAHHBH-HHHABABB-BHAHH-ABHA-BABH-AHH

*000166_00479364 BAHBHBHAHABA-AABBHBAABHHA-ABHAHHHBHAAHHBB-AHHHBBH-BABBAABHHBH-BAABAHBHHAHBBH-HABHAAH-HAHHBBHA-HHAHHHHB-AHBBBHHHHHHHAB--BABBABBAHHBHHHA-AHHHAAHHB

*004793_00050454 AHHBAHBBAHB--BA-BHBHBHBBABBBBHAB-AHHHHABBBBBHBHH-AAAHHHHHBAHB-AABHBB-HAAHAAH-HHHBHHB-AHHHBHAA-BHBHBBHHHHB-HHBHBBBHBBHAB-HHHBBBAAHBBHHBHAHBHBHHHH

*000499_00081848 HHHHABAHAHHABHHABBBBBHHHHBHAAHHABHBHBABBAHBAHHBHAHHABAHHHABAAHHBHBBB-BAABHHHAHAHHBBHHHHBHAHAA-BHBAHHBBBAAB-HHHHHBAHBABBABHHBBHHBAAAHHAAHB-BHHHHA

*000776_00063208 AHAHABBHABH-HAAHHBBBHHABHABBHAHAHHAAHBBBBHHBBBHHAHAHHHHBAA-AAHHHHBABBHBAAHAHHAHHHB-HAHHHBHHHHAAAABHHABHHAAHAHHHAAHAH-ABBABAHBAAAHHAHBBHAB-AAHHHA

*003544_00075785 HBBBHHAABBH-HHAHBBABHHHHB-HHAABHHHBHHHHBAAHAHBBHBABHBBHBHABBHHAAHBBBBB-ABBAA-BBHA-HHAHAHHHHHAAAHBHAHHABHAAHHAHBHBHAAAHHHBHHHBHBBABAHHAHHBAHBABHB

*000873_00237177 HAH-AA-HBAAAHHHAHAHBB-HBB-HHHABBAHHBAA-HAH-ABBHAHBBHB-A-H-BABABBHAAHAHA--AAH-HABH-AH-HBAH-AAABH-H-HA-ABHH--HHBHHHHAA-BAHHAA---HHHH-HAHAHH-H-BBA-

*001277_00039986 HHHHAHAHABABHB-ABAHBH-HHHAHBHAHAHABHH-BH-HAHHAHHAAABAABABBB-AHHHBABB--BAHBAH-HHAH--HBAABB-AHHAHHBBHHABHHHHB-BAAAHAH-A-HA-HBHHBAB--HHB-A-H-AHAHHA

*000024_00181289 B-HAHBBAABHH-HAAAHHAHH-AABAAAHHHBBHHHBBBAAHH-HHBHHBHHAABHB-AHAAAHHHA-H-HHHBB-HBBB--H--HA-HHAABAHABHABHHBHH-HAHHBBBABBHHBBHHBBHAABBHBABHBHAHBHABB

*000261_00336863 B-HAAHHAHBAAAA-HBBBAHBHBHBAHHHHAA-BHABBHHBBHBHBHAHHHBABHHAABAHHHHHA--HHHBAAHABH-AABBBHHBBAABH--BHAABHHHABA-A-HHBHHH-HABHHHHHHHAH--BHBABAB-ABHABB

*000136_00594255 AHHBABBBAHB-BHAABHBHBHBAAB-BBAHBAAHHH-AB-B-HAHHHAAAAHHH-BBAHBHAABHBBBHAAHAAHAHHHB--BHAHHHHHAA-AHBHH-HHHHHBHHBABHBHH--AHAHHBBBBAA-BBHH-HAHHHHAAHA

*000020_00629203 H-BBHHAABBH-AHBABBAHHHAHHAHHAABHHBBHHHHBAABAHBBHBHBHBHHBHHBBHHAAHBBBBBAABHAHABBHAABH-HAHHHHHHAAHBHAHHABHAA-AABBHBHAAAH-BBHHH-HBHHBAHHAHHBBHBAH-B

*001150_00095302 BHABHHABABBABHHHBAAHAHBHBHAH-BHAAHBABB-HAHAHHBHAABBHABH-BHBAAHHBBHABAHAAHABB-BAHBHBB-BHHHHBHAHHHBHAAHBHBBB-HAHHAHHHBAHHHHAHABAHHBHBAHABHBHHBHHHA

*002138_00131890 BBHABAAHAABHHHBBBAAHBBBHBAAHBHBB--HBAHBHBHABHBHAHAAABAABHHAABBBABHAHBHAHABBHAHHHAAHA-HHAHHAHHBAHBAAHHBBHBH-H-HHBBHHAHHAAHHHB-HHHH-BHBAHAHHHBHBHA

*000053_00355763 BBBHHBHAHHBHBHHBBHHHAHABAAAHAAHAABBAAHHHABHBBBABBAAAABHHBHHBAHBHAHBBBHHAHABAHHAHHAAHBHHHBBBHAAHHHABHHHHAHHHBHBHHAAHAHABHABBAHHAAABHBHABAAAHAHHHH

*000218_00077145 BBBHHBHAAHBABHHBBBHAAHHBAAAHAAHHABBAAHHHHBABBBABBAAAABHHBHHHHHBHABAHBHHAHABAHHABHAAHBBHHHBBHAAHHAABHHBHAHHHBHBHHAHHAHABBABBAHHAHABHBHAHAAHHAHHHH

*000617_00301444 AHBBHHAABBHBBHHHBBAHHBAHHHHBAABHHHBHHHHBAABAHBBHBHBHBHHBHHBBHBAAHBBBBBAABHAHABBHAABHAHAHHHHHHAAHBBAHHABHAAHAABBHBBAAHHHBBHHHBHHHABAHHABHBHBHAHHB

*000618_00218252 AHABHABHAHHBBAHHBHBBAHBAABABHAHBHBHHBBHAHAAHAHHAABHHHHHHHAHHBHHHAHBAAHHABABAAAAHAHBHHBAHHBBAHHAHAABAHHABHAHHHAHHHAHHBHABHAHAABABABHHAHAAHABHAAHA

*000661_00211247 BAAHHHAHHABHHAHAAAAABAHHHAABHHHABBBBAHBBHABAHAABHHHBAHABHBHHABHBABHHBBBHBHHHAAHAHBBHAAHBBBHBHAHHHABHBHHAHBAHHABHHAHBAHHBAAHBAAHAHBHHBAHBHBHHHHBB

*000704_00077408 BAHAAHAAAAHABHHBBHAHHHBBHABAHAAABHHBHHBHHHHHBBBAAHHHBHAABHBHABHHHHBHHABAAAHBHBBAHBHBHHBAABHHBHHHAHHBBHAHBAABABABHAHAAHHAAHBHHAHABHBBHHAHHHAABAHB

*000704_00235399 BAHAAHAAAAHABHHBBHAHHHBBHABAHAAABHHBHHBHHHHHBBBAAHHHBHAABHBHABHHHHBHHABAAAHBHBBAHBHBHHBAABHHBHHHAHHBBHAHBAABABABHAHAAHHAAHBHHAHABHBBHHAHHHAABAHB

*000800_00143325 AHHHHBHAAHBHHHHHBBBBBHABBHBAAHHHAHAHBBBBBBBBBBBBBHHHBBBHHHHHHBAHHAHHHBHHHAHHABAAABBBHBHABHHHHBHBBHBHHBHHAAHBBHHBAHABHBBBBBHHBBBBABABBBHAHAHHHHHA

*000813_00205963 HBBABHBHHAHHAAHHHBABHAHHBHBAAHBABHAAAAHAAHBHHAABBHAAABAHBHHBAABHHAAHHAHHBHHABHHBBHAHHBBABBHHAHHAAHHHHHHHHAABHBBAAHBABHHHAHBHHHHAABHBAABHBAHABHBH

*001435_00117204 HBBHBHBHBAHHHAHHHHABHHAHHHBAAABAHHAAAABHABHBHBABBHAAABHBBBHBAHBHHAHHHABHBHBABHHBBAAHBBBABBHHHHHHHAHHHHHAHAABHBHAAHHABHHHABBHHHHAHBHBAABAHAHABBAB

*001461_00021616 HAAAHHHHBBAHABABHBABHBHHBHHAAHHHAAAHBHHBBHHHHAAHHHAAHBBBAHAAHHBHHABBAHHBAAAHHAAABABHBHAHBAHHHHHAAABBAAHAAAAAABHABAAAHHHBBHAHHBHBHHAHAHHHHBHAAHHH

*001461_00154742 HAAAHHHHBBAHABABHBABHHHHBHHAAHAHAAAHBHHBBHHHHAAHHHAAHBBBAHAAHHBHHABBAHHBAAAHHAAABABHBHAHBAHHHHHAAABBAAHAAAAAABHABAAAHHHBBHAHHBHBHHAHAHHHHBHAAHHH

*001505_00086863 BAHAAHAAAAHABHHBBHAHHHBBHABAHAAABHHBHHBHHHHHBBBAAHHHBHAABHBHABHHHHBHHABAAAHBHBBHHBHBHHBAABHHBHHHAHHBBHAHBAABABABHAHAAAHAAHBHHAHABHBBHHAHHHAABAHB

*001505_00090689 HAHAAHAAAAHABHHBBHAHHHBBHABAHAAABHHBHHBHHHHHBBBAAHHHBHAABHBHABHHHHBHHABAAAHBHBBAHBHBHHBAABHHBHHHAHHBBHAHBAABABABHAHAHAHAAHBHHAHABHBBHHAHHHAABAHB

*002386_00037838 HBBHBHBHBAHHAAHHHHABHAHHHHBAAABABHAAAABHABBHHBABBHAAABABBHHBAHBHHAAHHABHBHHABHHBBHAHHBBABBHHHHHAHHHHHHHHHAABHBBAAHBABHHAAHBHHHHAABHBAABABAHABHHH

*005900_00024588 BBHABAHHHAHABHBBBAABBBBHBAAHBBBBBHHBABBHHHAHHHHAHAAAHAABHHAABBBAHHHABBHHHBBHHHAAAAAABBAAHHAHHBHHHAAHHBBHAAHHAHHHBHHAHHABHHHBABHAHHAHBAHAHHHBABHH

*000192_00518897 AAHHAHBBAHHHBHAHHHABHHBHHHAHAABAHHBAAAHHBHAAHHBABHHBABAABHBHAABHAABAAHBAAHHHBHBAHBHABHHAAAHHHHHBAAHHHAHAABHAHHAABBHHBAHABABH-BHAHAAHHHHHBABHHABB

*000370_00366704 AHAA-HBHHHA-AB-AHHH-B-AAHBA-AHBHHH-AH-BHAHHHA-HBBHBHHHABHBBHHAHAHBHH--H-AABB-BB-HH-BAHHAAHHAAAAAA-AABABHHHBHHH-BBAA--B-BB-HBBHAABHAHAHHBHHH-HAB-

*000970_00214048 -HHHABAH-BHABH-ABHBBBHHHHBHAAHHABHBHBAHBHHBAHH-H-HHABABH-HBAAHHBHBBBHB-ABB-A-B-HHB-HHHABBAHAABBHBAH--BBAAHBHHHHHBABBABB-BHHB-HHBAHAAH-AHBHBBHHHH

*002008_00069183 AHHHABAH-AH-BH-HHBBBBHHH--HAAHHABHBBBAHH-H-HHHBHAHHABABAHHBA-HHBABBB-B-AHHBAH-AHHBBABHHBAAHAH-BHBAHBBBHAHHBH-HHHBBA-ABBABHHBBHHHA-HHHAABBAB-BHHA

*000045_00835783 HHHBBAAHAAHABHHAHB-HAABHAB-AHBBHHHBAHBAHABAHHHHHHHAHBAHBHHHHHABBHBHB-HBAAHHBBHBHA-BHAHAHABABHHBBH-BHHBAHHH-AHHAABHAA-BHABBHA-AAHBHHBABAABAHHAAHH

*000177_00470271 HAABAHHH-AHBAHAHABABBAA-B-HHBHAAHBAHH-BBAH-HAAHBHAHBABABHHHHABHAHHAHHHBHBAHH-ABABHBB-BHHA-BBHBABHHBABABABH-BBAHHAAHAHHAHAHHBHHAHHBBAHAHHAHHHHHHB

*001779_00045422 ABHBHHBHBAABABHHBHHAAAHAAAABBBHHH-HHAAHHBHABABHBHBBAHHH-AHHBH-BAAHHHAAHAAHBHBAHHB-AHAAH-HABHBAABHBBAHBHAHBBHHHAHHHH-BHHAHHAH-HAHHH-ABBBBA-AAHAA-

*001959_00022704 AHBBHHAHBBH-AHHHBBAAHHAAHHAHAAHHHHBHBHHBAA-AABBBAHBHBHH-HHBBHHAAHBBBABAABAAH-BBHHA-H-HAHAHAHHAABHHAHHABHAAHHABBHBAAAHBHBHHHH-BAH-BAHHA-BBHABAHHH

*000034_00112945 ABHHABHHA-HHBH-ABABBBBAHHBHAAHHABHBHBAHBHHBHHHBHAHHABAHHHHBAAHHBHBBB-B-ABHHA-HABHBBH-HHBAAHAHBBHBAHBBBAAAH-HHHHHBAA--BBABHHBBHHBAHAHHAAHB-BBHHHA

*000018_00743732 HHHHHBBBBBHBHAHBABHABHABHBBHHHBBBAHABBHBBBBHBHBHHHBBAHHHHHAHBHBHBHBBHHHABABHAAHHHBBH-AHBABHHHHBBHABBBHABHBHBBHHBBHHBBBBBBHHBBHBBHHHBHABBHBAHHBHH

*000512_00092462 BHABABBBABAAHAHAHABBHABABAHAHHHHBABABAAHHHHAAAHHBHBAHBBBHBHAHHBHHHHBHHAABHABAHAAHAHHBHBAHHHHBAHAHHHHHAHHBH-BAAAHAAHHHABHHHHHBBAHBHBABBAHHAHBBBHA

*000588_00214315 BHABHHABHHBABHHHBAAHABBHBHAHHBHAABBABBHHAAAHHBHAABBHABHHBHBAAHHBHHABAHAAHABHABAHBHBBABHHHHBHAHHABHAAHBABB-BHAHHAHHHBHHBHHHBABAHHBBBAHHBHBHHBHHHA

*001047_00348687 AHHHABAHAHHHBHBABHBBBHHHHBHAAHHABHBHBAHBHHBBHHBHAHHABAHHHHBAAHHBHBBBBBAABHHA-HAHABBABAHBAAHAHBBHBAHBBBAAAH-HHHHHBAABHBBABHHBBHHBAHAHHAAABHBHHHHH

*001959_00074378 AHBBHHAABBHBHHBHBBAAHHAAHHHHAAHHHHBHBHHBAAHAABBBAHBHBHHBHHBBHHAAHBBBABAABAAHABBHHABHHHAHAHAHHAAHHHAHHABHAABHABBHBAAAABHHHHHHBBAHHBAHHAH-B-ABAHHH

*000054_00719724 BAHB-HAHBHHHBB-HBBHHHA-BBBHHHABAA--AAHAHABHA-BAAAAHBHBHAHBB-H-ABH-HH--BHBHH--BA----BHHAB-HBBHBHBAAHAHAABBHAHHBHHBH--A-HHBHHAHHHHHABA--AAH-AHABB-

*001100_00097051 HBHBHAHB-BBB-HBHHAAHAHBAAHBHHHHHH--HHABB---HAHHHHBABH-A-BBAHHBABBAA-AHB-BABB-AHH-A-BAHA-HBHBHAHAHBAABBHHHB-AHHBAHAHH--H-HHBHAHAAB--HH-B-H-AH-ABB

*000036_00659554 HHHBAHBB-HBHAH-H-A-HBHBHABBBBHHBHAHHA-AB-BB-HBHHAHAAHHABBBAHBHAHBHBH-AAABAAAAHHHBH-B-ABBHAHAA-HHBAHHHH-HHBHHBA-HBHH-BAHAHH-BBBAA-BHAHBHAB-HHHAHA

*010603_00005197 -BHBBHHHHHA--B-HBHHHABHBH-HHBHHAH-HHBBHBABHHBABAABAHABH-BHBHABHHHBHAAHHABBAA-A--BH-B-HHAAHBHB-HBAHBHHB-AAA-BHHBAHHAABHBAHABHHHAB-AAHA-HHBAAH-AB-

*000107_00092773 BHHABBAHAAH-BHAABHAHHHHHH-HBAHAHHHHABBABABAHHAAAHHHHHBAHBAAHHBAHHHAHBBBHBBBHBHBBH-BHBBHAHHABBB-HAHBAHHAHHBHHBBHABAABA-BHB-BHAAHA---BBAH-AHAHHHAH

*000835_00004304 HHBAHAAHABH-BABHAHABAHHHHHHAHHABHHAABHBHHBABHAABAHAAHHAAHBAHBABBBHHBHB-HHHHBBBBBHHBH--HHAHAHB-AHBHBAAHABH-HAHBBAHBA-HBAAHHHABABH-BHAHAABH-AHHHH-

*002268_00142323 BHHHBHAHBAHABBHBHBHHH-ABB-HHBABAA-HAHHAHABAABBAAAABBHHHAABBAAHHBHBHHHBBHBHHHABAAHH-BBHABAHHBHBHBAAHAHHABBH-HABAHAHH-HHHHBBH--BHHHABHBHA-H-AHABBH

*001321_00007076 AHHBAHBBABBHBHABBHBHB-BHABB-BHHBAAHHHHABAB-HABBHAHHAHHAHB-AHBHAHBHBH-HAABAAA-HHHBHHB-AHBHHHAAHHHBAHHHHHHHB-HBABHBHHBBA-AHBBBBBAAABH-HBAAB-HHHAHH

*002024_00075707 ABHBHHBHBAA-HH-BBHHAHAAAA-AHBBBBABHHAAHHBA-BAHABHBBAHHHAAHHBBBBAAHHH--HAAHBHBHBHBHABBAHHAHBAH-HBHBBAHBAAHB-HHHAHHHHAHHHAHHAH-BHH--HABHBHABA-HAAB

*000093_00087932 HHHAHHAAAHBABAHHHHHHHAHBH-BAHAAHHHBBAAHBBHABBBBAAHAHHHAABBHBHHHBAHBABHHAAHHH-BHAHAHBHHBAAHBBBHAHABBHBHHBBHAHAHHBHABHAAHHHBBHBHAAHBBHBAAAH-AABHHB

*002841_00124080 BBAHBHBHHABAHAABHBAAABABAABHHHBHABABHHAAHHHBBBBAAABHBAHAHAHHHBHBHHHHAHHAHHHBAH-AB-BAHAHH-BAHBBAAHHAHHHABAHAHABHHHAH-BHHHHAHAAHBBHBABHAAHHHAAHHBB

*000197_00758378 ABHHAHBBA-HBBHAHHHABH-BH--AHAABAHHBAAAHHBHAAHHBABHHBHBAABHBHAHBHAABAAHBAAHHHBHHAHBAA-HHAAAHHHHHBAHHHHABAABHAHHAABBHHBHHABABBHBHAHAAHHHHHBABHHABB

*003929_00009015 ABHBBAABHBBHHH-AHBAHBBHHAHHAHAAHHAHBHAAHAHAAHHAHAAAHABAHBHAHHAAHHHAABBBHBHBBHBHABHAB-AHAHBHHHBABAHBAHABBBH-HHAHBHHABHHABBBBBBBHA-HBHHHHBBHHHHBH-

*000299_00058502 H-BA-HBH--BBHA-AHHBHHHAHA-HHHHHHHAABHA-BAHB--HBHAHHHH---HAA--AAHBAB--AB-BHHA-BHABA-HBBABABHAH--AH-BBHHBHHA-ABHBHBHA-BHBB---A--BH--HHB-A-HBHHAB-B

*000645_00114625 BBAB-BBBHBA-BABHAAHBBH-ABAHHAHHH-A-ABHAHHHHA-AHHHHBAHBBBHBAAH-HHHHHB--AAHHA--H-AHB-HHHB--HAHBAHAHHABHAHBBAHBAAHBAA--BABHHHABHBAHHBBA-BAHBHHH-BH-

*000112_00616694 HHHHBBBHH-HBBHA-HHBAABABH-ABBHBAA--HHHAHHBHBBAHAABHHAABBAAAHAHBHHHAAHBAHHHH-BBHHH--B-BHB-HBAHBBBHHHBBHAABAAHHHBHHAB-HHBHAHAAHAHA-BBABAHHHBBHHBHB

*000665_00003900 AHBHHA-HBBB-HHABHHAABHHAHAAHAH-HBHHBBAABAAAHABHHAHBBHHHBHHHBHHHAHABBAH-HBAAHABBBHABHAHHAAHHHHBHHAHHBHA-HA--BABBHBA-AABBHHBHB-HHH---ABABBBHHB-AAH

*000078_00799721 BHBAAAAAABAHAHHHBBHBHBHHHAABHHHAHBHHHBHHHH-BHABBAHAHBABBBAAHAABHAHBA-BHABBAHHHHHHABHABHBBHAAHABHHBBBHHAHBA-AHBHBHHHH-AAAHBBHAHHHHHBBBHBAHBABHAH-

*000502_00043403 BHHBAHBBHBAAHAHHHHHAAABAB-HABHAHAABHHHAHHBBAAAHHBABAHHBBAHHHHHHHHHAHHAAABHAHAHAAABAABHHAHHHHBABABHHABA-HH-ABAABHAAHBBAHHHBAABBHAHHHAH-H-HBBAHBHA

*000705_00348103 HHABHHHA-HHBBHAABBHBBHHBBBAHAAHHABBAHHAHAAAABBHHHHHAHBBBHAHHHHBAABBABAH-HBHAAHHHABAHAHAHHHHAAAABBHAHHBBHAAHHAHBHBHAA-BHHHAHB-HBHABABHHHHHBABABH-

*001493_00254512 HAABAHHHAAHBAHAHHBAABAAHA-AHHHAAHBHHHBHHHAHAHAHBHHHHABABHHHBABHHHHHBBAHHBHHA-AHAHB-B-AAHHBBBHAAHHABHBABABHBHBAHBAAHAB-HBAHHBAAAHABBHBAH-HBHHAHBB

*002755_00027960 AHHHA-BHAAHABAAAHHBBAHH-AHBBBAAAHHAAHHHBHBHBBBHHAHAHHBHBAAAAAHHHHHHBBBBAHHAHAHHAHHBHAHHHHB-HHAAAABAHABAB--AHBHHAHHHA-AHBABAAHAHAHBBHBBHBBAAHHHHA

*001565_00005812 ABHHAHBBAHH-BH-HHHABHHBHHHABAABAHHBAAABHBHAAHHBABHHBHBAABHBHAHBHAABAAHBAAHHHBHBAHBBABHHAAAHAHHHBAHHHHA-AAB-AHH-ABBHHHHAABABHHBHAH-AHHHHHBAB-HABB

*000389_00053190 AABHABHHABHHAHABHBHHBHBHAHAHHBBHAAHABHHAHBBAHHBAHAAABHHHHAAHHBBBAAAHBBHBAHHBABHBHHHBBAHHABHBBAHBABBBBHAHHHABHHABAHHHBHBBHAHBBHAHBBHBHHBBHAHHHABH

*001778_00087017 AABHABHHABHHAHHBHBHHBHBHAHAHHBBHAAHABHHAHBBAHHBAHAAABHHHHAAHHBBBAAAHBBHBAHHBABHBHHHBBAHHABHBBAHBABBBBHAHHHABHHABAHHHHABBHAHBBAAHBBHBHHBBHAHHHABH

*001778_00129902 AABHABHHABHHAHHBHBHHBHBHAHAHHBBHAAHABHHAHBBAHHBAHAAABHHHHAAHHBBBAAAHBBHBAHHBABHBHHHBBAHHABHBBAHBABBBBHAHHHABHHABAHHHHABBHAHBBAAHBBHBHHBBHAHHHABH

*001349_00180643 HHBH-H-AB-B--HBAHH-HAAHAH-H-HBBHABBAHHAH-B-HHHHHH-HHB-B-HHH-HAB-HBHA-B-AAHBBHABAAABHAHAHAAABB-BBHABH-BAH---A-HHABHH-------HAHAA-BA--HBA-B-HHAHAB

*000721_00216736 BBAHBHBH-HB-HHABHBHHABHBAABHHA-H-BABH--AHBHBBBBAAABBBAHAAAHHHBHBHHHH-HHABHHH-HBABHBH-AH-HBABB-HAHHHAHHABAA-HABHHHHBHBHHHBAA-ABBBH-ABBAAHHBA-HHBB

*000558_00159396 BHHBAHHHHBBAHABHHBBAH-ABH-BAHBBBHHHABH-HHB-BBHBAAABAABHAHHHHBHHHHAB-HHAAHABH-HBABH-HAAHHBBABBBHABHAHBAABAAAHAHHHAHHBHHBBBHHABHBBHHAHHAHHBAAAHBHB

*000112_00360198 AHHHBBBHHAHHBHHBHHHAABHBHBABBHBAAHHHHAAHHB-BBA-AABHHAABBAAAHAHBHHHAHABAHBHHA-BHHHBHBHBHAHHAAH-BBHHHBBHAABAAHHHBHHABABBBAAHAA-AHAAHBHBAH-H-BHHBH-

*000112_00683691 AHHHBBBHHAHBBH-BHHAAABHBH-ABBHBAAHAHHHAHHBBBBAHAABHHAABBAAAHAHBHHHAHHBAHHHBA-BHAHBHB-BH-HHHAHBBBHHHBBHAABA-HHBBHHABABHBHH-AAHAHAAHBHBAHHHBBHABH-

*000352_00000126 HAHBH-BHHAHBBH-HAHAAHBBBB-HHBAABB-BBHHAHBBBHBHBHBABBBHBAHHHBBBBHABAHBHBAAHBBHBHBBHH-AHHHABHHBA-AH-HAHBHBH-AHHABAHHHABABBHBHHHHBHBBHHAHBBHAHHHAHB

*001975_00089670 BAHBAHHBA-AHAHHHBHABABBAB-HAHHHAABHABABHB-HAHHBHABHHAHHHBABAAHHHHHHBAAAAHHBHABAHBAAB-BHHBBAHABHABBHAHB-BBH-HHAHHHBABHB-HHBBABHBHAHBHBHH-HAHHHBAA

*004115_00013549 BBABBHBHH-B-HAHBHBAAABABAABHHHBAABABHHAA-HBBBBBAAABHBAHAHAHHHBHBHHHHHHHAHHHHAHBABH-H-AHHHBAHBBAAHHAABHABAH-HABHHHHHBBHHBHAHA-HBHA-ABHAAHHAAAHHBB

*000970_00082796 AAHHABAHAHHABHAABHBBBH-HHBHAAHHAB-BHBAHBHHBAHHBHAHHABAHHHHBAA-HBHBBBABAABHHA-HABH--HH-H-AAHAABBHBAHHBBBAAHHHHHHHBAH-ABBABHHBBHHBAHAHHAAHBBBAHHHB

*000018_01043174 BHHHHBBBBBHBHAHBABHABHABHBBHHHBBBAHABBHBBBBHBHBHHHBBAHHHHHAHBHBHBHBBHHHABABHAAHHHBBHAAHBABHHHHBBHABBBHABABHBBHHBBHHBHBBBBHHBBHBBHHABHAHBHBAHHBHH

*000918_00052581 HHBBBAHAHHAHHBBHBHBHHBBHAHBHHHBHBHBHAHHAAHBHHHBHBHBBHHABHBHAHHHBBABABAAHHBABBHHBHABHHAHBHHAABAHHABBAAHAHBHBHAAAHHBABHAAHABHHHBHHBBBBBAAAHBAHHHBB

*001477_00001373 BHHABBAHAAHHBHAABBHHAHHBHAHBAHAHHHHABBHBHBAHBAAAHHBHHBAHBAAHHBHHBAAHBHBHBBBHBHBHBABHBBHHHHAHBBHHAAHAHHAHABHHBBBABHABAHBHBHBHAAHAHAABBAHBABAHHHHH

*010955_00001385 HHHHAAABAHHBAHHBBABHHHHHAHHABHBAHABHHABABAAHHHHHAAHAAABHBBHHABBHHABBHHBHAHAHAAABAAAHHAHBHBHHHAHABBAAHBHHHHBBBAAHAAHHAHAAAABHHHBBHHHHABHBHAAAHHBA

*000200_00456281 BHHAAHAAABAAAHHHBBHBHBHHHHABHHHAHBHHHBHHHHBBHABBAHAHBABHBAAAAABHAHBAHBHABBAHHHHHHABHABHBBHAHHABHHBBHHHAHBAAAHBHBHHHHAAHABBBBAHHHHABBBHBABBABHAHB

*000337_00052242 HBBHBHBHBAHAHAHHHHABHAHHHHBAAABABHAAAABHABBHHBABBHAAABABBHHBAHBHHAHHHABHBHHA-HHBBHAHBBBABBHHHHHAHHHHHHHHHAABHBBAAHBABHHAAHBHHHHAABHBAABABAHHBHAA

*000267_00162082 ABHAHHABBHHBAHHAHABAABHHHBHBBBHHHBHAAAHAAAHHHBHBBAHHHABHHAHHHBHHBAHHAB-HBABHBHHABHHAAHBHHAAHBBBBHABBAHHHBBBHAAHBBHABAHHAHHHAHAABH-BHHAHBHABBBABB

*003084_00022507 HHHHAHBBA-HHBHAHHHABHHBHHHAHAABAHHBAAAHHBHAAHHBABHHBHBAABHBHAHBHAABAAHBAAHHH-HBAHBAABHHAAAHHHHHBAHHHHAHAABBAHHAABBHHBABABABHBBHABAAHHHHHBABHHABB

*007093_00009966 HHHBBAAHABB-BAAAHHAHBHHBAHAHHHABHAHBAAABBAAABHHAAAAHHBA-BHAHBHAHHHAHBBBHBHBBABBHBHABHHHAHHBHHBAHAHBAHHBBBBHHHAHBHHHBAHHBBBBBBHHABHBAHHHHBHHBHHBH

*000047_00699891 BAHAHHAAAHBABAHHHHHHHHHHHABAHAAHB-HBAAHBBHABHBBAAHAHHHAABBHBHHHBHHBHAAHAABHH-BHAAAHBHHBAAHBBBBAHABBHBBHBBH-HAHHBHABHAAHHHBBHBHAAHBBHBHAAHHAABAHB

*001794_00028638 BBHHBHHHHABAHA-BHBAAABABAABHHHBHHBABBHAAHHHBBHBAAABH-HHAHAHHHHHBHAHHAHHAHHHH-HBABHBHHABBHBAHBBAAHAAHBHABAAAHABHHAABHBBBHBAHAAHBBAHABHAAHHHAAHHHB

*001541_00037002 AB-HABAAAHHH-HBABHBBBAHHHBHAABHABHBHB-HBHH-BHHBHAHHABABHHHBAAHHBABBB-BAABHHA-A-HH-BHBHHBHAHAHBBHBAHBBBHAAH-HHHBHBAAB-BBA-HHBBHHBAHAHHAAHBHBHHHHB

*000011_00377891 AHHBABBHAHA--AHHBBHBHBHBH-BBBAHABHAHBHHH-HBBBHHH-HAHBBHBAHHAAAHBHHBB-BBAAHAHAABABABH-HHAHHHHAAA-ABA--BABBH-H-HHHHHH-HABBH-AHHAHA-HBAB-A-BBHHHBHA

*000336_00286748 BHBABAHHB-BBHHBH--AHAAHAABH--HAHHA-AHHHH-ABBAAHHABHAAHHAHBH-HABABBHBBH-AAHABAB-HHAABAAH-HHAHH-ABBAB-HBHBHH-H-BHAAHHHB--HB-HA-BHAB-AHH-HAH-AHAHB-

*000040_00875349 AHHAHHAAB-A--B-AHBBHABBA-BAABAHBAAAHAHBH-HHAHHHBAAHHBBHHABA-BBBHHHH--H-AHBH-BH-HBABHBAHAAH-AH-ABAABHHAHHHA-H-HHABBBH-H-BHHAA-H-HB-BHB-H-H-BHHAAH

*000088_00308293 BHBAAHHAABAHAHBHBBBBHBHAHBABHHHAHBHHHBBHHBBBHABBAHHABA-HHAAHAABHHHBABB-HBBAH-HHHHABBHBHBBHAHHABHAHHHHHBHBAHABBHBAHHHBAHBBBHH-HAHHHBBBHBABBAHHABB

*000930_00176887 BHHBAHBBHBAA-AHHHHHAA-BABBHABHAHHABHHHAHABBAAAHHBABAHHBBAHHHHHHHAHAHAAAABHABAHAAAHAABHBAHHHHB-BABHHAHAHHHHHBAAAHAABBHAAHHHAABBHABHHAHBHHH-HHBBBA

*003507_00057348 BHAABBHBBBAHHBABHHABAHBBHBHHHHHHAHAHHBHBBHBAHAAAHHABHHBHHHAAHABAHABH-HBBHAAH-HHBAAHHHHABBAAHHHHAHAHBHHAAAAAHAAAAHABABHHBHBHAHBABHBHHHBA-H-BBABBH

*005742_00020694 BHABABBBHBAABAHAAAHBHABABAHHAHHHBAHABAAHHHHAAAHHBHBAHBBBHBHAHHHHHHHBHHAABHA-AHAAH-HHHHBAAHAHBAHAHHAHHAHBBA-BAAHHAAH-HABHHHHAHBAHBBBABBAHHHHBBBHA

*000148_00100142 AABHABAHABH-AHHBHHHHBHBHAHHAHHBHHABABAHAHBAABABHHAAABHHHHAAHHBBBAAAHBBBBAHH--HH-HHHHBABH-BHBBAHHABBBBHAHHHHBBAABAHH-AABBHAHBBAAHBHHHHHHBHAHABHBA

*000051_00143542 BHHB-HBBHBA-HAHHHHHAAABABBHABHAH--BBHHAHBBBA-AHHBABAHHBBAHHHHBBHBHAHBAAHBHAB-HAAA-AABHHA-HHHBABABHHABAHHHHABAABHAAH-HAHBHHAABBHAHHHABBH-H-HAHHHA

*001082_00195766 HHABBHHHH-H-BAAHBHHHAHHBB-AABHAHHAHAHABHHHABBHBAAAHHBBHHHHAAHHBHHAB-HAHHBHHH-HAHHBAH-BAAAABHA-HABHBHAH-ABBBHHAHAHBAB-BHABBHA-AABHABAABBHH-HHAABB

*002387_00043331 AHBBHHAA--HBHHAHBBAAHHHAH-HAAABBHBBHHBHB-AHAHBBHBABHBHH-HHBBHHAA-BBB-BAABBAAABBHAHBHAHAHHHHHH-AHBHAHHABHAA-AABBHBHAAAHHHBHHH-BHHABAAH-HHB-HBHBHB

*001374_00110964 BBHABBAHAAHHHHAAHHHHHH-HH-HBAHAB---ABBHBABAHBHAAHHHHHHAHBA--BB-HHBAHBBBHBBB--HBBB-BHBBB--H-BBBHHAHBAHBAHHBBHBBHHBAA--HBHBBB-AHH-HAHBB-HBAHABHHHB

*000752_00366829 H-H-AAAHHAA--B-AB-HBAHHHH-HBHAHAHABHH-BHBH-HHAHHAAAB-AH-BBBAA--HHABB--BA-BAH-HHAHABHAAA--BAHHAAHBBHHAB-AHH-BBAAHHA-HABHAHHHH-BABHBAHH--BH-AHAHHA

*000008_00351987 BHHBBH-ABHHA-BHBHBHHHAABBHH-BA-AA-HAH--H-B-ABBAAAABBHHH-BBBAAHBBHBHH-BBHBHHH-BHAB-ABBHABAHHBH--BAAHAHHABBH-HABHHHHHB--HHBA-H-AHHH--HBHA-BH-HABB-

*000720_00263095 -HA-HABAAAABAAHHHH-H-AHA-ABHAAAAHHHHHA-HHHBAAHHHAHHHA-B-HHAAH-HAHAHAHA-A-BAABAB-AHHH--AHBHAAH-ABHAA-BAAHAB-HHHAHAHAB--BAAHB-AHHA---HA-H-H-BBAH-B

*000352_00350840 HHABHBBAHAH-BH-AHBHAHHH---AHHAABBBBBAHAHBB-HBHHH-ABHBB--H-HBBBBHA-AB-B-A-HBB-BH-B-AHAAHHAHHHH-AAHHAAHB-BBAAHHA-AHH-A-A-BH-H--HBH--HHA---H-HBHAHB

*000862_00256121 HAHBAABAHHHAHBHAHBHBBBBHBBHHHHHBBBBHAAHBAAABHAAHHAAHBBHBBBHHBHHHAABHHABHAAHBHABBBBHAHAHBBHAAHHAAHHBHHHHBBHBHAAAHAHABBBAAHHBBAHHHBHBHBAHHAHHBHAHH

*001372_00168679 AHBBHHAABBHBBHHHBHAAHHAAHHHHAAHHHHBHBHHBAAAAABHBAHBHBHHBHHBBHHAAHBBBABAABAAHABBHHABAAHAHAHAHHAAHAHAHBABHAAHBABBHBAAAABHHHHHHHBAHHBAHHAHHBHABAHHH

*001948_00093675 HAAAHHHHBBAHABABHBABHBHHBHHAAHAHAAAHBHHBBHHHHAAHHHAAHBBBAHAAHHBHHABBAHHBAAAHHAAABABHBHAHBAHHHHHAAABBAAHAAAAAABHABAAAHHHBBHAHHBHBHHAHAHHHHBHAAHHH

*002657_00076658 AABBHHAABBB-HHHBHHAABHHAHAAHAHHHHHHBBAHBHAAAABHBAHBHHBHHHHBBHHHAHABBAAAHBAAHABBHHABHAHAAAHAHHHHHAHAHBAHHAHHBABBHBAAAABHHHBHBHBAHBHAHBAHHBBABAAAH

*000310_00204843 HHABAABAABHBABAAHBHBBBHHHBAHAAHHAHBABAAB-AHHHBHHHHHHHHBHHAHHHABHABBA-HBAHBHAAHBAABAHABHBHHHHAA-BBAHBHBBHAAHHHBBHBHA-AHAHHABBHHBHAH-BBHHAH-ABHBHH

*000591_00048899 -AHABAHHBABABBBBHHBHAABAH-HBHHHHBAHHHHHAHH-BAAHHHBBAAHHHHHHAHABABHHBHHBBAAHB-B-AH-ABAAHAHHAHAAAHAABHHHBBHBAAHBHHBHHH-BAHBAHAABHAB-HHABAAHBAHBHBB

*001909_00070639 BHHBAHBBHBAAHAHHHHBAAABABBHABHAHA-BHHHAHHBBAAAHHBABAH-BBAHAHHHHHHHAHHAAABHAH-HAAAHAA-AHAHHHHBABABHHABAHHHHABAABHAAH-HAHBHHAABBHAAHHAHBA-HABHHBHA

*004466_00002993 HH-AHA-H-HA-AA-HAB-HHHAHH-ABAAHAHABBHHBAHABHBHHBAHB-BHAHBABHAHBA-HBHHAHH-BBH-BHHHABB-AHAAHHBH-BHHHBBHA-HHH-BBAHBBBAHA-AAB-B-AHHBBBHHHHBAB-ABHH-B

*000701_00098288 -BBBBBHABHA-AB-AHHH-HBHBB-BHAHAHB-HHHAHABHHBBAAH-BBAHB-HHHBHH-HH-HBBHH-BAHB--A-H-BAB-BHBBBHHHABHAHHHAAHHAA-ABHBAHBHAHA--HAHH-HHHHBABAAB-BBH-AAAB

*001115_00110612 AHAHHB-HAAH-BBAHAH-AAHBBBAHHHAAB-BBBHH-HBB-HBAHHBABBBH--HHHBBBB-A-AH-A-AAHBBBBH-B--HAHHHHAHHHAHAAAHAHBABHA-HAHBAHHABHABHB-HHBHBHB-HHAH-BH-H-HABH

*001406_00099689 B-HBAHHBH-A-AH-ABHABABBH-HHAHHHAHB-ABABH-AHAHBBHABHHA-H-BABAAHHHHHHB-A-AHABH-BAHB-BB-BHHBBAHAHHABBHABBHBBH-HHHH-ABA-HBAHHBB-BHHH--BHBBAHH-HBHBAA

*000056_00121093 HBHHHBHHAHA-BH-HBHBHHBHHA-BHHHHBAAHB-HAAAB-HHABAHAAHBAABAHAABBBBAHA-A---HBHA-AHHB---H-HBBHBAA-HHHHHHHHHAA--BHHHBABAB-H-AH-AHBAHBAA-HBHABBBHBBBHA

*007682_00000935 H-HHH-HAHBAA-HH-B-BAABHBB-BHBHAHAAHHH--HBA-ABAAB-AHBHHBHAHBH--BHHAH-AAHBBAA--H--HAB--ABAHBH-A-H-AHBHAAHHHHAH-A-AHABH----BB-ABHHB---BBAH---HHHHBA

*000018_00811576 HHHHHBBBBBH-HAHBABHABHABHBBHHHBBBAHABBHBBBBHBHBHHHBBAHHHHHAHBHBHBHBBHHHABABHAAHHHBBHAAHBABHHHHBBHABBBHABABHBBHHBBHHBBBBBBHHBBHBBHHABHABBHBHHHBHH

*000961_00063724 BHABHHABBHBABHHHBAAHAHBHBHAHHBHAAHBABBHHAAAHHBHAABBHABHHBHBAAHHBHHABAHAAHABB-BAHBBBBABHHBHBHAHHABHAAHBABBHBHAHHAHHHBABHHHHBABAHHBHBAHHBHBHHBHHHA

*000248_00242989 AHABHABHAHH--AHBBHBBBHHAABHBHHAHHBHBBBHBHAAAAHHAHBAHAHA-HBHBHAHBAHBH-HAABABA-AAHAABHHBAHHBBAAAHHAAHHHAABB--HHAHHHABHBHAHHHHH-BAHABBHHHAAH-HHAAAA

*000846_00170972 HAHBAAAHAAA-BHHAHHHBBAHBB-HHHABBAHHBABHH-H-ABBHAHBHHBA-HHHBABHBHHAAHAAABAHAHAHABHAAH-HBHHAAAABHHBAHAHHBHHH-HBBHHHHAAAHAAHAHB-BAHHA-HHAAABHHABBAB

*001337_00193731 AHBAHABAHBHAAABAA-ABAHHHHHAHHHAHHBHHBHBHBB-HHHHBAHAAHHAABBAHHHHBBHHB--AHBABBBHBHHBAH-HHHABAHHHAHH-BHAH-BAB-HBHBAHBABBBAAAHBAHAHBBBAAH-BBHHABHHHA

*001233_00160587 BAAHBHAHHHB--A-BAAABBHHHHAABHHHHBABBA-HBBA-AHA-BBHHBHAHBHBBHH-HHABAABH-ABHHBAAAHH--B-AHBBBH-BAAHHAHABBHAHA-HHA-HHAH--HHBAHH--ABH--HABAA-H-HAHHB-

*003996_00040345 --HHABAAA-H-BH-ABB-BB-HAH-HAAHHABHBH-AHBHH-HAABH-HHABAA-HHB-AHHBHBB-HB-A-HHA-H-BHB-A-HHBA-HAABBHBAHBBB-AAA-H-H-HBAA--BB-BHHB--H----HHA---HBAHHH-

*001049_00413877 -BHHBBH-BB-BHH-BBBHAHAH-A--BHBHHHBHH--BBHB-B-HHHAHH-HHH--HHHHBHA-BB---B--BBBBABBHH-B--BBH-BBBAHBBBB-BHHA-B-HBB-BBHHBHB--BB-H-H-HBHH-H-HB--AABA-B

*000007_01020448 BHABAHABHHBABHHHBHHHAHBHBHAAAHHAAHBABBBHAABAHBHHABBHABHHBABHAHHBAHABAAHAHABH-B-HBBBHABHHBBHAHHHABHAABBABBHBHHHHAHHBBBAHHHBBA-AHHHBBAHHBHBHHBHHHA

*000099_00538351 HBBABHBHHAHH-AHHHB-BHAHHBHBAHHBABHAAAAHAAHBHHAABBHAAABAHBHHBAABHHAAHHAHHHHAABHHBBAAHABBHBBHHAHHAAHHBHHHHHAABHBBAAHBABHBAAHBHAHHAABHBAABHBHH--HBH

*000726_00291562 AHABHABHAHH-BAAHBHBBHHHAABABHHHHHBHHBBHBAA-HAHHAABHHHHABHHHHHAHBAHBA-BHABABABAAHAHHHA-AHHBBAAAHHAABAHHABHHAHHAAHHABHBHABHAHHABABABHHAHAAHABHAAHA

*000019_00094657 AHABHABBA-HBBA-HBHBBHHHAABABHHHAHBHHBBHBAAAHAHHAABHHHHABHHHHHAHBAHBAHBHABABA-AAHAHHH-BAHHBBAHAHH-ABAHHABHH-HHAAHHABHBH-BHABHABABA--HAHA-HABHAAH-

*001950_00129200 HBAHHABABBHHABB-BH-HAHBAAB-B-B-HB-HHHAB--HAAABAHB-B-BH-HHBHBHBHAAHHA-AA-H-HHBABHHHAHBAHB-HBABBHHHBBHHBAHBHAHBHAHHHHB-HHHHBAAAAHAHB-BHAAAA-HABAHH

*004577_00004147 HAHABAHHBHB-HBHBHHHHA-BAH-BBHHHHBAHHHHHH-HBBAAHHHBBAAHH-BHHAHABABHBB-HBHAAHB-BBHAHABAAHABAAHABAHAABHHHBBHH-AAB-HBHH-HBA-BAHA--HA-BHHABB-H-A-B-BB

*000021_00893727 BBHH-AHABBHB-ABHBBBBHHBHHHAHHAAA-A-BHBBHBHHHBHBHHAHBBAHBHAB-ABHABBHAB-BHBAHB-BB----HHHB-BH-HB-BBBAAABHBBBAHAHHHHBH--H-ABHAHHHHABHHHBBAHBH-AHHAHB

*001155_00081506 AHAH--BH-HH-BAAHHHBBH-ABBHBBHA-AHHAAH-HHHHHBBBHHAHAHHH-BAAAAAHHHHHAB-B--HHAH-A-AB-BH--HHBBHHBAA-ABH-AHAHH--HHHBAABH-BA-BABA-BAAA-HAHBBHBBBAAHHA-

*000113_00039192 ABHHABHHHAHBBHHHHABAABHBHHHBBBHAABAHAHBAAAHHBBHABABHHAHBHBHHABHHHBBHHHBABHBHBHBHBHAAABBAHHAHBBHBHABBHHAHBHBHAAHBBBAHHHBHBBHAHAAAHABAAHABHAHHBHAH

*000115_00020015 ABHAHHABBHHHHHHAHABAABHHHBHBBBHHHBHAHHHAAAHBHBHBBAHHHAHAHAHAHBHBBAHHABHHBABBBHAABHAAHABHHAAHHABBBABBHHAHBBBHAAHBBHABHHHAHHHAHAABBHBHHAHBHABBBABB

*000410_00290037 ABHHHHAAAHHAHHBHBHHBBAHHBBBAAAHHAHABBAABBHBHHABHAHHHBABHAHBAHHABBBBHAAHAHAHAABAAABBHHHHBBAHAHBBHBHHHHBBHAAHHBHABAAABHHHABAABBHBBAHAHHAHHBAHHHHHH

*001976_00120658 ABHHHHAAAHHAHHHHBHBBBAHHBBBAAAHHAHABBAABBHBAHABHAHHHBABHAHBAHHABBBBHAAHAHAHAHBAAABBHHHHBBAHAHBBHBHHHHBBAAAHHBHABAAHBHHHABAABBHBBAHAHHAHHBAHHHHHH

*005100_00047304 BBA-BHBHAHB-HA---B-AA-HBAAB-HHBHA--BHHAA-HHBHBB-AABHBAHABAHBHBHBHHH-A-HAHAAH-HBABBBHH-HHHBAHH-AAHHHA-HAB-A-HABHHBHAB-H-HH-HA-BBH---BHA-AH-AA--B-

*000932_00345916 AHAHHBHHABHBBBAHAHHHB-BBB-HHHAABBBBBHHAHBHBHBHAHBABBBHBAHBBHBBBB-BAHBABAAHBBBBHBBAHHAHHAHAAHHAHAAHHHHBBBHA-BHHBHHHBHHABHBBHBHHBHBHHHABH-H-HBHABH

*005281_00017546 BAAHHHAHHABHBABAAAAABAHHH-ABHHHABBBBAHHBHABAHAABHHHBHHABHBHHABHBABHH-HBHBHHHAAAHHBHBAHHBBBHBBAHHHABHBHHAHB-HHABHHAHBAHABA-HBAABH-BHHBAA-BBHHBHBB

*000056_00688313 ABHHHBHBAAABBHBHBABBBBAHA-BHAHHBAHHBBHAHHBHHHABAHAAHBHABAHHABBBHHHAHABBHHBHAAAHHB--HHBBBHHBAHBHHHHHHHHHAAA-BHHHBABABHHBAHHABBHABAAAHBH-BB-HB-BHA

*001500_00147902 B-ABHAHHBBB-BHAH-AHB-AHABAHBHHHHHABBAHHBHAAAHAHBBBBBHHHHABBHAAAHABAH-BB-BHABBHHBH--BHAHBABBBBAHHHAH-B--AHB-HHABHHHH--AABAB-B--BHB-HHHAA-H-HHHHBH

*000209_00285717 HHBHABHABHA-AHHHHHHBHHHBH-ABHBHABB-HABAHBH-BBAHBHBHAHB--BHHHAABHAHHHHBBAHHHH-AAHH---ABHBBAAAH-HAA-HBBAAAHA-ABBHBHHAAHABHH-BHB-HB--ABHHB-B-ABHAAB

*000595_00228187 ABHAHHAB-AH-HH-AAHBAHHHAHBHBB-HHHB-AH--AAAHBABHBBHAAAHH-HAHAHBHBBHH-ABH-BA-HBHHA-HHA-ABHHHHAHBBBBABBBHAH-B-HAAHHBHAB-HHHH-HB-AABB--HBHB-HABBBABB

*000151_00209285 BHHABBHHAAHHBHAABBHHAAABHAAHAHAHHHHAHBHBHBHHBAAAHHBAHBAHHAAHHBHBBAAHBHBHBBBHHHHHBABHBBAHHAAHHBHBAAHAHAABABHHBBBABHABHBBAAHHAHAHAHAABBAHHAHAHAHHH

*000183_00571266 HHHHAAABAHHBAHHBBABHHHHHAHHABHBAHABHHABABAAHHHHHAAHAAABHBBHHABBHHABBHHBHAHAHAAABAAAHHAHBHBHHHAHABBAAHBHHHHBBBAAHAAHHABAAAABHHHBBHHHHABHBHAAAHHBA

*000526_00292707 HHHHAAABAHHBAHHBBABAHHHHAHHABHBAHABHHABABAAHHAHHAAHAAABHBBHHABBHHABBHHBHAHAHAAHBAHAHHAHBHBHHBAHABBHAHBHHHHBBBAABAAHHABAAAABHHHBBHHHHABHBHAAHHHBA

*000590_00009473 BHHBHBBHBBHBBAHBABHABHABHBBHHHBBBAHABHABBBBHBHBHHHBBAHAHAHHHBHBHHABBBHHABABHAAHHBBBHHAHBABAHHHBHHABHBHABABHBHHHBBHHBHBBHBHABBHBBBHABHAHBHBHHHBHH

*000930_00113039 BHHBAHBBHBAAHAHHHHHAAABABBHABHAHHABHHHAAHBBAAAHHBABAHHBBAHHHHHHHAHAHAAAABHABAHAAAHAABHBAHHHHBABABHHAHAHHHHABAAAHAABBBAHHHHAABBHABHHAHBHHHHHHBBBA

*005578_00037532 HHHBBHABABBHBAAAHBAHBHHBAAAHHAABHAHBAAABHHAABHHAAAAHHBAHBHAHBAAHHHAHBBBHBHBBABHABHABHHHAHBBHHBABAHBAHHBBBBHHHAHBHHHBAHHBBBBBBHHABHBHHHHHBHHHHHBH

*006791_00001184 HHABBHHHHBHABAAHBHHHAHHBBBAABHHHHHHAHABHBAABBHBAAAHHBBAAHHAAHHBHHAHAHAHHBHHHHHAHHBAHBBAAAABHABHABHBAAHBABABHHAHAHBAHHBHABBHAAAAHHABHAHHBHBHHAABB

*000174_00342641 BBBAHBHAHHBABHHBBBHAAHHBAAAHAAHHA-BAAHHHHBHBBBABBAAAABHHBHHHHHBHABAHBHHAHABAAHAHHAAHBHHHBBBHAAHHAABHHHHAHAHBHBHHAAHAHABBABBAHHAHHBHBHABAAAHAHHAH

*000395_00304038 HBHAHBAAABBHBAAHHHHHHHHBAHHAHAAHHHBBAAHBBHABBHBAAHAHHHAAHBHHHHHBABBAHBHAHHBHBBHABAABHHBAAHBBBHAHABBABHHBBB-HHHHBHAHHAHBHHBBHBHAAHBBHBAAAHAAABHBH

*001262_00071268 AHBBHHAABBHBHHHHBHAAHHAAHHHHAAHHAHBHBHHBAAAAABHBAHBHBHHBHHBBHHAAHBBBABHABAAHABBHHABHAHAHAHAHHAAHAHAHBABHAAHBABBHBAAAABHHHHHAHBAHHBAHHAHHB-ABAHAH

*002002_00074675 BBHABAHHHAHAHBBBBAABBBBABAAABHBBHHHBHHBBHHAHAHAAHHAHHAABBHAABHBAABHAABHHHBBHHHAAHAAAHBAHABAHHBHBHAAHHBBHAABHAHHHBHHAHHAHHHHB-HHAHHAABAHAHBHHABAH

*003247_00026995 --AAHHHHB----B--HBABHBHH-AHAAA-HAAAHBH-BBBHHHAAHABAAHBB-HHAAABBHHABB-HHBAAAH-AAHBABH-HAHB-HHHHHAAABB-AAAA--AAHHABAA--HH-BBHH-BHBHA-HAHHHH-H--HH-

*007730_00001598 HH-BABBAHAB-BABHBHHAHHHBH-HHHAHBHBHHAAABBBHHBHHHHABABHHABHHBBBBAABAHBBBAAHBH-HAHBHAHHHHHABHHH-AAHBHABB-ABBAHAHBAAHHABABBABBHHHAHHHBHHAHAH-HAAAAH

*000677_00009346 AHHHABHH-AA-BH-HAABAAHHBHHHBBHHAHBAHAHBAAAHHBHAABABHHAH-HBHHABHHHBBHAHBHBHBHBHBHBB--ABBAHHAHBBHBHHBBHHHHAB-HAAHBBBA-AHBHBBHAAHAAHAAHAHABAAHHBHAB

*000050_00420280 BHABABBBBBAAHAHHAAHHHABAB-HABHHHBABAHHAHHHBAAAHHBHBAH--BHBB-HHBHHHHBAH--BAAB-HAAHH-HHBBAHHHHB-HAHHHAHAHHB--BAAAHAAHB-ABHAHHHBBHBBHBABBH-H-HBBBBA

*000206_00469004 -HABBHAHABH-BA-HBH-BAHHBBBAABHHHHHHAH-BHHAABBHBA-AHHBBAAHHAAHHBHHAHAH-H-BHHHHHAHH-AHHBAAAABHABHABHBAAH-ABA-HHAHAHBAHABHAHBHAAAAH-ABHA-H-HBH-AABB

*001318_00186762 HHAHAHBHHBHA-A-HBHABHHHBBBAAHHHAAABAAABHAHABBBBABHABHBAAHHHHAHBBAHHAHA-HBHHH-HAHHB-A-BAAAA-HHHHHH--AAABAB--AHAHAHB-HBBAABBH-HHHAHHBHHHABH-BHAAHB

*000284_00589459 BHHBHBHHBBBHBAHBABHABHABBHBHHBBBBHHABHABBB-HBHBHHHBAAHHAHHHHBHHHHABB-HHABABHAAHHBHBHHAHBBBAHBBBHBHBHBHABAHAHAHHHAHABHBBBBHHBBHBBHHABHAHBBAAAHBBH

*000548_00009158 BHHBHBBHBBBHBAHBABHABHABBBBHHBBBBHHABHABBBBHBHBHHHBHAHAHHHHHBHBHHABBHHHABABHAAHBBHBH-AHBHBAHBBBHHHBHBHABAHAHAHHHAHHBBBBBBHHBBHBBHHABHAHBB-AAHBHH

*000565_00069676 BBBAHHBHHHB-BHBAHBBBAHHBABHBBAHBBBHAHBBHHHAABHHAHAHHHHBBHHHBBAHHAHHHBHAAHBHHBBABBH-A-BABAAHHHBBAHBAAAHHHAHHABHBHAHHHBHBHHHHAHHAAHAAABBAABABHABBB

*004134_00004732 AHHBAHBBAHHBBHAHHHABHHBHHHABAABAHHBAAAHHBHAAHHBABHHBABAABHBHAABHAABA-HBAAHHH-HBAHBBABHHAAAHAHHHBAHHHHAHAABAAHHAAHBHBBHHABABHHBHAAAAHHHHHB-BHHAHB

*002101_00043571 H-AHAHBHHBH--AAHBHABHHHBBBAAHHHAHHBAAHBHAH-BBBBABHABHBAAHHHHAABBAHHAHAHHBHHHHHAHHBAA-BAAAABHHBAHHBHAAABABA-AHAHAHBAH-B-ABBHHBHHABHBHHHA-H-BHAAHB

*001278_00130024 AHBHABHHHAABHBHHABBAHHBBAHHHBHHABB-HAHBBAHAABABAHHHHBABBABA-ABAHABBABBHHBHH--HBBH--AHBH--HAHBHHHHHHBHAHHAAABHHBBHBB-HHBHHBAAAHAHHAAHAHBHABHHBBA-

*001070_00116458 B-BHAABHBHAAHBBAHBAHHHBBH-BHHBBBHABBBAAA-H-HHHAHHAHHHHHAHBABBHHAHBAHH-HHBHAABHHAAH-AABB-AHBBH-AB-HHHBAAHH--AAHBAHBAB-AA-H-HHBBHAA-HAB-HAH-BBBHAH

*000191_00474249 BBHBBHHHHHBA-A-HHBAAABABAHBHHBBHHBHHHHAHHHHBBHBA-ABAAB--HAHHHHHBHAB--HHA-ABH-HBABHBH-ABHBBABBBHABAAHBHABAA-HAB-HAHB-HHH-BHHABHBBHHABHAHBB-AA-BH-

*000970_00071715 AHHHABABAAHA-HAABBBBBHHHH-HAAAHABHBHBAHBAHBAHH-HAHHABAHHHHBAAHHBABBBBB-ABHHA-HAHH-BH-AHBHAH-A--HBAHBBB-AAA--HHHHBAHBHHBABAHBB-HBAHAHHA-HB-BH-HHH

*000548_00365786 BHHBHBBHBBB-BA-BABHABHABBBBHHBBBBHHABHABBBBHBHBHHHBBAHAHHHHHBHBHHABBAHHABABH-AHHBBBHHAHBHBAHBBBHHHBHBHABAHAHHHHHHHHBHBBBBHHBBHBBHBABHAH-BBAAHBHH

*002409_00044930 HHBBHHAABBABBHHHBBAHHHHAH-HHAABHHHBHHHHBAAHAHBBHBABHBAHBHABBHHAAHBBBBBAABBAAABBHAHBHHHAHA-AHHAAHBHAHHABHAA-AABBHBHAAAHHHBHHHBHHHABAHHAH-BHHBABHB

*001506_00143621 BAHAAHAAAAHABHHBBHAHHHBBHABAHAAABHHBHHBBAHHHBBBAAHHHBHAABHBHABHHHHBHHA-AHAHBHBBHHB-B-HBAABHHBHHHAHHBBHAHBA-BABABHAHA-AHAABBHHAHABHBBHHAHHHAABAHB

*001543_00116909 HAAAHAAHBBA-ABABHBABAHHHBAHAHABHAHAHHHHBBHHHHAAHHBAAHB--HHAAHABAHABHHHHBHAAH-AAHBABHHAAHBAHHHBHAHAHBAAAAAABAHAHAHAHAAHHBBBHAHBHBHBAHAHAHH-HAAHBH

*000164_00562148 HHHHHAHHH-HAAH-HAHAHAAAH--HBAAHAA-BBBA-H-BABHHBBAHBABBAABHBAAHBBBHBHHA-BHBAB-HHHH--H-AHAABHHHBAHAAHHHH-HH--ABA-BBAAH-H--BABHA-HB-B-HABB-BAHHHHAH

*000012_00333891 BAHBABBABABABAHBBHBAAHHBAAHBBAHBHBHAAHHBHBAHBHBHHABABHABBHHBBHBAABAHBHBAHHBHHHABHAAHHBAHHBHHAABHHHHHHBHHHBABHHHHHHHAHABBABBAHBABHBHHHAHABBHAAHAH

*000102_00419051 HBBHBHBABHBHHAHHBHABHHAHAHBAAABAHHHAAABHABHBHBABBHAAABHBBBHBAHBHHABBHABHBHBABHHBHAAHBBBABBHHHHHHHAHHHHHAHHABHBHAAHHABHHHABBHHHHAABHBAABAHAAABHAH

*000169_00035401 BBBHHBHAHHBHBHHBBHHHAHABAHAHAAHAABBAAHBHABHBBBABBHAAABHBBHHBAHBHAHBBBHBAHHBABHAHHAAHBHHABBBHHAHHHAHHHHHAHHHBHBHHAAHAHABHABBAHHAAABHBAABAHAAAHHAH

*000268_00327913 HBBHBBHABHBHBHHBBHHHHHABAHAHAAHAABHAAABHAHHBBBABBHAAABHHBHHBAHBHAHBBBABAHHBABHAHHAAHBBHABBBHHAHHHAHHHHHAHHABHBHHAAHAHHBHABBHHHAAABHBAABAHAAAHHAH

*000426_00014829 HBAAABHAHBHHABAHBHAHAHHHHBAHAAAAAHAHBAAAABHHHBBBHBBBAAHHBHBHAAHHHHBABABHBHBHAHAHHHAAAHHHHHHBBBBHBBHHBAHHBBHBBAABBBAHHHBHABBAAHHBHHBBHHAHHHBHHBAA

*007355_00007340 BBBHHBHAHHBHBHHBBHHHAHABAAAHAAHAABBAAHHHABHBBBABBAAAABHBBHHBAHBHAHBBBHHAHABAHHABHAAHBHHHBBBHAAHHHABHHHHAHHHBHBHHAAHAHABHABBAHHAAABHBHABAAAHAHHHH

*000298_00096822 AHHAAHBBHHH-BHAHHHABHHBAH-AHAAHAHABAAAHHBHAAAHBHB-HBHBAABHBHAHBHAABAAHBAHHHHBHAAHBAABHHAAAHAHBHBAHHHH-BAAB-AHHAABBHHBHBABHBHHBHAH-AHH-HBBABHAAHB

*000918_00242976 HHBBBABABHA-HBBHBHBHHBBHA-BHHHBHBABHAHHAAHBHHHBHBHBBHHABHBHAHHHBBABABA-HHBAB-HHBHAHH-AHBHHAABHHHABBAAHABBHHHAAAHHBABHAABABHHHBHHBABBBAAAH-HHHHBB

*002200_00040951 HBAHABHAABHBHBHHHHABHHHHBHHHAAHHAAAABAAAABBAHHBHHHAHAAH-BHBBHAHBBHBA-ABHBHBHAHAHBH-AAHHHBHHHBBBHHBH-BAHHBBABBAABBHB-HHBHABBH-BHB-ABBHHABHHBHHBAA

*002747_00029129 BBHHBHAHBHHA-BBBHB-HAAHB-BHHBABAABHHHHAAHBHHBBHAAABBBAH-AABHABHBHBHH-BBABHHH-BAAHHABAAABHHABBBHAHAHAHHABHHAHABAHHHH-AHHHBHAAHBHHHHHBBBAHHHAAABBB

*000312_00226721 HHHHABABHAHHBH-AABBAA-ABB-BHHBABHABAA-HABH-HBHAHAAHHHAB-HHBHB-HHHAHHAA-BAAHH-HHHH--B-AHHHBHAABA-ABHHBHHHA--H-AHHBHAB-H-HB-AA-BAB--ABABBBH-AH-A-H

*000062_00273351 BHBAHAAABHHB-ABHABH-HAABB-AHAAHAHABBHBB-BHHHHHABHHBHBHAHBHHHAHBAHBBABHHHAHBHBBAHH--BHAHAA-AHHAB-HABAHBHHBB-ABAHBBHH-HBAABBBHAHH-HB-HHHBABAH-HHAB

*000562_00355356 BAHBAABAAHH-HBB-HBBBBBB-B-HHHHHBBBBHAAHB-AABHAAHHAHHBB-BBBHHBHHHAABH-ABHAAHB-AHBBB-A-AHBBAAAHHHAHHBHBHHBHH-HAAAHAH-BB-AHHHBBAHHHHBHHBAHHA-BBHAHH

*000690_00278732 BHHBHBHHBBBHBAHBABAABHABBHBHHBBBBHHABHABBBBHBHBHHHBAAHHHHHHHBHHHHABHBHHABABHAAHHBHBH-AHBBBAHBBBABHBHBHABAHAHAHHHAHHBHBBBBHHBBHBBHAABHAHBBAAHHBBH

*001718_00039160 HHHBBBHHABBHBAAAHHAHBHHBAHAHHAABHAABAAABAAAHBHHAAAAHHBAHBHAHBHAHHBAHBBBHBHBBABBABHABBHHAHHBHH-HHAHBAAHBBBBBHHAHBHHHBAHHHBBBBBHHABHBAHHHHBHHBHHBH

*000161_00569110 -BHBHHHHBHA--B-HBAHHHBHHH-B-BBBHB-HBBAAB-B-BHABBABHHABBBBHBHBBHHBHHA--BABBAH-ABH---B-HHAHHHHBAHAHHBABBBAHABBBHBHHHA-H-AHHABHHABBH-HHH-BBBAH-HAB-

*002269_00106496 HHABBHHHHBHABAAHBHHHAHHBBBAABHAHHHHA--BH-AABBHBAAAHHBBAAHHAAHHBHHAHAHAHHBHHHAHAHHBAH-BAAAABHABHABHBHAHBHBA-HHABAHBAHHB-ABBHAAAAHHBBAAHH-H-HHAABB

*000468_00302654 BAHBHAAABBBHAABBABABBABAH-AHHHAAH-ABBBAA-H-HHHBHHAHBBAHHHAHBAHHABHAAB--BBAHHABBHHAAHBHHBBHAHB-HHBHAAHHBBHHHHAHHHAHAHAAABHHHBBHAB-HHABBHBAHHH-AHB

*000848_00157556 HBHBABBAHAB-BA-HBHHAHHHBHAHHHAHBHBHAAAABBBHHBHBH-ABABHHABHHBBBBAAHAB-HBAAHBHBHABBHAHAHHHA-HHH-AAHHHAHBHH-BAHAHBAAHHABABBABBHHHABHBBHHAHAH-HAHAA-

*002180_00034509 BBHBAHBBHBA--AHHHHHAAABABBHABHAHH-BAHHAHHBBAAAHHBABAHHBBAHHHBHHHHHAH-HAABHAB-HAAAHAA-HBAHH-HBABABHHAHAHHHH-BAAAHAABBBAHHHAAABBHABH-AHBHHHHHABBBA

*000150_00255792 --HBHHBHBAA-AHB-HABAHAH---AHBABBA-BAAHAHH---HHABHBBABH--ABBHBHHAAHAHAH-A-AB-HHHHBHAH-AHHABHAH-BBBABHB--AABBAHH-HHAHHB--ABHAB--HHH-B-HAAHH-AHHA-B

*003146_00070287 -HBBH--ABBHBHH-HBBAHHHHBHHHHAABHHABHHHH-AABAHBBHBABBBAHBHHBBHHAAHBBB-B-ABBAA-BBHAHAHHHAHHHHHAAAHBHA--ABHA--AABB-BAAA-HHHBHHHBHBHABAHH-HBB-HBABHB

*000233_00213562 BBHHH-HA-HA-HHAAHHBHA-H-B-BHHBAHAABHAAHAHHBABAABAAHBAHHHAHBHHABAHAH-AA-A-AABAHHAH-BBAAHA-BHAAAHHAABHAAHHHH-AHAHAHHHHAB-BBAAA-HHBAAHBBHH-H-AAHHBA

*009381_00000324 AABHAAHHAHA--HHAHBHBHHHB-HHHHABB-HHBAHHBHH-ABHHABBAHBAAAHABHBHBAAAAH-AABAAABAHABH-AA-ABHH-AHAHHAHBHHHA-HH-BHBHHABBA--BAHBAHHBBHBBBBHHH--H-H-BBAB

*001056_00211961 BHHABBAHAAHHBHAABBBHAHHBHAHHAHAHHHHAB-HBHBAHBAAAHHBAHBAHHAAHHBHHBAAHBHBHBBBHBHBHBA-HHBAHHAAHHBHHAAHAHAAHABAHBBBABHABAHBABHHAAAHAHAABBA-HAHAHAHHH

*002294_00173878 HHAAHHAHBBAHABABHBABAHBBHAHHHABHAHAHHBHBBHBHHAHAHHAAHBBBHHAAHABAHHBHAHHBHAAHAAAHBABHBHAHBAHHH-HAHAHBHAAAAAHAAABAHABHAHBBBBHAHBHBH-AHABAHH-HBAHHH

*001206_00244690 AAAHHAHAHAHAAHA-HHHAHHAAAAHHAHHAHAHHHAAAAAABAHAAHAAAAAA-HAAHHHAHAHAAAHHHAHHHAHHHAAAAAAAHHAAAHHAAAHHAAAAAAA-HAHHHAAHAHAAAHA-AHAHAH-AHAHAAAAHHAAHH

*002761_00007607 AAHBAHBHABHHHHAHABHBHHBHBHABAABAHBBHAAHHBAAAHABAHHHBABHBBHBHAHBHAABAHHBAAHBH-ABAHBHABHHAHAHAHBHHAHHHHAHAAHHAHHAHBBHBBAH-BABH-BHAB-AABHAHB-BHHAHB

*000206_00357757 BHABBHHHHBH--AAHBHHHAHHBBBAABHHHHAHAHABHHAABBHBAAAHHBBA-HHAAHHB-HAHA-AHHBHHH-HAHHBAHBBAAAABHABHABHBAAHBABABHHAAAHBA-HBHAHBHA-AAHHBBHA-HBH-HHAABB

*000308_00379782 HHBHAAHHBHAA-BBH-BHHHHBBHHBHABBBBABBH-AA-H-HBHAHHAAHAHBAHBABBHBHBBAH-HHHBHAA-BHAAHBAABBAHBBBHHA-AHHHHHHHHB-AAHHAHBAHBAAAHHH-BBHAAHBABHBAHBHABHHB

*000218_00230776 BBBHHBHAAHB-BHHBBBHAAHHBA-AHAAHHABBAAHHH-BABBBABBAAAABHHBHHHHHBHABAH-HBAHABA-HABHAAHBBHHHBBHHAHHAABHHHHAHB-BHBHHAHH--A--ABBAAHAHABHBHAHAA-HABHHH

*001252_00248553 BBAHBHBHB-B-BA-BHBAAAHHBA-BAHABH-BABHHAAHHHBBBBAAAB-BAHAAAHHHBHBHHH-----HHHH-HBABABHAAHHHBABB-HAHHH-HA-BAHAHABHHHHH-BBH-HAAAAHBH-HABHAA-H-A--HB-

*001828_00112227 AHAHHBHHA-HBBBAHAHHABHBBBAHHHAABBBBBHHAHBHBHBHAHBABHBHBAHBBHBBBBABAH-AB-AHBBBBHBB-AHABHAHAAHHAHAAHHAHB-BHA-HHHBAHHHAHHBBBBHBBHBHBBHHAHHHHBHHHABH

*000137_00148318 HBBBHHAABHBHBHAHHBBBHBHHABHHHHAABHBHHABHBB-AHHHHHHHABA--HBHHAABBABHABHAAAHBAABAHBABHHABHHAHAHHBHBBHHBHBABABBBBHHBBAB-HH-HHHHHHHAABBBHAHHB-BABAAB

*002145_00035980 BBHABAHHAABHHHBBBAAHBHBHBAAHBHBB--BBABBHBHAB-BHAHAAABAABHH--B-BAHHHH--HHHBB--HAAA-HAHAB--HAHBBAHBAAHHBBHBHAHAHHBBH--BHAAHBHAAHHHHHBH-AH-HHHB-BHA

*002383_00029542 HAH-HBHHBBH-ABHBBB-BH-HHH-HHAHABAB-HB-AAHBAHHH-HAAHAHBBAAHHHB-BBAABBAHBBHABBHA-AHABH-AHHHAAHBHBAHBBBAA-AAAAHABHABHA-HH--HHAH-HBBHB-HHAHAHBB-HHHB

*002594_00044710 --BBAH-H-AA-BA-BH-BAA-HHAAAHBABB-ABHA-AHHH-HHAAB-BBAHHABAHBHB-H-ABHH-AAA-ABB-B-H-H-H-AHHAAH-HAHBBHBHHH-AHB-AHHAAHAB-BH-AHHAA-BHHHBHAH-AHH-AH-HA-

*000027_00684968 AHAHAHBHH-HABH-AHBABHAHHAAAAABHAHBBA---B-H-B-HBHBHABHBA-BAHHAHBBAHH--A-HBHHB-HAHHB-A-B-AAAH-HHHBH-H-AA-AHA-ABHAAHBH-BB-ABBHH-HH--AH-HHHBB-B-AAH-

*001009_00195340 HHHAAB-BH-B--H-HHHABAHBHH-AAAHHAH--AA-HH-HAAHHBHBAABBBA-HAB-AHBBAHH-HB-A---HHAAAH-BA-HHAAAHHHAHBABH-AABAAH-AHHAAHHHABHBAB--A-BHA-AHHH-HHB-B--AHB

*000018_00322875 HHHHHBBBBBHBHAHBABHABHABHBBHHHBBBHHABBHBBBBHBHBHHHBBAHHBHHAHBHBHBHBBHHHABABHAAHHABBHAAHBABHHHHBBHABBBHABHBHBBHHBBHHBBBBBBHHBBABBHHHBHABBHBAHHBHH

*000018_00502312 HHHHHBBBBBHBHAABABHABHABHBBHHHBBBAHABBHBBBBHBHBHHHBBAHHHHHAHBHBHBHBBHHHABABHAAHHHBBHAAHBABHHHHBBHABBBHABHBABBHHBBHHBHBBBBHHBBABBHHHBHABBHBAHHBHH

*000018_00775433 HHHHHBBBBBHBHAHBABHABHABHBBHHHBBBAHABBHBBBBHBHBHHHBBAHHHHHAHBHBHBHBBHHHABABHAAHHHBBHAAHBABHHHHBBHABBBHABABHBBHHBBHHBBBBBBHHBBHBBHHABHABBHBAHHBHH

*000192_00372498 HHHBBHABABBHBAAAHBAHBHHBAHAHHAABHAHBAAABHHAABHHAAAAHHBAHBHAHBAAHHHAHBBBHBHBBABBABHABHHHAHBBHHBABAHBAHHBBBBHHHAHBHHHBAHHBBBBBBHHABHBAHHHHBHHHHHBH

*001056_00197409 BHHABBAHAAHHBHAABBHHAHHBHAHHAHAHHHHABBHBHBAHBAAAHHBAHBAHHAAHHBHHBAAHBHBHBBBHHHBHBABHHBAHHAAHHBHHAAHAHAAHABHHBBBABHABABBABHHAAAHAHAABBAHAABAHAHHH

*001229_00112235 HHHHHHAHABBBBAAAHHHBBHHBAHAAHAABHABBAAABBAABBHHAAHAHHHAABBHHBHABABAABBHHHHBHABBABAABHHHAHHBHHHABABBAHHHBBBHHHAHBHHHHAHHHBBBBBHHAHHBHHAAHBAABHHBH

*001311_00034077 BHHBHBBBBBHBBAHBABHABHABHBBHHHBBBAHABBHBBBBHBHBHHHBBAHHHHHHHBHBHBHBBHHHABABHAAHHHBBHHAHBABAHBHBHHABBBHABABHBBHHBBHHBBBBBBHHBBHBBHHABHAHBHBAHHBHH

*000078_00199497 HHBAAAAAABABAHHHBBHBHBHHHHABHHHAHBHHHBHHHHBBHABBAHAABHBHBAAHAABHAHBAABHABBAHHHHHHABHABHBBHAHHABHHBBBHHAHBAHAHBHBHHHHAAAABBBHAHHHBHBBBHBABBABHAHB

*000562_00165227 HAHBAABAAHHABBHHHBHHBBBHBBHBHHHBBBBHAAHBAAABHAAHHAHHBBHBBBHHHHHHAABHAABHAAHBBAHBBBBAHAHBBAAAHAHAHHBHBHHBHH-HAAAHAHHBBBAHHHBBAHHHBHHHBAHAAHBBHAHB

*000726_00142304 AHABHABHAHHBBAHHBHBBAHHAABABHAHBHBHHBBHBHAAHAHHAABHHHHABHHHHBAHHAHBAHBHABABAHAAHAHHH-BAHHBBAAAHHAABAHHABHAHHHAAHHABHBHABHAHAABABABHHAHAAHABHAAHA

*002386_00068187 HBBHBHBHBAHHAAHHHHABHAHHHHBAAABABHAAAABHABBHHBABBHAAABABBHHBAHBHHAAH-ABBBHHABHHBBHAHHBBABBHHHBHAHHHHHHHHHAABHBBAAHBABHHAAHBHHHHAABHBAABABAHABHHH

*000024_00167052 BHHAHHBAHBH--HAAAHHAHHAAA-AAAHBHBBHHA-BBAAHBAHHBHHBHHAABHBHAHAAA-HHAAHAHHHBBAH-BBHHHAHHAHAHAABAHABHAHH-BHB-HAHABBBABHHBBB-HHBHAA--HBABBBH-HBHABB

*003664_00009776 HBBBHAAAB-HA-H-HBBAHHHAHHHHHAABHHABHHHABAABHHBBHBH-HBH-BHHBBHHAAABBB-BAABHAAABHHAABA-HAHHHBHH-AHBHAHHABHAA-AABBHBH-AAA-HBAHHBHAHHBAHAAAHBAH-AHH-

*001288_00120685 HHAAHHABBBA-BB-BHHABAHBH--HHHHHAAHAHHB-BBHBHHAHAHHABB--HHHAA-ABABABA-HB-BAAH-HHBAAHHH-A-BHA-AHHAHAHBBH-ABA-HAAHAHABH-HHBBBH--BABHH-HH-A-H-B-ABBH

*001660_00095573 HBBBHHAABBH-AHAHBBHBBHH-BBHHAAHHAHBHHHHBAAHAHBBHBABABHHBHABBHHAAHBBB-BAABBAA-BHHAHAH-HAHHHHHAAAHBHAHHHBHAA-HAHBHBHA--BHHBHHBBHBHABABHHHHBAABABAB

*005559_00033431 AABH-HHHABHHAHBBHBHHBHBHAHAHHBBH-BBABHHHHBBA-HBAHAAABHHHHAA-HBBBAAAHBBHBAHHB-BHBH-HBBHB--BHBHAHBABBBBAAHHHABHHAHABH-HAHBHAHBBAAHBBHBHHBBBAHHBAHB

*000315_00295162 A-HAHHHA--B-AHAHBHBBBAHABABAAAAHAHAHB--HBA-HAHHH-HHHBHB-HBHHB-AH-HB--AHAHAHAAB-AA-B-HBHABHHHH-A-HBBHHBAHA--BBHAB-HA--BHAB-HBBHBBA--AB-H---H--AH-

*000922_00381172 BHBH-BHA-BA-AH---HHBHAHH--ABHBHAHB-HAB-H-BBBHAABHHAAH-HBBHABAABAAHHHBHBHBBAH-A-HH--A-BH--AAHH---HBHBBAAHHA-AHBHBBH-BHABA-HBHHHAB---BHHBAB--BHAH-

*007853_00001604 HBHBAAH---BA-HAHAABBB-AAB-ABHHHHH-HBHHHBHA-HAHHBBBBB---HHHHH--ABHHHA-B-AHAAB-BABA-AB-AHHABBBBAHBHAHABBBHH--AHABHAHHA-A-B-BH--ABH-AHHH-H-H-H--HB-

*000562_00334100 HAHBAABAAHHAHBHHHBHBBBBHBBHHHHHBBBBHAAHBAAABHAAHHAHHBBH-BBHHBHHHAABHAABHAAHBBAHBBB-AHAHBBAAAH-HAHBBHBHBBHHHHAAAHAHABBBABHHBBAHHHBHHHBAHHAHBBHAHH

*001959_00022618 AHBBHHAABBHBHHAHBBAAHHAAHHHHAAHHH-BHBHHBAABAABBBAHBHBHHBHHBBHHAAHBBBABAABAAH-BBHHABHHHAHABAAHAAHHHAHHABHAAHHABBHBAAAABHHHHHHBBAH-BAHHAAABHAHAHHH

*003463_00074445 HHBB-HAABBHH-HBHBBAHBHAHHBHHAABHAB-BHBHBAAHABBBHBHBHBHHBHHBBHHAAHBBBBBAABHA--BB-A-BHHB-B-HHHHAAHBHAHHABHAABAABBHBH--HHABBHH-BHHHHBAHBAHHB-HB-HHB

*001076_00036745 HBHHAAAHBBHBAB-HABABBHHB---BAHBHBB-HAAHHABAHHABH-HBBH-HHHBHAHHHAHABA-ABHBHHH-ABAHHBHBAHHHHHAB-HHAAHBHAAHBBBHHBBABHA-HHABBBBB-HBBH-AHHAB-B-HBHAAB

*000304_00390807 AHBBAHA-BBH-HH-HBBAHHHH-BHHHAABH-HBHHHHB-A-AHBBHBABHBHH-H-BB-BA-HBBB-B-AB-AAABBBAHBH-HAHABHAAA-HBHAHHA-HAA-HAB-HBHAAHA-HBHHHB-B--B--HA--BHHHABHB

*001124_00049537 B-HHAHAH-BA--B-ABAABAB--HHHAHHHABHHA-ABH-B-AHABHAHHHAAA-BABAAHHHHHBB---A-HAH-B-AHA-BAHAH-BAHA-HHBBHAABAHBH-BHAAHHBBBA-HHHHBA--HBH-BAHBAHH-H-AHAA

*004712_00032147 A-HHAA-HB-H-ABHAHBAAA-A---BBAAHHBBAH-AAHBBHHHA-HHBHAH-HAHHBAHAHAAH-AAA-BHHHHAAHHH-HH-ABHHBAHH-BBAHBBAB-HA--B-BHABAA-BBA-HHH-HHHHBB--BA--AHBH-AAB

*000274_00268918 ABHBH-BH-AA--B--BAHHBAH-HBHHBHHHH-HHBHABBB-HBABH-BHAA-ABBHBHHBHHBHHAHB--BBAABA--B--BB--AH-BHBHHBHBBAHBHA-A-BHHBAHHAA--HH-ABAHHBBAAAHHB-HB-HHHAB-

*001715_00053487 HHBHHBAHBBHBBBABHBAAB-BHHAAAAHHBBA-BHABHBHABHBBAABBBAHHHHAHABHHHBABHAHAH-AAHHBBAHAHBABBAHHHABHHHAHHHHHAHHH-BAH-HAHHAABBHHBHB--HAHAHABBHBB--HBAHB

*000022_00473187 HHBABBBHBHB-BHBHHHBHHBAHHAHHHBBHBBABH--HBHBAHHBH-HBHHBABHAAHHHHBBAHHBHBBBHHH-BHABBBA-BHBBBH-HBABBBBHHHBAHABHBHHBBHABBHHHH-HHHBBBAAHHBAB-BBBBBBBB

*000031_00263085 HBHBHHHB-HB-BBBBHHHBH-BAA-AHHHHHBHBHHAHHAB-HAHHHHHABHBBBBBABAAAAHAAB-HBHBABBHAH-BAABBAHABBHHHHBAHBAHBBBHAH-AHHBAHAHBHAHHHAHAHBABBHABHA-BHAAHHA-B

*000983_00098367 BBHHBHAH-HH-BBHBHBHHAAHBAAHHBABAABHHHHAH-BHHBBAAAABBHAHAAABAABHBHBHH-B-ABHHHABHAHHHB-HABHAA-BBHAHAHABHABHH-BABAH-HBBAHHHBHAABBHHHBHHBBAAH-AHABBH

*000587_00184022 HHABABBBHHA-BAHHHHHBBABABAHHAHAABAHABHAA-HHAAAHHBHBAHBHBHBHAAHHHHHHBHHAAHHABAAAAHHAHAHBAHHAHBAHAHHAHHHHBBA-BHAHBAAHBHABBHHHBHBAHB-BABBAHBAHBBBHA

*000289_00205204 A-HAHHABBHHBHHAAHHBAHHH-H-HBBBHHHBHABHHAAAHBHBBBBAHHHAHAHAHAHBHBBHHHABHHBABBBHHABBHA-ABHHHAHHHBBBABBHHAHBBBHAABHBHAB--AAHHHHHAABBBBHBABBHBBBBABB

*002311_00019243 AHAHABHH-AABBBHHAABAAHHBA-BHBHHAABAHAHBHAAAHBHHABABHHAHBHBHAABHHHBBHAHHHBHBH-HBHBHAAABBAHHAHHHHBHHBBHA-HAHBHAAHBBBAHBHBHBBHA-HAAHAAHAAABAAHHBHAH

*000160_00183207 ABHHHBHH-HHBHHHHHABAA-HHHBHBBBHAABHAAHBAAA-HHBHHBAHHHABHHAHHABHHBAHH-B-HBABHBHAABHHAAHBAHAAHBBBBHABBAHAHBBBHAAHBBBABHABAHHHAHAAAAHBAHAA-HABBBHHA

*000132_00069761 BABBH-AA--B-AAB-H-AB-ABHH-AHHA-AAHABBBHBAHAHHHBHHAHB--H-HAHB-HHA-HAA---HBAHH-BBHAA-H-HHBBHAHBAHHBAAABHBB---HAHHHHHA-----HHBB--ABBAHABA--A-HA-AHH

*000035_00713647 AAHHHAHHH-AABBHAHBHBHHHBBHAHH-BBAAABAAAB-H-ABHHAHBAHBAABHABHBABAHAHH-AABHAABHH-BBAAA-ABHHB-HA-AHHHHHH-BAAH-BHAHABBH--BAHBAHH-BH-B--HHHHAH-HBBBA-

*000429_00240771 AHBBHHAABBBBHHHBBHAABHHAHAAHAAHHHAHHBAHBAAAAABHBAHBHHHHHHHBBHHHAHABBAHAABAAHABBHHABHAHAAABAHHAAHAHAHBAHHAHABABBHBAAAAHHHHBHBHBAHHHAHHAHABAAHAAHH

*001222_00252736 BBHABAHHHAHAHBBBBAABBBBABAAABHBBHHHBHHBBHHAHAHAAHHAHHAABBHAABHBAABHAABHHHBBHHHAAHAAAHBAHABAHHBHBHAAHHBBHAABHAHHHBHHAHHAHHHBBAHHAHHAABAHAHBHHABAH

*001505_00087044 BAHAAHAAAAHABHHBBHAHHHBBHABAHAAABHHBHHBHHHHHBBBAAHHHBHAABHBHABHHHHBHHABAAAHBHBBAHBHBHHBAABHHBHHHAHHBBHAHBAABABABHAHAAAAAAHBHHAHABHBBHHAHHBAABAHB

*000379_00172998 HHBBBABAA-H-H-BHBHBHHBBHA-BHBHBABHBHAAHAAHBHHHBHBHB-HHABHBHAAHHHBABAHAAHHBAH-HHBHABHHAHBHHAAH-HHABBAAHAB-BHHAAAHHBABBAAAABHHH-HAAH-B-AAAHBHAHHBB

*000477_00283947 BHABBHHAAHHBAHAAHBHBBAHBB-AHAAHHAHBABHHHAA-ABBHHH-HAHABHHAHHHHBAABBA---HBBHA-HHHAHAH-HAHBHHAAAABBHABHBBHAABH-HBABHAAHBBHHAHBHBBBABAHHAH-HBAB-BHH

*003571_00044178 BAHBA-AHHAA-AHHABH-HBAHBB-HHHA-BAH-BAHHHAH-ABBHABBHHBAAHHHBABHBBBAAHAHABAHAHAHABHA-H-HBHAAAAA-HHBAHAHHBHHHBBHBHBHHAAHAAHBAHB-BAAHAAHHHA-HHHABBAB

*000666_00321997 ABHAH-ABBHAB-HHAAHHAABHBHBBBBBBHHBBAHH-AAABBHBHBHHHHHHHAHAHAHHHBBHH-AB-ABABH-HHABHHABABHHHHHHABBBABBHHAHBB-HAAHHBHA-AA-HHHHHAAAB--BHB-BBH-BBBABB

*000590_00213425 BHHBHBBHBBH-BAHBABHABHABHBBHHHBBBAHABHAB-BBHBHBHHHBBAHAHAHHHBHBHHABBHHHABABHAAHHBBBHHAHBABAHB-BHHABHBHABABABHHHBBHH-BHBBBHAB-HBBHBABHAHBH-AHHBHH

*000437_00052544 BAHAAHAAAAHABHHBBHAHHH-BHABAHAAAB-HBHHBB-HHBBBBAAHHHHHAABHBHABHHAHBHHABAAAHH-BBAHBABHHBAAB-HB-HHAHHBBHAHBAABABABHAHAHHHAHHBHAAHAHHBBHHAAHBHABAH-

*000277_00226529 HHAAHHHBBBAHHBABHHABABBHHHHBBHHHAHAHHB-BBHBHHABAHHABHBBHHHAAHABAHABHHHBBHAAHAHHBAAHBAHAHBHAHHHHAHAHBBHAABAHHAABAHABHBBBBBBHAHBABHBBHHBABHHBBABBH

*000713_00118116 HHABABBAHHHBBAAAAHHBAAAHHABHAHHAAHHHHABBHBHBHBBHHAAHHHHBAAHAAAHHABAABABAAHAHAAHHHHBBHHHABAHHHHAAABHHABHHAA-HHHHAHHHBBABBAAAABAAHHBAHHAHABAAHHHBA

*001505_00090603 BAHAAHAAAAHABHHBBHAHHHBBHABAHAAABHHBHHBHHHHHBBBAAHHHBHAABHBHABHHHHBHHABAAAHB-BBAHBHBHHBAABHHBHHHAHHBBHAHBAABABABHAHAAAHAABBHHAHAB-BBHHAHHHAABAHB

*000011_00356286 AHABABBH-HA--AHHBBHBHHHBHABBBAAA-AAHBHBHBHBBBHHHAHAH--HBAHBAAAHHHABB--BBHHAH-A-AH-BHAHHAAHHHAAAAABAHABABBH-HBHHHHHA-HA-BHAAHAAHA-H-AB-A-B-HHHHHA

*000132_00517663 BABBHAAABBB--ABHABAB-ABHHHAHHAAAB-A--BHH-HHHHHBH-AHBBAB-HAHBAHHA-HHA-AAHBAHH-BBHAAAABHHB-HAHBA-HBAAABHBBH--AHHAHHHABAA-HBBHHAHAB--HHBBHAHHHHBAHH

*000492_00231641 ABAHHBBAAA-AHA-BHHBHBBHHBABHAHHHABBBHB-B-BBAHAAH-HHHHAAAB-H-HBHHHBAH-H-HBHHHAHBABA-H-AHHAHBBA-BHHHBAHH-AAAABBH-BHHA--HB-BHBBHH-BH--HAAABBBHBHBBA

*003393_00065377 BAA-HABHBHB-HAAH-AABBHHHHHABHHAH-HBBAHABBAHAHAHBHHBBHHHBHBHHHAHHAHA-BHB--HHBAAAHHBAB-AHBBBA-HAHBHAHABHHAHB-BHABHHAH-AAABA-HBAABHB-BABAABH-HHHABB

*001026_00237534 H-HBAA-BHHH-BA-HHA-BHHB-HHAAAAAABHBAA-HH-H-ABHBHBHABH-AABHBHAHB-AHB---BABHBHBH-AH--A-HHAAAHHH-BBAHHAHA-AAHHAHAAAH-A--H-ABHB-H-HAA-AHH-AHBAB-HA--

*000524_00226834 AHHHAH-BH-H--H-HHHA-HHBB--AHAAHA-BBAAAAHBBAAHHBHBHABHBAAHAB-ABBB-HBAAH-ABHHH-HAAB-BA-H-AAAHHHABBA-HAHA-AHH-AHH-AHHHHBH--BH-H--HAB--HH-A-B-B-BABB

*000587_00133829 HHABABBBHHA-BA--AAHBBABAB-HHAHHABAHABHAHBHHAAAHHBHBAHBH-HBHAAHHHHHHBAAAAHHABAHAAHHHHAHBAHHAHBAHAHHAHHHHBBA-BHAHBAAHHBABBHHH-HBAH-BBABBAABHHBBBHA

*000565_00285966 -BBA-HHHHHBA-H-AHBBBAH-BABHBBAHB---AABBHHHHABHHAHAHHHHBBHHH-BAHHAAAH--AAHBH--B-----ABAA--A-HAHBAHBAAAHHHAHBABHHHAHA--HHHHAH-HHAAAHAA-BAABABA-BB-

*006410_00031002 H-ABBHHHHBHABAHHBHHHAHHBBBAABHHHHHBAHABHBAABBHBAAAHHBBAAHHAAHHBHHAHA-AHHBHHHHHAHHBAHBBAAAABHABHABHBAAHBABABHHAHAHBABHBHABBHAAAAHHA-AAHHBH-HHAABB

*000930_00227397 BBHBAHBBHBA-BAHHAHBAAABABBHABHAHHABHHHAHHBBAAAHHBABAHHHBAAHHHHHHAHAHBAAABHABAHAAAHAABHHAAHHHB-BABHHAHAHHHH-BAAAHAABBBAHHHHAABBHABHAAHBH-HHHHHBBA

*000044_00026052 ABHBABBAAHHHABHBHBHBHHHHHHABBA-ABBHAHAAAAH-HHHHHHHHBBBBBHBBHAHABBHAABBBHBABHAAHHBAHBBAHAB-H-HHBAABHBBBHHABBHHHBABABH-ABHHHHHAHAHHBABHBAAHAABHHHB

*004491_00135618 AHHHABAHAHHBBHHABHBBBHHBHBHAAHHABHBHBAHBHBBHHHBHABHABAHHHHBAAHHBHBB-HBAABAHA-HAHHBB-BHHBAAHAHBBHBAHBBBHAAHBHHHHHBAAB-BBABHHBBBHBABABHAABBHBHHHBH

*000046_00162108 HBBBHBBBBBHBHBHBBBHHBBHHBHBBHHBHBHBHHBBBBBBHHBHHBBBBBHHBHBHBBBHHHHHBHHHBBHHHBBHHHBHBHHHBBBHBHBHBBBHHBHBHHBBBHHBBHBBBBBBHBBBBBBBHBBBHHBBBHHHBBBBB

*000013_01214252 -B-AHHAHBAHAA-HAAB-AHAHB--H-BHHHH-BA---AAAHBHBHB-HH-H--AHAHAHHHBBHHA-B---ABBBH-AHB-H-ABH--AHHA-BBABBH--HB-BAAA-HBHA-A-H-H-H--AABH-BHBHHBHBB--A-B

*000127_00383073 B-HAHHBHHBA-AHAAAHAAHHAAA-AHHHHABBHHH--BAHAAAHHBHHBHHAAHHBHAAAAHAAHA-HABHABHAHBHBBBHAHHHAHHAAAAH-BHABB-BBH-AAA-HBBABBBHBBHHH-AAA-B-BABH-H-HBHHB-

*001565_00163929 AHHH-HBBAHH-BH-HHHABHHBHH-AHAABAB-BAAA-HBHAAHHBA-HHBH-AABHBHAHBHAABA-HBAAHHH-HBAHBHABHHAAAHHH-HBAHBAHAHAA--A-HAABB-AHAAABABH-BHAHAAHHHHHBABBHABB

*000598_00088964 HHHBBH-HABB-BAAAHHAHBHHBAHAHHA-BHAHBAAABHA-HBHHAAAAHABAHBHAHBHAHHHA--BBBBHBBABBABHAB-HHAHHBHHHHHAHBAHHBBBB-HHAHBHHHBAAHBBBBBBHHAB-BAHH-ABBHBHHBH

*003222_00051159 AHHBHHABAHB-BBAAHBBAHHBBA-BBHBABHABHHHABABBHBHHHAAAABHHHHHHHBAAHHHBA-AAAHABB-HHHAABBBAHHHBHAABAHBHBBHHBHABAHBBHH-HA-AAAABHH-HBAH-BAHAHHBH-H-HAH-

*000946_00239466 H-AAHBABBB--AB--HH-BAH-HH-AHHHB-ABAHHB-BBHBHHAHA-HABHHB-HHAABABAHABBHH--HAAH-HHBAAHB-BAABAA----AHAH-HH-AH--AAA-AHA-HA-H-B-HAHBAH-HBHHBA-HHB--BBH

*000526_00170115 HHHHAAABAHHBAHHBBABAHHHHAHHABHBAHABHHABABAAHHAHHAAHAAABHBBHHABBHHABBHHBHAHAHAAABAHAHHAHBHBHHBABABBHAHBHHHHBBBAABAAHHAHAAAABHHHBBHHHHABHBHAAAHHBA

*002668_00030151 HHABABBAHHHBHAAAAHBHAHAHHABHHHHAAHHAHABBHHHBHBBHHAAHHHHBAAHAAABHABAABABAAAAHAAHHHHBBHHHABAHHHHAAABHHABHHAAHABHHAHHHBBABBAAAABAAHHBAHBAHABHAHHHBA

*003475_00020546 BHHBHBBBBBHBBAHBABHABHABHBBHHHBBBAHABBHBBBBHBHBHHHBBAHAHHHHHBHBHBHBBHHHABABHAAHHHBBHHAHBABAHBHBHHABBBHABABHBBHHBBHHBBBBBBHHBBHBBHHABHAHBHBAHHBHH

*001461_00159776 HAAAHHHHBBAH-BABHBABHBHHBHHAAHAHAAAHBHHBBHHHHAAHHHAAHBBBAHAAHHBHHABBAHHBAAAHAAAABABHBHAHBAHHHAHAAABBAAHAAAAAABHABAAAHHHBBHAHBBHBHHAHAHHHHBHAAHHH

*003508_00005520 HB-HHBAAHHA-HB-A-BBHABHBBBHHBHBHAAHHHAHHBH-ABA-BAAABHH-HAHBHHHBHHHBBAA-HBH-B-H--BA-B-ABA-BHAABHHAAB-AAHHHA-HBAHAHHH-ABBBBHA--ABB--HHBABBH-HHHHBA

*000374_00046646 A-H-H-ABAHH--BA-BBBAHAHBBBBHHBABH-BHHAAHBB-HBHH-AAAABHHAHH-HBAABHHBH----HABB-HAHHAAB--HHHBHAAAAH-HBBHH-HAB-HBBAHBHA-AA-ABAHHABAH-BAHAHHBH-AHAAHH

*007050_00002931 A-H-HBAB-HH-BB-AHBBABHBBA-BHHBAB-ABHHAABHBBHBHAHAAAABHHHHHBHBAHHHHBH-ABAHABBAHHHH--B--HB-BHAA-AHBHBBBH-HAH-HBBAHBH--AAHABHBHBBAHB-AHAH-BH-H--AH-

*000347_00316268 BAHAHAHABBH--ABBBBHBHHHAH-AHHAAAAAABABBA-H-HHHBHBAHHBAH-HAABAHHABHHABA-B-AAH-BB-A--H-BHBBAAHBAHBBAAABHBBH---HHHHHHAAHHAHHHHHAHABH-AHBAAHH-AHBAHB

*001361_00090100 AHHAHHAA-HHBAB-BHBBBH-HHB-HBAAAHBBAAH-BHBHHHBHHAH-HBH-BBHHBHBHABHHHH-B-ABABHHHHBBAHHAAAAB-HBH-BHAHHBHBBHHBBBBBHHBAB--A--BAHHBHHBH-HBBBAABBA-AAA-

*004507_00005361 BBHAB-AHAAH-AHAAHHBHHAHBB-HB-HHHHBHAHBHBHBBBHHAAHHAHHA--BAAAHBHHHBHHHBBH-HBB-HBBHABBBHHAH-HBB-AAHABAHAAHB-BABBBH-AHBA-BHBBBBAHHAB-BBA-HBH-AH-HB-

*000091_00138155 HABABAHHBABHHBBBBHBHA-BAHBH-BH-HH-HHHH-H-HBBAAHHABBAAHHHBHHAHABABHHB-HB-AAHBABAAHH-BAAHAHHABA-H-AABHHHBBHH-AHBHHBHH-HBABBABAA-HAB-HBABAAH-AH-HB-

*000768_00130334 HHABBHBABHHAHBBHBB-HHBBHABBHBHBABHBHAHH-AH-HHBBHBHHBHHABHBHAAHBHBABABAAHABAHBHBBHHBHBAHB-HHAH-HHABBHHBABBA-HAAAHABABBAHHABHHHHHABH-BBAAHABHAHHBH

*000077_00265268 BAHBAHHBHBAAAHHHBHABABBHB-HAHHHAHBHABABH-ABAHBBHABHHABHHBABAAHHHAHHBAAHAHHBHBBAHBABBABHHBBABAHAABHHABBABBHBHHHHHHBHBHAHHHBBABAHHHHBABBBHH-HBHBAA

*000099_00565344 HBBABHBHHAH-AAHHHBABHAHHBABAH-BABHAAAAAAAHBHHAABBHAAABAHBHHBAABHHAAHAAHHHHAA-HHBBHAHABBHBHHHAHHAAHHHHHHHHAABHBBAHHBABHBAAHBHHHHAABABAABHBAHABHBH

*001018_00064034 ABBABABABHBAAAHAAHHBAHBHHAABHHHHAHBHHAHAHH-HHHHBAAHAHHAABHHHHHHHHAHAHHABBHBBBABBHBBH-BBHABAHAHBHHHBBAHHHHBBHBHBABBHBBHAAABBABHBBB-HAHABBH-ABBHBA

*000049_00505950 -BHABAHHHAH--H-BBAAA-BBBBAA-BB-B-AHBA-BHAH-AHH-AAAAAHA-BHHAAB-BAHHHA-B-HHBBH-HAHAAAA--AAHHAHHBBHH-AHHBBH---HAH-HBHH-HH-BHB-B-BHAH-AHBAH-HHH--BAA

*000111_00666458 -BHHBH-BH-HAAHHBAAAHH-AB--BHA-HAAABBHH-H-HABBH-HBHBAH--ABHB-AHBB-HBA-A-BHBABH--HBHBH-AHAA-HAHBAHAAH--HHHA--HHAHBBAAA-HH-BABHA-HBBBAAH-BAB--AHHH-

*000055_00772864 -BABHHBBAAB--HBHH--AHHBAA-HBHHHHHH-BH-BH-BHHAHHHBAAB--A-ABAHAHABBAA--H-ABABB-HHBB--B--AAHB-BHBAAH-AABB-HHH--HHBAHAHA-A-BA-HHABAABHHHH-BHHHA--AHB

*000660_00133890 HHA----H-AHHBHHA--AAHHHHH-BBAAHAH-HHB-BBAB-HHAAHBHAAHB-BA-BHAHAHHAHBAHABBHHBBAABABBH-HHH-BHBBAB-A-BBHH-AA---BHHAHH-HABBHAAAHAAHHBAAHHHHBBAB-BBH-

*000523_00212436 HHHHAAABHH-B-BHHBAHHHHHHH-HHBA-AHABHHA-HBH-HHH-B-AABAAB-BBBHABHBBABB-ABHHBABAHHHH-AH-AHBBBAHH-HABBHAHBHHBH-BBAAHAHHH-BAAAABB-HBBH-BBABHBH-A-BHBA

*003768_00041447 BHHBHAAABBB-AABHABABBABHHBABHAAAHAABBBHHHHHHHHBHHAHBBAH-HAHBABBABHAA-AAHBAHH-BBHHA-H-HHBBHAABAHHBAAAHH-BHB-HBH-HAHA--A-HHHHBBHAB-AHABHHBAAHHHAH-

*001372_00008496 AHBBBHAH-BB--HBHBHAAHHAAHBHBAAHHHHBHB-HBAAAAABHB-HBHBH--HHBBABAAHBBB-HAABAAHABBHAA-A-HAHAAAHH-AHAHAHBAAHAA--ABBHBHAA-BHHHHHHHBAHHHAHHAHHB-ABAHHH

*000770_00262837 AHHHABAHAAHABH-ABHBBBA-HH-HAAHHAB-BHBAHBHHBAHHBHAHHABABHHHBAAHHBHBBBBBA-BHHABHAABB-HHHHBHAHAAB-HBAHBBBBAAH-HBHHHBAHBA-BAB-HBBHHBABAHHAAHBBBHHHHH

*007699_00008452 BBHA-AHHAABH-HBBBAAHHBBHBAAHBBBBHAHBAB-ABH-BHBBAHAAABAABHHAABBBAHHHH--HBHBBHHHHHAABAHAHAHBABHBAHBAAHHBBHB-AAAHHBBHHABHAHHHHH-HHHBHBHBA-AH-HBHBHA

*000294_00045792 AAHBBABBABHBBAHAHHBHBABAHBAHHABHHHBHHHHHAABA-HBHAHHHBABBHHB-HBBBHABHBBAHHHABAH-BB--BBH-B-HBA-HHAHABBHBBHHHBAHBHHHH--BBABB-BBHBABHHHBBHAAHBHHABHB

*000507_00264715 BHHBHBBHBBBHBAHBABHABHABB-BHHBBBBHHABHABBBBHBHBHHHBBAHAHHHHHBHBHHABB-HAABABHAAHHBBBH-AHBHBAHBBBHHHBHBHABAHAHHHHBHHHBBBBBBHBBBHBBHHABHA-BBHAAHBHH

*000012_00138783 BAHBABBHBABABABBBHHAABHBAAHBHAABHBHAAAABABAHBHBHHABABHAHBHHBBHBAABA--ABAHHBH-HAHHAAHHHAHHBHHAAHHHHHHHBHHBBABHHHHHHHABABBABBAHHAHHBAHHA-AHBHAAHA-

*005365_00009124 HBBHBHBHBHB-BABHBHABHHAHAHBAAABAHHAHAABHABBBHBABBHAAABHBBBHBAHBHHABBAABHBHBABHHBH-AHBBBABBHHHHHHHAHHHHHAHHABHBHAAHHA-HHHABBHBHHAABHBA-BAH-HABHAH

*000005_00962963 AHBBHHHA--H-HH-AB--BB-HBB-HHAABHA--H--HB-A-ABBBHBHH-H-B-HAHBHHBA-BBB-B-HHBHA-H-HAB-H-HAHHH--A-AHBHAHBB-AA-BHAHBHBHA-HBBBBHAB-A-HA-ABH--H--ABABAH

*000704_00018802 BAHAAHAAAAHABHHBBHAHHHBBHABAHAAABHABHHBHHHAHBBBAAHHHBHAABHBHABHHHHBHHABAAAHBHBBAHBHBHHBAABHHBHHHAHHBBHAHBAABABABHAHAAAHAAHBHAAHABHBBHHAAHHAHBAHB

*004350_00038321 HAHBBHABABBHBAAAHBAHBHHBAHAHHAABHAHBAAABHHAABHHAAAAHHBAHBHAHBAAHHHAHBBBHBHBBABHABAABHHHAHBBHHBABAHBAHHBBBBBHHAHBHHHBAHHBBBBBBHHABABHHHHHBHHHHHBH

*001395_00186467 AHHBA-BAAAA--A-HBHBBHHHBA-BBBA-AABAHB-H--BBBBHHHAHAHB-HBHHA-AAHA-HB-BBBHHHAHHH-AHA--HHHAHHHAA-AAA-A-A--B--ABBHHHAA--BA-BHHHH-AHAH--AB-A-HAHHBHH-

*000756_00248631 BAH-AHAB-AA-AB-HHHABABBHB-HAHHHAA-HABA-H-HHAHHBHAHHHAA--BABAAAAHHHB-AABAHHAA-B--HA-B-HAHBBAAAHHHB-HAABHHBAAH-AHHBBHB-BAHH-BAHHHAHABHBBH-H-HHABAA

*000788_00177094 HAABHAHAAHHBAH-AHB-BBHH-H-AAAAHHAHBAB-HB-AAHHBHHHHHHHHBAHAHBHABHABBABH---BHAAHHAA-AHABHAA-H-AAABBAHBHBAHAA-HHBBHBHAB-AAHBAHBBHBH-AABBHHHH-A-HBHA

*001545_00277447 AAAHABHHAHH-BBAHAHHAHHBB-AHHHAABBBB-HHAHBH-HBHAH-ABBBHBAHBBHBBBBAHA--ABAAHBBBB-BB-AHAHHHHAAAH-HAA-HHH---HAA-HHBAHHHHHABBBBAB-HBABHHHAHH-A-HAAABH

*000485_00057798 HHBHHBAHB-BH-BABHHAABBBAAAABAHHHBHABBAHHHHABABHAAHBBHHBHHA--HHHA-ABBAHAHBAAHHBBAHA-H-BBAHABHB-HHAHHHHABHAAHBABBHBHHAABBHHBHBHHHHH--ABH-BBBBHBAHB

*000930_00057843 BHHBAHBBBBAAHAHHHHHAAA-ABBHABHAHHA-HHHAHHBBAAAHHBABAHHBBAHHAHHHHHHAH-AAABHA-AHAAAAAA-H-A-HHHBABABHHAHAHHHH-BAAAHAAB-BAAHHHA-BBHABAHAABBHHAHH-BBA

*000051_00468507 HHHB-HBBHBAABHBHHHHAAABABBBABHAH--BHHHAHBBBA-AHHBHBAHHBBAHHBHBBHHHAHB-AHBHAB-H--ABAABHH-BHBHBABABHHABABHBH-BAABHAABHBAHHHHA-BBHAHHHABBHBHHBAHHHA

*000330_00265126 HBHABAHHAABHHH-BBAAHBHBBBAAHBHBBAAHBABBHBHABHBHAHAAABA--HHAABBBAHHHB-HA-HBBHHHBHAAHA-AHAHHAHH-AHBAAHHB-ABA-HAHABBHHAHAA-HH-BAHHHA-BHBAHAHHHBHBHA

*000206_00257490 HHABBHBHHBH-BAAHBHHHAHHBBBAAB-HHHAHAHABHBAABBHBAHAHHBBAAHHAAHHBHHAHAHAHHBHHHHH-HHBAH-BAAAABHA-HABHBAAHBABABAHAAAHBAHHBAABBHAAAAHHBBHAHHBHBHHAAB-

*001082_00160111 HHABBHHHHBH-BAAHBHAHAHHBB-AABHAHHHHAHABH-HABBHBAAAHHBBBHHHAAHHBHHAHAHAAHBHHH-HAHHBAH-BAAAABAABAABHBHAHBABHBHHAHAHBAHHBBABBHAAAAHH-BAAAHAHBHHAABB

*002347_00072805 BAHAAAAAABAHAHHA-B-BHBHHH-ABHHHAHBHHHBHHAH-BHABBAHAHBABHBAAHAABHAHBA-BHAB-AAAHHHHABHABHBBHAHHABHHBBHHHAHBA-AABHBHHHHAAAABBBHAHHHHHBBBHBABBABHAHB

*001559_00044306 ABHBHBBHBHAB-HABBHAAHAHAAAABBBHHABHHAAHHBAHBABHBHBBAHHHAAHHBHBBAAHHHAAHAAHBHBAHHBBAH-AHBHABHBAHBH-BAAB-AH-AHBHAHHHHHBH-HHHAH-HAHH-AABHBBAAAAHAAB

*002311_00014147 AAHHABHHHAA-BBBAAABAAHHBABBHBHHAHBAHAHBHAAHHBHHABABHHAHBHBAAABHHHBBHBH-HBHBH-HBHBAAAABBAHHAHBHHBHHBBHAHHAA-AAAHBHBA-HBBHBBHA-HAAH-AHAHA-AAHHBHAH

*000053_00211163 BBBHHBHAHHBHBHHBBHHHAHABAAAHAABAABBAAHHHABHBBBABBAAAABHBBHHBAHBHAHBB-HHAAABA-HABHAAHBHHHBBBHHAHHHABHHHHAHHBBHBHHAAHAHABHABBAHHAAABHBHABAAAHAHHHH

*000053_00701201 BBBHABHAHHBABHABBHHAAHABAAAHAAHAABBAAHHHABBBBBABBAAAABHHBHHHAHBHAHBBBHAAHABAHHABHAAHBHHHBBBHA-HHHABHHHHAHHHBHBHHAAHAHHBBABBAHHAAABHBHABAA-HHHHHH

*001175_00030708 HHHHAHAHH-A--B--BABBHHHA--HBHABAH-BHA--ABHA-H-BHAAABAAHHBBBAAHHBHAB--A-A-BAHAHAAAA-H-AA-BBA-HAHHB--HABHHHH-BBAAHHAH-A-HAHB-HBBAB-BHHH-H-HHA---H-

*000139_00566087 -BB-B-BABHH-B----HHB-HBBB-ABHAHBBHBH-HBB--BHBHHH--HHH-ABH-HBHHHBHHHH-H-BBBBB-A-HHBBH-BB-ABHHH-HHBHBB-HHHBB-BHH-ABBBBBB---BB-BBBBB-H-BBB-A-BBBHHB

*000050_00000473 -BABABBBHBAABA-AH-HBHABAB-HABHHHBABAHBAB-H-AAAHHBHBAHH--HBHAHHBHHHHBBHAABHABAH-A-HBH-HBAHAHHB-HAHHHAHA-HBB-BAAAHAAHA-ABHHHHH-BAHB--ABBAH-HHBBBH-

*003063_00003442 BAHHBHHHBBH--BHBBBHBB-BAB-BHAAAB-BHBB-AAABBHABHH-AHHHHHAHHHHB-BBHABB-A-BBAHA-AHHAABH-HHBBAABHHBAABBHAABABA-HHBHBHBAAA-HHHHHHHHBBBHBHHHB-H-B--BH-

*003349_00001501 BBAHBHBHHHBAAA-BHB-AA-HBA-BH-HBHABABHH--HAHBBBBAAABBBAH-HAHHHBHB-HHH-H-A-HH-AHBABHBHHAHHB-AHBB-AHHAAHAABAH-HABAHBHH-BBH-HAHA-HBHAAABHAAHHAAAAHBB

*000358_00022196 BAHAAHAAAAB-BHBBBHHHHHBAHABAHAAABHHBHHBB-H-BHBBAAHHHHHA-BHHBABHHHHBHAA--AAHH-BBAHABB-ABAAHHBB-HHAHHBBHAHBA-BABABHAHAAAAHHHBHHAHAHHBBHB-HH-A-BA-B

*000874_00133115 BAHAAHAAAAB-BHHB-HBHHHBHHABAHAAHBAHBHH-B-HABHBBAAHHHHH-ABHHBHHHHHHBH-AHAAHHHBB-AAABB-AB-AAHBBHHHAHHBBBAHBH--ABABHAHBAAHA--BHBAHAHB-BHHABHBAABAAB

*003104_00094298 ABHABHHBBHHBHH-BHA-AABHHABHBBBHHABHAAHBA-ABHHBHHBAHHH-BHHAHHHBHHBAAH-B-BBABHBHHABHBA-HBBHAAHB-BBH-BBAH-HBBBHAAHBBBAHHAB-HH-A-AAH-HBHHA-BHABBBHBB

*000014_00939755 A-AHAAAHBHAHAH-HBBHHHBABHHBBABHBHAHBHHAAHHBH-HHHAAAHABHAHBB-BAHHBAAHHHAHBAHAHBAAAA-AABBA-HBBAAABAHHAABBHHHBAAHHAABA-AAAAHHHHBHHAAHHA-AHAH-HB-HHA

*001124_00018685 BAHAAHAHHHA-AHAABAABAB-HHHHAHHHAAHAA-ABHBHHAAABHAHHHAAAHBABAAHHHHABBAABAHHAA-BHAHAABAHAA-BAHA-HHBBHAABHHBHAHHAAHHBH-ABHAHHBAABHHHBBHA-HAH-HAAHAA

*000218_00151703 BBBHHBHAAABABHHBBBBAAHHBA-AHAAHHABBAAHHHHBABBBABBAAAABHHBHHHHHBHABAB-HHAHABABHABHA-HBBHHHBBHAAHHAABHHBHAHHHBHBHHAHHAAABBABBAHHAHAB-BHAHAAHHAHHHH

*000427_00457337 HBHHAAHHBBHHAB-HABABB-HHA-BBAHBHHBHHAABHBBAHAABHHHBBHBHAHBHAHHHAHAHAHABHBHHHAAHAHHHHBAHBHHHABAHAAHHBAHAHABAHABBABBAA-BABBBHBBBBHHBBHHABBHBHHAAAB

*004773_00065369 AHA-AABAAHHAHAA-BABBHAABAHBBBAHAHHAAHBAHHHA-BBHHAHABHHHBAAAAAHHHHHABBHBAHHAAHAHAHHBHHHHHBAHHAAAAABHHAHAHHABAHHBAAHHHBAABABAHHAAABH-HBBHHBBAAHHHA

*000389_00158909 AABHABHHABH-AHHBHBHHBHBHAHAHHBBHAAHABHHAHB-AHHBAHAAABHHHHAAHHBBBAAAHBBHBAHHBABHBHHHBBAHHABHBBAHBABBBBH-HHA-BHHABAHHHHABBHAHBBAAABB-BHBBBHAHHHABB

*000214_00366161 B-HBAHAHH-A-HHHABA-BAHBAH-HAHHBAHA-AB-BHBHAHHABHAHHHAAAHB-BAABHH-HBB-A-AAB-HA-HAH--B-H-----BAABABBHAA--HB--BBAAHBHH--BHHH-BA-BHBH--HH-A-H-A-AHA-

*001887_00102795 BBHHA-HHBAH--B-AAAAHH-BHHBBHAH-H-B-AB--B-H-BHABHHHHAH---ABBA-HABHAHA-AABB-HABABAH--H-BBHHBHHB-HAA-BBAAAHB--H-BHABHA--BB-HB---HBHHA-HHAH-B-BBHHA-

*000823_00019896 HHBHHHAHBBB-AB-BHH-ABBBA--AH-HHHHHABB-AB-HABABHHAHBBHAA-HAHBHHHAHABBAHA-BA-H-BBAHA-HABBAHA-HH-BHAHH-HA-HA-HB-BBHBAHA-BH-HBHB-BHHBAAABHA-B-H-BAHH

*002172_00078058 HHHHABAH-HA--HAB-HHHBH-BAAHABHBHHABHHAAABHAHHAHH-AHAAAB-BHHHABBHHABB-H--AHAH-HA-AHAHHAHBHBH-B-HAHBHHBHAHAB-BBAABAAB-ABBAAABB-ABHHBHHA--BH-AAHHBA

*000034_00333296 AAHAABAHAHHBBHAABHBBBH-HH-HAAHHA---HBAHBHH-HAHBHAHHABAHHHHBAAAHBHBBB--AABHHA-HAHH--H--A--A-AABBHBAABBBBAAHHHHHHH-AA-AB-AB-H-BHH-AHAAHAAABABAHHAH

*008460_00007836 BBHHBHAHB-HABB-BHBAHA-HBA-HHHABAABHHHHABHB-HBBHAAABBBAHAAABHABHBHBHA-B-ABHHHABHAHBHBHAABHHABB-HAH-H--H-BHH-B-BAHHHHBA--HBHA-HBBHA-HBBH--A-AAAHBB

*000064_00278384 HHHBABBHAHAAHAAHHBBBHAH-H-BHHAHABHAHBH-HBH-HBHHHAHAAHBH-HHAAAHHBHABB-B-AHBAHBA-ABHBHHBBAHHAHA-AAAAAHAHABBB-HBAHHAHHABHBBH-HH-AHA-BBABAAAB-HB-HAA

*000172_00382521 HHBHBBBHH-BBBHABHHBHBA-HH-HHHBHHAAABAA-BB-BAHHBHAHBAHHABHA-HHBHBBHHH-BBBBHHHABHABH---BHBHBHAABHHHBH-HHBAHA-B-HBBBHABBABHBA-BBBBB-AHBBABHBBAHHBHH

*000550_00169816 -BBHAHHHHHBBBHB-B-BBAHHBABHBBA-BBBHAABBH-H-ABHHAHAHHBHBBAHHBB-HHAHHH-HAAABHHAB-BHHBA-HA-A-H-HHBAHBAAAH-HAHBABHHHAHHABH-HHHH--HAAHBAABBAABABAABBB

*001370_00216784 HHBBBABAHHA--BBHBH-HHHBAA-BHBHBABHBHAHHAAHBHHHBHBHBBHHABHBHAAAHBBABAAAAHHBABBHHBHAHHH--BHHAABBHHABBAAHABBHHHAA-HHBABBAAHABHH-BHH-HBBBAAAH-HHHHBB

*000103_00714638 HAHBAABAA-H-AB-BHBBBBBBHBBHBHHABBBBHA-HBAAAHHAAHBAHHBBHBBBHHBHHHAABHAABHAAHB-AHBBBHA-AHHBAAAHHHAHHBHBHHBHH-HAAAHAHH---A-HHBBAHHHBHHHBAHAABBBHAHH

*000741_00172508 HBAHHBBBAA--HABBHHHHBBHHBHBBAHHHABBBHBABHBBAHAAHHHAHHAAAHBHHHBHHABAA-H-HBHBH-HBHBABHHAHHAHBBABBHHHHA-AHAAA-BBHBBHHAHBBBAHBHB-BHB-A-HABABHBH-ABBA

*001102_00057589 BHHBHBBHBBHBBAHBABHABHABBBBHHHBBBAHABHABBBBHBHBHHHBBAHAHAHHHBHBHHABBHHBABABHAAHHBBBHHAHBHBAHBBBHHABHBHABABAHHHHBBHHBBHBBBHABBHBBHHABHAHBHHAAHBHH

*000115_00155056 ABHAHHABBHHHHHHAHABAABHHHBHBBBHHHBHAHHHAAAHBHBHBBAHHHAHAHAHAHBHBBAHHABHHBABBBHHABBHAHHBBHAAHHHBBBABBHHAHBBBBAAHBBHAB-ABAHHH-HAABBBBHBAHBHABBBABB

*000416_00221232 HHHHBHAHABBBBAAAHHHBBHHBA-AHHAABHABHAAABBAABBHHAAHAHHHAABBAHBHABABAABBBHHHBHABBABAABBHHAHBBHHBHBAHBAHHHBBBHBBAHBHHHHAHHHBBBBBHHAH-BHHHHHBAHBHHBH

*000787_00210744 BAHHAHAHHAABHHHABAABAABHHHHHHHHAHHBABABH-HAHHAHBAHHHAAAHBABAAHHHBHBBAABA-BAHAB-AHAHBAAAHBBABHAHHBBHAABHHAHABBAHHHHHBABHAHABABBHBHBHBBBHBHBAHAHAA

*000851_00281994 HAAAHHHHBBAB-BABHBABAHHHBAHAHABHAAAHHHHBBHHHHAAHHHAAHBBBHHAAHHBAHABHAHABHAAHAAAHBABHBAAHBAHHHBHAAAHBAAAAA-HAAAHAHAAAHHHBB-HAHBBBHAAHAHABHAHAAHHH

*000088_00248943 BHHAAAHAA-AHAHBHBBBBHBAHHBABHHAAHBHHH-BH-BBBHABBAHAABABHHAAHAABHHHBA-BHHBBAHHHHHAABHABHBBHAHHABHAHHHHH-HBAAAHBHBAHHHBAABBBHH-BAHHABBBHBAB-AHHABB

*000696_00051328 HBHH--HHBBHBABBHABABBHHHHHBBAHBHBBAHAAHHHBAHBABHBHBBBBBHHBH-HBHAHHBAAABHAHHBAAH-B-BHBAHBHHB-BAHBAHBBHBAHABBHHBBABHA-HB-BHBHBHHBHHBAHHABBHHHBBAAB

*000019_00886938 AHABHABAAHH-BAHHBHBBHHHAABA-HHAHHBHHBBHBAAAHAHHAHBAHHHA-HBHBHAHBAHBABHHABABAHAAHHAHHHBAHHBBAAAHHAABAHAABB--HHAHHHABH-AAHHAHH-BAHA-BHABAAHABBAAAA

*001441_00111857 -BHAHBABBHH-HH-AA-HAHAHHHBHBB-HHABBAHHHAAABBHBHBHHHHHA-AHAHAHHHBBHHAABHABABBBHHABHHA-ABHHHHHHB-BBABBHHAHB--HAA-A-HABAAHHHHAA-AABBBBABBBBHABBB-BB

*000404_00150963 ABHBHHBBBHABAH-BBAAAHAHAAHAABBHH-BHHAA-HBA-BAH-B-BBAHBBAAHHBHBBAAHH--AH-AHBHBA-HB--H-AHHH-BHBAHBHBBAHB-AHB-H-HAHHHHABHHHHHAHAAAHA-HABHBHABAAHAA-

*001204_00118896 -BABABBABBH--HAAAHHAAHHHB-HHHHHBABBHH-BB-B-BABHAHAA-AHABH-HABAHHABHABHHAABAA-AHHBBHBBHBAHHHAH-AAABHAHBHHAH-HHHAAHABBBHB-A-HABHAHA-AAH-A-HAH-BHH-

*000887_00103464 BBHHBHABBBH--BABHBAHH-ABHBHHBABAABAH-B-HHB-ABBAAAABBHHH-HBBAAHHBHBHB-B-HBHHH-BAAHHHB--ABHHHBHB-AAAHAHHABBH-HABAHHHA---HB-HHHABB--ABHB-A-HHAH-BBB

*000242_00641888 H-AAHA-B-BA-ABA-B-ABAHBH--HHHHHHAAAHH-BB-HBH-AHAHHABHHB-HHAAHABAHABHHHB-HAAH-HHBAAAH-BAHBHAHH-HAAAH-HH-AB----AAAHABAHHABBBH--BABHAAHHBA-H-BBA-B-

*000380_00250057 -BA-H-BBAA--AABBB-BHBBHHH--HAHHHAHBBHB-B-BBAHA-AHHA-BHA-H-HHH-AABHAAB--HHBHA-HHABA-HH-HHAHBBA-BH-BBAHH-AAB-BBHBBHB---H-AH-HB-HHB-A-HAHABHB-BHBB-

*000840_00212039 ABB-BBHABHA-A---HA-H--AB-BBHAHABBBHHHA-H-HHBBAHBHBHAABHAHHA-AA-BAHB--BHHHHHH-AAHH--B--ABBHHHH-BHAAHH-A-HAA-ABHBHHBH--A-BH-A--AHHHBABA-BAB-H-BAA-

*000103_00680813 HAHBAABAAHHABBHHBBHBBBBHBBHBHHHBBBBHAABBAAAHHAAHHAHHBBHBBBHHBHHHAABHBABHAAHBBAHBBBHAHAHHBAAAHHHAHHBHBHHBBHBHAAAHAHHBBBAHHHBBAHHHBBHHBAHHABBBHAHB

*008107_00007994 HBBHBBBABHBHHHBBBHHBHHAHAHBAAABAHHHAAABHABHBHBABBHAAABHBBBHBAHBHHABBHABABHBABHHHHAAHBBBABBHHHAHHHAHHHHHAHHABHBHAAHHABHBHABBHHHHAABHBAABAHAAABHAH

*003230_00026689 AAHAAHBHAAHABAAHHHBBHHHBA-BBBAHAAAAAAHBAAHHBBBHHAHAHHHHBAAAAAAHHHHAB-HBAHHA--HAAH-BHHHHHBHHAHAAAABAAABAHHAHHBHBAHHAABAHBABAAHAHAHAHABBHHB-AAHHHA

*001838_00180804 AHAHAHBHAHHAAA-HHHBBH-ABAHBBB-HAHHAAHBHABB-BBBBHAHAHHHHBAAAAAHHHHHABBHBAHHAAAAHAHHBH-HHHBBHHHAAAABH-AH-HHABHAHBAAHBHBAABABABB-AAA-AHBBAH-HAAHHHA

*000033_00179598 AAHH-AHHHHAABHHAHBHBHHABBAHHHABBAB--AHHBHHBA-HHAHBAHBAAHHABABHBAHAAHAAABAAA--HA-H-AABA-A-HAHAHHHHHAAHABHAHBHHHHABBA-HBAABAHABBHBBHAHAAHAHHHABBAB

*000024_00253767 BHHAHHBAHBHHAHAAAAHAHBAA-BAAAHAHBHHHHB-BAA-HAHHBHHBHHAABABHAHAAAHHHA-HAHHHBB-HBBB-AHAHAA--HAAHAAABHABHHBH--AAHHBBBA--BBBBHHHBHAAB-HBA-BBHAHHHABB

*000806_00105556 AABHHAHHBBH-AB-HBBHBHHB-B-BBAABBBBHBBHAAHBHBABBAHHHHHHH-HHHHBBBBHAHBAAAHHABHAAHHAABA-HBBBAABHBBABBBHAABAB--HHB-BHBABA-BHHHHHAHHHHH-HH---A-BBHBH-

*000756_00253090 BAHBA-AB-HABAHBBBH-BABBHBAHAHHHAB-HABA-H-HBAHHBHAHHHAAA-BA-AABHHHHBB-A-AHHAHBB-AHA-B--AHBBAHA-HHBBHAABHHBH-AHAHHHBHB-BBBH-BABHHH-HBAB-HAHHHHABAA

*001951_00001358 A-HAHHABBHB-AH-AAHHAHHHHH-HBBB-HHBBAHB-AAA-BHBHBBAHHAHHABAHABHHBBHHAABAHBABH-HHAB-BA-ABAHHHHHHBBBABB-HAHB--HAAHHBHAB-A-H-B----A--BB-BHBBHHBBB---

*000498_00101068 ABAAAHAHHABA-HH-BBHHHHHBBHB-ABH-ABHBHBAB-ABABHHBAHHHAB-AHBHBHHABA-BA-HH-HB-AAHA-H-BAHBHAH-HBABHB--BHA-HHA--BHH-HAHABAHBAHA-HBHBAA-AAH-B-A-HA-HB-

*000268_00280169 HBBHBBHA-HBHBHHBBHHHHHABAHAHAAHAABBAAABHAHHBBBABBHAAABHBBHHBAHBHAHBBBABAHHBABHABHAAH-BHABBBHHAHHHAHHHHHAHHABHBHHAAHAHABHABBHHHAAABHBAABAHAAAHHAH

*000749_00054964 HBBHBHBHBAHHHAHBHHABHHAHAHBAAABAHHAAAABH-BHBHBABBHAAABHBBBHBAHBHAABHHABHBHBABH-BHAAHBBBABBHHHHHHHAHHHHHAHAABHBHAAHAABHHHABBHHHHAABHBAABAHAHABBAB

*000752_00073091 HAHHAHAHHHABABAABAHBHHHHHAHBHAHAHABHHABHBHAHHAHHAAABAABHBBBAAHHHAABBBABAHBAHAHHABAHA-AABBBAHHAHABBHAABHHHH-BBAAHHHBHABBAHHBHBBABHBHHHBBBHAAHAHHA

*000768_00238130 HBABBHBABHH-BBBHBHBHHBBHABBHBHBABHBHAHHAAH-HHBBHBHHBHHABHBHAAHHABABA-AAHHBAHBHBBHBAA-AHBHHHAHBHHABBHHBABBHBAAAAHHHABHA-HABHH-HHA-ABBBAAHABBAHHBH

*000496_00394601 HHHAAHAHAAA-HBBABAHBHBHHH-HBHAHAHABHHA-HBHAHHAHHAAABAA-ABBBAAHHHHABBBABABBAH-HAAHAHB-AABBBAHHABHBBHBAB-HHHBBBAAHHHH-ABHAHABHHBABA-HHH-H-HAAHAHHA

*000205_00182160 HHAAAB-HA--HBHBAAAAAHHAB-HABHHAHAAHHB-BHBB-BHHAABBAHHBHHAHBHHAAHAAHBAH-BHHHHHABBAB-HBBBHHHHBBABAAAHBAH-AA--HBHHAHHHA-BBHAAAA-AHBB-AHHAA-B-BHHBHH

*000372_00360098 HBABHABBABBHHH-HAA-BBHAABABBHBHHHHHBHHHB-A-HAH-BBBBBHHBAHHHHHAABHHHBAB--BAABAH-BABBBHAHHABBBBAHBHAHABBHHAH--HABHHHB-A-ABHB-B-ABHBAAHA-HHB-H-HHBH

*000887_00001560 -BHHBHAH-HH-BB-BHBHAHAABBA-HBABAABHHH-AHBBBABBAAAABBHHH-HBBAAHHBHBHH-B-ABHHHABHAH-HB-HABH-HBHB-BAAH-HHABBBABAB-HHHHB-H--BHHHBBHHHABHBAAAHBAAABBH

*004289_00033532 BBHABAAHHAH-BH-HBAABBB-H---HB-B-B-HBABB-HHA-HHBAHAAAHA-BH-A-BBBAAHHABBHAHBBH-HAAA-AABBAAHHAHH-HHHAAHHBBHA-AHAH-HBHHAAHABHHHBABHAAH-HB--ABHHBABHH

*000050_00107413 B-AB-BBBHBAAHA-AHAHB-A-ABAHABHHHBABAHHAHHHBAAAAHBHBAHHB-HBH-HHB-A-H--HAABHA-AHAAHA-HHA-A-HHH--AAHHHAAAAHBHHBAAAHAA---AB-HHHHBBAHB-BA-BA-HHHB--A-

*000012_00125057 BAHBABBA-AB-BAHBBHHAAHAB--HBHAH-AB-AAAA-HB-HBHBHHABABAAABHHBBHBAABAH-H-AHHBH-HABBA-H--A-HBHHAAA--HHHHBHHH-A-HHAH-H-ABABBABBA-HAHH--HHAH-HHA--HAB

*001848_00016390 ABHAH-ABB-H-HH--A--AH-HA--BBBBHHA-BAHHHAAAHBHBHBHHA-H-H-HAHAHHHBBHHA-BHABABHBHHAB-HABABHH-AHA-BBB-B-HH--BBBH-AHHBHABAA-HHHAA-AABA-BHB-BBHHBBBAB-

*002485_00001177 BBAHBHBHH-B--AHB-B-A--HBAAB-B-BBABABHH-AHHHBBBBAA-BHB-H-H-HHH-H-BHHHA--AHHHH-H-AB-BBHAH-HBAHBBAAHAAAHHABAB-BABHHHA--BHH-HAAAAH-H--ABBA-HH-AAAHB-

*000184_00649381 -BHBHHBHBBABBBBHBHBAHAHAAAABBBHHHBHHAAHHBAABABHBBBBAHHHAAHHBHBBAAHHHAAAAAHBHBAHHBBAHHAHBHABHHAHBHBHAAB-AHBB-BHABHHBBB-HHHBAHAHHHHHHBBHBBA-HAHAAB

*001545_00277693 AHAHHBHHAHHBBB-HABBABHBBBAHHHAABBBBBHHAH-H-HBHAHBABBBHBAHBBHBBBBABABBABAAHBBBBHBBBBHAHHHHAABH-HAAHHHHBHB-A-HBHBAHHH-HABHBBHBHHBBBBHHAHH-HHHA-ABH

*000497_00338343 BBHBBHHHHAB-BAHBBBAAA-ABA-BHHBBAHBABHAAH-HHBBHBAAABHAHHAHA-HHHHBHAHHABB-AHHH-HBABHBA-AHAHBAHBBAAHHAABHABA--BABHHAHHHHHHHB-H-AHBB-BABHAB-HBAAHBHB

*001850_00012508 HAAAAHHHBBAHAB-BHBABHBHHB-HAAABHAAAHBHHBBHHHHAAHHHAAHBBBHAAAHHBHHABB-HHBAAAH-A-ABABH-BAHBAHHH-HAAAB-A-AAAA-A-HHABAAAHBHBBHAH-BHBH-AHA-HHHBHAAHHH

*001044_00103525 ABAHHBBBAAA-HABBHHHHBBH--ABHAHHHAHBB-BABA-BAHAAABHAHBHAABHHHBBHAHHAA---HHBHA-HHAB--HHAHHAHBBABBH-BBAHH-AAH-BBHBB-BA--H-AH-HB-HHB--AHAHA-H-B-HBBA

*000670_00114788 ABHHHBHAAHHAHHHHBHBBBAHBBBBAAAHHABAHBBBBBBBBBBBBBHHHBHBHAHHAHBABHHBH-BHHHAHAABAAABBBHBHABHHHHBBHHHHHHBHHAAHBBHHBAAABHBBBBBABBBBBABABBBH-HAHHHHHH

*000601_00161503 HBABAHBABBHBABHHBHHAH-HAAHHBBBBHB-HHAAHH-BABABHBBBBHHHHAAHABHBBAAHHB-AHAAHHABAHHBAAAHAHBAHBHBHBB-BHAHBHAHBHBBHABHH-BBBAHBBAHAHHHHHHBBHBBAHHAHAAA

*000102_00721586 BBBHBHBH-HB-HAHHBHABHHAHA-BAAABAHHAAHABHABHBHBABBHAAABHBBBHBAHBHHHB-BA-HBHBABHHBH-AB-BBABBHBHHHBHAHHHAHAH-ABHBHAHHHABHA-ABBHHHHAABABAAB-H-BABHAH

*000147_00362281 AHHAHHAHAHA-ABBHABBHAABB-BAHBABHAAHBA-HBAHHAHHHBAAHHBBH-ABAHHBBHHBHB-H-AHBHB-HABBBBH-HHAA-HAH-ABAAB-HAHHHAAABHBABBBBAABBHHAAHHAHBHHHBHHBBBBHHAHH

*000017_00606983 BBBAHHB---BABHBA-BBBAHHBA-HBBABBBBHAH-BHHBAABHHAHAAHHABBHHHBBAHHHHH--HA-HBHHHBABB-BA-AABAAHHHBBAHBHAABHHAH-ABHHHAHHHBHHAHHA-AH-ABAAABHAHB-BA-BBH

*002297_00042338 AAHHHBHHB-B-ABABH-ABHBH-H-HAAHABABHHBHHA-BHHHHAH-AAAHBBBAHHHBBBBBABB-H-BAAAHAAHABABHBHHHHAAHHBA-A-BBAABAAA-HABHABHA--HHBHHAB-H-BHAAHAAHAB-BHHHHH

*000127_00618136 HHHBHAAAAAHAAHA-HHHBABHHA-A-HB-HAHBAAHAAH--HHHH-HABHB--BAHHHHABBHBHH-H-HAABB-HBAAABHAHAAAHABB--BAABHHB-H-A-ABH-ABBAA-HAHBHHABAAHB-BBHAHBBAHHAHHA

*000166_00499134 B-HBHBHAH-B--AABBH-AAHHBA-ABHAAHHBHAAH-BABAHHHBBHABA--AHBHBBHHBAABAH--H-HHBH-H-BHA-HBHABHBBHAAHBAHHHHB-AH-BB-HBBAHBAHABBABBABBAHH-AHHAHAH-HAAHB-

*006674_00008443 H-AHAAHAB-HHHH-A-BABHHH-BHH-AABAAAAABAAAA-HAHHBHAAAHAAHHBHH-AAHB-HHA-ABHBHBH-BHHBHAA-AA-HBHHBBBBHBHHBA-HBB-BBAABBHHBHAH-A-BHBBHB-ABBH-HBH-B-HBHH

*001521_00042222 B-ABAHAH-AHBAA-AHAAAB-HHAH--HHAAHBHHHB-BHABAHAABAHHH-BA-H-HBA-HHHBHB-AA--HAAAAHHHB-H-AAHH-B-HAAHHABHBAHAHH--BABBHA-A-ABBAABB-AAHAB-HBA--HHHBAHHB

*000017_00239681 BBB-HH-HHAB-HHBA-BBBABBBA-HBBA-BBBHAH-BH-HAABHHAHAHHHABBHHHBBAHHHHHH-BAAHBHH-BABBB-A-AABAAHHH-BAH-A-ABHHAHB--HHHAHHHB---HH---HAA-H-ABBAAB-BAABBB

*000507_00000766 BHHBHBBHBBBABA-BABHABBABBBBHHBBBBHHABHABBBBHBHBHHHBBAHAHHHHHBHBHHABBHHHABABHAAHHBBBHBAHBHBAHBBBHHHBHBHABAH-HHHHHHHHBBBBBBBHBBHBBBHABHAHBBHAAHBHB

*000566_00076266 AAHHAAB-AHHHBHAHHAABHHBAHHAHAABAHHBAAAAABH-AHHBABHHBHBAABHBHAHBHAABAAHBAAHHHBHBAHBHA-HHAAAHHHAHBAHHHHAHAABBAAHAABBHHBA-ABABHBBHAHAAHHAHHBABHHABB

*000771_00177223 HHHBAHAH-HAB-B-ABAHB-HHHAAHBHA-AHABHHABH-HAABAHH-AABAAH-BBB-AHHHHABB---AHBAHAHHAHA-B-AABBBAHH-HH-BHA-B-HHHAB-A-HH-B--BA-B-BB-BABHB-HHBH---AHAHHA

*000456_00286836 ABAHHH-HBBA--BBABHHHHBHB--AHBBHHBAAHB-A--BABH-BBABAHA--BB--HHBHHHHHAHB-ABBAH-ABHB-BBHHHAHHBHB--HHHBAHBBAH--B-HBA-HA-AHBAH-B-H-BB--H-HB--BAH-AABA

*000043_00439956 HHAHAHBHHBHABAAHBHHHHHHBBBAAHHHA-ABAAHBHAHAB-BBABAHBHBAAHHHAAHBBAHHA-AHHBHHH-HAHA-AABBAA-ABHHHHHHBHAAHBABABAAAHABBA-BBHABBAABHHHAHBHHHABHBBHAAHB

*000014_00467297 ABHHHAHAHHA-AHBBBBHHHBABH-BHABHBHHHBHHAA-H-HBHHBAAAHHB-AHBBBBAAHHHAHHHAHBHHA-HAAAA-AABBAHHBBHHABAHBAABBHHHBHAAHAABAHAAAAHHHABHHAAHAAB-B-AAHBBABA

*000231_00429141 BAHHAHAAHAHABHABBH-H-BBBHABAHAAABHHHHHBHHHHHBBBAA-HHBHAABHBHABHHHHBH-A-AAAHBABBAHBBBBHBAABHHBHHHAHHBBHAHBA-BAHABHAHAAAHAAHBH-AHAB-BBHBAAHHAABAHB

*002981_00017286 ABHHHHAAAHHAHHAHBHHBBAAHBBBAAABHABABBAABBHBHHABHAHHHBHB-AHBAHBABBHBH-AHHHAHAABAAAB-H-BHBBHHAH-BHBHHBHBHHAA-HBHABAAABHHHAB-ABBABBAHAHHAB-B-AHHHHH

*000350_00068835 B-HAAAAHHHBB-H--A-BBABHA--AHAHBBHABA-BB--B-HAHBBBABHAHABHBHBBBHBHHH--H-BAHBHBA-BA--B-AB-HHBAB--BHAAB-B-BAH-H-H-HAHA--B-HHAH-ABH-HH-HH-B-H-HH-HBB

*000391_00340891 HHBBHBAABBHBAHHHBBAAHHAAHHHHAAHHHH-HBHHB-A-AABABAHBHBHHBHHBBHHAAHBBBAB-ABAAHABBHHABH-BAHAHAHH-AHHHAHBABHAA-BABBHBAAAA-H-HHAA-BAH-BAHHA-ABHABAHHH

*001716_00097069 HHAAABBBHABH-HBBAAHAHBH-BBBAAAHBH-HHBHBBABBHHAAHBHHAHBHBABBABHAHBAHH--ABBHHB-AHHBHBHHB-HHB--BABAAH--HAAAB---BHHAHHHA-BBAAAAHAHBHBHBHHH--BABBHHHH

*003206_00034514 BBAHBHAHHHB-HH-BHBHH-HBBA-BAHABHABABHHAAAHABBBBAAABBBAHAA-HHA-HBHHHBAH-ABAHH-H--BBBHHAHHHBABBBHAA-HAHHABAH-HABHHHHBHHHHBBAAAAB-B--ABB-AAH-HAAHB-

*000478_00075945 BBHA-AAHAABHBH-BBAAHBHBH-AAABHBBHAHBA--HBAABHBHAHAAABAA-H-AABBBAHHH-BHHHHBBHBHAHAAAA-AHAHHAHHBA-BAAHHBBHB-AH-HHBBHHAH-AHH-HB-HHHHABBB-BAH-HBHBB-

*000387_00353139 H-ABBHBAH-HB-BBAB-BHHBBHA-B-BHAABHBH-BAAAH-HAB-H-HHBHHABHBHA-HHABAB-HAAHHBAH-H-BHH-HBAHBHHAAHBHHABBAHBAB-H-AAAAHHHA-BHHBABHH-BAABH-BB-A-A-HAHHBB

*000349_00416853 B-ABA-BAA-H-HBHHHHHB-BBA-BHBHH-BBBBHA--B-A-HAAAH-AHHBBABBHHHBHHHAABH-ABHAABHBAHB-B-AB-AHBAAAH-BHBBHHBHHBHH-HAHAHAHHB-HHAHABBAHHAABABB-HBA-B-HAH-

*000930_00122931 BHHBAHBBHBA-HAHHHHHAAABABBHABHAHHABHHHAHBBBAAAHHBABAHHBBAHHHHHHHAHABAAAABHABAHAAAHAABHBAHHBHBABABHHAHAHHHH-BAAAHAABBBABHHHAABBHABHHAHBH-H-HABBBA

*004112_00035142 BHHAABHAHBAAAABHBBHBBHH-HBAHHHAAA-H-ABBHHBH---BB-HHHBABHHAABAHHHBHAAAB-BBAABABHHAAH-HBHBB---BABBH-A-B-H-H---AA-HAH-AHHB-HHHH--A-BBHHBABHH-ABH-AB

*000481_00112914 -AHBABBA--B-BA--BAHAAHHBHAHBHAHBHB-AAAAB-BHHBHBHAABABHHHB-HBBHB-AHA--H-AAHBH-BABB--HHHAHABHHAAHA--HHHB-B--A-AHBHHHHA-A--AB-A-HAHHBBHHABAHAH-AHA-

*007344_00025846 BBHBHBHH-AAH-H-ABHBAABHBBBBHBB-H--HHHABA-H-HBA-B--HBHBHHAHBHHABAHABB-AH-BAAH-HHA--BBH-HABB-AABHHA-BH-AHAH---BAHAHHAB-B-B-BAA-HBBHBHBBHH-BBAHHHBB

*000220_00379735 AHHBABBHABH--HHHAH-BHBBHHHA--ABAHHBHA-HBBHAAHHBAHHBBABH-BHBHAABHAAB--A---HHH-HBAH-HAB-HAAA--A-HBA-AHH-AAAB-AHHAABB-B-A--BABH-BHAH---HHH-B-B-HAB-

*000449_00109044 BHHB-HABHHBAAHHHBHBHAHBBB-AAAHHAA-HABAHHAA-AHBAHABBHABH-BABAAHHHABAB-A-AH-BH-BAHBH-BABBHHBHAHHHABHAABBABBHBHBHBAHBHBA-HHHBBAB-HHH-BAHA-HBBHBHHHA

*002594_00007648 ABHBHHBHHAA-AABBHA-AAAHHA-AHBA-BAABHAHAHBHHHHHABH-BAHBABAHBABAHAABHH-H-AAABBHBAHBH-H-AHHAHHAHHABB-BHHH-AH--HHHAHHAHABH-AHBAA-BHABBAAHAAABBA-HHAB

*000782_00224581 ABHAHBAA--AH-B-AHBBHABH-BBAH-AHBAAAHAHAHBHBABAABAABHBBH-AHHHBBBHHHHBAAB-BBHB-HAHH--B-AHAHHAAAAAAAABBAAHH-AAHBHHAHHHHHHBBHBAA-HBBH-HHB-HBHBHHHAHB

*001333_00001119 --BBHHAABBB--H---A-ABAH---AHAHBHAHHBBAH-HA-AABHHAHBH--HHHHBBHAH-AAB--H-HBAAHAB-BHA-H-HAAA--HH-HHABH--A-HA---AB-HBAA--BHBHBHB-BABH-AHB-HHB-A-AAAA

*000578_00382492 BBBAAHBHHHB--HBABBBBAHHBA-HBBAHBB-HAH-BHBHBABBHAHAHHHHB-HHH-BHHAAHHA-HAABBHH-BABBH-A-HABAAHHHH-AH-AAAHHHAH-ABHBHABHB-HHHHA-AA-AAH-AAABA-BABAAB-B

*001949_00056357 HHABABBAHHHBHAAHAHBBAHAHHABHHHHAAHAABABBHHHBHBBHHAAHHHHBAAHAAABHABAABHBAAAAHAAHHHH-BBHHABHHHHHAAABHBABBHAAHHBHHAHHHBBABBAAAABAAAHBAHBAHABHAHHHBA

*003623_00034281 --HBHHBHBAA-HHB-BHHAHAAAA-AHBBBBABHHA-AAHAABAHHBBBBA-BH-ABHBBBBAAHHA-AH-AHBH-H-H--AA-AAH-HBAH-HBHBBAHBHAHBBHAHAHHHHH-HA-HHA--BH-H-BAB-BHA--HAAAB

*005739_00003602 BB-A-AHAAAB-BHBBBA-HBH--BAAABHBBHAHBAH-H-HABHBHAHAAABAA-HAAABBBAHHH---HHHBBB-HHHA-HA-HHAHHAHA-AHBAAHABBHBB-HAHABBHH-HHAAA-BBAHHH-HBHBAH-H-H--B-A

*000486_00166398 HHHBB-AHA-BHBAAAHHAHB-H-AA--HH-BHABBAAAB-AAABHHAAAAHHBAHBHAHBH-HHHAA-B--BHBB-BBABB-BHHHAHHBHH--HAHB-HABBBBHHHAHB-HB--AH-BBBBBHHAB-BAHHH-B-HBHHBB

*002398_00086912 -BHA-AHHH-H-BBBBBAABBB-HBAAHBHBBB--BAHBHHHAH-HHAHAAAHAABHHA-BBBAHBHA-BHHHBB--HA-H-AAHB---B-HB-HHHAABBBBHAABHAHHHBB--HHAHHBB-AHHAHHAH-AAAH-BA-BA-

*000158_00192575 -ABABAHBBHB-ABABHB-HAH-AHB-BHHHHHAHHHB-B-H-BAAHHHBBAAH--B-HA--BA-HHB-HBHAAHBA-H-H--B-A-AH-HHA-HAAABAHABBH-A-BB-HBBHA-BAHBABAHBHAB-HHAB--H-AHHHBB

*003360_00020084 AHBBB-AAB-H-HAHH-BA-HHAA--HBAAHH-HBHBHBBAA-AABBB-HBHBHHBHH-BHBA-HBB--BAABAAHABBHA-BHH-AAAHAHHAABHHAHHA-H---HABBHBAA-ABHHHHHA--AH--ABHHH-B-A--HA-

*004526_00000782 --HBHAABH-BAAB--HA-A-BAA-AHBH-HHHH-BAHH-HAA-AHHBBBBHHHBAHHHAHAABHHAH--BHHHAHAHA-H--BAAB-AB-B--A-HHHABBHAHHA-AABHHH--AAA-ABHBAABH--AH-A-AA-HABABA

*000302_00465564 --HBBHABAB---A-A-BAHBAH-A-AHHA-BHAHBAA-B-H-ABH-AAAAHHBA-BHAHB-AHHAAH---H-HBB-BAABH-BHHHAHBAAHBA-AHBAHHBBB--H-AHBHH--AH--BBBB-HHABA-HHHH-B-HA-H-H

*000186_00189555 HBAH-BHA-BA---BABB-HABHBBHHHB-HHAAHHHA-H-HHABAAB-AH-A-HAA-BHBHBHHH---A--BHAH-HBAAA-B-AHAB-HAA-HBAABHAABHHA--BHHAHB---B-BHA-ABABB-HHHBA-BA-H-HH-H

*000842_00322112 AHHBABAHAHHA-HBABBBBB-HHHBHAAHHABHBHBAHBHHBHHHBBAHBABAHBHHBAAHHBHBBBABAABHHAHHAHHBBA-HHBAAHAHBBHBAHBBBBAAHBHHHBHBHABABBABAHBBBHBAAAHHA-HBABHHHHH

*002951_00030191 -AHHAAHAHBA-AABHBBHBHHH-ABAHHAAAAHAHHBBAAHAHBA-HHAHH-AHAHAA-AHHABAAAHAHH-AAHAB-AAAHHHHHBBHAHBAABBAAABHBBHAHAHHAHHAAA-AAHHAAHHAABAAHABAAHH-AHHAHB

*000120_00315688 ABHBAHBBAHHB-H-HHHABHHBBHBAHAABAH-BAAA-HBHAAHHBABHHBHBAABHBBAHBHAABA-HB-AHHH-HHAHBBA-BHAAAHHHBBBABHHHAHAABHABHAABBHHBAHABABHHBHA-A-HHBHHBABHHABB

*002831_00023499 HAHHAHHHBBAHAB-BHB-BBBHHH-HAAHABA-HHB-HAHB-HAHAHBAAAHBBHAHHHBBBBBABB-H-B-AAA-AHAB--B-HHHH-AHH-HAA-BBAA-A--AHABHABAAAH---HHAH--BBH-AHA-HABBHHAH-B

*000586_00090443 BBHBHBHBAHB-BB-BHBHB-BBAA-AHHAHHH-HHHAHA-H-AAA-HHHABHBB-ABHBAAAHHAABHH-H-ABH-A-HB--BHA-ABBH-H-BAHBABHBHHA--HBHBABA-B-ABHHBHBHHABA--BHBB-H-ABHAA-

*000821_00004406 HBAABAHHAABBBH--BAAHBHBABAAHBHBBHAHBABBA-HA-HBHAHAAABA-BHAAABBBAHHHHBHHHH-BH-HAHAAAAHHHAHHAHHBA-BAA-HBBHBH-BAHHBBHAA-HAA-HABAHHHHBBHBAHAH-H-HBHA

*002654_00045213 HAHBAAH-A-A--H-A-A-BB-H-B-HHHABBAHHBAB-HAHBABBHAHHHHB-AABHBABHHHBA-H-A-BABAH-HABHA-H-HBBAAHAA-H-B-HAAB-HHBBH-BHHHHA-B-A-HAHB-BAB--HBHHA-H-H-BBAB

*001838_00183029 A-AHAHBAABB--AAHBH-BHHA-A-B--ABAHHAAH-HHBH-B-BHHAAAHHBH-AAA-AH-HHHAB--BAHHAHB-BABABHHHHBBB-HHA-AABHHAHAHH----HB-AH-BBA-BABAHH-AAHB--BB--B-A-HHHA

*001319_00109844 BBBABAHHBAHABBBBBAABBBBABAAABHBBBHHBHHBBAHAHAHHAHHAAHAABHHAABBBAHBHAABHHHBBHBHAAHA-AHBAHBBAHHBHBHAAHHBBHAABHAHHHBHHAHHAHHHHBAHHAHHAABAHAHBHHABAH

*000046_00460158 AABHABHHABHAAHHBHBHHBHBHAHAHHBBHAHHABHHAHBBAHHBAHAAABHH-HAAHHBBBAAAHBBABAHHBABHBHHBBBABHABHBBAHBABBBBAAHHHABHHAHAHHH-ABBAAHBBAAHBBHBHHBBHAHHBABH

*000270_00066903 -HAAHHAHBBABAB-BHBABA-HH-A-HHABHAHAHA-BB-HBHHAHHHAAAHB--HHAAHABAHABBHHH-HAAH-AAHBABH-AAHBAHHHABAHAHBB-AAAABAAAHAHABAAH-BBB-A-BHBH-AHAHA-HBHH-HHH

*000548_00364929 BBHBHBBHBBBHBAHBABHABH-BBBBHHBBBBH-ABHABBBBHBHBHHHBBAHAHHHHBBHBHHABBBBHABABH-AHHBBBHHABB-B-HBBBHHHBHBHABAB-HHHHHHHBBBBBBBHHBBHBBHHABHAHBBHAAHBHB

*000260_00061557 --ABABB-H-H--A--AHH-AAAHH--HA-HAAHB-H-BB-BH-HB-HHAAHHHHBAAHAAAHAABAA-A--AHAHAA--H-BB-HHABAHBH-A-ABHHAB-HAA-B-H-AHHH-BA--A-AAB--AHBAHH-BHB-AHHHBA

*000148_00293003 A-HAABAHA-HBAHHBHHHHBHBHAHHAHHBHBA-ABAHAHBAHHABHBAAABHAHHAA-HBBBAAAH--BBAHAB-HHBHAHHBAH--BBBB-HHABBBBHAHHHBBBAABAH--AABHHABBBAAHBAHHHHABHAHAHHBA

*000338_00083331 HHAAAHBHHBHABHABHHABHAHBH-AAAH-A-BBAA-BH-HABBBBABHABHHAAHAHHAHBBAHHAAABHBHHBHHA-HBHABBBAAAHHHBHHHBHAAA-AB--AHHAAHBAA-BHAHBHHAHHABABHHHABB-BHAAHB

*000629_00109955 HAABAHHHA-H-AHAHHBABBAAAH-BHBHAAHBABHHBBHAAHAAHBAHHBABABHHHHABHHHHAH-HBABAHAHABABBBB-B-HHHBBHBABHABHBA-ABH-BBAHHAAHAHH-HAHHBAHABA-BAHA-BHBABHHHB

*001593_00146499 BBBHAHBHBHBHBHBAABBBAHHBABHBBAHBBHHAHB-HBAAABBHA-AHHHHBBHBH-BHHHAHHHBH--BBHH-BABHH-A-BA-AAHBAHBABBAAAHHHAHHABHAHAHHH-BAHAHHAHHAAHBAAHBAABAB-ABBB

*007733_00002128 ABHHAABHBBHHAB-AHBABHBAH-BBBAABHABHHAAHHHBAHHABHBHBAHB--ABHAHAHAHHHA-AHHAHHHAAHHHHHBBABHABBHH-BHAHBBHHAHABBHHBAABHAA--ABHHHBAHBHA---BAB-AABABAAB

*006619_00006291 --HHAAAH-HAB-B-ABAHBH-HH-AHBHABAHABHBABH-HAHHAHHAAABAAHBBBBAAHHHAABBHA-A-BAHAHHAHA-HHAABBBAHHAAHBBH-ABHHHBAB-AAHHHHBAB-AHBBHHBABA-BHHBB-H-AHAHHA

*000102_00487672 HBBHBHBABHBH-AHHBHABHHAHAHBAAABAHBAAAABHABHBHBABBHAAABHBBBHBAHBHHABB-ABHBHBH-HHBHAAHBBBABBHHHBHHHAHHHHHAHHABHBHAAHHABHHHABBHBHHAABHBAABAHAAABHAB

*000019_00504401 AHABHABH-H--BAAHBH-BHHH--BAB-H-H-BHB---B-AAHAHHA-BAHA-ABBHHHHAHBAHBH-H-ABABA-AAHA-HHHB---BBAAAH---B-HHABB--HHAAHHABH-HABH-HB-BABABBHA-A-H-BHAAHA

*001921_00059123 H-AHAAHH-BH-ABBHAB-BBHAAH-BBAHBH-BHHA-HHBBABHABHBHBBH-HHHBAAHHHAHAHA-A--AHHH-ABABBHAB-HHBHBAB-BBABBBHH-AAB-HHBBABHA-HBABBBHBH-BHH-AHHABAH-HBHAA-

*001541_00007783 AHHHABABAHH-BHHABBBBBHBHHBHAAHHABHBHB-HBBHBHBHBH-HHABABHHHBAAHHBHBBBBBA-BHBAHH-BHBBHBHHBBAH-BBBHBAHBBBBAAHHHHBHHBAAB-BBABHHBBHBB-BABHAAHBBBBHHHH

*000069_00625116 HBHHBBBABHBHHHHBBHHBHHAHAHBAAABAHHHAAABHABHBHBABBHAAABHBBHHBAHBHHABBHABABHBABHABHAAHBBBABBBHHAHHHAHHHHHAHHABHBHAAAHABHBHABBHHHAAABHBAABAHAAABHAH

*000104_00349209 AHHHHBHAAHBHHHHHBBBBBHHBBHBHAHHHAHAHBBBBBBBBBBBBBHHHBBBAHHHHHBAHHHBHHBHHHAHAAHAAHBBBHBHABBHHHBHBBHBBBBHBBBBBBBBBBBBBHBBBBBHBBBBBABBBBBHHAAHHHHHA

*000104_00415706 AHHHHBHAAHBHHHHHBBBBBHHBBHBHAHHHAHAHBBBBBBBBBBBBBHHHBBBAHHHHHBAHHHBHHBHHHAHAAHAAHBBBHBHABBHHHBHBBHBBBBHBBBBBBBBBBBBBHBBBBBHBBBBBABBBBBHHAAHHHHHA

*000268_00033877 HBHHBBBABHBHBHHBBHHHHHABAHHHAAHAAHBAAABHAHHBBBABBHAAABHBBHHBAHBHAABBBABAHHBABHABHAAHBBBABBBHHAHHHAHHHHHAHHABHBHHAAHABABHHBBHHHAAABHBAABAHAAABHAH

*000727_00156631 BAHAAHAAAAHABHABBHAHHABBHABAHAAABHHBHHBBHHHHBBBAAHHHBHAABHBHABHHHHBHHABAAAHBBBBAHBHBAHBAABHHBHHHAHHBBHAHBAABABABHAHAAAHAAHBHHAHABHBBHHAHHBAABAHB

*000765_00104081 BHHA-HHAABABAHBHBBBHHBBHHBABHHHABBBHABBHHBBBHABBAHAABABHHAAHAABHHHBAHBHHBBAB-HBBH-BHABHB-HAHHABHAHAHHHBHBAHAHBHBAHBBHAHHBBHHAHAHHHBBBBBABBAHBABB

*000563_00236051 AHABHABHAHHBBABABHBBH-HAABABHHHHHBHHBBHBAAAAAHHAHBAHHHABHBHBHAHBAHBHHHHABABA-AAAAAAHBBAHHBBAAAHHAAHAHAABBB-HBAHHHABB-AAHHHHH-BAHHBBHHHAAHABHAAAA

*000777_00064137 -HHHABHB--ABBBHAAHBAAHHBHHBHBHAAA-AHAHBH-AHHBHBA-ABHHAH-HBHAABHHHBB--H-H-H-HBH-HBH-AABBBHHAHBHHBHHBBAAAAAHBBAAHBBBAH-H-HBBHA-HAA-AAHAAABAAHBBAAA

*007853_00001226 H-HBHAABABB--HAAAAHBBHA-B-HBHBHHHBABAH--HA-AAHHBBBBBHH-ABH-HHAABHHHAABB-BHAB-HABHBBBAAHHABBBB-HBBAHABBHAHHAAHABHHHA-H-ABHBHBAABHBAH-H-B-HHHAHHBH

*000005_01115568 HABBABHAHBHBHHAHBB-BBHHBBBAHAAHHA-BHHH-BAAHABBBHBAHAHHB-BABBHHA-ABBBBBAB-BHA-HHHAHAH--AHHBHBAAAH-HAHBBBHAAHHAHBHBH--HB-HBHA-HHBHAB-BHHH-B-ABABA-

*001201_00040615 BHHHHAAHH-A-AAB-ABAHHHA-HBAHAAHAAABBBB-AAAHHHHHBHHBABBA-BABHAHBHHHBH-AHHHBBBBBBHHABBBAAAA-HHA-HHH-BBHA-HB--HBABBBHABAHAABABHAHHBB-HHAHB-B-H-HHAB

*007650_00007513 BHBHBAHHHBH-BAHHAHAHAHHAA-HAHBABBHHHHBBH-BBBAAAHAHHAHAAAHBAHBHB-BHHB-B-A-HABBBABHBHH-AHHAHABBHABBABAHHABHHBBHBBAHBHBHBAHBHHABHBABBBABAAHHHABAHB-

*001688_00107822 HBHABAHABAHBAAAHAAHHHHBBH-AHBBHB--BAABAHBBBBBHAHAABBAAA-HAABBHAHHHHHAHAHHHHBAAHAAAAB-HBBHBHBA-AHHBABHHHABB-HHHBHBBH-BHAHHHHABBAABBBAB-HHHHHH-BBB

*007397_00012773 B-HABBABABHA-H-HHHHBB-H-AAHBAHBHHHHAH--BHBHBHHAAHHAHBAABBAAAHBHHHBHB-BBBB-BBAHBBHABB-H-HHBH-B-AAH-B-HHAHB---BBHH-AABABBHB-BHABHA-AABHA-BB-AHAHHB

*002021_00221141 BB-BBBBB--B-BB-HHHHBHHH-BBBBBBBAABAHHBHH-H-BHAHBHBBBBB-BBBAH-ABHAHHABHHHBBAH-AAHH--H-BHBBAAHH-BAABHBBHBH-A-AHBHBBHH--ABAHHBH-BH---HBHHBBB-ABHAHB

*003006_00014692 A--BAHBBAHH-HBAAHBBBHHBHA-BBHHABBABHH-AB-BBBHHHHAAAABHHAABAHBAAABHB----ABAAB-HBHH--BH-HBBHHAAHHHBHHB-HBH---HBBBHBHA--ABABHHHABAABBHHHBH---B-HAHB

*000232_00396732 HHA--AHHB-B--A-HBH-AAHHBB-AABH-A-HHAHA-HBHABBHBA-AHHB-HHAHAA-BBH-AH--AH-BHHH-H---B-H-BAAHABHA--ABHBB-HBABH-HAAHHHBAHA-AABBHAAAAH--BAA-A-H-HHAAB-

*000002_00763940 --BAAAHHAHH-AB-HHB---BAH-AHHAAHABBHHA--B-BAHHA-AAHAAABH-HHH--AHAAHAAB---HABH-AHHH--BHABAH--HH-BHHBBHAB-HAH-AABHABHA--BA-H-HA-HAH-AAAB---A-BB-AAB

*001176_00121048 --BHHBBB-BBBBB-H-H-BHBAHBBBBBBBBHHABB-AAHB-HHHBHAHAHH-ABAABBBBBBBBBHHH-HBHHAAHHHBB-A-BHBHBHAH-HHHBHHHBBAAA-HHHHBHBABBHB-H-ABABBBAAAHBAB-BBHHHBHH

*000642_00233347 AHBBAHAABBB--H-HBHAAHHHA-AHHAAHHH--HBHHBAAAAABHBAHBHHHHBHHBBHHHAHBBBAHAABAAHABBHBABAAAAAAHAHAAAHAHAHBAHHAH-BABBHBAAAABBHHHHHBBAHHBAHHAHHBAABAAHH

*000493_00056647 AH-BAH-B-HH-BBAAABBHHABBABBBBH-BHABH-H-BHBBHHHHHAAAABHH-HBHHBAAABHB-AAA--AHB-HAHAAHB-AHHH-HAAHHAB-HBAHBHH-A-BBBHBHABBAHAH-BB-BAAB-BHHHHAHBHBHABH

*000490_00143500 B-H--HABA-A-HH--BBA-A--HB-HAHHHA--BABABHBH-AHHBHAHHHAAHHBAB-AAHHHHBB-ABAHBA--B--B--BABA--B-AAHHABBAAABHHBHAHAAHH-B--A-AAHBB-HAHHH-BA-BHHHHH-ABAA

*001225_00017957 BHBAAAAAABA--H-HBBHBHBHHH-ABHHHAHBHHABHHBHBBHABBAHAHBABABAAHAABHAHBAABHABBAH-HHHH--H-BHBBHAHHABHHBBBHHAHB-AAHBBBHHHH-AAABBBHAHHHBBBBBHBAB-ABHAHB

*004758_00001918 H--BAAAH-AAA-H-AHHHBBAHBB-HHHABBAH-B-AHHAHBABBAA-BHA-AAHHHBBBHBAAAABAH--AHAH-HABH-AB--BAHAA-AB--HAHAHABHHH-H-BHHAHAAHBAAH-BBBBHHHHHHAHA-H-HABBA-

*002480_00086359 BHHBBBHA-AB-BA-BBH-AABH-AAAHHAA-ABBAA--H-BAHHHABB-B-H-AHBHABHA-HABAHBHHAAHBHAHABHAAHABHHH-BBAAHAAAHH-BHAH--BHBHHHHA-AABBABBABHAH-B-BH-H-H-HAHHH-

*004421_00008213 HBBHBBBABHBH-HHBBHHBHHAHAHBHAABABBAAAABHABHBBBABBHAAABHBBBHBAHBHHABBHABABHBABHHBH-AHBBBABBHHHAHHHAHHHHHAHH-BHBHAAHH-BHBHABBHHHHAABHBAABAHAAHBHAB

*000046_00525034 AABHABHHABHHAH-BHB-HBHBHABAHHBBHAHHABBAAHBBAHHBAHAAABHHHHAAHHBBBAAAHHBBBAHHBABHBHHHB-ABHABHBBAHBABBBBAAHH-ABHHABAHHHHABBHAHB-AAHBBHBHHBBHAHHBABB

*000284_00194893 BHHB-BHH--BA-A-BABAAB-ABBHBHHBBBBHHABHAB-B-HBHBAAHBAAAHAHHHHBHBHHABBHH-ABABHAAHHBH-HHAHBHBAAB-BA-HBHBHABAHAHAHHHAHHBA-BB-HHB-HBBHHABH-HBBAAAHBBA

*001799_00034124 AHHHABAHAHH-BH-ABHBBBAHHH-AAAAHAAHHHBAAB-HBAHHBBAHHABABHAHBAAHHBHBB--BAABHHA-HAHHBBH--HBBAHAA-BHBAHHBBBAAA-HHHAH-AH--HBABBABBHHBAHAHHAA-B-B-HAHA

*002856_00020689 HBHBAAHHBBHAAB-HABABBAHBHBBBA--HHBHHAAHHABA-HABABB-BHBHHHBHAHHHAHAHAHA-AA-HHBAHAHAHH-AAHAAHAB-AH-BBBHHAHAB-H-B-ABHAAHBABBB-B-HBHH-AHHABBBHHBHA-B

*000024_00893786 H-AAHHBAHBH--H--H--AHAAAA-AAAHH-BAHHHB-B-A-HAH-BHHBHHAA-BBBAH-AAAHHAAA-BAH-B-HBBAH-H-HHAH-HAAHAAABHAB-A-HH--AH-B-B-BBHH-BHHBB-AA--HBA-B-H--B-AB-

*000036_00038524 AHHBAHBBA-BA-H-HBHBHBHBAABBBBHHBAAHHHBABABAHAHHHAAAAHBA-BBAHBAAABHBB-H-AHAAA-HHHBHABAAHHHHHAAHHHBAHHHHAHHBAHBABHBHHBBABAHHBBBBAA-BAHHBAABHAHHAH-

*000587_00224874 BAAB-BBBAAAA-AAHHHHBBABABAHHAHHAB-HABHAHBH-AAAAHBHBAHBHBHBHAAHHHBHHB-H-AHHAB-HAH---HHHBAHBABBAH-HHAHHABBBA-BHAHBAAH-BABBHHHBHBAHBBBABBAHB-HHBBHA

*000704_00079311 BAHAAHAAA-H-BHHBBHAHHABBHABAHAAABHHBHBBHHA-HBBBAAHHHBHAABHBHABHHHHBB-AB-AAHB-BBAHBHBHHBAABHHBHHHA-HBBHAHB--BABABHAHAAH-AAABHAAHABHBBHHA-H-A-BAH-

*000796_00139869 HHHH--HH--HHAHH-AB-H--AB--H-AAHA-ABBBHBABABHHHHBHHBAB--ABHBHAHBBAHBH---BBBAB-B--AH--BAH-A-ABBHH-AAB--HBHHB-HBAABBAAH-HA-HABH-HHBBBBAA-BAB-HHHHA-

*001434_00197479 HBAH-AHH-BHA-BAAABA--H-HHABBAHBA---HAAHHHBAH-ABHBHBBHBAHHBA-A-HAHAAAAABHHHH--AH-A--HBA---HAA-AAHAH--HHAHAB-HHBBA-HA-AHABB-A-AHBHA-AH-A--HHA--AA-

*002882_00062913 BBBBBHBBBBBBBBBBBBBBABBBBBBBBBBHABABHBAAHHHBBBBAABBBBBBBBBHHHBHBHHHHBHHAHHHHAHBABBBHBAHHHBAHBBAAHHHAHHBBAHAHABHHHHHHHHHHHABAAHBHHHABHAABHHAAHHBB

*000729_00137965 ABAAHHBBA-B--B--AA-HBHAAAA-HHHBB-HHABHBHAH-HAHHB-HHHAHABH--HBAHHHBHB-BB-HHBB-HBBA--BAHHAAABAABAAAAAHBABAAB-BABHBHAABB---BHHB-HAA---HBH--HHHHHHB-

*001703_00201404 BHHAAHAAABAH-HBHBBHBH-BHBH-B-H-AH-HHHBBHHHB-HABBAHAABA-HBAAHAABHAHBAHB--BBAHAHHHBABH-BHBBBAHH-BAHBBHHHAHBABAAB-BHHBAAAHAB-BHAHHHH--BBAB-B-ABHAHB

*000843_00129378 AHBBHAAABBH--HBHBBAAHHAAHAHHAABHBHBHHBHBAA-AABBBAHBHBH-BHHBBHHAAHBBBAB-ABAAHABBHHABBHHAHH-AHHAAHHHAHHABHAAHHABBHBAA--BHHHHAH-BAHBBAHH-BAB-ABAHBH

*001909_00051736 BHHBAHBBHBAA-A-HHHAAAABAB-AABHAHAABHHHAH-B-AAAHHBABAHABBAHAHHHHHAHA-BAAA-HAHAHAAAHAABHHAHHBHB-BAB-HABA-HH--BAABHAAHB-AHBHHAABBHAHHBAHBBHH-BHHBH-

*000507_00231874 BHHBHBBBBBB-BA-B-BHABHABBBBHHBBBBHHABHABBBBHBHBHAHBBAHABHHHHBHBHHABB-HHABABH-ABHBBBHHAHB--AHB-BHHHBBBHAB-HAHHHBHHHHB-BBBBHHBBHBB--ABHAHBB-A-HBH-

*000342_00214940 BHBAHAHHHBA-HAB-AHAHAHBHHHAAHHABHHHABHBH-BABHAAHAHAAHAAAHBAHBHBBBHHBABAAHHAB-BBBHAABHHBBAHABBHABBHBAAH-BHB-HHBBAHBABHBAHH-HAAHBA-BHABAABBHABBHHH

*000994_00095459 BBBBBBHBBBHBBBBBBBHBBHHHBHHHABBBHHBHBBBBHBBBHBBBBBBHBBHBHHBBHHBHHBBBBBH-BBHHHBBHHBBBBHBHBHHHBBA-BBH-BHBHBAHHBBBH-HHBHBB-B-HHBBHBB--HHHHHB-ABBBBB

*000693_00114652 AHHHHBHAAHBHHHHHBBBBBHHBBHBHAHHHAHAHBBBBBBBBBBBBBHHHBBBHHHHHHBAHHABHBBHHHAHAAHAAABBBHBHABBHHHBHBBHBBBBHBBBBBBBBBBBBBHBBBBBHBBBBBABBBBBHHHAHHHHHA

*001757_00086321 AHAHHBHAAHBHHHHHBHBHBHHBBHBHAHHHAHHBBBBBBBBBBBBBBHHHBBHAHHHHHBAHHHBHHBHHHABAAHAAHHBBHBHABBHBABHBBHBBBBHBBBBBBBBBBBBBHBBBBBHBBBBBABBBBBHHAAHAHHHA

*001984_00063702 HBBHBBBABHBHHHHBBHHBHHAHAHBAAABAHHHAAABHABHBHBABBHAAABHBBBHBAHBHHABBHABABHBABHHBHAAHBBBABBBHHAHHHAHHHHHAHHABHBHAAAHABABHABBHHHHAABHBAABAHAAABHAH

*002135_00077600 H--BHBBH-BHBBHHH-BHHHHBHBBBBBHBBBBBHBB-BBH-BHBBHBABBBHBBBHBB--HBHBB-BBBA-HBB-BBH-BBBHBABB-BBHHHBBBHBBHBHHB-HHB-HBHHH-H-HBHH---B-HBHAHHB-BBBBHBBB

*000017_00245283 -BBAHHBHABBAHH--HBBBA-H-A-H-BABBBB-AH--HBH-AB-HABAHHBAB-H-H-BAHH-HHH-H--HBHHHB-BBB--BAABAAAHBHBAH-A-AH-HAA-A-AHHAHHABB-H-HHA-BAAH-AABA--BHB--BBH

*002224_00034576 A--BHABH-HHBBAH-BH-BAH-AA-ABBABBHBHHB-A--AAHAHHAABHHHHHAH-HHBAABAHBAHH-ABABABAAHAHH--BAAH--AH--HA-BAAH-BH----A-HHAH-HH----HA-BABA-HHABAAA--A-AHA

*002783_00083206 BBHA-AHH-ABBBHBBBA-BBH-HB-AHBHBB-A-B--BHBHAB-BHAHAAABAABHHA-B-BAH-HH-BAHHBB--HH----AHH---H-HBBAHBA-HHBBBBH-HAHHBBH---H-BHBHBAHHBHBBHB-H-BBBB-BB-

*000975_00261782 A-H--AHBH-BHABAAAAAA-HAABAHBHBHAHA-BAHHBHAHBAHHBBBBHHH-AHHAAHAABHAHHA-BHHHAH-HA-A--BHA-A-B---AH-HAHA--AAHHAAAABHAA-BAAA-H-H-AABHA-HAHAAAHAHA-H-A

*001324_00119604 HAB-AHBAAAAA-B-HHH-BBBBAB-ABHAABBBBHAAAAAHAHAAAAHHHHBBH-BABHBHHHAAB--AB-AAHH-AAHHBHA-A-HBAAAH--HHHHHHA-HB--HHH-HAHBB-BH-H-BBAHHAHAHAB-A-B-B--AHH

*001235_00064104 AHHBAB-B-AA-AAAHBH--HHHBAABBBAHAHHAHB-HHHABBBHHHAAAABHH-HHAA-A---HBB-B-HHAAH-HBA-A-H--HAAHHBA-A-ABAHABABBA-HBHBHH-H-HABB-HAH-AHAHHBABBAHBAAAHAH-

*004275_00019212 HHHAAAABAAHBHH-BBAHHHAH-AHHA-HBAHABHHA-ABAA-HHHHAAHAAABHBBHHABBHHABB-ABBAHAHAAAHAAAHHAHBHBAHHAH-BBAA-B-HHABB-A-HAAHHA-AAAABHAHBBHHHHAB--H-A--HBA

*000373_00203442 AHHBBAABABBHBAAAHBAHBBHBAHAAHAAHHAHBHHAHHH-ABHHAAAAHHBAABHAHHAAHHHAH-BBHBHBBABHABAAB-AHAHBHHHBABAHBAHHBBBBHHHABBHA-BAAABBBBBBHHABH-HHAH-BHHHHBA-

*000749_00118664 HBBHBBBHBAH-BAHHHHABHAABHHBAAABAHHAAAABHABHBHBABBHAAABHBBBHBAHBHHABH-ABHBHBABHHBHAAH-BBABBHHH-BHHAHHHH-AHAABHBHAAHH-BHHHABBHBHHAA-HBAABAHAHABBAB

*000128_00082836 HBBHBHBABAH--A-A-HABBHHHHHBAAABABHAAAA-HABHHHBABBHAAABABBBHBAHBHHAHHHA--BHHA-HHBBA-B-BBABBHHHAAHHHHHHHHHHA-BHBBAHHBAHHAAABBH-AHA-BHBAAB-B-HABBAB

*000089_00203818 -HBAHA-H-BH--A-HAAABA-HAH-AAHBABHA-HBABHHB-AHAABAHAAHHAAHBAHBHBB-HH--BAHHHBBBBBBH--BBHBBAHAHB-AHHHBAAHABHB-BHB-AHBABHBA-H-HAAABHBBHAH-ABHHA-HHH-

*002032_00085215 -B--AAHABBHH-B-H-A-HHBHAHA--AH-HHBA-HAAH-HABHA-HHAHHHBH-A-BAHAAAHAAAA-BBBAHA-A-A-B-HAHHHAHHABAHHAHBBAHAHAB-HHBBABHAA-BABHBAH-HBHABAHBABBHAB-HAAB

*000342_00288778 BH--HAHH-----ABH-B--AHH-H-BAHHABHHHABHBHHB-BHAAHAHAAHAA-BBAHBHBBBHH--B-AHHAB-BBBHB-HHHBHA-AHB-ABBBB-AHABHB-H-BBAHBA-HB--HHHA-HBABBBABA-BB-ABBBHH

*001003_00379467 BHBA-AAAABA--HHHBBBBHBHHH-ABHHHAHBHHHBHBHHBBHABBAHAHBABBBAAH-ABHAHBAHB-ABBAH-HBHHAB--BHBBHAHHABHHBBBBHAHBABAHBHBHHH-AAAABBBHA-HHHHBBBHBHBBABBAHB

*000411_00319849 AAHBABAHA---BH-ABHB-B-H-HBB-AHHABHBHBA-B-HB-HHBHAHHABAHHHHBA-HBBABBBABAABHHA-HAHH-B--HHBHAHAAB-HBAHAB--AA-BH-HHHBA-BAB-ABHHBBHABH-AAH-A-B-BA-HH-

*000609_00119638 AAHABAH-BBBB-B-B-HH-A-BAB-HBHHHHHAHHHBHBHHBBAAHHBBBAAA--HAHAHABA-HAB-B-BAAHB-BHAA-A--A--HHABA-AHAABHHH-B-H-ABBBAHHH--BAHBAHA--HA-BH-ABH-ABHHHHBB

*000460_00030272 -HABB--BB-HAHH-ABBAHB-HH-HHAHAAHAA---HAHAHAAHH-HAHABHBA-BH--HA-HHHA---BHBHBB-BBHBH-B-AHAHBH-HBABH-B-BABBB--H---BBBAB-BABBBBB-BHAB-BH--BHBHHH-BH-

*000538_00284554 AABABABAHBBAHABAAHABAHBHHHAHHHHHBHBHBHBHHBBHBHHBAAAAHHAABHHBHBHBBHHBBBAHBHBBBHBBHBHHHABB-BAHHHAHHHBHAHABABBHHHBABB-BBBAAAHBABAHBBBAAHABBHHABHHAA

*000420_00496509 H-ABHAHBHBB-ABAH-AAHBHAABAH-HBAHHBHBHH-B-A-BAAABBBBHHHB-HAH--AABAHHHBBBABHAHAH-BHBHBA--HABBBBABBHHAABBHHHHAAHABHHAHAHAABHBBB-ABH-ABHHA-HHAHH-HBA

*001253_00126072 --BABAHB-AH--BBBBAAHB-BHB-A-BHBBHBABABBHBHABBBHAHAAA--A-AHAA-BBAHBHABH-BHBBHHBAHA--A-HHAHBAAHBH-BAAHHBBHAH---H-BBHHAHHAHHHHB-HHH-H-HBAHAH-BBBBH-

*000475_00160706 ABHBAHABAHHABB-AHBBHH-BBABBHHB-BH-BHB-AB-B--HHBHAAAABHB-HHBHBAAABHB--A-AHA-B-AHHHABB--BHHHHAABH-BHB-HH-HAHAHBBBHBH-B-AHABH-H-BAHBBAHA-HBABBH-AH-

*001824_00009951 BB--AAHABBH-AB-BAB-BBHA---BBAHBHHBHHAA-HBBA-HABHBHBBHBH-HBHAHHHAHAB--ABB-HH-BAH-HHAHBAHHHH-ABA-HA-BBHH-HAB-HBBBABH-A-BA-BBA-BHB-BB-HH-B-H-H--A-B

*000461_00161312 B-BB-AB-BHA-BHBHHAAAHHB--ABAHBBHB-B-A-H--H-BHHB-BABBHAABHB-AHHBBBABA-H-HH-AB-H-BH--HH-H-BHHABHHBA-BA--ABBH-A-AAHHHABHAAAABHHHB-HBHBBBHA-BB-H-H-B

*001302_00170998 BBHABBAHA-HB-HAAHHHH-H-AH-HBAHHBAHAAABHBHB-BAHAAAHAHH-A-BAAABBHHHBBH--BBBHB--BBBH--B-HH--B-BBHAAHHBAH--HHH-ABBHHBAB-A-B-BBB-AHHAB-AB----H-AB-HA-

*005849_00004098 HAABABBAHHH-BAAAAHHBAAAAH-BHAHHAAHBHHABBHBHBHBBHHAAHHBHBAAHAAAHHABAABABAAHAHAAAHHABB-HH-BAHHHH-AABHHABAHA-AHHHBAHHHBBABBAAAABAAHHBAHHAHHBHABHHBA

*000320_00232424 H-HAHBAAAAB-BAAHHHHHHHBBH-BAHAAHB-HBAAHBBH-BBBBAAHAHHBAABBHBHHHBHHBH-AHAAHHHBBHAH-HBH-BAA-BBB-AHABBHBBBBBH-HAHHBHABHAA-AHBBHBHAABBBHBAAAH-A-BHHB

*000927_00015097 H-HBHAHB--B--BAHAABBBHAAB-HBBBHHHHHBH-HBAA-BAHHBBBBHH-BHBHHAHAABHBB--BB--HAH-HHBHB-BHABHABB-BA-BHHHABBHHHH-AAABHHHBAH-ABHBBBAABHBA--HAH-BABBBHBB

*000075_00658497 HBHAAHAH-HHABB-AAAAAHABHAHBAAAAAABHABAHHAAABHABHHHHAHBHAA-BAHHABHAHA-AH--HHHAAHHHHHHABHHH-HABAHAAHBBAAAHBHAAHBBA-AAA-BABHBAHAHBAHA-HHAH-BABBHAAB

*002547_00121165 HA-ABHAHB-B-HBB-HABHA-BABBHBABHHH-HHHHHA-HBBAAAHHBB-AHHAHBHAHABABHHB-HBHAAHB-BAAHA-BA-HAHAA-A--HAABBHHBBH-A-HB-HBAHA-BAHBABA-BHA---HABA-HHAH-B--

*000557_00015076 AAHB-HAHABHH-AAAHHAHBHHBA-AHHAABAAHB-AABBAAA-HHAAAAHHBA-BHA-BAAHHHA----HBHB-AB-AB-ABHHH--H-HH-A-AHB-HHBBBB-HHAABHH---HABB-B-BHHABABAA-B-BHHHAHBB

*004926_00004144 AHAHHBHAAHBHHHHHBHBHBHHBBHBHAHHHAHHBBBBBBBBBBBBBBHHHBBHAHHHHHBAHHHBHHBHHHABAAHAAHHBBHBHABBHBABHBBHBBBBHBBBBBBBBBBBBBHBBBBBHBBBBBABBBBBAHAAHAHHHA

*000049_01010057 BBHABAHHAAHABHBBBAABBB-ABAAHBBBBBHHBABBHAHAHHHHAHAAAHAABHHAABBBAAHHA-BAHH-BH-HAAAAAA-BAAHAAHHBHHHAAHABBHAAAAAHHHBHHAHHABHHHHABHAAHAHBAHAHAHBABHH

*001078_00030090 BBHAAHHA-BA-AAB----BHBHBHBABHHAAAB-H-BBABHHHBHBHHHHHBAB-BAABAH-HBHAAA--ABAAH-BHAAA-B-HH-BHAHB-BBHAAABB-H-A-AHHAHHHHA--A-HHHB-BAB--H-B---H-AHAAB-

*001280_00120241 BBB--HAABBH--H--BBAHHHHH-H-AA-BHHHBHHHHB-A-AHBBBB-BABHHBABBBHHAAHBB--B-AB-AAAB-HA--H-HABHBBHAA-HBAABBA-HAA--ABBHBHAA-H-HBH-H-ABA---HHAHHBBB-AHHB

*002607_00010067 BBHABAHHAABBHHBBBA-HBHBHBAAHBHHBH-HBABB-BBABHBHAH-AABAABHHAABBBAHHBH-HHABBBH-HAHAABAAHBAHAAHH-AHBAA-HBBHBBA-AHABBBBAHHAHHBHBAHHHH--HBABAH-HBHBH-

*000838_00199097 AHAB-HHH-BBABA-A-H-HHBHBB-AA-H-HB-HA-ABHBHABBABAAAHHBBBHAHAHHBBAHAHH-A-HBBHH-HHBHBAH-BAAHAHHA-AABABBAHBABH-HHHHHHB--H-HABBAHAAAHAABAHABBB-H-AABB

*000580_00036602 B-BHBAHHBBHBBH-HBAAHAHHAABHAABABBH-AHABHBBH-AAABAHHAAAHAHHHABABHBHHBABH-ABABABHHHBABABHBHAAHHHHBBABABAHBHH-B-BBAHHHB-BAABHBABHBABBHHHAA-H-AHHHBH

*000650_00005973 HBABA-BABBBB-H--AHHHAAAH--HHHHHBAB-HAAB-HB-BHHBA-AABHHABHBHHBAHHABHA-HAAABAA-AB-B-HB-HBA-H--H--HA-H-HBHH---HAHHAAAB--ABBA--A---A--AAHAAAH-H-BHB-

*000709_00082436 HAHBA--A-ABA-A-H-HHAA-HB--H--AHBA-HAAAABBB-HBHBAHA-ABH-HBHH-BHBAAHAB-H-AAHBH-HA-BH-H-AAHABH-HAAAABAHHB-HB-AHAHBAHHB----BABB-AHAHB-BHH-A---HA-HA-

*000704_00065554 BAHAAHAAAAH-BHHBBHAHHHBB-ABAHAAABAHBHABHAHHHBBBAAHHHBHAABHBHABHHHHBHAABAAAHB-BBAH-HB-HBAABHBBHHHAHHBBHAHB-ABABABHAAAAAHAAHBBHAHABH-BHHAHHAAABAHB

*001329_00090658 HHBA-BHA-BA--H-BHBABHAHBHHABHBHAHB-HHBHBH-BBHAABAHAAH--HBAAHAABHAHHA-B-BBBAH-A-BH--HAB-BBAAHB-B-HBBBBHAHBA-AHBHBAHHHH-BAH-B-AHHBH-BBHH-ABBAHHAB-

*004056_00012932 HBHBHAHBA-B-BB-AHAAHBHAABAH-B-AHH-BBHH-BBAHBAHABBBBBHH--BHBAHAABHBHH--BHHBAA-BBBH-HBBABHABBBBAHBBHHABBHHH----A-HHHBHHAAB--B-AABA-AHHH--BH-H-BHBH

*000380_00312800 -BABABB--AAH-A--HHHH-BHHBBBHAHAHAHBBHBABAB-AHAAA-HAHB-A-HBH-HBHAA-A--HH-HBAA-HHAB--H-AHHAAB-AB-BHHBA-HHAAH--BHBBBBAH-AB-HHHB-BAB--AHA-ABH-H--BB-

*000674_00317420 BHHBHBBBBBHBBAHBABHABHABBBBBHHBBBABABBHBBBBHBHBHHHBBAHHHHHHHBBBHBHBBHAAABABHAAHHHB-HHAHBABAHBHBHHABBBHABABHBBHHBHHHBBBBBBHHBBBBBAAABH-HBH-AHHBHH

*002917_00083747 AAHBBHABABBHBAAAHBAHBHHB-AAAHAABHABBAAABHHAABHHAAAAHHBAHBHAHBAAHHHA-BBBHBHBBABHABAABHHHAHBAHHBABAHBAHHBBBBBHHABBHHHB-HBBBBBBBHHA-HBHHH-ABHHHHHBH

*000093_00193499 -H-AHHAAAHB-BA--ABHHHHHBHHBAHA-HHHBBAAABBHABBB-AAHAHHB-ABBHBHHHBABBA-HBA-BHHHBHAHABB-HB-AABBBA-HABBHBBHB-HA--H-BHABHAAB-HBBHBHA-HB-HB-AAH-AABHH-

*000605_00121730 HAAHHHHHBBAH-B-BHBABHBHHBHHAAHAHAAHHB-HBBHBHHAAHAAAAHBBBAHHAHBBHHABBAHHBAAAB-AAABA---AAHHAAHHHHAAABBAA-AAAAAABAABAA--HHBHHAHHBHBH-AHAAHBB-HAAHBH

*000883_00174490 -AABHAABA-AAHAAAHBBBB-AHHBAAHABBAHHHA-HAAH-AHB-AAHHHB-H-HHHABBHHHABH---HAHAHAHA-B-AH-BAHAAAAA--AHABAHBBHB--BHBH-HHA---AHBABB--AHA--AH-A-H-H-A-B-

*002036_00069721 ABBHHBHHHAAB-BB-ABBAHBB--HH-BAHA--HHAHB-AH-ABAHA-HHHBA--ABAAABAAABBA-H-HBHHH-HBBAHAA--AABHAHB--HBHHB-A-HAA-B-HBBHBH--H-HH-AHA-AHH-AHABB-ABH-ABA-

*000961_00318956 BHABHHABABBABHBHBAAHAHBBB-AHHBHAAHBABBHHAAAHHBHAABBHABHHBHBAAHHBHHABAH-AHABBABAHBA-B-BHHHBBHAHAABHAAHBABBH-AAH-AHHHB-HAHHHBABAHHBHBAHAB-BAHBAHHA

*000767_00190087 H-HB-HBA-HA--B-HHHHBBBB-B-ABAAHBBBBHAA-H---HAAAA-HHBBBH-BH--B-BHAABH-A--AAHH-AHHABAA--HHBAAAHBHAH-H-AA-HH--HHHAHH-B-B-H--ABB-AAA-HHAB--HBABB-AA-

*000842_00300005 --BHABA--AHB-A-ABBBBBBHHH-BAAHHABHBHBAHBHHBHHHBH-HHA--H-HHBAAHHBHBBBHBA--HAA-HAABB-A-AABAAHAHBBABAHBBB-AAH-BHH-HBHA-AB-AB-HBB-HBA--HH-ABB-B-HHH-

*000358_00161176 BAHA-BAAA-BA-HHB-H-HH-B-HABAAAAABHHBH--BHHABHBBAAHHHHHAABHBHABHHHHB-AAHAAAHH-BHAHA-B-HBAAAH-BHA-AHHBB-AHBA-BABABBAH-A--AHHBA--H-BABBH---H-AABAH-

*001449_00163369 BBHBAAHBHHAABH-HBHHHABBHBHHAAHAAHHHABABH-A-AHBHHABHHABH-BABAABHAABABAA-AHHBH-BAHBABB-BHBHBAHAHAABHAA-BABBA-HHHHHHBBBHABHH-BA-A-H---ABB-AH-HBHBAA

*006245_00047991 -BH-ABABBHBB-HHAAAH-AA-HBBHBBBHHHBBAHHAAAA-B-BHBHHHAHHH-HAHAHHABBBHAABBAB-B-BHHABHB-BAB-HAHHH-BBBABBHHAHB--HAAAHBHABHAHHHBHHAAABH-BHB-B-HBBBBABB

*002656_00041995 BBHABAHAAABHBHBBBAABBABHBAAHBHBBHAHBA-BBBHABHBHAHAAABA-BHHAABBBAHHH-BHAHH-BBBHHHAAHABHBAHHAHHBAHBAAHHBBHBBAAAH-BBHBABHAHHHHBAHBHHBBHB-HAHBHBHBHA

*000047_00569517 B-HAHHAAAHB-BA-HHH-HAHH-HABAHAAHB--BAHHB-HABHBBAAHAAHBAABB-BHHHB-HBAH---AHHH-B-AHA-BB-B-AABBBAAHA-HHBBBBBH-HAHHBHAB-AA-BHBBHB-HAABBHB-A-HHAABAHB

*007079_00011883 HAHHHBH-BBHBABABBBABHBA-HHHAAH-BABHH-HAA-BHHHHHHAAHAABBAAHH-BBBBAABB-H-BHAHA-AHAHA-BB-BHHAAHH-BAHBBBAABAA-AHHB-HBAA----AHBAH-ABBH--HHB--H--HAHAB

*000056_00671710 HBHBHBHBAA--BH-H-ABHBBHHAABHAHHBAAHBBAAA-BAHHABA-AAHBHAAAHHABBBABHAH-HBBHBHA-ABHBHBH--HBHHBAABHHHHH-H-HA--ABHHHBAB-B--BAH-ABBHH-AA-HBBA-BBHBBBB-

*001886_00066481 H-AHHHHHAHH-BA--BABHA-AB-BAABABHAAHAH-BB-AABBHBAH-HHBBA-HHAAHHBHAAHAAA-HHBHHBHAHHBAH-HAAAAHHHBHABBHAAHBABA-AHABAHB-H-B-ABB-AAA-HBB-HAB-BH-HHAAB-

*000713_00113058 H-ABAB-AHBHB-AAAAH-BAAAAH-BH-HHAA-HHB-B--BHBHB-H-AAHHHH-AAHAAA-HABAA-A--AHAH-AHAH-BBAHHAB-HHH--AA-HBABHHAA-HHH-AHHHBBABBAAA-BAAHB-AHH-B-B-A--HB-

*000036_00408864 AAHBAH---HB--H-ABHBHBHBAA-BBBA--AA-HHH-B-B-AABHH-AAAHHA-BBAHBHA-BHBHBHAAHAAA--HHB--B-AHA----A-BHBHHHHAAHHB-HBABHBHHBBA-AH-BB-BAAA-H-HBBAB-HBHAH-

*000261_00418883 BAHAAHAAHBAAAA-HBBBBHHABH-AHHHHAA-AAABBHABHHBHBHAHHHBABAHAA-AHHAHAAAAAHHBAAHABAHAAABHHA--HABBABBHAAABHHHBAAAHHHBHHAAHAAHHHHHHHAAAABHBABABBAHAA--

*000704_00018888 BAHA-BAAAAHABHHBBHAHBHBBHABAHAAABBBBHHBHHHHHBBBAAHHHBHAABHBHABBHHHBHB-BAAAHBBBB-HBHBBHB--BBHBHHBAHHBBHAHBAABABABHAB-AHB-AHBHHAHABHBBHHAHHBA-BAHB

*000885_00185696 -BABABBABBH-BH-AAHBHAAHAH-HHAAA-AHBH---B-B--HH-AHAAHA-A-HHHHBAHHABHA-HB-ABAH-ABHH--BBHHAB-HAH-AAABHAHB-HAH-B-HAAHAHB-H-BA--ABHAH-BAAAAAHB-A-AHBA

*001506_00205786 BAHAAHAAAAHA-HHBB-AHHBBBHABAHAAABBHBHHBBHHHHBBBAAHHHBHA-BHBHABHHHHBH-ABAAAABBBBAHBHBHHBAABHABBHHAHHBBHAHBAABHBABHAHAAAHAAHBHAAHABHBBHAA-HBAHBAHB

*000135_00224150 HHHHHBABHAHABH-AAB-AABHBB-BHHBABHABAAAH--A-HBHHHAAAHBBBBHHHHBAHHHAHHAABHAAHB-AHHHABBBAHBH-HAAHAHABHBB-HHAH-BBBBHBH-BAABHBAHA-BAB-BABABB-HBAAHAH-

*000548_00347415 BHHBHBBHBBBBBAHBAB-ABHABB-BHHBBBBBHABHABBBBHBHBHHABBAHABHHHHBHBHHABBHH-ABABH-AHHBBBH-AHBHBA-BB--H-BHBHABA-AHHHBHHHBBB--BB-HBBABB-H-BHAHBBHAAHBHH

*000011_00261702 AHHBABBHAAA-AA-HBBABAHHBH-BBBAHABHAHBHHHBHBBBABAAHAHBBH-AHHAAAHHAHBBBB-AHHAH-ABAHA-H--BAA-HHAAAAABAHABABBHHHBH-HHHAA--BBHAABAAAAHH-ABHAHB-HHHHH-

*000123_00368008 AHHBAHBBAHH-HBAABHBABABAA-BBBHHBAAAHAHAB-B--AB-HAAA-HHH-HBABBAAABH-B-A--HAAH-BHH--BBAAHAH-HAAHHHBHHHHHAH-A-BBH-H--ABBA-AHHBB-B-AH-AHH-HAHBHBAAB-

*001628_00250434 --HBHAHB--BHAB-B-AHHBAAABAABABHHHHHBHA-B--ABAA-BB--AH--AHHHAHAABHHHA--BHHAAHHHHBH-HB-ABHA-BB-AABAHHABBHHA--ABABHHHABAA-BHBH-AABHAABAH-HBA-AA-A-H

*001404_00072787 B--B-HHBHAA--BBHBH-B-BBHB-BAHHH-BBBAB--H-AHAAH-HABHHAHH-BAB-AH-HHBHBAA-AHHBH-B-HBA-BABH-B---A-H-BBAAHB-BBBBBAH-HHBBBHB-HHBB-B---HABBBB--H-HBHB--

*002445_00053230 HBHBBHAHABB--AAAHHAHBHHB--AHHHABHAHBAAABHA-ABHHAAAAAHBA-BHAHBAAAHHAH-B-ABHBBABBABHABHHHAHBBHH-AHAHBAHH-BBBHAAAABHHHBAH-BB-BBBHBABHBAHBHABAHB-HHH

*000148_00361028 AABAABAHABHBAH-BHHHHBHB-A-HAHHBHHABABABA-BAAHABHHAAABHHHHAAAHBBBAAAABBBBAHAB-HHBHA-HBAHHABHBBAHHABBBBHAHH--BBAABAHH-HABBH-BBBAAHBHHHHHBBH-HAHHBB

*000426_00055651 HBAAAB-AH-H-ABAH-HAHAHHHA-AHAAAAH-AHBAAA-BHHHBBB-BBBAAH-BHBHAAHHBHBABABHBHBHAB-HHB-AAHHHHHHBBBBHBBHHBA-HBBHBBA-BBBAAHH--AB-AABHB--BB-HAHH-BHHBA-

*000541_00204118 BHHAAHHAHBA-AA-HBBABHAHBABAHBAAAABHHABB--H-HBHBHBHHHB-B-HAABAHHABHAAB--BBAAHABHHAA-BH-HBBBAAB-HBAAA-BH-HHA-AHHBAHHHAHAAHBAHAHHABABH-BA-HH-AA-AH-

*003726_00027552 BBHABBAAAAH--HABHHAAHAB--ABBA-HHHHAAHBHHHB-BBHAAHHAHHAABBAAAHBHHHBHBBBBHBABBAHBBHABBBHBH-BHBBBAAHHBABHAHHH-ABBAHBAA-A-BHBBBH-HBAB--BBAB-H-AHHHHH

*000069_00515197 HBHHBBBABHBHBHHBBHHHHHABAHBAAABAHHHAAABHABHBBBABBHAAABHBBHHBAHBHAABBHABABHBABHABHAAHBBBABBBHHAHHHAHHHH-AHHABHBHHAAHABABHABBHHHAAABHBAABAHAAABHAH

*000779_00222763 HHBBA-AABBH-BHHBBB-HHAA-AAHHAABAAHBHHH-B-ABAHBBH-ABHBHH-HHBB-HAA-BBB-B--BBAAABBHAH-H-HA-HHH-A-AHBHAHBABHAA-A-B-HBHAAABA-BHHH-ABHAB-HHAAHB-HHABHB

*000231_00119293 BAHH-BAABAH-BAAB-HAHBHBBB-B-H-AA-HHHHHBHHHHHBBBA-HH-BHAABBBBABHHHHB--A-AAAHB-BBAH-HB---AA-HHB-H-AHHBBHAHBAABABABHAH-AA-AAHB-HAHABHBBH-A-BBA-BAHB

*000337_00123262 H-BHB-BHBAHHBAHAH-ABHBHHH-BAAABABHAAA-BHABBHHBABBHAAABABBHHBAHBHHAHHHABHBHBABHABBBAA-BBABBHHHAHAHBHHHHBHHAABBBBAAHBA-HHAAHBHAHHA-BHBA---B-HABHAB

*001337_00023590 AHBAAABAHBH--A-AAH-BAABHABAHA-AHHBHHBABAHBAAHHHBAHAAHH-ABBAHHHHBBHHB-BAHBHBBBHBHHBHH-HHHABAHH-AH-HBHABABAB-HHHBAHBABBBAAAHBAHAHBB-AAHABBABABHHAA

*002097_00076678 BBBBBHBBBBBBBBHBBBBBABBBBBBBBBBHABABHBAAHHHBBBBAABBBBBBBBBHHHBHBHHHHBHHAHHHBAHBABABHHAHBBBAHBBAAHHHAHHBBAHAHABHHHHAHBHHHHABAAHBHHHABHAABHHAAHHBB

*003735_00022670 AABHABBHA-A-BB-HABBAHBBBA-HHBHHAHBABA-BBAH-ABAHABAHHBAHBABAAABAHABBA-H-HBH-H-H-HHH-A-B-HB-A-BBHBHHHBHAHHAA-B-H-BBBHHBHBHHBAH-HAH-AAHA-BHA-HA-BAA

*000456_00292796 -BHBHHBH-HABABBHBHHABBBHHBHBBBHHB-BHBBABHBABHHBBABBHABHBBHBHHBHHHHB-HBHABBAH-ABHB-BBHBHAHHBBBHHHHHBABBHABABBBHBAHHAABBBAB-BBABBBH-BHHBB-B-HBHABA

*000253_00304710 BHHBHBAABBB--HBBBBHA-HABA-AHAAAH---A-HHHHBABBHBBBAAAHB-BBHH-HBBBAB-H-HHAHHB--HA----HBBH--BBH---BAAB-BBBAHBBBHBHHHHA--ABBHBB--HAHHB-BHAH-H-B-AHB-

*000645_00104123 BHAB-BBB-BAA-A-HAAHB-A-AB-HHAHHH---A-HAHHHHA-AHHBHBAHB-BHBAAHAHHH-AB--AAHHA--HA-A--AHHB--HAA-AHAHHAHAAHBBA-BAAHBAA---AB-HAH-HBAABBBA--AHBHHB-BAA

*000071_00701276 ABBABABABBB-AAB--HABAHB---AHBHHHAHHHBBBBHB-HHHBBAAAAHAA-BHH-HAHBB-HB--AHBHBB-B-HH--BAHHHABAHA-HHH-BBAH-HA---HH-ABBA----AAABA-AH-B--AH---A-ABHHHA

*000017_00242106 BBBAHHBHHHBAAHBAABBBAHHBA-HBBAB---HABBBHHHAABHHAHAHAHAB-HHA-BAHHHHAH-AAAHBH--BABB--AHAA--AAHH-BAH-AAAHAHAHAABHHHAH---HHAHAAAHAAAAHAABAA-B-BAABBH

*000220_00245188 AHHBAHBH-HH--H-HAHHBHHB-H-ABAABAHBBHAAHHBBAAHABAHHHBAB--BHBHAHBBAABA-BB-AHBH-ABAHBBA-HH-HAHAH-HBAHBHHA-AA-AA-HAABBBBBABABABH-BHAH-AAHHA-BABHHAHB

*002862_00075498 HHHHAA-B-HBBBH-HBBBBH-HAHBHHHBBABHBHBA---HHAHHBHAHBABAH-HBBAAHBBHB-B-H-AHH-AAHAHBHAA-AB-AAAAHABHB-HHBHBABA-HBHAHBBABAHA-BHBBHHBAABBBHHHBB-B-BAAB

*001557_00196852 AAAAHAHHBBA-AB--HBABHBHHB-HAAABHAAABBHAB-H-A-AAHH-AAHBBBHHAAHHBHAABBHHH-AAAHHAAHBABH-HAHBAHHHBBAAABBAA-AAA-AAHAABAAAHHBBB-HH-B-BH--HAHHHH-HAAHHA

*005605_00002190 ABHHABHHAHHABH--BHBB-A-HHBHAAAHAB-HBBAABHHBAAHBHAHHABABHAHB-AAHBHBBH-BAABHH-BHA-B-BHH-H--A-A-BBHBAAHBBBAAAHHHHAHHA--ABBABHABBHHBAAHHBAAHBBBAHA-A

*000445_00043094 AAABBAABBHB--HAAHBABBBAHA-H-HAAH-A-BHHAHHHAABHBH-BAHHBH--H-AAAAHHAA--B-HBHB--BH-B-BB-AHAHBHHH-A-A-BAHHHBBB--HA-BBH-B-HABBBBBBBHAB-BHH-H-B-H-ABH-

*001279_00048901 ABHABHAHB-H-AAHA-H-HHHAB-AABBABHAHBAHA-A-A-BHBAHAA-BH-BAHAAAHHHABBBH-B-AAHBHBHHAHA---HB-H-ABB---HABBABAAH--AHABHBBB-AHH-HBH-AAAB----BHHAH-B-BHH-

*000124_00756191 ABHAHBABBHH-HHAABHBA-HH--BHBBBHHHBHABBAAAAABHB-BHAAHHAAAHAHAHHHBBHHH-BHABABBBHHABHHA-ABHHHAHH-BBBABBAHAH-B--AAAHBHABAHHAHBHA-AABBBBHBAB-HB-BBAB-

*000006_00865002 A-BHAA-HB-A-HB-BHBHHHHBB-BBBABBBHAABH-AAHH-HBHAHHAAHABH-HBBBBAHHBHAH-HAA-HAA-BAAAH-A--BAHBBBB-A-AH-AHH-HHB-A-BHAB-A-BA-AH-HHBBHA-BBABAAAH-H-BHH-

*000095_00114871 AB-BHHBABHAB-B-BBHHAHBH---ABBBHAHBHH--B-BBAB-BBBBBB-BBBAAHBBBBBAAHHB-AAA-HHH-ABHB--HH-HBHBBBH-BBBBH-HB-AHB-BHB-HHH-BBHHHHBAH-HHHBHBBB-BB--H-HAA-

*002337_00127243 HBABAHHAHB---H-HBBABHH-H--HBAAHAA-AABAAAAB-AHHBHAHAHAAH-BABBAAHBHHHA-ABHBHBH-BHHBHHAA-HBBHBAB-BBBBH-BAHHB--BBAABBHH--HHAABBH-BA--AB-H-BHHAB-HBHA

*000033_00533383 AAHHAAHHHAAABBHAHBABAHHBBHHHHABBAA-BAHHBHHBA-AHAHBAHBAAHHABH-ABAHAAHAAABHAA-AAABAAAAHABA-HAHAHHHHHHAHABAAH-HHHHA-BAA-BAABAHABBHBBHAHAAHAHAAA-BA-

*002333_00168866 AHH-A-BBA-H-BHA-BH-BH---H-A--A-AHHBAAAHHBHAABHBABHHBB--ABHBHAHBHAAB----AAHHH-BBAH--ABHHAAA-H---BAHHHHAHAAB-AAH-ABB-ABAHABABBBB-AHA-HH--HB-BHH-B-

*002311_00019448 AHHHABHHHAABBBHHAABAAHABHHBHBHHAABAHAHBHAAHABHHABABHHAABHBAAABHHHBBHHHHHBHB--HBABAAAABBA-HAABAHBHHB-HAHAAHBHAAHBBBA-HHBABBHAAHAAHAAHAAABAAHABHAA

*000813_00187445 HBBABHBHHAHAAA-BABABHAAHB-BAAHBAB--AAAHAAHBHHAABBHAAABAHBHH-AABAHAAH-AHAHHHA-HHHB--HABBA-BAAAHHAAHHHHHAHHAABHBBAAH---H-AAHB-HHHAABHBA--ABAHA-H-A

*001384_00077944 AHBAHABAHBHA-A-A-HABABHHH-ABHHAHABAH-HBH-BBHHHABAHAAA-A-BBAHBHHBBHHB-BAH-BBBBHBHHBAH-HHHABA-BHAHHBBHAHABABBHHABAHBABBBAAA-BA-AH-BBAA-AHBH--BHHH-

*000561_00018644 -BHABBAB--BBAHA-HB-AHHHA--ABAABHH-BAHHHH-B-HAHAHHHAB-A--BA-AHBBHBHA-HB--BABB-H-B-ABBBAHHHBBBB-AAH-BABAAHHH-ABB-AAAB--HB-B-BH-HAABH-HH-H-B-AAHHH-

*001143_00043062 ABBBBBBHH-H--H-BHAHAABH-B--BBABAAH-B-AA--HHBBAHAABAHA-BBAAABAHBBAHAH--ABHAHA-B-HH--B-BHHHBB-HHHBHBBBBH-AB--A-HBHHHHABBB-AHA--AH-A-BHB-H-H-B--BHH

*000422_00232272 A-HAHHAB-HA-HH-AAHHAAHHABBH-BBHHABBAHHHAAA-BABHBAAHHAAA-HAHAHHABBHHAABHBBABHBAHABAHAAABHA-HHH-B-BABBAHAHBB-HAAHHBHA-AAAHAHAHAAABHBBABH--HA--BA-B

*002733_00052891 ABH-HBBHBHABBH-BBAAAHAHAA-AHBBBBABHHAAAABAHBAHHBHBBAHH-AAHHBBBBAAHH-AABAAHBHBHHHBAAHB-BHBHBAHA-BHBBAHBAAH--HHHABAH-H-HHHAHAB-HAHHBHABHBB-BAA-AAB

*007981_00000839 HAH-HBA-HABAAHBAH-HHABAHBBB-HBBHAHBAHH---BBHHHB-BHHHHABBHHHHHABHHBHH-BBAAHBB-HBAAABABHAHAA-B--B-AABHHHAHAAAABHBA-A-AA-BAB-AA-A--BH-BB-HBB-HBABHA

*000836_00229300 BHHBHBBHBBB-BABBABHABHABBBBHHHBBBHHABHABBBBHBHBHHHBBAHAHAHHHBHBHHABBBHBABABHAAHBBBBH-AHBHBAHBBBHHABHBHABAB-HHHHBBHHBBBBBBHABBABBHBABHAHBBHAAHBHH

*003240_00006722 HBHBHBBHB-ABBB--BH---AHAAAAB-BHHABBHAAA--A-BABHBABBAHBHAABHBHBBAAHHHAA-AAABH-AHHBHAHHAHHHAB-B-HBBBBAH-HABBBHBHAHHHHABHHHHBAH-A-HHBHAB---AAA-BAAH

*001041_00064656 HHABABBBHHAABH-BHH-BBABABAAHAHHABABABBA-BH-AAHAH-HBAHBHBHHHAAHHHAABBBHAA-HABAAAAH-BH-HB-H-AHBAAAHHA--HHBBA-BHAHHAHHA-ABBBHHB-HAH-BBABAAHBAHH-BHA

*001986_00070594 AHAAHB-H-HHBBBAAAHHHB-BBBAHHAAABBBBBHHAHBB-HBHAHBABBBHBAHBBHBBBBABAB-A-AA-BBBBHBBAHHAHHAH-AHBA-AAHHHHBHB-A-HHHHABHAAA-BABBA--HB-B--HABHHH-HHHABH

*000235_00151206 A-BB-HA--BHB-HHBBB-AB-AAB-HHAAHHBABHBBHBAA-A-B-B-HBHBAHBHHBBBH-AHBBB-B-ABAAH-BBHHABH-HABAHAHH-AHHHAHH--HA----BBHBAAA-BAHH---B-ABB-AHH-HBB---A-H-

*007078_00012350 BBH-BBAB-HHBAHA-HB-BHBH---BBABBHBHHAHBBAHB--HHAAHHAHBA--B-AAHBHHBBHHHBB-BABBAHBBHABB-HHHHBH-H-AA-BB-AAAH-B---BAHBA-BAB--BB---BBAB-ABH-H-H--B-H-H

*001186_00144688 H-HHAAHHB-A--B--A-ABBBBA-HBB-HBBH-BHAABHHBAHBABH-HBBABH-H-H-H-HAHAH--A-HABHH-AHAB-HA--HBA-HABAB-AHBBAHAHAB-HHBBABHAAAB-BBBHB--BHHBAHH-B---HBAAAB

*005909_00022212 A-AHAAABHAA-HB-ABAHBHAAHA-H-HAAAAABHHA-A-HAHHAHHAAA-AAHABBBAAAH--A--HA-A--AH-HHAH--A-AA---AHH-HHBBHHABAHHAA-BAAHAHAH-BH-H-BHABABA---H-A-HAA-AHAA

*000601_00229554 HBABABBABBH-HB-HBHHAHHA---HBBB-HBBHHAABH-HABABHB-BBAAAHAAHABHBBA-HHH-A-AAHHH-AB-BBAH-AHBA--BH--B-BH-BBBAHB-HBH-HHHA--BABBBAHAHHHH-H-B-BBA-AAHAA-

*006984_00034145 B-HABAHAAAB-AHBBBAAH-HBHBAAABHBBAAHBAHBABHABABAAHAAABAA-HHAABBBAHHHH-H-AHBBHHHHHAAHAAHAAHHAHABAHBAAHHBBHBAAHAHABBAHAHHAHHHAHAHHAAABABAHAHAHBABHA
[truncated: 13,202 more chars]
