# Supplementary material for: Comparative Mapping of the Wild Perennial Glycine latifolia and Soybean (G. max) Reveals Extensive Chromosome Rearrangements in the Genus Glycine
Source: PLoS One. 2014 Jun 17;9(6):e99427. doi: 10.1371/journal.pone.0099427 (PMC4061007; doi:10.1371/journal.pone.0099427)
Supplement: Table S2 — GBS SNP Genotype data for the F5 mapping population. (DOCX) [file pone.0099427.s005.docx]

Table S2. GBS SNP genotype data for F_5_ mapping population

#line 11_12 11_2 11_27 11_3 11_32 11_8 15_14 18_10 18_20 18_2_2 18_3 18_6 20_1 20_15 20_17 20_19 20_24 20_6 20_8 26_13 26_5 27_1 27_15 27_21 27_24 27_26 27_27 27_5 27_6 36_12 36_13 36_14 36_16 36_20 36_3 36_6 36_8 3_10 3_11 3_16 3_18 3_26 3_28 3_32 3_33 3_7 3_8 4_20 4_28 4_35 4_5 4_7 5_11 5_18 5_20 5_22 5_25 5_8 6_12 6_22 6_36 6_5 6_7 7_1 7_10 7_104 7_110 7_111 7_115 7_117 7_119 7_12 7_128 7_129 7_13 7_151 7_160 7_166 7_17 7_184 7_19 7_20 7_21 7_25 7_28 7_29 7_30 7_4 7_5

*000000_00161740 ABBABABABBBABBBB-BAABBAABBABA-AABBBBBBBABAABBBBBAAAABABBBABABBAABABAABBAABABBABABBBBABBAA

*000000_00239253 ABBABABABBBABBBB-BAAABAABBABAAAAB-BBBBBABAABB-BBAAAA-ABBBABABBAABABAABBAABAABABABBBBABBAA

*000000_00415014 -BBABABABBBABBBB-BAA-BAABBABABAAB-BBBBBABA-BBBBBAAA-BABBBABABBAABABAABBAABA-BABABBB-ABBAA

*000000_00498031 ABBABABABBBABBBB-BAA-BAABBABABA-B-BBBBBABA-BBBBBAAA-BABBBABA-BAABABAABBAABA-BABA-BB-ABBAA

*000000_00580896 ABBABABABBBABBBB-BAA-BAABBABABAAB-BBBBBABAABBBBBAAA--ABBBABA-BAABABAABBAABA-BABABBB-ABBAA

*000000_00580982 ABBABABABBBABBBB-BAA-BAABBABABAAB-BBBBBABAABB-BBAAA-BABBBABA-BAABABAABBAABA-BABA-BB-ABBAA

*000000_00814989 ABBABABABBBABBBB-BAA-BAABBABABAAB-BBBBBABAABBBBBAAA--ABBBABA-BAABABAABBAABA-BABABBB-ABBAA

*000000_00924857 ABBABABABBBABBBB-BAA-BAABBABABAAB-BBBBBABAABB-BBAAAA-ABBBABA-BAABABAABBAABA-BABA-BB-ABBAA

*000000_00929585 ABBABA-ABBBABBBB-BAA-BAABBAB--AAB-BBBBBABAABB-BBAAA--ABBBABA-BAABABAABBAABA-BABABBB-ABBAA

*000000_01023120 ABBABAAABBBABBBBBBAAABAABBABBBAAB-BBBBBABAABBBBBAAAB-ABBBABA-BAABABAABBAABA-BABABBB-ABBAA

*000000_01125209 ABBABA--BBBABBBBBBAA-BAABBABBBAAB-BBBBBABAABBBBBAAAB-ABBBABA-BAABABAABBAABABBABABBB-ABBAB

*000000_01219529 ABBABA---BBABBBBBBAABBAABBABBBAAB-BBBBBABAABBBBBAAABBABBBABA-BAABABAABBAABA-BABABBB-ABBAB

*000000_01219615 ABBABA---BBABBBB-BAA-BAABBABBBAABBBBBBBABAABBBBBAAAB-ABBBABABBAABABAABBAABA-BABABBB-ABBAB

*000000_01352675 ABBABAAAB-BABBBBABAA-BAABAABBBAABABBBBBABAABBBBBAAA-AABBBABA-BAABABAABBAABA-BABABBB-ABBAA

*000000_01401510 ABBABAAAB-BABBBBABAA-BAABAABBBAAB-BBBBBABAABBBBBAAA--ABBBABA-BAABABAABBAABA-BABABBBBABBAA

*000000_01416024 ABBABAAAB-BABBBBABAA-BAABAABBBAAB-BBBBBABAABBBBBA-A--ABBBABA-BAABABAABBAABA-BABABBB-ABBAA

*000000_01441139 ABBABAAAB-BABBBBABAA-BAABAABBBAAB-BBBBBABAABBBBBAAA--ABBBABA-BAABABAABBAABA-BABABBB-ABBAA

*000001_00030203 ABAAAAAAAABAAAABAAAABBABBBBAAABB-BAAA-AAABBBBBBAABABAABAAAAABABA-AABABBBBBBAB-BBAAABBABBB

*000001_00031053 ABAAAAAAAABAAAABAAAABBABBBBAAABB-BAAA-AAABBBBBBAABABAABAAAAABABA-AABABBBBBBAB-BBAAA-BABBB

*000001_01259620 BAAAABAAAABAAAABAAAABBABBBBAAABBABABBB-AAB-BB-BAABABBABAABAA-ABA-AABABBBABBAB-BBAAABB-BBB

*000001_01481617 B-AAABAAAAB-A-BBAAAABBABBBBAAABBABABB--A-BBBBBBAABABBAA-ABAABABA-AABAB-BABBAB-BBAAA--A-BB

*000002_00195613 AAAAAABB-AABABBAAAABBABBB-ABBAAABABAAABBBBAABBBAAABABB--BABAAABAA-ABBBAA-AABABB-BBBBABAAA

*000002_00358577 A-AAAA-BBAAB-BBABAAB-ABB--ABBAAA-ABAAABBBB-AB--AAA-AB---BABAAABAAAABBBAA-AABABBAB-BBABA-A

*000002_00363202 AAAAAA-B-AABABBA-AAB-ABBBAABBAA-BAB-AABBBB-ABBBAAA-ABBA-BABAAABAA-ABBBAAAAABABBABBBBA-AAA

*000002_00406165 AAAAAA-B-AABABBA-AABBABBB-ABBAAABABAAABBBBAABBBAAABABB--BABAAABAA-ABBBAA-AABABB-BBBBABAAA

*000002_00763940 A-AAAA-B-AABABBA-AABBAABB-ABBAAAAAA-AABBBB-ABBBAAAAABB--BABAAABAAAABBBAA-AABABB-BBBBABAAA

*000002_00793122 AAAAAA-BBAABABBAAAABBAABB-ABBAA-AAAA-ABBBBAA-B--AAAAB---BABA-ABAAAABBBAA-AABABB-BBBB--AAA

*000003_00222132 BBBBBBAAAA-BBB-BAB--AAAAAABBABABAAABAABAABAAAA-BABBBBAABBB-ABAAAB-A-AABBBAAABBAAABBAABABB

*000003_00232994 BB--BBAA-ABBBBABAB---A-A-ABBA-ABAA-B-A-A-B-A-A-BABBBB-ABBB-ABAA-BBA-A-BBBAAABBAAABBA-BABB

*000003_00796342 B-B-BBAAAAABBB-BABA-AAAAAABBAAABAAABAABAABAAAABBABBBAAABBB-ABAAABBA-BABBBAAABBAAABBAABABB

*000003_01206319 BBBBBBAABAABBB-BABA-AAABAABBAAABAAABAABAABAAAABBABBBAAABB--ABAAABBA-BABBBAAABBABABBAABABB

*000003_01207777 B-BBBBAABAABBB-BABAAAAA-AABBAAABAAABAABAABA-AABBABBBAAABBB-ABAAABBA-BAB-BAAABB-B-BBAABABB

*000003_01208792 --BBBBAABAA-BB--ABA-AAABAA-BAAABAAABAABAA-AAAA--ABBBAAA-BB-ABAAA-BA-BABBBAAAB-ABABBAA-ABB

*000003_01349293 B-BBBBAA-AAB-B-BA-A-AAABAABBAAABA-A-AABAABAAAAB-A--BA---BB-ABAAABBA-BA-B-AA-BBAB---AA-ABB

*000003_01475061 B-BBBBA-BAABBBBBBBA-AAABAABBAAABAAABAABAAB-AAA--ABB--AABB--ABAAABBA-BABBBBAABBAB-BBA-BABB

*000004_00224728 B--AABAAABABBABBBB-BAAABA--ABA---B-ABBBAAAABAA-AABABABABBAAAAAAAABAAABBBABB-ABBBBAABBAAAA

*000004_01254246 AB-A--BBABA-BAA-B-ABA--B--BABA--BBB--BB-AA-BAAB---AB--BBBAAABAAAAAABABBBBBBABB---AABBBAAA

*000004_01423078 ABAAAABBABAA-A-BBBABAAABA-BABABABBBBBBBAAAABAABAABABABB-BAAABAAAA-ABABBBBBBABB-BBAAB-BAAA

*000004_01450135 ABAAAABBABAA-A--B-ABAAA-ABBABABABBBBBBBAAA-BAABAABABA-BBBAAABAAAA-ABABB--BBABB--BAAB-BAAA

*000005_00007918 B-BBABABABABBAABBBBBBAAA-BAABB-AABABBBABBB-AA-AABABBAA-BABBAABBBBBA-BBBBBBBABB-BABBBAAABB

*000005_00047918 BA-B-BABABABBAABBBBBBAAA-BAABB-AABABBBABBBBAABAA-A-BA-BBABBAABBBBBAABBBBBBBABBBB-BBB-AABB

*000005_00643729 -A-BBAAA-ABBBABAABABBBBBB-AA-BA-ABABBBBBBB-BBAAA-AAAA--BBABAAABABBBBAA-BBB-ABAABBBBAAABAA

*000005_00815196 AABBBAAB-AB-BABAABA-BBBBBBAABBBBABABBBBBBBBBBAAA-AAABBABBABAAABABBBBAA-BBBBABAABBBBAAABAA

*000005_00916548 AABBBAAB-ABBBABAABABBBBBBBAAB-BBABABBBBBBBBBBAAA-AAABBABBAB-AABABBBBAA-BBB-ABAABBBBA--BAA

*000005_00962963 AABBBAABAABABABAABABBBBBBBAA-ABBAB-BBBBB-BBBB-AA-AAAB-ABBABAAABAB-BBAA--BBBABAABBBBAAABAA

*000005_01115568 AABBBAAB-AB-BABAABABBBABBBAA--BBABABBBBBBBBBBAAA-AAABBABBABAAABABBBBAA-BBABABAABBBBAAABAA

*000006_00433063 AB-B-ABBAB-BA-BAAABAABAAABAAABBBAB-AABAABBAAABBAAAABAABAAABAAA-BAB-BBAABBBBAA-B---AAAAB--

*000006_00561661 AB-B-ABBAB-BAABAAABAABAAABAAABBBAB-AABAAB-AA-BB-AAABAABAAABAAAABAB-BBAABBBBAA-B-A-AAAAB--

*000006_00772572 AB-B-ABBAB-BAA-AAA-AABAAABAAABB-AB-AABAB-BAA-BB--AABAABAAA-AAAA-ABB-BAABBBBAA-B-ABBA--B-B

*000006_00796781 ABBBBABB-B-BAAB--AB----A-BAAABB-AB-AABABBB---BBBAAABA-BAAABAA-ABAB-BBAABBBBAA-BB-BBAAA-B-

*000006_00802722 A--B-ABBABABAABA-A-AA--A-BAAABBBAB-AABABBB-AABB-AAABA-BAAABAAAABAB-BB-ABBBBAA-BA-BBAAAB--

*000006_00865002 AB-B-ABBAB-BAABAAABAABAAABAAABBBAB-AAB-BBBAA-BBBAAABAABAAABAAAABAB-BBAABBBBAA-B-A-BAAAB--

*000006_01049018 A-ABBABBAB-BAABAA-B--BAAABAAA-BBAB-AABABBB-A-BBB--ABAAB-B-BAAAABA-BBBAABBBBAABB-ABBAAABA-

*000006_01075342 AB-B-ABBAB-BAABAAABAABAAABAAA-BBAB-AABABBBAA-BBBAAABAABABABAAAABAB-BAAABBBBAA---ABBAAAB--

*000007_00293832 B-B-BBB-BBB-ABBABABAB---A--BABB-AAA--BAAB-BAAB-BBBB-BABA--BBAAAAAABB-ABBABB-A--B-A---BAAA

*000007_00352014 --BBBBB-BBBAABBA-ABABAB-BABBAB-BAAA-ABAABBBAAB-BBBBABABABABBAAA-AABBBABBABBAABABBAABABAAA

*000007_00380862 B-BABBB-BB-AABBABABABAB--ABBABBBAAAAABAABBBAAB-BBBBABABABABBAAAAAABBBABBABBAABABBAAA-BAAA

*000007_00666934 B-BAB-B-BBBAABBAAABABAB--ABBABBBAAAAABAABB-AA--BBBBABABA-ABBAA-AAAB-BABBABBAABAB-A--A-AAA

*000007_00690643 BABABBB-BBBAABBAAABABAB--ABBABBBAAAAABAABBBAAB-BBBBABABABABBAAAAAABBBABBABBAAB-BBAAA-BAAA

*000007_00897244 BABABBB-BBBAABBAAABABAB--ABBABBBAAAAABAABB-AAB-BBBBABABABABBAAAAAABBBABBABBAAB-BBAAA-BAAA

*000007_01038475 B-BABBB-BBBAAB-AAABABABB--BBABBBAA-AABAABBBAAB-BBBB-BABABABBAA-AAAB-B-BBABBA-B-BBAAA-BAA-

*000008_00284834 ABBBBABBABBBABBBBAB-AAAABABAABABABAAA-AAABBBA-ABAA-BABBBBBBABBA-ABAAA-BBBABAABABAAAAABBBB

*000008_00351987 ABBBBABBABBBABBBBAB-AAAABABAABABABAAA-AA-BBBAAA-AA-BABBBBBBABBA-ABAAABBBBABAABABAAAA-BBBB

*000008_01012708 ABBBBABBABBBABBBBAB-AAAABABA-B-BABAAAAAAABBBAAABAABBAABBBBBBBBA-ABAAABBBBABAABABAA-AABBBB

*000008_01085352 ABBBBABBABBBABBBBAB-AAAABABA-BABABAAAAAAAB-BAAABAABBAABBBBBB-BA-ABAAABBABABAABAAAAAAA-BBB

*000008_01145733 ABBBBABBA-BBABBBBABBAAAABABA-B-BAB---AAA-BBBAAABAABBAABB-BBBBBA-ABABABBABABAABAAAAAAABBBB

*000008_01159093 ABBBBABBABBBABBBBAB-AAA-BABABB-BAB-A--AAAB-BA-ABAAB--AB-BBBBBB-AABA-AB-ABABAA-AAAAAAABBBB

*000009_00143922 BABBBBBABBBBABAABABAABAABAAAAA-AAAABBABAB-ABBBBB-AAAABBBBAAABBAABAABAB-BA-AABBBB-AABB-BAA

*000009_00734403 B-ABABBABBBB-AAAB-BAABAAB---A-B-A-A-BABA--ABBBBB-A-A-BABBAAAAB-BBAABAB-BAABABBBBAAABBB--A

*000009_00882090 B-ABABBABBBB-AAAA-BAABAABAAABABAAAABBABAB--BBBBB-AAA-BABBAAA-BABBAABAB-BA-B-BBBBAAABBBBAA

*000009_00917961 BAA-ABBABBBB-AAAA-BAABAABAAABABA-A--BABAB-ABBB-B-AAAABABBAA-ABABBAAB-B-BABB-BBBB-AABB-BAA

*000009_01135653 B-ABABBAB-BB-AAAA-B-ABAABAAA-A--AAA-BAB-BB-BBBBBAAAAA-ABBAAAABABBAABABBBA-BA-BBBBA-BBBBAA

*000010_00316958 -A-AA--BB-BAA--BB-AB-BBAB-ABBB--BBBAAB-AAB-AAA--BBBBBABBBBBA-A-BBBABA-BAABB--ABA-BB-B-ABB

*000010_00445344 AAAA-AAB-ABAABBBBA-BABBABB-BBBA--B--ABBAAB-AAAAABBBB-ABBBBBABA-BBBABABBAABB-AA-A-BB--AABB

*000010_00479212 AAAAAAABBABAABBBBAABABBABB-BBBABBBBAABBAAB-AA-AABBBBBABBBBBA-A-BBBABABBAABB-AABABBBBBAABB

*000010_00584698 AAAAAAABBABAABBBBAABABBABB-BBBABBBBAABBAAAAAAAAABBBBBABBBBBA-A-BBBABABBAABB-AABABBBBBAABB

*000010_00859129 A--AAAA-BABAABBBBAABABBABB-BBB-B-B-AABBAA-AAAAAABBBBBABBBBBA-A-BBBABABBAABBBAA-A-BB-B-ABB

*000011_00049376 B-BBBBAAABBAAABBBA-AAABBBAAAAABAAAAAABB-AA-AA---AB-ABABBABAABAAAABAABBBAA-BA-AABBBBBAABAA

*000011_00121688 B-BBBBAAABBAAABBBA-AAABBBAAAAABAAAAAAB--AABAA--BAB-ABABBABAABAAAABAABBBAA-BA-AAB-BBB-ABAA

*000011_00122911 B-BBBBAAABBAAABBBA-AAABBBAAAAABAAAAAABB-AABAA--BAB-ABABBABA-BAAAABAABBBAA-BA-AAB-BBBAABAA

*000011_00123945 B-BBBBAAABBAAABBBAAAAABBBAAAAABAAAAAABBBAABAA--BAB-ABABBABAABAAAABAABBBAA-BA-AAB-BBBAABAA

*000011_00261702 B-BBBBAAABBAAABBBA-ABABBBABAAABAAAAAABB-AABAA-ABAB-ABABBABAABAAAABAABBBAA-BA-AABABBBAABAA

*000011_00353725 B-BBBBAAABBAAABBBA-ABABBBABAAABAAAAAABB-AABAAB-BAB-ABABBABAABAAAAAAABBBAA-BA-AAB-BBBAABAA

*000011_00356286 B-BBBBAAABBAAABBBA-ABABBBABAAABAAAAAABB-AABAA--BABBABABBABAABAAAAAAABBBAA-BA-AAB-BBBAABAA

*000011_00356804 BBBBBBAAABBAAABBBABABABBBABAAAB-AAAAABB-AA-A---BAB--BABBABAABAAAAAAABBBAA-BAAAAB-BBBAABAA

*000011_00377891 B-BBBBAAABBAAABBBA-ABABBBABAAABAAAAAABB-AABAAB-BAB-ABABBABAABAAAAAAABBBAA-BA-AAB-BBBAABAA

*000011_00782031 BBBBBBAAABBAAABBBA-ABABBBABAAABAAAA-ABB-AABAAB-BABBAAABBAAAA-AAAAAAAABBAA-BA-AAB-BBBAABAA

*000012_00050453 A-BABAAABAABABABBA-AB-AAAABBBBBAAA--BAABBBBBB-ABBBBAA-BBAAAB-ABBABBB-BB-BBABB-BA-BB-B-BA-

*000012_00119996 A-BABAAABAABABABBA-AB--AAABB-BBAAAABBAABBBBBBBABBBBAA-BB-AAB-ABBABBBA-BABBABB-BA-BBB-ABAA

*000012_00125057 ABBABAAABAABABABBA-AB-AAAABBBBBAAAABBAABBBBBBAABBBBAAABBAAAB-ABBABBBA-BABBABB-BAABBBBABAA

*000012_00138783 ABBABAAABAABABABBA-AB-AAAABBBBBAAA-BBAABBBBBB-ABBBBAAABBAAAB-ABBABBBABBABBABB-BAABBBBABAA

*000012_00321072 ABBABAAABAABABABBA-AB-AAAABBBBBAAAABBAABBBBBB-ABBBBA-ABBAAAB-ABBA-BBABBABBABB-BAABBBBABAA

*000012_00663434 ABBABAAABAABABABBA-A-AAAAABBBBBAAAABBAABBB-BB-ABBBBAAABBAAAB-ABBA-BBABBABBABBABAABBBBA-AA

*000012_00880300 AB-ABAAABAABABABBA--B-ABAABBBBB-AA-BBAABBBBBBBABBBBA-ABBAAABBAB-A-BBABBABBABB-BAAB-BBABAA

*000012_01004948 A-BABAABBAABA-ABBABABAABAABB-BBA-AABBAABBBBBB-ABBBBAAABBAAAB-ABBA-BBA-BAB-ABB-BAAB---ABAA

*000013_00060968 BBAAABAB-BABAABABABABAAB-AABBAAABBAA-ABBBA-BA-BBAAAABBABAABAABAAAA-BBBABAABBAABBBAAAAAAAA

*000013_00404421 BBAAABABBBABABBABABABAABA-ABBAAABBA--ABBBA-BAABBAAAABAABAABAABAAAABBBBAAAABBAABABAAAAAAAA

*000013_00443988 BBAAABABBBABA-BABABABAABA-ABBAAABBA--ABBBABB-AB-AAAAB-ABAABAABAAAABBBBAAAABBAABABAAAAAAAA

*000013_00910363 BBAAABABBBABA-BABABABAABA-ABBAAABBA--ABBBABAAABBAAAABAABAABAABAAAABBBBAAAABBAABABAAAA-AAA

*000013_01013544 BBAAABABBBABA-BABABBBA-BA-ABBAAABB-A-A-BBABAAABBAAAABAABAABAABAAAABBBBAAAABBAABABAAAABAAA

*000013_01214252 B-AAABABBBABA-AABABBBA-BA-ABBAAABBA--A-BBABAAAB-AAAABAABABB-ABAAAABBBBAA-ABBAABABAAAABAAA

*000014_00143047 ---B-BBBA--AAB-ABABAAB-AA-AAAB-BABABBB-ABB--A-BABAAA-A-AB-B--A-AABAB-AAAABABAA-AAAA-B--AA

*000014_00337661 BB-B-BBBAB-AA-BABABAABAAABAAABABABABBBAABBAAABB-BAAA-ABABABAAA-AAB-BBAAAABA-A-BAAAAAB-AAA

*000014_00441637 B--BABBBAB-AA-BABABAABAAABAAABABABABBBAABB-AABB-BAAA-ABAB-BAAA-AAB-BBAAAABA-A-BA-AAAB-AAA

*000014_00467297 B--B-BBBAB-AA-BABABAABAAABAAABABABA-BBAABB-AABB-BAAA--B-BABAAA-A-B-ABAAAABA----A-A-AB-AAA

*000014_00475367 BBABABBB-B-AABBA-ABAAB-AABAAABABAB--BBAABB-AABB-BAAA-A-ABABAAA-AAB-ABA-AABA-A-BAAAAABBAAA

*000014_00864396 BB-B-BBBAB-AAABABABAABAAABAAABABABABBBAABBA-ABB-BAAAAABABABAAA-AAB-ABAAABBABA-BAA--AA-BAA

*000015_00781653 BBB-BBABAA-ABB-A-BBABBBB-A-AAAB-BB-ABABBAAAB-AAABBAABBBBBBBABAB-BAABABABAABAAABBB--B-BAAA

*000016_00765785 A-A-AAABA-BBB-ABBBBABBABAABBA-AABABBBBABABBBB--AA-AABBABABBAABBBB--BA-BABAAA-AAAAAAABAAAA

*000016_00806801 A-A-AAABA-BBB-ABBBBABBABAABBA-ABBABBBBABABBBBB-AA-AABBABABBAABBBB--BA-BABAAA-AAAAAAABAAAA

*000017_00239681 B-AAABBBBBBBBBABBBBB--AABBABA-BBAAAAABABBA-A--BABAAABB-BABBBBA-AAAABAABABABBBBBAABBAAABAA

*000017_00245283 B-AAABBBBBBBBBABBBBB--AABBABA-BBAAAAABABBA-A-BBABAAABBBBABBBBA-AAAABAABABAB-BBBAABBAAABAA

*000017_00250996 B-AAABBBBBBBBBABBBBB-AAABBABA-BBAAAAABABBA-AABBABAAABB-BABBBBA-AAAABAABABABBBBBAABBAAABAA

*000017_00606983 BBAAABBBBABBBBABBBBB--AABBABA-BAAAAB-BABBABA--BABAAABB-BABBBBA-AAAABAABABABBBB-AABBAAABAA

*000017_00802901 BBAAABBBBABBBBABBBBBA-AABBABAABAABAB--ABBA-A-BBABAAAAB-BA-BBBA-AAAAB-ABABABBBB-A-B--AABAA

*000017_00919032 B--AABABBABBBBABBBBB--AABBAB--B--BBB--ABBABA-BBABAA-AB-BABBBBA-AAAAB-A-ABABBBBBAABBA-ABAA

*000019_00068275 AAB-BABABBBBABABBAAAB-BAAABAABABBB-B-AB-BB-BA-BBBABAB-BBABBBBABBBBBAB-AABAAB-BAB-BBB-AAAA

*000019_00094657 AABBBABABBBBABABBAAABABAAABAABABBBBBBABBBBBBAABBBABABABBABBBBABBBBBAB-AABAAB-BABBBBBAAAAA

*000019_00504401 AABBBABABBBBABABBAAABABAAABAABABBBBBBBBBBBBBAABBBABABABBABBBBABBBBBBBAAABAABABABBBBBAAAAA

*000019_00537184 AABBBABABBBBABABBAAABABAAABAABABBBBBBBBBBBBBAABBBABABABBABBBBABBBBBBBAAABAABABAB-BBBAAAAA

*000019_00594022 AABB--BABB-BABAB-A-A--BA-AB-ABA--BBBBBBBBB-BAABBBAB-BABBA-BBB-BBBBBBBAAABAAB-BABBBBBAAAAA

*000019_00967569 AABBBABABBBBABABBAAABABAAABAABABBBBBBBBBBBBBAABBBABABABBABBBBABBBBBBBAAABAABABABBBBBAAAAA

*000020_00184780 AAB-BAABAAABB-AAABBBBBBBBA-ABABBAB-BBBABBBAB-AA-A-AAB--BBABA-AB--ABBBB---ABBBB-BB-A--BA--

*000020_00348055 AABABAABAAABBAAAABBBBBBBBA-ABABBABABBBABBBAB-AAAAAAABAABBABAAABABABB-B-B-ABBBBABBAAAABAAA

*000020_00629203 A-BABAABAAABBAAAABBBBBBBBA-ABABBABABBBABBB-B-AAAAAAABAABBABAAA-ABABB-BA--ABBBBBBBAAAABAAA

*000020_00989237 A-BBBAAABABABBBA-BABBABBBB--BBAB-BABA-B-BB-B-AAABB---B-BBABB-AAABBBBB--BBBAAB-ABBBBAA-BAA

*000020_01170664 AABABAABAAABBAAAABBBBBBBBA-ABABBABABBBABBBAB-AAAAAAAB-ABBABAAABABABB-B-B-ABBBB-BBAAAABAAA

*000020_01293917 AABA-AABAAABBAAAA-BB-BABBA-ABAB-ABABBBABBBABBAAAA-AABA-BBABA-ABABABB-B-BAAB-BB-BBAAAA--AA

*000020_01342362 AABABAABAAABBAAAABBBBBABBA-ABABBABABBBABBB-BBAAAAAAABAABBABAAABABABB-B-B-ABBBB-BBAAAABAAA

*000021_00059950 --B-B-BBBAA-BABAABABABA-BBAAABABBBBB-BBBBBAB--AA-AAAB-B-AAA-ABABAAB-BA-BBAAAAABBBB-B-B-AA

*000021_00544759 -AB-B-BBBAAABABAABABABAA-BAAABABBBBBBBBBBB-BAAABAAAAB-BAAAAAABABAAB-BAABBAAA-A-BBBBBBBBAA

*000021_00716877 A-B-BAAAABBBABBA-AABBBAB-BABAA-ABB--AABBA--BBBA--BA-B-B--BABAB-B-BBA---BABB-AAAB-AAAAAAAA

*000021_00953227 A-BBBAAAABBBABBAAAABBBAB-BABAABA-B-BAABBAA-BBBA-ABAABABAABABA-BBABB-AA-BABBBAAABBAAAAAAAA

*000021_00981124 ABBB-AAAABBBABBAAAABBBAB-BABAABA-BABAABBAABBBBABABAABABAABABABBBABB-AABBABBBAAABBAAA--AAA

*000022_00048645 ABAAAAAAAABABAABBBBABBB-AAA-BABAABAAA-ABBA-ABBABBAABAAABBAAAAABABBBABAAABAAB-BAABBBAAABBB

*000022_00120600 ABAAAAAAAAAABAABBBBABBB-AAAABABAABAAA--BBAAABBABBAABAAABBAAABABABB-ABAAABAAB-BAABBBAAABBB

*000022_00294460 ABAAA-AAAAA-BAAB-BBABBB-AA-ABA--ABAAA-ABBA-ABBAB-BABA-ABBAAABABABBBABAAABAAB-BAAB-BAAABBB

*000022_00376343 A-AAA-AAAAAABAA-BBBABBB-AAAABAB-ABAAABABBAAAB-ABBBABAA-BBAAABABABBAABAAA-AAB-BAA-BBAAABBB

*000022_00473187 ABAAAAAAAAAABAABBBB-BBB-AAAABABAABAAA-ABBAAABB-BBBABAA-BBAAABABABBAABAAABAAB-BAABBBA-ABBB

*000022_00563452 ABAAAAAAAAAABAABBBBABBB-AAAA-ABAAB-AA-ABBAAABB-BBBABAA-BBAAABABABBAABAAABAAB-BAABBB-AABBB

*000023_00166545 --ABABBABBABBB-ABBBBBBBBBA--AAABBABBBABA-AA-BBBABAAABBABBABABB-ABAABABBAABABBB-A-BBBBBAAA

*000023_00331417 BBABABBABBABABBABABBBBBBBAB--AABBABBBABABBABB-BABAAABBABBABABB-ABAABABBA--ABBBBA-BBBBBAAA

*000023_00386496 BBABABBABBABABBABABBBBBBBAB-AAABBABBBABABBABBBBABAAABBABBABABB-ABAABABBA--ABBBBA-BBBBBAAA

*000023_00480946 BBABABBABAABABBABABBBBBBBAB-AAABBABBBABABBABBBBABAAABBABBABABB-ABAABABBA--ABBBBA-BBBBBAAA

*000023_00757993 BBA-ABBABAABABBABABBBBBBBABAAAAAB-BBBABABBABBBBABAAABBABBABABB-ABAABABBAA-AABBBA-BBBBBBAA

*000024_00029994 B-AAA-AABBAABBAA-B--BB---AB-BAA-AAAB-AB-BA--BBAABBAA-BABA-B-BBBAAAAB-BAABBAB--BABBB-ABAAA

*000024_00061356 BBAAABAABBAABBAAABABBBBBBABBBAAA-AABBAB-BAA-BBAABBAABBABAAB-BBBAAAABBBAABBABB-BABBBAABAAA

*000024_00167052 BBAAABAABBAABBAAABABBBBAB-BBBAAA-AA-BAB-BAA-BBAABBAABBABAABABBBAAAABBBAABBABB-BABBBAABAAA

*000024_00206138 BBAAABAABBAABBAAABABBBBABABBBAAA-AABBAB-BAA-BBAAB-AABBABAABABBBAAAABBBAABBABB-BABBBAABAAA

*000024_00337407 B-AAABAABBAABBAAABABBBBABABBBAAABABBBAB-BA--BBAABBAAB--BAABBBBBAAAABBBAABBABB---BBBBAB-AA

*000024_00399170 BBAAABAABBAABBAAABABBBB-BABBBAAABBBBBAB-BAA-BBAABBAABAABAABBBBBAAAABBBAABBABB-BABBBBABAAA

*000024_00893786 --AAABAABBAABBA-ABABBBBBBABBBAABBBBBBAB-BA--BBAABBAABAABBABBBBBAAAABBBAABBABBBBAABBBABAAA

*000025_00023594 BB-B-BBBABAAAA-A-ABBAA-AABBAABABAAABBBAAABBAABBABAAB-ABABABAAA-AABABB-AA-BA-BABAAAABB-ABB

*000025_00541633 BB-B-BBAABAAAABAAA-BAA--ABBAABABAAABBB-AABBAABBABAAB-ABAAABAAA-AABABB-AA-BA-BABAAAABB-ABB

*000025_00702139 BB-B-BBAABAAAABA-ABBAAA-ABBAABABAA-BBB-BAB-AABBABAAB--BA-ABAAA-AABABBAAA-BA-BAAAAAABBBABB

*000025_00923463 BBA--BBAABAAAABAAABBAA---BBAABABAAABBB-BAB-AABBABAAB--BAAABAAA-AABABBAAA-BA-BAA--AABB-ABB

*000026_00553904 AABABABABB-BABABBAAA-ABABABAAB-BBBBBBB-BBB-BABB-BBAABABBABBBBABABBBBBABAAABBABABABBB-AAAA

*000027_00535275 BBAAABAAAABA-BBAA-AABBBABABABABABBBB-AABBBBBBBBAAAAAB-BAABBBBABBBABABBA-BABAABB-ABBAABAAA

*000027_00684968 BBAAABAAAABA-BBAA-AABBBABABABABABBBBBAABBBBBB-BAAAAABBBAABBBBABBBABABBA--ABAABB-ABBAABAAA

*000027_00766615 BBAAABAAAABA-BBAA-AABBBABABABABABBBBBAABBBBBBB-AAAAABBBAABBBBABBBABABBA-BABAAB--ABBAABAAA

*000028_00311393 AABBBAAABABABBAAABBBBABBBBAAABABAAAAAA-BBBABB-AAABAABBBABABBA-ABBAABBAABBB-AB-ABBBB-AABAA

*000028_00365899 AAB-BAAA-A-ABBAAA-BBB-B--BA-ABA-AAA-AABB-B-BBAA-A-AABBBA-ABBAAA-BAABBAABBBAAB----BBAAABAA

*000028_00914968 A-BBBAA--ABABBAAABABBABBBBAAABABAA-AAAB-BB-B-AAAB-AABBBABABBAAABBAABBAABBB-AB-ABBBB---BAA

*000029_00024485 AABBBAAB-ABBBABAABABBBABBAAAB-BBABA-BBBBBB-BBAAA-AAAB-ABBABAAABABBBB-A-BBABABA-BBAAAAABAA

*000029_00361197 AABBBAABAABBBABAABABBBABBAAAB-BBABA-BBBBBB-BBAAA-AAABAABBABAAABABBBB-ABBBABABA-BBAAAAABAA

*000029_00523925 AABBBAABAABBBABAABBBBBABBAAAB-BBAB--BBBBBBBBBAAA-AAABAABBABAAABABBBB-A-BBABABA-BBAAAAABAA

*000029_00675929 AABBBAA-AABBBAB-ABBBB-ABBAA-B-BBABA-BB-BBB-B-AA-BAA-BAA-BABA-A-AB-BB-A-BBA---A-B--A-AABAA

*000030_00070857 AB--BAABAABABBBBBBBABBAA-BBABBB-AAABBBBBBB--B-B-BABBBABAA-BBAABABABBBBBBBABBAB-BABB-ABABB

*000031_00071162 BABABBBAAABAABA-BAABABAAABABBBABABAAABABBABAABABAB-BBABABBBABAABB-AAAABABBAB-AAAABBAAA-BB

*000031_00097809 BABABBBAAABAAB--BA-BABAA--ABBBA-ABA-ABA-BABAABABAB-BBABABBBA-AABB-AAAABA-BA--AAAABBA-AABB

*000031_00263085 BABABBBAAABAABA-BAABABAAABABBBABABAAABABAA-AABAAAB-BBABABBBABAABB-AAAABABBAB-AAAAB-AAAABB

*000031_00280403 B-BABBBAAABAABA-BAABABAAABABBBABAB-AABABAABAABAAAB-BBABA-BB-BAABBBAAAABABBAB-A-AABBAAAABB

*000031_00538050 BABABBBBAABAABA-BAABABAAABABBBABABAAABABAABAABAAAB-BBABABBBABAABB-AAAABABBAB-AAAABBAAAABB

*000031_00800413 B-BABBBB-ABAAB-BAA-B-B--AB-BBB-B-BAAABAB-ABAABAAABBBBABABBB-BAABB-AAAABABBAB-AAAABBAA-ABB

*000031_00918886 BABABBBBAABAABA-AAABABAAABABBBABABAAAB-BAABAABAAAB-BBABABBBABAABB-AAAABABBAB-AAAABBAAAABB

*000032_00543121 BBBABBBBAABA-BABAAABABAAABABBBBBBBB-ABA-AAAAABA--B-BB-BABBBABAABB-A-AABABBAB-AAA--BBABABB

*000032_00659865 BBBABBBBAABAABABAAABABAAABABBBBBBBBAAAAAAAAAABAABBBBBABABBBA-AABBBAAAABABBAB-AAABBBBABABB

*000032_00811851 BBBABBBBAABAABABAAABABAAABABBBBBBBBAAAAAAAAAABAABBBBBABABBBA-AABBBAAAABABBAB-AAABBBBABABB

*000033_00179598 A-AB-A-B-AB-BABB--BAABBBB-A-BBB-AA-BBBBAAA-B-BA-AAAABBAABBAABBABAABABBBABBBABABABAABBABAA

*000033_00809478 ABABA-BB-BB-BABB-B-AAB-BB--BBBB--BABBB-A-A-B-B-AAAAABBABBBAABBABAABABB-ABA-AB-BABAABBA-AA

*000033_00887457 ABABAABBBBBABABBABBAABBBBAABBBBBABABBBBABABB-BAAAAAABBABBBAABBABAABABBBAB-BABABABAABBABAA

*000033_00910422 ABABAABBBBBABABBABBAABBBBAABBBBBABABBBBABA-B-BAAAAAABBABBBAABBABAABABBBAB-BABABABAABBABAA

*000034_00013100 A-AAAAABAABAAABBAAAABABBBABAABBBBBAAABAABB-BBBBAABABABBAAAAAAABB-AABABBBBABABBBBAAABBABBB

*000034_00013186 AB--AA-B-ABA-ABBAAAABABBBABAABBBBBA-ABA-BBBBBBBA-B-BABB--AA-AABB-AABABBBBABABBBBA-ABB-BBB

*000034_00112945 A-AAAAABAABAAABBAAAABABBBABAABBBBBAAABAABBBBBBBAABABABBAAAAAAABB-AABABBBBABABBBBAAABBABBB

*000034_00293793 ABAAAAAB-A-AAABBAAAAB--BB-BAABB-BBA-ABAABB-BBBBAABABA-BAAAA-AABB-AABABBBB-BABBBBAAAB-ABBB

*000034_00298767 A-AAAAABAABAAABBAAAABABBBABAABBBBBAAABAABBBBBBBAABABABBAAAAAAABB-AABABBBBABABBBBAAABBABBB

*000034_00339810 A-AAAAABAABAAABBAAAABABBBABAABBBBBAAAB-ABBBBB-BAABA-ABBAAAAAAABB-AABABBBBABABBBBAAABBABBB

*000034_00339896 A-AAAAABAAB-AABBAAAABAB-BABAABBBBB-AABAABB-BBBBAABABABBAAAAAA-BB-AABAB-BBABABBBBAAAB-ABBB

*000034_00367858 AAAAAAA-AABAAA-BAAAABAB-BABAABB-BBAAABAAB--BB---ABA-ABB-AAAAAA-B--A-ABB--A--BBBB-AA--ABBB

*000034_00539841 AAAAAAAB-ABAAABB-AAABABBB-BAABBBBBAA-BAABB-BB-BAABABA-BAAAA-AABB-AAB--BBBABABB-B-AABB-BBB

*000034_00574025 A-AAAAA-AA-AAABBAAAABA-B-ABAABB-BBAAABAABB-BB-BAA-A-ABBAAAAAAA---AA-A-B-BABA-BBBAAA--A-BB

*000034_00775541 A-AAAAABAABAAABBAAAABABBBABAABBBBBAAABAABBBABBBAAAA-ABB-AAAAAABB-AABABBBBABABBBBAAABB-BBB

*000035_00283007 A-ABAABBBBBABABBABBAABBBBAABBBBBAB-BBBBA-AAB--BAABBABBABBBAABAABAABABBBAB-BABA-ABAABBABAA

*000035_00352563 A-ABAABBBBBABABBABBA-BBBBAABBB--AB--BBBABABBAB---AB-BBABBBAABA-BA-BABBBAB-BABAAABA-B--BAA

*000035_00620643 AB-BAABBBBBABAB-AB-AABBBBAABBBB-ABABBBBABA-B-BAAAABABB--B-AABAABAABA-BB-B-BABA-ABAABBAB-A

*000035_00713647 ABABAABBBBBABABBABBAABBBBAABBBBBABABBBBABA-B-BAAAAB-BBABBBAABAABAABABBBAB-BABABABAABBABAA

*000036_00038524 BABABBBAAB-AAAABBA-ABAAABABA-ABAAAABBBA-BABBAB--BB-AA--BAA--BA-ABABB-AB-BABB--A-AB-BBBAAA

*000036_00069547 B-B-BBBAABAAAAABBABABAAAB-BABABA-AABBBABBA-BABBBBBBAAA-BAA-BBA-ABABBBAB-BABB--ABABBB-BAAA

*000036_00408864 BABABBBAAB-AAAABB--ABAAABABA-ABAAAABBBABBABBAB--BBAAAA-BA-ABBA-ABABB-AB--ABBAB-A-BBBB-AAA

*000036_00435213 BABABBBAAB-AAAABBA-ABAAABABA-ABAAAABBBABBABBA---BB-AAA-BAAABBA-ABABBBAB-BABB-BA-ABBBBBAAA

*000036_00625675 B-BABBBAAB-AAAABBABABABABABA-ABAAAABBBABBABBABBBBB-AAA-BAAABBA-ABABBBAB-BABB-BA-ABBBBBA-A

*000036_00659554 BABABBBAAB-AAAABBABA-ABABABA-ABAAAABBBABBABBABB--BBAAA-BAAABBA-ABABBBAB-BABB-BA-ABBBBBAAA

*000037_00035447 BAA---BABBAAAAB-BAABAAAABB-BABAA-B-AABABBABABBABBAABBBABBAB--AABAAAAAAAABA-BBAAAAAA----BB

*000037_00128852 BAAAABBABBAAAAB-BAABAAAABB-BABAA-BBAABABBABABBABBAABBBABBABA-AABAAAAAAAABA-BB-AAAAAAAAABB

*000037_00156139 ---AA-BABBAAA-B-BAABAA-ABB-B-BA-A---ABABBA-AB-A-BAABBBABBABA-AABAAAA-AAAB--BBAAAAAAA-AABB

*000037_00156225 BAAAABBABBAAAAB-BAABAAAABB-BABAA-BBAABABBABABBABBAABBBABBABA-AABAAAAAAAABA-BBAAAAAAAAAABB

*000037_00558324 BAAAA-BABB-AAABABAABAAAA-B-BAB-ABBB-A-ABBABAA-A-BAABBBABBABABAABAAAA--BABA-BBAAAAA--A--BB

*000037_00559379 BA----BA-BA-AA--BA-B-AAABB-BAB---BBAA-ABBA--A-ABB-AB--ABBABAAAABAAAAAABAB-BB-AAAAAAAA--BB

*000037_00686996 B-AAABBABBAAAAB-BAABAAAABB-BBB-AABAAABABBABAABABBAABBBABBABA-AABAAAAAABABA-BBAAAAAAAAAABB

*000037_00797938 B-AAABBABBBAAAB-BAABAAAABB-BBBAAABA-ABABBABAABA-BAABBAABBABA-A-BA-AAAABABABBBA-AAAA-AAABB

*000037_00800847 BAAAABBABBBAAAB-BAABAAAABB-B-BAAABAAABABBABAA-ABBAABBAABBABA-AABA-AAAABABA-BBAAAAAAAAAABB

*000039_00080448 BBAAABAAAABBABABBABABAA-BAAAAAB-BBBAA-ABBBBBBBBAABABAAABBABBBABABBAAAAAABBBB-BAABBBABABBB

*000039_00105560 B-AAABAAAABBABABBABABAA-BAAAAAB-BBBAA-ABBBBBBBBAABABA-ABBABBBABABBAAAAAABBBB-BAABBB-BABBB

*000039_00301463 B-AAABAAAA-BABABBABABAA-BAAAAAB-BB--A-ABBBBBBBBAABABAAABBABBBABABBAAAAAABBBB-BAABBBABABBB

*000039_00323093 BBAAABAAAABBABABBABABA--BAAAAAB-BBBAA-ABBBBBBBBAABABAAABBABBBABABBAAAAAABBBB-BAABBBABABBB

*000039_00519579 B-AA-BAAAAB--BABBAB-BBB-BAAAAABABBBAAAABB-BBBB--ABA----B-ABBBABABBA--A-ABBBB-B-ABB-ABABBB

*000040_00284820 AAAAAABAABAABA-BABABAAABABBABBBAB-BBBBBAAAABAAB-ABABABBBBAAABAAAA-ABABBBBBBABBBBBAABABAAA

*000040_00374581 A--AAABAABBABAABAB-BAAABABBABBBAB-BBBBBAAAABA-BAABABABBBB-AABAAABAAB-BBBB-B-BB-B-AABABAAA

*000040_00377737 AAAAAABAABBABA-BABABAAABABBABBBAB-BBBBBAAA-BAABBABABABBBBAAABAAAB-ABABBBBBBABBBBBAABABAAA

*000040_00379341 AAAAAABAABBABA-BABABAAABABBABBBAB-BBBBBAAAABAAB-ABABABBBBAAABAAAB-ABABBBBBBABBBBBAABABAAA

*000040_00387150 AAAAAABAABBABA-BABABAAABABBABBBAB-BBBBBAAAABAAB-ABABABBBBAAABAAA--ABABBBBBBABBBBBAABABAAA

*000040_00603861 AAAAAABAABBABA-BABABAAABABBABBBAB-BBBBBAAAABAAB-ABBBABBBBAAABAAAB-ABABBBBBBABBBBBAABABAAA

*000040_00759070 AAAAAABAABBABA-BABABAAABABBABBBAB-BBBBBAAA-BAAB-ABBBABBBBAAABAAAB-ABABBBBBBABBBBBAABABAAA

*000040_00905948 AAAAAABAABBABA-BABABAAABABBABBBAB-BBBBBAAAABAAB-ABBBABBBBAAABAAAB-ABABABBBBABBBBBAABABAAA

*000041_00084244 B--BABAA-BBA-A-BBABABABAAABAAABA-AABBBBAAABAAAAA-BBAAABBAAA-B-AAAAAA-BBABABBBABABBB--ABAA

*000041_00925290 B-A-ABA-ABB-AAA-B-BABABAAABAAA-AA-ABBBBA-AB-A--A--BAAAABAAAABAAAA-AAAB-ABAB-BABA-B-BAABAA

*000042_00361901 B-AAABAA-ABAAABBAAABBABBBAABBBBBBBAAABBBBB-BBBBAAAABAB-AAABBBBBBBBABABBBBBBBBBBBBAABBBBBB

*000042_00662123 BBAAABAA-ABAAABBAAABBABBBAABBBBBBBAAABBBBBBBBBBAAAABABBAAABBBBBBBBABABBBBBBBBBBBBAABBBBBB

*000043_00486337 B-A-ABAAAAAAAABAAAA-B-AABABBB-AA-B-AAABBBBB-ABBBAAAABB-AABBBBA-BBABA-AABAAABABA-B-B-AB-AB

*000043_00498437 B-AAABAAAAAAAABAA-AAB-A--ABBBAAA-BAAAABBBBBBABBBAAAAB--AABBBBABBBAB-BAABAAAB-BABBBB-ABAAB

*000044_00026052 BBBABBABAABAABBBBAABABAA-BABBBBABBBAAABAAAABBBAABBBBBA-BBBAABAABBBAAAABAABABAAAABBBBB-A-A

*000044_00153566 BBB-BBABAAB--BBBB--BABA--B-BBBBABB--AABAA--BBBA-BBBBBABBBB-AB-ABBBA-AABAABABAAA-BB--B----

*000044_00224406 BBBABBABAABAABBBBAABABAA-BABBBBABB-AAABAAAABB-A-BBBBBA-BBBAABAABBBAAAA-AABABAA-ABBBBB-A-A

*000044_00781275 B-BABBABBABAABBBBAABABAABBABBBBABBB-ABB-AAA-AB-ABBBBB--BBBAA-A-BBBAAABBAABABAAB-BB-BB-ABB

*000045_00090374 AAABAA-BA-BB-BAAA-B-AAAABAAAAABBAABBBABAB-A-BBBB-ABABBABA-BAABAABBAB-B-BA-BAABBBBAABBBAAA

*000045_00628144 AAABAA-BA-AB-BAAA-BABAAABAAAAABBAABBBABAB-A-BBBB-ABABBABABBAABBABBAB-B-B-BBABBBBBAAB-BAAA

*000045_00659062 AAABAA-BA-AB-BAAA-BABAAABAAAAABBAABBBABAB-A-B-BB-ABABBABABBAABBABBAB-B-B-BBABBBBBAABABAAA

*000045_00713516 AAABAA-BA-AB-BAAA-BABAAABAAAAABBAABBBABAB-A-B-BB-ABABBABABBAABBABBAB-B-B-BB-BBBBBAAAAAAAA

*000045_00813215 AAABAA-BA-AB-BAAA-BABAAABAAAAABBAABBBABAB-A-BBBB-ABABBABABBAABBABBAB-B-B-BBABBBBBAAAAAAAA

*000045_00815624 AAABAA-BA--BABAAA-BABAAABAAAAA-BAABBBABAB---B-BB-ABABBABABB-ABBABBAB-B-B-BBABBBBBAAAAAAAA

*000046_00149553 A--B-ABBABB-ABAB-ABAB-ABBBBBABB-AAAA--ABAA-BA-BB-AAAB-A-AAAA-BBBB-ABBBAABABAAABAABBBBAAAA

*000046_00452826 AA-B-ABBABBBABABBABABAABBBBBABAAAAAAA-A-AAABABBB-AAABAABAAAA-BBBB-ABBBAABAAAAABAABBBBAAAA

*000046_00525034 A--B-ABBABB-ABABBABABAABBBBBABAAAAAAA-A-AAABABBB-AAABAABAAAA-BBBB-ABBBAABAAAAABAABBBBAAAA

*000046_00701859 AA-BBABB-BBBABAB-ABAB--BBBBBABA-AAAAA-A-AA--ABBBBAAABAA-AAAA-BBBB-ABBBAABAAAAABAAB--BAAAA

*000047_00259538 BAAAABAAAABAAABAAAAABAAAAAABBAABBA-BBAAAAABAABABBABAAABAAABABBBBABBAAABBAAABBBAA-AAAAAAAA

*000047_00569517 BAAAABAAAABAAABAAAAA-AAAAABBBA-BBABBBAAAAABAABABBABAAABAAABABBBBABBAAABBAAABBBAABAAAAAAAA

*000047_00699891 BAAAABAAAABAAABAAAAABAAAAABBBAABBABBBAAAAABAABABBABAAABAAABABBBBABBAAABBAAABBBAABAAAAAAAA

*000048_00063975 B-BA-BBBAB-BBBAB-B-ABA-B-BB-A-BABA-AA-BBABABB--BB--A--ABAABBBBBAAB-AB---BABABBBA-AAB-BBA-

*000048_00185590 BBBABBBBABABBBABABBABAA-ABBAABBABABAABBB-BABB-A-BA-ABBABAABBBBBAABAABBBABA-AB-B-AAABB-BAA

*000048_00616769 BBBAB-BB-B--B-ABABBA-A-BABB-A-B-BA-AABBBBB-BBAABBABAABABAABBBBB-ABAABB-ABA-AB--AAAABBBBAA

*000048_00854371 B-BABBBBABAB-BABABBA--ABABBAAB-ABABAAB-B-BABB-ABBA-A-BABAABBBB-AABAABB-ABABAB-BAAAA-B-BAA

*000048_00872390 BBBA-BBB--ABBBABAB-AB---ABBAAB--BABAABBB-B-BB-ABBA-A-BAB-ABBBBBAABAABB-ABABAB-BAAAAB-ABAA

*000049_00505950 B-ABABAB-ABBBBBBABBBBBAAAABABB-ABBBBBAABABABABBAABAB-AABABAB-ABABBAAABAABABBAB-AA--ABBBBB

*000049_00528421 B-ABABAB-ABBBBBBABBB-BAAAABABB-ABBB-BAABABABA-BAABAB-AABABAB-ABABBAAAB-ABAB-ABB----ABBBBB

*000049_01010057 B-ABABAB-ABBBBBBABBBBBAAAABABB-ABBBBBAABAAABABBAABAB-AABABAB-ABABBAAABAABAB-AB-AA--AABBBB

*000050_00000473 B-BBBBB-BABABAB-ABABABAABBAAABABBBBAABBBBBABAAABAAAABAAAAAAAABABAAA-BAABB-AAAABBBBBBBBBAA

*000050_00037311 BABBBBB-BABABAB-ABABABAABBAAABABBBBAAB-BBBABAAABAAAABAAAAAAAABABAAA-BAABB-AAAABBBBBBBBBAA

*000050_00446724 BABBBBB-BAAABAB-ABABABAABBAAABABBB-BABBBBB-BAAABAAA-BAAAAAAAABABA-ABBA-B--A-AABBBBBBBBBAA

*000050_00653946 B--BB-B-BAA-B-BBABABABAABBAAA-ABBBBBABBB-B-BAAAB-AAA-AAAAAAA-BABAAA--AA-B-AAA-BBBBBBBBBAA

*000050_00738285 BABBBBB-BAAABAB-ABA-ABAABBAAABABBBBB-BBBB-ABAAABAAAABAAAAAAAABABAAB-BAABB-AAAABBBBBBBBBAA

*000050_00778103 BABBBBB-BAAABAB-ABABABAABBAAABABBBBB-BBBBBABA-ABAAAABAAAAAAAABABAAB-BAABB-AAAABBBBBBBBBAA

*000051_00019895 --BABABBBAAABABAABABABAA-BAAABABBBB--BBB-BAB-ABAAAAABABBABBAABABAAB-BAABBAAAABBBABBBBBBAA

*000051_00035531 -ABAB-BBBAAABABA-BABABAA-BAAABABBBB---BBBB-B-ABAAAAABABBABBAABA-AAB-BAABBAAAABBBABBBB-B-A

*000051_00183131 -ABAB-BBBAAABABAABABABAA-BAAABABBBB--BBBBBAB-ABAAAAABABBABBAABABAAB-BAABBAAAABBBABBBBBBAA

*000051_00251662 -ABAB-BBBAAABABAABABABBA-BAAABABBBB--BBBBBAB-ABAAAAABABBABBAABABA-B-BAABBBAAABBBAB-BBBBAA

*000051_00260083 B-BAB-BBBAAABABAABA--BBA-BAAAB--BBB--BBBBB-B-AB-AAAABABBABBAABABA-B-BAABBBAAABBB-BBBBBBAA

*000051_00405387 -ABAB-BBBAAABABBABABABBA-BAA-BABBBB--BBBBBABAABAAAAABABBABBAABABA-B-BAABBBAAABBBABBBBBBAA

*000051_00469872 -ABAB-BBBAAABABBABABABBA-BAAABABBBB--BABBBABAABAAAAABABBABBAABABA-B-BAABBBAAABBBABBBBBBAA

*000051_00661984 --BAB-BABAAABABBABAB-BBA-BAAAB-BBBBB-BABBB-BAABAAAAABABBABBA-BABAABB-A-BBBAAABBBAB-BB--AA

*000053_00017077 BABBBBABBA-BBBABABBAA-ABABABABABAAABBBBBABBBBA--BB-A-ABBBBABAAABA-BAA-B-BBAABAA-B--BBBBAA

*000053_00211163 B-BBBBABBA-BBBABAB-AABABABABABABAAABBBBBAB-BBA-BBB-A-ABBBBABAAABA-BA-AB-BBAABAA-B--BBBBAA

*000053_00687510 BABBBBA-BAABBBABBB-AABABABABABAAAAABBB-BABBBB-ABBB-A-ABBBBAAAAABA-BAA---BBAABABBB-AB-BBAA

*000053_00701201 B-BBBBABBAABBBABBB-AABABABABABAAAAABBBBBABBBB--BBB-A-ABB-BAAAAABAABA--B-BBAABAB-B-ABBB-AA

*000054_00628120 A-B---BB-BBB--BBBABAAAAABAB-AB-BAB---AA-ABBBAAABA-BBAA-BBBBBBBA-ABAB--BA-ABA--AAA-A-ABBBB

*000055_00063093 BABABBBAAABAABA-BAABABAAABABBBAAAB-AABA-BABAABABAB-BBABABBBABAABB-AAAABABBAB-AAAABBAAAABB

*000055_00189092 BABABBBAAABAABA-BAABABAAABABBBAAABAAABABBABAABABAB-BBABABBBABAABB-AAAABABBAB-AAAABBAAAABB

*000055_00194164 BABABBBAAABAABA-BA--ABAAA-ABBBA-ABAAABABBA--ABABAB--BABABBBABAABBBAAAABABBAB-AAAABBAAA-BB

*000055_00253373 BABABBBAAABAABA-BAABABAAABABBBAAABAAABABBA-AABA-ABABBABABBBABAABB-AAAABABBAB-AAAABBAAAABB

*000055_00430177 BABABBBAAABAABA-BAABABAAABABBBAAABAAABABBABAABABAB-BBABABABABAABB-AAAABABBAB-AAAABBAAAABB

*000055_00620098 BA-ABBBA-ABAABA-BAAB-BAA-AABBBAAAB-AABABB--AABA-ABBBBABAB-BA-AABBAAAAA-ABBAB--AA-BBA-AABB

*000055_00772864 BABABBBAAABAABA-BAABABAAAAABBBAAABAAABAB-ABAABABAB-BBABABABABAABB-AAAABABBABBAAAABBAAAA-B

*000055_00788231 --BABBBA-ABAABA-BAA-ABAAAAABBBAAAB-AABA-BABAAB-BA--BB-BABABABAABBBAAAABABBABBAAAA-BAAA-BB

*000055_00868791 -ABAB-BAA-B-ABAABAABA-AA-AB-BB-AAB--ABAB--BAABABABBBB-BABABA-AABB-AAAABABBABBAAA-BBAA--BB

*000056_00110544 BAAAABBAAAB-BAABAB-ABAA-BABBAABBBB-BBAABAB-B-BAAAAABAAABBAA-BBBBABABBBAABBBBABBABAABBABBB

*000056_00121093 BAAAABBAAAB-BAABABAABAA-BABBAABBBBBBB-A--BBB---AAAABAAABBAABBBBBABABBBAABBB-ABBABAABBABBB

*000056_00559559 --A--BBAABBBBAABABAABAA-B--BA-BBBB--B-A-ABBB---AA-A-AAABB-AABBBBABABBB-ABBB-AB-A-AA-B-B-B

*000056_00671710 BAAAABBAABBBBAABABAABAAABA-BAABBBB-BB-A-AB-B-B-AAAABAAABBAAABBBBABABBBAABBBBABB-BAABBABBB

*000056_00688313 B-AAABBAABBBBAABABAABAA-BABBAABBBBBBB-A-ABBB-B-AAAABAAABBAAABBBBABABBBAABBBBABBABAABBABBB

*000056_00694795 B-AAABBAABBBBAABABAABAA-BABBAABBBBBBB-A-ABBB-B-AAAABAAABBAAABBBBABABBBAABBBBABBABAABBABBB

*000057_00004973 BBBBBBB--BBAABBABAAABABA-ABAABBAAAABBBAABB-BAB-BBBB-B-BABABB-AA-AABBBBB-ABBAAB-BBAA--BAAA

*000060_00406390 B-AAABABABAB-BAB-BAB-BAB-AAABAAA-BAABABBA-BABAA-AAB-A-A---B---BABBAB-B-BBAAAAABB-AA-B-BAA

*000062_00072532 BAABABAAAAABB-ABBBAAAAAABAABBABBAA-BBAABAA-ABABBBBAAAAAAAA-AAAAABBBBB--BAABBAA-BBAAA-ABAA

*000062_00129460 BAAB-BAA---BB--BBBAAA--ABAABBAB-A-A-BAABAA-ABABBBBAAAA-AAA-AAAA--BBBB-ABAABBAAA-BAAAA-BAA

*000062_00273351 BAABABAAAAABB-ABBBAAAAAABAABBA-BAAABBAABAABABAABBBAAAAAAAA-AAAAAB-BBBAABAABBAA-BBAAA-ABAA

*000062_00451118 BAABABAAAAABB-ABBBAAAAAABAABBABBAAABBABBAA-ABAABBBAAAAAAAA-AAAAABBBBBAABAABBAA-BBAAA-ABAA

*000063_00575594 BA--BBBBBBA-AABBBAABBBBBABBABAB----A-BA--B-AA-ABBBBAAABBBAA-BAB-BBAAAAB-BABBA--B--BA--BAA

*000063_00591174 B-BABBBBBBABAAB--AABBBBB-BBABABAA-AAABA-BBAA-A-BBBBAA-B-BAA-BABABBAAAA-BBA-BAAABBB-AAB--A

*000063_00744729 BABABBBBBBABAABBBAABBBBBABBABABAABAAABABBBAAAAA-BBBAAABBBAA-BABA--AAAABBBABBAAA--BBAABBAA

*000064_00199144 BBBABBA-ABB-BABBBBAA-ABB-AAAAAB-AAAAABBBA--AAA-BAABAB-BBABAABAAA-BAABABBA-BA-AAB-BBBAABAA

*000064_00271875 B-BA--A-ABBAAABBB-AAAA-BAAAAAAB--AA-ABBBABB-A--BBA-ABBBBABAAB-AA-BAABAA-ABBA-AABBBBB-ABAA

*000064_00278384 B-BABBA-ABBA-ABBB-AA-ABB-AAAAABAAAAAABBBABBAAA-B-A-ABBBBABAABAAAABAABAABA-BAAAABBBBBAA-AA

*000064_00431717 BBBABBA-ABBA-ABBB-AAAABB-AABAABAAAAAABBBABBAAA-BAA-ABBBBABAABAAA-BAABAABA-BAAAABBBBB-A-AA

*000064_00454476 BBBABBA-ABBABABBB-AAAAB-A-ABAABAA-AAABBBAB-A-A-BAA-ABBBBABAABAAA-BAABAABA-BAAAABBB-BA--AA

*000064_00660591 BBBABBA-ABBA-ABBB-AAAABB-AABA-BAAAAAABBBABBA-A-BAA-ABBBBABAABAAAABAABAABA-BAAAABBBBBAA-AA

*000065_00172720 B-B-BBBBAAABB-BAAB-B-AAABBBBBAB--AA-ABAABA-BBABBBABAAABBBABBBA-AAB-BB-BAAAA--A-AABBBA-AAA

*000065_00192001 BABBBB-BAAABB-BA--ABAAAABBBBBABAAAAAABAABAABBABBBABAAABBBABBBAAAAB-BB-BAAAABAA-AABBBABAAA

*000065_00197374 BABBBB-BAAABB-BA-BABAAAABBBBBABAAAAAABAABAABBABBBABAAABBBABBBAAAAB-BB-BAAAABAA-AABBBABAAA

*000065_00513107 B-BBBB-B--ABB--A-BA-AAAABBB-BAB-AAAAABAABAABB-BBBABAAAB--A-BBAAAAB-BBAB-AAA-AAAB-BBBA-AA-

*000065_00679571 B-BBBB-BAAABBBBA-BABAAAABBBBBABAAAAAABAAAAABBABBBABAAABB-ABBBAAAAB-BBABBAAABAA-B-BBB-BAAA

*000065_00885223 BABBBB-BAAABB-BAABABAAAABBBBBABAAAAAABAAAA-BBABBBABAAABBBABBBAAAABBBB-BBAAABAA-BABBBABAAA

*000066_00047948 -BBAB-BBAAAABBABABBAAABAABBBABAAAAAAAAB-AAAABBBAAABBBA-BBABABABABAB-BAAAAAAAAAAABBBAAABBB

*000066_00145235 -BBAB-BBAAAABBABABBAAABAABBBABAAAAAAAAB-AAAABBBAAABBBA-BBABABABABAB-BAAAAAAAAA-A-BBAA-BBB

*000066_00478099 -BBAB-BBAAAABBABABBAAAB-ABBBABAAAA-AAAB--AAABAB-AABBBABBBABABABABAB-BAAAAAAAAA-ABBBAAABBB

*000066_00619935 -BBAB-BBAAAABAABBBBAAAB-ABBBABA-AAAAAABBAAAABAB-AABBBABBBABABABAB-B-BAAAAAAABA-ABBBAAABBB

*000067_00192838 BAAAAB-AAABBAAABAABABAA-BA-AAAB-BBBAABAB--BBBBBAABABAAAABABBBABBBBAAABAABBBB-BAABAABBABBB

*000067_00230894 BBAAABBAAABBAAABAABABAA-BA-AAAB-BBBAABABABBBBBB-ABABAAAABABBBABBBBAAABAABBBB-BAABAABBABBB

*000067_00391618 BBAAABAAAABBAAABBABABAA-BA-AAAB-BBBAABAB-BBBBBBAABABAAAABABBBABBBBAAABAABBBB-BAABAABBABBB

*000067_00425762 BBAAABAAAABBAAABBABABAA-BA-AAAB-BB-AABABABBBBBB-ABAB-AAABABBBABBBBAAABAABBBB-BAABAABBABBB

*000068_00078918 AAABAAABAAABB-BAABBABABABBBBABBBAABBBA--BA--BBBA-BBABAABABBAABBABBAB-AAAABBBAAAABAAAAAAAA

*000069_00155386 BA-B-BABAAB-BAABA--AA-BAAAABAB---AA-ABBBA--BBAABBBBAAAB--BAB-BABABBAAABB-BBAAAA-BBBBB-BAA

*000069_00253611 B--BBBABAABBBAABABBAABBA-AABABB-AAA-ABBBAB-B-A--BBBA-ABBB-AB-BAB--BAA-B-B-B--AA-BBBB-B-AA

*000069_00349167 BABBBBABAABBBAABABBAABBAAAABABBBABAAABB--BBBBAABBBBAAABBBBABABABABBAAABBBBBA-AA-BBBBBBBAA

*000069_00515197 B-BBBBABAABBBAABABB-ABBAAAABABBBABA-ABB-ABBBBAA-BBBAAABBBBABABABABBA-ABBBBBB-AA-BBBBBBBAA

*000069_00625116 BABBBBABAABBBAABABBAABBAAAABABBBABAAAB--ABABBAABBBBAAABBBBABABABABBAAABBBBBBAAA-BBBBBBBAA

*000069_00684068 BABBBBABAABBBAABABBAA-BAAAABABB-AB-AABB-AB-BBAABBBBAAABBBBABAB-BABBAAABABBBBAAAA-BBBBB-AA

*000070_00190981 B-AAABBBAAAAAAAB-ABBBAABBABABBB-ABABBB-BA-BABBABAABABBBABBAABA-BA-ABBB-B-ABAABBBAAAB--AAA

*000070_00256380 BA-AA-BBAAAAA-ABAABB-AAB-ABABB---BAB-B--AB-ABBABAABA--BAB-A-BAAB--ABBBAB-ABAABBBAAABBAAAA

*000070_00287376 BBAAABBBAA-AAAABAAB---ABBABABBBAAB--BB--ABBA--ABAABABBB--BAA--AB--A----BBABAABBBAAAB-AA-A

*000070_00364719 BAAAABBBAAAAAAABAABB-AABBABABBBAABABBB-BABBABBABAABABBBABBAABAAB--ABBB-B-ABAABBBAAABBAAAA

*000071_00001350 BAAAABABBBBBBBBBABAAAAAAAABAAABBBBBBBBABBBBAA-AABAABBBAAAABABBBB-AABBABB-BBAB-ABBBBABBBBB

*000071_00001436 BAAAABABBBBBBBBBABAAAAAAAABAAABBBBBBBBABBB-AAAAABAABBBAAAABABBBB-AABBABB-BBAB-ABBBBABBB-B

*000071_00239035 B-AAABABBABBBB-B-BAA--AAAABAA-BBBBB-BBABBB-AAAAAB-ABB-AAAABABBBB-AABBA-B--BA--A-BB-A-BBBB

*000071_00360626 BBAAABABBABB-BBBABAAAAAAAABAAABB-BBBB-ABBB-AAAAAB-ABBBAAAABABBBB-AABBABB-BBAB-ABBBBABBBBB

*000071_00633623 BBAA-BAB-ABBBBBBA-AAAAAAAABAAABBBBBBBBABBB-AAAAAB-ABBBAAAABABBAB-AABBABB-BBAB-ABBBBABBBBB

*000071_00701276 BBAAAB-BBABBBBBBABAAAAAAAABAAABBBBBBBBABBBBAAAAAB-ABBBAA-ABABBAB-AABBABB-BBABA-BBB-ABBBBB

*000072_00226325 B--AABBA-B-BBABBABAAABABBABBAABA-B-BBAB--BBABBBAA-ABAAABAAAABBA-AAABBBABAABBBBBBBAABBAB-B

*000072_00281757 B-AAAB-AABBBBABB-BAAABABBABBAABAABA-BABAA-BABBBA--ABA-A-AAAABBAB-ABBBB-BAAB-BBBBBAABBABBB

*000072_00506715 B-AAABBAABBBBABBABAAABABBABBAABA-BABBABAABBABBBAA-ABABABAA-ABBABAABBBBABAAB-BBBBBAAB-A-BB

*000072_00542472 B-AAABBAABBBBABBABAAABABBABBAABA-BABBABA-BBABBBAA-ABABABAA-ABBABAABBBBABAABBBBBBBAABBABBB

*000072_00624799 BABABB-AABBBBABBABAAABABBABBAABA-BABBABAABBABABAA-ABABABAA-ABBABAABBBBABAABBBBBBBAABBABBB

*000073_00097991 BABAB-BB-A-AABA-AAAB-BAAABABBBABABA-ABABAA-AABAAABBBBABABBBABAABB-AAAABAB-AB-AAAABBAAAABB

*000073_00203683 BABABBBBAABAABA-AAABABAAABABBBABABAAABABAA-AABAAABBBBABABBBABAABB-AAAABABBAB-AAAABBAAAABB

*000073_00371648 BABABBBBAABAABA-AAABABAAABABBBABAB-AABA-AABAABAAABBBBABABBBABAABB-AAAABABBAB-AAAABBBA-ABB

*000073_00557675 BABABBBBAABAABA-AAABABAAABABBBABABAAABABAABAAAAAABBBBABABBBABAABB-AAAABABBAB-AAAABBBAAABB

*000073_00640571 BBBABBBBAABAABA-AAABABAAABABBBABABA-A-ABAABAA-AAABBBBAB-BBBABAABBBAAAABAB-AB-AAAABBB--ABB

*000074_00469048 ABBBBABAA-BBBABAABAA-BABAABABB-ABBBBBBBAAABABBABBA-AB-ABBAAAA-AB-BB-ABBBBA-BBBABBBBAAA-AA

*000074_01129154 A--BBABAA-BBAABAAAAAA-ABAABABBBBBB-BBBBAAABA-BABBAAA--ABBAAAAAAB-BB--B-BBA-BBBABBBB-----A

*000075_00513497 -ABAB--AAB-BAAABBAAABABABABA-BBABABBBB-BBABBAB--BB-ABA-BAAABBAAABBBBBAB-AABBABA-ABBBAAAAA

*000075_00513583 BABAB--AAB-BAAABBA-AB-BABABA-BBABABBBB-BBA-BAB--BBBABA-BAAABBAAABBBBBAB-AABBABA-ABBBAAAAA

*000075_00515412 -ABAB--AAB-BAAABBAAABABABABA-BBABABBBB-BBABBA---BB-ABA-BAAAB-A-ABBBBBAB-AABBABA-ABBBAAAAA

*000075_00658497 BABABB-BBAABAABAAAAABAAAAABBBBBAABAA-AABABA-AABBABAAABAABAAAABBBBBAA-AABAABAAAABBAAABAAAA

*000076_00031394 -BB-B-BBABABBAABBBBBBAB-ABBBBB--AAAAABB-AA--BABBAABBBABBABBABABAB-A-BAAAAAAABABABBBAAABBB

*000076_00137106 -BB-B-BBABABBAABBBBBBAB-ABBBBBABAAAAABBBAA-ABAB-AABBBABBABBABABAB-A-BAA-AAAABABABBBAAABBB

*000076_00220097 -BBAB-BBABABBAABBBBBBAB-ABBBBBA-AAAAAABBAAAABAB-AABBBABBABBABABAB-A-BAAAAAAABABABBBAAABBB

*000076_00303088 --BAB-BBAAABBAABBBBBBAB-ABBBBBA--AAAAABBAAAABAB-AABBBABBABBABABAB-AABAAAAAAABABABBBAAABBB

*000076_00692112 -BBAB-BBAAAABAABBBBBAAB-ABBBABABAAAAAABBAA-ABABAAABBBABBABBABABA--A-BAAAAAAABABA-B-AAABBB

*000076_00735273 B-BAB-BB-AAABAABB-BBAAB--BBBAB-AAA--A-BBAA-AB-BAAABBB-BBBA-AB-BABAB-B-A-AAAABAAABBB---BBB

*000077_00017244 AABABABABBBBABBAAABABBBBAABAABBBAAAAABABBB-BAA-BBBBABABBBABBAABAAABBBAABBBBAA-BBBAAABBBAA

*000077_00173906 AABABABABBBBABBAAABABBBBAABBABBBAAAAABABBBBBAA-BBBBABABBBABBAABAAABBBAABBBBAA-BBBAAABBBAA

*000077_00265268 AABABABABBBBABBAAABABBBBAABBABBBAAAAABABBBBBAA-BBBBABABBBABBAABAAABBBAABBBBAA-BBBAAABBBAA

*000078_00199497 B-ABABABA-BBABBABA-BBABABBAABBBABBBAAAABABBABBABBBBABABAA-BBAABBAABBAA-BAABABAABBAAAA-AAA

*000079_00143022 B-BABBBA-BB-B--BBBBAABAAB-B-BBB-AAABBBBB-AAAABABABA-ABAA-BAB-BABBAB-AABBBBABBBAABBBBBABBB

*000079_00188853 BABA--BA-BBABAAB--BAABAAB-BABBB-AA-BBAB-AA-A-BAB-BABA-AA-BABA-ABB-BBAA-BBB-BBBAABBB--ABBB

*000079_00755099 BAB-BBBAABBABAABBBBAABAAAABABBB-AA-B-ABBAAA-ABABABABABA-BBAB-BABBAB-AABABBABBBAABBB-BABBB

*000079_00778622 BAB-BBBAABBABAABBBBAABAAAABABBB-AAAB-ABBA-A-ABABABABABAABBAB-BABBAB-AABABBABBBAABBB-BABBB

*000080_00002610 BBAAABABBBBB-BBAA-AA-AAAAABAAAA-BBBBBBBBBB-AAAABBABABBAAAABABBABBAABAA-BABAABAABBBBABBBAA

*000080_00527836 BBAAABAABABB-BBAA-AA-AAAAABAAAA-BBBBBBBBBBBAAAABBABAABAAAABABBABB-A-AA-BABAABAABBBBABBBAA

*000082_00038306 BABABB-AAAABB-BBABABBAAABBBBBABAAAAAABAAAAABBBBBBB-AAABB-A-BBABBBBABBBBBABBBAABBABBAAAAAA

*000082_00112843 BABABB-AAAABB-BBABABBAAABBBBBABAAAAAABAAAAABBBBBBB-AAABB-A-BBABBBBAB-BBBABBBAABBABBAAAAAA

*000083_00321165 BAA-A-BBABABBB-BBB-B--AAB---A-A--BBB--B-BB-BAB-BBAB-AABBBBBB-A-BA-ABAAABBAABAB-B-AAA-BBBB

*000083_00323646 B-ABABBBABABBBABBBBB-BAABBB-AAAABBBBBABABB-BABBBBABBAABBBBBBBA-BA-ABAAABB-ABAB-BAAAABBBBB

*000084_00228064 B-BABBBBBBBBB-BBB-BB-B-BABAA-BABBBBAABBA--BAAAABB-BAB-B--BBABAABB-ABBAABAAAABAABBBBABABAA

*000084_00367259 BBA-ABAABAABBBBBABABBAAAAAAABBABBB-AABBABBBAAAABBABAABAAAABABAABB-ABAAABAAAABAABBBBABABAA

*000085_00198496 BABABBAABBBAABABAAAABB-BB-ABBA-BBBBBBB-A-ABBAABBAAABAABBAABABABAA-ABB-A-ABBBBAABAAABB-ABB

*000085_00209525 BABABBAABBB-ABABAAAABB-BB-ABBABBBB-BBBBABABBAABBAAABAABBAABABABAABAB-BABABBBBAABAAABB-ABB

*000085_00575068 BABA--AA-BBAA--BAAAA--AB-AABBAB-BB--B--A-A--AA--AAABAAB-AA-AAABAABABABABA-BBBAABAAABBAABB

*000086_00198483 B-BBBBAAAB-A-ABB-B--BABBBABBBABBBBBBABAA-A--BABABBAAA-B-BBBAA-AAA-AAA-AABBBA-AAA-B--BABBB

*000086_00248993 B-BBBBA-ABAABABB-BA--AB-BABBBABBB-BBABAA-A-BB-BABBAAA-BABBBAAAAAA-AAABAABBBAAAAA-BBBB-B-B

*000086_00426301 B-BBBBA-ABAABABB-BA--AB-BABBBABBBBBBABAA-ABBBABABBAAAABABBBAAAAAAAAAABAABBBAAAAABBBBBABBB

*000087_00387320 BBBABBAAABA-AABBBAABAAABBA-ABAABABBBB-BBBAABABA-ABA-ABABBABAAAABAAAAAABAAABABBAABAAAAAAAA

*000087_00555962 BBBA-BA-ABABA-B---A-AAAB--AABAAB-BBBBBBBBA-BABABABA-ABABBABAAAABAAAAAAB-AABABBAAB-AAAA-AA

*000087_00578017 BBBABBAAABABAABBBAABAAABBA-ABAABABBBBBBBBAA-A-A-ABA-ABABBABAAAABAAAAAABAAABABB-ABA-AAAAAA

*000088_00248943 BBABAB-BAABBAB-ABAABBABBBBBBBBB-BBBAAAABAB-BBAAB-BBAAAAAABABAABBA-B-AA--AABBBAABBBBAABAAA

*000088_00308207 BBAB--AB-ABB-B--BA-BB----BB-BBB-BB-AAAABAB-BB-AB-B-AA-AA--ABAABBAAB-AA-BAABBBA-BBBBAABAAA

*000088_00308293 BBABABAB-ABBAB-ABAABBABBBBBBBBB-BBBAAAABABBBBAAB-BBAAAAAABABAABBA-B-AA-BAABBBAABBBBAABAAA

*000088_00524956 B-AB--ABAA--ABBABAA-BABBB-BBBBBBBBB-AAABAB-BB--BBBBA-A--AB-BA-BBABB--ABBAAB-BAAB--BAABAAA

*000088_00539329 BBAB-BABAABBAB-ABAABBABBBBBBBBB-BBB-AAABABBBBBA-BBBAAAAAA-ABAABB--B-AA-BAABBBAAB-BB-ABAAA

*000088_00596673 BBABABABAAB-AB-ABAAB--BBBB-BBB-BBBBAA-ABA-BBB-ABBBB-AAA-ABA--A-B--BB-A-B--BBBAA--B-AA--AA

*000089_00203818 ABAAAAABAABABABBABAAAABAAABAAA-BBBBBBB-BBB-AA-AAA-ABBBAAAABAABABBAABBAABBABAB-ABABBBBA-BB

*000089_00317029 A-AAAAABAABABABBABAAAABAAABAAABBBBBBBBABBBBAABAAAAABBBAAAABAABABBAABBAB-BABAB-ABABBB-ABBB

*000090_00185327 BABABBBABBBAABB-BAABAAAAAB-BBBAAABAAABABBABAABABBA-BBABBBABA-A-BA-AAAA-ABA-BBAAAAAAAA-ABB

*000090_00466833 BAAAABB-BBBAAAB-BAABAAAAAB-BBBAAABAAABABBAB-A-ABBA-BBABBBABA-A-BAAAA-ABABA--BA-AA-AA-AABB

*000090_00572557 BAAAABBABBBAAAB-B-ABA-AAABBBBBA-ABAAABABBABAABABBA-BBABBBABA-AABA-AAAABABA-BBAAAAAAAAAABB

*000090_00572643 BAAAABBABBBAAAB-BAABAAAAAB-BBBAAAB--ABAB-ABAABABBA-BBABBBABA-AAB--AAAAB-BA-BBAAAAAAAA-ABB

*000091_00138155 A-AAAA--BAAABBA-BBBBBBBBB-BBBAAA-A--BBABABBAABAABBBBB-ABBBBAABABBBBBBBBABA-AAABAABB-A-ABB

*000091_00145515 AAAA-A-B-AAABBA-BBBB-BBBBBBBBAAA-A-BBBABAB-A-BAABBBBBBABBBBAABABBBBBBBBABABAAABAABBAAA-BB

*000091_00819967 AAAAAA--BAAABBA-BBBBBBBBB-BBBABA-A-BBBABABBA-BAABBBBBBABBBBAABABBBBBBBBABA-AAABAABBAAA-BB

*000091_00852092 A-A-AABABAAABBA-BBBBBBBBB-BBBABA-A-BBBABABBA-BAABBBBBBABBBBAABABBBBBB-BABA-AAABAABBAAA-BB

*000093_00056447 BAAAABABAABAAA-AAAAABAAAAABBBAABBABBBAAAAABAAAABBABBBABAAABABBBB-BBAAAB-AAAABBAABAAAAAABB

*000093_00087932 BAAAABABAABAAA-AAAAABAAAAABBBAABBA-BBAAAAABAAAABBABBBABAAABABBBB-BBAAAB-AAAABBAABAAAAAABB

*000093_00130914 BAAAABABAABAAA-AAAAABAAAAABBBBABBABBBAAAAABAAAABBABBBABAAABABBBB-BBAAAB-AAAABBAABAAAAAABB

*000093_00193499 BAAAABABAABAAA-AAAAABAAAAABBBBABBABBBAAAAABAAAABBABBBABAAABABBBB-BBAAAB--AAABBAABAAAAAABB

*000094_00077391 --BABBAAAAABB-BBABBBBAAAA-BBBABAAAAA-BAAAA-AB-BBBB-ABABB-A--BABBBBABABBBAB-BAA-BABBAB-AAA

*000094_00658688 B-BABBAAAAABBBBBABAB-AAAABBBBABAAA-A-BAA-A--BBBBBB-AAABB-AABBABBBBABBBBBABBBAABBABBAAAAAA

*000095_00131692 BABBBBAAAAAB-BAAB-BAA-BB-BBAAAABAAAABABAABAAAA-AABAA-BAABAAAABAAAABABABBBAABBBBB-BBAABAAA

*000095_00488616 BABBBBAAAAAB-BAAB-BAA-BB-BBAAAABAAAABABAABAAAA-AABAA-BAABAAAABAAAABABABBBAABBBBBABBA-BAAA

*000096_00241888 BBA-ABBBAABABBBBABAABBBABABABBBABBBBBAABBBBBBBB-AAAABABAABBBBABB-A-ABBB-BABAAAB-ABBA--AAA

*000096_00264828 BBAAABBBAABABBBBABAABBBABABABBBABBBBBAABBBBBBBBAAAAABABAABBBBABB-AA-BBB-BABAAABBABBAABAAA

*000096_00334248 BBAAABBBAABABBBBABAABBBABABABBBABBBBBAABBBBBBBBAAAAABABAABBBBABB-A-ABBB-BABAAAB-ABBAABAAA

*000096_00369927 B-AAABB-AABABBBBABAABBBABABABBBABBBBBAAB-B-BB-BAAAAABABAABBBBABB-A-ABBA-BABAAAB-ABBAABAAA

*000097_00124185 B--ABBBAAB-ABA--BBBAABAAAAA-BBB-AAABBBBB-A-A-BABABABAAAABBABBBAB-ABB-ABABBAABBAABBBBB-BBB

*000097_00260494 BBBABBBAABB-BA-BBBBA-BAAAAAABBB-AA-BBBBBAA-A-B-BABABA-AABBAB-B-BBAB-AABABBA-BB-A-B--B-BB-

*000097_00337857 BBBABBBAABBABAABBBBAABAAAAAABBB-AAABBBBB-AAAABABABABAAAABBAB-BABB-B--ABABBAABBAABBBBB-BBB

*000098_00123925 AAAAAAAAABBABA-AAA-ABAABBBBAAA-BAAABB-BAA--BB-BAB-ABA-ABAAAABAABAAA-AB-AA-AAA-AAB-BBB-BBB

*000098_00225868 ABAAAAAAABBA-ABA--BBBAABBBBAAABBAAABB-BAABBBBBBABBABABBBAABBBBBBAAABABAAABA-A-AA-BBBB-BBB

*000098_00635102 AAAAAA-AABB---BABABBA-ABB-BAAAB--AABBBBA-BBBBBBABBABAABBAAAA-AABABABA--A-BA-A-A--BBBB---B

*000099_00163331 B-A-A--BA-BABABAAB-BAABAAAABBBBBBB-AAB-BAB-BBB-BBBBB--ABABBBB-ABAB--ABBABABBBAA--BBB--ABB

*000099_00394168 B-ABABB-ABB-BABAABBBAABAA-ABBB-B-BBAABBBAB-BBBBBBB-B--ABAB-B-BABABA-A-B--A--BAA--BBBBB-BB

*000099_00538351 BBA-ABBBABB-BABAABBBAABAAAABBBBBBBBAABBBABABB-BBBBBBA-ABABABB-ABABB-ABB-BABBBAA-BBBBBBABB

*000099_00565344 B-A-ABB-ABB-BABAABBBAABAAAABBBBBBBBAABBB-BABB-BBBBBBA-ABA-A-BBABA-B-AB--B-BBBA--BBBBBBA-B

*000099_00596928 BBA-ABBBABB-BABAABBBAABAAAABBBBBBBBAABBBABABBABBBBBBA-ABABABB-ABABB-ABB-BABBBAA-BBBBBBABB

*000100_00009348 -ABAB--ABB-BABABBAAABABABABAABBABBBBBB-BBABBAB--BBAABABBAAABBAAABBBBBAB-AABBABA-ABBBAAAAA

*000100_00009961 -ABAB-AABB-BABABBAAABABABABA-B-ABBBBBB-BBABBA---BBAABABBAAABBAAABBBBBABBAABBABABABBBAAAAA

*000100_00220497 --BABA-ABB-BABABBAAAB-BABABAABB-BBBBBB-BBA-BA---BBAAB--BAAABBAAABBBBBAB-AABBABA-ABBBAAAAA

*000100_00264152 -ABAB--ABB-BABABBAAABABABABAABBABBBBBB-BBABBA---BBAABA-BAAABBAAABBBBBAB-AABBABA-ABBBAAAAA

*000101_00497385 A-AAAABB-AABABBAAAABBBBBBAA-BAAABBBAAABBBAABBBBAAABAABBAB-BAAABAA-ABBBAAAAABABB-BBBBABAAA

*000101_00582947 AAAAAABB-AABABBAAA--BBBBBAA-BAAABBB-AABBBAABBBBAAABA--BABAB-AABAA-ABBB-AAAABABBB-BBBA-AAA

*000101_00662738 A-AAAABB-AABABBAAAABBBBBBAA-BA-ABBBAAABBBAABBBBAAABA-BBABABAAABAA-ABBBAAAAABABB-BBBBABAAA

*000102_00419051 BABB-BABABBBBAABABBAABBAAAABABBBAB-AABBBABAABAABBBBBA-BBABABABABBBBAAABABBBBAAAABBBBBBABB

*000102_00487672 BABBBBABABBBBAABABBAABBAAAABABBBABAAABBBAB-ABAABBBBBA-BBABABABABBBBAAABABBBBAAAABBBBB-ABB

*000102_00721586 BABBBBBBABBBBAABABBAABBAAAABABBBABAAABBBABAAB-BBBBBBA-BBABABABABBBBAAABABBBBAAAABBBBBBBBB

*000103_00015853 A-BBBAAB-AB-BBB-BBAA-BAA-BBABAABBAB-ABB-BBAABAB-BBBBBBABBBBB-BA-BA--B--BBAAABBBB-B-B--A-B

*000103_00714638 ABBBBAABAABAABB-B-AAABAAABBABAABBABAABBBBB-AAAB-BBBBBBABBBAB-BAABAAABAABBAAABBBBABBBAAAB-

*000104_00349209 ABABAAAAABBA-ABAB-BBBAABBBBBABABAAABB-BAABBBBBBBBBBBABBBAABBBBBAABBBABAAABA-A-AABBBBB-BBB

*000104_00415620 AAAAAAAAABBA-ABABBBBAAA-BBBA-BABA-ABB-BAA-BBB-BABBABAB--AAAABAAAABABAB-AAB--ABAA-BB-BBBBB

*000104_00415706 ABABAAAAABBA-ABAB-BBBAABBBBBABABAAABB-BAABBBBBBBBBBBABBBAABBBBBAABBBABAAABA-A-AABBBBB-BBB

*000104_00484133 AAAAAAAAABBA-ABAB-BBAAABBBBAABABAAABB-BAABBBBBBABBABA-BBAAA-BAAAABABABAAABA-A-AABBBBB-BBB

*000105_00225229 A-ABAAABA-ABB--A-BBABAAABBB-ABB-AABBBABA---ABB--ABBA-AABABBAA-BAB-AB-AABABBBBAA--AA-AAAAA

*000106_00304008 ABABAABB-B-BBAAAABAAA-AB-AB-BB-ABBBBBB----BBBBABA-B-B----B-BB-ABBBB-B-BBBABAAAABABBA-BB--

*000107_00092773 -AABA-AABBA-BABABBAAA-AABAABBAAABBBAABBBBB-AB-ABBBAB-BAABAABAAABBBBBBBAABBAB-AAAAAABBB-BB

*000107_00093397 --AB--AA-BAABA-AB-A-A--A-AA-BAA--BBAABBBBB-AB--BB-ABBBAABAABAAABBBBBBBAABBAB-A-AAAAB-BABB

*000107_00280870 A--B---A-B-ABABA-BAAA-AA--ABBAAABBBAABBB-B-ABAAB---BBBAA-AABAAABBBB-BB-ABBAB-AAAA-AB-B-BB

*000108_00173831 B-ABABBBABBBBBBBBBBBBBAAABB-AB-AABABBABBBBABABBBA-AB-ABBAABBBABBA-BBAAABB-AAA-BBBAAABBBBB

*000109_00185991 BABABBAAABBA-ABBB--AAABBBAAAAABAAAAAABB-ABBAAB-B-A-ABBBBABAABAAAABAABABAA-BAAAA--BBBAABAA

*000109_00187456 BABABBAAABBA-ABBB--AAABBBAAAAABAAA-AABBBABBAAB-B-A-ABBBBABAABAAAABAABABAA-BAAAA--BBBAABAA

*000109_00281097 BABABBAAABBA-ABBB-A--ABBBAAAAABA-AAAABB-ABB-A--B-ABABBBB-BAABAAA-BA-BABAA-B-AA--ABBB---AA

*000110_00081956 BAAAABBABBAABBAAABBBBBBBBAB-BAABB-BBBABBBAA-BBAABBAABAABBABBBBBAAAABBBAABBABBBBAABBBBBAAA

*000111_00245144 BABBBBA-ABAABABB-BA-BAB-BABBBABBBBBBABAA-A-BB-BABBAAAABABBB-AABAA-AAABAABBBAAAAA-BBBBABBB

*000111_00432205 B-BBBBA-ABAABABB-BA-BAB-BABBBABBBBBBABAA-ABBBABABBAAAABABBBAAABAA-AAABAABBBAAAAABBBBBABBB

*000111_00557495 B-BBBBA-ABAABABB-BA--AB-BABBBABBBBBBABAA--BBBABABBAAAABABBBAAAAAA-AAABAABBBAAAAABBBBBABBB

*000111_00666458 BBBBBBA-ABAABABB-BA--ABBB-BBBAB-BBBBABAA-ABBBABABBAAAABABBBAAAAAAAAAABAABBBAAAAABBBBBABBB

*000112_00353111 -BB-B-BBABABBAABBBBBBAB-ABBBBBA-AAAA-BB-AAAABABBAABBBABBABBABABAB--BBAA-AAAABABABBBAA-BBB

*000112_00360198 -BB-B-BBABABBAABBBBBBAB-ABBBBBA--AAA-BB-AAAAB-BBAABBBABBABBABABAB--BBAA-AAAABABABBBAAABBB

*000112_00561863 -BB-B-BBABABBAABBBBBBAB-ABBBBBABAA-A-BB-AAAABABBAABBBABBABBABABAB--BBAA-AAAABABABBBAAABBB

*000112_00615160 -BB-B-BBABABBAABBBBBBAB-ABBBBBA-AAAA-BB-BAAABABBAABBBABBABBABABAB--BBAA-AAAABABABBBAAABBB

*000112_00683691 -BB-B-BBABABBAABBBBBBABAABBBBBA-AAAA-BB--AAAB-BBAABBBABBABBABABABB-BBAA-AAAABABABBBAAABBB

*000113_00039192 BBAAAB-BBAABBBBABBABBAABBAAABA-AABBAABAABBBBBA-BBBABAAAAA-B-BABAABBBABABBABBBABBBBBABAABB

*000113_00039278 BBAAAB-BBAABBBBABBABBAABBAAABA-AABBAAB-ABB-BBA-BBBABAAAAA-B-BABAABBBABABBABBBA-BBBBABAABB

*000113_00352588 B-AA-BAABAABBBBA-BAB-AABBA-ABAAAAB-AABAA---BBA-BBBABAAAAA-B-BAB-AB-B-BABBABBBABBBB--BAABB

*000114_00095893 BBBABBBBAABAABABBAABABAAABABBBBBBBBAAAAAAAABABAABBBBBABABBBA-AABBBAAAABABBAB-AAABBBBABABB

*000114_00231818 BBBABBBBAABAABABBAABABAAABABBBBBBB-AAAAA-AABA-AABBBBBABABBBA-AABBBAAAABABBABBAAABBBBA-ABB

*000114_00254454 BBBABBBBAABAABABBAABABAAABABBBBBBBBAAAAAAAABABAABBBBBABABBBA-AABBBAAAABABBAB-AAABBBBA-ABB

*000115_00155056 B-AAABBAB-ABBAAABBBB--ABA-ABBAABBBAAAA-ABA-BAABBAAAABAAABBBBABAAAB-BBBA-AABBAABA-AABAAAAA

*000115_00742858 BBAAABBAB-ABBAAABBBBBAABAAABBAABBBAAAA-ABA-BAABBAAAABAAABBB-ABAAAB-BBBA-AABBAABABAA-AAAAA

*000116_00067434 BA--ABBB-BBBBABBABBB-BBA-AAABAA-B-B-AAAABA-AAB-AB-A-A-ABA--BAA-ABBAAA-BBBAA-A-BA-BBAA-BBB

*000116_00407088 BAABABBB-AB-B-BBABBBABBAAABABA--BBBAAAAABB-AABAABAAB-AABA--BA-AABBAAABBBBA--AB-AABBA-ABBB

*000118_00099216 B-BBBBABAAABBAABABBABAAAAAA-ABBABBBBBABAAAAAA--BBBAAAAAB-ABABBBAAAABBABBAABABBAABBBBAAA--

*000118_00217503 B-BBBBABAAABBAABABBABAAAAAA-ABBABBBBBABAAAAAAB-BBBAAAAAB-A-ABBBAAAABBABBAABABBAABBBBAAA--

*000118_00294589 B-BBBBABAAABBAABABBABAAAAAA-ABBABBBBBABAAA-AAB-BBBAAAAAB-A-ABBBAAAABBABBAABABBAABBBBAAA--

*000118_00591853 B-BBBB-BAABBBA-BABB-BAA--AABBBB-BBBBBAB-AA-AAB--BBA-AAAB---ABBB-AAABBABBA-BABB--BBBB-AABA

*000118_00648813 B-BBBBABAABBBAABABBABAAAAAA-BBBABBBBBABAAAAAAB-BBBAAAAAB-A-ABBBAAAABBABBAABABBAABBBBAAA--

*000120_00315602 BB-AABBBA-BABBBB-BB-B--ABABABBB-AAAB-AAABB-BBABABAABBABBABBBAABABABABBBBBABBA-B-ABBAABABB

*000120_00437969 BB-A-BBBAABABBBBABBABBBABABA-BBAAA-BBAAABBABBA-ABAABBABBABBBAABABABABBBBBABBAABBABBAABABB

*000120_00439247 BB-AABBB-A-ABB-BA-BAB-BAB-BAB-B--AABBAA---ABBABABAA--AB-ABBB--BABA-ABBBBB-BB--B-ABBAAB-BB

*000121_00737552 ABBABAABAABABB-BBBBAABABBB-ABBAABA-BBBBBBB-BB-BAB-BBBABBABBB-AAABABBBBABBAABBBBBABBAABABB

*000122_00068532 B--B-BBA-ABBABBABA-BABBAB-A-AAAA-ABBBABA--AB-BB-BAAABBBBBAB-BBAABAABABBAAAAA-BBA--ABBBBAA

*000123_00368008 BABABBBAAB-AAAABBABABAAAB-AA-ABAB-BBBBAB-B-BAB-AABBAAA-BBA-BBA-ABABBBAB-BBBB--A-ABBBBBAAA

*000124_00756191 BBAAAB-BBBABB-AAB---BAABA-A-BA---BA--AAABA-BA-BBAAAABAAA-BBAABAAAB-BBBA-AAB-AABABAABA--AA

*000125_00749788 AAAAAA--BAAABBA-BBBBBBBBB-BBBABA-A-BBBAB-BBA-BAABBBBBBABBBBAABABBBBBBBBABA-AAABAABBAAA-BB

*000126_00243691 A-BABAABAABAAAAAAAABBAA-AABABBABBBBBBABBABBABAABBA-BBAAAAABAAABB-BBAAAB-AAAAABABBAAAAB-BB

*000126_00467580 A-BABAABAABAAAAAAAA-BAA-AABABBBBBBBBBABBABBABAABBA-BBAAAAABAAABB-BBAAAB-AABAABABBAAAAB-BB

*000127_00071364 B-AA-BAABBAABBAA-B--BBBBBAB-BAAA--ABBAB-BAA-BBA-BBAABBABAABABBBAAAABBB-ABAABB-BABBBAABAAA

*000127_00072224 BBAAABAABBAABBAAABABBBBBBABBB-AA-AABBAB-BAA-BBA-BBAABBABAAB-BBBAAAABB-AABAABB--ABB--ABAAA

*000127_00142361 -BAAABAABBAABBAA-BABB-BB--BBBAAA-AABBABABA--B-A-BBA--B-BA-B--BB-AAABBAAABAABB-BA-BBAA-AAA

*000127_00383073 BBAAABAABBAABBAAABABBBBBBABBBAAA-AABBAB-BAA-BB-BBBAA-BA-AAB-BBBAAAABB-AA-AABB-BABBBAABAAA

*000128_00082836 BAB-BBBBABBBB-ABABBAAABAAAABBBBBBBBAABBBABAABABBBBBBA-ABABABABABABB-ABBABABBBAAABBBBBBBBB

*000128_00153011 BAB-BBBBABB-BAABABBAAABAAAABBBBBBBBAABBBAB-ABABBBBBBA-ABABABABABABB-ABBABBBBBAA--BB-BBB-B

*000129_00322035 -BBBB-BBBAAABABAABABABAABBAAABABBBB--BBBBB-B-ABAAABABABAABAAABABAABBBA-BBAAAABBBABBBBBBAA

*000130_00232517 BABABBAABAAAAB-BBAB-BBBBAABAAABBBBBABAABBBAABA-ABBBAAABBBBBABABABAABABBBAABBAABBBBBA-BAAA

*000130_00318949 BABABBAABAAAAB-BBABBBBBBAABA-ABBBBBABAABBBAABA-ABBBAAABBBBBABABABAABABBBAABBAABBBBBA-BAAA

*000130_00376651 BABABBAABAAAAB-BBABBBBBBAABAAABBBBBABAABBBAABA-ABBBAAABBBBBABABABAABABBBAABBAABBBBBA-BAAA

*000130_00462292 BABABBAABAAAAB-BBABBBBBBAABAAABBBBBABAABBBAABA-ABBBAAABBBBBABABABAABABBBAABBAABBBBBAABAAA

*000131_00037329 B-BA-BABBABAABBBB--B-BAABBABBBBA-BBAABBAAA--ABA-BB-BBABBBBBABA-BBBAAABBAABAB-ABABBBBB-ABB

*000131_00103040 B-BA-B-BBAB-ABBBBAAB-BAA--ABBBBAA---ABBAAABAAB-ABBB-B-BBBBBABAAB-BAAA-BAAB---ABABBBBBAABB

*000132_00069761 AABBBAAAABBBABBABAA-BBABABABAAAAABA-A-BB-ABBB-ABAAAABAABABBBABBBABBAAABBBBAAABABBAAABBAAA

*000132_00225376 A-BBBAAAABBBABBABAA-BBAB-BABAAAAABA-AABBAABBB-ABAAAABAABABABABBBABBAAABBBBAAABABBAAABBAAA

*000132_00265694 A-BBBAAAABBBABBABAA-BBAB-BABAAAAABA-AABBAABBB-ABAAAABAABABABABBBABBAAABBBBAAABABBAAABBAAA

*000132_00410994 A-BBBAAAABBBABBABAA-BBAB-BABAABAABA-AABB-ABBB-ABAAAABAABABABABBBABBAAABBBBBAABABBAAABBAAA

*000132_00517663 ABBBBAAAABBBABBABAA-BBAB-BA-AABAABA-AABBAABBBAABAAAABAABABABABBBABBAAABBBBBAABA-BAAAABAAA

*000134_00117976 A-BBBAAAABBBABBABAA-BBAB-BABAABA-BABAABBAABBB-ABAAAABAABABABABBBABBAAABBBBBAABA-BAAAABAAA

*000134_00125375 AAB-BAAAABBB-BBA-AA-BBAB---B-A-AABA-AAB-AA-BBAABA-AAB-A-ABABAB-BABBAAABBBBBAA-ABBAAAAB-AA

*000134_00149736 A-BBBAAAABBBABBABAABBBAB-BABAABA-BA-AABBAABBB-ABAAAABAABABAB-BBB-BBAA-BBBBBAABABBAAAABAAA

*000134_00220159 A--BBAAAABBBABBABAA-BBAB-BABAABA-B-BAAB-AA-BB-ABAAAABAABABAB-BBBABB-A-BBBBBAABABBAA-ABAAA

*000134_00290847 A-B-BAAAABBBABBABA-B-BAB--AB-ABABBA-AABB-ABBBAABAAAABA-BABA--BB-ABBA-A--BBBAABABBA--A-AAA

*000135_00028244 BB-A-BBAABABABBBBAABAAABBB-ABAABBBB---BBBAABABAAABABBABBBABAAAABAAAAABBABABA-BBABAAA-AABB

*000135_00224150 BABA-BBAABABABBBBA-BAA-BBBAABAABBBBA--BBBA-BAB-AABABB-BBBABAAA-BAAAABBBABABA-BBABAA-AA-BB

*000135_00450970 B--A-BBAABABABBBBA-BAAABBB-ABAABBB---BBB-AABABA-ABABBABBBABAAA-BAAAABBBABABA-BBABA-BBAABB

*000135_00611696 BA-A-BBAABABABBBBAABAAABBB-ABAA-BB---ABBBAABABAAABABBABBBABAAA-BAAAABBBABABABBBABAABAAABB

*000136_00132900 BA-A-BBAAB--AAABBABABA-ABBAA-ABA---BBB-B--BBA-BBB-B-AA-BB--BBABAB-BB-A--BBBBAAAAABB--BAAA

*000136_00201769 BABABBBAAB-AAAABBA-ABAAABBAA-ABABABBBBAB-ABBAB--BBBAAA-BBA-BBA-ABABBBAB-BBBB----ABBBBBAAA

*000136_00211063 BABABB-AAB--AAABB--ABA-A--A--AB-BAB-B-ABBABBA-BABBBAAA-B-A-BBA-ABABBBABABBBB-A-BABBBB-AAA

*000136_00286016 B-BABBBAAB-AAAAB-A-ABAAABBAA-ABABABBBBABBA-BAB-BBBBAA-ABBA-BBA-A-ABBBAB-BBBB--A-ABBBB-AAA

*000136_00356644 BABABBBAAB-AAAABBAAABAAABBAA-ABABAB-BBABBA-BA-A-BBBAAA-BBA-BBABABABBBAB-BBBB--A-ABBBBBAAA

*000136_00421566 BABABBBAAB-AAAABBA-ABAAABBAA-ABAAAABBBAB-ABBABA-BB-AAA-BBA-BBA-ABABBB-B-BABB-AA-ABBB-BAAA

*000136_00594255 B-BABBBAAB-AAAABBAAABAAABBAA-ABAAA-BBBAB-ABBA---BB-AAA-BA--BBA-ABABBBAB-BABBA-ABABB-BBAAA

*000137_00148318 BAB-BBAAABBA-AABA-ABBB-BB-ABBABBBBBBBABAAABA--BBAAABAABBA-BABAAAABAB-BABABBBBAABAAABB-ABB

*000137_00180229 BAB-BBAAABBA-AABA-ABBB-BB-ABBABBBBBBBABAAABA-ABBAAABAABBA-BABAAAABAB-BAAABBBBAAAAAABB-ABB

*000137_00421111 B-B-BBAAABBA-AABA-ABBB-BBBABBA-BB--B-ABAAA-A-ABBBAABBABBB-BABAAAABAB---AA-BBBA-AAA-BB-ABB

*000137_00626115 B-BABBAAABBA--ABA--BB-ABBAABBABBBBB-BA-AAABA-A-BBAABB-BBB-BA-AAAABA--B-AABBBBAAAAAABBAABB

*000138_00125878 BAAAABBABBAAAAB-BAABAAAABB-BAAAA-B-AABABBABBBBABBAABBBABB-BA-AABAAAAAAAABA-BBAAAAAAAAAABB

*000139_00007581 B-AAABAABABB-BBAA-AA-AA-AAAAAAA-BBBBBBBABBBAAAABAABAABAAAABABBABB-A-AAABABAABAABBBBABABAA

*000139_00339003 B-A-A-AAB-B-BB-AA-AA-A-AA--AAAA-BBBBBBABBB-AAAAB---AABAA-ABABBABB-A--AAB-BAABA-BBBBABABAA

*000139_00566087 BBAAABAABABB-BBAABAABAAAAAAAAAA-BBBBBBABBBBAAAABAAB-ABAAAABABBABB-ABAAABABAABA-BBBBABAB-A

*000140_00070373 BABBBBAABBABABBBBAABA-B-A-ABBABB-AAAABBA-AABAABABABABABBBABBAAAB-BBAABBBAABABBABBBBAAAAAA

*000140_00283882 -AB-BBAABBABABBBBAAB--B-A-ABBABB-AAAABBABA-BAAB-BAB-BABB-ABB-AABB-B-ABBBAAB-BBAB-BB----AA

*000140_00311546 BABBBBAABBABABBBBAABABBBA-ABBABB-AAAABBA-A-BA-BABABABABBBABBAAABBBBAABBBAABABBABBBBAAAAAA

*000140_00444229 BABBBBAABBABABABBAABABBBA-ABBABB-BAAABBA-AA-AABABABABABBBAB-AAABBBBAABBBAABABBABBB-AAAAAA

*000140_00451335 BABBBBAABBABABABBAABAB-BA-ABBAB-A-A-ABBA-AA--AB-BABABABB--BBAA-BB-BAAB-BAABABBABBBBAAA-AA

*000141_00087427 B-BABB-BBAABABBABAABBAAAB-ABBAAAAAA-AABBBBAABBBAAAAABB-BBBBAABBAAAABBBAA-AABAAB-BBBBBAAAA

*000141_00203553 B---BB--BAAB-BBABAAB-AAAB-BBBA-AAAA-AABBBBAABBB-AAAA-B--BBBAAB-AAAAB-BA-AAA-AA--BBBB-AAA-

*000141_00522119 BBBABB-BBAABAABABAAB-AAAB--BBAAAAA--AABBBBAAB-B-ABAAB-AABBBAABBAAAABBBA--AABAA-ABBBA--AAA

*000141_00586346 B-BABB-BBAB-AABABAABBAAAB-BBBBAAAAAAAAB-BB-ABBBAA-AABBA-BBBAABBAAAABBBAA-AABAABABB-A-AAAA

*000142_00433281 BABBBBAABB-BABABBAABA-BBA-ABAABBAAAAABBA-AABAABABABAAABBB-BBAAABBBBAA-BBAABABBABBBBA-AA-A

*000143_00109664 A-BABAAABAAAABBBBA-AB-AAAABBBA-AAAAAAAABB-ABBBABBBBAAABBAAAB-ABBABBBABBABBABB-BAABBABABAA

*000143_00694976 ABBABAAABAAAABBBBA-AB-AAAABBBABAAAAAAAABBBABB-ABBBBAAABBAAAB-ABBABBBABBABBABB-BAABBABABAA

*000143_00712611 ABBABAAABAAAABBBBA-AB-AAAABBBABAAAAAAAABBBABBBABBBBAAABBAAAB-ABBABBBABB-BBABB-BAABBABABAA

*000144_00364419 BAABABAAAAABB-ABBBAA-AAABAABBABBBABBABBBAABAB-ABBBABAABAAA-AAABABBBABBABAABBAABBBAAABABBB

*000144_00465491 B-A-ABAAAAABBAABBBAA-AAABAABBABBBABBABBBAABABAABBBABAABAAAAAAABABBBABBABAABBAABBBAAABABBB

*000145_00156234 -BBA--BBAAAAA--AB--BBBABB--ABBBB--BBABBB---A-AABBA-BB-AB-ABAAABABABAAAB-AAB--ABB-AAAAB--B

*000146_00039399 BAABABBABBA-BABBBB-BAAAABA-BA-AA-BBAABBBBABB--ABBAABBBABBAABAAABAABBAAAABBAB-AAAAAAA-B-BB

*000146_00079732 BAABABBABBA-BABBBB-BAAAABA-BA-AA-B-AABBBBABB--ABBAABBBABBAABAAABAABBAAA-BBAB-AAAAAAAAB-BB

*000147_00246459 AAAAAABAABBABA-BABABAAABABBABBBBBBBBBBAAAABBA-B-ABBBABBBBAAABAAAB-ABABABBBBABBBBBAABABABB

*000147_00350754 AAAAAABAABBABA-BABABAAABABBABBBBB-B-BBAAAABBAAB-ABBBABBBBAAAAAAAB-AB-BABBBBABB-BBAABABABB

*000147_00362281 A-AAAABAABBABA-BABABAAABABBABBBBB-BBBBAAAABBAAB-ABBBABBBBAAAAAAAB-ABBBABBBBABBBBBAABABABB

*000147_00405173 AAAAAABAABAABA-BABABAAABABBABB-BB-BBBB-AAA-BAAB-ABBBABBBBAAAAAAAB-ABBBABBBAABBBBBAABABABB

*000147_00445569 A-AAAABAA-AA-A-BA-A-AAA-A--A-BA----BBBAAAA-BAABBABBBAB-B-AAA-AAAB-AB-BABBBAABB-BBAABABABB

*000147_00493122 AAAAAABAABAABA-BABABAAABA-BABBAB--BBBBAAAABBAAB-A-BBABBBBAAAAAAAB-ABBBAB-BAABBBBBAABABABB

*000147_00675829 BBBBB-AABAABBB-BBBA-AAABA-B-AAABAAAAAABA-B-AAABBABBBAAAB-B--BAAABBA-BABBBBA-BBBBABBA--ABB

*000148_00100142 A--A-A-BABBBABABAABAB-ABBBAAABBAAA-AA-ABAA-BABBB-AB-BAABAA-ABBBA-AAABBAABABA-ABAAAABBABAA

*000148_00126232 AABA-A-BAB-BA-ABAABA-AABBBAA-BBA-A--AAABA--BAB-BBABABA-B-AAB-BB-BAAABBAABA-AAABAAAAB-A-AA

*000148_00293003 A-A-AA-BAB-BABA-A--A-A-BBBAAABB-A-A-A-A--AABA-BB-ABABA-BA-ABABBAB-AAB--ABA-AAABAAAABBABAA

*000148_00361028 AAAAAA-BABBBABABAABABAABBBAAABBAAAAAAAABA-ABABBB-ABABAABAAAB-BBABAAABBAABA-AAABAAA-BBABAA

*000148_00571624 ---A-A-BAB-BABABA--ABAAB-BAAABB---A-ABAB---BAABB-ABA-AABAAAB-BBABAAA-B-ABA-A-BBAAAA-BABAA

*000148_00612312 AAAA----AB-BABABA-BABAABB-AAABB-AAAAAAABA--BAABBA-BABAABAAABABBABAAABBAABA-A-BBAAAABB--AA

*000150_00199509 BABABBBBBAABBBAABBBBAABBAABABAABBABAAAABABABB-AAA-ABBBBABAAAA-AAAAAABAAABAABBBBABBBAABA--

*000150_00237437 B-BABBBBBAABBBAABBBBAABBAABABAABBABAAAABABABBAAAA-AB-BBABAAAA-AAAAAABAAABAABBBBABBBAA-A--

*000150_00255792 BABABBBBBAABBBA-BBBBAABBAABABAABBABAAAABABABB-AAA-ABBBBABAAAABAAAAAABAAAB-ABBBBABBBAABA--

*000150_00373182 BABABBBB-AA-BBA--BBBAABBAABABAA-BAB--AAB-BA-BAAAA-A-B-B-BAAAA-AAA-AABAAABAABBB-A--BAA-AB-

*000150_00434739 B--A-BBBBAAB-BAAB-BB-ABBAABABAA-BABAAAABABABBAAAA-ABBBBABAAAA-AAAAAA-AAA-AABBBBA-BBAA-AA-

*000150_00570864 BABABBBBBAABBBAABBBBAABBAABABAABBABAAAABABABBAAAA-ABBBBAAAAAA-AAAAAABAAABAABBBAABBBAABA--

*000151_00209285 BAAAABAABBAAAABABAAAAAAAAABBBAAABB-AABABBABAAAAAABABBBABAABABAABBBBBB-BABAAAAAAAAAABBBBBB

*000152_00488578 BAABABA-BBABA-BABABABAABABABBAAAAAA--ABBBABBA-BBAAAAABABAABAA--AAABBBAABAA-BAAABBAAAAAAAA

*000154_00334906 BABABBBAAB-A--ABBAAA-A-AB-AA-AB-BBBBB-AB-B-BAB-AABBAAABBBAABBA-ABAABBAB-BBBB--A-ABBBBBAAA

*000154_00363800 B-BABBBA-BBAAAABBAAAA-AAB-AABABABBBBBBABBB-BAB-BA-BAAA-BBAABBA-ABABBBAB-BBBB--A--BBBBBA-A

*000154_00370146 BABABBBAAB-AAAABBAAAAAAAB-AA-ABABBBBBBAB-B-BAB--ABBAAA-BBAABBA-ABABBBAB-BBBB--A-ABBBBBAAA

*000154_00562039 BABABBBAAB--AAABBAAAAAAAB-AA-ABAB-BBBBABBB-BAB--ABB--A-BBAABBA-ABABBBAB-BBBB--A-ABBBBBAAA

*000154_00606386 BABABBBAABBAAAABBAAAAAAAB-AA-ABAB-BBBBABBBBBAB--ABBAAA-BBAABBA-ABABBBAB-BBBB--AAABBB-BAAA

*000154_00616253 BABABBBAAB-AAAABBAAAAAAAB-AA-ABABBBBBBABBB-BAB--ABBAAAABBAABBA-ABABBBAB-BBBB--A-ABBBBBAAA

*000154_00616339 B-BABBBAAB-AAAABBAAAAAAABBAA-ABAB-BBBBABBBBBAB--ABBAAA-BBAABBAAABABBBAB-BBBB--A-ABBBBB-AA

*000156_00008757 ABAAAABBAAAABAABBBAAAABAAABBABBBAA-A-BABAB-BAABAAAAABBAABABA-AABAAABAABABBBBA-ABBA-ABABAA

*000156_00165380 ABAAAABBAAAABAABBBAAAABAAABBABBBA-AA-BABABBBAABAAAAAB-AABABA-AABAAABAABABBBBA-ABBAAABABAA

*000156_00166124 ABAAAABBAAAABAABBBAAAABAAABBABBBAAAA-BAB-B-BAABAAAAABBAABABA-AABAAABAABABBBBA-AB-AAABABAA

*000156_00294534 B-AAABBBAAAAB-ABBBAAAABAAABBABBBAAAA-BABABBBAABAAAAABBAABABA-AABAAABAABABBBBA-AB-AA--ABAA

*000156_00423665 B-A-ABBBAAAABAABBBAAAABAAABBABBBA-AA-BA-ABBB-A-A--AABBAABABAAA-BA-AB-ABABBB-A-AB-AAA--BAA

*000156_00552922 B--AABBBAAAABAABBBAAAA-AAABBABBBABA--BABABBBAABAAAAABBAA-ABA-AABAAAB-BBABBBBA-ABBAA-B-BAA

*000157_00010534 AAABAA-BA-BBABAAA--AABAABAAA-ABAAABBBABAB-AABBBB-AAABBABAABAA-AABBABAB-BA-BAABBBBAABBBBAA

*000157_00103114 AAABAA-BA-BB-BAAA-BAABAABAAA-ABAAABBBABAB--ABBBB-AAABBABAABAABAABBABAB-BA-BAABBBBAABBBAAA

*000157_00415735 AAABAA-BA-BB-BAAA-B-ABAABAAA-ABAA-BBBABAB-AABBBB-AAABBABAABAABAABBABABABA-BAABBB-AABB-A-A

*000158_00192575 A-A-AAA-BAAABBA--BBB-BBBB-BBBAAA-A-BB-ABABBA-BAA-BBBBBABBBBAAB-BBBBBBBBABA-AAA-AABBAAAABB

*000158_00200352 AAAA-A--BAAABBA-BBBBBBBBBABBBAAA-ABBBBABAB-A-BAABBBBBBABBBBAABABBBBBBBBABA-AAABAABBAAA-BB

*000158_00589760 A-AAAA--BAAABBABBBBBBBBBBABBBAAA-AABBBABAB-A----BBBBB-ABBBBAAB-BBBBBBBBABA-AAABAABBAAA-BB

*000159_00387983 B-AAAB--ABBAAABBBA-AB-BAAA-B---A-A--B-BAA--AA-AA--BAA-ABAAAABAAA-ABAABBAB-BBBABA-B-B--BAA

*000159_00677087 B-AAABAAABBAAABBBABABABAAABBAAB-AAABBBBA-ABAAAAAABBAAAABAAAABAAAAABAA--ABABB-A-ABBBBAABA-

*000160_00550565 B-BABBBAB-ABBAAABBBBBAABABABBA-BABA-AA-ABABBBA-BABABBAAAABB---AAAB-B-BABBABBAABBBBB---ABB

*000161_00569110 B-BABBAABAAABBBBBBBBBBBB-ABAAABBBBBABABBBBAA---ABBAA-ABBBBBABABBBAABABABAABBAABBBBBA-BAAA

*000162_00197655 AAAAAAB--AABABBAAAA-BBBBBBA-BAAABB-BBABBBAABBBBAABBA-BBABABABABA-AABBBAAABABBBB-BBBBABAAA

*000163_00145507 BBAAABAABBBAAAB-BAABBABBABBAAAAABBBAAA-AABAAB-BBBABBAABABAABABBBBAABA-AAABBAABBAAAABBABBB

*000163_00583842 BBAAABAABBBAAAB-BAABBAB-ABBAAAA-ABAAAA-AAAAAB-BBBAABAABABAABABBBBAABA-AAABBAABBAAAABBABBB

*000164_00126512 B-BBBBA-AAAABAAB-BA-BAB-BABBBABBBBBBABAA-ABBBABABBAAAABABBBAAABAA-AAABAABBBAAAAABBBBBABBB

*000164_00240075 B-BBBBA-AAAABAABABA-BAB-BABBBABBBBBBABAA-ABBB-BABBAAAABABBBAAABAA-AAABAABBBAAAAABBBBBABBB

*000164_00562148 B-BBBBA-AAAABAABABA-BAB-BABBBABBBBBBABAA-A-BBAA-BBAAAABAABBAAABAA-AAA-AABAB-AA-A-AAABABBB

*000164_00577854 B-B-BBAAAAAABAABABA-BABABABBBAB-BB-B-BAA-ABBBA-BBBA-AABAABBAAABAA-AAABAABAB-AA-ABAAABABBB

*000164_00591259 BABBBBA-AAAABAABABA-BAB-BABBBABBBBBBABAABA-BBAABBBAAAABAABBAAABAA-AAABAABAB-AAAABAAABABBB

*000165_00007000 ABBABABBAABABABBBBBAABBBBAAABBBBAAABBBBAAAA-ABABAAABBBAABAAB-BABBABABBBAB-BABBBABAAABABBB

*000165_00117008 ABBABABBAABABABBBBBAABBBBAAABBBBAAABBBBAAAA-A-ABAAABBBAABAAA-BABBABABBBAB-BABBBABAABBABBB

*000165_00135557 ABBA-ABBAABABABBB--A--BBBAA-BB-BAAABBBBAAA--A-ABAAA-BBAABAAA-BABB-BABBBABABABBBA-AABB--BB

*000165_00193698 ABBABABBAABABABBBBBAABBBBAAA-BBBAAABBBBAAAA-A-ABAAABBBAABAAAABABBABABBBAB-BABBBABAAB-ABBB

*000165_00292450 ABBABABBAABABABBB-BAABBBBAAABB-BAAABBBBAAA-BA-ABAAABBBAABAAA-BABBABABBBAB-BABB-ABAAB--BBB

*000165_00319020 ABBABABBAABABABBBBBAABBBBAAABB-BAAABBBBAAA--A-ABAAABBBAABAAA-BABBABABBBAB-BABBBABAABBABBB

*000166_00050204 ABB--AABB-ABABAB-A-AB--B---BBBB-AA-BBAABBB-BB-ABBBBAAABBAAABBABBA-BBABBABBABB-BAABBBBABAA

*000166_00479364 ABBBBAABBAABABABBA-AB-ABAABBBBBAAAA-BAAB-BBBB-ABBBBAAAAB-BAB-A-BA-BBA-BABBABB-AAABBBBABAA

*000166_00499134 ABBBBAABBAABABABBA-AB-ABAABBBBBAAAABBAABBBBBBAABBBBAAAABABAB-ABBABBBA-BABBABB-AAABBBB-B-A

*000167_00477084 -ABABBBBBABBAABBBAABBBBBABBABABAABAAABAABBAAA-A-BAAAA--BBAABBABABBAAABBBBAB-A---BB-----AA

*000167_00574207 BABABBBABABBAABBBAABBBBBABBABABAABAAABA-BBAAA-ABBAAA-ABBBAABBABABBAAABBBBAB-AAB-BBBAAB-AA

*000168_00452204 B-ABABBAABBBBBABBBBAAABABAAAABBB-BA-ABBB-BABBAAABBAA--ABAABBB-BAABBABBB-A-BABBBBAAABB-BAA

*000169_00004195 BAB--BAB-AABBBABA-BAA-BAABABABB-AAA-BBB-ABBBBAAB--BA-ABB-BA-AAABA-BAAAB-BBBABAAABAAB-BBAA

*000169_00022881 B-B-BBABBABBB-ABABBAABBAABABAB-BAAABBBBBAB-BBA-BBBBA-AB-B-ABAAABA-BA-A-B-BBABAA-BAAB-BBAA

*000169_00026430 BABBBBABBA-BBBABABBAABBAABABABBBAAABBBBBABBBBAABBBBA-ABBBBABAA-BA-BAAAB-BBBABAA-BAA--BBAA

*000169_00035401 BABBBBABBA-BBBABABBAABBAABABABBBAAABBBBBAB-BBAABBBBA-ABBBBABAAABA-BAAAB-BBBABAA-BAABBBBAA

*000169_00524156 B-BBBBABBA--BBABABBA-BABA-ABABABA-ABBBB-ABBBBA-BBB-ABAB--BABAAABA-BAAABABBAABAA-BAAB-BBAA

*000170_00463455 A-BABAABAAABBBAAABBBBBBBBABAAAB-ABABBBABBB-B-AAAAAAABBABBABAAABABABB-B-B-ABBAB-BBAAABBAAA

*000171_00180448 ABBA-AB-AA-A-BAB-B--AABAAB-AAB-AAAAA-AB--B--ABBAAABBBA--BAAAB-B-BAB--AAAA-BAAAAAAB-BBABBB

*000171_00252850 ABBABABBAAAABBAB-BBAAABAABBAAB-AAA-AAAB--BAAABBAAABBBA-BBAAABABABAB--AAAAABAAAAAABBAB-BBB

*000172_00245034 --B---AAAA--BA-BBBBABBBB-A-AB-B-BBBA--ABBABABB-BBB--AABBBABBBABABB--BAAABABB-BAABB-AB-BBB

*000172_00362965 A--ABAAAAAAAB-ABBBBABBB-AAAAAA-ABBB-A-ABBABABB-BBBABAA-BBABBBABABBAABAAABABB-BAABBBABABBB

*000173_00354356 B-AAABBB-AABBBAABBBA-ABBA-BABAABBABAAAABABABB-AAA-ABBB-AAAAAABAAA-AAAAAAAAABBBA-BBBAAB--B

*000174_00142056 BBBBBBABBAABBBABBB-AA-ABABABABA-AAABBABBBB-BBA-BBBBA-AA-BBAAAAABA-BAA-B-BBAAB---BBBB-BBAA

*000174_00342641 BBBBBBABBAABBBABBB-AABABABABA-AAAAABBA-BBB-BBA-BBBBA-AABBBAAAAABA-BAA-B-BBAAB-A-BBBBBBBAA

*000175_00048530 B-AAABBBBAAAAAABAABB-AABBABABBBAAB-BBB-BABBAB-ABAABAB-BAB-BBB-AB--BBB-ABAABBAB-B-A-BBAAAA

*000175_00066951 B-AAABB-BAAAAAABAAB--AABBABABBBAABABBB-BABBABBABAABABBBABBBBBAAB--BBBBABAABBABBBAAABBAAAA

*000175_00279982 BBBABB-BBAAAAAAB--AB--ABBABABBBAABABBB--AB-ABBA-AABAB-BA-B-ABAAB--BB-BBBAABBABBBAAAB-AAAA

*000175_00566389 B-BABBBBB-AAABABAAAB-AA-B-BABBBAABABBB-BAB-ABBAAAABA-BBABBBAAABB--BBBBABAABBAB-BAAABBAAAA

*000177_00169443 ABBBBABAAABAAABAAAAAABABAAAAB-ABBBBBBBBAAA-ABBABBB-AB-ABBAAAAAAB-BBBABABBAABBBABBBBBBAA--

*000177_00171488 A-BBBABAAA-AAABAA-AAA-ABAAAABBABBBBBBBBA-A-AB-ABBB----ABB--AAAAB-BBBA--BBAAB-B-BBBB---A--

*000177_00466318 ABBBBABAAA-AAABAAAAAABABAAAAB-ABBBBAABBAAABABBABBB-AB-ABBA-AAAAB-BBBABABBAABBBABBBBBBAA--

*000177_00467027 ABBBB-BA-ABA-ABA-A--ABAB-AA-BB-BBBBA-BB--AB-BBABBBBAB-ABB-BA--ABBBBBABABBA-BBBABBBBBB-A-B

*000177_00470271 ABBBBABAAABAAABAAA-AABABAAAAB-ABBBBAABBAAABABBABBB-AB-ABBA-AAAAB-BBBABABBAABBBABBBBBBAA-A

*000177_00553664 ABBBBABAAABAAABAAAAAABABAAAAB-ABBBBAABBAAABABBABBB-AB-AABA-AAAAB-BBBABABBAABBBABBBBBBAA--

*000178_00008625 BBBBBBAABAAABBABBBABABB-B-BBBAAAAAABBBABABBA-BAAABBBBBAB-BBAABABBBABBAAAAA-AAAAAABBBBA-BB

*000178_00067585 BBBBBBBA-AAAB--BBB-BABB-BB--BAA-AA-BBBABABB-B-AAABB-B-ABB-BAABAB-BA-BAA-AA-AAAAAA--B---B-

*000178_00105085 B-BBBBB-BAAABBAABBABAB--BABBBAAAAAABBBA-AB-AA--AABBBB-ABABBAABAB-B-B--AA-AAAAABA--BBBAABB

*000178_00447603 BBB--B-A-AAA-BAABB-BAB--BABBBAAAAAABB-A-AB-ABBAA-BBBBBAB-BBA-B--BBABBA-AAA-AAA-AABBBB-BBB

*000179_00142144 B-BABBABABAAABBAAABABBABAABBAABAAAABBABB-B-AA-AABABBBAA-AAAABBBBAAABABBABABABABABBBABABBB

*000179_00143488 --BABBABABAAABBAA-BABBABAABBAABAAAABBABBAB-AAA--BABBBAA-AAAABBBBA-AB-BBABABA-ABAB-BAB-BBB

*000179_00170605 BABABBABABAAABBAAABABBABAABBAABAAAABBABBAB-AAAAABABBBAA-AAAABBBBAAABABBABABABABABBBABABBB

*000179_00205035 BABABBABABBAABBAAAB-BBABAAB-A-BAAAABBABBAB-AA-A-BABBBAA-AAAABBBBAAABABBABABABABABBBABA-BB

*000180_00599847 AABABAABAAABBAAAABBBBBBBBABAAABBABABBBABBBAB-AAAAAAABAABBABAAABABABB-B-B-ABBBB-BBAAAABAAA

*000181_00368932 ABBABA-BAB--BBABABBABAA-ABBAABBABABAABAB---BAAABBABAABABAABBBBBA--AAABBABA-ABBBAAAABB-BAA

*000181_00480688 A-AAAA-BAB-ABBABABB-BAAB-BBAABBABABAABAB--ABAAABBAB-BBABAABBBBBA--AAABBABA-ABBBAAAA-B-BAA

*000183_00090689 BABBBBBBBAAB-BAAB--BA-BB--BABAABBAB-BA-A---AB-AAA-AABBAABAAAAAAAAABABAA-B-ABBBB-ABBABBAAA

*000183_00095410 B-B-B-BBBAA-B-AAB-BBA-BBA-BABAA-BA-BBABAAB--BA--ABAABBAABAA-A-AAAABABAABBAABBB-B-BBA--AAA

*000183_00144988 B-BBBBBBBAAB-BAAA--BA-BB--BABAA-BABBBABAAB--BAAAAAA-BBAABAAAA-AAAABABAABB-ABBBB--BB-BBAAA

*000183_00571266 B-BABBB-ABABBBABABBABAA-ABBAABBABA-AAB-B-BABBAABBAAAABABAABBBBBA-BAABB-ABA-AB-BAAAABB-BAA

*000184_00649381 B-BBBBAAAAAB-BAAB-BBABBB-BBAAAABBA--BABA-B-AAA--ABAA-B-ABAAAABAAAABABAABBAABBBBBABBAABAAA

*000185_00565920 A-BABAABAABAAAAABAABBAA-BABABBBBBB-BAABBAB-AAAABBA-BBAAAAABAAABB-BBAAAB-AABAABBBBAAAAB-BB

*000185_00566539 A-BABAABAABAAAAABAABBAA-BABABB--BBBBAAB-ABBAAAABBA-BBAAAAABAAABB-BBAAAB-AABAABBBBAAAABBBB

*000186_00189555 B-BABBAAABABBABBBBABAAABAA-ABABBBBBAABBAAA-BABAAABA-ABABBAAAAAABABAAAABAABBAABAABAABB-BAA

*000186_00378354 B---BBAA-BABBA-BBB-B-AABAA-ABAB-B-BAABBAAAA-A-AAABABABABBAAAAAAAABAAAABAABBAABAABA-BBABAA

*000187_00358775 BBBBBBABABABBAABBBBBBAAABBAABBAAABABBBABABBAABAA-A-BA-BBABAAABBBBBBABABBBBBABBABA-ABAABBB

*000188_00319712 AA-B-ABBABB-AAAABAAAAAABBBBBABBAAAAAA-A-AAABABAB-AAABAABBAAA-BABB-ABBBAABABAAABAABBBBAAAA

*000188_00426222 A--B-ABBABB-AAAABAAAAAABBBBBABBAAAAAA-A-AAABABAB-AAABAABBAAA-BABB-ABBBAABABAAABAABBBBAAAA

*000189_00194217 B-ABABABABBBBABBBBBABBAAAAB-AB-ABBBBBABBBBABABBBABAB-ABBAAABBABBA-BBAAAABAAAA-BAAAAABBBBB

*000189_00489526 BBABAB-B-BBBBABBBBB-BBAAA-B--B-ABBBBBABBBB-BABBBA-ABBABBAAABBABBABBBAA--B-AAABBABAAABBBBB

*000189_00695358 B-ABABABABBB-ABBB-BABB-AAAB-AB-ABBBBBAB-BB-B--BBA-AB-ABBAAABBAB-A-BBAA--BA-AA-BABAAAB-BBB

*000190_00013528 ABA-AABBBABBBBAB-B-BA-AABBBBBBBBAAAA-BBB-ABAA-BAAAAA--ABA-AB-AAAAAAB-A-ABABBBBAABB-AA-BAA

*000192_00372498 ABBABABBAAAAAAAABAABBBA-BA-ABBBBBBBBABBBBBBAAAABBA-BBAABA-BAAABABABAAAB-AABAAABBBAAAAB-BB

*000192_00518897 BB-A-BBBAABABBBBABBABBBABABABBBAAAABBAAABBABBABABAABBABBABBBAABABABABBBBBABBABB-ABBAABABB

*000193_00157554 BBBABBBBAA-A-BBAA-ABBBBBBBBABBAABBBAAABBBBBABABBBAABAABABAABAAB-AAB-BA--AABBABBBAAABBABBB

*000193_00412031 BBBABBBBAA-A-BBAA-ABBBBBBBBABBAABBBAAABBBBBABABBBAAAAABA-AABAAB-A-B-BAA-AABBABBBAAABBABAA

*000193_00432184 BB-ABBBBAA-A-BBAAAABBBBBBBBABBAABBBAAABBBBBABABBBAAAAABABAABAAB-A-B-BAA-AABBABBBAAABBABAA

*000193_00490990 BBB-BBBBAA-A-BBA--AB-BBB-BBA-BA--BBAAABBBBB-BABBBAAAAAB-BAABAAB-A-B-BAA--ABBAB-BAAABBABAA

*000194_00190297 BAABABAABBABA-BB-ABAABAAABABBBAAAAABBABBBBBAAABBABBAAAABAAB-AAAAABBBBAABAA-BAA-BBAAAA-BAA

*000194_00434608 B-ABABAABBABA-BBBABA-BAAABABBBAAAAABBABBBB-AAABBABBAAAABAABAAAAAABBBBAABAA-BAAABBAAAAABAA

*000194_00657995 B-ABABAABBABA-BBBABAABAAABAABBAAAA--BABB-BBAAABBABBA-AA--ABAAAAAAABBB--BAA-BA-ABBAA-AA--A

*000196_00431709 B--A-BBA--AB-BBBBAABBAAABB-ABAA-BBBBAAB-BA-B-BAAABA-BA-BBABAA--B-ABB-BBA-BB---BABAAB-AAB-

*000197_00758378 B--A-BBBAABABBBBABBABBBABABABBBAAA-BBAAABBABB-BABAABBABBABBBAABABABABABBBABAAAB-ABBAABABB

*000198_00399354 BAABABABBBA-BABBBB-BAAAABAABA-AA-BBAABBBBABB-AABBAABBBABBAABAAABAABBABAABBAB-ABAAAAAAB--B

*000198_00400512 BAABABABBBA-BABBBB-BAAAABA-BA-AA-B-AABBB-A-BAAA-BAABBBABBAABAAABAABBABAABBAB-ABAAAAAAB-BB

*000198_00449581 BAABA-ABBBA-BABBBB-BAA-A-A-BABA--B-AABBBBA-B--AB-AABB-ABBAABAAABAABBABAABBA-BABAAAAAABABB

*000198_00462759 B-ABABABBBA-BABBBB-BAAAABA-BA-AA-BAAABBBBABB-BABBAABBBABBAAB--ABAABBAB-ABBAB-ABA-AAAAB-BB

*000199_00180016 BBAAABBAB-ABBAAABBBBBAABA-ABBAABBBAAAA-ABABBAAB-AAAAB-AABBB-ABA-AB-B-BA-AAB-AABABAA-AAAAA

*000199_00362425 BBAAABBAB-ABBAAABBBBBAABA-ABBAABBBAAAA-AB-BBAABBAAAABAAABBB-ABAAAB-BBBA-AABBAABABAA-AAAAA

*000200_00456281 BBABABABAABBAB-ABA--BABABBABBBBABB-AAAABABBAB-ABBBBA-AAAABABAAB-A---AA-BAABBBAA-BAAAAAAAA

*000200_00740775 BBAB-BABAABBABBABA-BBABABBABBBBABBB-AAABAB-A--ABB-BAB-AAABAB-ABBA--BAA-BAABBBA--BAAAA-AAA

*000201_00183592 BABABBBBBBABABBBBAABBBBBAABABABAABAAABABBBAAA-ABBBBAAABBBAA-BABABBAAAAABBABBAAA-BBBAABBAA

*000202_00467228 A-BBBAAAABBAABBABAA-BBABABABABAAAB-AAAB-AABBA-BBAAAABAABAB-BABBBABBAAABBBBAAABABBAAABBAAA

*000203_00040671 AAAAAA--BAAABBA-BBBBBBBBB-BBBABA-AABBBABABBA-BAABBBBBBABBBBAABABBBBBBBBABA-AAABAABBAAA-BB

*000203_00598796 A-AAAA--BAAABBA-BBBBBBBBB-BBBABA-A-BBBAB--BA-BA-BBBBBBABBBBAABABBBBB-BB-BA-AAABAABBAAABBB

*000203_00670488 AAAAAAA-BAAABBA-BBB-BBBBB-BBBAB--A-BBBABAB-A-B-ABBBBBBA-BBBAABABBBBBBBBABA-AAA-AABBAAA-BB

*000204_00325048 B-A-A-AAABB-AAAB-A-ABA-AA-BAAAB-AA--BBBAA--AAA-AABBAAA-BAAAABA-AAAAAABBABABBBAB-BBBBAABAA

*000205_00182160 BBAAAB-BAAAABAABBBAAAABAAABBBBB-A-A---ABAB-BAABAAAAAB-AABABAAAABAAABAABABB-BA-ABBBBAB-BAA

*000205_00384980 BBAAABBBAAAABAABB-AA--BA-ABBABB--AAA-BABAB--AABAAAAA-BAABABA--ABAAABAABABBBBA-A-BBBABABAA

*000206_00227854 B-BABBAAAABBABBAAAAAB-AABA-BBA-AB----AA-BABBA-BBA-AB--AAABBB-BBBAABABBABAAA-BABABB-AA-ABB

*000206_00357757 B-BBBBAAAABBABBAAAAABBAABABBBAAA-BAAAAA-BABBABBBAAABABBAABBBBBBBAABABBABAAA---BAB-ABBBABB

*000206_00469004 BBBBBBAAAABBABBAAAAABBAABABBBAAA-B-AAAA-BABBABBBAAABABBAA-BBBBBBAABABBABAAA---BAB--BBBABB

*000208_00125479 AAAAAA-BAB--BBABABBBBAAB-BBAABBABABAABAB--ABAAABBABABBABAABBBBBAB-AAABBABA-ABBBAAAABB-BAA

*000209_00285717 B-BBBBAABBBBABBBBA-BABBAAAAABABBB-BAABABAAAAABB-BABABABABABBBAABABBAAAABAABABBABBBBAAAAAA

*000209_00426190 B-BBB-AABBBBABBBBA--ABBAA-AABABBB-BAABABAAAAABB-BABABA-ABABBBAABABBAAA-BAABABBABBBBAAAAAA

*000210_00128733 AA-B-ABBABABBAB-BB--AABBAABABBBABBBBBABAABBABBABAA-AB-BBA-ABBAAAAB-AA-BABAAABAA-AAABBBBAA

*000210_00357200 AA-BBABBA-ABBAB-BBB-AA-B-ABABBB-BBB-BABAAB--BBABAA-ABBBBAAABBAA--BBAAA-ABABABAAA-AABBB-AA

*000210_00380425 AA-B-ABBABABBAB-BB--AABBAABABBBABBBBBABAABBABBABAA-AB-BBA-ABBAAAAB-AA-BABABABAA-AA-BBBBAA

*000210_00450105 AA-B-ABBABABBAB-BB--AABBAABABB-ABBBBBABAABABB-ABAA-AB-BBA-ABBAAAAB-AA-BABABABAA-AAABBBBAA

*000210_00484208 A-AB-ABBABA-BABBBB-AAABBAAB-BB-ABBB-BABAABB-BB--AA-AB-BBA-ABBAAAABAA-A-ABABABAA-AA--BB-AA

*000212_00035391 BAABAB-BBABBAABABABABAAAAABBBBAAABA-AAABABAABABBABAAABABB-ABABBBBBAA-AABAAABAABBBAAABAAAA

*000214_00132005 AB-BAABAABBBABABAABABBBAAABAABBBB-BAABABABABBA-BBBAA-BABAABBBBBAAABABBBAA-BAAABA-AAABBBAA

*000214_00257998 ABABA-BAABBBABABAABA-BBAAABBAB-BBAB-ABBBBB-BB--B-BAA-BAB-A-BBBBAAABA--BAABBAAA-A-AA--A-AA

*000214_00342996 A-ABAABAABBBABABAABABBBAAABBABBBBABAABBB-BABBAABBBAA-BABAABBBBBAAABABBBAA-BAAABA-AABB-BAA

*000214_00366161 ABABAABAABBBABABAABABBBAAABBABBBBABAABBB-BABB-ABBBAA-B-BAABBBBBAAABABBBAA-BAAABABAABB-BAA

*000215_00088643 BABABBBAAB-AAAAB-AABAAAAB-AABAAA-BBB-AABAB--ABAAABA-AA-B-ABB-A-AAABB-BBBBBBA--BBA-ABBBABB

*000215_00263833 BABABBBAAB-AAAAB-AAB-AAAB-AABA-A-BBB-AABAB-BABAAABABAA-BBABB-A-AAABBBBBBBBBA--BBAAABBAABB

*000215_00281382 BABABBBAAB-AAAAB-AAB-AAAB-AABAAA-BBB-AABABABABAAABABAA-BBABB-A-AAABBBBBBBBBA--BBAAABBAABB

*000215_00289449 BABAB-BAAB-AAAAB-AABBAAA-BAABA-A-B-B-AABABABABAAABABAA-BBA-BAA-AAABBBB-BBBBA--BBAAAB-AABB

*000215_00407206 BABABBBAAB-AAAAB-AAB-AAAB-AABAAABBBB-AABABABABAAABABAA-BBABB-A-AAABBBBBBBBBA--BBBAABBAABB

*000216_00150177 BABABBBAAABAABA-BAABAAAAAABBBBAAABA-ABAB-ABAABABBA-BBABBBABABAABB-AAAABABB-BBAAAAB-AAAABB

*000216_00252234 BABAB-BAAABAABA-BAAB-AAAAABBBBA-ABAAABABBABAABABBA-BBABBBABABAABB-AAAABABABBBAAAABBAA-ABB

*000217_00055522 BABBBBAABBABAB-BBABBBBBBAABABABAABABBBABBB-ABABABBBAAABBBBA-BABABAABAA-BAABBAAABBBB-ABAAA

*000217_00599231 BABBBBABBBABAB-BBAABBBBBAABABA-AABAAABABBBAAB--ABBBAAABBBAA-BABABAAAAA-BBAB-AAA-BBBAABAAA

*000218_00070204 BBBBBBABBAABBBABBB-AABABABABABAAAAABBAABBBBBB--BBBBA-AABBBAAAAABA-BBA-B-BBAAB-A-ABBBBBBAA

*000218_00077145 BBBBBBABBAABBBABBB-AABABABABABAAAAABBAABBBBBB--BBBBA-AABBBAAAAABA-BBA-B-BBAAB-A-ABBBBBBAA

*000218_00151703 BB-BBBABBAABBBABBB-AABABABABABAAAAABBAABBB-BBB-BBBBA-AABBBAAAAABA-BBA-B-BBAAB-A-ABBBBBBAA

*000218_00230776 BBBBBBABBAABBBABBB-AABABABABABAAAAABBAABBB-BB-BBBBBA-AABBBAAAAABA-BBA-B-BBAAB-A-ABBBBBBAA

*000218_00271711 BBBBBBABBAABBBABBB-AABABABABABAAAAABBAABBBBBB--BBBBA-AABBBAAAAABA-BAA-B-BBAAB-A-ABBBBBBAA

*000218_00346093 BB-B-BABBAA--BABBBBAABABABABABAAAAABB-BBBB-BB--BBBBA-AABBBAAAAABA-B-A-B-BBAAB-A-BB-B-B-AA

*000218_00501316 BBBBBBABBAABBBABBB-AABABABABABAAAAABBABBBBBBB--BBBBA-AABBBAAAAABA-BAA-B-BBAAB-A-BBBBBBBAA

*000219_00086243 BABBBBBABBBBABAABABBABAABAAAAAAABA-BBABAB-ABB-BB-AAAABBBBAAABB-ABAABABBAA-AABBBA-AABBBBAA

*000219_00264353 BABBBBBABBBB-BAABABBABAABAAAAAAABA-BBABAB-ABBBBB-AAAABBBBAAABBAABAABAB-BA-AABBBB-AA-ABBAA

*000219_00287652 -ABBBBBABBBBABAABABBABAABAAAAAAABAAB-ABAB-ABBBBB-A--ABBBBAAABBAAB-ABAB-BA-AABBBB-A-AA-BA-

*000220_00340919 BBBABBABAABABBBBABBABBBABBBABBBAAAABBABABBABBABABAABAABBABBBAABABABB-BBBBABBABBBABBAABABB

*000220_00379735 BBBABBABAABABBBBABBABBBABBBABBBAAAABBABABBABBABABAABAABB-BBBAABABABBBBBBBABBABBBABBAABABB

*000222_00019242 AABABAABAA-BABBBAAABBBBBABAB-AAA-B-BBABBBAABBBABABBA-BBABABAB-BAAAABB-BAABABBAB-BBBBABAAA

*000222_00019608 AABABAABAA-BABBBAAABBBBBABAB-A-A-BBBBABBBAABBBABABBA-BBABABAB-BAAAABB-BAABABBAB-BBBBABAAA

*000222_00163613 AABABAABAA-BABBBAAABBBBBABAB-AAAAB-BBABBBA-BBBABABBABBBABABAB-BAAAABB-BAABABBAB-BBBBABAAA

*000222_00440975 --BABAABAA-BABBBAAABBBBBABABBAAAAB--BAB-BA-BBB-BABBA-BBABABAB-BA-AABB-BAABABBABBBBBBABAAA

*000222_00486132 AABABAABAA-BABBBAAA-BBBBABABBAAAAB-BBABBBAABBBBBABBA-BBABABAB-BA-AABB-BAABABBAB-BBBBABAAA

*000223_00105504 A-BABAABAA--BBAAABBB-BBBAABAAAB-AB-BBBABBBA---AAAAAAB-A-BABAAA--B-BBA--B-AB-AB-BBAAA--A-A

*000223_00207323 AABABAABAA-BBBAAAB-BBBBBAABAAABBABABBBABBB-AA-A-AAAABB-BBABAAABAB-BB-B-B-ABBABBBBAAABBAAA

*000223_00308309 AABABAABAAA-BBAAABBBBBBBAABAAABBABABBBABBBAAAAAAAAAABBABBABAAABABABB-B-B-ABBABBBBAAAB-AAA

*000225_00066185 A-AA-AA-BBBAAABBBAABAAB-AABAAA-BA-A-AA-AAAABAB-AB-ABAAB--AA-ABABBAABA-AA-BBABBB-AAA--AABB

*000226_00465376 B-A-ABBBABB-BABA-BBBAABAAAABBBBBBB-AABBAAB-BB--BBABBA-ABABBBB-ABABAAABBABABBBAAABBBB-BABB

*000227_00085579 BBBABBA-ABBA-ABA-BAA---B-AABAAB-A-AAABBBAB--AAABA--ABBB-AAAABA-AAB-ABAABB-BAAB-BBB-B--AAA

*000227_00319335 B-BABBAAABB--ABA-AA-AAB--AAAAA--AA--ABB-AB-AAAB-AA-ABBBAAAAAB-AAABAABAABB-B-ABA-BBB----AA

*000227_00431073 B--ABBA-ABBA-ABAB-AAAABB-AAAAABAAAAAABBBABBAAA-BAABABBBAAAAABAAAABAABAABB-BAABABBBBBB-AAA

*000228_00025339 ABBBBAABBAABBBABBB-A-BABAABBBBBAAAAB-AABBB-BB-ABBBBAA-ABABABAABBA-BBA-BBBBAABAABABBBBABA-

*000228_00040075 ABB-BAABBAABB-ABBBAAB-ABAABBBBBAAA-BBAA-BBBBB-ABBBBAA-ABAB-BAAB---BBABBBBBAAB-AB-BBB-ABAA

*000228_00375302 ABBBBAABBAABBBABBBAAB-ABAABBBBBAAAABBAABBBBBB--BBBBA-AABABAAAABBA-BBA-BBBBAAB-ABABBBBABAA

*000228_00391051 ABBBBAABBAABBBABBBAAB-ABAABBBBBAAAABBAABBBBBB--BBBBA-AA-ABAAAABBA-BBA-BBBBAAB-ABABBBBABAA

*000228_00545746 ABBBBAABBAABBBABBBAAB-ABAABBBBBAAAABBAABBBBBB--BBBBA-AABABA-AABBA-BBA-BBBBAAB-ABABBBBABAA

*000229_00209135 B---B-AAAABABBAB-BBAB-B-A-AAAAB--BBAA-ABBAB-BB--BBAB-A--BABB--BA-BAAAAAAB-B-BB-ABBBA--BBB

*000230_00007039 AAA--A-BBAAAB-A---BBBBBBB-B-B-AABA--BBABABBABBAABBBBBBAB-BB-A-AB-BBBBBBA-A-AAABA-BBA-A-BB

*000231_00026731 A-AB-AAB-BBAAA--AAAAB--AA----AA-BABAAAABAA-BA--BBA-AA-AA-ABABBBBABBB-ABBAAABBAA--AA--AAAA

*000231_00079928 ABABAAABABBAAABAAAAABA-AAABBBAA-BABAAAABAABBABABBA-AAAAABABAB-BBABBBAABBAAABBAABAAABBAAAA

*000231_00119293 ABABAAABABBAAABAAAAABA-AAABBBAABBABAAAABAABBABABBA-AAAAABABABBBBABBBAABBAAABBAABAAABB-AAA

*000231_00224777 ABABAAABABBAAABAAAAAB--AAABBBA-BBABAAAABAA-BABABBA-AAAAABABABBBBABBBAABBAAABBA-BAAABBAAAA

*000231_00317979 A-ABA-ABABBAAABAAA--BA--AABBBAAB-A-AA-A-AABBAB-BBA-A--AABABABBBBA-BBA-BBAAABBAA-AAAB--AAA

*000231_00367231 A-A-AA-BABBAAABAAAAABAAAAABBBAABBA-AAAA-AABBABABBA-AAA--BABABBB--BBBAABBAAABBA-B-AABBAAAA

*000231_00415977 A-ABAAABABBAAABAAAAABA-AAABBBAABBABAAAABAABBABABBA-AAA-ABABABBBBABBBAABBAAABBAABAAABBAAAA

*000231_00429141 ABA-AAABABBAAABAAAAABA-AAABBBAABBABAAAABAABBABABBA-AAA-ABABA-B-BABBBAABBAAABBAABAAABBAAAA

*000231_00455765 ABABAAABABBAAABAAAAABA-BAABBBAABBABAAAABAABBABABBA-AAA-ABABABBBBABBBAABBAAABBAABAAABBAAAA

*000232_00019621 B-BBB-AAAABBABBAAAAABBABBABBBA-BABAAABA--AABA-BBABABABBAAABBBBBBAABABBABAA-AABBAB--BBBABB

*000232_00216182 BBBBBBAAAABBABBAAAAABBABBABBBA-BABAAABABBAABA-BBABABABBAABBBBBBBAABABBABAA-AA-BAB--BBBABB

*000232_00396732 BBBBBBAAAABBABBAAAAABBABBABBBAAAABAAAA-BBAABABBBA-ABABBAABBBBBBBAABABBABAA--A--AB--BBBABB

*000233_00242517 BBBABBAAABA-A-BB-A--A-ABBA-ABA---BBBBB-BBA-B-BA-ABA-A-ABBAAAAAAAA-AAAABAABBABBA---AAAA-AA

*000233_00244982 BBBABBAAABABAABBBAABAAABBA-A-ABBABBBBBBBBAABABA-ABA-ABABBAAAAAAAAAAAAABAABBABB-ABAAAAAAAA

*000233_00421644 BBBABBAAABABAABBBAABAAA-BA-ABABBAB-BBBBBBAABABA-ABA-ABABBAAAA-AAAAAA-ABAABBAB-AABAAAAAAAA

*000233_00428737 B-BABBAAABA-AABBBAAB-AABBA-ABABBAB-BBBB-BAAB-BA-ABA-ABABBAAAAAAAAAAAAABAABBA-BAABAAAAAAAA

*000233_00687359 BBBABBAAABABAABBBAABAAABBA-ABABBABBBBBBBBAABABABABAAABABBA-AAAAAAAAAAABAABB-BBAABAAAAAAAA

*000234_00038885 A-AAAAABABABB-AAABB-BAABABBBABABAABBBABBAB--BB-A--AABAABABBAABBABBABBA-AABAAAAAA-AAABAAAA

*000234_00224993 AA-A-AABABABB-AAABB-BAABABBBABABAABBBABBABB-BB-A--AABAABABBAABBABBABBA-AABAAAAAA-AAABAAAA

*000235_00432718 AABBBAABAAABBBAAAB-BBBBBAABAAABBABABBBABBBAAAAAAAAAA-B-BBABAAABABABBABAB-ABBABBBBAAAB-AAA

*000236_00263877 BBBAB-AA-BABBA-BB-ABAAABAB-ABA--BBBABBBAAAAB-AAAABABA-ABBAAAAAAAABAA-BBAAB-AABBA-AABBAAAA

*000236_00444030 BBBABBAAABABBABBBBABAAABA-BABABBBBBABBBAAAABA-A-ABABABABBAAAAAAAABAAABBAABBAABBABAABBAAAA

*000238_00153216 AAABAAABABBAAAABBAAABB--ABABBABAA--AA-AABAAABBABBAABAAABABAAAABBBBBBBAAABAABBBAABBBAAABBB

*000238_00159009 AAABAAABA-BA-A-B-AAA-BA-AB-BBABAA-A-ABAA-AAAB-ABB-ABA-ABABA-AA-BBBBBBAAAB-ABBBAA-BBAAABBB

*000238_00194558 AAABAAABABBAAAABBAAABB--ABABBABAA-AAA-AABAAAB-ABBAABAAABABAAAABBBBBBBAAABAABBBAABBBAAABBB

*000238_00194644 AAABAAABABBAAAABBAA-BB--ABABBA-AA-AAA-AABAAABBABBAABAAABABAAAABBBBBBBAAABAABBBAABBBAAABBB

*000239_00025826 ABAAAAAAABBABAABBBAABBA-ABA-BABAABAAA-AA-AAABBABBAABAAABABAAAAB-BBBABAAA-AAB-BAA-B-AAABBB

*000239_00191178 ABAAAAAAAABABAABBBAA-B-AABA-BABAAB-AA-AABA-ABB-BBAABAAABABAAAABABBBABAAABAAB-B-ABBBAAABBB

*000240_00135198 ABA-AABABBBABB-BA-AAAAAAAB-AAABAAAABAB-BBABABBBAABAAABBAB-BABBAABABAABBAAABBBAB-BBB-A--AA

*000240_00533366 A-AAAABBBBBAAB-BA-AAAAABABAAAABBAAA-AB-BBABABBBAABAAA--AB-BABBAABABAABBAAABBBABABBBAABBAA

*000242_00038796 A-AAAABBAABA-BAA-BA-AA-ABB-AAA-A-A-AAAAAABAB-B-B-AB-B-AABAAAABBBBABABABABABAAAAAABB-ABBAA

*000244_00289457 BBAAABBB-BABBAAABBBBBA-BA-ABBAAABB---ABABAB-A--B-A-A-----BB-ABAAABBBBBAAAABBAABABAAAAA-AA

*000244_00591564 BBAAABBBBBABB-AABBBBBAABA-ABBAAABBA--A-ABABBAABBAAAABAAA-BB-ABAAAB-BBBA-AABBAABA-AA-AAAAA

*000245_00368453 B-AAABBBABABBBBBBBBA-AAABB-BA-BABBBB-AABBBBAB-BABA-AABABBBBBBA-AAAABAAAAB-BBBBBAABBAAABAA

*000246_00062827 BABBBBB-BABA-AB-A-ABABBABBAAABABBBBAABBBABABAABBAAAABABAAAAAAAABAAA-BAABBBAAAAABBBBBBBBAA

*000246_00296621 BA-BBBB-BABA-AB-B-AAABBABBAAABABBBBAABBAABABAABBAAAAB-B-AAAAAAABAAA-B-A-BBAAAAAB-B-B-BBAA

*000246_00439695 --BB-BB-BAB-AA----AAA--AB-AAAB--BABAABBAAB-BAABB-AAABABAAAAAAAABAAA-BAABB-AA-A-BB-BBBBBAA

*000247_00185553 BBABABBABABBABAABABBABAABAA-AAAABA-BBABAB-AB-BBB-AAAA-BBBABABBAABAABABBAA-AABBBA-A-BBBBAA

*000247_00358595 BBABABBABABBABAABABBABBABAAAAAAABAABBABABAABB-BBBAAAABBBBABAB-AABAABA-BAA-AABBBAAAA-BBBAA

*000248_00011679 AABBBABABBBBABABBAAABABAAABAABABBBB-BBBB-BBBAABBBABABABBABBBBABABBBBBAAABAABABABBBBBAAAAA

*000248_00200058 A-BBBABABBBBABABBAAABABAAABAABABBBB-BBBBBBBBAABBBBBABABBABBBBABABBBBBAAABAABABABBBBBA-AAA

*000248_00242989 AABBBABABBBBABABBAAABABAAABAAB-BBBBABBBBBBBBAABBBBBABABBABBBBABABBBBBAAABAABABABBBBBAAAAA

*000250_00387896 BAABABAABBABA-BBBAAAAAAAABABBAAAAAABBABBBB-AAABBABBAAAABAABAAAAAABBBBAABBA--AA-BBAAA-ABAA

*000251_00135570 ABBABABBAABABABBBBBAABBBBAAABBBBAAABBBBAAAAAABABAAABABAABAAB-BABBABABBBABBBABBBABAAABABBB

*000252_00394891 BABA-B-BBAB-AABABAB-BAAABBBBBBAAAAAAAAA-BB-ABBBAABAA--ABBBBBA-BBABAABA-B-AABAABB-AAABAAAA

*000252_00561505 BA-A-B-BBABBAABABABBBAAABBBBBBAAAAAAAAABBB-ABBBAABAAABABBBBBABBBABAA-AAB-AABAABBBAAABAAAA

*000253_00227305 A-BBBA-BBAABBBABBBAAB-ABAAA-BBB-A-ABBAA-BBBBB-BBBBBA-AABB-AAAABBA-BBA-BBBBAAB--BABBB---AA

*000254_00352101 BABBBBABBBABAB-BBAABBBBBAABABABAABAAABABBBAAAA-ABBBAAABBBAA-BABABBAAAAABBABBAAA-BBBAABAAA

*000256_00018335 BBAAABAABBBAAAB-BAABB-B-ABBAAAA-AB-AAA--AAAABBB-BAABA-BABAAB-BBBBAABA-AAABBAABBAAAABBABBB

*000256_00159700 B-AAABAABBBAAAB-BAABBAB-ABBAAA--A-AAAA-A-AABB-BBBAAB-ABABAABABBBBAABA-AAABBAAB-A-AABBABBB

*000257_00304834 AABBBABABBBBABABBAAABABAAABAABABBB-BBBBBBBBBABBBBBBABABBABBBBBBABBBBBAB-BAABABABBBBB-AAAA

*000257_00397691 AABBBABABBBBABABBAAABABAAABAABABBB-BBBBBBBBBABBBBBAA--BBABBBBAB--B-BBAB-BAABABABBBBB--AAA

*000257_00462624 AABBBABABBBBABABBAAABABAAABAAB-BBBBBBB-BBB-BABB-BBAABABBABBBBABABBBBBABAAAABABABBBBBAAAAA

*000257_00462744 A-BBBABABBBBABABBAAABABAAAB-ABABBBBBBBBBBBBB--B-BBAAB-BB-BBBB--ABBBBBABAAAABABAB-B-B-A-AA

*000257_00471875 AABBBABABB--ABABBAAABABAAABAABABBB-BBBBBBBBBABB-BBAA-ABBABBBBABABBBBBABAAAABABABBBBB-AAAA

*000258_00090553 AAABAAAABBA-BABABBBAAAAABAA-BAABBBBAABBBBABB---BBAABBBABBAABAAABAABBBBAABBAB-ABAAAAAAB-BB

*000258_00336344 AAABAAAABBA-BABABBBAAAAABAABB-AB-B-AABBBBABB--ABBAABBBABBAABAAABAABBBBAABBAB-ABAAAAAAB-BB

*000258_00487981 A-ABAAAABBABBABABBBAAAAAB-ABB-A-BB--ABBBBA-B--A-BAABBBA--A-B-AA--ABBBB---BA-AA-A--A--BBBB

*000258_00519861 AAABAAAABBA-BABABBBAAAAABAABB-AB-B-AABBBBABB-AABBAABBBABBAABAAABAABBBBAABBAB-ABAAAAAAB-BB

*000258_00542589 AAABAAAABBA-BABABBBAAAAABAABB-AB-B-AABBBBA-B-AABBAAB-BABBAABAAABAABBBBAABBAB-ABAAAAAAB-BB

*000258_00544052 AAA-AAAABBA-BABABBBAAAAABAABB-AB-BBAABBBBABB-AABBAABBBABBAABAAA-AABBBBAABBAB-A--AAAAAB-BB

*000258_00548646 AAABAAAA-BA-BABABBBAAAAABAABB-A--BBAABBBBA-B-AABBAABBBABBAABAAABAABBBBAABBAB-ABAAAAAABABB

*000259_00174363 BBBA-BBBAABAABABBAABABAAABABBBBABBBAAAAAAA-BABAABBBBBA-ABBAABAABBBAAAABABBAB-AAA--BBA-ABB

*000260_00061557 BA-ABBAAABBAA-BA-ABA--A-AABBAABAAA--BABBA--AAAA-ABBB-AA--AA-B--BAAABABBA-A-BB-BAB--A-ABBB

*000260_00073349 BABA--AAABBAAABA-ABABBABAABBAABAAA-BBABBABBAAAAA-BB-BAABAA-ABBB-AAABABBABAB-BA-ABBB---BB-

*000261_00132142 A-ABAAA-ABABABBABAABBAABBBBBBABBABA-AAABAABBBAABABBABABAAAABAABBABB-BAABABBBBAAB-BBAAAAAA

*000261_00317794 A--BAAABABABABB--AAB-AAB-B-BBA-BABAAAAABAA--BAABA-BABABAAAABAA--A-B-BAA-ABBBBAABBBBA-AAAA

*000261_00336863 ABABAAABABABABBA-AABBAAB-BBBBABBABAAAAABAA-BBAABABBABABAAAABAABBABB-BAABABBBBA-BBBBAAAAAA

*000261_00441437 ABABAAABABBBABBA-AABBAAB-BBBBABBABAAAAABAABBB-ABABBABABAAAABAABBABB-BAABABBBBAAB-B-AAAAAA

*000262_00574204 BB-AABBBABABBBBBBBBA-BAABB-BA-BABBBB-AABBA-AB-BABA-AABABBBBBBA-AAAAB-AAAB-BBBBBAABBAAABAA

*000265_00182220 A-BBBAABAABABBB-BBBAABAA-BBABAABBABAABBBBB-BBAB-BBBBB-ABBBBB-BAABAAABAABBAAABB-BABBBAAAB-

*000267_00162082 BBAAABBAB-ABBAAABBBBBAABABABBAABBBAAAA-ABABBAABBABABBAAAABB-ABAAAB-BBBABAABBAABBBA--AAABB

*000268_00033877 BABBBBABBABBBAABABBAABBAABABABBBAA-AABB-ABBBB-ABBBBAAABBBBABABABA-BAAAB-BBBA-A--BBBBBBBAA

*000268_00280169 B-BBBBABBA-BBAABABBAABBAABABABBBAAAAABBBABBBBAABBBBAAABBBBABAAABA-BAAAB-BBBABAA-BAABBB-AA

*000268_00327640 BA-BBBABBA-BBAABABB-ABBA---BABBBAAABBBBBAB-BBAABBBBA-ABBBBAB-AABA-BAAAB-BBBABAABBAABBBB-A

*000269_00073522 AABABA-ABBBBAB-BB-AABABABABAABBBBB-BBB-BBABBABBBBBAABAB-ABABBAAAB-BBB-BAAABBABABABBBAAAAA

*000269_00238416 -AB-B--ABBBBABABBAAABABABABAABBBBB--BB-BBABBABB-BBAABABBAB-BBAAABB-BBABAAABBABABABB-AAAAA

*000269_00294190 AABABBBABBBBABA-BAAABA-ABABAABB-BBBBBB-BBA-BABB-BBAABABB-BABBAAABBBBBABAAAB-ABABABBBAAAAA

*000270_00066903 B-AAABBBBABABB-A-BB-AA-ABB-AAABBAABAAAAAAA-BBBBA-BBAB-AABAAABBABBA-AAAAAAA-AAAAAAAA-ABBAA

*000270_00119957 B-AAABBBBABABB-ABBB-AA-ABB-AAA-BAABAAAAAAA--B-B--BBAB-AABA-ABB-BBA-AABAAAA--AA-AAA----BAA

*000270_00133471 B-AAABBBBABABB-A-BB-AA-ABB-AAABBAABAAAAAAABBBBBA-BBAB-AABAAABBABBA-AA-AAAA-AAAAAAAAAABBAA

*000270_00174985 B-AAABBBBABABB-A-BB-AA-ABB-AAABBAABAAAAAAA-BB-BA-BBAB-AABAAABBABBA-AA-AAAA-AAAAAAAAAABBAA

*000270_00381837 B-A-ABBBBABABB-AABB----ABB--AAB-A-BAAAAA--BBBBBA-B-ABBAABAAA-BABBABA--AAAA--AA--AA-AABBAA

*000271_00497069 A-AAAA--BAAABBA-BBB-BBB-B-BBBAAA-A-BBBABABBA-BAABBB-BBA-BBBAABAB-BBBBBBABA-AAABAABBAAA-BB

*000272_00209996 BABBBBAABBABABABBAABABBBAAAABABB--AAABBAAAA-AABABABABABBBABBBAABBBB-ABBBAABABBABBBBAAAAAA

*000274_00241771 BBBABBAA-AAABB-ABBBBBABB-ABBAABBBBBABABBBBAB-ABABBAABABBBBBABAB-BAABABABAABAAABBBBBBBBAAA

*000274_00268918 -BBABBAABAAABB-ABBB-BABB-ABBAABBBBBABABBBBABA-BABBA-BABBBBBA-ABABAABABABAABBAABBBBBBBBAAA

*000274_00403521 BBBABBAABAAABB-ABBBBBABB-ABBAABBBBBABA-BBB-B--BABBAA-ABBBBBABAB-BAABABABAABBAABBBBBBBB-AA

*000274_00415703 BBBABBAABAAABB-ABBB-BABB-ABBAABBBB-ABABBBBAB-ABABBAA-ABBBBBABAB-BAABABABAABBAABB-BBBB-AAA

*000274_00435537 BBBABBAABAAABB-ABBBBBABB-ABB-ABBBBBABA-BBB-B-ABABBAA-ABBBBBABAB-BAABABABAABBAABBBBBBBBAAA

*000275_00030800 BBAAABA-BBAABB-AABABB-BBBABBB-AA-AABBAB-BAA-BBAABBAABBA-AAB-BBBAAAAB-B-ABAABB-B--B-AABAAA

*000275_00153075 BBAAABAABBAABBAAABABBBBBBABBBAAA-AABBAB-BAA-BBAABBAABBABAAB-BBBAAAABB-AABAABB-BABBBAABAAA

*000276_00746814 B-ABABABBB-ABABBBBA-AAAAB--BBAAA-BB-ABBBBAB--A-BBAA-BBABB-ABAAABAABBABAABBA--ABAAAAA-B-BB

*000277_00226529 ABAAAABBAABABBAA-B--AA-ABB-AAA---B-AAAAAAB--BBBB-ABAB-A-BAAAABBBBABABABABABAAAAAABB-ABBAA

*000277_00595891 AAAAAABBAABABBAA-BAAAA-ABB-AAAB----AAAAA-BAA-BBB-ABAB-A-BAAAABB-BABABABABABAAAAAABBAABBAA

*000277_00705399 A-AAAABBAABABBAA-BA-AA-ABB-AAA-A---AAAAAABA---BB-ABAB-A-BAAAABBB-ABABABABABAAAAAABBAABBAA

*000278_00385665 BBBABBABAAABBABAABAAAAAAABBBBBBAABA--AABABABAABBA-AAABAABAAAABABB-AA-AABAABAA-ABBAABB-AAA

*000280_00056800 BAAAABBABBAAAABABAABAAAABB-BAAAA-B-AABBBBABBBBABBABBBBABB-B-AAABAAAAAAAABAABBAAAAAAAAAABB

*000280_00355390 BAABABBABBAAAABABAABAAAABB-BAAAA-B-AABBBBABBBBABBABBBBABB-B-AAABAAAAAAAABAABBAAAAAAAAAABB

*000282_00325938 B-AAABAABAAABBAABBBBBABB-A-BAABBBBBABABBBB-A-ABABBAA-ABBBBBABAB-BAABABABAABBAABBBBBBBBAAA

*000282_00440858 B-AAABAABAAABBAABBBBBABB-ABBAAB-BBBABABBBB-ABABABBAA-ABBBBBABAB-BAABABA-AABBAABB-BBBB-AAA

*000282_00495398 B-A-ABA-BAAAB-AABBBBBABBAABBAABBBB--BAB-BB-A-ABABBAAAABB-BBABA---AABABABAABBAA-BBB--B-A-A

*000282_00535938 BBBABBAAB-A-BBAABBBBB-BBA-BBAABBBBB-BABBBBAB-ABABBAABABBBBBABAB-BAABABABAABBAABBBBBBBBAAA

*000283_00301266 BBBABBA-ABABBABBBBABAAABB--ABABBBBBBBBBAA-ABAB-AABA-ABABBAA-A-ABABAAAAB-ABBAAB-A-AABBABAA

*000283_00398853 BBBABBAAABABBABBBBABAAABBA-ABABBBBBBBBBAAAABA-AAABA-ABABBAAAAAABABAAAABAABBAABAABAABBABAA

*000283_00449861 BBB-BBAAABABB--B--ABA-ABB-BAB-B--B-BBBBAAA-B---AABA-ABA-BAAAAAA-ABAA-ABAABB-A--ABAA--A-AA

*000285_00288163 AA-BBAAAABBAABBABAAABBABABABABAAABA-AABBAABBA-BBBAAABAABABBBABBBABBAAABBBBAAABABBAAA-BAAA

*000285_00293364 A-BBBAAA-BBAABBABAA-BBA-ABABABAAABABA-BBAA--A-BBBAAABA-BABBBABB-ABBAAA-BBBAAA-A-BA--B-AAA

*000287_00090262 BBBABBABAABAABABBAABABAAABABBBBABBBAAAAAAAABABAABBBBBA-BBBAABAAB-BAAAABABBABAAAABBBBB-A-A

*000288_00030243 AAABAAABA-ABBABAA-BA--AABBB-ABBBAA-BBA-AB---B----BBA-AABA--AA-BABB-B-A-BABBBBAABAAAAAAA-A

*000288_00599502 AAA-A-ABA-ABB-BAABBA-AAABBBBABBBAABBB-BAB-B-BBBB-BBA-AABABBAABBABBAB-AB-AB-BBAABAAAAAAAAA

*000289_00205204 BBAAABBBB-ABBAAABBBBBAABA-ABBAABBBAB-A-ABABBAABBAAAABAAABBB-ABAAAB-BBBA-AABBAABABAA-AAAAA

*000289_00207891 BBAAABBBB-ABBAAABBBBBAABAAABBAABBBA--A-A-A-BAABBAAAABAAABBB-ABAAAB-BBBA-AABBAABABAA-AAAAA

*000289_00220084 BBAAABBBB-ABBAAABBBBBAABA-ABBAABBBA--A-ABABBA-BBAAAABAAABBB-ABAAAB-BBBA-AABBAABABAA-AAAAA

*000289_00425797 BBAAABBAB-ABBAAABBBBBAABA-ABBAABBBA--A-ABABBA-BBAAAABAAABBB-ABAAAB-BBBA-AABBAABABAA-AAAAA

*000289_00542679 BBAAABBAB-ABBAAABBBBBAABA-ABBAABBBA--A-ABABBAABBAAAABAAABBB-ABAAAB-BBBA-AABBAABABAA-AAAAA

*000290_00329801 BB-BABABAA-BBAB-BBB-BB-A-ABA-BB---B-BABBBB-BABB-A-A-AA-BAAAB--BAABBB-AAA-AAA-AB-AAAA--BBB

*000291_00041159 ABABAABBA-ABBAAAAB-AAAABAABABB-ABBBBBB-AA---BBABAA-AB-AAABABBAABBBB-B-BBBABAAAABAAAABBBAA

*000291_00097426 ABABAABBA-ABBAAAAB-AAAABAABABB-ABBBBBBAA----BBABAA-ABBAAABABBAABBBB-B-BBBABAAAABAAAABBBAA

*000291_00677697 AAABAA----ABBABA---BAAABAABABB-ABBB-BB-AA-BBBBA--AAAB--AABABBAA--BB-B-BBBA--AAABAAAA-B--A

*000292_00213662 BA-BABABABABBAABBBBBBAAA-BAAB-AAA-ABBBABBBBAABAA-A-BA-BBABBAABBBBBBABBBBBBBABBBBABBBAAABB

*000292_00509720 B--B-BABABABBAABBBBBBAAA-BAABBAAABABBBABBBBAABAA-A-BAABBABBAABBBBBBABBBBBBBABBBB-BBBAAABB

*000293_00085623 BBB-BBBBABABBAABBBBBBAB-ABBBBBABAA-AABB-BAAABABBAABBBABAABBABABAB--BBAABAAAABBBABBBABABBB

*000293_00171750 B-B-BBBBABABBAABBBBBBAB-ABBBBBABAAAAABB-BAA-BABBAABBBABAABBA-ABABA-BBAABAAAABBBABBBABABBB

*000294_00229071 A-BABABBABBAAA-B-ABABBBABBBBBA-AAAABBBABAAAAA-AB-AABABAAABABAABBB-BBBAAABBABBBAAB-BAAABBB

*000294_00273473 AABABABBABBAAA-B-ABABBBABBBBBA-AAAABBBABAA-AABAB-AA-ABAAABABAABBB-BBBAAABBABBBAABBBAA-BBB

*000295_00054864 BAAB-BBBAABBBABBABBBABBA--BABA-ABBBAAAAAB--BA---BBAB--ABAB-B-AAABBAAABBBBAB-AB---BBAABBBB

*000296_00067987 BABABBBABABBAABBBAABBBBBABBABABAABAAABAABBAAAA-BBAAAAABBBAABBABABB-AABBBAABBAAB-BBBBBBBAA

*000296_00147023 BABA-B-ABA-BABBBBAABBBBBAABABABAABA--BAABB-AAABBBAAAA-BBBAABBABABB-A-BBBAABBAAB--BBB---AA

*000298_00096822 B--A-BBBAABABBBBABBABBBABABABBBAAAABBAAABBABBBBABAABBABBABBBAABBBABA-ABBBABAAABAABBAABABB

*000299_00059181 A-AAAAAAABBAB-ABB-AABB--ABA-BA--A-AAA--ABAAABBABBA-BA-ABABAAAABBBBBA-AAA-AABA-AA-B-A-ABBB

*000299_00202728 AAAAAAAAABBABAABBBAABB-BABA-BAB-ABAAA-AABAAAB-ABBAABAAABABAAAABBBBBABAAABAAB-BAABBBAAABBB

*000301_00276961 B-ABAB-BBABBAAB-BABBBAAABBBBBBAAAB-AAAAB-BAABABBABAAABABBBBBABBBBBAA-AABAAAB-ABBBAAABAAAA

*000303_00153133 B-AAAB-BBAABBBBABBABBAAB-AAABAAAABBAABAABB-BBA-A--AAAAAAA-B-BABAABABABA-BAABBABBBAAABAAAA

*000303_00209810 BB-AABA-BAABBBBABB-BBAABBAAABAAAABBAABAA-B-B-A-ABAA-AAAAA-B-BABAA-ABABABBAAB-ABB-AA--AAAA

*000304_00390807 A-BABAABAAABBABAABBBBBABBA-AB-BBABA-BBBBBBBBB-AA-AAABAABBABAAABAB-BBBA-BBABABA-BBAAA-A-AA

*000304_00391121 A-BABAABAAABBABAABBBB-ABBA-AB-BBABABBBBBBB--B-AA-AAABAA-BABAAABABABBAA-BBABABAABBAAAAABAA

*000305_00231237 BA-AABA-ABBAAABBBA-ABABAAABAAAB-A-ABBBB--ABAAAAAA-BAA-ABAAAA-AAAAAB-ABBABABBBABABBB-A-BAA

*000306_00100827 A-A-A-ABA-BBB--B-B-ABB-BAA-B-BA-B--BBBBBAB--BB-AAAAABB-BABBAABBAB-ABA-BABBAA-AAAAA---A-AA

*000306_00131757 AAABAAABA-BBB-ABBBBABBA-AABBA-ABBABBBBBBABB-BB-AA-AABAABABBAABBAB--BA-BABBAA-AAA-AAA-AAAA

*000306_00437567 A-A-AAABA-BBB-ABBBBABBABAABBAAABBABBBBB-AB--BBAAA--A--AB-BBAABBAB--BA-BABBAA-AAAAA-A-AAAA

*000307_00075855 BAABABAABBA-BABBBB-BAAAABA-BA-AA-B-AABBBBABB-AABBAABBBABBAABAAABAABBABAABBAB-ABAAAAAAB-BB

*000307_00479106 BAABABAABBA-BABBBBABAAAABABBABAAAB---BBBBA-B-AABBAABBB-BBAABAAABAABBABAABBA--ABAAAAAABBBB

*000308_00072198 ABBBB-BB-BAB-A--AABA---A----A-B-AB-AABABBB-A-BBB-AABA-BABABAA-ABABA-AAABBBBAAA-AABBAA-B-B

*000308_00116029 ABAB-ABBAB---ABAAABAA-AAA-A-A-BBAB-AABABBB-A-BBBAAABAABA-A-AAAABAB-BAAABBB-A-BBAABBAA-B--

*000308_00172603 AB-B-ABBAB-BAABAAABAABAAABAAA-BBABBAABABBBAA-BBBAAABAABABABAAAABAB-BAAABBBBAA-B-ABBAAAB--

*000308_00291841 AB-BBABBAB-BAABAAABA-BAAABAAA-BBAB-AABABBBAA-BBB-AABAABABABAAAABAB-BAAABBBBAA-BBABBAAABB-

*000308_00369902 AB-B-ABBAB-BAABAAABAABAAABAAA-BBAB-AABABBBAA--BBAA-BAABABABAAAABABBBAAABBBBAA-B-ABBAAAB--

*000308_00379782 A--B-ABBAB-BAABAAABAABAAABAAA-BBAB-AABABBBAA--BBAAABAABABABAAAABAB-BAAABBBBAA-B-ABBAAAB--

*000309_00613752 -AAAA-BBAAAAAAA-AABBAAABBABAB-BAABA--B-BA--A--A-AABABBBABBAABBAB--ABBBBB-ABAABB-AAABBAAAA

*000310_00069000 AABB--AABABABBAAA---BABBB--AAB---AAAAAB-BB-B-AAAABAABBBAAABBA---BAA--A-BBB-AB-ABBBBAA--AA

*000310_00109362 AABBBAAABABABBAAABBBBABBBBAAAB-BAAAAAABBBBABBAAAABAABBBABABBA-ABBAABBAABBB-AB-ABBBBAAABAA

*000310_00204843 AABBBAAABABABBAAABBBBABBBBAAAB-BAAAAAABBBBA-BAA-ABAABBBABABBA-ABBAABBAABBB-AB-ABBBBAAABAA

*000310_00209112 --BBBAAABABA-BAAABBBBABBB-A-AB-B-AAAAABB----B-AAABAA-BBA-ABBA---BAABBAABBB-AB-ABBB--AABAA

*000310_00223856 A-BBBAAABABABBAAABBBBABBBBAAAB-BAAA-AABBBBABBAAAABAABBBABABBA-ABBAABBAABBB-AB-ABBBBAAABAA

*000310_00395116 AABBBAAABABABBAAA-BBBA-B-BAAAB--AAAAAABBBBABBAAAABAABBBABABBA-ABBAABBAABBB-ABAABBBBAAABAA

*000311_00031901 ABAAAA-BAABABABBBBAAAABAAABAAABBBBB-BBABBBBAABAAB-ABBBAAAABABBABBAABBABBBABA--ABABBBBABBB

*000311_00266713 A-AAAABBAABABABBBBAAAABAAABAAABBBBBBBBABBB-AA-AAB-AB--A-AABABBABBAABBAB-BABAB-AB-BBBBA-BB

*000311_00266799 A-AAAA-BAABABABBBBAAAABAAABAAABBBBBBBB-BBBBAA-AAB-ABBBAAAABABBABBAABBABBBABAB-ABABBBBABBB

*000312_00075689 A-BABABAABABAB-BBAABAAABBB-ABAA-BBB---BBBA-BABA-ABABBABBBABA-AABAAA-ABBABABA-BBABAAA-A-BB

*000312_00226721 B-BABBBAABABABBBBAABAAABBB-ABAABBBBA--BBBAABABAAABABBABBBABAAAABAAAA-BBABABA-BBA-AAAAAABB

*000313_00017373 BBBBBBA-AAB-B-ABABB-AAAAAAABABAB-A-B-A--AB-A---BABB-BAABBB-ABAAAB-A-AAB-AAAABB-A-BBA--ABB

*000313_00307732 BBB-BBAAAA-BBBABABB-AAAAAAABABABAAABBA-AABAAA--BBBABBAABBB-ABAAABBA-AA-BAAAABB-ABBBAAAABB

*000313_00319450 BBB-BBAAAA-BBBABABB-AAAAAAABABABAA-BBA-AAB-AA--BBBABBAABBB-ABAAABBA-AABBAAAABB-ABBBAAAABB

*000313_00373999 BBB--BAAAA-BB-ABABB-AAAAAAABABABAAABBA-AAB--A--BBBABBAABBB-ABAA-BBA-AABBAAAA-B-ABBBAAAABB

*000313_00402663 BB--BBAAAA-BBBA-ABB-AAAAAAAB-BABAAABBAAAABA-AAB-B-ABBAABBBBA-AAABBA-AA-BAAAABB-A-B-A--A-B

*000315_00077724 A-AA-AAA-BBA-ABAAABABAABBBBAAABBAAABBBBAABBBBBBAB--BB-AA-AAABAAB-AABABAAABA-A-AAABBBB-BBB

*000315_00208503 AAAAAAAAABBA-ABAA-BABAABBBBAAABBAAABBBBA-BBBBBBABBABBAAAAAAABAABAAABABAAABAAA-AAABBBB-BBB

*000316_00014946 AABBBAAABABABBAAABBBBABBBBA-AB-BAAAAAABBBBABBAAAABAABBBAAABBAAABBAABBAABBB-AA--BBBBAAABAA

*000316_00025877 AABBBAAABABABBAAABBBBABBBBAAAB-BAA--AABBBBABBAAAABAABBBAAABBAAABBAABBAABBB-AA-ABBBBAAABAA

*000316_00231426 AABBBAAABABABBAABBBBB-B-BBAAABA-AAAA-ABB-BABB-A-ABA-B-BA-ABBA-ABBAABBAAB-AAA---BBBBA-A--A

*000316_00327443 AABBBAAABABABBAABB-B-ABBBBAAAB-BAAAAAABBBBABBAAAABAABBBAAABBA-ABBAABBAABBAAAA-ABBBBA-A-AA

*000317_00251321 B-BABBABABAAABBAAABABBABAABBAA-AAAABBABBAB-AAAAAAABBBAA-AAAAABBBAAABABA-BABABABB-BBABA-BB

*000317_00277268 BBBABBABABAAABBAAABABBABAABBAA-AAAABBABBAB-AAAAAAABBBAA-AAAAABBBAAABABA-BABABABBBBBABA-BB

*000318_00008953 A-AAAABAAABABBAA-BA-AA-ABB-AAA-A---AAAAAABAB-BBB-ABAB-ABBAAAABBBBABABABABABAAAAAABBAABBAA

*000318_00011295 A-AAAABAAABABBAA-BA-AA-ABB-AAA-A---AAAAAABA--BBB-ABAB-ABBAAAABBBBABABABABABAAAAAABBAABBAA

*000318_00011739 A-AAAABAAABABBAA-BA-AA-ABB-AAA-A---AAAAAABA--BBB-ABAB-ABBAAAABBBBABABABABABAAAAAABBAABBAA

*000318_00189091 ABAAAABAAABABBAA-BA-AA-ABB-AAA-A-B-AAAAAAB---BB--ABAB-ABBAAAABBBBABAB-BABABAAAAAABBAA-BAA

*000318_00270770 A-A--AB-AABAB-AA-BA-A--ABBAAAA------AAAAABA-B-BB-ABAB-ABBAAA-BBBB--ABA-ABABAAAAAABBAABBAA

*000319_00391598 BABBBBAABBABABABBAABABBBAAAAB-BB--AAABBA-B--A-BABABABABBBABBBAABBBBAA-BBBABABBABBBBA-AAAA

*000319_00420413 BABBBBAABBABABABBAABABBBAAAABABB---AABBA-BA-AABABABABABBBABBBAABBBBAABBBBABABBABBBBAAAAAA

*000320_00232424 B-AAABAAAABAAABAAAAABAAAAABBBAABBABBBAAAAABAABABBAB-AABAAABABBBB-BBAAABBAAABBBAABAAAAAABB

*000321_00077637 B-BBBBA-BB-B--ABBAABABBBAAAABA---B--ABBAAB--ABBABABA--B-B-BBBAABB-BAA--BBABABB-BBBBAA-AAA

*000321_00110896 B-BBBBAABBBB-BABBAABABB-AA-ABABBB-BAABBA----ABBABABABABABABBBAABBABAABABBABABB-BBBBA-AAAA

*000322_00348898 BBAAABAAAABBABABBABABAA-BA-AAAB-BBBAA-ABBBBBBBBAABABAAAABABBBABABBAAAAAABBBB-BAABAAABABBB

*000324_00186696 ABAA-AAA-BBABAABBBAABBB-A-A-BABAABAAA-AABA-ABBABBAABAAABABAAA-BBBBBABAAABAABABAABBBAAABBB

*000326_00186339 BB-A---BAAAABB--BB-ABBBBAAAAAABB-B-ABA-BA--BBAAA-BAABBBBBBAAB-B-BAABABAB-A-AAABBB---BBAAA

*000326_00188542 BBBABBABAAAABB-ABBBABBBBAA-AAABBBBBABABBA-AB-AAABBAABBBBBBAAB-B-BAABABABAABAAABBB--BBBAAA

*000328_00065869 AAAAAAABAABAAABBAAAABABBBAAAABBBBBAAABBABBBABBBAAAABABBAAAAAAABB-AABABBBBABABBBBAAABBABBB

*000328_00068009 AAAAAAABAABAAABBAAAABABBBAAAABB-BBAAABBABBBABBB-AAABABBAAAAAAABB-AABABBBBABABBBBAAABBABBB

*000328_00235333 A-A-AAABA-BAAABBAA-ABA-BB-AABBBBB--AABB-B--A---A-AABAB-AAAAAAABB-AA-A-BBBABABB-----BBABBB

*000329_00210386 AAABAAABAAABB-BAABBABABABBBBABBBAABBBA-AB-BBB-BB-BBA-AABA-BAABBABBAB-A-BABBBAAABBAAAAAAAA

*000329_00395003 AAABAAABAAABB-BAABBABABABBBBABBBAABBBA-A--B-B-BB-BBA-AA-A-BAABBABBAB-A-BABBBAAABBAAAAAAAA

*000330_00265126 BAABABABAABBBBBBBBBABBAAAABAABBABBBBB-BBBBABABBBA-AB-AABAAAB-ABAABBBAAAABABBABBAAAAABBBBB

*000330_00482988 BA-BAB-BAABBBBBBBBBA-BAA--BAABBABBB-B-B--B-B-BB--BABA-AB--ABBA-AA-BB-A-AB-B-ABBAAA-A--BBB

*000332_00133619 B-ABABAAABABA-ABBAAAAAAABBABBABBAAABBAABAABAAABB-BB-AAABAAAAAAAABBBBBAABAABBAABBBAAAAABAA

*000332_00150820 BAABABAAABABA-ABBAAAAAAABBABBABBAAABBAABAA-A-AB-BBB-AAA-AAAAAAAABBBBBAABAABBAAB--AAA--BAA

*000332_00179148 BAABABAAABABA-ABBAAAAAAABBABBABBAA-BBAABAABAAABBBBBAAAABAAAAAAAABBBBBAABAABBAA-BBAAAAABAA

*000332_00183525 BAABABAAABABA-ABBAAAAAAABBABBABBAAABBAABAABAAABBBBBAAAABAAAAAAAABBBBBAABAABBAA-BBAAAAABAA

*000332_00218203 BAABABAAABABA-ABBAAAAAAABBABBABBAA-BBAABAABAAABBBBBAAAABAAAAAAAABBBBBAABAABBAA-BBAAAAABAA

*000333_00027166 ABBBBABBAB-BAABAAABAAAA-ABAAA-BBAB-BABABBBAA--BBAAABAABABBBAAAABAB-BABABBBBBB-B-ABBAAAB--

*000336_00286748 BBAAAB-ABA-ABBA-BBAB-BB-B-BBBAAAAAABBBABABBA-BAAABBBBBAB-BBAABA-BBABBBBABA-AAA-AABBB-AABB

*000336_00441640 BBAAAB-ABAAABBA-BBABBBB-B-BBBAAAAAABBBABABBA-BAAABBBBBAB-BBAABA-BBABBBBA-A-AAABAABBB-A-BB

*000336_00446672 BBAAABBABAA-BBA-BBABBBBBB-BBBAAA-AABBBABABBA-BAAABBBBBABABBAA-A-BBABBBBA-A-AAABAABBB-A-BB

*000336_00450739 BBAAAB-ABAAABBA-BBABBBB-B-BBBAAAAAABBBABABBA-BAAABBBBBAB-BBAABA-BBABBBBA-A-AAABAABBB-A-BB

*000337_00052242 BAA-ABBBABBBBAABABBAAABAAAABBBBBBBBAABBBABAABABBBBBBA-ABABABABABABB-ABBABABBBAAABBBBBBABB

*000337_00120938 BAA-ABBBABBBBAABABBAAABAAAABBBBBBBBAABBB-BAABABBBBBBA-ABABABABABABB-ABBABABBBAAABBBBBBABB

*000337_00123262 BAA-ABBBABBBBAABABBAAABAAAABBBBBBBBAABBBABAAB-BBBBBBA-ABABABABABABB-ABBABABBBAAABBBBBBABB

*000337_00341971 B-BABBBBABBBBA-B-BB--AB-AAABBB-BBBBAABB-AB-ABAB-BBBBA-ABABABABABA-B--BBABABBBAAA-B--BBBBB

*000338_00047849 B-AAABAAAABA-BBAA-AA-BBA-ABABABABB-BBAABBBBBB--BAAA--BBAABBBBAB-B--ABBAA-ABAABAABBBAA--AA

*000338_00083331 BBAAABAAAABA-BBAA-AABBBABABABABABBBBBAABBB-BB-BBAAAABBBAABBBBABBBA-ABBA--ABAABAABBBAABAAA

*000339_00030205 AAAAAA-BAB--ABABAABBB-ABBBAAABBAAAAAA-AB--ABAABBBABABA-BAAABBBBABAAABBBABA-ABBBAAAABB-BAA

*000339_00133155 AAAAAA-BAB--ABABAABBBAABBBAAABBAAAAAA-ABA-ABAABBBABABA-BAAAB-BBABAAABBBABA-ABBBAAAABB-BAA

*000339_00142206 AAAAAABB-B--ABABAABBBAABBBAAABB-AAAAA-ABA-ABA-BBBABABA-BAAAB-BBABAAABBBA-A-ABBBAAAABB-BAA

*000340_00343850 BBAA-BAA-ABAAABAAAAA--AAAA-BBAA-BAB-AAAAAABAABABBABAA-AABABABBBBABBAAA-B-AABBAAAAAABBAAA-

*000341_00287804 ABBBBABAAABAA-BAAAAAABABAAAAB-ABBBBAABB--A-ABBABBBBABBAABA-AAAABBBBBAB-BBAABBBABBBBBBAAB-

*000341_00329222 ABBBBABAAABAA-BAAAAAABABAAAABBABBBBAABBAAABABBABBB-AB-AA-A-AAAAB--BBABABBAABBBABBBBBBA---

*000342_00214940 ABAAAAABBABABABBABAAAABAAABABA-BBBBBBBABAB-ABBAAABABBBAAAABAABAABAAABBABBABBBAABABB-BAB-B

*000342_00288778 ABAAAAABBABABABBABAAAABAAABABABBBBBBBBABBBBABBAAABABBBAAAAB-ABA-BAAABBABBABBBA-BABBBBABBB

*000342_00300105 A-AAAAABBABABABBABAAAABAAABABABBBBBBBBABBBBABBA-ABABBBAAAABAABAABA-ABBABBABBBAABABB-BABBB

*000343_00228127 ABBBBAABBA-BB-ABBBAAB-ABAAABBBBAAAABBAABBB-BB--BBBBA-A-BBBAAAABBA-BBA-B-BBAAB-AAABBBBABAA

*000343_00228213 A-BBBAABBAABBBA-B-AAB-AB-AAB-BB-AAABBAA-BB-BBA-BB-BA-AABBBAAAABB---BA-B-BBAAB--BABBBBA-AA

*000344_00036312 BAABABBBABBBBBABBBBBABAABBB-AB-ABB-BBABBBBABABBBB-BB-A-BBABBBAABAABBAAABB-ABAABBAAAABBBBB

*000346_00009676 ABBABAAAB-BABBBBABAABBAABAABBBA-BABABBBAAAABB-BBAAAB-ABBBABA-BAABABAABAAABABBA-ABAA-ABBAA

*000346_00289729 ABBABAAAB-BABBBBABAA-BAABAABBBA-BA-ABBBAAAABBBBBAAA--ABBBABA-BAABABABBAAABA-BABABAA-ABBAA

*000346_00439891 ABBABAAAB-BABBBBABAA-BAABAABBBA-BABABBBAAAABBBBBAAAA-ABBBABABBAABABABBAAABA-BABABAA-ABBAA

*000347_00006061 ABBBBAAAABBBABBAAAABBBAB-BABAABA-BABAABBAABBBBABABAABABAABABABBBABB-AABBABBBAAABBAAAAAAAA

*000347_00063996 ABBBBAAA-BBBABBAAAABBBAB-BABAA-A-BABAABBAABBB-ABABAABABAABABABBBABB-AABBABBBAAABBAAAAAAAA

*000347_00316268 ABBBBAAAABB-ABBAAAABBBAB-BABBA-A-BABAABBAABBB-ABABAABABAABABAABBABB-BA-BABBBAAABBA-AAAAAA

*000348_00175185 BBAAAB-BAA-BBABBBBAAAABAAABAAABBBBBBBBABBBBAA-AAB-ABBBAAAABABBAB-AABBABBBABAB-ABABBBBABBB

*000349_00248858 ABB-BAABAABABBB-BBBAABAA-BBABAABBABAABBBBBABBAB-BBBBBBABBBBB-AAABAAABAABBAAABBBBABBBBAAB-

*000349_00416853 ABB-BAABAAB-BBB-BBBAABAA-BBABAABBA-AABBBBB-BB-BABBBBBBABBBBB-AAABAAABAABBAABBBBBABBBBAAB-

*000350_00036634 -AA-A-ABA-BBBBABBBBABBABAABBBBAAB-BBBBABABBB-BBAA-AAB-ABB-BAABBBB-ABABBABAAA-A-AAAAAB-AAA

*000350_00068835 -AA-A-ABA-BBBBABBBBABBA-AABBBBAABAB-BBAB-B-B-B-AAAAABBAB--BAABBBB-ABAB-ABAA---BAAAA-B-AAA

*000350_00368242 --A-ABABA-BBBBABBBBABBABAABBB-AABABBBBABABBBB-A-A-AA--ABB-BAABBBB-ABABBABAAA-A-AAA-AB-AAA

*000351_00104802 -ABAB--ABBBBABABBAAABABABABAABBBB-BBBB-BBABBA-BABBAABABBABABBAAABBBBBAB-AABBABA-ABB-AA-AA

*000351_00153961 -ABAB--ABBBBABABBAAABABABABAABBBBBBBBB-BBABBABB-BBAABABBABABBAAABBBBBAB-AABBABA-ABBBAAAAA

*000351_00262959 -ABAB--ABB--A--B-AAAB-BA--BAABB-BBBBBBBBBA-BA-BA--AABABBABAB-AA-BBBB-ABBAABBABA-ABBBA-AAA

*000352_00000126 BABABBAAAAABB-BBABABBAAAABBBBABAAAAAABAAAAABBBBBBB-AA-BB-A-BBABBBB-BBBBBABBBAABBA-BAAAAAA

*000352_00001724 BABABB-AAAABB-BBABABBAAAABBBBABAAAAAABAA-AABBBBBBB-AAABB-A-BBABBBBABBBBBABBBAABB-BBAAAAAA

*000352_00236841 BABABB-AAAABB-BBABABBAAAABBBBABAAAAAABAAAA-BBBBBBB-AAABB-A-BBABBBBABBBBBABBBAABBABBAAAAAA

*000353_00046377 -BABABBAA-B-BB-BBBBABA-AB-AAABB-BBB-ABB--B---AA-BBAABBABBABBBBB-ABAABBB--A-A-BB-A----AB-A

*000354_00230359 ABBAB-BAAAAABBAA-BBAAAAABBBAAA--AA--AAA-BBAABBBAAABABAA-BAAAB-B-BA-ABA--AA-A-A-AABB-AABAA

*000354_00230445 ABBABABAAAAABBAA-BB-AA-ABBBAAA-AAAAAAAAAABAABBBAAABABAABBAAABABBBA-ABAAAAABAAAAAABBAAABAA

*000354_00380764 ABBABABAAAAABBAA-BB-AA-ABBBAAA-AAAAAAAAAABAABBBAAABAB-ABBAAABABBB--ABAAAAABAAAAAABBAAABAA

*000355_00048791 BBBBBBBBABABBBABBBBABAA-ABBAABB-B-BAABBB-BABB-A-BB----ABBABBBBB-ABAAB-BAAABABBBAAAABB--AA

*000355_00050768 BBBBBBBBABABBBABBBBABAA-ABBAABBABABAABBBABABB-A-BB-A-BABBABBBBBAABAABBBAAABABBBAAAABB-BAA

*000357_00070949 BBABABBAABBBBBABBBBABAA-B-AAABBBBBBAAB-B-BABBAAABB-A-BABBABBBBBAABBABBB-AABABBBBAAABB-BAA

*000358_00017395 ABA-AAAAA-BAAABAAAAABA-AAABBBAABBABAAAAAAABAABABBA-AAAAABABABBBBABBBAABBAAABBAAA-AABBAAAA

*000358_00022110 AB---AAA-ABA-A-A-AAABA-A--BBBAA---BAA-AAAA-AABABB-B-AAAABABABBBBABBB--BBAAABBAAA-AA-BAAAA

*000358_00022196 ABA-AAAAA-BAAABAAAAABA-AAABBBAABBABAAAAAAABAABABBA-AAAAABABABBBBABBBAABBAAABBAAAAAABBAAAA

*000359_00345240 BBBBBBBAABABBBABBBBABAA-ABBAABBABBBAABBB-BABB-AAB--A-BABBABBBBBAABAABBB-AABABBBBAAABB-BAA

*000362_00040275 BBBABBAABABABAABABABABB-B-BBBAAAAAAB-BABAB-A-BA-ABBBBBABABBABBA-BBABBA-ABAB-AAAA--BB---BB

*000362_00085714 BBBABB-ABABABAABAB-BABB-B-BBBAAAAAABBBABABB--BAAABBBBBABABBA-BA-BBABBAAA-A-AAAAAABBBBA-BB

*000362_00174317 BBB-BBAABABABAABABABABBBB-BBB-AAAAABBBAB-B-A-BAAABBBBBABABBABBA-BBAB-A-ABA-AAAAAAB-BBAABB

*000362_00305143 BB---BAA-ABAB-ABAB-BA-BBB--BBAAAAAABBBABAB-A--AAAB-BB-ABABB-BBA-BBABBAAABA-A-AAAAB-BBAABB

*000362_00327774 BBBBBBAABABAB-ABABABABB-B-BBBAAAAAABBBABABBA-BAAABBBBBABABBABBA-BBABBAAABA-AAAAAABBBBA-BB

*000363_00074804 --B----B-BAAAB-B---BBB-B-AB-AB--ABABBB-BAB-A-BAABAAAABBABABAAABA-BBABBAB-ABBABB--AAB-AAAA

*000363_00110911 B--ABBABBBAAABABBA--BBABBABAABB-AB-BBBABABBABB-ABAAAABBAB-B-AABA-BBABBABAABBABB-AAA-BAA-A

*000363_00110997 BABABBABBBAAABABBA-BBBABBABAABBAABABBB-BAB-ABBA-BAAA--BABABAAABA-BBABBABAABBABB-AAABBAAAA

*000363_00114956 B-BABBABBBAAABABBA-BBBABBABAABBAABABBB-BAB-ABBAABAAA-BBABABAAABA-BBABBABAABBABB-AAABBAAAA

*000364_00215237 BBBABBA-ABBA-ABBB-AAAABB-AAAAABAAAAAAB-B-BBAAABB-A-ABBBBABAABAAA-BAABABAA-BAAAA-BBBBAABAA

*000365_00122470 AABABABB-BBAAA-BBAAABB-BBBBBBA-AAAAABBABBA-ABAAB-AABABAAABABAABBBBBBBAAABBABBBAABBBAAABBB

*000365_00203131 AABABABB-BBAAA-BBAAABB-BBBBBBA-AAAA-B-AB-A-ABAAB-AABABAAABABAABBBBBBBAAABBABBBAABBBAAABBB

*000367_00359875 BB-B-BBBAB-AAABABABAABAAABAAABBBABAAABAABB-BABBAAAABAABAAABAAA-BAB-ABAAABBBAA--AA-AAA---A

*000368_00130538 BABBBBAAABBAAAABBA-ABABAAABAAABAAA-BBBB-AABAA-ABABBAAABBAAAABAAAAAAAABBAAABABABAABBBAABAA

*000368_00311069 BABB-BAAABBAAAABBA-ABABAAABAAABAAA-BBBBAAABAABABABBAAAB-AAAA-AAAAAAAA--AAABABABBAB--AABAA

*000368_00311155 BABBBBAAABBAAAABBA-ABABAAABAAABAAAABBBB-AABAABABABBAAABBAAAABAAAAAAAABBAAABABABBABBBAABAA

*000369_00036161 B-BBBBA-ABAABABB-BA--AB-BABBBABBBBBBABAA-A-BB-BABBAAAAB-BBBAAAAAABAAABAABBBAAA-ABBBBBABBB

*000369_00303245 B-BBB-A--B-A-ABBABA-BAB-BABBBABBBBBBABAAAA-BB-BABBAA-ABABBBAA--AAAAAABAABBBAAAA-BBBB-ABBB

*000370_00341638 ABA-AABABB-BBBBABBBBBBBBBAB-AAABBABBBAB-BAABBBBABAA-BBABBABABB-ABAABBBBAABABBBBA-BBBBBAAA

*000370_00366704 ABA-AABABB-BBBBABBBBBBBBBAB-AAABBABBBAB-BAABBBBABAAABBABBABABB-ABAABBBBAABABBBBAABBBBBAAA

*000372_00195952 BABABBBBBABBAABBBAABBBBBABBAB-BAABAAABAABBAAAAABBAAAAABBBAA-BABABBAAABBBBABBAAB-BBBAABBAA

*000372_00360098 BABABBBBBABBAABBBAABBBBBAB-ABA-AABAAABAABBAAA-ABBAAAAABBBAA-BABABBAAABBBBABBAABBBBBAABBAA

*000373_00200605 A-BABABBAAAAABAAAAABBBA-BA-ABBBBBBBBABBBBBBAAAABBAABBAABAABAAABABABAAA--AA-AAABBAA-AABBBB

*000373_00203442 A-BABABBAAA-ABAAAAABBBA-BA-ABBBBBBBBABBBBBBAA-ABBAABBAABAABAAABABABAAAB-AABAAABBAAAAABBBB

*000373_00326567 ABBABABBAAAAAAAABAABBBAABA-ABBBBBBB-ABBBBBBAA-ABBA-BBAABAABAAA-ABABAAAB-AABAAABBAAA-A--BB

*000374_00046646 BABABBB-ABABAABB-AAB-AAAB-AABAAABBBB-ABBBBABABAAABABAA-BBABAAA-AAABBBBBABBBA--BABAAB-AABB

*000374_00393367 BABABBBAABABAABBBAAB-AAAB--ABAAABBBB-ABBBAABABAAABABAA-BBABAAA-AAABBBBBABBBA--BABAAB-AABB

*000375_00113936 BBBBBBBABABBABAABABBABAABAABAAAABA-BBA-AB-ABBBBB-AAAABBBBABABBAABAABAB-AABAABBBA-AABBBBAA

*000375_00397508 BB-B-BBABABBABAABABBABAABAA-AAA-BA-BBABAB-ABBBBB-AAAABBBBABABBAABA-B-BBAA-AABBBA-AABBBBAA

*000378_00255401 BAABABAB-BB-AB-A-A-BBABABBA-BBB-B-BAAAA-AB-AB---B-BAB-B-BABBAABBA-BB-A-BAAB-BAABBA-AAA-A-

*000379_00172998 B-BBBBAAAABBBBABABBBBAAAAAABBB-BBABBBABAABAAAB-BBBABBAABBA-ABBBA-AABBABBAA-ABBAAB-B-AAABB

*000380_00034348 B-BABBBAABBBBABBABAAABABBABBAABA-B-BBABAABBABABAA-ABABABAA-ABBABAABBBBABAABBBBBBBAABBABBB

*000380_00037904 BBBABBBAABBBBABBABAAABABBABBAABA-BABBABAABBABABAA-ABABABAA-ABBABAABBBBABAABBBBBBBAABBABBB

*000380_00120264 B----BBAABBBBABBABAAABABBABBAABA----BABAA-BABABAA-A-ABABAAAAB-ABAAB-BBA-AA-B-BBB-AAB-AB--

*000380_00250057 B-BABBBAABBBBABBABAAABABBABBAABA-BABBABAABBABABAA-ABABABAA-ABBABAABBBBABAABBBBBBBBBBBABBB

*000380_00312800 B-BABBBAABBBBABBABAAABABBABBAAB--BABBABAAB-ABABAA-ABABABAA-ABBABAABBBBABAABBBBBBBBBB-ABBB

*000381_00506573 BB-AABBA--A-B-AABBB-BAA-A-AB-AA-BB-AAA-ABA-BAABBA-AABAAAAB--ABAAAB-BBB--AABBAABBBAABAAAAA

*000382_00095793 B-AB-BA-ABB-AAABBAA-BABAA-B-AAB-AA-BBBBA-ABAAA-AABBAA-A-AAAABAAA-ABAABBABAB-BA-ABB-BAA-AA

*000382_00319998 B--BABAAABBAAABBBA-ABABAAABAAABAAAABBBBA-A-AAAAAABBAAAABAAAABA-AA-BAABB-BABBBAB-BBBBA-B-A

*000382_00385661 BAAAABAAABBAAABBBA-ABABAAABAAABAAAABBBBAAABAAAAAABBAAAABAAAABAAAAABAABBABABBBABABBBBAABAA

*000383_00099313 BBAAABAABABB-BBAA-AA-AAAAAAAAAA-BBBBBBBABBBAAAABAABAABAAAABABBABB-A-AAABABAABAABBBBABABAA

*000383_00134404 BBAAABAABABB-BBAABAA-AAAAAAAAAA-B-BBBBBABBBAAAABAABAABAAAABABBABB-A-AAABABAABAABBBBABABAA

*000383_00398917 BB-AABAABABB-BBAA-AA-AAAAAAAAAA-BBBBBBBABB-AAAABAABAABAAAABABBABB-A-AAABABAABAABBBBABABAA

*000383_00423007 BB-AABAABABB-BBA--AA-AAAAAAAAA--B-BBBBBABB-AAAABAABAABAAAABABBABB-A-AAABABAABAABBBBABA-AA

*000385_00058327 A-BBBAAA-ABABABAABABBBBBBBAA-BABABABABBBBB-BAAAA-AAAAB-BBABBAABABBBBAAABBBBABAAB-BBAAABAA

*000385_00130944 AABBBAAA-ABABABAABABBBBBBBAA-BABABABABBBB-BB-AAAAAAAAB-BBABBAABABBBBAAABBB-ABAABBBBAAABAA

*000385_00339652 AABBBAAA-AB-BABAABABBBBBBBAA-BABABABBBBBBBBB--AA-AAAAB-BBABAAABABBBBAA-BBB-ABAABBBBAAABAA

*000386_00045115 BB-B-BBBAB-AAA-A-ABBAB-AABAAABABABABBBAAAB-AABB-BAAA-ABABABAAA-AABABB-AA-BA-AABAAAA-B-AAA

*000386_00052354 BB-B-BBBABAAAA-A-ABBAB-AABAAABABABABBBAAABBAA-B-BAAA--BABABAAA-AABABB-AA-BABAABAAAA-BBAAA

*000386_00168789 BB-B-BBBAB-AAA-A-ABBAB-AABAAABABABABBBAAAB-AABB-BAAA-ABABABAAA-AABABBAAA-BA-AABAAAA-B-AAA

*000387_00119115 BBB-BBAA-A-BBBABABB-AAAAAAABABABAA--BA--ABAAA--BBBABBAABBB-ABAAABBABAABBAAAABB-AB-BA-AABB

*000387_00353139 B-B-BBAAAA-BB-AB-BB-AAAAAAABABABAAABB-AAAB-AA---BBAB-AABBBA-BAAA-BABBA-BAAAA-B-A-BBAA-ABB

*000389_00053190 AA-AAABBABBBABABBABABAABBBBAABAAAA-AA-ABAAABA-BB-AAABAABAAAA-BBBB-AABBAABAAAAABAABBBBAAAA

*000389_00158909 A--A-ABBABBBABABBABABAABBBBAABAAAAAAA-ABAAABA-BB-AAABAABAAAA-BBBB-AABBAABAAAAABAABBBBAAAA

*000389_00279291 AABA-A-B-BBBABAB-AB-BAABBBBAABA-AA-A---BA--BA-BBAAAABAABAAA--BBBBAAABBAABAAAAABAAB-BBAAAA

*000390_00135461 B-BBBBAAABBAAAABBA-ABABAAABAAABAAA-BBBB-AA-AAB-B-BBAAABBAAAABAAAAAAAABBAA-BABABBABBBAABAA

*000391_00340891 AABBBAABAAABBBAABB-BBBBBAABAAABBABABBBABBBAAAAAABAAA---BBABAAABAB-BB-BAB-ABBABBBBAABBBA-A

*000391_00407598 AABBBAABAAABBBAABB-BBBBBAABAAABBABABBBABBBAAA-AABAAA-B-BBABAAABAB-BB-BAB-ABBABBBBAABBBAAA

*000392_00071022 B-B-BBAAAA-BBBA-AB--AA-AAAABABABAA-BAABAAB-AA--BABBBBAABBB-ABAAABBA-AABBBAAABB-AAB-AAAABB

*000392_00211830 BBB-BBAAAA-BBBABAB--AAAAAAABABABAAABAABAABAAAA-BABBBBAABBB-ABAAABBA-AABBBAAABBAAABBAAAABB

*000392_00246484 B-B--BAAAA-BBB-BABB-AA-A---B-BA-A--B-ABAAB--A--BABBBBAABB--ABAAABBA-AABBAAA--BAAABBAAAABB

*000392_00320801 B-B-B-AAAAABBBABAB-AAAA-AAABABAB-AAB--BAAB-AA--BABBB-AA-BBAABAAA-BA-AABB-A--BBAA-BBAA-ABB

*000392_00454146 BBB-BBAAAA-BBBABAB--AAAAAAABAB-BAAABAABAABAAA--BABB-B-ABBBAA-AAABBA-AABBAAAABBAABBBA-AABB

*000393_00251696 ABBABABBAABABABBBB-AABBBBAAABBBBAAABBBBAAA-BA-ABAAABBBAABBAABBABAABABBBAB-BABBBABAABBABBB

*000393_00293176 ABBBBABBAABABABBBBBAABBBBAAABBBBAAABBBBAAABBA-ABAAABBBAABBAABBABAABABBBAB-BABBBABAAB-ABBB

*000393_00364800 ABBBBABB-ABABABBBBBAABBBBAAABBB--AABBBBAA--BA-A-AAABB-AABBAA--ABAABABBBAB-BABBBABA-BBABBB

*000395_00304038 B-AAABABAABAAAAAAAA-BAABAABBBBABBBBBBABBAABABAABBA-BBAAAAABAAABB-BBAAAB-AAAAABAABAAAAAABB

*000395_00387276 --AA-BA-AABAAAAAAA--BAABA--ABBABBBB-BABBA-BAB-ABBA-BBAA-AABAAABBBBBA-AB-AAAAABAABAA-A-ABB

*000396_00210320 BABABBBBB-ABBBAABBBBAABBAABABAABBABAAA-BABAABAAAA-ABBBBABAAAA-AAAAAABAAABAABBBBABBBA-BA--

*000397_00029719 BABAB-BBBAABAABBB-ABBBBBABBABABAA--AAB-A-BAAA--BBAAAA-BBBAA-BAB-BBAAAABBB-BBAAAA-B-AABBAA

*000399_00002493 BABABBBAABBAAA-B-ABAABAABBBBBB--AA-BBBBBABAAA-AB-BABABAAABABAAABB-BBAAAABBABBBAABBB-AABBB

*000400_00232396 BA-A-B-BBABBAABABAABBAAABABBBBAAAAAAAABBBBAABBBAA-A-BBABBBB-ABBBABAA-AAB-AABAABBBBBABAAAA

*000400_00381823 B--AA---BA-BAABABAAB-AAABABB-BAAAAAAAABBBBAABB-AA-AAB-A-BBB-AB-BA-AA-AAB-AABAABBB-B-BAAAA

*000400_00381909 BA-A-B-B-ABBAABABAABBAAABABB-BAAAAA-AABBBBAAB-BAA-AABBABBBB-ABBBABAABAAB-AABAABBBBBABAAAA

*000400_00416946 BAA---BBBABBAABABAABBAAABABBB-A-AA-AAAB-BBAABBBAA-AABBABBB-AABB-ABAA-AA--AABAABBBBBA-AAAA

*000400_00438066 B----BBBBABBAABABAABBAAABABBBBAAAAAAAABBBBA-BBBAA-A-BBABBBB-ABBBABAAAAABBAABAABBBBBABAAA-

*000402_00264732 BB-A-BA--ABBAAB-BAABB-AABABBBBA-AAAAAABBBBAAB-BAA-AABBABBBB-ABBBABA--AAABAABAA-ABBBA-A-AA

*000403_00211122 BBABABAB-AABAB-AB-AB-AABBBBBBB-BBBBAAAAB-B-BBAABABBAAABAABABAABBABB-BAABAABBBAABBBBAABAAA

*000403_00239589 BBABABABAAABAB-ABAABBAABBBBBBBB-BBBAAAABABBBBAABABBAAABAABABAABBABB-BAABAABBBAABBBBAABAAA

*000403_00382265 BBABABABAAABAB-ABAABBAA-BBBBBBB-B-BAAAABAB-BBAAB-BBAAAAAA-ABAABBA-B-BA-BAABBBAABBBBA-BAAA

*000404_00150963 BABBBBAABAA--BAAA--BA-BBA-BAAAABBABBBABAABA-BAAAA-AA-BAABAAAA-AAAABABAABB-ABBBBBABBABBAAA

*000405_00374498 AAABAABBA-ABBABAAB-BAAABAABABB--BBBBBB-AA-BBBBABAAAAB-BAABABBAAB-BBAB-BBBABABAABAAABBBBAA

*000405_00562557 ---B--BBA-ABB-BA-BBBA--BA-BABBB-BBBBBB-AA-BBB-AB-AAAB-B-ABABBAAB-BBAB-BBBABABAABAAAB-BBAA

*000408_00066711 BABABBBAAB-AAAABBAAA-AAABBAABA-A-BBB-AABABABABA-ABABAA-BBAABBA-ABA-BBBB-BBBAA-B-ABBBBBA-B

*000408_00167595 B-BABBBAAB-AAAAB-AAA-AAAB-AAAA-A-BB--AAB-B-BABAAABABAA--BAABBA-ABA-BB-BBB-BB--B--B-B-BAB-

*000410_00103195 B-AA-BAAAABAAA--AAA-B---B-BAAABBA-A-B-BA-BBB--BAABA-B-A-A-AABABA--ABABABABB-BBABAAABBABBB

*000410_00181187 BAAAABAAAABAAABBAAAABBABBBBAAABBABABB-BAABBBB-BAABABBAAAABAABABA-AABABABABBAB-ABAAABBABBB

*000410_00290037 BAAAABAAAABAAABBAAAABBABBBBAAABBAB-BB-BAABBBBBBAABABBAAAABAABABA-AABABABABBAB-BB-AABBABBB

*000410_00314527 BAAAABAAAABAAABBAAAABBABBBBAAABBABABB--AABBBBBBA-BABBAAAABAABABA-AABABABABBAB-BBAAABBABBB

*000411_00319849 ABAAAAAB-A-AAABBAA-A-BBBBABAABBBBBAAABA-AB-BB-B-ABABA-BAA-AAAA-BAAA-ABBBBABABBBBAA---AABB

*000413_00463923 A-BABAABAAAAAAAABAABBBA-BABABBBBBBBBABBBABBAAAABBA-BBAAAAAB-AABABABAAAB-AABAAABBBAAAAB-BB

*000413_00539874 A-BABAABAAAAAAAABAABBBA-BABABBBBBBBBABB-ABBAAAABBA-BBAAAAABAAABABABAAAB-AABAAABBBAAAAB-BB

*000414_00167322 AABBBA-B-AB---BA-B-BB-ABBBAAB-B-ABABB-BB-BABBAAABA-ABBA--A-AA-BABBBBAA-B-AB-BAAB-AAA-ABA-

*000414_00245486 AABBBAAB-AB-BABAABA-BBA-BBAAB-BBABABBBBBBBBBBAAA-AAABBABBABAAABABBBBAABBBABABAABBAAAAABAA

*000415_00141189 BBAAABABBBABA-BABABABAABABABBAAABBA--ABBBABBA-BBAAAABBABAABAABAAAABBBBABAABBAABBBAAAAAAAA

*000416_00043643 A-BABAABAAAAAAAABAABBAA-BABABBBBBB-BABBB-BBAAAABBA-BBAAA-ABAAABBBABAAAB-AABAAABBBAAAAB-BB

*000416_00221232 A-BABAABAABAAAAABAABBAA-BABABBBBBBBBAABBABBAAAABBA-BBAAAAABAAABB-BBAAAB-AABAABBBBAAAAB-BB

*000417_00012649 ABAAAAABBABABABBABAAAABAAABABABBBBBBBB-BBB-ABBAAABABBBAAAABAABA-BAAABBABBABBBAABABBBBABBB

*000417_00171033 ABAAAAABBABABABBABAAAABAAABABABBBBBBBBABBBBAABAAABABBBAAAABAABA-BAAABBABBABBBAABABBBBABBB

*000418_00096211 A-BAB-B--BBAA----ABAABBABB--BABAAAA-BBAB-A-AABAB-AABABAAA-A-A-ABB-BBA-AABBAB-B-ABB----BBB

*000419_00085353 B-BABBAA-BBAAAB--ABAB-ABAABBAABAAAA-BABAABBAAAAAA-BBAAA-AABABABBA-ABABBABAB-BA-A-BBB---A-

*000419_00150085 BABABBAAABBAAABBAABAB-ABAABBAABAAAABBABA-BBAA-AAAABBAAABAABABABBAAABABBABABBBABABBBBAABA-

*000419_00168638 BABABBAAABBAAABBAABAB-ABAABBAABAAAABBABA-B-AAAAAAABBAAA-AABABABBAAABABBABABBBABABBBBAABA-

*000419_00386483 BABABBAAABBAAAB-AABAB-ABAABBAABAAAABBABAAB-AAAAAAABBAAAAAABABABBAAABABBABABBBABABBBBAABA-

*000420_00496509 B-BABBBBBAAB-ABBBAABBBB--BBAB-B-AA-AA-AABB-A--A-BAAA-ABBBAA-BABABBA-AA--BABBAAAAB--A-BBA-

*000421_00339806 BABBBBAAAAAB-AAAB--BA-BB-BBAAAABBABBBABAABA-AAAAABAA-BAABAAAABAAAABABAABBAABBBBBABBA-BAAA

*000422_00154136 BBAAABBBBBABA-AABABBBA-BA-ABBAAA-BAB-AABBA-BAABBAAAABAAA--B-ABAAABBBBBAAAABBAABA-AA-AAAAA

*000422_00225038 B-AAABBBBBABA-AABABBBA-BA-ABBAAA-BA--A-BBABBAABBAAAABAAA-BB-ABAAABBBBBA-AABBAABABAA-AAAAA

*000422_00232272 BBAAABBBBBABA-AABABBBA-BA-ABBAAA-BAA-A-B-A-BAABBAAAABAAA-BB-ABAAABBBBBA-AABBAABABAA-AAAAA

*000423_00077725 ABBBBABBAB-BAABAAABAABAAABAAA-BBAB-AABABBBAA--BBAAABAABABABAAAABAB-BAAABBBBBB-B-ABBAAAB--

*000423_00422158 ABBBBABBAB-BAABAAABAAAA-ABAAA-BBAB-BABABBBAA-BBBAAABAABABABAAAABAB-BABABBBBBB-B-ABBAAAB--

*000424_00153786 BBBABBBBAA-A-BBAA-ABBBBBBBBABBAABBBAAABBBBBABABBBAAAAABABAABAAB-A-B-BAA-AABBABBBAAABBABAA

*000424_00173684 BBBABBBBAA-A-BBAA-ABBBBBBBBABBAABBBAAABBBBBAB-BBBAAAAABABAAB-AB-A-B-BA--AABBABBBAA-B-ABAA

*000424_00421265 BBBABBBBAA-A-BBAA-ABBBBBBBBABBAABBBAAABBBBBABABBBAAAAABABAABAAB-A-B-BAA-AABBABBBAAABBABAA

*000424_00421866 BBBABBBBAA-A-BBAA-AB-BBBBBBABBAABBBAAABBBBBAB-BBBAAAAABABAABAAB-ABB-BAA-AABBABBBAAABBABAA

*000424_00421952 BBBABBBBAA-A-BBAA-ABBBBBBBBABBAABBBAAA-BBBBABABBBAAAAABABAABAAB-A-B-BAA-AABBABBBAAABBABAA

*000424_00422442 BBBABBBBAA-A-BBAA-ABBBBBBBBABBAABBBAAABBB-BABABBBAAAAABABAABAAB-A-B-BAA-AABBABBBAAABBABAA

*000424_00491804 BBBABBBBAA-ABBBAABAB-BBBBBBABBA-BB-AAABBBB-ABABBB-AAAABABAABAAB-A-B-BAAAAABBABBBAAABB-BAA

*000425_00188497 B-ABABAB-ABBBBBBBBBBBBAAAABABBBABBBBBABBBBABABBAABAB-AABAAAB-ABAABBBAAAABAB-ABBAAAAABBBBB

*000425_00221266 B-ABABAB-ABBBBBBBBBBBBAAAABAABBABBBBB-BBBBABABBAABA--AAB-AABAABAABBBAAAABABBAB-A-AAABBBBB

*000426_00009948 BAAA-BAA-BBAAA--BAABBA--ABBAAA--ABAAAA-AAA-BABBBBAABAABABAABABBBBAABA-AAABBAAB---AABB-BBB

*000426_00014829 BAAAABAABBBAAABABAABBAB-ABBAAAA-ABAAAA-AAAABABBBBAABAABABAABABBBBAABA-AAABBAABBAAAAB-ABBB

*000426_00055651 B-AAABAABBBAAAB-BAABBAB-ABBAAAA-ABA-AA--AAABABBBBAA-AABABAABABBBBAABA-AAABBAABBAAAABBAB-B

*000426_00175897 B-A--BAABB-AAAB--AAB--B--B-AAAABABA-A--A-A-BABBB-AABAA-AB-ABA-BBBAABA--AABBAABB-AAABBABBB

*000426_00227231 AAAAAAAABBBAAABBBAABB-B-ABBAAAAA-BAAAA-AAAABAB-BBAABAABABAABABBBBAABA-AAABBAABBAAAABBABBB

*000426_00229413 A-AAAAAAB-BAAAB-BAABBAB-ABBAA-A--B-AAA-AAA-BABBBBAABAABABAABABBBBAABA-AAABBAAB-AAA-B-ABB-

*000427_00457337 B-ABAB-BBABBAABABABBBAAABBBBBBA--BAAAAABBBAABABBABAAABABBBBBAB-BBBAA-AABAAABAABBBAA-BAAAA

*000427_00457423 BAABABBBBABBAABABABBBAAABBBBBBA-AB-AAAAB-BAAB-B-ABAAABABBBBBABBBBBAAAAABAAABAA-B-AA-B--AA

*000427_00462000 BAABAB-BBABBAABABABBBAAABBBBBBAAABAAAAABBBAABABBABAAABABBBBBABBBBBAA-AABAAABAABBBAAABAAAA

*000428_00320334 BBBBBBAABAAABBABBBABABB-B-BB-AAAAAABBBABABBA-BAAABBBBBAB-BBAABABBBABBAAAAA-AAAAAABBBB--BB

*000428_00428757 BBBBBBAABAAABBABBBABABB-B-BBBAAAAAABBBABABBA-BAAABBBBBAB-BBAABABBBABBAAAAA-AAAAAABBBBABBB

*000428_00428843 -BBBB-A--A-AB--B--ABA---BABBBAA--AA-B--BAB-A-BA-ABBBBBABABBAABABBBABBAAAAAAAAAAAAB-BBA-BB

*000429_00007872 AABABAABAAAABBAABB-BBBBB--BAAABBABABBBABBBAAAAAABAAA-BBBBABAAABABBBBABAB-ABBABB-BAABBAABA

*000429_00065852 AABABAABAA-ABBAABB-BBBBBB-BAAA-BABABBBABBB-AAAAABAAA-BBBBABAAABABBBBABAB-ABBABB-BAABBAABA

*000429_00237506 AABABAABAAAABBAABB-BBBBBB-BAABBBABABBBAB---AAAAABAAA-BBBBABAAABABBBBABAB-ABBABB-BAABBAABA

*000429_00240589 AABABAABAAAABBAABB-BBBBB--BAABB-AB--BBABBB-AAAAABAAA-BBBBABAAABABBBBABABAABBABB-BAAB-A-BA

*000432_00068070 BBA-ABBBABABBBABBBBB-BAABBB-AAAABBBBBAAABBBBA-BBBABAAABBBBBBBAABAAABAAABB-ABABBBABBABBBAA

*000432_00134777 BBBABBAABABABAABABAAAABABAB-BAAAABABBBA-ABBA-BAAABBBABABABBABBA-BBAABAAABA-AAA-AABBBBABBB

*000433_00607326 ABABAABBA-ABBAAAABAAA-ABAABABB--BB--BBAAAB-ABBABAA-ABAAAABABBAABBBBBB-BBBABAAA-BABBABBBAA

*000434_00275339 ABBABAB-ABBBBBABABBABAA-ABBAABBABABAABAB-BABBAABBABAABABA-BBBBBA-BAAABBABA-A--BAAAABB-BAA

*000434_00312013 ABBAB-B-ABB-BBABABBABAA-ABBAABBABABAABAB-B--BAABBABAABAB--BBBBBA-BAAABBABA-A--BAAAABBABAA

*000437_00052544 ABA-AAABAA-AAABAA-AAB--AAA-B-AABBAB-AAA--ABBABABBAB------ABABBBBABBBAA-BAAA--AAA-AABBA-A-

*000437_00058406 ABA-AAAB--BAAABAAAAABA-AAABBBAABBABAAAAAAABBABABBA-AAAAABABABBBBABBBAABBAAABBAAAAAABBAAAA

*000439_00117145 BBBABBAAABABAABBBAABAAABBABABABBABBBBBBBBAABABAAABA-ABABBAAAAAAAAAAAAABAABBABBAABAAAAAA-A

*000440_00237428 BABBBBAAAAAB-BAAB-BBA-BB-BBAAAABBA-ABABAABAAAA-AABAA-BAABAAAABAAAABABAABBAABBBBBABBA-BAAA

*000441_00295125 ABAAAABABBBA-BBBA-AAAAAAAB-AAABAAAA-AB-BBAAABBBAABAAABBAB-BABBAAAABBABBAAABBBABABBB-AB-AA

*000441_00429579 ABAAAABABBBA-BBBA-AAAAAAAB-AAABAAAA-AB-B-A-ABBBA-BAAA-BBB-BABBAAAABBABBAAABBBABABBB-AB-AA

*000442_00298652 B--BBBBAABBBBBBBBBBBABBBABAABABBBBBAABABAAAAABA-BABABABBBBBBBAABABBABAABAABABAABBBBAAAAAA

*000445_00043094 A-BABABBAAAAABAAAAAB-BBBBBBABBBBBBBAAABBBBBABAABBAABBBBBBABBAABABABABAB-AABAABBBAAAAABBBB

*000445_00179003 --BABABBAAAAABAAAAABBBB-BABABB--B-B--AB-BB--B-ABBAABBBB-BABAAABABABABAB-A-BAAB-BAAAA-BB-B

*000446_00141272 AAAAAABAAB-ABA-BABABAAABABBABBBAB-BBBBBAAAABAAB-ABBBABBBBAAABAAAB-ABABABBBBABBBBBAABABABB

*000446_00334930 AAAAAABAABBABA-BABABAAABABBABBBBBBBBBBAAAAABA-B-ABBBABBBBAAABAAAB-ABABABBBBABBBBBAA-ABA-B

*000447_00159322 BABBBB-BBABBAABABABBBAAABBBBBBAAAAAAAAABBBAABBBAABAAABABBBBBABBBBBAA-AAB-AABAABBBAAABAAAA

*000448_00092579 A-AAAAABAABABABBABAAAABAAABABABBBBBBBBABBBBA--AAABABBBAAAABAABA-BAAABBABBABBBAAB-BBBBABBB

*000448_00112127 A-AAAAABAABABABB-BAAAA-AAABABABBBBB-BBABBBBAABAAABABBBAAAABAABA-BAA-BBA-BABB-AABABBBBABBB

*000448_00215288 ABAAAAABAABABABBABAAAABAAABAAABBB-BBBBABBB-AABAAA-ABBBA-AABAABABBAAABBABBABBBBABABBBBABBB

*000449_00101874 BABABBBBBBBAABBAAABABAB--ABBABBBAAA-ABAABBBAA--BBBBABABABABBAAAAAABBBABBABBAAB-BBAAAABAAA

*000449_00170332 BABABBBBBBBAABBAAABABAB--ABBABBBAAAAABAABBBAAB--BBBABABA-ABBAAAAAABBBA-BBBBAAB-BBAAAABAAA

*000452_00008526 AA-A-A-BABBBABABBABABAABBBAAABAAAAAAA-ABAAABABBB-AAABAABAAAA-BBBBAAABBAABAAAAABAABBBBAAAA

*000452_00328759 AA-A-A-BBBBBABAB-ABABAABBBAAABB-AAAAA-AB-AABA-B--AAABAABAAAA-BBABAAABBAABA-AAABAAAABBABAA

*000453_00241472 BAABABABBBAABABBBB-BAAAABA-BA-AA-B-AABBB-ABB--ABBAABBBABBAABAAABAABBABAABBAB-ABAAAAAABABB

*000453_00283118 BAABABABBBAABA-BBBA-AAAABA-BABAAA-BA-BB--A-B-AAB-AABBBABB-ABAAABA-BBABAAB-A--ABAA-AA---BB

*000453_00304987 B--B-BABBBAABABBBB-BAAAABAABA-AABB-AABB-BABB--ABBAABBBA-BAABAAA--ABB-BAA-BAB-A-A-AAAAB-BB

*000453_00354114 BAABABABBBA-BABBBB-BAAAABA-BA-AABB-AABBB-A-B-AABBAABBBABBAABAAABAABB--AABBAB-ABAAAAAAB-BB

*000454_00047540 AAABAAABA-BBB-ABABBABBABABBBABABBABBB-BBAB--BB-AA--ABAABABBAABBAB-ABBA-ABBAAAAAABAAABAAAA

*000454_00164524 AAABAAABA-BB-BABABBABBABAB-BA-ABBA-BBBBB-BB-BB-A---ABAABABBAABBABBABBA-AB-AAAAAABAAA-AAAA

*000454_00261787 AAA-AAABA-BBB-ABABBABBABABBB-BABBABBBBBBABB-BB-AA--ABAABABBAABBAB--BBA-ABBAAAAAABAAABAAAA

*000455_00006569 BBAA-BBBAAAABAABBBAAAABAAABBABBBABAA-BABABBBAABAAAAABBAABABA-AABBAAB-BBABBBBA-ABBAAABABAA

*000455_00317051 B-AAA-B-AAAABAABBBAAAABA-ABBAB--AB-A-BABA--BA-B-A-AA-BAABAB-B-ABBAABBBBABBB-A---BA--B-B-A

*000456_00132644 BBBABBAABAAABB-BBBBBBABB-ABBAABBBBBABABBBBAA--BABBAA-ABBBBBABABBBAABABABAABBAABBBBBB-BAAA

*000456_00286836 B-AAABAABAAABB-ABBBBBABB-ABBAABBBB-ABABBBB-ABABABBAAAABB-BBABA--BAABAB-BAAB-AABBBBBBBBAAA

*000456_00287476 BBAAABAABAAABB-ABBBBBABB-ABBAABBBBBABABBB--A-A-ABBA--ABBBBBABAB--AABABABAABBAA-BBBBB-BAAA

*000456_00292796 BBAAABAABAAABB-ABB-BB-BB-ABBAABBBBBABABB-B-A--BABBAA-ABBBBBABAB-BAABA-ABAABBAABBBBBBB-AAA

*000459_00403854 BABBBBBBBAAB-BAAB-BBA-BB--BABAABBABAAAABABA-BAAAA-AABBAABAAAA-AAAAAABAABB-ABBBB-BBBA-BAAA

*000459_00437137 B-BBBBBBBAAB-BAAB-BBA-BB--B-BAABBA-AAAABABA-BAAAAAAABBAABA-AA-AA-AAABA-BB-ABBBB-BBB-BBAAA

*000459_00438603 B-BB-BBBBAAB-BAABBBBABBBAABABAA-BABAAAABABA-BAAAA-AAB-AABAA-AAAAAAAABAABB-ABBBB-BBBA-BAAA

*000460_00030272 A-BABABBAAAAABAAAAABBBB-BA-ABBBBB-BAAABBBBBABAABBAA-BBBBBABAAABABABABAB-AABAABBBA-AAAB-BB

*000461_00018171 B-BBBBABAABBBBABABBABAAAAAA-BBBABBBBBABAAAAAA--BBBAAAAAB-A-ABBBAAAABBABBAABABBAABBBBAAA--

*000461_00018257 BA-BBBABAABBBBABABBA-AAAAAA-BBBABBBB-AB-AA-AAB-BBBAAA-A-AA-ABBBAAAABBABBAABABBAABBBBAAA--

*000461_00161312 B-BBBBA-AABBBB-BABBAB-AAAAAABBBABA-BBA-AAB-AAB-BBBAAAAAB-A-ABB-ABAAB-ABBAABABBAABB-B-AAAB

*000461_00189249 BAB-BBABAABBBBABABBABAAAAAA-BBBABAB-BABAABAAAB-BBBAAAAAB-A-A-BBABAABBA--AABA-BAAB---AAA--

*000461_00312987 B--BBBABAABB--ABA-BABAAAAAAAB-B-BA-B-A-AA-AAABA-B-AAAAAB-A-ABBBA-A--BAB-AA-AB-A-BBBBA-A--

*000462_00081123 B-A-ABA-BBABA-BBBABAABABAAABBAAAAAA--ABBBB-BAABBABBAABABAABAA-AAAABBAAABAA-BBAABBAAAAABAA

*000463_00263592 B-AAABBAABBBBABBABAAABABBABBAABA-BABBABAABBBBBBAA-ABAAABBAAABBABAAAABBABAABBBBBBBAABB-BBB

*000464_00201492 ABAAAABBBAAABBA-BBBBBBBBB-BBBAAA-A-BBBABABBA-BAABBBBBBABBBBA-BABBBBBBBB-BABAAABA-BBA---BB

*000464_00402361 A-AAAA--BAAABBA-BBBBBBB-B-BBBAAA-A-BBBAB-B-A-BAABBBBBBAB--BAABABBBBBBBBABA-AAABAABBAAA-BB

*000464_00433724 A-AAAA--BAAABBA-BBBBBBBBB-BBBAAA-A-BBBABABBABBAABBBBBBAB-BBAABABBBBBBBBABA-AAABAABBAAA-BB

*000465_00232420 -AABA-AAABAA-ABAA-BBAAABABBAABABAAABBBBAABBBA-BABAABA-BAAAAABA-AABABBBAABBA-BBAABBBBB-BBB

*000465_00241305 --ABA-AAABAA-ABAA-BBAAABABBAABABAAABBBBAABBB-BBABAABA-BAAAAABA-AABABBBAA-BAABBAABBBBB-BBB

*000465_00330109 -AABA-AAABAA-ABAABBBAA-BABBAABABAAABBBBAAB-BABBABAAB-BBAAAAABA-AABA-BBAABBAABBAABBBBB-BBB

*000466_00041961 -B-BABBBABA-A--BBABBAA-ABABAAB--AB--A--BABBBAAA-A--BABBBBBBAABA-ABAAABBBBABAA-AB-A-A---BB

*000466_00260658 BBABABBBABABABABB-B-AAAA-ABAAB-B-BA-A-A--B-BAAABA-BBABBB-BBAABABABAAAB-BBA-AAB-BA-AABBB-B

*000467_00235341 AAABAA-BA-AB-BAAA-BABAAABAAAAABBAA-BBABAB-A-BBBB-ABABBABABBAABBABBAB-B-B-BBABBBBBAAAAAAAA

*000468_00067655 AABBBAAAABBAABBABAA-BBABABABABAAABA-AABBAABBA-BBBA-ABAABABBBABBBABBAAABBBBAAABABBAAABBAAA

*000468_00106482 A-BBBAAAABBAABBABAAABBABABABA-A-ABA-AABBAA-BA-B-BAAAB-ABABBBAB-BABBAAA-BB-AAAB-BBAAABBAAA

*000468_00187934 AABBBAAAABBAABBABAA-BBABABABABAAABA-AAB-AABBA-BBBAAABAABABBBABBBABBAAABBBBAAABABBAAABBAAA

*000468_00268441 AABBBAAAABBAABBABAA-BBABABABABAAABA-AABBAABBA-BBBAAABAABABBBABBBABBAAABBBBAAABABBAAABBAAA

*000468_00302654 A-BBBAAAABBAABBABAA-BBABABABABAAABA-AABBAABBAABBBAAABAABABBBABBBABBAAABBBBAAAB-BBAAABBAAA

*000470_00198234 BBBA-BBBAABABBBB-B-ABBBABABABBBABBBBBAAABBAB-BBABAABBABAABBBBABBBA-ABABBBABAAABBABB-ABABB

*000470_00198465 B--AABBBAABABBBBAB-ABBBABABABBBABBB-BA-ABBABBBBABAABBA-AABBBBAB-BAAABABBBABAAA-AAB-A-BA-B

*000471_00431704 BA---BABABABBAABB-BB-AAA-BBABBA--B-AABABAB-AAAAAAA-BABBA-BBAABBBBBA-BB-BA-BAABA---AB-A-BB

*000471_00463347 BABB-BABABABBAABBBBBBAAABBBABBAABB-AABABABBAA-AAA--BABBABBBAABBBBBAABBBB--BAABAB-AABAAABB

*000471_00474616 B-A--BABABABBAABBBBB-AAABBBABB-ABB--ABA-AB-AAAA-AA-BAB-ABB--A-B-BBAA-B-B--BAA-AB--ABA-AB-

*000472_00100456 -BAA-BAA-A-B-BB-BBABB-AA---A-BAB-BAAAB-ABB-AA-AB-AAAABAAA--A-AA-BAA-AB-BAAAABA-BBBB-B-BAA

*000472_00134202 BBAAABAA-AABB-BBBBABBBAAAAAABBAAABA-ABB--BBAAAABAAAAABAAAAAA-AA-B-A-ABABAAAA-AA--B-A-A-A-

*000472_00169225 BBAAABAABAABBBBBBBABBBAAAAAABBA-AB-AABBABBBAAAAB-A-AABAAAAAABAABB-ABABABAAAABAABBBBABABAA

*000473_00165683 ABAAAAABAABABABBABAAAABAAABAAABBBB-BBBABBBBAABAAA-ABBBAAAABAABABBAAABAABBABBB-ABABBBBABBB

*000473_00181725 ABAAAAABAABABABBABAAAABAAABAAA-BBBB-BBABBBBAABAAA-ABBBAAAABAABABBAAABAABBABBB-ABABBBBABBB

*000473_00235941 ABAAAA-B-A--BABB-B-A---AAA-A-AB-BBBB--ABBB-A-BA--AA-BBAA-ABAAB-BBAA-BAAB-ABBBAABA-BBBA-BB

*000475_00160706 B-BABBBAAB-AAAAB-AAB-AAAB-AABA-A-BB--AABABABABAAA-ABAABBBAABBABABABBBB--BBBA-BB-AAABBBABB

*000475_00312784 BABABBBAAB-AAAAB-AAB-AAAB-AABA-A-BBB-AABABABABAAABABAA-BBABB-A-ABABBBBBBBBBA--BBAAABBBABB

*000476_00012147 BABABBBBBBBA-AB-B-AAABBABAAAABBBBABAAB-BABBAAABBAA-ABABAAAAABAABAAA--AABBBBAA-ABBBBBBB-AA

*000476_00153849 BABABBBBBBBA-AB-B-AAABBBBAAAABBBBABAABBBAB-AAABBAA-ABABAAAAABAABAAA-BAABBBBAA--B-BBBBB-AA

*000477_00254828 AABBBAAA-ABABBBAABABBABBBBAA-BABABABAABABBBB-AAA-BAA-B-BBABBAAAABBBBAAABBBAAB-ABBBBAAABAA

*000477_00283947 A-BBBAAA-ABABBBAABABBABBBBAA-BABAB-BAABABBBB-AAA-BAA-B-BBABBAAAABBBBAAABBBAAB-ABBBBAAABAA

*000478_00075945 B-ABABABAABBBBBBBBBABBAAAABAABBABBBBBABBBBABA-BBABAB--A-AAAB-ABAABBBAAAABAB-ABBAAA-A--BB-

*000478_00133516 B-A-ABABA-BBBBBBBBBABBAAA-BAAB--BBBBBAB-BBABA-BBABA--AABAAAB--BAA-BBAAAABAB--BBAAAAABBBBB

*000478_00421547 BB-BABAB-AB-BBBBBBB--BAAAABAABBABB-BBABB-B-B-B-BABAB-AA-AAAB-A-AA-BB-AAABAB-ABBAAAAA-BBBB

*000479_00080342 BA-B-BBA-BAAAABAB-A--AAAB-ABAAAAA-A-AB-BBABBBBA-B---B-ABBABBA-ABAAAAAA---AABBAAA-AAAA--BB

*000479_00080428 BAABABBA-BAAAABABAABAAA-B--BAAA--BAAABBBBA-BB---BABBBBABB-B-AAABAAAA-A-ABAABBAAAAAA-AAB-B

*000479_00086877 BAABABBABBAAAABABAABAAAABB-BAAAA-B-AABBBBABBB-ABBABBBBABB-B-AAABAAAAAAAABAABBAAAAAAAAABBB

*000479_00088834 BAAB--BA-BA-AA-A-A-BA-A-BBB-AA--A---ABBB-A-BBB-BBABB--ABBAB--AA-AAAAAAAABAAB-AAAAAAAAA-BB

*000479_00298143 B-ABAB--BBAAAABABAABAAAABB-BAAAA-BAAABBBBABBBBABBABBBBAB--B-AAABAAAAAAA-BAABBAAAAAAAAAABB

*000479_00312618 B--BA-BAB--AAABABAABA-A--BABAAAAB-BA-BB--A-B-B-B-ABB-BABBBB-AAABAAA-AA-ABAABBA-AAAAAAAABB

*000480_00007762 AAAAAABB-AABABBAAAABBBBBBA--BAAABBBAAABB-AABBBBAAABA-BBABABAAABAA-ABBBAAAAABABB-BBBBABAAA

*000480_00060299 AAAAAABB-AABABBAAAABBBBBBA-BBAAABABAAABB-AABBBBAAABABBBABABAAABAABABBBAA-AABABB-BBBBABAAA

*000480_00084386 AAAAAABB-AABABBAAAABBB-BBA-BBAAABABAAABBBA-BBBB-AABA-BBABABAAABAAAABBBAA-AABABB-BBBB-B-AA

*000480_00144844 A-AAAABB-AABABBAAAABB-BBBAABBAAABABAAABB-AABBBBAAABABBB-BABAAABAA-ABBBAA-AABABB-BB-BABAAA

*000480_00258748 AAAAAABB-AABABBAAAABBBBBB--BBAAABABAAABB-AAAB-BAAABABBBABABAAABAA-ABBBAA-AABABB-BBBBABAAA

*000480_00300205 AAAAAABB-AABABBAAAABBBBBB--BBAAABABAAABBBAAABBBAAABABBBABABAAABAA-ABBBAA-AABABB-BBBBABAAA

*000481_00112914 ABBABAAABAAAABBBBA-AB-AAA-BBBABAAAAAAAABBBABBBABBBBAAABBAAAB-ABBAB-BABBABBABB-BAABBABABAA

*000481_00113000 A-BABAAABAAAABBBBA-AB-AAA-BBBABAAAAAAAABBBABB-ABBBBAAABBAAAB-ABBAB-BABBABBABB-BAABBABABAA

*000481_00183894 ABBA-A-ABAA-ABBBBABA-AAAA-BBBABA-AAAAAAB-BABBBABB--AA-BB-AAB---B-B-BABBAB--B--BAABBABA-A-

*000481_00375960 ABBABAAABAAAABBBBA-AB-AAA-BBBABAAAAAAAABBBABBBABBBBAAABBAAAB-ABBAB-BABBABBABB-BAABBABABAA

*000483_00144617 A-AAAA-BAABABBAA-BB-AA-ABB-AAA-A--BAAAAAA-A--BBB-BBAB-AABAAABBBBBABAAAAAAA-AAAAAABBAABBAA

*000484_00037692 ABBABAAAB-BABBBBABAA-BAABAABBBA-BABABBBA-AABBBBBAAA--ABBBABA-BAABABA-BBAABAABABABAA-ABBAA

*000484_00067764 ABBABAAABBBABBBBABAABBAABAABBBABBA-ABBB-BAABBB-BAAA--ABBBAB--BAAB--A-BBAABA-B-BABA---BBAA

*000484_00366185 ABBABAAAB-BABBBBABAA-BAABAABBBA-BABABBBAAAABBBBBAAA--ABBBABA-BAABABAABAAABA-BABABAA-ABBAA

*000485_00057798 B-BABBBBBBAAABABAAABBBABBABABB-AABABBBABAB-ABBAABABA-BBABBBAAABB-ABB-BABAAB-ABBAAAAB-AAAA

*000486_00031836 A-BABAABAAAAAAAABAABBBA-BA-ABBBBBB--ABBBABBAAAABBA-BBAABAA-AAABA-ABAAABAAABAAABBBAAAA--BB

*000486_00060605 A--ABAABAAAAAAAABAABBBA-BABABBBBBBBB-BBBAB-AAAA-B--BBAABAABAAABABABAAAB-AABAAABBBAAAABBB-

*000486_00166398 A-BABAABAAAAAAAABAABBBA-BA-ABBBBBBBBABBB-BBAAAABBA-BBAABAABAAABABABAAAB-AABAAA-BBA-AAB-BB

*000486_00401426 A-BABAABAAAAAAAABAABBBA-B--ABB-BBB--ABB-AB--A-ABBA--BAAB---AAABAB--A-AB-AABAAA-B-AAAABA--

*000490_00091109 AABABABABBBBABBBAAAABBB-AABAABBBBABAABABBBABAA-BBBAA-BABAABBAABAAABBBBAABBBAA-BA-AAABBBAA

*000490_00248520 -AB-B-BABBBBA-BBAAAABBB-AA-AABB--ABAABABBB-BAA--BBA-BBABBABBAABAAABBBBAABBBAA-BAAA-ABBBAA

*000490_00268271 A-BABABABBBBABBBAAAABBB-AABAABBBBABAABA-B-ABA-ABBBA--BABBABBAABAAA-BBBAABBBAA-BA-AAABBBAA

*000492_00112357 B-B--B-AAB-BBABB-BAAABA-BAABAA-A--BBBAB-ABAAAABABAABA-A--A-AABABBA--B-ABAAA-BABB-BB-B--BB

*000492_00231727 B-BABBAAABBBBABBABAAABA-BAABAAAABBBBBABA-BAA-ABABAABABABAA-AABABBAABBBABAAABBABBABBBBABBB

*000493_00056647 BABABBBAAB-AAAABBAAAAAAAB-AA-ABA-BBB-BABABABA-A-ABAAAA-BBAABBA-ABA-BBBB-BBBB----ABBBBBAAA

*000493_00135817 BABABBBAAB-AAAABBAAAAAAAB-AAAABA-B-B-BABABABABB-ABAAAA-BBAABBA-ABA-BBBBBBBBB--B-ABBBBBAAA

*000493_00210950 BABABBBAAB-AAAABBAAAAAAAB-AAAABA-BBB-BABABABAB--ABAAAA-BBAABBA-ABA-BBBB-BBBB--B-ABBBBBAAA

*000493_00216990 BABABBBAAB-AAAABBAAAAA-AB--AAABA-B-B-BABABABA---ABAAAA-BBAABBA-ABABBBBB-BBBBB---ABBB-BAAA

*000493_00232859 BABABBBAAB-AAAABBAAAAAAAB-AAAABABBBB-BABABABAB--ABAAAA-BBAABBAAABA-BBBB-BBBB--B-ABBBBBAAA

*000494_00075376 BABABBBBBBBBABBAAABA-AB--ABBABBBAA-AABAABBBBAB-BBBBABABABABBAAAA-ABBBAA-BBBAAB-BBAAAABBAA

*000494_00210943 BABABBBBBBBBABBAAABABAB--ABBABBBAAAAABAABBBBA--BBBBABABABABBA-AAAABBBAABBBBAAB-BBAAAABBAA

*000494_00221255 BABABBBB-BBBABBAAABA-AB--ABBABBBAAAAABAABBBBAB-BBBBABABABABBAAAAAABBBAABBBBAAB-BBAAA-BBAA

*000495_00159570 BBBA-BAAABABAABBBAABAAABBA-ABABBABB-B-BBBA-BABA-ABABABABBAAAAAAAAAAAA-BAAA-ABBAAB-AAAAAAA

*000495_00351338 BB--BBAAABABAABBBAABA--BB--ABABBABBBBBBBBA-BA-ABABA-ABABBAAA-AAAAAAAAABA-ABABB--BAAA-AAAA

*000496_00394601 BBABABBAABBBBBABBBBABAABB-AAABBBBBBAABBB-BABB-AABB-ABBABBABBBBBAABBABBB-AABABBBBAAABB--AA

*000497_00338343 BBBBBBABABABBAABBBBBBAAABBAABB-AABABBBABAB-AA-AA-A-BA-BBABAAABBBBBBABABBBBBABBABA--BAABBB

*000497_00338429 BBB-BBA-ABABBAABBBB-BAAABBAABBAA-B-BBBABAB-AAB---AAB-BBBABAA--BBBBBABABB-B-ABBABA--BA-BBB

*000497_00445704 B-BBBBABABABBAABBBBBBAAABBAABBA-ABABBBABAB-AA-AA-A-BA-BBABAAABBBBBBABABBBBBABBABA--BAABBB

*000497_00493839 B-BBB-ABABABBAA-BBBBBA-ABB-ABBAA-BABBB-BAB-AABAA-A-B--BBA-AAABBBBBBABA-BBBBABB-BA-ABAABBB

*000497_00494736 BABBBBABABABBAABBBBBBAAABBAABBAAAB-BBBABABBAABAA-A-BA-BBABAAABBBBBBABABBBBBABBABA--B--BBB

*000497_00603892 B--BBB-BABABBAABB-BB-AAA-B-ABBA--BABBBABAB-AABAA---B--BB-BAAABBBBB-ABA-BB-BABBABA--BAABBB

*000498_00101068 B--B-BBAABAA-ABAAABBAAB-ABBAABABAAABBABBAB-A--BABAABAABAAABABA-AABAB-AAA-BABBAAAAAABBABBB

*000498_00174907 B-BB-BBAABAAAABAAABBAA--ABBAABABAAABBB-BABBAABBABAAB--BAAABAAA-AABABBAAA-BAABAAAAAABB-BBB

*000499_00019301 --A-AAABAABAA-B--AAABBBBBABAABBBBBA-ABAAA--BBB-AABAB---AAAA-AABBA-A-ABB-B-B--BBBAAABB-ABB

*000500_00000797 B-BBBBABB--BBAABABBAABBAA-AB-BBBAAABBBBBAB-BBAABBBBAAABBBBABAAABA-BAAAB-BBBABAABBAABBBBAA

*000500_00069998 B--BBBABBA-BBAABABBAABBAABABABBBAA-BBBBBAB-BBAABBBBAA--BBBABAAABA-BAAAB-BBBABAA-BA-BBB-AA

*000500_00269771 BAB-BBABBA-BBAABABBAABBAABABABBBAA-BBBBBABBBBAABBBBAAAB-BBABAA-B--BAAAB-BBBABAABBAABBBBAA

*000501_00151655 B-ABABAABBABA-BBBA-AA-AAAABBBBAAAAA--A-B---AAABB--BAAAAB-A-AAAAAAABB-AAB-A---AA-BAAAAABAA

*000502_00012876 -BBAB-BBBAAABABAABABABAAB-AAABABBBBA-BBBBBAB-ABAAAAABABBABBAABABAAB-BAABBAAAAB-BABBB---AA

*000502_00043403 -BBAB-BBBAAABABAABABABAABBAAABABBBBAABBBBB-B-ABAAAAABABBABBAABABAAB-BAABBAAAABBBABBBBBBAA

*000502_00139535 -BBAB-BBBAAABABAABABAB-ABBAAABABBBBAABBBBB-B-A-AAABABABBABBAABABAAB-BAABBAAAAB-BABBBBBBAA

*000502_00262031 --BAB-BBBAAABABAABABABAABBAAABA-BBBAABBBBBAB-ABAAABABABBABBAABABAAB-BAABBAAAABBBABBBBBBAA

*000503_00246870 AAABAAAABABAABAABAA-BABBBBAABBAABABAAABBABABBAABAAAABBBBAABBAB-BBAABBAABBAAAA-ABBBBAAA-AA

*000503_00271905 AAABAAAABABAABAABAA-BABBBBAABBAABABAAABBABABBAABAAAABBBBAABBAB-BBAABBAABBAAAA-ABBBBAAA-AA

*000504_00261660 AABBBAAA-ABABBBAABABBABBBBAAABABAAAAAABABBAB-AAABBAA-BBBBABBAAAABABBBAABBBAAB-ABBBBAAABAA

*000505_00066359 ABBABABAAA-ABBAB-BB-AABAABBAAB-AAAAAAAAAABAABBBAAABBBA-BBAAABABBBABABAAAAABAAABAABBAAABBB

*000505_00093260 A-BABABAAA-ABBABBBB---BAA-BAAB-AAA-AAAAA-BAAB-BAAABBBA-BBA-ABABBBABAAAAAAAB-AABAAB-A-ABBB

*000505_00182586 ABBABABAAA-ABBAB-BB-AABAABBAABBAAAAAAA-AABAABBBAAABBBA-BBAAABABBBABA-AAAAABAAABAABBAAABBB

*000506_00059929 BABABBBA-B-AAAABBAAAAAAAB-AA-ABABBBB-BABBBBBA--AABBAAA-BBAABBA-ABA-BB-B-BBBB--B-ABBBBBAAA

*000506_00188678 BABABBBAAB-AAAABBAAAAAAAB-AAAABABBBB-BABABABAB--ABBAAA-BBAABBA-ABA-BBBB-BBBB--B-ABBBBBAAA

*000508_00063904 BBBABBBBBBAAABABAAABBBA-BABABBBAABABBB-B-B-ABBAAAABA-BBA-BBBBBBB-BBBBBABAABBAB-BAAABBAAAA

*000509_00094986 AABBBAAB-ABBBABAABABBBABBAAABABBABABBBBBBB-BBAAA-AAABBABBABAAABABBBBAABBBABABA-BBAAAAABAA

*000510_00020356 ABAAAABBAAAABAABBBAAAABAAABBABBBAA-A-BABAB-BA-BAAAAABBAABABA-AABAAABAABABBBBA-ABBBBABABAA

*000510_00299308 A-AAAABBAAAABAABBBAAAABAAABBABBBA-AA-BABAB-BAABAAAAABBAABA-A-AABAAABAABABBBBA--BBAAAB-BAA

*000510_00381093 ABAAAABBAAAABAABBBAAAABAAABBABBBAAAA-BABABBBAABAAAAABBAABABA-AABAAABAABABBBBA-AB-AAA-ABAA

*000511_00123030 BABBBBAAAAAB-AAAB-BBA-BB-BBAAAABBABBBABAABAAAAAAABAA-BAABAAAABAAAABABAABBAABBBBBABBA-BAAA

*000512_00083078 BABBBBB-BABABAB-ABABABAABBAAABABBB-AABB-BBABAAABAAA-BABAAAAAABABAAABBAABB-AAAABBBBBBBBBA-

*000512_00092462 B-B--BB--ABAB-B-ABABA--A-BAAABA-BB-AABBBBB-BAAA-AAAA-A-AAAAAA--BAAA-BAABB-A-AABBBBB-BBBAA

*000512_00186012 BAB-BBB-BABAAAB-A-A-ABAABBAAABABBBBAABBBBB-BA-ABAAAABABAA-AAABABAAA-BA-BB-AAAABBBBBBBBBAA

*000516_00045065 ABBABA-ABBBA-BBBB-AAABAAAB-AAABAB-BBABBBBAAABBBAABAAAABBB-BABBAAAABBABBAAABBBABB-BB-AB-AA

*000518_00170542 BBBABBA-ABBA-ABAB-AAAABB-AABAABAAAAAABBBABB-AA-BAA-ABBBAAAAABAAAABAABAABB-BAABABBBBBBBBAA

*000518_00277370 BBBA-BA-ABBA-ABA-AAAAA-B-AABAABA-A-AABB-ABB-AA-BA-BABBBAAAAA-AAAABAABAA---BAAB-BBB-B--BAA

*000520_00062449 A-B--AABAABA-BB-B-AAABA-ABBABAABAAAAABBABB-AAAB-BBABBBABBBABABAAB-AB-AA--AABBBBA-BBB-A-B-

*000521_00421390 A-BABAABAAAAAAAABAABBAA-BABABBBBBBBBABBBABBAAAABBA-BBAAAAABAAABBBABAAAB-AABAAABBBAAAAB-BB

*000523_00212436 BBBBBBBBABABBBABBBBABAA-ABBAABBABBBA-BBB-BABB-AABB-A-BABBABBBBBAABAABBBAAABABBBA-A-BB-BAA

*000524_00226834 B--A-BB-AABABBBBAB-ABBB-BABABBBABBB-BA-ABB-BBBBAAAAABABAABBBBABBBAAABBBBBAB-A-BBABBAA--AA

*000524_00226920 BBAABBBBAABABBBBAB-ABBBABABABBB-BBBBBAAABB-BB-BAAAAAB-BAABBBBABBBA-ABBBBBABAAAB-ABBAAB-AA

*000526_00010151 --BABBB-ABA-BBABABBA-A-AABB--B-A-ABAABBB-BAB---BBAAA-BABA-BBBBBA-BAABBBABA-AB--AA-----BAA

*000526_00170115 BBBABBB-ABABBBABABBABAA-ABBAABBABABAABBB-B-BBAABBA-AABABA-BBBBBA-BAAABBABA-AB-BAAAABBBBAA

*000526_00292707 ABBABAB-ABABBBABABBAB-A-ABBAABBABABAABAB-BABBAABBAAAABABA-BBBBBA-BAAABBABA-ABBBAAAABBBBAA

*000527_00027157 B-AAABAABBB-AAB-BAABBABBABBAAAA-BBBAAA-AABAABBBBBABBAABABAABABBBBAABA-AAABB-ABBAAAAB-A-BB

*000527_00233929 BB-AABABBBB-AABABAABBAAB-BBAAAAA-B-AA---AB-AB-BBBABB-ABABAABAB-BBAABAAAAABBA-BBA--ABBABBB

*000530_00279550 AA-ABAABAAAAA-AABAABBBA-BA-ABBBB-BB-ABB-AB-AA-ABBABBBAAAAA-AA-BAB-BAA--AAABAAA-BBA-A-BBBB

*000531_00017620 A-ABAABB--B-AA-A-AAA--ABBBBBABBAAA-AA-A-AAABABABBAAABAABBAAABBABB-ABBB--BABAAABAABBBBAAAA

*000531_00024271 AA-B-ABBABB-AAAABAAAAAABBBBBABBAAAA-A-A-AAABABAB-AAABAABBAAA-BABB-ABBBAABABAAA-AABBBBAAAA

*000531_00212044 A--BBABB-BBBABAABA----ABBBB-ABB-AA-AA-ABA-ABAB-B-AAABAABAAAA---B-AABBBAABA-AAABAABBBBA---

*000531_00250501 A--B-ABBABB-AB-ABAB-BAABBBBB-BBAAAA-A-A-AAABAB---AAABA-BA-AA-BBBB-ABBBAABABAAABAABBB-AAAA

*000533_00046994 BBABABABAABBAB-ABA-BBABABBABBBB-BBBAAAABABBBBBABBBBABAAAABABAABBA-B-AA-BAABBBAAB-AAAAAAAA

*000538_00241307 BAAAABABBABBBBBBABAAAAAAAABAAABBBBBBBBABBB-AAAAAB-ABBBAAAABABBAB-AABBABBBBBAB-ABBBBABBBBB

*000539_00269072 A--B-ABAABBBABBBAAAA-BBAAABAABB-BBBAABAB-BABBA--BBAAA-AB-ABBABB-AABAB-BA--B-AA-AB--A-BBAA

*000539_00312609 A-BBB-BA-BBBABBBAAA-BBBAAABAA-B---BAABAB-B--BA-BBBAA-BA-AAB-A-BAAABABBBAB-BAA--A-AAAB-BAA

*000539_00318491 AAB-BABAAB-BABBBAAA-BBBAAABAABBBB---ABAB-BABBA-BBBAAABABAAB-ABBAAABABBBAB-BAA-BAAA-ABBBAA

*000540_00053076 AABABABBABBAAA-BBABABB-ABBBBBA-AAAABBB-BAAAAABAB-AABABAAABABAABBB-BBBAAABBABBBAABBBAAABBB

*000540_00126373 AABABABBABBAAA-BBABABB-ABBBBBA-AAAABBBABAAAAA-AB-AABABAAABABAABBB-BBBAAABBABBBAABBBAAABBB

*000540_00228661 AABABABBABBAAABBBABABB-ABBBBB--AAAABBBABAA-AABA--AABABA-ABABAABBBBBB-AAABBAB-BAABBBA-ABBB

*000540_00317989 AABABABBABBAAA-BBABABB-ABBBBBA-AAAABBBABAAAAA-A--AABABAAABABAABBB-BBBAAABBA-BBAABBBAAABBB

*000541_00121433 ABABAAABABBBABBA-AABBAAB-BABBABBABAAAABBAA-BB-AAABBABABAA-ABAABBABB-BAABABBBBAABBBBAAAAAA

*000541_00121519 ABABAAABABBBABBA-AABBAAB-BABBABBABAAAABBAABBBAAAABBABABAABABAABBABB-BAABABBBBAABBBBAAAAAA

*000541_00204118 A-ABAAABABBBABBAAAABBAAB-BABBABBABAAAABBAABBBAABABBABABAABABAABBABB-BAABABBBBAABBBBAAAAAA

*000541_00225645 ABABAAABABBBABBAAAABBAAB-BABBABBABAAAABBAABBBAABABBABABAABABAABBABB-BAABABBBBAABBBBAAAAAA

*000541_00274658 ABABAAABABBBABBAAAABBAAB-BABBABBABAAAABBAA-BBAABABBABABAABABAABBABB-BAABABBBBAABBBBAAAAAA

*000541_00274744 ABABAAABABBBABBAAAABBAABABABBABBABAAAABBAABBBAABABBABABAABABAABBABB-BAABABBBBAABBBBAAAAAA

*000542_00059547 BABABBAABBBAAAABAAAABB-BB-ABBABBBBBBBBBABABAAABBAAABAABBAABABABAABAB-BABABBBBAABAAABBAABB

*000542_00162149 BAB-BBAA-BBAAAABAAAABB-BB-ABBABBBBBBBBBABABAAABBAAABAABBAABABABAABAB-BABABBBBAABAAABB-ABB

*000545_00097280 BBBBBBA-ABABAAABBABBBAAABABABB-AABAAABA-ABBAAAAAAA-BABBBB-BAABB-ABAAABBBBABAABABAAABABBBB

*000545_00298668 B-BBBBAB-BABAAABBABBBAA-BABAABABABA-A-A-AB-BAAAAAA-BABBBB-BAABB-A-AAABBBBABAAB-BAAA-B-BBB

*000547_00080477 B--ABBABBAAAA-A-BA-BBB-B-ABAAB-AA-A-BBBB-B-ABBAAB-AA--B--ABAA-BABBBABBABAABB-BB-AAABBA-AA

*000547_00270529 B-BABBABBAAAABAB-ABBB-ABAABAABB-AB-BBBABAB-AB-AABAAA-BB-BABAA-BAABBABBABAABBABBA-AAB-AABA

*000547_00318498 B-B-BBABBAAAABABBA-BBBAB-A-AABBAABABBB-BABB-B-AABAAA-BBBBA-AA-BAABBABBABAA-B-BB-AAABBAAB-

*000549_00264340 BAA-A--BAB-BB-ABBB-BA-AABBB-ABA-BB-B-ABBBB-BABB-BBBB-A-B-ABBBAABA-BB-AABB-ABAAB-AAAABB-BB

*000549_00314818 B---A-BBABBBB-ABBBB-ABAABBB-ABAA-B-BBABBBB-BABBBBBBBB--B-ABBBAABA-BBAAABB-ABAABBAAAA-BBBB

*000550_00164664 BBABABBBBABBBBABBBABA-AABBABB-BBAAAAABBBBA-AABBAAAAABB-BAB-BBA-AAAABAABABABBB-BAABBAABBAA

*000550_00169816 BBABABBBBABBBBABBBABA-AABBABB-BBAAAAABBB--BAABBAAAAABB-BABBBBA-AAAABAABABABBB-BAABBAABBAA

*000550_00194372 B-A-AB-B-AB-B-AB--ABAAAAB-ABB-BBAAAAABB-AABAABBA--AABB-BABBBB-A-AAA-AA-A-A-BBABAA---AB-A-

*000551_00234903 AAABAAABABABB-AAABBABABA-BBBABBBAABBBAB--AB-B-BA--BABAABABBAABBABBABBA-AABBBAAAABAA-AAAAA

*000552_00006969 BBABABABAABBAB-ABA-BBABABBABBBBABBBAAAAB-B-ABBABBBBABAAAABABAABBA-B-AA-BAABBBAABBAAAAAAAA

*000553_00011842 AA-B-ABAAABBABBBBAA-BABBAABABBBAABAB-AAABBBABABBABAABAABAAABBABAAB-AABBABABABABABBBBBBBAA

*000553_00012112 AA-B-ABAAABBABBBBAA-BABBAABABBBAABAB-AAABBBABABBABAABAABAAABBABAAB-AABBABABABABABBBBBBBAA

*000553_00220902 A-ABBABAAABBABBBBAAA-ABBA-BAB-BAAB-B-AAABBBABAB-ABAAB-ABAAABBABAAB-AA--ABABABABABB-BB---A

*000555_00175737 BBABABBAABBBBBAB-BBABAABBBAAABBABBBAABBBAB-BBAAAB--A-BABB-BBBBBAABAABBB--ABABBBBAA-BBABAA

*000556_00158667 A-BABAAAB-BABBBBABAA-BAABAABBBA-BABABBBAAAABBBBBAAA--ABBBABA-BAABABABBAAABA-BABABAA-ABBAA

*000560_00014968 BBAAABABABABBBBB-BABBBABBAAABAAA-B-AA-AAABBBBA-AAAAAAAAAB-B-BABABBABABABBAABAABBAAABBABAA

*000560_00253152 B---ABA-AB-BBBA--BAB-BABBAAABA-A-B-AA-AA-B-BBA-AAAAAA-AA--BABAB-BBAB-B--BAABAABBAAABBABAA

*000561_00018644 B-ABABBABBAAAABBBAABAAAABB-BAAAABBB-ABBBBAB--AABBABB-BABB-B-AAABAABBAAAABBABBA-AAAAAA-BBB

*000561_00170094 B-AB--BABBAA-A-BBAABAAAABB--AAA-AB-AABBBBA-B-AABBABBBBABBAB-AAABAABBAAAABBABBA-AAAAAAABBB

*000562_00165227 ABB-BAABAABABBB-B-AA-BAAABBABAABBA--ABB-BBAAAABABBBBBBABBB-B-BA--A-A-A---AAABBBBA-BB--ABB

*000562_00334100 A-BBBAABAABA-BB-B-AAABAAABBABAABAAAAABBABBAAAAB-BBABBBABBBABABAABAAA-AAABAAABBBAABBB-AAB-

*000562_00355356 ABBBBAABAABA-BB-B-AAABAAABBABAABAAAAABBABBAAAABBBBABBBABBBABABAABAAA-AAABAAABBBAABBBAA-B-

*000562_00355442 ABBBBAABAABA-BB-B-AAABAAABBABAABAAAAABBABBAAAAB-BBABBBABBBABABAABAAA-AAABAAABBBAABBBAAAB-

*000563_00003199 A-B-BABAB-BBABA-B-A---B-AAB-ABAB-BBB-B-BBBBBA-B---BABABBABBBBA-BB-BB-A-ABAABA-AB-B-B-AAAA

*000563_00180711 AABBBABABBBBABABBAAABABAAABAABABBBBBBBBBBBBBAABBBABABABBABBBBABBBBBBBAAABAABABABBBBBAAAAA

*000563_00236051 AABBBABABBBBABABBAAABABAAABAABABBB-BBBBBBBBBAABBBABABABBABBBBABBBBBBBAAABAABABABBBBBAAAAA

*000564_00185755 AA-BBABB-BBAABABBABABAAB-BBBABBAAAAAA-A-AA-BAB-BBAAABAABAAAA-BB-B-ABBBAABABAAABAABBBBAAAA

*000565_00043604 BBABAB-BBABBB-ABBB-B--A--BABBBBBAA--A-B-AAB-ABBAA-AABB-B--BBBAAA-AABAABABABBBABAABB-AB-AA

*000565_00069676 BBABABBBBABBBBABBBAB--AABBABB-BBAAAAABBB-ABAABBAAAAABB-BABBBBA-AAAABAABABABBB--A-B-AABBAA

*000565_00155638 BBABABBBBABBBBABBBAB--AABBABB-BBAA-AABBB-ABAABBAAAAABB-BABBBBA-AAAABAA-ABABBB-BAABBAA-BAA

*000565_00251535 B-A-ABBBBABBB-A-BBAB--AABBA-B-BBAAAAABBB-ABAA-BAAAAA-B--A-BBBAAAAAABAABABABB---AABBAA-BAA

*000567_00036151 ABAAAA-ABBBA-BBBB-AAABAAAB-AAABAB-B-ABBBBAAABBBAABAAABBBB-BABBAAAABBABBAAABBBABA-BB-AB-AA

*000567_00064291 ABAAAA-ABBBA-BBBB-AAABAAAB-AAABAB---ABBBBA-ABBBAABAAABBBB-BABBAAAABBABBAAABBBABA-BB-ABBAA

*000572_00206868 A-AAAABBAABABBAA-BA-AA-ABB-AAA-A--BAAAAAABAB-BBB-BBAB-A-BAAABBBBBABABABABABAAAAAABBAABBAA

*000575_00044431 ABAAAAABAABAAABBAAAABBBBBABAABBBBBAAABAAABBBBBBAABABABBAAAAAAABB-AABABBBBABABBBBAAABBAABB

*000575_00148226 A-AAAAABAABAAABBAAAABBBBBABAABBBBBAAABAA-BBBBBBAABABABBAAAAAAABB-AABABBBBABABB-BAAABBAABB

*000576_00203536 BBAAABBBBBBBBBABBBBB--AABBABB-BBAAAAABAB-A-A-BBABAAABB-BABBBBA-AAAABAABAB-BBB-BAABBAAABAA

*000578_00300180 BBABABBBBABBBBABBBABAAAABBABB-BBAAAAABBBBABAABBAAAAABB-BABABBAAAAAABAABABABBB-BAABBAABBAA

*000578_00333185 BBABABBBBABBBBABBBABA-AABBAB--BBAAAAABBBBABAABBAAAAABB-BABABBAAAAAABAABABABBB-BAABBAABBAA

*000578_00382492 BBABABBBBABBBBABBBABA-AABBABB-BBAA-AABBBBABAA-BAAAAABB-BABBBBAAAAAABAABABABB--BAABBAABBAA

*000579_00251796 B-BABBABBABABAABABAAAABABABBBAABBBBBBBABABBABBAAABBBABAAABBABBA-BBAABBABAAAAAAABABBBBABBB

*000580_00112429 BBBBBBAABABABBABABABABB-B-BBBA-AAAABBBABABBA-BAAABBBBBAB-BBAABA-BBABBAAAAA-AAAAAABBBBA-BB

*000580_00319269 BBBBBBAABABABBABABABABB-B-BBBAAAAAABBBABABBA-BAAABBBBBAB-BBAABA-BBABBAAAAA-AAAAAABBBBA-BB

*000583_00217386 AAABAAABA-AB--AAA-BABAAABBAAABBBAABBBABAB-B-BBBB-BBABBABABBAABBABBAB-B-BABBBBABBAA-AAAAAA

*000584_00241550 BBAAABAAAABBABBAAAAABBAABABBBAAA-B-AAAABBABBABBBAAAAAB-A-BBBBBBBBABABB---AABABB-BBBAABAB-

*000585_00079278 BABBBB-AAAABB-BAABABBAAABBBBBABAAAAAABAAAAABBABBBABAAABBBA-BBABAAB-BBBBBAAABAABBABB-ABAAA

*000585_00359751 BABB--BAAAABB--AABABB--ABBBBBA---A-AABAA-A-BB----ABAA-BBB-ABBA-AAB-BB-BBAAABAABBABBAA-AAA

*000586_00090443 BBBABBBBAAB-ABA-AAABABAAABABBBABABAAABAAAABAABAAABBBBABABBBA-AABBBAAAABABBAB-AAABBBBAAABB

*000586_00130566 BBBABBBBAABAABABAAABABAAABABBBABABAAABAAAABAA-AAABBBB-BABBBAAAABBBAAAABABBAB-AAABBBBAAABB

*000587_00133829 BABBBBB-BABA-AB-B-AAABBABAAAABBBBABAABBAA-BAAABBAAAABABAAAAABAABAAA-BAABBBBAAAABBBBB-BBAA

*000587_00184022 B-BBBBB-BABA-AB-B-AAABBABAAAABBBBABAABBAABBAAABBAA-AB-BAAAAABAAB-AA-BAABBBBAAAAB-BBBB-BAA

*000587_00188473 BABBBBB-BAB-BAB-B-AAABBABAAAABB-BABAABBAABBAAABBAA--BABAAAAABAABAAA-BAABBBBAAAABBBBBB-BAA

*000587_00224874 B-BBBBB-BABAAAB-B-AAABBABAAAABBBBABAAB-BABBAAABBAA-ABABAAAAAB-ABAAA-BAABBBB-AAABBBBBBBBAA

*000588_00012734 B-BBBBB--BBA-BBABA-AB-BA--B-AB--AAABBBAABBBBABB-BBBA--BABAB-AAAA-ABBBBBBABBAA-ABBAA----AA

*000588_00178388 B--B-BBBBBB-ABBA-AAA-A-A--BAAB---A-B-BAABBBB-BB--BBAB-BABABBAAAAAABBBBBBABBAAB-B--A--BAAA

*000589_00201251 B-BABBAAABA-A-BB-AABA-ABB--ABAB--BBBBBBBAAABABBBABAB-BABBAAA-AAAABAAAABAABBAABAABAAAAA-A-

*000591_00048899 A-AAAA-ABAAABBA-BBBBBBBBB--BB-BA-A-BBBABAB-A-BA-BBBB-BABBBBAABABBBBBBBBABA-AAABAABBAAA-BB

*000591_00355600 --AAAABBBAAABBAABBB-BBBBB-BBBA--BA-BB-AB-BBA-BAABBBBB-A--BBAAB-B-BBBBB--BA--AA-AABB-A--BB

*000592_00235638 BABABBAA-AABB-BBABBBBAAAABBBBAB-AAA-AAAABA-ABBBBBBBABABBAA-BBABBBBABABBBAB-BAABB-BBABAAAA

*000592_00235724 BABABBAAAAABB-BBABBBBAAAABBBBABAAAAAAAAABA-ABBBBBB-ABABBAA-BBABBBBABABBBAB-BAABBABBABAAAA

*000593_00561107 BABBBBAAAAAB-AAA--ABAABBABBAAAABBABBBABAABA-A-AAABAAABAABAAAABAAAABABAABBAABBBBBABBA--AAA

*000596_00147079 AAAB-AABBBBAA-AB-AAABB--BBBBBA--A-AAA-ABBAAABBABBAABAAABABA-AABBBBBBBAAABBABBBAB--BAAABBB

*000596_00248736 A-ABAABB-BBAAAABBAAABB--BBBBBABAA---ABABBAAABBABBAABA-ABABAAAABBBBBBBAAABBABBBA-BBBAAABBB

*000596_00259098 AAA-AA-BABBAAAABBAAA-B--BBBBBABAA--AA-A--AAABBA--AABAAABABAAAAB--BB--AAA-BAB--A-B--A-AB--

*000596_00386661 A-AB-A-B-BBAAAABBAAABB--BBB-BABAA-A-AAABBAAABBABB-ABAAAAABAAAABBBBBBBAAABBABBBAA-BBA--BBB

*000598_00085443 A-B-BAABAAA-AAAABA-BB-A-BA-ABB-BBBB-ABBBAB-AAAABBA--B-A--ABA-ABABABAA-BBAA--AABB--A-ABBBB

*000598_00088964 ABBABAABAAAAAAAABAABBBA-BA-ABBBBBBBBABBBABBAAAABBA-BB-ABAABAAABABABAAAB-AABAAABBBAAAAB-BB

*000599_00032819 BBA-ABBBABB-BAABABBBAABAAAABBBBBBBBAABBBABABBABBBBBBA-ABABABABABABB-ABBABABBBAAABBBBBBABB

*000599_00184417 BBA-ABBBABB-BAABABBBAABAAAABBBBBBBBAABBBABABBABBBBBBA-ABABABABABABB-ABB-BABBBAA-BBBBBBABB

*000600_00405132 AAABAAABABBAAAABBAAABB--ABABBABAA-AAA-AABAAABBABBAABAAABABAAAABBBBBABAAABAAB-BAABBBAAABBB

*000601_00076120 BABBBBAABAAB-BAAB-BAA-BBBBBBAAABAAAABABAABAAAAAAABAA-BAABAAABBAAABBABABBBAABBBBBABBABBAAA

*000601_00084692 B-B-BBAABAAB-BAAB-B-A-BBBBBBAAABAA-ABAB--BAAAA-A-BAA-BAABA-ABBAA-BBABA--BAABBBBBABBA-BAAA

*000601_00088228 B--B--AA-AAB-BA--BBA-BBB--BBAA-BAAAA-ABAAB-AAAAAA-AA-B-ABAAA-BAAABBABABBBAA-BB-BABBA-BAAA

*000601_00229554 BABBBB-ABAABABAAB-BA-BBBBBBBAAABAAA-BABAABAAA--AABAA-BAABAAABBAAABBABABBBAAB-BBBABBA-BAAA

*000603_00057210 BAABAB-BBAABAABABABABAABAABBBBAAABA-AAABABABBABBABAAABAA--AAABBBBBAA-AABAAAAAABBBAAABAAAA

*000603_00156998 BAABAB-BBAABAABABABABAABAABBBBAAABA-AAABABABBAB-ABAAABAAB-AAABBBBBAA-AABAAAAAABBBAAABAAAA

*000603_00261358 BAABABABBAABAABABABABAABAABBBBAAABA-AAABABABBABBABAAABAAB-AAABBBBBAA-AABAAAAAABBBAAABAAAA

*000605_00121730 ABAAAABBBBBA-BBBA-AAAABBAB-ABABBAAA-ABABBA-AB-BAABAAAB-BB-BABBABB-BA-BAAAABBBABABBBAAB-AA

*000605_00241455 ABAAAA-BBBBA-BBBA-AAA--BB-AABABBAAA-A-ABBABAB-BA--AAABAABABABBABBABAA-AAA--BBABAB-BAA-BAA

*000606_00241837 B-ABABABAABBBBBBBB-A-BAAAABAAB-ABB-B-ABBBBABAB--ABAB-AABAAA-AABAABBBAAAABABAABBAAAAA-BBBB

*000607_00278297 ABAA-AABAABABABBBBAAAABAAA-AAABBBB-BBBAB-BBAABAAA-ABBBAAAABAABAB-AABBA-BBAB-B-AB--BB-ABBB

*000608_00036976 BABA-BAAABBA-AABA-AB-B-BB-ABBABBBBBBBBBA-A-AAABBAAABAABBA-BABABAA-ABABABA-BBBAABAAABBAABB

*000608_00131173 BAB-BBAAABBA-AABA-ABBB-BB-ABBABBBB-BBBBABABAAABBAAABAABBA-BABABAABAB-BABABBBBAABAAABBAABB

*000608_00133148 BAB-BBAAABBAAAABA-ABBB-BB-ABBABBBBBBBBBABABAAABBAAABAABBA-BABABAABAB-BABABBBBAABAAABB-ABB

*000608_00136891 BAB-BBAAABBA-AABA-ABBB-BB-ABBABBBBBBBBBA-ABAAABBAAABAABBA-BABABAABAB-BABABBBBAABAAABB-ABB

*000608_00228939 BAB-BBAAABBA-AABA-ABBB-BB-ABBABBBBBBBBBABABAA-BBAAABAABBAABABABAABAB-BABAB-BBAABAAABB-ABB

*000609_00119638 A-AAAA--BAAABBA-BBBBBBBBB-BBB-B--AABBBABABBA-BAABBBBBBABBBBAABAB-BBBBBBABA-AAABAAB-AAA-BB

*000610_00251186 A-AAAABAAABABBAA-BA-AA-ABB-AAA-A---AAAAAABA--BBBAABA--ABBAAAABBBBABABABABABAAAAAABBAABBAA

*000610_00276937 A-AAAABAAABABBAA-BA-AA-ABB-AAA-A---AAAAAABA--BBBAABAB-ABBAAAABBBBABABABABABAAAAAAB-AABBAA

*000612_00173458 --BBBABABBBBABBBAAAABBBAAABAAB-BBABAABABBB-BAA-BBBAA-BABAABBAABAAABABBBAB-BAAABA-AAABBBAA

*000614_00049193 ABAAAA-BAABABABBBBAAAABAAABAAABBBBBBBBABBBBAABAAA-ABBBAAAABAABABBAABBABBBABAB-ABABBBBABBB

*000614_00242005 ABAAAAABAABABABBBBAAAABAAABAAA-BBBBBBBABBBBAABAAA-ABBBAAAABAABABBAABBABBBABAB-ABABBBBABBB

*000615_00163063 AABBBABABBBB-BABBAAABABAAABAABABBBBBBA-B-ABBAABBBAAABABBABBBBABBBBBAB-AABAA--BABBBBB-AAA-

*000615_00192671 AABBBABABBBBABABBAAABABAAABAABABBBBBBAB-AA-BAABBBAA-BABBABBBBABBBBBAB-AABAAA-BABBBBBAAAAA

*000615_00244802 AABBBABABBBBABABBAAABABAAABAABABBBBBBABBAABBAABBBAAABABBABBBBABBBBBAB-AABAAA-BABBBBBAAAAA

*000617_00301444 A-BAB--BAAABBAAAABBBBBBBBABABABBAB-BBBAB-B-BAAAA-AAABA-BB-BAAABABABB-BBBA-BBBBAB-AA---A--

*000618_00028128 AABBBABABBBBABABBAAABABAA-BAABABBBBBBABBAABBAABBBAAABABBABBBBABBBBBAB-AABAAA-BA-BBBBAAAAA

*000618_00218252 AABBBABABBBBABABBAAABABAAABAABABBBBBBABBAABBAABBBAAABABBABBBBABBBBBAB-AABAAA-BABBBBBAAAAA

*000620_00012784 ABBAB-B-BAAABABAABABABAABBAAABA-BBB--B-BBB-B-ABAAAAABABBABBAABABAAB-BAABBAAAABBBABB-BBBAA

*000621_00297050 AAABAAABABABB-B-ABBABAAABBBBABBBAABBBABAB-B-BBBB-BBABBABABBAABBABBAB-B-BABBBBABBAAAAAAAAA

*000624_00063253 BBBABBABBABAABBBBAABABAABBABBBBABBBAABBAAAA-ABAABBBB-A-BBBAA-A-BBBAAABBAABABAABABBBBB-ABB

*000624_00067530 BBBABBABBABAABBBBAABABAABBABBBBABBBAABBAAAA-ABAABBBBBA-BBBAA-A-BBBAAABBAABABAA-ABBB-B-ABB

*000624_00205777 BBBABBABBABAABBBBAABABAABBABBBBA-BBAABBAAAA-ABAABBBBBA-BBBAA-A-BBBAAABBAABABAABABBBBB-ABB

*000624_00308853 BBBABBABBABAABBBBAABABAABBABBB-A-BBAABBAAA--ABAABBBBBABBBBBABA-BBBAAABBAABABAABABBBBB-ABB

*000625_00167525 B-ABABAAABABB-A-BBAAAAAABA-BBA-BAAAB--ABAABA-ABBBBB-A-AAAA-AAAAA--BB-AABA-BB-AABBAAA-ABAA

*000629_00109955 ABBBBABAAABAAABAAAAAABABAAAABB-BBBBBBBBAAABAB-ABBB-ABAA-BAAAAAAB-BBBABBBBA-BBBABBBBBBAAA-

*000630_00187922 B-B-BBAAAABBBBABABB-BAAAAAABAB-BBABBBABAABAAAB-BBBABBAABBA-ABBBABAABBABBAA-ABBAABBBBAAABB

*000630_00189058 B-B-BBAAAABBBBABABB-BAAAAAABAB-BBABBBABA-BAAAB-BBBABBAABBA-ABBBABAABBABBAA-ABBAABBBBAAABB

*000630_00323096 BBBBBBAA-A-BBBABA-B--AAAAA-BBB--BA-BB-B----AAB-BBBAB--ABBABABBB-BA-BBA-BAA-ABBA-BBBBAAABB

*000631_00040605 BBB-BBBBABABBAABBBBBBAB-ABBBBBABAAAA-BB-BAAAB-BBAA-BBABAABBABABABA-BBAABAAAABBBABBBAAABBB

*000631_00106852 BBBBBBBBABABBAABBBBBBAB-ABBBBBABAA-A-BB-BAAABABBAABBBABAABBABABABA-BBAABAAAABBBABBBAAABBB

*000632_00292308 BABABBBBB-ABBBAABBBB--BB-ABABAABBABAAAABABAABAAAA-ABBBBABAAAA-BAAAAABAAABAABBBBABBBA-BA--

*000632_00333603 BABAB--BBAA-BB-ABBBBA-BBBA-ABAA-BABAAAAB-B--BAA-AAA-B-BABAA---AAAAAABAAA-AA-BBBA-B-A-BABA

*000634_00239438 B-BABBBBBAABAABBBAABBBBBABBABA-AAAAAA-AABBAAAAABBAAAAAB-BAABBABABBAAAABBBABBAAA-BBBAA-BAA

*000635_00357588 BABABBABAA-BBA-AABAAAAAAA-BBBBBAABA--AABABABAABBABAAABAABAAAABABB-AA-AABAABAAAABBAABBAAAA

*000636_00206824 BBAAAB-AB-ABBAAABBBBBAABABABBAABBBA-AA-ABA-BBABBABABBAAA-BB-ABAAAB-BBBABBABBAABBBBB-BAABB

*000636_00212492 BBAAABBAB-ABB-AABBBBBAABABABBAABBB--AA--BABBBABBABABBAA-ABB-ABAA-BBBBBA-BABBAABBBBB-B-ABB

*000638_00137985 ABBABABAAA-ABBAB-BBAAA-ABBBAAB-AAAAAAAAAABAAB-BAAABABA-BBAAABABBBA-ABAAAAABAAAAAABBAAABAA

*000638_00241640 ABBABABAAAB-BBABABB-AABABBBAAB--AA-A-A-AAB-ABBBA-A-ABA-BBAAA-ABB-A--BAA--ABAAAB--BBAAA-AA

*000642_00212578 A-BABAAB-AA-BBAABB-BBBBB--BAAABBABABBBABBBAAAAAABAAA-B-BBABAAABABBBBABAB-ABBABBBBAABBBABA

*000642_00302176 AAB-BAABAAAABB-ABB-----B--BAAAB-ABABB--BBB-AAAA-BAAAA-BBB-B-A--ABBBBA-AB--BB-B-BBAABBBABA

*000643_00224787 B---BBBBAB-BBA-BBBBBBAB-A-BBBBABAAAABBB--A-ABA--AABBB-B-ABBA-ABABABB-AA-AA-ABB-ABBB--A-BB

*000643_00260396 B-B-BBBBABABBAABBBBBBAB-ABBBBBABAAAA-BB--AAABABBAABBBABAABBABABABA-BBAA-AAAABBBABBBAA-BBB

*000643_00283949 B-B-BBBBABABBAABBBBBBAB-ABBBBBABAAAA-BB-BAAABABBAABBBABAABBABABABA-BBAA-AAAABBBABBBAAABBB

*000644_00336333 BB-A-B-BAABABBBBAB-ABBBABA-ABBBABB-BBAAABB-B-BBAAAAABABAA-BBBAB-BA-ABBB-BABAAAB-AB-AAAAAA

*000645_00007336 BABBBBB-BABA-AB-B-AAABBABBAAABABBABAABBAABABAABBAAAABABAAAAAAAABAAA-BAABBBAAAAABBBBBBBBAA

*000645_00099300 BABBBBB-BABA-AB-B-AAABBABBAAABBBBA-AABBA-BABAABBAAAABABAAA-AAAABAA--BAABBBBAAAABBBBBBBBAA

*000645_00104037 B-BBBBB-BABA-AB-B-AAABBABBAAAB-BBA-AABBA-B-BAABBAAAABABAAAAAAA-BAAA-BAABBBBAAA-BBBBBBBBAA

*000645_00204752 BABBBBB-BA-A-AB-B-AAABBABAAAABBBBAB-ABBAAB-BA-BBAAAA--BAAAAAAAABAAA-BA-BBBBAAAABBBBBBBBAA

*000645_00242586 BABBBBB-BABA-AB-B-AAABBABAAAABBBBABAABBAAB-BAABBAAAABABAAAAAAAABAAA-BAABBBBAAAABBBBBBBBAA

*000645_00288156 BABBBBB-BABA-AB-B-AAABBABAAAABBBBABAABBAABBBAABBAAAABABAAAAAAAABAAA-BAABBBBAAAABBBBBBBBAA

*000648_00248133 BBABABBBABABBBBBBBBB-AAABBBBAABABBB-AAAABBBB-BBBBA-AAABBBBBBBAABAAABBAAAB-BBABBAABBABABAA

*000650_00141665 B-BABBABABAAABBAAABABBABAABBAA-AAAABBABBAB-AA-BAAABBBAA-AAABABBBAAABABA-BABABABBBBBABABBB

*000651_00055953 BB-AB-AA-BA-ABB-B-AB---B--AABAABABBA--B-B--BABABABABBBABBABAA-ABAAAAAAB-AABABBAA-AAAB-AAA

*000655_00343093 BAB--B-BBABBAABABABBBAAABBBBBBAAABAAAAABBB-ABBBAABAAABABBBBBABBBBBAA-AABAAABAABBBAAAB-AAA

*000656_00264910 ABB-BAABAABABBB-BBBAABAB-BBABAAABABAABBBBBABBAB-BBABBBA-BBBB-AAABABABBABBAABBBBB-B-BABAB-

*000657_00110646 B-BABBB-ABBB-ABBABBB-BBAAAAABAAABBBAAAAABAAA-BA-BAABA-ABAABBAAABBAA-B-BBBAA-AB-BAB--ABABB

*000657_00147422 BABABBBBABBBBABBAB-BABBAAAAABAAABBBAAAAABAAA-BAABAABA-ABAA-BAAABBBABBBBBBAA-ABBBABBAAAABB

*000657_00171899 B-BABBB-ABBB-ABBAB-B-BBAAAAABAAABBB-AAAABA-A-B-ABAABA-ABAA-BAA-BBBABBBB-BAA-ABBBABB-AAABB

*000659_00269734 B-BBBBAABBABABABBAABABBBAAAABABB---AABBA-BA-A-BABABABABBBABBBAAB-BBAABBBBABABBABBBBAAAAAA

*000660_00060512 BBAAABABAAAABAABBBAAAABAAABBABBBABAA-BABABBBA-BAAAAABBAABABA-AABBAAB-BBABBB-A-ABBAAABABAA

*000660_00133890 B-AAABABAAAABAABBBAAAABAAABBABBBABAA-BABAB-BAABAAAAABBAABABA-A-BBBAB-BBABBBBA-ABBAAABBBAA

*000661_00068493 AA-BA-BBAB-BBAB---A--A-B--BABBB-BBB-BA-AAB-BB-ABAAAAB--BA-ABB-AAABAAA-BABABA-A-BA-ABBBBAA

*000661_00211247 AABB-ABB-BAB--BABB-B-ABBAABABBB--BBBBAA-AB-BBBAB-ABABABBA-ABBAABABAAA-BABABA-AAA-A-B-BBAA

*000662_00192327 BBA-ABAA-ABA-ABAA-AA-AAAA-BBBAA--ABA-AAAAA-AABABBABAAAAA-AB--BB-ABBAAABBAA-BBAAAAAABB-A-A

*000663_00022673 B-B-BB--BBABBBAABBBBBAABBAABBA-BABB-ABAA-BBBBABB-BABBAAAABBAB-BAABBBABBBBABBBABB-BBA-AABB

*000663_00123574 BB----AABB-B-BAAB-BB-AAB-AABBA--ABB-ABAABBBBBAABBBABBAAA-BBABBB-ABBBABBBB-BBBA-B-BBABAABB

*000663_00189695 BBBABB-ABBABBBAABBBBBA-BBAABBA-BABBAABAAB-BBBA-BABABBAAAABB-BBBAABBB-BBBBABBBABBBBBABAABB

*000663_00200344 BBBABB-ABBABBBAABBBBBAABBAABBA-BABBAABAAB-BBBA-BABABBAAAABB-BBBAABBBABBBBABBBABBBBBABAABB

*000665_00267934 BABA--ABBBAAABAB-A-B-B-BB--AABB-A-AB---BA--A--AABAAA-BBAB-BAA-BA-BBABBABAABBAB-BA-ABBAAAA

*000666_00321997 B-AAABBBBBABA-A-B-BB-A-BA-ABBAAA-B---A-B-ABBAABBAAAABAAB-B-BABA--BBBBBA-AABBAABAB-AB--AAA

*000666_00373570 BBAAABBBBBABABAABABBBA-BA-ABBAAA-BA--A-BBA-BAABBAAAABAABABBBA-AAABBBBBA-AABBAABABAABAAAAA

*000667_00018474 B-AAA-AABAABABBBBAAB-BAAAAABBBA-BA--ABBABA-AA-AB-A-AAAAAAAAABABBB-ABBB-BAAAABAABBBBABABAA

*000668_00202057 BA-A-B-BBAABBBAA-BBAAABAAABAAAABBABAAABBBBABBA-AAAABAB-BAAAAB-AAAAABAAAAAAABB-AABBBAAAAAA

*000668_00220435 BAAA--BBBAABBBAA-BBAAABAAABAAAA-BABAAABBBB-BBA-AAAAB-B-BAAAABBAAA-ABA-AAAAABB-AAB-BAAAAAA

*000670_00002263 BAAAABABAABAAABBAABABAABBBBAAABBABABB-BAABBBB-BAABABBBAAAAAABAABAAABABABABAAB-ABABBBB-BBB

*000670_00114788 BBAAABAAAABAAABBAABBBAABBBBAAABBABABB-BAABBBBBBAABABBBAAAABBBBBBAAABABABABAAB-ABABBBB-BBB

*000670_00165143 BAAAABAAAABA-ABBA-BABAABBBBABABBABABB-BAABBBBBBAABABB-AAAAAABAABAAABABAAABAAB--ABBBBBBBBB

*000671_00050914 ABBBBAABBAABABABBA-AB-ABAABBBBBAAAABBAABBBBBB-ABBBBAAAABABABAABBA-BBA-BBBBAAB-ABABBBBABAA

*000672_00107306 A-BA--BAABBAB-BB-BBAA-ABAAA-BBB--A-BB-BBAA-AABA-A-ABAAAA--ABB-ABBABAABBAB-AABB-AB-BBBB-BB

*000674_00317506 AABBBAB-BBABBAABBBBBBAAB-B-B-BBABBBAAB-BAA-A-BAAAAB--B-BABABBBBA-BBBB--B-BB-BB--BB---BBAA

*000677_00009346 B-AA-BABBAABBBBABBABBBABBAAABAAA-BB-ABAAAB-BBA-ABAAAAAA-A-BABAB-ABABABA-BAABAABB-A--BAAAA

*000678_00194635 B-BABBBBBBA-ABABAAABBBABBABABBBAABABBBAB-B-ABBAA-AB-ABB-BBBAAABB--BBBBABAAB-ABBB-AAB-AAAA

*000678_00260718 B-BABBBBBBAAABABAAABBBABBABABBBAABABBB-B-BBABBAABABA-BBABBBAAAB---BBBBABAABBABBAAAABBAAAA

*000679_00034024 BBABABABAABBA--ABA-BBABBBBBBBBBBBBBAAAABABBBB-ABBBBABAAAABABAABBAAB-AA-BAABBBA-BBBBAAAAAA

*000681_00247340 BAAAAB-BBAABBBAA-BBAAABAAABAAAABBABAAAAB-BABBA-AA-ABBB-AAAAAA-AAA-AAAAAAAAABBBAABBBAABAAA

*000684_00053208 ABBABABBAA-A-BBAA-ABBBBBBBBABBAABBBAAABBBBBAB-BBBAABAABABABBAAB-AAB-BAB-AABBABBBAAABBABBB

*000684_00079889 ABBABAB-AA---BBAA-ABBBBBBBBA-BA-BBBA--BBBB---A-BBA-BAABABABB--BBAAB-BAB--AB--BBBA----ABBB

*000684_00165105 ABBABABBAA-A-BBAA-ABBBBBBBBABBABBBBAAABBBBBABABBBAABAABABABBAAB-AAB-BAB-AABBABBBAAABBABBB

*000684_00210965 A-BABABBAAAA-BBAA-ABBBBBBBBABBABBBBAAABB-BBABABBBAABA-BABABBAAB-AAB-BAB--ABBABBB-AABBABBB

*000684_00255301 A-BABABBAAAA-BBAA-A-BBBBBBBABBABBBB-AABBBB-AB-BBB-ABAABABABBAAB-AAB---B-BABBABBBA-ABBABBB

*000684_00255387 ABBABABBAAAA-BBAA-ABBBBBBBBABBABBBBAAABBBBBABABBBAABAABABABBAAB-AAB-BAB--ABBABBBAAABBABBB

*000686_00171771 B-ABABABBABBAABABABBBAA-BBBBBBAAABAAAAABBBAABABBABAAAB-BBBBBABBBBBAA-AABAAABAABBBAAABAAAA

*000689_00107295 --BAB--AAB-BAAABBA-ABABABABA-AB-BABBBB-BBABBAB-BBB-AAA-BAAABBA-ABBBBBABAAABB-B-AABBBAAAAA

*000691_00207988 BABBB-ABAABABAABBBA--AAABAABBABBB-BBABBABA-ABA-BBBABAABAABBAAABA--AAABAABABAAAAABAAABA-BB

*000691_00282129 BABABBABAABABAABBBA--AAABAABBABBB-BBABBABABAB-ABBBABAABAABBAAABA--AAABAABABAAA-ABAAABABBB

*000692_00036812 AAABAAA-A-AB--AAA-BABAAABBAAABBBAABBBABAB-B-BBBB-BBABBABABBAABBAB-AB-B-BABBBBABB-AAAAAAAA

*000693_00112023 A-AAAAAAABBA-ABAB-BBAAABBBBAAABBAA--B-BAABBBBBBABBABA-BBAAAABAAAABABABAAABA-A-AABBBB-ABBB

*000693_00252144 A-AA-A---BBAB-BA-BBBAAA-BBBA-ABB-AA-B-BAABBBBBBABBA-A-BBAAAA-AAAABABA-AAABA-ABAAB-BBB-B-B

*000693_00289597 A-AAAAAAABBA-ABAB-BBAAABBBBAAA-BAAABB-BAABBBBBBABBABA-BBAAAABAAAABABABAAABA-A-AABBBBB-BBB

*000694_00133360 BAAAABBABBAAAAB-BAABAAAABB-BAAAA-B-AABABBABBBBABBAABBBABB-BA-AABAAAAAAAABA-BBAAAAAAAAAABB

*000694_00154173 BAAAABBABBAAAAB-BAABAAAABB-BAAAA-B-AABABBABBBBABBAABBBABB-BA-AABAAAAAAAABA-BBAAAAAAAAAABB

*000695_00172920 BABABBBAAB-AAAABBA-A-ABABABA-ABAAAABBB-BBA-BAB-BBB-A-A-BAAABBAAABABBBAB-BABB-BA-ABBBBBAAA

*000697_00413348 --ABABABABBBABBBBAA-AABAA-A-BBB-BBBAAAA--B-AAB-BB-BABABAA-B-B-ABABBB-AABAABABAAB-AAAAAAAA

*000697_00415597 BA-BABABABBBABBBBAABAABAA-AA-BBABBBAAAABABBAABABBBBABABAABB-BAABABBBAAABAABABAABBAAAAAAAA

*000698_00200460 B-BBBB-B--ABBBB--BABBA-ABBBBBAB----A-B--AAABBABBB-BAAABBBABBB-BA-BBBBBABA-A-AABB-BB-ABA--

*000700_00301713 ABABAABAABBBABABBABAAABAA-BBABBBBABAABBB--ABBAAABBAA-BABAABBBBBAAABABBB-A-BAABBB-AABB-BAA

*000701_00080395 BABBBBA-BB-BAB--BAABABBBA-AB-A-B-A-AABBA-AA-AAB-BABABABBBAB--AABB-B-ABBB-AB--B-BBBBAA-AAA

*000701_00098288 BABBBBAABBABABBBBAABABBBA-ABB-BB-AAAABBA-A-BA-BABABABABBB-BBAAABBBBAABBBAABABBABBB-AAAAAA

*000703_00085867 BAABAB-BBABBAABABABBBAAABBBBBBAAABAAAAABBBAABABBABAAABABBBBBABBBB-AA-AABAAABAABBBA-ABAAAA

*000704_00018802 ABABAAABA-BAAABAAAAABA-AAABBBAABBABAAAABAA-BA-ABBA-AAAAABABABBBBABBBAABBAAAB-AABAAABBAAAA

*000704_00065554 ABABAAABA-BAAABAAAAABA-AAABBBAABBABAAAABAABBABABBA-AAAAABABABB-BABBBAABBAAABBAABAAABBAAAA

*000704_00077408 ABABAAABA-BAAABAAAAABA-AAABBBAABBABAAAABAABBABABBA-AAAAABABAB-BBABBBAABBAAABBAABAAABBAAAA

*000704_00079311 ABABAAABA-BAAABAAAAABA-AAABBBAABBABAAAABAABBABABBA-AAAAABABABBBBABBBAABBAAABBAABAAABBAAAA

*000704_00235399 ABABAAABA-BAAABAAAAABA-AAABBBA-BBABAAAABAABBABABBA-AAAAABABABB-BABBBAABBAAABBAABAAABBAAAA

*000705_00348103 AABBBAAA-ABABBBAABABBBBBBBAA-BABABABAABABBBB-AAA-BAA-B-BBABBAAAABBBBAAAB-BAABAABBBBAAABAA

*000706_00028819 ABBBBAABAABAABB-BAAAAAAAABBAAAABAAAAABBABBAAAAABABABBBABBAAA-BAABAABBAAABAABBBBAABBBAAAB-

*000706_00233415 AABBBAABAABAABB-BAAAAAAAABBAAAABAAAAABBABBAAAAABABABBBABBAAA-BAABAABBAAABAABBBBAABBBAAAB-

*000706_00235142 --BBBAABAA-AABB-BAA-AAAAAB-AAAABAAAA-B--BBAAAAA-ABA-BBA-B-AA-BAABAAB-AAABAABBBBAABBBAAAB-

*000708_00125432 B-A-ABBAABBBB--BBB-ABA--BAAA-BBBB-BAABBB-B-BBAAAB-AA-BA--A-BB--AAB-A-B--AAB-BBBBA-ABB-BAA

*000709_00082436 ABBABA-ABAAAABBBBA-ABBA-A-BBBABAAA--AAABBBABB-ABBBBA-ABBAA-B-ABBBB-BABBABBABB-BAABBABABAA

*000709_00141530 ABBABAAABAAAABBBBA-BB-A-A-BBBABAAAAAAAABBBABBBABBBBAAABBAAAB-ABBBB-BABBABBABB-BAABBABAAAA

*000710_00242647 B-BBBBA-ABAABABB-BA--AB-BABBBAB-BB-BABAA-ABBBABABBAAAABAB-BAAAAAAAAAABAABBBAAAA-BBBBBABBB

*000711_00120761 BABABBBBBBBA-ABAB-AAAABB-AAAAB-BBABAABBB-B-AAABBAA-ABABAAAAABAABAAAABAABBBBAA-ABBBBBBB-AA

*000712_00163155 AABABAABAAAAAAAABAABBBA-BABABBBBBBBBABBBABBAA-ABBA-B-AAAAABAAABABABAAABAAABAAABBBAAAAB-BB

*000712_00310453 --BABAABAAAA-AAABAABBBA--ABABBBBBBBBABBBABBAA-ABBABBBAAAAABAAABABABAAAB-AABAAABBBAAAABBB-

*000713_00113058 BABABBAAABBAAABAAABABBABAABBAA-AAAABBABBABBAA-AAAABBBAA-AA-ABBBBAAABABBABABBBABABBBABABBB

*000713_00118116 BABABBAAABBAAABAAABABBABAABBAABAAAABBABBABBAAAAAAABBBAA-AA-ABBBBAAABABBAB-BBBABABBBABABBB

*000714_00150886 BBABABBAABBBBBABBBBAAABAA-BBABBBBBBAABBB-BABBAAABBAA-BABAABBBBBAABBABBB-A-BABBBB-AABBBBAA

*000716_00025418 B-AAA-AAAABA-ABBAABA-A--BBBAAA-BAB-BB--A-BBBB-BAABA-BAAAAAA-BAAB-AABAB-BABBABBA--BBBBAB-B

*000716_00042653 BAAAABAAAABAAA-BAABABAABBBBAAABBABABB-BAABBBB-BAABABBAAAAAAABAABAAABABABABAAB-ABABBB-ABBB

*000718_00114031 BAAAABABAABBBBBBABAA-AAAAABAAABBBBBBBBABBBBAAAAAB-A-BBAAAABABBAB-AABBABBBBBAB-ABBBBABBBBB

*000718_00114779 B-AAABABAABBBBBBABAAAAAAAABAAABBBB-BBBABBBBAAAAAB-ABBBAAAABABBABBAABBABBBBBABBABBBBABBBBB

*000720_00039146 AABABABBABBAAA-BBABABB-ABBBBBA-AAAABBBABAAAAAAAB-AABABAAABABAABBB-BBBAAABBABBBAABBBAAABBB

*000721_00216736 BA-B-BABAB-BBAABBBBBBAAABBAABBAAABAAABABBBBAABAAAA-BABBBBBBAABBBBBAABBBB--BA-BBB-BBBAAABB

*000721_00388515 B--B-BABAB-BBAABBBBBBAAABBAABBAAABA-ABABBB-AA-AAAA--AABBABBAABBBBBAABBBB--BA-B-BABBBA-ABB

*000722_00145494 B-BABBBBBAAB-ABBBAABBBBBABBABABAABAAABAABBAAAAABBAAAAABBBAA-BABAB-AAAABBBABBAAA-BBBAABBAA

*000723_00028716 B-BABBAAAAB-BBABABB-BAAAAAABAB--BA-BBABAAB-AAB-BBBABBAABBBAABABA-AABBABBAA-ABBAA-B-BAAAB-

*000723_00117797 BBB-BBAAAABBBBABABB-BAAAAAABAB-BBABBBABAABAAAB-BBBABBAABBB-ABABABAABBABBAA-ABBAABBBBAAABB

*000724_00111604 BAABA-AB-AB-BBBBBBBBBBAAAA--BB-ABBBBBABBB--BABBAABAB-AABAA-B-ABAA-BBAAAABAB-AB-A-AA-BBBBB

*000724_00235034 B-ABABAB-ABBBBBBBBBBBBAAAABABBBABBBBBABBBBABABBAABAB-AABAAAB-ABAABBBAAAABAB-ABBAAAAABBBBB

*000725_00172634 B-BABBAABBAAAB-BBABBBBBBAABAAABBAB-ABAABBBAAB--ABBBAAAB-BBB-BABABAABAA-BAABBAA-BBBBAABAAA

*000725_00188168 BABBBBAABBAAAB-BBABBBBBBAABAAABBABAABAABBBAABA-ABBBAAABBBBB-BABABAABAABBAABBAABBBBBAABAAA

*000726_00142304 AABBBABABBBBABABBAAABABAAABAABABBBBBBABBAA-BAABBBABABABBABB-BABBBBBAB-AABAAA-BABBBBBAAAAA

*000726_00291562 AABBBABABBBBABABBAAABABAAABAABABBBBBBABB-ABBAABBBABABABBABBBBABBBBBAB-AABAAB-BABBBBBAAAAA

*000727_00156631 ABABAAABA-BAAABAAAAABA-AAABBBAABBABAAAABAA-BABABBA-AAAAABABABBBBABBBAABBAAABBAABAAABBAAAA

*000728_00460670 BABABB-BAABBBABA-BABAAAABBBBBABAAAAAABAABAABBABBBABAAABBBBBBBAAAAB-BB-BAAAABAA-AABBBABAAA

*000729_00137965 BBA-AB-A-ABBABBABABBBBBABAB-AAAABA--BAB-B-ABBB-ABAAABBABBA-AB----AA-AB--A-AABBBAA-BB-AB-A

*000729_00277894 B-AB--B--AB-A-BABABBBBBA-AB-AAAABABBBABABAABBBBABAAAB---BABABB-ABAAB-BBA--AABBBA-BBBB-BAA

*000730_00165903 BBAAABABBAABBBBABBABBBABBAAABAAA-BBAABAAABBBBA-ABA-AAAAAA-B-BABAABABABABBAABAA-BAAAABAAAA

*000730_00172870 BBAA-BABB-ABBBBABB-BBB-BB-A-BA---BBAABAA---BB-AABA-AAAAA-BBB-AB-ABABABABBAAB-ABB--AB-A-AA

*000731_00071525 -ABAB-BBBAAABABAABABABAA-BAAABABBBB--BBBBBAB-ABAAAAABABBABBAABABAAB-BAABBAAAABBBABBBBBBAA

*000731_00073851 -ABAB-BBBAAABABAABABABAA-BAAABABBBB--BBBBBAB-ABAAAAABABBABBAABABAAB-BAABBAAAABBBABBBBBBAA

*000732_00075557 BBAAABAAAABA-BBAA-AABBBABABABABABBB-BAABBBBBBBBBAAAABBBAABBBBABBBA-ABBA--ABAAB-ABBBAABAAA

*000733_00122431 ---B-BABBBAABABBBBBBAAAABABBABA--BBAABBBBABB---BBAABBBABBAA-AAA-AABBABA-BBAB-A-A--AAAB-BB

*000734_00194196 ABABAABBBABABABBBBBAABBBBAABBBBBAAABBBBAAABB-AABAAABBBAABBAABBABAABABBBAB-BABABABAABBABBB

*000734_00201185 A-A-AABBBABABABB-BBAABB-BAABBBB-AAA-B-B-AA-B--ABAAABBBAABBAABBABAABABBBAB-BABABA-A-BBABBB

*000735_00072103 B-ABABABAABBAB-ABAABBABBBBBBBBB-BB-AAAAB-BBBBBABBBBABAAAABABAABBA-BAAA-BAABBBAABBBBAABAAA

*000736_00045658 ABBABABBAA-A-BBAA-ABBBBBBBBABBAABBBAAABBBBBABABBBAABAABABABBAAB-AAB-BAB-AABBABBBAAABBABBB

*000736_00342398 ABBABABBAA-A-BBAA-ABBBBBBBBABBAABBBAAABBBBBABABBBAABAABABABBAAB-AAB-BA--AABBABBBAAABBABBB

*000736_00421639 ABBABABBAA-A-BBAA-ABBBB-BBBABBAABBBAAABBBBBABABBBAABAABABABBAAB-AAB-BA--AABBABBBAAABBABBB

*000742_00007146 A--A-AABBABAABBBBAA-AB--BBABBBBA-BA-ABBAA--BA-AAB-BBBABBBBBA-A-BBBA--B-AABABAAB-BBBBBB--B

*000742_00117661 AB-A-AABBABAABBBBAABABAABBABBBBA-B-AABBAAAA-ABAABBBBBABBBBBABA--BBAAABBAABABAABA-BBBBBABB

*000742_00223922 AB-A-AABBABAABBBBAABABAABBABBBBA-BAAABBAAAA-ABAABBBBBABBBBBABA-BBBAAABBAABABAABABBBBBBABB

*000742_00267422 AB-A-AABBABAABBBBAABABAABBABBBBA-BBAABBAAAA-A-AABBB--AB--BBABA--BBAAABBAABBB-ABABBBBBAABB

*000745_00275882 BABBBBAAAAAB-BAAB-BAA-BB-BBAAA-BAAAABABAAB-AAA-AABAA-BAABAAABBAAABBABABBBAABBBBBA-BA-BAAA

*000745_00356480 BABBBBAAAAAB-BAAB-BAA-BB-BBAAAABAAAABA-A-BAAA--AABAA-BAABAAABBAAABBABABBBAABBBBBABBA-BAAA

*000746_00096220 B-AAAB-B-AABBBAA-BBAA-B--ABAAA--BAB-AA-BBB-BB--AA-AB-B--AAAAB--AAAAB-AAAAAABB-AABB-AAB--A

*000746_00098074 BAAAAB-BBAABBBAA-BBAAABAAABAAAABBABAAABBBBABBA-AAAABAB-AAAAAB-AAA-ABAAAAAAABB-AABBBAABAAA

*000747_00144690 BABABBBAAB-AAAABBA-ABAAABBAA-ABAAAABBBABBA-BAB--BB-AAA-BAA-BBA-ABABBBABABABB--A-ABBBBBAAA

*000747_00269820 BABABBBAAB-AAAABBA-ABAAABABA-ABAAAABBBABBABBA---BB-AAA-BAA-BBA-ABABBBAB-BABB--A-ABBBBBAAA

*000749_00054964 BABBBBBBABBBBAABABBAAABAAAABBBBBABA-ABBBABAABABBBBBBABBBABABABABBBBAAABABBBBBAAABBB-BBBBB

*000749_00118664 B-BB-BBBABBBBAABA-BAAABAAAABBBB-ABAAABBBABAA--BBBBBBABBBABABA-ABBBBAAABAB-BBBAAABB-B-BBBB

*000749_00145121 B-BBBBBBBBBB--ABABBAA-BAAAABBBBBABAAABBBAB-ABABBB-BBA-BBABABABABBBBAA-BABBB-BAAABBB-BB-BB

*000752_00005903 B-ABABBAABBBBBAB-BBABAAAB-AAABB-BB-AABBB-B-BBAAABB-A-BABBABBBBBAABBABBBAAABABB-BAAABB-BAA

*000752_00073091 BBABABBAABBBB-ABBBBABAA-B-AAABBBBBB-ABBBABABBAAABB-A-BABBABBBBBAABBABBB-AABABB-BAAABB-BAA

*000752_00073177 BB-BABBAABB-BBABBBBAB-A-B-AAABB-BBBAABBB-B-BBAAAB-AA-BAB-AB-BBBAABBA-BBBAABABBB-AAABBABAA

*000752_00366829 BBABABBAABBBBBABBBBABAA-B-AAABBBBBBAABBB-BABBAAABB-A-BABBABBBBBAABBABBB-AABABBBBAAABB-BAA

*000752_00366915 BBABABBAABBBBBABBBBABAAAB-AAABBBBBBAABBB-BABBAAABB-ABBABBABBBBBAABBABBB-AABABBB-AAABBBBAA

*000753_00292029 BABBBBAABABBBBBABBAAA-BBBABBBAABAAAABABAABAAAA-AABAA-AAABBAABBAAABBABABBBBABBBBB-BBA-BAAA

*000754_00093011 B-AAABBBBBA-BBAABBBBBAB-AA-BBA--BB-B-A-BBABBAABBAAAA-AAAB-B-ABAAA-B-BBA-AABBAA-AB-A-AA-AA

*000755_00072513 BBABABAB-ABBBBBBABBBBBAAAABABBA-BA-AA-AAB--BABAAABAB-AA-BBABBABABBAAABAABAB-ABBA---AABBBB

*000755_00280845 B-ABABAB-ABBBBBBABBBBBAAAA-ABB-ABAB-AAAABB-B-BA-ABAB-AABBB-B-A-ABBAAAB-AB-B-AB-A---A-BBBB

*000756_00003257 AA-ABABA-BBBABBBAAAAB-BBAA-AABB--ABAAB-BBB-BAAABB-AA-BABBA-BAABAAABBBBAABBB---BA---AB-BAA

*000756_00248631 AA-ABABA-BBBABBBAAAABBB-AABAABBBBABAABABBB-BA-BBBBAA-BABBABBAABAAABBBB-ABBBAA-BA-AAABBBAA

*000756_00253090 --BABABABBBBABBBAAAABBBAAABA-BBBBABAABABBBABA--BBBAA-BABBABBAABAAABBBBAABBBAA-BABAAABBBAA

*000757_00058349 AABABAABAA-BABABAAABBBBBABAB-BAA-B-BBABB-AABBBABABBA-BBABA-ABBBAAAA-B-BAABAB-AB-BBBBABAAA

*000758_00106875 BAABABBBABBBBBBBBBBABBAAAA--AB-ABBBBBABBBBABABBBA-AB-ABBAAAB-ABBA-BBAAABBAAAAABBBAAABBBBB

*000759_00029863 BABABBAABBBAAABBAAAABB-BBAABBABB-BABBBAABABBAABBAAABAABAAABAAABBAAAA-BABABBBBAABAAABB-ABB

*000759_00032436 BABABBAA-BB-A-BBA--A-B-BB-A-BAB--B-BB-AABA-BA-BBAAABAABAAABAAA-BAAAA-BABA-BBBAA--AABBAABB

*000760_00086678 AA-A-A-BABBBABABBABABAABBBBAABAAAAAAA-ABAAABA--B-AAABAABAAAA-BBBBAAABBAABAAAAABAABBBBAAAA

*000760_00108310 AA-A-A-BABBBABABBABABAABBBBAABAAAAAAA-ABAAABABBB-AAABAABAAAA-BBBBAAABBAABAAAAABAAB-BBAAAA

*000761_00006561 ABB-BABAAA-ABBABBBBB-A-ABBBAAB-AA---AAA--B-ABBB---BABABBBA-AB-BB-A----A-A-BAAABAABBAAAB-A

*000761_00080827 ABBABABAAA-ABBAB-BB-AABABBBAAB-AAAAAAAAAABAAB-BAAABABA-BBAAABABBBA-A-AAAAABAAABAABBAAABAA

*000761_00129370 ABBABAB-AA-A-BABBBB--ABABBBA-B-A-AA-AAA-ABAABBBAA-BBBA--B-AABABBB--ABAAAA-B-AA-AABBAAABBB

*000761_00137525 ABBABABAAA-ABBAB-BB-AABABBBAABBAAAA-AAAAABAABBBAAABBBAABBAAABABBBA-A-AAAAABAAABAAB-AAABBB

*000762_00239920 A---BA--A-BB-AAA-BA-ABABAABABBAAB--BBBBAAA-ABBABBABAB-AB-AABAB-B-BB-ABBBBA-ABBABAB--AA-AA

*000768_00134062 B-B-BBAAAABBBBABABB-BAAAAAABABA-AAA-BABA-B-AA--BBBAB-AABBB-ABABABAABBABBAAAABBAABBBBAAABB

*000768_00238130 BBB-BBAAAA-BBBABABB-AAAAAAABABABAAA-BA-AABAAAB-BBBABBAABBB-ABAAABBABBABBAAAABBAAB-BAAAABB

*000769_00246929 BAB-BBBAABBAAAABB-BAABA-BBBABBB-AAABBBBB-A-AABA--BAB-BAAABABA-AB--BBAAB-BBABBBAAB--A--BBB

*000770_00001491 AB--AAABAABAA-BBAA-ABBBBBABAABBBBBAAABAAABBBBBBA--ABABBAAAAAAAB--AABABBBBABA-BBBA-ABBAABB

*000770_00213808 A---A-ABA-B-AAB-AAA--B-BBAB-ABBBBBAAABAAABBBB-B-ABAB-BB-A-AA-A-B--ABABBBB-BABB-BAA-B-A-BB

*000770_00262837 A-AAAAABAABAAABBAA-A-BBBBABAABBBBB-AABAAABBBBBBAABABABBAAAAAAABBBAABABBBBABABBBBAAABBAABB

*000771_00030368 AABAB--BAA-BBAA--BBBBBBBB-B-BABBAB--BBABBB-BB-AA-AAA--ABBAB--ABABABB-BBBBA-BBBBBB-AA-BA--

*000771_00177223 BBABA-BAABBBBBABBBBABAA-B-AAABBBBBBAABBB-BABBAAABB-ABBABBA-BBBBAABBABB--AABABBBBAAABB-BAA

*000773_00173034 BBAAABBAABBBBAABABAAABABBA-BAABB-BABB-BAABBB-B-AAAABAAABBA-ABBABAAAABBABAABBBBBBBAABB-BBB

*000773_00236433 B-AAABBAABBBBAABABAAABABBA-BAABA-BABB-BAABBB-B-AAAABAAABBAAABBABAAAABBABAABBBBBBBAABBBBBB

*000775_00066264 AA-A-ABBABBBA-ABBABABAABBBBBABAAAAAAA-A-AAABABBB-AAABAA-AAAAABBBB-ABBBAABAAAAABA-BBBBAAAA

*000776_00060674 BAAAABA-ABBAAABBBA-AB-BAAABBAABAAAABBBBAAABAAAAAABBAAAABAAAABA-AAABAABBABABBBABABBBBAABAA

*000776_00063208 BAAAABAAABBAAABBBA-AB-BAAABBAABAAA-BBBBAAABAAAAAABBAAAABA-AABAAAAABAABBABABBBABABBBBAABAA

*000776_00236827 BAAAABAAABBAAABBBA-AB-BAAABBAABAAAABBBBAAABAAAAAABBAAAABAAA-BAAAAABAABBABABBBABABBBBAABAA

*000777_00027995 BBAAABABAA-BBB-AAB--BBABB-AABAA--B--ABAAA--B-A--BAAAA-AA--B-B-BA-BABABABBAAAAA-BAAAB-A-AA

*000777_00064137 BBA-AB-BAAABBBBAA-ABBBABBAAABAAAAB--ABAAABBBBA-ABAAAAA-A--B-BABA-BABABA-BAAAAA--AAABBAAAA

*000778_00023376 A-BABA--BBB-ABBB--BABB-BAABAABBBAA--ABA-BB-BAAA-B-A--BAB--BBAABAAABBBAAABBBAA----A-ABBB-A

*000778_00032740 AABABABAB--BABBBAA-ABBBBAABAABB--AAAABABBB-BAA--BBAABBABB-BBAABAAABBBAAABBBAA-BA-A-A-B-AA

*000778_00077631 AABABABABBBBABBBAABABBBBAABAABBBAA-AABABBBABAABBBBAA-BABB-BBAABAAABBBAAABBBAA-BABAAABBBAA

*000778_00137212 A-BABABABBBBABBBAABABBBBAABAABBBAAAAABABBB-BA--BBBAA-BABB-BBAABAAABBBAAABBBAA-BABAAABBBAA

*000778_00159346 AABABABABBBBABBBAABABBBBAABAABBBAAAAABABBBBBAAABBBAA--ABBBBBAABAAABBBA-ABBBAA-BABAAABBBAA

*000778_00185881 AABABABABBBBABBBAABABBBBAABAABBBAAAAABABBBABAA-BBBAA-BABB-BBAABAAABBBAAABBBAA-BABAAABBBAA

*000779_00222763 A-BABAABAAABBAAAABBBBBABBA-ABABBAB-BBBABBBABBAAAAAAABAABBABAAABABABB-A-BBABBBABBBAAAABBAA

*000780_00172447 B---ABABA-ABBBB-ABAB-BABBAAABAAA-B--A-AAAB-BBABAAAA--AAA--B---BAB-ABA-ABBAABAABB-AABBABAA

*000782_00224581 -BAAABAAABABBABBBBABAAABA-BABABBBBBABBBAAAABAAAAABABABABBAAAAAAAABAAABBBABBAABBBBAABBAAAA

*000783_00187502 B-BABBAAABABBABBBBABAAABAA-ABABBBBBAABBAAAABA-AAABA-ABABBAAAAAABA-AAAABAABBAABAA-AABBABAA

*000783_00187588 BB-ABBAAAB-BBABB-BABAAABAA-ABABBBB--ABBA-AABAAA-ABABABAB-A-AAAABABAAAABA-BBAABAABAABB-BAA

*000786_00314595 BAA-ABBB-BBBBBABBBBBAB-AB-BBABA---A-B-BBBB-BABBBBABBAABB-AB-BAA-A-BBA-ABBBAB-ABBAAAAB-BBB

*000786_00314793 B-ABABBBABBBBBABBBBBABAABBB-ABAABB-BBAB--B-BABBBBABBAABBBABBBAAB---B-A--BAABAABBA-AAB-BBB

*000787_00210744 A-A-AABAABB--BABAABA-ABA-ABBABBB-A--ABB--B-B-AAABB-A-BABAABB-B-AAABABBB-A-BAAABBAAABBAB-A

*000788_00177094 AABBBAAABABAB-AAABBBBAB-BBAAAB--AAAAAABBBBABBAAAABAABBBAAAB-A-ABBAABBAABBB-AB-A-BBBAAA-AA

*000788_00195338 A-BBBAAA-ABABBAAABBB-ABBBBAAAB-BAA--AAB-B--BBAA-ABAABBBAAABBA--BBAA-BAABBB-AB-ABBBBAAABAA

*000788_00244388 AABBBAAABABABBAAABBBBABBBBAAAB-BAAAAAABBBBABBAAAABAABBBAAABBA-ABBAABBAABBB-AB-ABBBBAAABAA

*000788_00273976 AABBBAAABABABBAAABBBBABBBBAAAB-BAAAAAABBBBABBAAAABAABBBAAABBA-ABBAABBAABBB-AB-A-BBBA-ABAA

*000789_00241085 BAABABA-BBBBA-BABABAAAABAAABBAAAAAA--ABBBABBA-BBABBAA-ABAABAA-AAAABBAAABAA-BAAABBAAAAABAA

*000790_00225529 BABABBBBBAABABAABABB--BB--B-BAABBABAAAABABAABAAAA-ABBBBABAAAA-BAAAAABAAABAABBBBABBBA-BA--

*000791_00201032 A-AAAABBABAABA-BBBABAAABABBABABABABB-BBAAAABAABAABABABBBBAA-BA-AA-ABABBBBBBABBBBBAAB-BAAA

*000792_00025961 A-BABAAAB-BABBBBABAAABAABAABBBA-BABABBBAA---BB-BAAAB-ABBBABA-BAABBBABBAAABAABABABAA-ABBBB

*000792_00146243 ABBABAAAB-BABBBBABAAABAABAABBBA-BAB-BBBAAAB-BBBBAAAB-ABBBABA-BAABBBABBAAABA-BABABA--ABBBB

*000792_00164651 ABBABAAAB-BABBBBABAAABAABAABBBA-BAB-BBBAAA-BBBBBAAAB-ABBBABA-BAABBBABBAAABA-BABA-AAAABBBB

*000792_00173315 ABBABAAAB-BABBBBABAAABAABAABBBA-BABABBBAAAB-BBBBAAAB-ABBBABA-BAABBBABBAAABA-BABABAA-ABBBB

*000792_00277758 ABBABAAAB-BABBBBABAAABAABAABBBA-BABABBBAAAB-BBBBAAAB-ABBBABA-B-ABBBABBAAABA-BABABAA-ABBBB

*000793_00137658 BAAAABAB-ABAAABBAAAABABBBAABBBBBBBAAABBABBBABBBAAAABABBAAAAAAABB-AABABBBBABABBBBAAABBABBB

*000794_00324354 -ABAB-BBBAA--ABB-AABBBBBA--ABAB---AA-BAA-BAAAAA-BAA---B-BAA-BABABBAAAABBBABBA-A-BBBA---AA

*000794_00344246 BA-ABBB-BAA-AABBBAABBBBBABBABABAAAAAA-AABBAAAAABBAAAAABB-AA--ABABBAAAAB-BABBAAAA-BBAABBAA

*000794_00344332 B--ABBBBBAA-AAB-BAABBBB-A-BABABAAAAAABAABBAAAAA-BAAAAABBBAA-BABAB-AAAABBBAB-AAA-BBBAAB-AA

*000795_00352180 BBAAABBBBBABB-AABB-BB-AB-B--BAAABBAAAA--BA-BAABBAAAABAAA-BBAABAAAB-BBBA-AAB-AABABAABAAAAA

*000796_00139869 BABABBABAABABAABBBA--AA-BAABBABBBBBBABBABABABAABBBABAABAABBAAA-AA-AAABAABABBAAAABAAABABBB

*000800_00014550 AAAAAAAAABBA-ABAA-BABAABBBBAAABBAAABBBBAABBBB-BABBABA-ABAAAABAABAAABABAAABAAA-AABBBBB-BBB

*000802_00077988 ABBBBAAAABBBABBAAAABBBAB-BABBAB-ABABAABBAA-BB-ABABAABABAABABAABBABB--ABBABBB-AA--AAAA-AAA

*000803_00319826 BBABABBAABBBBBABBBBABAA-B-AAABBBBBBAABBB--ABB-AABB-A-BABBABBBBBAABBABBB-AABABBBBAAABB-BAA

*000805_00013959 BAAAABBABBAAAAB-BAABAAAABB-BAAAA-BBAABBBBABBBBABBAABBBABB-BAAAABAAAAAAAABAABBAAAAAAAAAABB

*000805_00041050 BAAAABBABBAAAAB-BAABAAAABB-BAAAA-B--ABBBBABBBBABBAABBBABB-BAAAABAAAAAAAABA-BBAAAAAAAAAABB

*000805_00131985 BAAAABBABBAAAAB-BAABAAAABB-BA-AA-B-AABBBBABBBBABBAABBBABB-B-AAABAAAAAAAABA-BBA-AAAAAAAABB

*000806_00072791 ABBABAA-B-BABBBB-BAAABAABAABBBA-BA-ABBBAAAB-BBBBAA-BAABBBABA-BAABBBABBAAABA-BABABAA-ABBBB

*000806_00260103 A-BABAAABABABBB--BAAABAABAABBBA-BABABBBAAA--BBBB-AABAAB-BABABBAABBB-BBA-AB--BABABAA-ABBBB

*000809_00213381 BABAB-ABAABABAABBBA--AAAB-A-BAB-B-BB-BBABA-ABA-BBBABA-BAABBAAABA--AAABAABABAAA-ABAAA-A-BB

*000809_00233374 BABABBABAABABAABBBA--AAABAABBABBB-BBABBABABAB-ABBBABAABAABBAAABA--AAABAABABAAAAABAAABABBB

*000809_00248300 BABABBABAABABAABBBA--AAABAABBABBB-BBABBABABABAABBBABAABAABBAAABA--AAABAABABAAA-ABAAABABBB

*000809_00248669 BABABBABAABABAA--BA--AAABAABBABBB--BABBABA-ABAABBBABAABAABBAAABA--AAABAABABAAA-ABAAA--B-B

*000810_00234625 ABBABAB-ABABBBABABBABAA-ABBAABBABABAABAB-BABB-ABBAAAABABA-BBBBBA-BAAABBABA-AB-BAAAABB-BAA

*000814_00302684 BA-BABABBBA-BABBBB-BAAAABA-BAB-ABBAAABBBBA-BB-ABB-ABBB-BBAABAAABAAB-ABAABBABAA-AAAAAABB-B

*000814_00433766 BAABABABBBA-BABBBB-BAAAABA-BA-AA-B-AABBBBABB-AABBAABBBABBAABAAABAABBABAABBAB-ABAAAAAAB-BB

*000818_00064608 BBABABBABAB-ABBABABBBBBABAB-AAA-BAB-BABABB-BBBBABAAABBABBABABB-ABAABABBA-AAABBBA-BBBBABAA

*000818_00152337 BBABABBABABBABBABABBBBBABABAAAAABABBBABAB-ABBBBABAAABBABBABA-B-ABAABABBA--AABBBA-BBBBABAA

*000819_00233128 BA-AABBAABABABBBBAABAAAAB-AABAAABBB-BABBB--BABAAAB--BABBBABAAA-BAABBBBBABBBA-BBABAAB-AABB

*000821_00004406 B-ABABABAABBBBBBBBBA-BA-AABA-BBABB-BBABBBBABABBBA-ABBAABAAAB-ABAABBBAA-ABABAABB-AAAABBBBB

*000821_00184705 B--BAB-BAA-BBBBBBBBA--AAAABAAB-ABBBBBABBBB-BAB--ABA--AABAAABAABAABBBA-AABAB-AB-A-AAA-BBBB

*000822_00018070 BBBBBBAABAABB-BBBBA-ABBBAABBAAABAAAABABAABAAAA-BABBBAAAABBAABBAABBBBBABBBBABBBBBABBA-BABB

*000822_00245151 BBBBBBAABAABB-BBBBA-ABBBAABBAAABAAAAB-BA-BAAAA-BABB-AAAABBAABBAABBBBBABBBBABBBBBABBAA-ABB

*000822_00265746 BBBBBBAABAABB-BBBBA-ABBBAABBAAABAAAAB-BAABAAA--BABBBAAAABBAABBAABBBBBABBBBABBBBBABB--BABB

*000823_00019896 BBBABBBBBBAAABABAABBBBABBABABBBAABABBB-BAB--B-AABABA-BBABBBAAABAABBA-B-BAAB-ABB--A-BBAAAA

*000823_00160303 BABABBBBBBAAABABAAABBBABBABABBBAABABBB--AB-ABBAABABA-BBABBBAAABB--BBBBABAABBABB-AAABBAAAA

*000824_00054369 BAA-ABAA-ABAAAAABAABBABBBBAABBAABA-AAA--ABABBAABAAAABBBBAAABAB-BABABBAAABAAABBAABBBAAAAAA

*000824_00080866 BAABABAABABAAAAABAABBABBBBAABBAABABAAA-BABAB-AABAAAABBBBAAABAB-BABABBAAABAAABBAABBBAAAAAA

*000825_00017599 AB-A-AAAAAAABAABBBBABBB-AAAAAABABBBAA-ABBABABB-BBBABAA-BBABBBABABBAABAAABABB-BAABBBABABBB

*000825_00050081 A--A-AAAAAAABAABBBBABBB-AAAAAABABBBAA-ABBABAB-ABBBABAA-BBABBBABABBAABAAABABB-BAABBBABABBB

*000825_00088578 AB-ABAA-AAAABAABBBBABBBAAAAAAABABBBAA--BBABABBBABBABA--BB-BBBAB-BBAABA-ABABB-BAABBBABABBB

*000825_00179017 A-BA-AAA-AAABAABBBBABBB-AAAAAABABB-AA-ABBABABB-ABBABAA-BBABBBA-ABBAABAAABABB-BAABBBABABBB

*000826_00015483 B-ABABBBBABBBBABBBAB--AABBAB--BBAAAAABBB-A-AABBAAAAABB-BABBBBA-AAAABAABABABBB-BAABBAABBAA

*000826_00062035 BBABABBBBABBBBABBBAB--AABBABB-BBAA-AABBB--BAABBAAAAABB-BABBBBA-AAAABAABABABBB-BAABBAABBAA

*000826_00247608 BBABABBBBABBBBABBBAB--AABBABB-BBAAAAABBB-ABAABBAAAAABB-BABBBBA-AAAABAABABABBB-BAABBAABBAA

*000827_00006700 BAAAAB-BBABBBBAA-BBAAABAAABAAAABBABBBABBBBA-BA-AAA--AB-BABBAA-AAA-ABAAAAAAABB-AABBBBBAAAA

*000827_00214723 B-AAAB-BBABBBBAA-BBAA-BAA-BAAAABBABBBABB-BA-BA-AAA-BAB-BABAAB-AAA-ABAAAAAAABB--A-BBBBAA-A

*000828_00256283 BA-BABABBBA-BABAB-BBAAAABA-BBAAAAB-AABBBBA-B--ABBAABBBABB-ABAAABAABBABAABBAB-ABAAAAAABABB

*000828_00266217 B-ABABABBB--BABABBBBAAAA-A-BBBAA-B-AAB-BB--BAA--BA--BBABBAABAA-BA-B-A--A-BAB-ABAA--A-BA-B

*000830_00175710 AAAAA-A-BAAABBABBBBBBBBBB-BBBABA-A-BBBABAB-A-B-ABB-BBBABBBBAABABBBBBBB-ABA-AAA-AAB-AAAABB

*000831_00099942 AAABAAABAAABBBBAABBABABABBBBABBBAABBBA-ABBB-BBBA-BBA-AABABBAABBABBAB-A-BABB-AAABBAAA-AAAA

*000831_00100028 AAABAAABAAABB-BAABBABABABBBBABBBAABBBA-AB-B-B-BA-BBA-AABABBAABBABBAB-A-BABBBAAABBAAAAAAAA

*000832_00025228 AB-A-AABBABAABBBBAABABBABB-BBBBB-B-AABBAAAA-AAAABBBBBABBB-BABA-BBBAAABBAABBBAABABBBBB-ABB

*000832_00150109 AB-A-AABBABAABBBBAABABBABB-BBBBB-B-AABBAAA--AAAABBBBBABBBBBABABBBBAAABBAABBBAABABBBBB-ABB

*000835_00004304 ABAAAAABAABABABBABAAAABAAABAAA-BBBBBBBABBB-AABAAA-ABBBAAAABAABABBAAABAABBABBBBABABBBBABBB

*000837_00085588 BAABAB-BBABBAABABABBBAAABBBBBBAAABAAAAABBBAABABBABAAABAB-BBBABBBBBAA-AABAAABAABBBAAABAAAA

*000838_00199097 B--BBBAAAABBABBA-AAABBABBABBBA-BABA-ABAB-A-BABBBABABABBAAABBBBBBAA-ABB-BBA--A----B-B--ABB

*000840_00013632 B--BBBAA-BABABABBA-BABBBA-A-BAB-A--AABB-AA--A--A-ABA--BBBABB--ABBBBAABBBAABABB-B-BBAA-AAA

*000840_00185860 BABBBBAABBABABABBAABABBBABAABABB-AAAABBAAA--AABABABABABBBABBBAABBBB-ABBBAABABBABBBBAAAAAA

*000840_00195807 B-BBB-AABBABABABBAAB-BBBA-AABAB--B--ABBAAAA-AABABAB--AB-BABBBAABBBBAA---AABABBABBBB-AAAAA

*000840_00212039 BABBBBAABBABA-ABBAAB-BBBA-AABABB--AAABBAAA--AABABAB-BABBBABBBAABBBBAABBBAABABBABBBBAAAAAA

*000841_00217699 BBBBBBAABA-BB-BBBBAAABBBBABBBAABAAAAB-BAABAAAA-AABBB-AAABBAABBAABBBBBABBBBABBBBBABBA-BABB

*000842_00300005 AAAAAAABAABAAABBAAAABABBBABAABBBBBAAABAABBBABBBAAAABABBAAAAAAABB-AABABBBBABABBBBAAABBABBB

*000842_00322112 AAAAAAABAABAAABBAAAABABBBABAABBBBBAAABAABBBABBBAAAABABBAAAAAAABB-AABABBBBABABB-BAAABBABBB

*000843_00129378 AABABAABAAABBBAAAB-BBBBBAABAAABBABABBBABBBAAAAAAAAAABBABBABAAABAB-BB-BBB-ABBABB-BAAABBAAA

*000844_00018576 BABABBBBBAABAABBBAABBBBBABBABABAA-AAABAABBAAAAABBAAA-ABBBAA-BABABBAAAABBBABBAAA-BBBAABBAA

*000844_00099845 --BABBBBBAABAABBBAABBB-BABBA-ABA-AAA-BAABBAAA-ABBAAAAABBBAA-B-BABB----BBBABBAAAABBBA-BBAA

*000845_00068952 AB-AB-AA-AABABAB-AAA-BAAA-BBBA--AA-BBAA-B--BBBAB-BB---BBAAABBAB-ABBBABBAB-ABBABAA-BBB--AA

*000845_00275651 A-BABAAA-AA-ABABB-B-BBAAAA-BBA--AAABBAABBB-BB-A-BB-AAABBAAAB-ABBABBBABBABBABBABA-BBBBABAA

*000846_00155173 ABB-B-BAABB-BABBBBBAA-BB--AABBB--AA-BBBBAA-AABA-AAABAAAABBAB-BABBABAABBABBAABBBAB-A-BBBBB

*000846_00170972 ABB-BABA-BBABABBBBB-ABBBAAAABBBBAA-BBBB---AAABA---ABA---BB-B-BAB-ABA-BB-BB--BBBAB---B-BBB

*000848_00059859 BBBABBAAAAAABBBBABBBB-A-A-BBBABAAAAAAAAABAAAB-BBBB-AAABBAAABBABBBB-BABBABB-BB-BBABBABAAAA

*000848_00157556 BBBABBAAAAAAABBBAABBB-A-A-BBBABAAAAAAAA-BAAAB-BBBBBAAABBAAABBABBBB-BABBABB-BB-BBABBABAAAA

*000850_00015168 B-BABBBAAB-AAAABBAAA-AAAB-AA-ABA-BBB-BABABABABAAABAAAAABBAABBA-ABA-B-BB-BBBB--B-ABBBBBAAA

*000850_00061863 BABABBBAAB-AAAABBAAA-AAAB-AA-ABABBBB-BABABABABA-ABAAAA-BBAABBA-ABA-BBBB-BBBB--B-ABBBBBAAA

*000850_00170907 BABAB-BAAB-AAAABBAAA-AAAB-AA-ABA-BBB-BABABABABAAABAAAA-BBAABBA-ABA-BBBB-BBBB--BAABBBBBAAA

*000851_00243465 --AAAABBBBBABBBAABBAAA-BBB-AAA-B-ABAAAABAABBBBBA-BAABAAAB-BA-BABBA-AABAAAA-ABAAAABBAABBAA

*000851_00281994 A-AAAABB-BBABBB-ABBAAA-B-B-AAAB--A-AAAA-AA-B-BBA-BAAA-AA--BAB-AB-A-A--AAAA-ABA-AABBAABBAA

*000854_00179912 AAABAAABABBAAAABBAAABB--ABABBABAA-AAA-AABA-AB-ABBAABAAABABAAAA-BBBBBBAAABAAB-BAABBBAAABBB

*000854_00181988 AAABAAABABBAAAABBAAABB--ABABBABAA--AA-AAB-AABBABBAABAAABABAAAABBBBBBBAAABAAB-BAABBBAAABBB

*000854_00183348 AAABAAABABBAAAABBAAABB--ABABBABAA-AAA-AABAAAB-ABBAABAAABABAAAABBBBBBBAAABAAB-BAABBBAAA-BB

*000854_00183434 AAAB-AABABBA-AABB-AA-BB-A--BBAB-A-AAABAAB-AABBA----BAAABABAAAABBBBBBB--ABAAB-B--BBB-AABBB

*000855_00015405 BABBBB-AAAABB-BAABABBAAABBBBBABAAAAAABAAAAABBABBBABAAABB-A-B-ABAAB-BBBBBAAABAABBABB-ABAAA

*000855_00091215 -ABBBB-AAAABB-BAABABBAAABBBBBABAAAAAABAAAAABB-BBBABAAABB-A-BBABAAB-BBBBBAAABAABBABB-ABAAA

*000855_00106858 BAB-BB-AAAABBBBAABABBAAABBBBBAB-AAAAABAAAAABBABBBABAAA-B-A-BBABAABABBBBBAAABAABBAB--ABAAA

*000855_00106944 BABBBB-AAAABB-BAABABBAAABBBBBABAAAAAABAAAAABBABBBABAAABB-A-BBABAAB-BBBBBAAABAABBABB-ABAAA

*000855_00113818 BABB--AAAAA---BAABA-BAAAB-BB-A-A-A--AB-AAA--BABBBABAAABB-A-BBABAAB-B-B-BA-ABAABBA-B-ABAAA

*000855_00144833 -A-BBB-AAAABB-BAABABBAAABBBBBABAAAAAABAAAA-BBABBBABAAABB-A-BBABAAB-BBBBBAAABAABBABB-ABAAA

*000855_00202154 BABBBB-AAA-BB-BAABABBAAABBBBBABAAAAAABAAAAABB-BBBABAAABB-A-BBABAAB-BBBBBAAABAABBABB-ABAAA

*000856_00136169 BABABBAA-BBAAABBAAA-BBB-BAABBBB-BBAAABBABB-BB-BAAAABABBAAAAAAABBAAAAABABBABABABBAAABBAB-A

*000857_00076616 B-AA-BBBAABABBBBABBABBBABABABBBAAAABBAAA-BABB-BABAABBABBABB-AABABABABBBBBABBABB-AB-AABABB

*000857_00096166 B-AA-BBBAABABBBBABBABBBABABABBBAAAABBAAABBABB--ABAABBABBABBBAABABABABBBBBABBABB-ABBAABABB

*000857_00096252 BB-A-BBBAABABBBBABBABBBABABABBBAAAABBAAABBABBABABAABBABBABBBAABABABABBBBBABBABB-ABBAABABB

*000859_00055016 BABBBBAAAAAB-AAAB--BA-BB-BBAAAABBABBBABA-BA-AAAAABAA-BAABAAAABAAAABABAABBAABBBBBABBA-BAAA

*000859_00133680 BBAAABBAABBBBABBABAAABABBABBAABA-B-BBABA-BBABBB-A-AB-BAB-A-ABBABAABBBBABAABBBBBBBAABB-BBB

*000860_00020320 BBABABBAABBBBBABBBBABAA-BBAAABBABB-AAB-B-BABBAAABB-ABBABBABBBBBAABAABBBAAABABBBBAAABB-BAA

*000861_00133434 B-BABBBAAB-BAAABBA-AB-B-BABA-ABABA-BBB-BBABBAB-ABBBAAA-BAAAB-A-A--BBBAB--ABBBB-AABBB-AAAA

*000861_00150312 BABABBBAAB-BAAABBA-ABABABABA-ABABABBBB-BBA-BAB--BB-AAA-BAAABBA-AB-BBBAB-AABB-BA-ABBB-AAAA

*000862_00256121 A-BBBAABAAB--BB-BAAAABAAABBA-AABAA--ABBABBAAAAA---ABB-A--A-AABAABAAB-AAA-A-BB-BA---B-AA--

*000863_00026011 BA-AABBABBAAB--A-BBBBB-B-ABBBAABBBB-BABB-AAA---ABB-ABAABBABBB-BAAAABBB-ABBABBBBA-B--A--AA

*000863_00090899 BAAAABBABBAABBAAABBBBBBBBABABA-BBBBBBAB-BAA-BBAABBAABAABBABBBBBAAAABBBAABBABBBBAABBBBBAAA

*000866_00056381 BABBBBAAAAAB-BAAB-BAA-BB-BBAAAABAAAABABAABAAAA-AA-AA-BAABAAABBAAABBABABBBAABBBBBABBA-BAAA

*000866_00060273 BABBBBAAAAAB-BAAB-BAA-B--BBAAAABAAAABABA-BAAAA-AABAA-BAABAAABBAAABBABABBBAABBBBBABBA-BAAA

*000866_00060359 BABBBBAAAAAB-BAAB-BAA-BB-BBAAAABAAAABABAAB-AAA-AABAABBAABAAABBAAABBABABBBAABBBBBABBA-BAAA

*000866_00168643 BABBBBAAAAAB-BAAB-BAA-BB-BBAAAABAAAABABAABAAAA-AABAA-BAABAAABBAAABBABABBBAABBBBBABBA-BAAA

*000866_00181765 BABBBBAAAAAB-BAABABAA-BB-BBAAAABAAAABABAABAA-A-AABAA-BAABAAABBAAABBABABBBAABBBBB--BAABAAA

*000871_00189961 B-AAABAAAAA---BAAAAABBAA--BBBAA--B--A---BABB-B-BAAAAA--AABBBB-BBBA-ABAABAAABABABBB-AAB-BB

*000873_00106935 ABBABAB-ABBABABBBBBAABBBAAAABBBBAAABBBBAAAAAABABAAABAAAABBAB-BABBABAABBABBBABBAABAAABBBBB

*000873_00237177 ABBABAB-ABBABABB--BA-BBBA-AABBB-AAA-BBB-AA-AABABAAABA-AABBABABA-BABAABBABBB-BB-ABAAA-BBBB

*000875_00180055 B-ABAB-BBAB-AABABABBBAAABBBBBB-AA-AAAAABBB---BBAABAAABABBBBB-B-BBBAA-AABA-A-AA-B-A---AA-A

*000876_00016216 A-AAAABBBBBA-B-BA-AAAAABAB-AAABBAAA-AB-BBABABBBAABAAABBAB-BABBAABABA-BAAAABBBABABBBAABAAA

*000877_00010305 ABBBBAAAABBBABBABAA-BBAB-BABAABA-BABAABBAABBBBABAAAABAAAABABABBBABBAAABBABB-A-AB-AAAABAAA

*000877_00047124 ABBBBAAAABBBABBABAA-BBAB-BABAABA-BABAABBAABBBBABAAAABAAAABABABBBABBAAABBABBBA-ABBAAAABAAA

*000877_00047408 A-BBBAAAABBBABBABA--BBAB-BABAABA-BABAABBAABBBBABAAAABAAAABABABBBABBAAABBABBBA-ABBAAAABAAA

*000877_00095921 ABBBB-AAAB-BA-BAB-A-B-A-AB-BAA-A-BABAA-BAA-BBBABA-AABAAAABABABBBABBAAABBABBB--ABBAAAAB-AA

*000877_00253837 ABBBBAAAABBBABBAAAA-BBAB-BABAABA--ABAA-BAABBBBABABAABAAAABABABBBABBAAABBABBBA-ABBAAAA-AAA

*000878_00091865 AABBBAAABABABBAAABBBBABBBBAAAB-BAAA-AABBBBABBAAAABAABBBAAABBA-ABBAABBAABBB--A--B-BBA-ABAA

*000878_00100287 AABBBAAABABABBAAABBBBABBBBAAAB-BAAAAAABBBB-BBAAAABAABBBAAABBA-ABBAABBAABBB-AA-ABBBBAAABAA

*000881_00159631 AAAAAA--BAAABBA-BBBBBBBBB-BBBABA-ABBBBABAB-A-BAABBBBBBABBBBAABABBBBBBBBABA-AAABAABBAAA-BB

*000883_00174490 BABABBBAABBABAABBBBAABAABABABBB-AAABBBBBAAAAABABABABABAAABAB-BABBAB-AABBBBABBBAABBB-BABBB

*000884_00083733 A-AAAAABAABAAABBAAAABABBBABAABBBBBAAABAABBBBBBBAABABABBAAAAAAABB-AABABBBBABABBBBAAABBABBB

*000885_00102261 B-BA-BAB-BAAA-BAAABAB-ABAABBAAB-AAABB-BBABAA-AAABABBBAA-AAAAABBBAAABABAABABABABABBBABA-BB

*000885_00185696 BABAB-ABABAAABBAAABABBABAABBAABAAAABBABB-BAAAAAABABBBAA-AAAAABBBAAABABAABABABA-ABBBABA-BB

*000886_00240944 BABBBBAA-AAB-AAAB--BA-BB-BBAAAABBABBBABAABA-BAAAABAA-BAABAAAA-AAAABABAABBAABBBBBABBA-BAAA

*000887_00001560 BBABABBBABABABBBBAB-AAAABABAABABABAAA-AAABBBAAABAA-BABBBBBBABBA-ABAAABBBBABAABABAAAA-B-BB

*000887_00103464 BBAB-BB--BABAB-BB-BBAAAABABAABABAB--A-A-ABBBAAAB---BABBBBB--BBABAB---B-BBABAABAB-AAA-BBBB

*000889_00160602 --AAABAAAABBAB-AAAAA--AA-ABBBAA-BBBAAAB-B-BBABBBAAA-A--AABBBB-BBB-BABBAAAAAAAB--B-B--B---

*000892_00155186 BB-B-BBBAB-AAA-A-ABBAB-AABBAABABAAABBBAAAB-AA-BABAAA-ABABABAAA-AAB-BB-AA-BA-AABAAAA---AAA

*000893_00105976 B--AAB-ABAAABBA-BBABB-B-B-BBBAAAAAABBBABAB-A--AA-BBBBB-B--BAABA-B-ABBBBABA-AAA-AABBB-A-B-

*000898_00107482 BAABABAABABAABAABAA-BABBBBAABBAABABAAA-BA-ABBAABAAAAB-BB-ABBAB-BBAABBAABBAAABBABBBBAAA-AA

*000899_00082730 BBAAABBAB-ABBAAABBBBBAABAAABBAABBBAAAA-A-ABBAABBAAAABAAAA-B-ABAAAB-BBBABAABBAABBBAA-AAAAA

*000899_00083773 B-AAABBAB-ABBAAABBB-BAABAAABBAABB-AAAA-ABABBAABBAAAABAAAABB-ABAAAB-BBBABAABBAA-BBA--AAAA-

*000900_00133924 BBABABBABB-BBBBABBBBBBBBBAB-AAABBA-BBABABAABBBBABAAABBABBABABBAABAABABBAA-ABBBBA-BBBBBAAA

*000901_00266636 BABBBBAABA-BB-BABBAAA-BBBABBBAABAAAABABAABAAAA-AABAA-AAABBAABBAAABBABABBBBABBBBBA-BA-BAAA

*000906_00032862 ABABAAABABBBABBAAA-BB-AB-BABBABBABAAAABBA-BBBAABABAABAB-ABABAABBABBBBABBABBB-A-BBBBA-AAAA

*000910_00004794 B-ABABABABBBBABBBBBABBAAAAB-AB-ABBBBBABBBBABABBBA-AB-ABBAAAB-ABBA-BBAAAABAAAA-BAAAAABBBBB

*000910_00141953 B-ABABABAABBBABBBBBABBAAAABAAB-ABBBBBABBBBABABBBA-AB-ABBAAAB-ABBA-BBAAAABAAAA-BAAAAABBBBB

*000910_00175570 B-A-ABABAABBBAB-BBB--BAA-A-AABB-BBBB-AB-B--BABBBA-A-B-BBAAAB-ABBA-BBAA-ABAA-A-BA-AA-BB-BB

*000912_00217577 BBBBBBBBABABBBABBBBABAA-ABBAABBABBBAABBBBBABBAA-BB-A-BABBABBBBBAABAABBBAAABABBBAAAABB-BAA

*000913_00170831 BBBABBAB-AAABB-ABBBABBBB-A-BAABBBBBABABBABAB-AAABBAABBBBBBBABAB-BAABABABAABAAABBB--BBBAAA

*000915_00048163 A-ABAAAB-BBAAAABBAAABB--BBABBABAA-A-A-ABBAAABBABBAABAAABABAAAABBBBBBBAAABBABBBA-BBBAAABBB

*000918_00052581 BBBBBBAAAABBBBABABB-BAAAAAA-BB-BBABBBABAABAAABABBBAABAABBA-ABBBABAABBABBAA-ABBAABBBBAAAAA

*000918_00242976 BBBBBBAAAABBBBABABB-BAAAAAA-BB-ABABBBABAABAAAB-BBBAABAABBA-ABBBABAABBABBAA--BBAABBBBAAAAA

*000920_00197928 AABBBAABAAABBBAABB-BBBBBAABAAABBABABBBABBBAAAAAABAAA-B-BBABAAABABBBB-BAB-ABBABBBBAABBBAAA

*000921_00040828 BBAAABBBAAAAAAABAABB-AABBABABBBAABABBB-BABBABBABAABABBBABBABBAAB--ABBB-BAABBABBB-AABBAAAA

*000921_00210124 B---ABBBBAAAAAABAABBB-A-BABA-BBAABA-BBABABBABBABAABABBBA-BABBAAB--BBBB-BA-BBABB---AB-AAAA

*000923_00124960 ABA-AABABB-ABBAAABBBBBBBBAB-BAABBBBBBAB-BAABBBBABAAABAABBABABB-ABAABBBBAABABBBBAABBBBBAAA

*000923_00134938 ABA-AABABB-ABBAAABBBBBBBBAB-BAABBBB-BA--BAABB-BAB-AABAABBABABB-ABAABBBBAABABBBBAABBBBBAAA

*000927_00015097 BABABB-BBBABAABBBAABBBBBABBABABAA-AAABAABBAAAAABBAAAAABBBAA-BABABBAAAABBBABBAAA-BBBAABBAA

*000927_00052536 B-BAB-BBBBABAABBBAABBBBBABBABAB-A-AAABAABBAAAAA-BAAAAABBBAA-BABA-BAAAABBBABBAAA-BBBAABBAA

*000927_00070387 BABABBBBBBABAABBBAABBBBBABBABABAA-AAABAABBAAAAABBAAAAABBBAA-BABABBAAAABBBABBAAA-BBBAABBAA

*000928_00034402 BBBABBAAAAABBBBBABBBB-AAABBBBABAAAAAAAAABAAABBBBBB-ABABBAA-BBABBBBABABBABB-BB-BBABBABAAAA

*000929_00043213 B-BABBBBABB--ABAB-AAAABB-A-AABBBB-BAAB-BAB-AAABBAA-ABABA-AAABAABAAAAB-ABBBBA--AB-BBBBBA-A

*000930_00122931 -BBBB-BBBAAABABAABABABAABBAAABABBBB--BBBBBAB-AAAAABABABAABAAABABAAB-BAABBAAAAABBBBBBBBBAA

*000930_00176887 -BBBB--BBAAABABAABABABAABBAAABABBBBB-BBBBBAB-AAAAABAB-BAABAAABABAAB-BAABBAA-AABBBBBBBBBAA

*000932_00345916 BABABB-BAABBBABA-BABAAAABBBBBABAAAAAABAABAABBABBBABAAABBBBBBBAAAAB-BB-BAAAABAA-AABBBABAAA

*000933_00012027 BABBBBAAABAABABBBBA--ABBBABBBABBBBBBABAA-ABB-AB-BBAAAABABBBAAAAAA-AAABAABB-AAA-ABB--BABBB

*000933_00131858 B-B-BBA-ABAABABBABA-BA--BABBBA-B-BB-ABAA-ABBBABABB-AAABAB--AAAAAAAAAABAABBBAAAAABBB-BABBB

*000935_00251371 B-BBBBAAAABBBBABABBABAAAAAA-BBBABABBBABAABAAAB-BBBAAAAABBA-ABBBABAABBABBAABABBAABBBBAAAAA

*000935_00258204 B-BBBBAAAABBBBABABB-BAAAAAA-BBBABABBBABAABAAAB-BBBAAAAABBA-ABBBABAABBABBAABABB-ABBBB-AAAA

*000936_00131342 BAB-BBAAAAAB--AA---BA-BB-AB-AAABBABBBABAABA-BA--ABA------AAAA--AAA-ABAABBAABBB-BABBAABA-A

*000937_00017677 BBBABBAAABABA-BBB-ABAAABB-A-BAABAB-BBBBBBAABABA-ABA-ABAB-ABA-AAAAAAAAABA-ABABBAABAAAAAA-A

*000937_00141409 BBBABBAAABABAABBBAABAAABBA-ABAABABBBBBBBBAABABA-ABA-A-ABBABAAAAAAAAAAABAAABABBAABAAAAAAAA

*000937_00387535 -B-ABBAAABABAABBBAABAAABBA-ABAABABBBBBBBBAA-ABAA-BA-ABABBABAAAAAAAAAAABAAAB-BBAAB---AAAAA

*000938_00275578 B-ABABABAABBBBBBBBBABBAAAABAABBABBBBBABBBB-BABBBABAB-AABAAAB-ABAABBBAAAABAB-ABBAAAAABBBBB

*000940_00071464 ABBABABBAABABABBBBBAABBBBAAABBBBAAA-BBBAAA--A-ABAAABBBA-BAAABBABBABABBBAB-BABBBA-AABBABBB

*000943_00008597 BAAAABABBABAAABBAAAABBBBBAABBBBBBBAAABBABBBBB-BAAAABABBAAAAAAABB-AAAABABBABABABBAAABBAB-A

*000948_00064647 BBAAABAABAABBBBBBBABBBAAAAAABBABABBAABBABB-AAAAB-A-AAAAAAAAABAABBBABABABAAAABAABBBBABABAA

*000949_00155785 AAABAAAABABAABAABAA-BABBBBAABBAABABAAABBABABBAABAAAABBBBAABBAB-BBAABBAABBAAAA-ABBBBAAA-AA

*000954_00114188 BAABABAABBABA-BBBABAABAAABABBBAAAAABBABBBBBAAABBABBAAAABAABAAAAAAABBBAABAA-BAAABBAAAAABAA

*000958_00104289 AABABABA-BBBA-BAAABAB-BBAA-AABB-AAAAABA-BB-BAA-BBBAABBABB-BBAABAAABBBAAABBBAA-B--AAABBBAA

*000961_00065807 B-BBBBBB-BBAABBABAAA-ABAA-BAAB--AAABBBAAAB--AB--BBB-B-BABABBAAAAAABBBBBBABBAAB-BBAA--B---

*000962_00156513 B-AAABBAABBBBAA-ABAAAAA--ABBAABBBBBBB-B-AB-B---AAAAB-AA-BAAABB-BABABBBAABBBBBB-ABAABB--BB

*000963_00079639 BAABABAABABAABAAB-A-BABBBBAABBAABABAAA-BAB-BBAABAAAABBBBAABBAB-BBAABBAABBAAABBABBBBAAABAA

*000963_00120917 B-A-ABAABABAABA-BAA-BABBBBAABBAABABAAA--AB-B-AA-AAAABBBBAABBABABBAABBAABBAAAABA-BBBAAA-AA

*000963_00193653 BAABABAABABAABAABAA-BABBBBAABBAABABAAA-BAB-BBAABAAAABBBBAABBAB-BBAABBAABBAAAABABBBBAAA-AA

*000963_00207357 BAABABAABABAABAABAA-BABBBBAABBAABABAAA-BABABBAABAAAABBBBAABBAB-BBAABBAABBAAAABABBBBAAA-AA

*000966_00035276 AAABAAABA-BBB--BBBBA-BAB-BBBABA-BAB-BBBBAB--B--AA-AAB-ABABBAAB-AB-BBBABABB-AAAA-BA--BAAA-

*000966_00085063 A-A-AAABA-BBB-ABBBBABBABABBBABABBABBBBBBABB-BB-AA-AABAABABBAABBAB-BBBA-ABBAAAAAABAAABAAAA

*000969_00157255 BAABABAABBA-BABABBBAAAAABAABB-AB-B-AABBBBABB-AABBAABBBABBAABAAABAABBBBAABBAB-ABAAAAAAB-BB

*000970_00071715 ABAAAAABAABAAABBAAAABBBBBABAABB-BBA-ABAAA-BBBB-AABABA-B-AAAAAABB-AABABBBBA-AB-BBAAABBAABB

*000970_00214048 ABAAAAABAABAAABBAAAABBBBBABAABBBBBAA-BAAABBBBBBAABABABBAAAAAAABB-AABABBBBA-ABBBBAA-BBAABB

*000976_00037188 BAA-ABABAABBB-B-B-BA-BAAA-B-ABBABBB-BABBBBABA-BB--ABA-ABAAABA-BA--BBAAAABABBAB-A-AA---BBB

*000976_00185499 B-AB-B-BAABBBBBBBBBABBAAAABAABBAB--BBAB-BBABA-BBABAB-AABAAAB-ABAABBBAAAABA--A---AAAABBBB-

*000977_00210752 AABBBAAA-ABABBBAABABBBBBBBAA-BABABABAABABBBB-AAA-BAA-B-BBABBAAAABBBBAAABBBAAB-ABBBBAAABAA

*000983_00069609 BBBBBBBBABABAAABBABBAAAABABAABABABAAA---ABBBA-AAAA-BABBBB-BAABA-ABAAABBBBABAABABAAAB-BBBB

*000983_00098367 BBBBBBBBABABAAABBABBAAAABABAABABABAAA-A-ABBBA-AAAA-BABBBB-BAABA-ABAAABBBBABAABABAAAB-BBBB

*000984_00080072 AA-BAABAAABBABBBBAA-BABBAABABBBAABAB-AAABBBAAA-BABAABAABAAABBABAAB-AABBABABABABABBBBBBBAA

*000984_00215261 AA-B-ABAAABBABBBBAA-BABBAABABBBAABAB-AAABBBABABBABAABAABAAABBABAAB-AABBABABABABABBBBBBBAA

*000986_00169413 B-AAABBBBBA-AA-ABABBBA-BA--BBA--BB--AA-B-A-AAABBA-AA-AABABB-AB-AABBBBBAA-A-BAAB--AABAAAAA

*000986_00171288 BBAAABBBBBABA-AABABBBA-BA-ABBAAABBA--A-BBABAAABBAAAABAABABB-ABAAABBBBBAAAABBAABABAA-AAAAA

*000988_00281375 A-AAAABBAABABBAA-BA--A-ABB-A-A-A--BAAAAAABA----B-BBAB-AABAAABBBBBABAAAAABA-AAAAAABBAABBAA

*000990_00185494 ABABAABBA-ABBAAAABAA-BABAABABB-ABBBBBBAAAA--BBABBA-ABAA-BAABBAABBBB-B-BBBABAAAA-ABBA-B-AA

*000992_00266994 BAB-BBBAABBABAABBBBAABAAAABABBBBAAABB-BBAAA-ABA-ABABAAAABBAB-BABBAB-AABABBABBBAABBB-BABBB

*000998_00054056 B-BA-BBBBA-ABAB-B-AAABBAB-AAABBBBA-AABBB-BBAAABBAA-ABABAAAAAB-A-AAA-BAABBBBAAAABBBBBB-BA-

*001000_00213717 AAABAAABAAABB-BAABBABABABBBBABBBAABBBA--B-B-BBBABBBA--ABABBAABBABBAB-A-BABBBAAABBAAAA-AAA

*001000_00295361 AAABAAABAAABB-BAABBABABABBBBABBBAABBBA--B-B-B-BA-BBABAABABBAABBABBABAA--ABBBAAABBAAAAAAAA

*001001_00178483 B-AAABABABAABABAABAAAABAA-BBABBAABABBAABABABABBBAAAAABAABAAAAAABB-AABBAABABBABAA-AA-AAAAA

*001001_00304860 B-AAABABABAABABAABAAAABAA-BBABBAABABBAABABABA-BBAAAAABAABAAAAAABB-AA-BAABAB-ABAABAAAAAAAA

*001002_00229563 BBABABABAABBBBBBBB---BAAAA-AABBA-BB-BABBBBABAB-BA-ABAAA-AAAB-A-AABBBAAAA--B-AB-A-AAAB-BBB

*001002_00242869 B-ABABABAABBBBBBBBBABBAAAABAABBABBBBBABBBBABA-BBA-AB-AABAAAB-ABAABBBAAAABABBABBAAAAABBBBB

*001003_00283375 BAABABABAABBABBABA-BBABABBAAB-BABBBAAAABABBABBABBBBABAAAABBBAABBA-BBAABBAABBBAABBAAAAAAAA

*001003_00285206 BAABABABAA-BABBABABBBABABBAABB-ABBBAAAABA--ABBABB-BABAAAABB-AABBA-B-AA-BAABBBA-BBA-AAAAAA

*001003_00379467 B-ABABABAABBABBABA-BBABABBAABBBABBBAAAABAB-ABBABBBBABAAAABBBAABBA-BBAA-BAABBBAABBAAAAAAAA

*001003_00380598 BAAB--AB-AB--BBABA-B-ABA-BAABBB-BBBAAAABAB-ABBABBBB-BAAAABBBAABBA-BBAA-BAABBBAABBAA-AAAAA

*001003_00388123 B-A-AB---ABBA-B-BAABBABABBAABBBABBB-AAABA-BAB-ABBBBA-AAA-B-BAABBABBBA---AA--BAABB-AAAAA-A

*001007_00080945 BBAAABBBBBABA-AABABBBA-BA-ABBAAA-B---A-BBABBAABBAAAABAAA-BB-ABAAABBBBBA-AABBAABABAA-AAAAA

*001008_00041557 BBB-BBBABAABABAAA--BA-B--BBAAAABBA-BBABAABA-B-AAA-AABBAABAAAA-AAAABA-A-BB-ABBBB-AB-ABB--A

*001008_00245483 B-BB-BAABAABBBAAA-AB--BB--BAAAABBA-BBAB-ABA-B-AA-B-AA-AA-AAAABAAAAB-B-A-BBABBBBBAB--ABAAA

*001009_00195340 BBAAA-BBAABABBBBABAABBBABABABBBAABA-BAABBBBBB-BAAAAABABAABBBBA-B-A-ABBB-BABAAAB-ABBAA-AAA

*001010_00209395 BBAAABABBBBA-BBAA-ABBBBBABBABBAABBBAAA-ABBAABABBBABAAABABAABAAB-B-BBB-AAAABBABBAAAABBABAA

*001011_00059561 BABBBB-B-ABBAABABABBBAAAB--BBB--AAAA-AABBB-ABBBAABAAABABB-BBABB--BAAAAABA-ABAABBBAAA-A-AA

*001011_00202159 BABBBB-BBABBAABABABBBAAABBBBBBAAAAAAAAABBBAABBB-ABAAABABBBBBABBBBBAA-AABAAABAABBBAAABAAAA

*001014_00298892 BBABABBABABBABBA-ABBABBABAA-AAAABA-BBABAB-ABBBBABAAABBBBBABABBAABAAB-BBAA-AABBBA-AAB-BBAA

*001015_00128215 BAABAB-BBABBAABABABBBAAABBBBBBAAAB-AAAABBBAAB-BBABAAABABBBABABBBB-AAAAABAAABAABBBAAABAAAA

*001015_00175519 B-ABAB-BBABBAABABABBBAAABABBBBAAAB--AAABBBAABABBABAAABABBB-BABBBBBAA-A-BAAABAABBBAAABAAAA

*001017_00038064 BBBABBAAABABAABBBAABAAABBA-A-ABBABBBBBBBAAABABB-ABA-ABABBAAAAAAAABAAAABAABBAABAABAAA-AAAA

*001018_00064034 BAAAABABBBBBABBBA-AAAAAAAABAAA-BBBBBBBBBBBBAAAABBAABBBAAAABBBBBBBAABAA-B-BBABAAB-BBABBBBB

*001018_00144459 BAAAABABBBBB-BBBA-AA-AAAAABAAAB-BBBBBBBBBB-AAAABBAA-B-AAAABBBBBBBAABAABB-BBAB-ABBBBABBBBB

*001020_00026818 BBAAAB-BAA-ABBBBA--ABB-A-A-ABBBAAAA-BAAABBA-BABABAAB-AB-AB-BAABABA-ABB-B-ABBABB-A-BAABABB

*001022_00132845 ABBABABABBBA-BBB--AABBAA--A-AAA-BABA-BBBBAAABBBAAAAAA-BBBABA--AAAABBABBAAAAB-AB-BBB-AB-AA

*001024_00002370 AABABAABAA-BABBAAAA-BBBBABABBAAAAB-BBABBBAABBBBBABBA-BBABABABABA-AABB-AAABABBBB-BBBBABAAA

*001026_00237534 BB-A-BBBAABABBBBAB-ABBBABABABBBABBBBBAAABBABBBBABAAABABAABBBBABBBA-ABABBBABAAAB-ABBAABAAA

*001027_00089281 BBBABBAAABABAABBBAABAAABBA-AB-BBABBBBBBB-AABABA-ABA-ABABBAAAAAAAAAAAAABAAABABBAABAAAAAAAA

*001029_00037171 BAABABABAABBBBBBBBB-BBAAAABAAB-ABBBBBABBBBABABBBA-A-A-ABAAABAABAABBB-AAA-ABBAB-A-A-A-BBBB

*001030_00089329 BA---B-BBABBAABABABBBAAABBBBBBAAAAAAAAABBBAABBBAABAAABABBBBB-BBBBBAA-AABAA-BAABBBA--B-AAA

*001034_00164864 AABABAA-AAABBAAAABB-BBBBBA-ABAB-ABA-BB-BBBAB-AAAAAAA-AABBABAAABABABBAB-B-ABBBB-BBAAAA-AAA

*001039_00227082 --ABABAA-BABA-ABBAAAAA-AB-A-BAB--AA-BAABAA-AA-BBABBAA-ABAAA-AAAABBB-BA-BAAB--A-BBAAAAABAA

*001039_00238783 BAABABAAABABA-ABBAAAAAAABBABBA-BAAABBAABAABAA-BBABBAAAABAAAAAAAABBBBBAABAABBAA-BB-AAAABAA

*001041_00064656 BABABBBBBABA-AB-B-AAABBABAAAABB-BABAABBBABBAAABBAA-ABABAAAAABAABAAA-BAABBBBAA-ABBBBBBBBAA

*001044_00103525 B-BABBBAABBBBABBABAAABABBABBAABA-BA-BABAABBABABAA-ABABABAA-ABBABAABBBBABAABBBBBBBBBBBABBB

*001044_00110846 BABABBBAABBBBABBABAAABABBABBAABA-BABBABAABBAB-BAA-ABABAB-A--BBABAABBBBABAABBBBBB-BBB-AB--

*001045_00043403 A-AAAABAAABABBAA-BA-AA-ABB-AAA-A---AAAAAABA--BBB-ABAB-ABBAAAABBBBABABABABABAAAAAABBA-BBAA

*001046_00037360 ABBABABAAA--BBA-BBBBAAAABBBAAA--A-A-AA-A-B-B-BBBAAB-BBABBAAAAABBB--A---AAAB--A-AABB-A-B-A

*001047_00348687 AAAAAAABAABAAABBAAAABABBBABAABBBBBAAABAABBBABBBAAAABABBAAAAAAABB-AABABBBBABABBBBAAABBABBB

*001047_00348773 AAAAAAABAABAAABBAAAABABBBABAABBBBBAAABAABBBABBBAAAA-ABBAAAAAAABB-AABABBBBABABBBBAAABBABBB

*001048_00034307 BBB-BBAAAA-BBBABABBAA-AAAAABABABA-ABBA-A-BAAA--BBBABB-ABBB-ABAAABBABBABBAAAABBAABBBAAAABB

*001048_00230953 B-BABBAAAA-BBBABABB-AAAAAAABABABAAABBA-AABAAA--BBBABBAABBB--BAAABBABBABBAAAABBAABBBAAA-BB

*001049_00033962 BAB-BBAAAAAB--AA--ABA-BBABBAAAA-B--BB-BAABAAA--AABAAABAABAAAABA-AABA-AABBAABBBBBABB-AB-AA

*001049_00187560 B-BBBBAA-AAB-AAAB--B--BB--BAAAAB-ABBBABAA---A-AAABAA-BA-B-AAABAAAABABAABBAAB-BBBAB--BBAAA

*001051_00175452 BBABABABAABBAB-ABAABBABBBBB-BBBBBBBAAAABAB-BBBABBBBABAAA-BABAABBA-B-AA-BAABBBAABBBBAABAAA

*001054_00204618 BBABABBAABBBBBABBBBABAA-B-AAABBBBBBAABBB-BABBAAABB-A-BABBABBBBBAABBABBB-AABABBBBAAABB-BAA

*001056_00211961 BAAAABAABBAABABABBAAAAAABAABBAAABBBAAB-BBA-AAAAAABABBBABBABABAABBBBBB-BABAAAAAAAAAABBBBBB

*001058_00175642 BBBABB-AB-ABBBAABBBBBAABABABBA-BABB-AA-AB-BBBABBABABBAAAABB-ABAAABBBAB-BBABBAABBBBB-BAABB

*001061_00061538 B-ABABAABBABA-BBBABAAAAAABABBBAAAA-BBABBBBBAAABBABBAAAABAABAAAAAABBBBAABBA--AAABBAAAAABAA

*001063_00269039 A-AAAA--BAAABBA-BBBB-BBBB-BBBAAA-A-BBBABAB-A-BAABBBBBBABBBBAABABBBBBBBB-BA-AAABAABBA-A-BB

*001064_00032894 AAABAAABABABB-BAABBABABABBBBABBBAA-BBA--BAB-BBBA--BABAABABB-ABBABBABBAAAABBBAAAABAAAAAAAA

*001064_00064813 A-ABAAABABABB-BAABBABABABBBBABBBAABBBAA-BAB-BBBA--BABAABABBAAB-ABBABBAAAABBBAAAABAAA-AAAA

*001064_00100167 A-ABAAABABABB-BAABBA--BABBBBABBBAA-BBAABBA--BBBA--BAB-AB-BBAABBAB-ABBAAAABBBAA-ABAAAA-AAA

*001070_00116458 ABBBBABBAB-BAABABABAAAA-ABBAA-BBAB-BABABBBAA-BBBAAABAABABBBAAAABAB-BABABBBBBB-B-ABBAAAB--

*001071_00023496 B-BBB-AAABAABABB--ABBABABABBBABBBBBBABAA-A-BBAB-BBAAA-BABBBAAAAAABAAABAABBBAAA--BBBBB-BBB

*001071_00024309 B-BBBBA-ABAABABB-BA--AB-BABBBABBBBBBABAA-ABBBABABBAAAABABBBAAAAAA-AAABAABBBAAAAABBBBBABBB

*001071_00026334 BBBBBBA-ABAABABB-BA--AB-BABBBABBBBBBABAABABBBABABBAAAABABBBAAAAAA-AAABAABBBAAAAABBBB-ABBB

*001071_00036974 B-BBBBA-ABAABABB-BA---B-BABBBABBBBBBABAA-ABBB-BABBAAAABABBBAAAAAA-AAABAABBBAAAAABBBBBABBB

*001076_00036745 BAABAB-BBABBAABABABBBAAABBBBBBAAABAAAAABBB-ABAB-ABAAABABBBBBABBBBBAABAABAAABAABBBA-ABAAAA

*001078_00030090 ABABAAABABBBABBA-AABBAAB-BABBABBA-AAAAB-AABBBAAAABBABABAABABAABBABB-BAABABBBBAAB-BBAAAAAA

*001080_00198862 ABABAAABABABABBABAABBAABBBBBBABBABAAAAABAA-BBAABABBABABAAAABAABBABB-BAABABBBBAAB-BBAAAAAA

*001080_00206533 ABABAAABABABABBABAABBAABBBBBBABBABAAAAABAABBBAABABBABABAAAABAABBABB-BAABABBBBAABBBBAAAAAA

*001082_00160111 -BBBBBAAAABBABBAAAA-BBABBABBBA-AABAAAAA-BAABA-BBABABABBAABBBBBBBAABABBABAA--A-BAB--BBBABB

*001082_00184200 BBBBBBAAAABBABBAAAAABBABBABBBA-AABAAAAA-BAABABBBABABABBAABBBBBBBAABABBABAA-AA-BAB--BBBABB

*001083_00050118 AABABABABBBBABBBAABABBBBAABAABBB-A-AABABBB-BAA--BBAA-BABB-BBAABAAABBBAAABBBAA-BABAAABBBAA

*001083_00122031 AABABABABBBBABBBAABABBBBAABAABBBAAAAABABBB-BA--BBBAA-BABB-BBAABAAABBBAAABBBAA-BABAAABBBAA

*001086_00073778 --AA-BAB-AAA-AABBB-AAABAA--BABB--BAAA-ABAB-BA-BAAAAABBAABABAAAABBBABB-BA-BBBABA-B-AABB-AA

*001087_00208459 BB-B-BABABABBAABBBBBBAAA-BAABBAAABABBBABBBBAA-AA---BA-BBABBAABBBBBBABBBBBBBABBBBABBBAAABB

*001091_00071219 ABAAAAABAABAAABBAAAABBBBBABA-BBBBBAAABAA-BBBBBBAABABABBAAAAAAABB-AABABBBBABABBBBAAABBAABB

*001091_00071630 A-AAAAABAABAAABBAAAABBBBBABAABBBBB-AABAAABBBBBBAABABABBAAAAAAABB-AABABBBBABABBBBAAABBA-BB

*001092_00149353 BAABABABBBA-BABBBBBBAAAABA-BB-AA-B-AABBBBABB-AABBAAB-BABBAABAAABAABBABAABBAB-ABAAAAAAB-BB

*001095_00074963 BAABABAABBABA-BBBAAAAAAABBABBABBAAABBAABAABAAABBABBAAAABAABAAAAABBBBBAABAABBAA-BBAAAA-BAA

*001095_00118099 BAA-ABAAABABA-BBBAAAAAAABBABB-BBAA-BBAA-AABAAAB-ABBAA-ABAA-AAAAA-BBBB-A-A--BAAABBAAAAABAA

*001096_00063185 B-ABABAABBABA-BBBABAABAAABAABBAAAA-BBABBBBBAAABBABBAAAABAABAAAAAAABBBAABAA-BAAAB-AAAAABAA

*001098_00196997 A-ABAA-BBBBB-AAAA-BAABAABAAABABAAA-BBABAB--B-BBB-AAAABABAAAAABABBAABAB-BA-BAABBB-AABBBBAA

*001100_00097051 BABABBBAAABAABA-BAABABAAAABBBBAAABAAABABBABAABABBA-BB-BBBABABAABB-AAAABABBABBAAAABBAAAABB

*001100_00133913 BABABBBAAABAABA-BAABABAAAABBBBAAABAAABABBABAABABBA-BBABBBABABAABB-AAAABABB-BBAAAABBAAAABB

*001103_00144766 BBB-BBAAAA-BBBABABBBAAAAAAABABABAAABBA-AABAAA--BABBBBAABBB-ABAAABBA-AABBAAAABBAABBBAAAA-B

*001106_00228337 B-AB-B-B-AABA-BABABA-AABAABBBBAAABABAAA-AB-BBAB-ABAAA-A-B-ABABBBBBAA-AABAA-AAABBBAAA-AAAA

*001107_00096283 BABABB-BBA-BAABAAAAA-AAAA-BBB-BAABA--AABAB-BA-BBABAAABAABAAAABBBBBAAAAABAABAAAABBAAABAAAA

*001110_00094606 BBAA-B-BBABBAABABAABBAAABABBBBAAAAAAAABBBBAABBBAABAABBABBBBAABBBABAA-BAA-AABAABABB-AAAAAA

*001110_00163683 ---AA----A--AABA-AA-BAAA-AB--BAAAAAA-ABBBBAABBBAA-AAB-ABBBBAA-BBABAA-BAAAAABAABA-BBABAAAA

*001111_00285590 AABBBAABAAABBBAAAB-BBBBBAABAAABBABABBBABBBAAAAAAAAAABB-BBABAAABAB-BB-BABBABBABBBBAAABBAAA

*001112_00049984 BBAAABBBBB-BA-AABABBBA-BA-ABBAA-BBA--A-B-ABAA-BBAAAABAABABB-ABAAABBBBBAAAABBAA-ABAA-A-AAA

*001115_00110612 B-BBBB-B-A-BB-BA-B-BBAAABBBBBAB--A-AABA--AABBABBB-BA-ABB-A--BABAAB-B--ABA-ABAABBAB-BA---A

*001116_00078718 B-BBBBAABA-B-ABBBBA-AB--BABBBAABAAAA-B---BAA---AABAA-AAA-BAABBAAB-BB-A-BBBA-BB-BA-B---A-A

*001120_00138488 BAABABAABABAABAABAAABABBBBAABBAABA--AA-BABABBAABAAAABBBBAA-BAB-BBAABBAABBAAABBABBBBAAA-AA

*001120_00211988 BAABABAABABAABAABAA-BABBBBAABBAABABAAA-BABABBAABAAAABBBBAABBAB-BBAABBAABBAAABBABBBBAAA-AA

*001122_00054690 B-BBBBAA-AAB-BAABB-BAABB-B-AAAA-BAB-BAB--B-A-A-AAB--BBAA--AAAB-AAAB-BA--BAABBBBBAB-AA-AA-

*001124_00049537 ABABAABAABBBABABAABABBBAAABAABBBB-BAABAB--ABBA-BBBAA-BABAABBABBAAABABBBAA-BAA-BAAAAABBBAA

*001125_00037138 BABABBBAAAB-ABBBBAA-BABBAABABBBAABAB-BAABB-AAA-BABAABABBBAABBA-AAB-AABBBBABAAA--BBBB-B-AA

*001125_00042236 B-B-B-BA-ABBABBBBA--B-BBAABA-BB--BAB-B-A-B-AA--BABA-BAB-BAABBABAA-AAA-BBBAB-AABA-B-B-B-AA

*001125_00079470 BABABBBAAABBABBBBAA-BABBAABABBBAABAB-BAABBAAA-BBABAABABBBAABBABAAB-AABBBBABAAAB-BBBBBB-AA

*001125_00237516 B----BBAAABBABBBBAA-BABBAABABBBAABABBBAABBAAAABBABAABABBBAABBABAAB-AABBBBABAAAB-BBBBBB-AA

*001127_00020815 BBABABBAABBBBBABBBBABAA-B-AAABBBBBBAABBB-BABBAAABB-A-BABBABBBBBAABBABBB-AABABBBBAAABB-BAA

*001127_00138509 BBABABBAABBBBBABBBBABAA-B-AAABBBBBBAABBB--ABBAAABB-A-BABBABBBBBAABBABBB-AABABBBBAAABB-BAA

*001128_00068338 B-AAABAAAABAAABBAABABAABBBBAAABBABABB-BA-B-BBBBAABABBAAAABAABABABAABABABABBAB-ABAAABBABBB

*001129_00222239 B--BBBAAAAAB-AAABB-BA-BB-BBAAAAB--BBBABAABA-A-AAABAA-BAABAAAABAAAABABAABBAABBBBBABBA-BAAA

*001133_00275980 B-ABABAAABBAAAABB-AABABAAABAAABA-A--BBBAAABAAAAA-BBAA--B-A-ABAA--AAAAB--BAB-BAB-B-B---B-A

*001134_00086983 BBBBBBAABAABBB-BBBA-AABBAABBAAABAAAAAABAABAAAA-BABBBAAAABB-ABAAABBBB-ABBBBABBBBBABBA-BABB

*001134_00177664 BBB-B--ABAABBB-BBBA-AAABAABBAAABAA--AABAAB-A-AABABB-AAA-BBBAB-AABBB-BA-BBBABBB-B-BBA-BABB

*001137_00043707 A-AAAABBAABABBAA-B--AA-AB--AAA-A--BAAAAAABA---BB-BBAB-A-BAAABBBBBABABABABABAAAAAABBAABBAA

*001142_00121662 A-BBBABBA-ABBAAAABAABBABAABABB-ABBBBBBBAAAB-BBABBA-ABAA-BAABBAABBBB-B-BBBABAAAABABBABB-AA

*001143_00043062 B-B-BBBBABABBAABBBBBBAB-ABBBBBABAAAAABB-BAAABABBAA-BBABAABBABABABA-BBAABAAAABBBABBBAAABBB

*001143_00093134 B-B-BBBBABABBAABBBBBBAB-ABBBBBABAAAA-BB-BAAABABBAABBBABAABBABABABABBBAABAAAABBBABBBAAABBB

*001144_00066886 BAAB-BAAAAABBAABBBAB--AA-AABBA--BABBABBBAA-ABAA-BBABAABAAB-A-ABABBBABBAAAABBAABABA-ABABBB

*001144_00219448 BAABABAAAAABB-ABBBA-BAAABAABBABBBABBABBBAABABAABBBABAABAAB-AAABABABABBAAAABBAABABAAABABBB

*001145_00051554 A-BABABAB-BA-BBB--AA-B-AB-AAAAAAB-BAABBBBAAABBBBAAAA-ABBBAB-BBAAAABBA-BAA-ABBABABBBAABBAA

*001145_00114686 ABBABABABBBA-BBB--AABBAABBAAAAAAB-BAAB-BBA-AB-BBAAAA-ABBBABABBAAAABBABBAAAABBABA-BB-ABBAA

*001147_00066683 B-AAAB-B-BABAAAAB--BBA-BABABBA---BA--ABB-A--AABBAA-A-AABABB---AAABBBB-AAAAB-AA-A-AA-A-AAA

*001150_00095302 B-BBBBB-BBBAABBABAAABABA-ABAABBABABBBBAAABBBAB-BBB--B-BABABAAAAAAABBBBBBABBAABABBAA--BAA-

*001153_00052258 B-BABBA-ABBAAAB-AABABAABAABBAABAAAA-BAB-AB-AAA-AAAB-BAA-AABABABB-AABABBABABBBA-A-BB--ABAA

*001157_00142615 B-BBBBAABAAB-BAAAA-BA-BB--BAAAABBABBBABAABAABAA-A-AA-BA-BAAAA-AAA-BABA-BB-ABBB-BAB-A-BAAA

*001159_00237711 A-BBBAAABABABBAAABBBBABBBBAAAB-BAAA-AABB-BABBAAAABAABBBA-ABBA-ABBAABBAABBB-AB-ABBBBAAABAA

*001164_00132177 BA---B--BABAAAB-A-ABABAABBAAABABBB-AABB-BBABAAABAAAABAB-AAAAA-A--AAA-AA---AAAABB--B---BAA

*001164_00147005 BABBBBB-BABABAB-A-ABABAABBAAABABBBBAABB-BBABAAABAAAABABAAAAAABABAAAABAABB-AAAABBBBBBBBBAA

*001164_00231319 BABBBBB-BABA-AB-A-ABABBABBAAABABBBBAABBBBBABAAABAAAABABAAAAAABABAAA-BAABBBAAAABBBBBBBBBAA

*001166_00070228 B-BBBBA-ABAABABB-BA--AB-BABBBABBBBBBABAA-ABBB-BABBAAAABABBBAAAAAA-AAABAABBBAAAAABB-BBABBB

*001169_00160124 BBBABBBBAA-A-BBAA-ABBBBBBBBABBAABBBAAABBBBBABABBBAAAAABABAABAAB-A-B-BAA-AABBABBBAAABBABAA

*001170_00016049 BAABABABAABBABBABAABB-BAB-AABBBABBBAAAABABBAB-ABBBBABAAAABBBAABBA-BBAABBAABBBAABBAAAAAAAA

*001174_00013265 B-AAABBAABBBBAABABAAAAABBA-BAABB-BABB-BAABBB-B-AAA-BAAABBAAABBABAAABBBABAABBBBBBBAABB-BBB

*001174_00155674 B-AAABBAABBBBAABABAAAAABBA-BAABB-BABB-BA-BBB-B-AAAABAAABBAAABBABAAABBBABAABBBBBBBAABB-BBB

*001175_00026260 BBAB--BAA-B-BBAB-BBABAA--BAAABB-BBBAABBB-BAB--AABBBA-BAB-ABBBBB-ABBABBB-AABABBB--A-BBBBAA

*001175_00030708 BBABABBAABBBBBABBBBABAA-BBAAABBBBBB-ABBB-BABB-A-BBAA-BABBABBBBBAABBABBB-AABABBBBAAABB-BAA

*001175_00173057 BBAB--BAABBBBBAB-BBABAA----AABB-BBBAABBBAB-BBAAABB-A-BAB--BBBBBAABBABBB-AABABBBBAAAB--BAA

*001175_00219918 BBABABBAABBBBBABBBBABAA-BAAAABBBBB-AABBBA-ABBAAABB-AABAB-ABBB-BAA-BABBB-AABABBBBAAABB-BAA

*001176_00121048 BB-A-BBAAABBBBBBBBBABBB-AAAAAAB-BBBAAAABBABBBB-AABABAABBBBBBBABABBAABAAABABB-BAABBBABABBB

*001176_00124651 BBBAABAAAABBBBABBBBABBB-AAAAAAB-BBBAAAABBA-BBB-AABABAAABBABBBABABBAAAA-ABABBABA-BBBABABBB

*001178_00126309 BA-BAB-BBABBAABAB--BBAAABBB-BB-A---AAAA--B-AB-BBABA-AB-BBBB-ABBBBB-A--ABAAABAABBB-AA--AAA

*001179_00010971 B-A-ABBBABB-BABA-BBBAABAAAABBBBBBBBAABBA-BABB--BBABBA-ABABBBB-ABABAAABBABABBBAAABBBBBBABB

*001179_00183772 B-A-ABBBABB-BABA-BBBAABAAAABBBBBBBBAABBAABABB-BBBABBABABABBBB-ABABAAABBABABBBAAABBBBBBABB

*001186_00144688 BABB-B-B-ABBAAB-BABB-AAABBBB-BA-AA---AA-BBAABBB-AB-AABABBBBBABB-BBAA--A--AABAAB--A---A-AA

*001186_00359301 BABBBB-BBABBAABABABBBAAABBBBBBAAAAAAAAABBBAABBBAABAAABABBBBBABBBBBAA-AABAAABAABBBAAAB-AAA

*001187_00189588 A-B-B-ABA-AA-A-A--ABBBAABA--BBBBBBBBABBBAB-AA-A-BAABB-A-A---A-BABABAA-B-AAB-AA-BBAAAABAB-

*001188_00099095 BABABBBBBB-AA-B-B-AAAABB-AAAABB-BA-AAB-BABBAAAB-AA-ABABAAAAA-AAB-AAA-AA-BB-AA-AB-------AA

*001188_00121146 BABABBB-BBB--AB-B-AAA-BB-AAAABBBBA-AABBBAB-AAABBAABAB-BAAAAABA-B-AAABA-BBB-AA--BBBB-BB-AA

*001189_00096458 ABA-AAABAABABABBBBAAAABAAABAAABBBBBBBBABBBBAABAAB-ABBBAAAABAABAB-AABBABBBABAB-ABABBBBABBB

*001193_00187380 ABBBBAABAABABBB-BBAAABAA-BBABAABBABAABBBBBAABAB-BBBBBBABBBBB-BAABAAABAABBAAABBBBABBBAAAB-

*001195_00005919 BAAAAB-BBAABBBAA-BBAAABAAABAAAABBABAAABBBBAABA-AAAA-AB-BABAAB-AAA-ABAAAAAAABB-AABBBBBAAAA

*001195_00139921 B-B-BB-BBAABBBAA-BBA-ABAAABAAAABBA-AAAB-BBABBA--AAABABBB-A-AB-AA---BAAAAAA-BB-AA-BB---AA-

*001196_00184199 BBBABBAAABABAABBBAABAAABBA-ABABBAB-BBBBB-AABABA-ABA-ABABBAAAAAAAAAAAAABAAABABBAABAAAAAAAA

*001197_00006651 B-BBBBBABBBBABAABABBABAABAAAAAAABA-BBABAB--BBBBB-AAAABBBBABABBAABAABABBAA-AABBBA-A-BBBBAA

*001197_00057695 BABBBBBABBBBABAABABBABAABAAAAAAABA-BBABAB-ABB-BB-AAAA-BB-AAABBAABAABABBAA-AABB-AAAABB-BAA

*001197_00127862 BABBBBBABBBBABAABABBABAABAAAAAAABA-BBABAB-ABBBBB-AAAABBBBAAABBAABAABABBAA-AABBBA-AABBBBAA

*001197_00141346 BA-BBBBABBBBABAABABBABAABAAAAAAABAA-BAB-B-ABBBBB-AAAAB-B-A-A-BA--AA-ABBAA-AABBBAAAABB-BAA

*001199_00021037 BAABABABBBA-BABBBB-BAAAABA-BA-AA-B-AABB--ABB-AABBAABBBAB-AABAAABAABBABAABBAB-ABAAAAAAB-B-

*001201_00040615 B-BBBBABAABABAABBB-A-AAABAABBABBBA-BABBAAA-ABAABBBABAABAABBAAABA-ABABBA-BABAAA-ABAAABABBB

*001201_00055452 BABBBBABAA-ABAABBBA--AAABAABBA-BBABBABBAAABABAABBBABAABAABBAAABA-ABABBAABABAAA-ABAAABABBB

*001202_00071754 BBB-BBA-ABBA-ABAB-AAAABB--ABAA-AAAAAABB-AB-A-A-BAA-ABBB-ABA-BAAAABA-B-ABA--AAAABBBBBA-BAA

*001202_00218172 BBBABBA-ABBA-ABAB-AAAABB-AABAABAAAAAABBB-BBA-A-BAA-ABBBAABAABAAAABAABAABA-BAAAABBBBBAA-AA

*001203_00283713 B--B-BABABABBAABBBBBBAAA-BAABBAAABABBBABBB-AAB-A-A-BA-BBABBAABBBBBBABBBBBBBABBBB-BBBAAABB

*001204_00118896 BBBABBABABAAABBAAABABBABAABBAA-AAAABBABBAB-AAABAAABBB-A-AAABABBBAABBABA-BABBBABBBBBABABBB

*001207_00158686 BBAAABAABABB-BBAA-AA-AAAAABAAAA-BBBBBBBBBBBAAAABAABAABAAAABABBABB-A-AAABABAABAABBBBABBBAA

*001207_00164200 BBAAABAABABB-BBAA-AA-AAAAABAAAA-BBBBBB-BBBBAAAABAABAABAAAABABBABB-A--AABABAABAABBBBABBBAA

*001208_00069496 B-BBB-ABAB-B-AABBABBBAAABABABB-ABBBAABA--B-AA-AAAA--ABBBBABAABB-B-AA-BBB-ABAABABAAAB-BBBB

*001212_00130462 BBAAABABBBBB-BBAAAA----A-ABA-AAABBB-BBBBBBBAAAAB-AAA--AAAABBBBABB-AB---BABAABAA-BB--BB-AA

*001214_00055944 AABBBAAABABAABAABAABBABBBBAA-BABBABAAAB-BB-BBAABABA-BBBAAABBA-ABBAABBAABBAAAA-ABBBBAAA-AA

*001214_00060036 AABBBAAABABAABAABAA-BABBBBAABBABBABAAABBBBABBAABABAABBBAAABBA-ABBAABBAABBAAAA-AB-BBAAA-AA

*001214_00203886 AAAB-AAABABAABAABA--B-BBB-A-BBA--ABAAABBBB-BB--BABAABB---ABBA-AB-AABBAABBAAAAAABBB-A-A-AA

*001214_00218676 AAABAAAABABAABAABAABBABBBBAA-BABBABAAA--BBABBAABABAABBBAAABBA-ABBAABBAABBAAAAAABBBBAAA-AA

*001214_00219152 A-ABAAAABABAABAABAA-BABBBBAABBABBABAAABBBBABBAABABAABBBAAABBA-ABBAABBAABBAAAAAABBBBAAA-AA

*001215_00023602 AABABAABAAABBAAAABBBBBBBBA-ABABBABABBBABBBAB-AAAAAAABAABBABAAABABABB-B-B-ABBBB-BBAAAABAAA

*001220_00025473 A-AAAABAAABA-BAA-BB-AA-ABB-A-A-AABAAAAAAAB-B-BBB-ABABAABBA-AAB-BBAAABABABABAAA-AAB--A-BAA

*001220_00123854 A-AAAABAAA-ABBAA-BBBA--ABB-AAA-AA-AAA-AAABA-ABBB-AB-B-ABBAAAABBBBA-ABA-A-AB-A-AAABBA-BBAA

*001222_00252736 BBABABAB-ABBBBBBABBBABAAAABABB-ABABAAAAABAABABAABBABBAABBB-B-AAABBAAABAABAB-ABBAABBAABBBB

*001222_00356540 BBABABA--ABBBB-BABBB-BAAAABABB--BABA-A-AB--BABA-BB-B-AABBB-BBAAABBA-ABAABAB-ABBAABBAABBBB

*001223_00156772 BBABABBAABBBBBABBBBABAAAB-AAABBBBBB-ABBB-B-BBAAABB-A-BABBABBBBBAABBABBB-AABABBBBAAAB-ABAA

*001224_00204352 BABABBBABABBAABBBAABBBBBABBABABAABAAABAABBAAAA-BBAAAAABBBAABBABABB-AABBBAABBAAB-BBBBBB-AA

*001225_00017957 BAABABABAABBABBABA-BBABABB-ABBBABBBAAAABABBABBABBBBABAAAABBBAABBA-BBAA-BAABBBA-BBAAAAAAAA

*001225_00154749 BAABABABAABBABBABA-BBABABB-ABBBABBBAAAABAB-AB-ABBBBABAAAABBBAABBA-BBAA-BAABBBAABBAAAAAAAA

*001226_00204605 BABBBB-BBABBAABABABBBAAABBBBBBAAAAAAAAABBBAABBBAABAAABABBBBBABBBBBAA-AAB-AABAABBBAAABAAAA

*001229_00112235 A-BABAABAABAAAAAAAABBAA-BABABBBBBB-BAABBABBABAABBA-BBAAAAABAAABB-BBAAAB-AABAABBBBAAAAB-BB

*001233_00160587 AA-B-ABAAABBABBBBAABBABBABBABBBABB-B-AAABBBABABBAB-AB-BBAAABBABAAB-AABBABAAABABAAAAB-BBAA

*001235_00064104 B-BBBBAAABBAAAABBA-ABAB-BABAAABAAAAAABB-AABAAB-BABBAAABBAAAABAAAAAAAAB-AA-BA-ABBABBBAABAA

*001236_00132775 AAAAAA--BAAABBA-BBBBBBBBB-BBBABA-A-BBB-BABBA-BAABBBBBBABBBBAABABBBBBBBBABA-AAABAABBAAA-BB

*001236_00177436 A-AAAA--BAAABBA-BBB-BBBBBABBBABABA-BB-ABA-B--BA-BBB----BBBBAABABB-BBBBBABAAAAABA-BBAAABBB

*001240_00162140 BBBBBBAABAABBB-BBBA-AABBAABBAAABAAAAAABAABAAAA-BABBBAAAABB-ABAAABBBBBABBBBABBBBBABBA-BABB

*001244_00104701 A--ABAABAA-BBBAAABABBBBBAABAA-BBA---BBAB-BAAAAAAAAA-BBABBABAAA-ABABB-B-BAABBAB-BBA-ABB--A

*001246_00000572 BBAAABAABB-AAAB-BAABBABBABBAAAAA-BBAAA-AAB--BABBBABBAABABAABABBBBAABA-AAABBAABBAAAAB--BBB

*001246_00000658 BBAAABAABBBAAAB-BAABBABBABBAAA-ABBBAAA-A-BAAB-BBBABBAABABAABABBBBAABA-AAABBAABBAAAABBABBB

*001247_00079351 AAAAAAAABBBAAAB-BAAB-AB-AABAAAA---AAAABA-AABA-BBB-ABAABA-AABABABBAABA-AAABBAABBA-AABB-ABB

*001250_00159596 A-BBBAAA-BBBABBABAA-BBAB-BABAABA-BABAABBAABBB-ABAAAABAAAABABABBBABBAAABBABBBA-ABBAAAABAAA

*001250_00178039 A-BBBAAAABBBABBABAA-BBAB-BABAABAABABA-BBAA-BBBABAAAABAAAABABABBBABBAAABBABBBAAABBAA-ABAAA

*001252_00117707 B-AB--AB-BABBAABBBBB-AAA-B-ABBA--BABBBABBB-A-BAAA--BAABB-BBAABBBB-AAB-BBB-B-BB---BB--AA-B

*001252_00248553 BB---BABABABBAABBBBBBAAA-BAABBAAABABBBABBBBAABAAAA-BABBBABBAABBBBBAABBBBB-BABB-B-BBBAAABB

*001253_00126072 B-ABABAB-ABBBBBBABBBBBAAAABABBBABBBBBABBABABABBAABAB--ABABABB-BAABBBAAAABAB-ABBAAAAABBBBB

*001257_00004272 -BBBBABAAABAAABAAAAAABABAAAABBABBBBA-BBAAABABBABBB--B-AABA-AAAAB-BBB-BABBAABBBABBBBB--A--

*001257_00111107 ABBBBABAAABAA-BAAAAAABABAAAABBABBBBAABBAAABABBABBB-AB-AABA-AAAAB-BBBABABBAABBBABBBBBBAA--

*001257_00153007 A-BBBABAAABAA-BAAAAAABABAAAABBABBBBAABBAAABABBABBB-AB-AABA-AABAB-BBBABABBAABBBABBBBBBAA--

*001262_00071268 AABBBAABAAABBBAABB-BBBBBAABAAABBABABBBABBBAAA-AABAAA-B-BBABAAABABBBB-BAB-ABBABBBBAABBBAAA

*001266_00036367 ABBABABBAABABBAB-BB-AABAABBAAB-AAAAAAAB-ABAAABBAAABBBA-BBAAABABBBAB--AAAAABAAA-AABBBBABBB

*001269_00072604 B--BBBABBA-BBAABABBAABBAAAABABBBAAAAABB-ABBB-AABBBBAAA-BBBABAB-BA--AA--ABBBAAA-BBB-BBBB-A

*001272_00117337 B-A-ABAAABBAAA-BBA-AB-BA-A-AA-BAAAA-B-BAAA-AA-AA-B-A--ABA-AABAAA-AAAABB-BABBBABA-BBBAA-AA

*001273_00027781 --BAB-BAAB-BAAABBA-ABABABABA-ABABABBBBBB-ABBAB---B--AA-BAAABBA-ABBBBBAB-AABB-B--ABBB-AAAA

*001274_00212638 BA-BBBABABABBAABBBBBBAAAABAABBAAABABBBABBBBAA-AA-A-BA-BBABBAABBBBBBABBBBBBBABBBB-BBBAAABB

*001274_00237396 B--B-BA-AB--B-ABB-BBBAA-BBAA-B-AABABBBABBBBAA-AA-A-BABBBABBAABBBBBB-BB-BBBBABBBB-BBBAAABB

*001276_00213450 B--ABBBBABB-A--B-A-AAB-AB--BBB-A-AA-BBABAAAAABABAAA-ABA-ABABAAABB-BBA-AABBAB-B--BBB-AABBB

*001277_00039986 BBABABBAABBBBBABBBBABAA-B-AAABBBBBBAABBB-B-BBAAABB-A-BABBABBBBBAABBABBB-AABABBBBAAABB-BAA

*001278_00125693 BBAAAB-BABABBBAB-BABBBBBAAAABAAA-BB-BABBABBAB--AAABABAAABBBABABABBABABABBAAAAABBAA-BBBBAA

*001278_00130024 BBAAABAB-BABBBAB-BAB-BBBAAAABA--BB--BABBAB-AB--AAABABAAAB-BABABABBABABA-BAAAAABBAAABBB-AA

*001278_00140004 BBAAABABABABBBAB-BABBBBBAAAABAAA-B--BABBABBABA-AAABABAAAB-BABABABBABABABBAAAAABBAAABBBBAA

*001279_00048901 BAABABAABBBBA-BABABAAAABAAABBAAAAAA--ABBBABBAA-BABBAABABAABA-BAAAAB-AAABAA-BBAABBAAAAABAA

*001280_00120241 AABABAABAAABBAAAABBBBB-BBAAABAB-ABABBBABBB-BB-AAAAAABAABBABAAABABABB-A-B-ABBBB-BBAA-ABAAA

*001282_00003979 -BBAB-BBAAAABAABBBBBAAB-ABBBABABAAAAAABBAA-ABABBAABBBABBBABABABABAB-BAAAAAAABA-ABBBAAABBB

*001282_00052887 -BBABABB-AAABA-BB-BB-AB--BBBABA-AAAAAABB-A-ABABBA-BBBABBBABABABABAB--AAAAAAABAAABBBAAABBB

*001282_00058935 -BBAB-BBAAAABAABBBBBA-B-ABBBABAAAAAAAAB-AA-AB-B-AABBBABBBABA-ABABAB-BAAAAAAABAAABBBAAABBB

*001282_00104637 ABBAB-B-AAAA-AABBBBAA-B-ABBBABAAAA-AAAB-AAAABAB-AABBBABBBABA-A-ABAB-BAAAAAAABA-A--BA--BBB

*001284_00117143 A-B-BAABAABAABB-BAAA-BAAABBAAAABAA--ABBABBAAAAABBBABBBA-BAAAABA--AAB--AABAABBBBAABB-A-AB-

*001287_00089116 B-BABBA-ABBBBABBABAAABA-BABBAAAABBBBBABA-B-A-ABAA-A-ABABAA-AABABAAABBB-BAAA-BABB-BBBBA-BB

*001287_00098345 BBBABBAA-BBBBABBABAAABA-BABBAAAABBBBBABAAB-A-ABAA-ABABABAA-AAB-BAAABBBABAAABBABBBBBBBABBB

*001288_00001628 A-AAA--A-ABABB-AA------A---AAA---B--AAAAAB---BBBAABAB-A-BAA-ABBBBABABABABABAAAAAABBAABBAA

*001288_00120685 A-AAAABAAABABBAA-BA-AA-ABB-AAA-A---AAAA-ABA---BB-ABAB-A-BAAAABBBBABABABABABAAAAAABBAABBAA

*001291_00041924 BBAAABABBBBAAABABAABBAABABBAAAAABBBAAABAABAAB-BBBABBAABABAABABBBBABBA-AAABBAABBAAAABBABBB

*001292_00043248 AAAAAABBAABABBAAABAAAAAABB-AAA-A--BAAAAAABAB-BB--ABAB-A-BAAABBBBB-BAB-BABABAAAAAABB---BA-

*001292_00047241 A-AAAABBAABABBAA-BA-AA-ABB-AAA-A--BAAAAA-BA--BBB-ABAB-A-BAAABBBBBABABABABABAAAAAABBAABBAA

*001292_00297194 A-AAAABBAABABBAA-BA-AA-ABB-AAA-A--BAAAAAABA--BBB-ABAB-A-BAAABBBBBABABABABABAAAAAABBAABBAA

*001294_00070732 B-BAB-BAABBBBABBABA-ABA---BBAA-A-B--BABA-BBABABA--ABABABAA-A-BABAA-B-B-BAAABBB-B-BBB--BBB

*001294_00179094 B-BABBBA-BB-BABBABAAABA-BABBAA-ABBBBBABAAB-ABABAA-ABA-A-AA-AABABAAAB-BABAAA--BBBBBB-BABBB

*001297_00203688 B-BBBB-BAABBBABA--ABAAAABBBBBABAAAAAABAA-AABBABBBABAAABBBABBBAAAAB-BB-BAAAABAA-AABBBABAAA

*001301_00090885 AABAB-ABAA-BAB-AAA-BB-BBABAB-AA-----BABBB--BB-BBABB---B-BA-AB-BAB-ABBA-A-BABBBBABB-BABAAA

*001302_00091022 B-ABABABBBA-BA-B-B--AAAABA-BA-AA--AAABBBBABB-AA-BAA--BABBAABAAABAABBA-AABBAB-ABA-AA--B-BB

*001302_00170998 -AABA--BBBA-BA-BBB-B-AAA--BBABA--B-AABBB-ABB--AB-AA--BAB-AABA-ABAABB-B-ABBABAABAAAAAAB-BB

*001305_00084426 BBAAABBA-AABBAAAB--B-AABA---BA--BB---ABABA-BA-B-A-AABAAA-BB-ABAAAB-BB-A-AAB-AA-ABAA-AA-AA

*001305_00092490 ----ABBABB--BAAABBBBBA-BA-ABB--B-BAAAA-A--BBAA--AAA-BAAAB-BBAB-A-BBBBBABA-BBAABABAA--AAA-

*001306_00050934 BAB-BBAAAAB-BBABABBABAAAAAA-BBB-BAB-BA-AABAAAB-BBBA-AAABBA-ABBBABAAB-ABBAABABBAA-BBBAAA-B

*001306_00152811 BBBBBBAAAABBBBABABB-BAAAAAA-BBBABABBBABAABAAAB-BBBAAAAABBA-ABBBABAABBABBAABABBAABBBBAAA-B

*001307_00082815 ABAAAAABAABABABBABAAAABAAABAAABBBBBBBBABBBBAABAAA-ABBBA-AABAABABBAAABAABBABBB-ABABBBBABBB

*001312_00189844 BAABABAABBABA-BBBAAAAAAABBABBAB-AAABBAABBA-AAABBABBAAAABAABAABAABBBBBAABAABBAA-BBAAAAABAA

*001318_00054579 BBAAABAAAABABABAABAABBBABABABAAABB-BBABBBBBBBBB-AAAAB-BAABBBBABBBABABAABAAA-ABABBBBAABAAB

*001321_00005579 BABABBBAAB-AAAABBA-ABA-ABABA-ABA-AABBBABBABBAB--BB-AAA-BAAABBA-ABABBBAB-BABB--A-ABBBB-AAA

*001321_00007076 BABABBBA-B-AAAABBA-ABAB-BABA-ABAAAABBB-BBABBAB--BBBAAA-BAAABBA-ABABBBAB-BABB-BA--BBB-BAAA

*001321_00051948 BABABBBAAB-AAAABBA-ABABABABA-ABAAAABBBA--ABBA---BB-AAA-BAAABBA-ABABBBAB-BABB-BA-ABBBBBAAA

*001321_00076491 BABABBBA-B-AAAABBABABABABABAAABAAAABBBABBABBABB-BB-AAA-BAAABBABABABBBAB-BABBABA-ABBBBBAAA

*001321_00078664 B-B-BBBAAB-AAAABBA-A-AB-BABA-ABAAAABBBABBABBABB-BB-AAA-BAAABBA-ABABBBAB-BABB-BABABBBBBAAA

*001321_00123067 BABABBBAABAAA-AB--BABABABABABAB--A--BBABBA-BAB--BB-AAAAB-A-BB----ABBB-B-BABB-BA--BBBBB-A-

*001322_00246714 -BB-B-BB-AAABBAB-BBAAAB-AB--ABAAAAAAAABBAAAABBB-A-BBBA-BBABABABABAB--AAAAAAAAABA-BBAB--BB

*001322_00352415 -BBAB-BBAAAABBAB-BBAAABAABBBABAAAAAAAAB-AA-ABBBAAABBBA-BBABABABABAB--AAAAAAAAA-ABBBABABBB

*001323_00050197 BABABBBBBBBBABBAAABABAB--ABBABBBAAAAABAABB-AAB-BBBBABABABABBAAAAAABBBAABBBBAAB-BBAAA-BBAA

*001324_00119604 ABBABAABAABABBB-BBBAABAB-BBABAAABABAABBBBBABBABABBBBBBABBBBB-AAABABBBB-BBAABBBBBABBBA-A--

*001327_00014752 ABBABABBAAAA-BAAA-ABBBBBBBBABBBBBB-AAABBBBBABAABBAABABBBBABBAABAAABABABAAABBABBBAAAAAABBB

*001327_00041610 A-BAB-BBAAAAABAAA-ABB-BBBBBABBB-BBBAAABBBBB-B-ABBAAB-BBBBABBAABAAABABABAAABBABBB-A-A-A-BB

*001328_00031991 BABABBBAABBAAAABBABAABAABABABBB-AAABBBBBAAAAABABABABABAAABABABABBABBAABBBBABBBAABBBBB-BBB

*001329_00090658 BBABABABABBBABBBBA-BAABAA-AABBBABBBAAAABABBAABABBBBABABAA-B-BAABABBBAAABAABABAABBBBAAAAAA

*001329_00148850 B-ABABA-ABBBABBBBA--AABAA-AABBBABBB-AAA-AB-AAB-BBBBA--B-A-B-BAABABBBAAABAABABAAB-B-AAAAAA

*001329_00292327 BAABABABABBBABBBBA-BAABAA-AABBBABBBAAAABABBAABABBBBABABAA-B-BAABABBBAAABAABABAABBBBAAAAAA

*001331_00028307 AAABAAABAAABB-BAABBABABABBBBABBBAABBBA-BBAB-BBBA--BABAABABBAABBABBABBAAAABBBAAAABAAAAAAAA

*001333_00001119 AABABAABAAAAABABBA-BB-AB-ABAABBAABABBBABAB-AAAA-BAAA-BBBBABAA-BAABBBABAB-ABBABB-AAABBAAB-

*001335_00096736 B-ABABBABBBBBBBABBBBBBBBBAB-AAA-BABBBAB-BAABBBBABAAABBABBABA-B--BAAB-BBAA-ABBBBA-BBBBBAA-

*001336_00013872 A--BAABB-BBAAAABBAAABBB-BBBBBABAAAAAA-AB-AAAB-ABBAAB-AAAABAAAABBBBBBB---BB-B-BA-BBB-AABBB

*001336_00181906 AAABAAB--BBAAAABBAAABB--BBBBBABAA-AAA-ABBA-ABBABBAABAAAAABAAAABBBBBBBAAABBABBBA-BBBAAABBB

*001336_00182649 A-ABAABBBBBAAAABBAAABB--BBBBBABAAB--ABABBA-ABBABBAA-AAA-AB-AA-BBBBBB-AAABBABBBA-BBB-A-B-B

*001337_00023590 B-AAABABAABBBBBBABAAAABAAABAAA--BBBBBBABBB-AA-AAB-A-B-AA-ABABBAB-AABBABBB-BAB-AB-BBABBBBB

*001337_00193731 BAAAABABAABBBBBBABAAAABAAABAAABBBBBBBBABBB-AAAAAB-ABB-AAAABABBAB-AABBABBB-BAB-ABBB--BBBBB

*001337_00193817 BAAAABABAABBBBBBABAAAABAAABAAABBBBBBBBABBB-AAAAAB-ABBBAAAABABBAB-AABBABBB-BAB-ABBBBABBBBB

*001338_00010913 AABABAABAAABABBAAAA-BBBBABABBAAAAB-BBABBBAABBBBAABBA-BBABABABABA-AABBBAAABABBBB-BBBBABAAA

*001342_00133864 AA-BAAB-AA-BAABBBA-BBABBABBABBBABB-B-AAABBBABABBAB-ABABBAAABBABAAB-AABBABAAABABA-AABB-B-A

*001344_00000907 ABA-AABBABABABBBBAB-AAAABABAABABABAAA-AAAB-BAAABAA-BABBB-BBABB--ABAAABBBBABAABABAAAAA-BBB

*001348_00054691 BBBABBB-ABABBBABAB--BAA-ABBAABB-BABAAB-B-B-BBAABBAAAABABAABBBBBA-BAABB-ABAAAB--AAAABB-BAA

*001348_00129051 BBBABBBB-B--BBAB--B-BAA-A--AABBABA-AAB-B-BABBAABB--A-BABAABBBBBAABAAB-AA-ABA-ABA-AABBB-AA

*001348_00131932 B-BABBBBABABBBABABBABAA-ABBAABBABABAABBB-BABBAABBA-A-BABAABBBBBA-BAABB-ABA-AB-BAAAABBBBAA

*001349_00141928 AAABAAABA-ABB-BAABBABAAABBBBABBBAABBBABAB-B-BBBB-BBA-AABABBAABBABBAB-A-BABBBBAABAAAAAAAAA

*001349_00180643 AAABAA-BA-ABBABAABBABAAABBBBABBBAA-BBABAB-BBBBBBBBBA-AABABBAABBABBAB-ABBABBBBAABAAAAAAAAA

*001350_00164465 ABBABABBAAAAAAAABAABBBA-BA-ABBBBBBBBABBBBBBAAAABBA-BBAABAABAAABABABAAAB-AABAAABB-AAAAB-BB

*001352_00097874 B--B-BABABABBAABBBBBBAAABBAABBAAAB--BBABBBBAABAA-AABA-BBABBAABBBBBBABBBBBBB-BBBBAB-BAAABB

*001353_00289462 B-AAABBBAAB-BBBBABBABBBABABABBB-A-ABBA--BBABB-B--AA-BAB---B-A-BABAB-BBBBBABBA-B-ABBAAB-BB

*001357_00207663 ABBABABAABABABBBBAABAA-BBB-ABAABBB----BBBAABABA-ABABBABBBABAAAABAAAAABBABABA-BBABAAA-AABB

*001360_00110201 BBAAABAAAABBABABBAB-BAA-BAAAAAB-BBBAA-ABBBBBB-BAABABAAABBABBBABABBAA-AAABBBB-BAABA-ABABBB

*001361_00090100 AAAAAAABBABAABBBBAABABBABB-BBBBB-B-AABBAAAA-AAAABBBBBABBBBBA-A-BBBABABBAABBBAABABBBBB-ABB

*001361_00098725 A--AAAA-BABAABBBBAABABBABB-BBB-B--BAABBAAAA-AA-ABBBBBA-BBBBA-A-BBBABA-BAABBBAABABBBBBBABB

*001365_00040007 AAAAA-AABBBAAAB-B-ABAA--AABAAAA-ABA-AA-AAA-BA-BABAABAABABAABABABBAA-A-AAABBABBB-A-ABBAABB

*001368_00035841 BABABBABBAAAABABBA-BBBAB-ABAABBAABABBBBBABBABBAABAAA-BBABABAAABA-BBABBABAABBABB-AAABBAAAA

*001369_00091061 B-BAB-BBBBB-AB-AAABAB-B--A-BABB--A--ABAA-BBAA-BBBBB-BABABABB-AAA-ABBBAABBBBA-B-BB-AA-BB-A

*001371_00033776 B-AAABBBBBABA-AABABBBA-BA-ABBAAABBA--A-BBABAAABBAAAABAABABB-ABAAAABBBBAAAABBAABABAA-ABAAA

*001371_00097809 B-AAABABBBA-A-AABABBBA-BA-ABBAAABBA--A-BBABAAABBA-AA--A-ABB-ABAAAABB-BAAAABBAABA-AAAABAAA

*001372_00008410 A-BABAABAAABBBAABB-BBBBB-ABAAABBABABBBABBB-AAAAABAAA-B-BBABAAABA-BBB-B-B-ABBABBBBAABBB-A-

*001372_00008496 AABABAABAAABBBAABB-BBBBB-ABAAABBAB-BBBABBBAAAAAABAAA-B-BBABAAABABBBBABAB-ABBABBBBAABBBAAA

*001372_00034420 AABABAABAAABBBAABB-BBBBB-ABAAABBABABBBABBBAAA-AABAAA-B-BBABAAABABBBBABAB-ABBABBBBAABBBAAA

*001372_00168679 AABABAABAAABBBAABB-BBBBBAABAAABBABABBBABBBAAAAAABAAA-B-BBABAAABABBBB-BAB-ABBABBBBAABBBAAA

*001374_00131537 AAABAAAABBAABABABBAAA-AABAABB---BB-A-BBBBB-A---BBAABBBAABAABAA-BBBBBBB-ABBAB-A-AA-ABBB-BB

*001375_00270905 AAABAAABAAABB-BAABBABABABBBBABBBAABBBA--B-B-B-BA-BBABAABABBAABBABBAB-A-BABBBAAABBAAAAAAAA

*001384_00104078 BAAA-BAB-AB-BBBB-B-AA-BA-ABAAAB--BBBBBABBB-AA--ABAABBB-A-ABAB-AB-AABBABBBBBAB-ABBBBABB-BB

*001384_00142616 ---AAB-BAAB-B-BB-B-AA-BA-ABAAABBBBBBBBAB-B-AA--AB-ABB-A-AAB-BBAB-AABBABBBBBAB--B-BBA-BBBB

*001385_00055747 BABBBBBABAAB-BAAA--BA-BB--BABAABBABBBABAAB--BAAAA-AABBAABAAAA-AAAABABAABB-ABBBBBABBA-BAAA

*001385_00056442 BABBBBBABAAB-BAAA--BA-BB--BABAABBABBBABA-BA-BAAAA-AABBAABAAAA-AAAABABAABB-ABBBB-ABBA-BAAA

*001386_00063504 BBBABBAAABABBABBBBABAAABAA-ABABBBBBAABBAAAABA-AAABA-ABABBAAAAAABABAAAABAABBAABAABAABBABAA

*001386_00165869 B-BABBAAABABBABBBBABAAABAA-ABABBBBBAABBAAA-BA-AAABA-ABABBAAAAAABABAAAABAABBAABAABAABBABAA

*001390_00037467 BBBBBBAABAABBB-BBBA--AABAABBAAA-AAABAABAAB-AAAA-ABBBAAABBB-ABAAABBA--ABBBBAABBABABBAABABB

*001390_00037553 BBBBBBAABAABBB-BBBA-AAABAABBAAABAAABAABAABAAAA-BABBBAAABBB-ABAAABBA-BABBBBAABBABABBAABABB

*001390_00202620 BBB-BBAABAABBB-BBBA-AAA-AABBAAABA-A-AABAAB-AAA-BABBBAAABBB-ABAAABBA-BABBBBAABBABABBA-BABB

*001394_00173115 AB-AAABBBBBABBBBABBAAAABBBAABABBAAA-ABABAA-BBBBAABA-AA-AB-BABBABB-BAA-AAAAAABABA-BBAABBAA

*001394_00221787 ABAAAABBBBBABBBBABBAAA-BBB-ABABBAAABABABAA-BBBBAABAAAA-AB-B-BBABBABAA-AAAA-ABA-AABBAABBAA

*001395_00186467 BABBBBAAABBAAAABBA-ABAB-BABAAABAAAAAABB-AABAAB-B-BBAAABBAAAABAAAAAAAABBAA-BABABBABBBAABAA

*001398_00165404 BAABABAABBA-BABABBBBAAAABAAB--AB-B-AABBBBABB-AABBAABBBABBAABAAABAABBBBAABBAB-ABAAAAAAB-BB

*001401_00081876 BABABB-BAABBBABA-BABAAAABBBBBABAAAAAABAABAABBABBBABAAABBBBBBBAAAAB-BBBBAAAABAA-AABBBABAAA

*001401_00248651 BABABB-BAABBBABA-BABAAAABBBBBABAAAAAABA--AABBABBBABAAABBBBBBBAAAAB-BB-BA-AABAA-AAB-BABAAA

*001402_00159839 BB--A-AB--ABBBBB-BABBBAB-AAABAA--B-AABAAAB-BBA-AAAAAAAAABABABABABBABABABBAABAAB-A-A--A-AA

*001403_00132984 AABABAABAAABBAAAABBBBBABBA-ABABBABABBBABBBABBAAAAAAABAABBABAAABABABB-A-BBABBBB-BBAAAABBAA

*001404_00057176 AABABABABBBBABBAAABABBBBAABAABBBAAAAABABBBABAA-BBBAABBABB-BBAABAAABBBAAABBBAA-BABAAABBBAA

*001404_00072787 AABABAB-BBBBABBAA-BABBBBAABAABBBAAAAABABBBBBAA-BBBAABBABB-BBAABAAABBBAAABBBAA-BABAAABBBAA

*001404_00074401 AABABABABBBBABBAAABABBBBAABAABBBAAAAABABBBBBAA-BBBAABBABB-BBAABAAABBBAAABBBAA-BABAAABBBAA

*001406_00099689 AABABABABBBBABBAAABABBBBAABAABBBAAAAABABBB-BAA-BBBAABBBBBABBAABAAABBBAAABBBAA-BABA-ABBBAA

*001406_00125498 A-B-BABABBB---BAA--ABBBBAABAA-BB-A---BABBBABAA-BBBAAB-B--ABBAA-AAABBBA-ABBBAAABABA-A--BAA

*001407_00043627 BAABABA-BBA-BABABBBB-AAABA-BB-AA-B-AABB-BA-B-AABBAA-BBABBAABAAA-AABBABAABBAB-ABAAAAAAB-BB

*001408_00146209 A-BABABBAAAAABAAAAABBBA-BAAABBBBBB--AAB-BBBABAABBAABBBBBBA-AAABA-ABABABAAABAABBBAAAA-BBBB

*001415_00076360 A-BBBAA-BABBBABAABABBBA-BAAAB-BBABABBBBB-BBB-AAA-AAABBABBABAAABABBBB-A-BBABA-A-BBAA-AABAA

*001417_00063255 BA-A-BBAABABABBBBAABAAAABB-ABAAABBB--ABB-AABABAAABABBABBBABAAAABAAABBBBABABA-BBABAAB-AABB

*001420_00044983 -BABA-ABAAABAB-ABAABBAABBBBBBBB-BBBAAAAB-BBBBAABABBAAABAABABAABBABB-BAABAABBBAABBBBAABAAA

*001422_00055761 B-BABBABBA-A--AB-BAAAAB-BABBB-A-BB-BBBABABBAB-A-ABAB-BA--BBABBA--BA-B-ABAAAAAAAB-BBB--BBB

*001426_00091934 ABB-BAABAAAAAAAABA---BA--A-ABBBBBBBBABBBAB-AAAABBA-BBAABAABAAABABA--AAB-AABAAABBBAAAAB-BB

*001426_00233985 A-BABAABAAAA-AAABAABBBA-BA-ABBBBBBBBABBBAB-AA-ABBA-BBAABAABAAABABAB-AABAAABAAA-B-AAAABBBB

*001427_00152906 B--BBBABAAABBAABABBABAAA-A-AABBABA-BBABAA--AABABBBAAAAAB-ABABBBAAAABBABBAA-ABBAABBBBAAA--

*001428_00025770 BBBABBAB-AAABB-ABBBABBBB-A-BAABBBBBABABBABAB-AAABBAABBBBBBBABAB-BAABABABAABAAABBB--BBBAAA

*001432_00004177 ABBABAABAAAAAAAABAABBBA-BABABBBBBBBBABBBABBAAAABBA-BBAAAAABAAABABABAAAB-AABAAABBBAAAAB-B-

*001432_00004541 ABBABAABAAAAAAAABAABBBA-BABABBBBBBBBABBBABBAA-ABBA-BBAAAAABAAABABABAAAB-AABAAABBBAAAAB-BB

*001434_00198681 BABBBB-BBABBAABABABB-AAABBBBB-AAAB-AAAABBBAABBBAABAAABABBBBBABBBBBAA-AABAAABAA-BBAAABAAAA

*001435_00117204 BABBBBBBABBBBAABABBAAABAAAABBBBBABAAABBBABAABABBBBBBA-BBABABABABBBBAAABABBBBBAAABBBBBBBBB

*001438_00055320 ABAAAAAAAABAAAABAAAABBABBBBAAABB-B-AA-AAABBBBBBAABABAABAAAAABABABAABABBBBBBAB-BBAAABBABBB

*001438_00056982 ABAA-AAAAABAA-ABAAAA--AB-BBAAAB-BBAAABAAAB-BBBBAA-ABAA-AAAA-BABA-AABABBBBBBAB-BBAAABBABBB

*001438_00076718 A-AA-AAA--BAAA-B-AAA-BAB-BBA-ABB-BA-AAAAAB-BBBBAA-ABA-BA-AAABABA-AA-AB-BBB-ABABBAAA-B--BB

*001443_00112775 AAA-AAA-AABAAABB-A-A-ABBBABAABBBBBA-A-A-BB---BBAAA--ABBA-AAAAABBBAAB-B-BBAB-BBBBAA-BBABBB

*001445_00112389 BAABABAABBA-BABBBB-BAAAABA-BA-AA-B-AABBBBA-B--ABBAABBBABBAABAAABAABBABAABBAB-ABAAAAAAB-BB

*001446_00009270 ABBABAB-AABABABBBBBAABBBAAAABBB-AAABBBBAAAAAABABAAABABAABAABABABBABAABBABBBABBBABAAABBBBB

*001446_00070646 ABBABAB-AABABABBBBBAABBBAAAABBBBAAABBBBAAA-AA-ABAAABABAABAAB-BABBABAABBABBBABBBABAAABBBBB

*001446_00087102 ABBABAB-AABAB-BBBBBAABBBAAAABBB-AAABBBBAAA-AABA-AAABABAABAAB-BABBABAABBABBBABBBABAAA-BBBB

*001448_00021752 A--A-AABABABB-AAABB-BAABABBBABABAABBBAB-ABB-BB-A--AABAABABBAABBABBABBAAAABAAAAAA-AAABAAAA

*001448_00149348 A--A-AABABABB-AAABBABAAAABBBABBBAABBBAB-AB--BBBA--AABAABABBAABBABBABBA-AABBAAAAA-AAABAAAA

*001449_00080873 B---BBBABBBB-B-AAABA-ABBAABBABBBAAAAABAABB-BAB-BBBBABAB-BABB-AAAAABBB-ABBBBAABABBAAABBBAA

*001449_00120639 B-BABBBABBBBABBAAABA-ABAAABBABBBAAA-ABA-BBBBAB-BBBB-B-BABABB-AAAAABBBAABBBBAABBBBAAABBBAA

*001449_00163369 B-BABBBABBBBABB-AABA-AB-AABBABBBAAAAABAABB-BAB-BBBBABABABABBAAAAAABBBAABBBBAAB-BBAAABBBAA

*001451_00047919 BAABABABAABBBBBBBBBABBAAAABAABBABBBBBABBBBABABBBABAB-AABAAAB-ABAABBBAAAABAB-ABB-AAA-BBBBB

*001453_00101869 A-AAAABBAABABBAA-BA-AA-ABB-AAA-A--AAAAAAABA-ABBB-ABAB-AABAAABBBBBABABABABABAAAAAABBAABBAA

*001454_00184605 ABBABAABAAAAAAAABAABBBA-BA-ABBBBBBB-ABBBABBAAAABBAABBAABAABAAABA-ABAAAB-AABAAABBBA-AA--BB

*001456_00101100 B-ABABBAABBBBBABBBBABAABB-AAABBBBBBAABBB-BABBAAABB-A-BABBABBBBBAABBABBB-AABABBBBAAABB-BAA

*001457_00143393 B--ABB--ABABAABBBAABAAABBA-ABA--ABBBBBBBBA-BA-A-AB----ABBABAAA-AA-AAAABAAABAB-AABAAA-AAAA

*001461_00021616 ABAAAABBBBBA-BBBA-AAAABBBB-ABABBAAA-ABABAAAABBBAABAA-A-AB-BABBABBABAA-AAAA-ABABAABBAAB-AA

*001461_00154742 ABAAAABBBBBA-BBBA-AAAABBBB-ABABBAAA-ABABAA-ABBBAABAAAA-ABABABBABBABAA-AAAA-ABABAABBAAB-AA

*001461_00159327 A-AAAABB-BBA-BBBA-AAAABBBB-ABABBAAA-ABABAA-ABBBAABAAAA-AB-BABBABBABAA-AAAA-ABABAABBAAB--A

*001461_00159776 ABAAAABBBBBA-BBBA-AAAABBBB-ABABBAAA-ABABAA-AB-BAABAAAA-AB-BABBABBAB-A-AAAA-ABABA-BBAAB-AA

*001465_00029060 BABABBB-BBBAABBAAABABAB--ABBABBBAAAAABAABBBAAB-BBBBABABABABBAAAAAABBBABBABBAAB-BBAAA-BAAA

*001469_00118507 B-B-BB-BBABBAABABABBBAAABB-B-BAAAAAAAAABBB-AB-BAA-AAABABBBBBABBBBB-A-AABAAABAA-BBAAA-AAAA

*001469_00254288 B-B-BB-BBABBAABABABBBAAABBBBBBAAAAAAA-ABBBAABBBAABAA-BABBBBBABBBBBAA-AABAAABAABBBAAABA-AA

*001477_00001373 BAAAABAABBA-BABABBAAAAAABAABBAAABBBAABBBBBBABAAABBABBBABBABABAABBBBBBBBABAAAAAAAAAABBBBBB

*001478_00022019 BAABABAAABBAAAABBA-ABABAAABAAABAAA-BBBBAAABAA-AAABBAAA-BAAAABAAAAAAAABBABABBBABABBBBAABAA

*001478_00192770 BAABABAAABB--AABBA-ABABAAAB-AAB-AAABBBBAAA-AA-A-ABBAAA-BAAAABAAAAAAA-BBABABBBABAB-BBAAB-A

*001480_00008481 BB-B-BBBAB-AA--ABABBABAAABAAABABABABBBAABBAAABB-BAAA-ABABABAAA-AAB-BBAAAABA-AABAAAAAB-AAA

*001481_00096660 BABABBBBABBAAA---ABAABAABBBBBB--AAABBBABAAAAA-AB-AABABAAABABAAABB-BBAAAABBABBBAABBBAAABBB

*001488_00002062 ABBABAAAB-BABBBBABAA-BAABAABBBABBABBBBBABAABBBBBAAA--ABBBABA-BAABABAABBAABA-BABABAA-ABBAA

*001488_00135615 ABBABAAAB-B-BBBB-B--BBAABAABBB--B-BABBB-BA-BBBBBAAA---BBBABA-BAABAB-ABBAABA-BABABAA-A-BA-

*001493_00254512 ABBBBABBA-ABBAAAABAA-BABAABABB-ABBBBBB-AAAB-B-ABBA-ABAA-BAABBAABBBB-B-BBBABAAAABABBABB-AA

*001494_00148130 ABAAAABBBABBBBABBBABA-AABBBBBBBB-AAAABBB-ABAA-BAAAAABBABABABBAAAAAABAABABABBBBAABBBAABBAA

*001500_00147902 BABABBBAAABBABBBBAA-BBBBAABABBBAABAAABA-BB-AAABBBBAABABBBAABBABAAB-AABBBAABBAAB--BBBBB-AA

*001501_00086602 BBBA-BAABABABAABABAA-ABABABBBAAAAAABBBABABBA-BAAABB-B-ABABB--BAB-BAABAAABABAAAA-ABBBB---B

*001501_00172835 BBBABBAABA-ABAABABA-AABAB-BBBAAAAAABBBABAB-A-BAAABBB-BABABBABBA-BBAABAAABA-AAAA-ABBB-ABB-

*001505_00051122 AB-BAAABA-BA--BAAAAABAAAAABBBAABBABAAAA-AAB-A-ABBA-AAAAABAB-BBBBABBBAABBAAA-BA-BAAABBAAAA

*001505_00086863 A-ABAAABA-BA-A-AAA-ABABAAABBBAABBA-AAAABA-BBA-ABBAAAAAAA-ABABBBBABBB-ABBAAABBAA--AAB-A-AA

*001505_00090603 ABABAAABA-BAAABAAAAABAAAAABBBAABBA-AAAABAA-BABABBA-AAAAABABABBBBABBBAABBAAABBAABAA-BBAAAA

*001505_00093964 ABA---ABA-BAAABAAAAABAAA-ABBBAABB-B-AAABAABBA-AB-A-A-AA--ABABBBBABBBAABBAAABBAABAAA-BA-AA

*001505_00094854 ABABAAABA-BAAABAAAAABA-AAABBBAABBABAAAABAABBABABBA-AAAAABABABBBBABBBAABBAAABBAABAAABBAAAA

*001506_00143535 ABAB--AB--BAAABAA--AB-BAAABBBA--BABAAAABAA-BABABBA-AAAAA-ABABBBBABBB-ABBA-A-BAABAAABBAAAA

*001506_00158600 ABABAAABA-BAAABAAAAABA-AAABBBAABBABAAAABAABBA-ABBA-AAAAABABABBBBABBB-ABBAAABBAABAAA-BA--A

*001506_00174754 ABABAAABA-BAAABAAAAABA-AAABBBAABBABAAAABAABBABABBA-AA-AABABABBBBABB-AABBAAABBAABAAABBAAAA

*001516_00241160 BABBBBAAAAAB-AAAB--BA-BB-BBAAAABBABBBABAABA-AAAAABAA-BAABAAAABAAAABABAABBAABBBBBABBAABAAA

*001517_00101548 -ABABBBBABBB-ABBABAB--BAAAAABA-BBBBAAAAABAAA-BAABAABAAABAA-BAAABB-ABBBBBB-A---BBA-BAABABB

*001519_00036545 B-ABABAB-ABBBBBBABBBBBAAAABABB-AB---BA-BBA-B--BA--A-BAAB-BAB-A-A-BAAAB-ABABAABBAA-AAABBBB

*001521_00042222 A-AB-ABBA-ABBAAAABAAA-ABAABABB-ABBBBBBAAA-B-BBA-AA-ABA-AABABBAABBBB-B-BBBABAAAABABBA-BBAA

*001524_00015362 B--AABBB-AAAAAAB-ABBBAABB-BABBB-ABABBB-BAB-A-BAAA-BA-BBABBAA--AB-AABBBAB-ABA-BB-A-ABBAAAA

*001525_00041969 BAAAAB-BBAABBBAA-BBAAABAAABAAAABBABAAABBBBABBA-AAAABAB-AAAAAB-AAA-ABAAAAAAABB-AABBBAABAAA

*001525_00079464 BAAAAB-BBAABBBAA-BBAAABAAABAAAABBABAAABBBBABBA-AAAABAB-AAAAAB-AAA-ABAAAAAAABB-AABBBAABAAA

*001526_00143224 A-BABAABAAAAAAAABAABBAA-BABABBBBBBBBABBBABBAAAABBA-BBAAAAABAAABBBABAAAB-AABAAABBBAAAABBBB

*001527_00015666 BBBABBABBABABAABABAAAABABABABABBBB-BBBABABBABBAAABABABAAAABAABA-BBAABBABAABABAABABBBBABBB

*001527_00025547 B-BABBABBABABAABABAAAABABABABABBBB-BBBABABB-BBAAABABABAAAABAABA-BBAABBABAABABAABABBBBABBB

*001529_00222254 ABAA-AABAABAAABBAAA-B-BB-ABAABBBBBAAABAABB-BBBBAABABABBAAAAAAABBAAABABBBBABABB--AAABBABBB

*001532_00020336 BBA-AB-BBBB-AABABAABBA-BABBAAAA-B-BAAABAAB-AB-BBBABB-ABABAA-ABBBBAAB--AAABBAAB-AAAABB-BBB

*001534_00015401 BAABABABBBA-BABBBB-BAAAABA-BA-AA-B-AABBBBABB-AABBAABBBABBAABAAABAABBABAABBAB-ABAAAAAAB-BB

*001536_00045890 BBBABBBBAABAABA-AAABABAAABABBB-BABAAABABAA-AABAAABBBBABABBBA-AABBBAAAABABBAB-AAABBBBAA-BB

*001536_00164233 BBBABBBBAABAABA-AAABABAAABABBBABABAAABABAABAABAAABBBBABABBBA-AABBBAAAABABBAB-AAABBBBAAABB

*001540_00047030 B--ABBBBBAABAABBBAABBBBBA-BABABAABA-ABAA-B-AAAABB-A-A-BBBAA-BABA-BAAAABB-ABBAAA-BB-A-B-AA

*001541_00007183 A-AAAAABAA-AAABBAAAABABBBABAABBBBBAAABAABBBBB-BAABABABBAAAAAAABB-AABABBBBABABBBBAAABBABBB

*001541_00007783 ---AAAAB-ABAAABBAAAABABBBABAABBBBBAAABAABBBBBBBAABABABBAAAAAAABB-AABABBBBABABBBBAAABBABBB

*001541_00011389 AAAAAAABAABAAABB-AAABABBBABAABBBBBAAA-AABB-BBBBAABABABBAAAAAAABB-AABABBBBABABBBBAAAB-ABBB

*001541_00021542 -BAAAAABAABAAABBAAAA-ABBBA-AABB-BB-AABA-BBBBBBB-ABABABB-AAAAAABB--A-A--BBABABB--A-ABB-BBB

*001541_00037002 ABAAAAABAABAAABBAAAABABBBABAABBBBBAAABAABBBBBBBAABABABBAAAAAAABB-AABABBBBABABBBBAAABBABB-

*001541_00042650 A-AAAA-BAABAAABBAAAABABBBABAABBBBBAAABAABBBBB-BAABABABBAAAAAAABB-AABABBBBABABBBBAAABBABBB

*001541_00044160 A-AAAAABAABAAABBAAAABABBBABAABBBBBAAABAABBBBBBBAABABABBAAAAAAABB-AABABBBBABABBBBAAAB-ABBB

*001542_00152836 ABBABABBAAAAABAAAAABBBA-BAAABBBBBBBBABBBBB-ABAABBAABBAABBABAAABABABABAB-AABAABB-AAAAABBBB

*001543_00166563 A---AABBBBBA-BBAABB-AA--BBBAAA-BAABAAAABAABBBB-A-BBAB-AAB-BABBABBAAAA-AAAAA-BA-A-A-AABBAA

*001545_00277447 BABBBB-BAAABB-BA-BABAAAABBBBBABAAAAAABAABAABBABBBABAAABBBABBBAAAAB-BB-BAAAABAAAA-B-BABAAA

*001545_00277693 B-BBBB-BAAABB-BA-BABAAAABBBBBABAAAAAABAABAABBABBBABAAABBBABBBAAAAB-BB-BAAAABAA-AABBBABAAA

*001546_00131476 AA-B-ABBABABBAB-BB-BAABBAABABB-ABBBB-A-AAB-ABAABAB--BBBBA-ABBAAAAB-AA-BABAAABAA-AAABBBBAA

*001548_00043442 ABBABAAAB-BABBBBABAAABAABA-BBBA-BABABBBAAAB-BBBBAAABAABB-ABA-BAABBBABBAAABA-BABABAA-ABBBB

*001553_00166458 B-BBBBABABABBAABBBBBBAAABBAABBAAABABBBABABBAA-AA-AABA-BBABAAABBBBBBABA-BBBBABBABA--BAABBB

*001553_00261695 B-BBBBABABABBAABBBBBBAAABBAABB-AABABBBAB-B-AA-AA-A-BA-B-ABAAABB-BBBABABBBBBABBAB---BAABBB

*001557_00196852 AAAAAABBBBBABBBBABBAAA-BBB-ABABBAAA-ABABAA-BBBBAABAAAAAAB-BABBABBABAA-AAAA-ABAAAABB-AB-AA

*001558_00067861 AAA--A--BAA-BB--BBBBBBBBBBBBBAB--A-BBBA-ABBA-BAABBBBBBAB-BBAABAB-BBBBB---A-AAA-AAB--A-ABB

*001559_00040930 BABB--AAAAA--BAA---BAA-BA--AAAA--ABB-ABAAB-ABAAAA-AAB-AABAAAA-AAA-BABAABBAABBBBBABBA-BAAA

*001559_00044306 BABBBBAAAAAB-BAAB--BA-BB--BAAAABBABBBA-AABA-BAAAA-AA-BAABAAAA-AAAABABAABBAABBBBBABBA-BAAA

*001559_00071251 BAB-BBAAAAAB-BAAB--BAABB--BAAAABBABBBA-AAB--B-AAA--A--AABAAAA-AAAABABAABBAA-BBBBABB--BAAA

*001559_00082024 BABBBBAAAAAB--AAB--BA-BB--BAAAABBABBBA-AAB--BAA-A-AA-BAAB-AA--A-AABABAA-BAABBB-BAB-ABBAAA

*001561_00004165 B-BABBBBBBABAABBBAABBBBBABBABABAA-AAABABBBAAAAABBBAAAABBBAA-BABABBAAAABBBABBAAA-BBBAA-BAA

*001562_00023542 B-B-BBAB-ABABAABABAAAABABABABABBBB-BBBABABBABBAAABABA---ABBABBA-BB-AB--BAABA-AABA-B-BAB-B

*001562_00044220 BBBABBAB-A-ABA-BA-A-AA-A-ABABA----BBBBA--B---BAAABAB-BAAABBABBA-BBAABBABA--ABAABA-BBB-BBB

*001563_00167245 B-A-ABAB-ABAAABBAAAABABBBAABBBBBBBAAABBABBBABBBAAAABABBAAAAA-AB--A--ABB-BABABBBBAAAB-ABBB

*001565_00005812 BB-A-BBBAABABBBBABBABBBABABABBBAAAABBAAABBABBABABAABBABBABBBAABABABABBBBBABBAAB-ABBAABABB

*001565_00009461 BB-A-BBBAABABBBBABBABBBAB-BABBBAAA-BBAAABBABB-B-BAABBABBABBBAABABABABBBBBABBAABAABBAAB-BB

*001565_00163929 BBBA-BB-AABABBBBABBABBBABABABBBAAAABBAAABB-BBAB-BAABBABBABBBAABABABABBBBBABBAAB-ABBAAB-BB

*001569_00011390 B-AB-BAB-ABBAB--B-ABB-BABBABBBBBBBB-A-A-ABBBBB-BBBBAB----B-BAABBA-BBAA-BAABBBAAB-AAA--AAA

*001569_00014449 BBABABABAABBAB-ABA-BBABABBABBBBBBBBAAAABABBBBBABBBBABAAAABABAABBA-B-AA-BAABBBAABBAAAAAAAA

*001571_00054847 A-AAAAABAABAAABBAAAA-ABBBAAAAB-BBB-AABAABB-ABB--A-AB-BBAAAAAAABB-AABABBBBABABBBBA-ABBABBB

*001575_00029060 B-A-ABABBAB-A-BABAB-BAAA-BBBBBAAAB-A-AA-BB-A--B-AB-AABABB-BBABBBBBAA-A-BAAABA-BBBAA-B-AAA

*001576_00166660 B-ABABABBBA-B-BBBB-BAAAABABBABAA-BBAAB-BBA-B--ABB-A-B-A-BAABAAABAAB--B-ABBAB-A--A-A-A--BB

*001577_00099168 BABBBB-BAABBBABA-BABAAAABBBBBABAAAAAABAABAABBABBBABAAABBBABBBAAAAB-BB-BAAAABAA-AABBBABAAA

*001579_00093664 B-BBBB--BA-BAABABABBBAAA-B-BBB----A-AAAB-B-ABBB-ABAAA-A--BBB-B-BBBAA-AABAAAB-A-B-A-A-AAAA

*001579_00128599 BABBBB-BBABBAABABABBBAAABBBBBBAAAAAAAA-BBB-ABBBAABAAABABBBBBABBBBBAA-AABAAABAABBBAAAB-AAA

*001580_00012543 BAABAB-BBABBAABABABBBAAABBBBBBAAABAAAAABBBAABBBAABAAABABBBBBABBBBBAA-AABAAABAABBBAAABAAAA

*001580_00250376 BAABAB-BBABBAABABABBBAAABBBBBBAAAB-AAAA-BBAABBBAABAAABABBBBBABBBBBAABAABAAABAAB-BAAABAAAA

*001592_00076119 AAAAAAABAABAAABBAAAABABBBABAABBBBBAAABAABBBABBBAAAABABBAAAAAAABB--ABABBBBABABBBB-AABBABBB

*001593_00146499 B-ABABBBBABBBBABBBABA-AABBABB-BBAAAAABBBBABAA-BAAAAABB-BABABBAAAAAABAABABABBB-BAABBAABBAA

*001594_00048874 A-BBBAAABABABBAAABBBBABBBBAAAB-BAAAAAABBBBABBAAAABAABBBAAABBA-ABBAABBAABBB--B-ABBBBAAABAA

*001594_00204379 AABBBAAABABABBAAABBBBABBBBAAAB-BAAAAAA-BBB-BBAAAABAABBBAAABBA-ABBAABBAABBB-AB-ABBBBAAABAA

*001597_00139574 AABABABABAAABABBABABABBA-BAAABABBBBBBBABBB-BAABAAAAABABBABBAAAABA-B-B-ABBBAAABBBABBBBB-AA

*001597_00171555 AABABABABAAABABBABABABBA-BAAABABBBBBABABBBABAABAAAAABABBABBAAAABA-B-B-ABBBAAABBBABBBBBBAA

*001598_00022150 B-ABABABAABBAB-ABA-BBABBBBBBBBBBBBBAAAABABBBB-ABBBBABAAAABABAABBA-B-AA-BAABBBAABBBBAAAAAA

*001600_00133983 A-BABAABAAAAAAAABAA-BAA-BABABBBBBBB-ABBBAB-AAAABBAABBAAAAABAAAB-BABAAAB-AABAAABB-AAAA--BB

*001602_00017079 BBBABBBBAA-A-BBAA-ABBBBBBBBABBAABBB-AABBBB-ABABBBAABAAB-BABBAAB-AAB-BA--AABBABBBAAABBA--B

*001602_00051090 B-BABBBBAA-A-BBAA-ABBBBBBBBABBAABBBAAABBBBBAB-BBBAABAABABABBAAB-AAB-BA--AABBABBBAAABBABBB

*001605_00089736 BABBBBAABA--B-BABB-AA-BBBA-BBAABAA--BABAABAAAA-AABAAAAAABBAABBAAABB-B-B--BABBBBB-B-A-BAAA

*001605_00108616 -ABB-BAA--B-BB-ABBAA-BBB--B-BAA-A-AABABAAB-AAA---BAA-AAABBAAB-AAABB-BA-BBBABBB-BABBA-B-AA

*001608_00059566 --AAAAA-BAA-B-AA-BBBBBBBB-BBBAB--AB-B-A-AB-A-BAABBB--BABBBBAABABBBB-B--ABA-AAABAABB-AAA-B

*001609_00102057 B-BABBBBBAABAABBBAABBBBBABBABABAA-AAABAA-BAAA-ABBAAAAABBBAA-BABABBAAAABBBABBAA--BBBAABBAA

*001611_00013998 BAABABABAABBBABBBBBABBAAAABAABBABB-BBABBBB-BABBBA-AB-AABAAA--ABAABBBAAAABAB-ABBAAAAABBBBB

*001617_00003121 BBAAABAABABBBBBAABAB-AAAAAAABAA-BBBBBBBABBBAAAABBABAABAAAABABAABB-ABAAABAAAABAABBBBABABAA

*001623_00023972 BBABABBBBABBBBABBBABA-AABBABB-BBAAAAABBBBABAA-B-AAAABB-BABABBAAAAAABA-BABABBB-BAABBAABBAA

*001623_00135821 B-A--BBBBABBBBABBBAB--AABBABB-BBAA-AABB-BABAABBAAAAABB-BABABB-AAAA-BAABABABBB-BAA-B-A-BAA

*001625_00037092 BABABBBBBAABAABBBAABBBBBABBABABAA-AAABAABBAAAAABBAAAAABBBAA-BABABBAAAABB-ABBAAA-BB-AABBAA

*001626_00092240 B-BBBBAAAAAB-AAAB--BA-BB-BBAAAABBABBBABAABA-B-AAABAA-BAABAAAABAAAABABAABBAABBBBBABBA-BAAA

*001636_00057109 B-ABABBAABBBBBABBBBABAA-BBAAABBBBBBA-BBB-BABBAAABB-A-BABBABBBBBAABAABBB-AABABBBB-AABB-BAA

*001637_00073276 BAAAAB-BBABBBBAA-B-AAABAAABAAAABBA-BBAB-BB--BA--AA--A--BABBAABAAA--BAAAAAAABB-AABBB-BA-AA

*001639_00064999 BB-AAB-BBBABA-AABAB-BA-BA-ABBA-A-B-BBA-BBABBAABBAAAA-AAA-B-BABAAABBBBBAAAABBAABABAAAAAAAA

*001644_00026945 ABAAAAABAABAAABBAAA-BBBBBABAABBBBBAAABAAABBBBBBAABABABBAAAAAAABB-AABABBBBABABBBBAAABBAABB

*001644_00142853 ABAAAAABAABAAABBAAAABBBBBABAABBBBBAAABAAABBBB--AABABABBAAAAAAABB-AABABBBBABABB-B-AABBAABB

*001646_00116071 BBBBBBABBAAB-BABBBBAA-ABABABABAAAA-BBAA-BBBB-B-BBBB--AABBBAAAAABA-B-A-B-BBAAB---ABBB-BBAA

*001648_00108742 BABABBBAABABAABB-AAB-AAAB-AABAAABBBB-AABBBABABAAABABAA-BBABAAA-AAABBBBBABBBA--BABAABBAABB

*001656_00065782 BABABBBB-BBBABBAAABA-AB--ABBABBBAA-AABAABBBBAB-BBBBABABABABBAAAAAABBBAABBBBAAB-BBAAABBBAA

*001657_00065233 BBBBBBAAAABBABBAAAAABBA-AABB-A-BABAAABA-BAABABBBABABABBAAABBBBBBAABABBABBA-AA-BA---BBBABB

*001660_00095573 AABBBAAB-ABBBABAABABBBABBAAAB-BBABABBBBBBB-BBAAA-AAABBABBAB-AABABBBBAA-BBABABA-BBAAA-ABAA

*001664_00126313 B-BBBB-BBABBAABABABB--AABBBBBBAAAAAAAAAB-BAABBB-ABAAABABBBBBABBBBB-A-AABAAABAABBBAAABAAAA

*001667_00144534 A--AAA--BAAABBA-BBBBBBBBB-BBBABA-A-BBBABAB-A-BAABBBBBBABB-BAABABBBBBBBBABA-AAABAABBAAA-BB

*001679_00012484 BAB-BB-AAAAB-AAAB-BBA-BB-BBAA-ABBABBB-BA-BA-AA-AAB--ABA---A-ABA-AABABAA-BAA-BBBBA---BBAAA

*001682_00024252 AAA-AAABA-ABB-ABABBABAABABBBABABBA-BBBBBABB-BB-AA--ABAABABBAABBAB-ABBA-ABBAAAAAABAAABAAAA

*001684_00088975 B---BB-BABBAAA-B-ABA-BAABBBBBB--AA--BBABAAAAA-AB-AAB-BAAABAB-AA-BBBB-AAA-BABBB-ABB----BBB

*001685_00001909 BABBBBAAABAAABBABAA-B-ABABABABAAABA-AAABAABBA-BBBAA---ABABBBAABBAB-ABABB-BAAABAB--AABB-AA

*001688_00107822 BBAAABAABAABABBBBAABBBAAAAABBBA-BA-AABBBBABAABAB-A-AAAAAAAAABABBB-ABBBABAAAABAABBBBABABAA

*001693_00106349 BBBBBBBABBBBABAABABBABAABAA-AAAABA-BBABAB--BB-BB-AAAABBBBABABBAABAABABBAA-AABBBA-AABBBBAA

*001697_00229927 B-B-BBAA-AAB--AA--ABA-BBABBAAAABBABBBABAA---AAA-ABA-ABAABA-AAB-AAABABA-BBAABBBBB-BBA-BA-A

*001703_00201404 B-AB-BABAABBAB-ABA-BBABABBA-BBBABB-AAA-B-B-BBBABBB-AB-A---ABAABBABBAAABBAABBBA-BBA--AA-AA

*001705_00087302 -ABB---A--BBABAB-AA-B--AAABAAB-BBBBBBB-BBB-BA-B-BBAAB-BBABBBBABABBBB-ABAA-ABABA-BBBB--AAA

*001708_00050256 AABABABABAAABABBABABABBA-BAAABABBBB--BABBBABAABAAAAABABBABBAAAABA-B-B-ABBBAAABBBABBBBBBAA

*001711_00165547 A-BABABBAA-ABBAB-BB-AABAABBAAB-AAAAAAAB-ABAAABBAAABBBA-BBAAABABBBAB--AAAAABAAA-AABBBBABBB

*001712_00023168 BBABABABAABBAB-ABA-BBABBBBABBBB-BBBAAAABABBBB-ABBBBABAAAABABAABBA-B-AA-BAABBBAABBAAAAAAAA

*001715_00053487 B-AAABBBAAAAAAABAABB-AABBABABBBAA-ABBB-BABBABBA-AABABBBABBAABBAB--ABBBABAABAABBBAAABBAAAA

*001716_00097069 B-AAABABAA-ABABBABAA-ABAAABBA-BA-BABBAAB-B-B--BBAAAABBA-BAAAAAABBBAB-BAA-ABBAB-A-AAABBAAA

*001720_00048197 BBAAA-BABAAABBAAB-ABBBBBBABBBAAAAAABBBABAB-AABAAABBBBBABBBBAABA-BBABB-BABA--AABA-B-B-A-BB

*001726_00232203 BABBBB-BBABBAABABABBBAAABBBBBBAAAAAAAAABBB-ABBBAABAAABABBBBBABBBBBAA-AABAAABAABBBAAA-AAAA

*001728_00168749 AABABAABAAABBAAAABBBBBBBBABABABBABABBBABBBAB-AAAAAAABAABBABAAABABABB-B-B-ABBBB-BBAAAABAAA

*001730_00014718 B-BABBBABBBAABA--AABAAAA--ABBBAAABAAABA-BA-AABABBA-BBABBBABABAABAAAAAABABABBBAAAAAAAAAABB

*001733_00070627 AB-A-AAAAAAABAABBBBABBBAAAAAAABABBBAA-ABBABABB-BBBABAA--BABBBABAB-AABAAABABB-BAABBBAB-BBB

*001733_00136891 A--A-AAAAAAABAAB-BBABB--AAAAAA--BBB-A-A--A-A---BBBABA-----BBBABABBAABA-ABABB-BAABBB-BABBB

*001736_00068737 BA-A-B-BBABBAABABABBBAAABABBBBAAAAAAAAABBBAABBBAABAAABABBBBBABBBABAA-AAB-AABAABBBAAABAAAA

*001736_00161075 BA-A-B-BBABBAABABABBBAAABABBBBAAAAAAAAABBBAABBBAABAAABABBBBBABBBABAA-AAB-AABAABBBAAABAAAA

*001739_00013449 BBA-ABBAAB-BBB-BBBBA-A-BBBAAABBA-B--ABB--B-BB-A-BBA-BBABBA-BBB-AABA-BBB-A-B-BBBB-A-BBABAA

*001739_00222225 BBABABBA-BBBBBABBBBABAA-BBAAABBABBBAABBB-BABB-AABB-A-BABBABBBBBAABAABBB-AABABBBBAAABB-BAA

*001749_00110181 BAABAB-B-BBB-AAAA-BAABA-BAAABABAAAABBABAB-ABBBBB-AAAABABAAAAABABBAABAB-BA-BAABBB-AABBBBAA

*001751_00157181 A-A-AABABAAABBA-BBABBBBBB-BBBAAA-A-BBBABAB-A-B--BBBBBBAB-BBAABABBBBBBBBABA-A-ABAABBB-ABBB

*001752_00193921 BBBABBBBAABABBBBABBABBBABABABBBAAAABBAAABBABBABABAABAABBA-BBAABABABABBB-BABBABB--BBAABABB

*001753_00144247 A-ABAAABABBAAAABBAAABB--ABABBABAA-AAA-AABA-ABBAB-AA-AAABABAAAABB-BBBBAAABAABBBAABBBAAABBB

*001757_00086321 -BA-A-AAABAA-ABAB-BBBAABBBBBABABAAA-BBBAABBBA-BBBBB-A-BAAA-BBB-AABBB-BAA-BA-BBAA--B--AB--

*001761_00005749 B-BBBBAABAABB-BABBAAA-BBBABBBAABAAAABABAAB-AAA-AABAAA-AABBA-BBAAABBABABBBAABBBBBABBA-BAAA

*001767_00032941 AAAAAAABAAB-AABBAAAA--BBBABAABBBBBA-ABAAB-B-BBBAABAB-BB-AAAA-AB--AABABBBBAB-BBBBAAA--ABBB

*001768_00134701 B-BABBBABBBA-BA-BAABAAAA-A-BBBA-AB-AABABB--AA-A-BA-BB-BBBABABAAB-BAAA-BABABBBAAA-B-A-A-BB

*001772_00193603 AABBBAABAAABBBAAAB-BBBBBAABAAABBAB-BBBABBBAAAAAAAAAABB-BBABAAABAB-BB-BAB-ABBABBBBAAABBAAA

*001772_00194653 A-BBBAABAA-BBBAAAB-BB-BBAABAAAB-A--BBBABB-A-A-AAAAAABB-BBABAAABAB-BBB-AB-ABBABBBBAAA--AAA

*001772_00222235 A--BB-ABAAABB--AAB-BBBB-A-BA--B--B-BBBABBB---A-AAAAAB-ABB-BAAABAB--BBB-BBABBABB-BAAABBAAA

*001778_00087017 AA-A-ABBABBBABABBABABAABBBBAABAAAAAAA-ABAAABA-BB-AAABAABAAAA-BBBB-AABBAABAAAAABAABBBBAAAA

*001778_00129902 AA-A-ABBABBBABABBABABAABBBBAABAAAAAAA-ABAAABABBB-AAABAABAAAA-BBBB-AABBAABAAAAABAABBBBAAAA

*001779_00042100 BABBBBAAAAAB-AAAB--BA-BB-BBAAAABBA-BBABAAB--AAAAABAA-BAABAAAABAAAABABAABBAABBBBBABBAA-AAA

*001779_00045422 -ABBBBAAAAAB-AAAB--BA-BB-BBAAAABBA-BBABAABA-AAAAABAA-BAABAAAABAAAABABAABBAABBBBBABBA-BAAA

*001782_00096517 A-BBBAAB-ABAABB--A-AABAAABBAAA-BA-AAABBABBAAAAABBBABBBABBAAA-B-ABAA--AAABAABBB-AABBBA-AB-

*001783_00114226 BBAAABABAAAABAABABAAAABAAABBABBAABAA-BABABBBAAB-AAAABBAABABAAAABBBAB-BBABBBBABAABAAABBAAA

*001783_00167769 BBAAABABAAAABAABABAAAABAAABBABBAABAA-BABABBBABBAAAAABBAABABAAAABBBAB-BBABBBBABAABAAABBAAA

*001785_00117624 ABAAAA-ABBBA-BBBB--AABAAAB-A-ABAB-B-ABBBBAAABBB-ABAAABBBB-BABBAAAABBABBAAABBBABA-BB-AB-A-

*001786_00062707 A-A-AAABAAABB-BAABBA-ABABB-BABBBAABBBAB-BAB-BBBAAAB-B-ABABBAABBABBABBAAAABB-AA-AB-A-AAAAA

*001788_00025193 ABABAABBA-ABBAAAABAAAAABAABABB-ABBBBBBAAA---BBABAA-ABAABABABBAABBBB-B-BBBABAAAABABBA-BBAA

*001788_00076734 A-A-AABBBBBBBAAAABAAAAABBABBBB-ABB-BBBB-BBBBBBABABBBBBB--B-BB-AB-BB-B---BABAAAABABBAA-B-B

*001792_00208593 BBABABABAABBAB-ABA-BBABBBBABBBB-BBBAAAABABBBB-ABBBBABAAAABABAABBA-B-AA-BAABBBAABBAAAAAAAA

*001793_00015413 AABBBABABBBBABABBAAABABAAABAABABBBBBBABBAABBAABBBAAABABBABBB-ABBBBBAB-AABAAA-BABBBBBAAAAA

*001796_00030749 BBAAABBBBBABB-AABBBBBA-BA-ABBAAABBA--A-BBABBAABBAAAABAAA-BB-ABAAABBBBBA-AABBAABABAA-AAAAA

*001799_00113740 ABAAAAAAAABAAAABAAAABBABBBBAAABB-BAAA-AAABBBB-BAABABAABAAAAABABA-AABABBBBBBAB-BBAAABB-BBB

*001800_00118441 AABABAAB-A-BABABAAABBBBBABAB-BAA-BBBBABBBAABBBABABBA-BBABABAB-BAAAABB-BAABABBAB-BBBBABAAA

*001803_00056372 ABBABABBAAAAABAAAAA-BBB-B-BA-B-BBB-AAABBBBBAB-ABBA-BBBB-BABAAABABABABAB-AAB-ABBBAAAAABBBB

*001803_00056458 A-BABABBAAAAABAAAAABBBB-BABABBBBB--AAABBBB-ABAABBAABBBBBBABAAABABABABAB-AABAABBBAAAAABBBB

*001804_00001557 B-ABABABAAABBAABBBA--AAABAABBABBBABBABBAAA-ABAABBBABAABAABBAAABA-ABABBAAAABBAAAABAAABABBB

*001804_00122911 BAAB--AB-AAB-AABBB-BBA---A-BB-B-BA-BA-BAAA-ABAABB-AB-ABAA-BAA-B--ABABBAAAAB-AA-ABAAA--BBB

*001806_00088826 BBBBBBBBABABBBABBBBABAA-ABBAABB-BA--ABBB-B-BBAA-BB-A-BABBABB-BBAABAA-BBAAABABBBAAA-BB-BAA

*001808_00156940 BABABBBBBBABAABBBAABBBBBABBABABAA-AAABA-BBAAAAABBAAAAABBBAA-BABABBAAAABBBABBAAA-BBBAABBAA

*001811_00184235 B-B-BBBBBAABAABBBA-BBBBB-BBABA--ABA-AB-ABB-AA-AB--AAAA-B-AABB-B-BBAAA-B-B-BB-A-B-B---BBAA

*001812_00077164 BBAAABBBBBABB-AABBBBBA-BA--BBAAABBA--A-BBABBAA-BAAAABAAA-BB-ABAAABBBBBA-AABBAABABAA-A-AAA

*001813_00099470 B-BBBBAAAAAB-BAAB-BAAABB-BBAAAABAA-ABABAAB-AA--AAB-A-BAABAAA-BAAAABABAB-BAABBB-B-BBA-B-AA

*001819_00108416 B-AB-BBAAB-BBBABBBBABAABB-AAABBBBB-AABBB-BABB-A-BB-A-BABBABBB-BAABBA-BB-A-B-B-BB-AABBBB-A

*001821_00121716 BABABBBAAABAABA-BAABABAAABABBBABABA-ABABBABAABABABABBABABBBABAABB-AAAABABBAB-AAAABBAAAABB

*001821_00122705 BABABBBAAABAABA-BAABABAAABABBBABABAAAB-BBABAABABAB-BBABABBBABAABB-AAAABABBAB-AAAABBAAAABB

*001826_00159492 BBAAAB-ABAAABBA-BBABABB-B-BBBAAAAAABBBABABBA-BAAABBBBBAB-BBAABA-BBABB-AA-A-AAABAABBB-A-BB

*001827_00070296 AABABABABA-AB-BBABABABBA-BAAABABBBBB-BABBB-BAAB-AAAABABBABBAAAABA-B-BAABBBAAABBBA-BBBBBAA

*001828_00112227 BABBBB-BAAABB-BA-BABAAAABBBBBABAAAAAABAABAABBABBBABAAABBBABBBAAAAB-BB-BAAAABAA-AABBBABAAA

*001830_00034815 BBA-ABBB-BABBAAABBBBBAABAAABBAAABBA--A-AB-BBAABBA-AABAAA-BBBABAAAB-BBBA-AAB--ABABAA--AA-A

*001831_00122081 A-A-AAAABBABBABABBBAAAAABAABB-AB---AABBBBB-B--ABBAABBBAABAABAAABBABB-BAABBAB-ABAAAABB--BB

*001834_00201285 AAABAAABA-ABB-BAABBABAAABBBBABBBAABBBABAB-B-BBBB-BBA-AABABBAABBABBAB-A-BABBBBAABAA-AAAAAA

*001834_00203182 AAABAAABA-ABB-BAABBABAAABBBBABBBAABBBABAB-B-BBBB-BBA-AABABBAABBABBAB-A-BABBBBA-BAAAAAAAAA

*001835_00096947 B-ABABABAABBBBBBBBBABBAAAABAAB-ABBBBBABBBBABABBBABAB-AABAAAB-ABAABBBAAAABAB-ABBAAAAABBBBB

*001837_00037713 ABBABABAABABABBBBAABAAABBB-ABAA-BBBAA-BBBA-BABAAABABBABBBABAAAABAAAAABBABABAABBABAAA-AABB

*001838_00091672 BAABABAAABBAAAABBA-ABABAAABAAABAAAABBBBAAABAAAAAABBAAAABAAAABAAAAAAAABBABABBBABABBBBAABAA

*001838_00183029 BAA-ABAAABB-AAABBA-ABABAAABAAABA-A-BBBBAAABAAAAA-BBAAAABAAAAB-AAAAAAABBABABBBA-A-BBBAABAA

*001841_00077424 BAAAABAAAABAAABBAABABAABBBBAAABBABABB-BAABBBBBBAABABBAAAAAAABAABAAABABABABBAB-ABABBBBABBB

*001842_00070153 BBABABBAABBBBBABBBBABAA-BAAAABBBBB-AABBB-B--BAAABB-A-BABBABBBBBAABBABBB-AABABBBB-A-BBBBAA

*001843_00037866 B-ABAB-BAAB-BBBBBBBA-BAAA-BAABBABBBBBABB-B-BABBBA-AB-AABAAABBABAABBBAA-ABAB--BBAAAA---BBB

*001849_00047217 BABBBBAABAABB-BABBA-AABBBABBBAA-AAAABABAAB-AAA-AABAA-AAABBABBBAAABBABABBBAABBBBBABBA-BAAA

*001850_00012508 A-AAAABBBB-ABBBBABBAAA--BB-ABABBAAA-ABABAABBBBBAABAAAA-AB-BABBABBABAA-AAAA-ABAAA-BBA--BAA

*001850_00044086 A-AAA-B-BBBABBBBA-B-AABBBB-ABAB-AAA--BA-AA-B-BBA-BAA-A-AB-BABBABBABAA-AAAA-AB--AABB---BAA

*001853_00136235 BBBABBAABAAABB-BBBBBBBBB-ABAAABBBBBABABBBBAA---ABBAAABBBBBBABABBBAABABABAABBAABBBBBA-BAAA

*001853_00138784 BBBABBAABAAABB-BBBBBBBBB-ABAAABBBBBABABBBBAA-A-ABBAAABBBBBBABABBBAABABABAABBAABBBBBA-BAAA

*001854_00066999 BB--ABBAABBBBBAB---A--A-B--AAB-BB-BAABBBBBA--AAABBAA-BAB-ABBBBB-ABBABB-AA-B-BBBBAA--B-BAA

*001860_00049915 AB-A-AAAAAAABBABBBBABBB-AAAAAABABB-AA-A-BAAABB-ABBABAA-BBABBBABABBAABAAABABB-BAABBBA-ABBB

*001860_00113046 AB-A-AAAAABABBABBBBABBB-AAAAAABABBBAA-ABBABABB-ABBABAA-BBABBBABABBAAAAAABABB-BAABBBABABBB

*001862_00034590 BB-BABBBAB-AAA-ABABBABAAAB-AABABAB-BBBA-AB-AABB-BAAAA-BABABAAAAAAB-BBAAAABABAABAAAAA-AAAA

*001862_00036367 BB-B-BBBAB-AAA-ABABBABAAABAAABABABABBBAAABBAABB-BAAA-ABABABAAA-AAB-BBAAAABA-AABAAAAAB-AAA

*001863_00126485 ABBABA--ABABBBABABB--AA-ABBAAB-ABABAABAB-B--B---BAAAABABA-BBBB-A-BA-ABBABA-AB--AAAAB--BAA

*001864_00005557 BA---B-A-A--BAAA---BA-B--A-A-A--BAB-BABAAB-ABA-AABAAABAAB-A-A-AAAABABA-BBAABBBBBABBAAB-AA

*001867_00023661 A-----BAAABAAABAAAAAABABA-AABBA-BBBBBBB-AA-ABBABBB-A--AB-AA-A-AB-BBBABABBAAB-BABBBBBBAA--

*001869_00024794 BBAAABABBBBAAABABAABBAABABBAAAAABBBAAABAABAAB-BBBABBAABABAABABBBBABBA-AAABBAABBAAAABBABBB

*001872_00077208 --A-ABABBABBAABABABB--AABBBBBBA-AB-AAAABBBAA-B--AB-AABABB-BBABBBB-A--AABAAABAA-B-AAABAAAA

*001876_00175471 ABABAABBA-ABBAAAABAA-B-B-ABABB--BBBBBBAAAA-BB-ABAA-ABAA-AAABBAABBBB-B-BBBABAAA--ABBA---AA

*001877_00048713 A-BB-ABABBBBABABBAAABA-AAABAABABBBBBBABBAABBA-BBBA-AB-BBABBB-ABBBBBAB-AABAAAABABBBB-AA-AA

*001881_00106865 AABABAAB-AA-BAA-AB-B-BBBBA-ABAB-AB--BBABBBAB--AAAAAABAABBABAAABA-ABBABBB-A-BBB-BBA-A-BAAA

*001886_00061238 B--A-BAAAABBABBAAAAABBAABABBBAAABBA-AAAB-ABBABBBAAAA---AABBBBBBBBABABBAB-AA-ABBABBB---ABB

*001886_00066481 ---AA-AAAA-BA-BAAAAA-BAA-ABB-A-A-B-AAAA-BABBABB-AAAAAB-AA-BBBBB-BABABBABAAABA-BAB-BAABAB-

*001887_00102795 BABABBABBA-BAABAAAAABAAAA-BBB--AABAB-AAB-B-BAA-BABAAABAABAAAABBBBBAA-A-BAABAAAABBAAA-A-AA

*001887_00114849 BA-A--ABBA-BAABAA-AABAAAAAB-B-B-ABA--AABAB-BAA-BABAAA-AA-A-AABBBBBAA--AB-ABAAAABBAAABAAAA

*001889_00136129 BA--AB-BBABBAABAB-BBBA-ABBBBBBAA-B-AAA-B-BAABABBABAAA-ABBBBBABB-BBAA-AABA-ABAABBB--ABAA-A

*001892_00008881 BAABABAAABBAAAABBA-ABABAAABAAABAAAABBBBAAABAAAAAABBAAAABAAAABAAAAAAAABBABABBBA-ABBBBAABAA

*001892_00010602 BAA--BAA-BB--AABBABABABAA-B-AAB--AA-BBBAAA-AAAAA-BBAAAABAAA-BAAAAAAAAB---A-BBABAB-B-A--AA

*001892_00134223 BAABABAAABBAAAABBA-ABABAAABAAABAAAABBBBAAABAAAAAABBAAAABAAAABAAAAAAAABBABABBBABABBBBAABAA

*001898_00039831 B-B-BBAAABBAAABBBAB-B-BAAABBAABAAA-BBBBAAABAAA-AAABAAAA-AAA-BA-AAABAABB-BAB-BA-A-BBBA-B-A

*001909_00051736 -BBAB-BBBAAABABAABABABAABBAAABABBBBAAB-BBBAB--BAAAAABABBABBAABABAAB-BAABBAAAABBBABBBBBBAA

*001909_00070639 -BBAB-BBBAAABABAABABABAABBAAABABBBBAABBBBBAB-ABAAAAABABBABBAABABAAB-BAABBAAAABBBAB-BBBBAA

*001911_00038823 BAAA-BAB-A-AAAAAA-AB-AABAAB-BBA-BBBBBAABAABABAA-BA--B-B-AABAAABB-BBAAAB-AAAAABAABAAAAAABB

*001912_00134769 AAA-AA-BA-AB-B-AA-BAB---BAA-AAB-A--BBABAB---B--B-ABABBABABBAA-BABBAB-B-B-BBABB-BB-AAAAAAA

*001913_00116242 BB-A-BBBAABABB--A-BABBBABABA---AAA-B-AAABB-BBABABAABBA-BA---AA-ABABABB-BBABBAA--A-B-ABABB

*001914_00137127 ABBABABAABBABAABBBBAABAAAAAABBBBAAABBBBBAA-AABABABABAAAABBAB-BABBABAABBABBAABBBABBB-BBBBB

*001918_00120209 B-BABBBBABBAAA-B-ABAABAABBBBBB--AAABBBABAAAAA-AB-AABABAAABABAAABB-BBAAAABBABBBAABBBAAABBB

*001921_00059123 BABBBB-BBABBAABABABBBAAABBBBBBAAAAA-AAABBBAAB-BAABAAABABBBBBABBBBBAAAAABBAA-AABBBAAABAAAA

*001924_00093650 BBAAABAABAABABBBBAABBBAAAAABBBA-BA-AABBABABAA-AB-A-AAAAAAAAABABBB-ABBBABAAAABAABBBBABABAA

*001925_00081475 A-A-AA--BAAABBA-BBBBBBBBB-BBBABA-A-BBBABABBA-BAABBBBBBABBBBAABABBBBBBBBABA-AA-BAABB---BBB

*001929_00166110 AAABAAAABABAABAABAA-BABBBBAABBAABABAAA-BABABBAABAAAABBBBAABBAB-BBAABBAABBAAAABABBBBAA--AA

*001929_00167374 AAABAAAABABAABAABAABBABBBBAABBAABABAAA-BABABBAABAAAABBBBAABBABABBAABBAABBAAAABABBBBAAABAA

*001931_00056952 BBBABBA-ABBA-ABAB-AAAABB-AABAABAAAAAABBBABB-AA-BAA-ABBBAAAAABAAAABAABAABA-BAAAABBBBBB--AA

*001938_00099257 BBAA-BBBBB-BA-AA-ABBBA-BA-ABBAAABBAA-A-BBABAAABBAAAABAABABB-ABAAABBB-BA-AAB-AA-ABAA-AAAAA

*001939_00110967 BABABBB-BBBAABBAAABABAB--ABBABBBAAAAABAABBBAAB-BBBBABABABABBAAAAA-BBBABBABBAABBBBAAA-BAAA

*001940_00051414 BAABABAAAB-AAAABBA-ABABAA-BAAA-AAA-BBBBAAABAAAAAABBAAAABAAAABAAAA-AAABBABABBBABABBBBAABAA

*001944_00181021 B----BABABA-BA-BBBBBBAAA-BAABBAAABABBBABBBBAABAAAAA---BBABBAABBBBBAABBB-B-BABBBB-BBBAAAB-

*001945_00114722 A-BABAAAB-BABBBBABAAABAABAABBBABBABABBBAAABBBBBBAAAA-ABBBABAABAABABABBAAABABBABABAA-AB-AA

*001949_00056357 BABABBAAABBAAAB-AABABAABAABBAABAAA-BBAB-ABBAAAAAAABB-AA-AABABABBAAABABBABABBBABABBBBAABA-

*001950_00129200 BBBBB-AABAABB-BBBBA-AAABAABBA-ABAAAAAABAAB-AA--BABBBA-AABBBABAAABBABBABBB-ABBB-BABBA-BABB

*001957_00050548 BA---BBAAABBABBBBAA-BABBAABABBBAABAB-BAABBAAAABBABAABAA-BAABBABAAB-AABBBBABAAAB-BBBBBB-AA

*001958_00069806 BBBABBAABABABAABABAAAABABABBBAAAABABBBABABBA-BAA-BBBA-AAABBABBA-BBAABBAAAAAAAAAAABBB-ABBB

*001959_00022618 AABBBAABAAABBBAAAB-BBBBBAABAAABBABABBBABBBAAAAAAAAAABBBBBABAAABAB-BBABAB-ABBABBBBAAABBAAA

*001959_00022704 AABBBAABAAABBBAAAB-BBBBBAABAAABBABABBBABBBAAAAAAAAAABB-BBABAAABAB-BB-BAB-ABBABBB-AAABBAAA

*001959_00074378 AABBBAABAAABBBAAAB-BBBBBAABAAABBABABBBABBBAAAAAAAAAABB-BBABAAABAB-BB-BAB-ABBABBBBAAABBAAA

*001959_00074464 AABBBAABAAABBBAAAB-BBBBBAABAAABBABABBBABBBAAAAAAAAAABB-BBABAAABAB-BB-BAB-ABBABBBBAAABBAAA

*001965_00148091 B-A--BBAABBBBBABBBBABAABB-AAABBBBBB-ABBB-BABB--ABB--ABABB-BBBBBAABBABBBBAAB-BBBBAAAB---AA

*001967_00069841 AAB-BAA-AAA-BAAAAB-B-BA-BABABABBA-AB-B---B-BBAA-AAAABAAB-ABAA-BAB-BBBA-BBAB--A--BAAA---AA

*001975_00012319 AAB-BABABBBBABBBAABABBBBAABAABBBAAAAABABBBBBAA-BBBAA-BABB-BBAABAAABBBAAABBBAA-BABAAABBBAA

*001975_00089670 AABABABABBBBABBBAABABBBBAABAABBBAAAAABABBB--AA-BBBAA-BABBBBBAABAAABBBAAABBBAA-BABAAABBBAA

*001976_00120658 BAAAABAAAABAAABBAAAABBABBBBAAABBABABB--AABBBB-BAABABBAAAABAABABA-AABABABABBAB-BBAAABBABBB

*001976_00129718 BA-AABA--ABAAABBAAAABBABBBB-AABBAB-BB--AABBBB-B-ABABBAAAABAABA-AAAABAB-BABBAB-BBAA-BBABBB

*001978_00069627 B--BAB-AABA--AB-A-B-A---A-BAABA--AAB--B--B-BA-BA-AABABBAAAAABA-AABABBBAABBA-BB-AABBBB-BBB

*001984_00063702 BABBBBABABBBBAABABBAABBAAAABABBBABA-ABBAAB-BBAABBBBAAABBBBABABABABBAAABABBBBAAAABBBBBBAAA

*001984_00127810 B-BBBBABABBBBAABABBAABBAAAABABBBABAAABBAABABBAABBBBAA-BBBBABABABBBBAAABABBBBAAAABBBBBBAAA

*001986_00070594 BABABB-BAAB-BABA--ABAAAABB-BBABAAAAAABAABA-BBAB-BA-AAABBBBBBBAAAAB-BBABAAAABAAAAAB-BABAAA

*001986_00070680 BAB-BBA-A-BBB-BA-BABAAAABBBBBABAAAAAABA-BAABBABBBABA--BBB--BBAAAAB-BBABAAAABAAAAABBBABAAA

*001996_00102074 BABA-B-B-AA-ABAABABB--BB-AB--AABBAB-AAA-ABAABAAAAAABBBAA-AAAAABAAAAABAA---AB-BBABBBAABA-A

*001996_00132711 BABABBBBBAABABAABABB--BB--B-BAABBABAAAABABAABA-AA-ABBBAABAAAA-BAAAAABAABB-ABBBB--BBA-BA-A

*001998_00014771 BB-B-BBBAB-AAA-ABABBABAAABAAABABAB-BBBAAAB-AABBBBAAA-ABABABAAABAAB-BBAAAABA-AABAAAAAB-AAA

*002002_00037331 BBABABAB-ABBBBBBABBBABAAAABA-B-ABA-AAAAABAABABAABBAB-AABBB-B-AAABBAAABAABAB-ABBAABBAABBBB

*002003_00002798 B-ABABAAABB-AAABBA-AB-BAAAB-AABAAAABBBBAAABAABABABBAAABBAAAABAAAAAAAAB-AAABBB--AAB---ABAA

*002004_00053324 BAABABABBBA-BABBBB-BAAAABA-BA-AA-B-AABBBBABB-AABBAABBBABBAABAAABAABBABAABBAB-ABAAAAAAB-BB

*002008_00164735 BAAAABABAA-AAABBAAAABAB-BAAABB-B-BAAABBABB-ABBBAAAABA-BAAAAAAABB-AABAB--BABABBBBAAABBA-BB

*002014_00119553 B-ABABAAABBAAAA-BA-ABABAAABAAABAAAABBBBAAABAABABABBAAABBAAAABAAAAA-AABBAAABBBBBAABBBAABAA

*002017_00055088 ABBABABABBBA-BBB--AABBAABBAAAAAAB-BAABBBBAABBBBBAAAA-ABBBABABBAAAABBABBAAAABBABA-BB-ABBAA

*002020_00018186 BAAAABABBBBB-BBBA-AA-AAAAABAAAB-BBBBBBBBBBB-AAABBAABBBAAAABBBBBBBAABAABB-BBAB-ABBBBABBBBB

*002020_00133311 BAAAABABBBBB-BBBA-AA-AAAAABAAAB-BB-BBBBBBBBAA-ABBAABBBAAAABBBBBBBAABAA-B-BBAB-ABBBBABBBBB

*002024_00075707 BABABBBBBAAB-BAAB-BBA-BB--B-BAABBABAAAABABAAB-AAA-AABBAABAAAA-AAAAAABAABB-ABBBB-BBBA-BAAA

*002030_00080608 AA-A-AABABABB-AAABBABAAAABBBABBBAABBBAB-ABB-BB-AB-BABAABABBAABBABBAB-A-AABBBAAAABAAABAAAA

*002031_00153619 AAABAABBABABBABAAB-AAAABAABABBBABBBBBB-AABBBBBABAAAAB-BAA-ABBAAB-BBAB-BABABABAA-AAABBBBAA

*002038_00031180 BABAB--BAABBBABA-BABAAAABBBBBABAAAAAABAABA-BB-BBBABAAABBBBBBBAAAAB-BBBBAAAABAA-AABBBABAAA

*002040_00261770 B-BB-BABABABBAABBBBBBAAABBAABBAAABABBBABBBBAABAA-A-BA-BBABBAABBBBBBABBBBBBBABBBBBBBBAAABB

*002045_00062007 BBAAABBBAABA-BBAA-ABBBBBBBBABBAABBB-AABBBBBABABBBABAAABABAAB-AB-B-BBBAAAAABBABBAAAABBABAA

*002051_00120592 BBBBBBBAABABBBABBBBABAA-ABBA-BBABBBAABBB-BABBAABBB-A-BABBABBBBBAABAABBBAAABABBBAAAABB-BAA

*002059_00124418 B-BABBAAABBBBAB-ABAAABA-BAABAA-ABBBBBABAABAA-ABABAABA-ABAA-AABABBAA-BBABBAABBABBABBBBABBB

*002068_00023252 A-BB-AB-BB-B--A-BAAABABAAABAABA-BBBBBB--B--BA-B-BBAABABBABB-BABABBBBBAB--AABABABBBB-AAAAA

*002069_00142769 B-BBBBAAAAAB-AAAB-ABA-BB-BBAAA-BBABBBABAABA-AAAAABAABBAABAAAABAAAABABAABBAABBBBBABBA-BAAA

*002081_00067862 AABBBAAABABABBAAABBBBABBBBAAAB-BAAAAAABBBBABBAAAABAABBBABABBA-ABBAABBAABBB-AB-ABBBBAAABAA

*002083_00002600 A-A-AAABA-BBB-ABABBABBABABBB--A-BABBBBBBAB--BBBAA-BABAABABB-ABBAB--BBA-ABBAAAAAABA--BAAAA

*002083_00017159 A-AAA-A-ABBB---BAB--BBAB--BBABA---BBBBBBAB--B--AAABAB-AB-BBAABBABA-BBAAABBAAAA-ABAAAB-AAA

*002086_00021803 BABABBBBBABA-AB-B-AAABBABAAAABBBBABAABBBABBAAABBAA-ABABAAAAABAABAAA-BAABBBBAAAABBBBBBBBAA

*002086_00078604 BABABBBBBABA-AB-B-AAABBABAAAABBBBABAABBB-B-AAAB-AA-ABABAAAAABAABAA--BAABBBB-AA-B--BBBBBAA

*002089_00081907 A-AAAAABBABABABBABAAAABAAABABABBBBBBBBABAB--BBAAABABBBAAAABAABA-BAAABBABAABBBAABABBBBABBB

*002100_00013349 B-ABABABAABBBABBBBBABBAAAABAAB-ABBBBBABBBBABABBBA-AB-AABAAAB-ABAA-BBAAAABAAAA-BAAAAABBBBB

*002100_00014622 B-ABABABAABBBABBBBBABBAAAABAAB-ABBBBBABBBBABABBBA-AB--ABAAAB-ABAA-BBAAAABAAAA-BAAAAABBBBB

*002100_00080444 B-AB-BABAABBBABBBBBABBAA-ABAAB--BBBBBABBBB-BABBBA-AB-AABA-AB-A-AA-BBAA-ABAAA--BAAA-ABBBBB

*002101_00033592 BBAAABAAAABABABAABAABBBABABABAAA-B-BBABBBB-BBBBBAAAABBBAABBBBABBBABABAABAAABABABBBBAABAAB

*002101_00043571 BBAAABAAAABABABAABAABBBABABABAAA-B-BBABBBBBBB-BBAAAABBBAABBBBABBBABABAABAAABABABBBBAABAAB

*002101_00090215 B-AAABAAAABABABAABAABBBABABABAAA-B-BBABBBBBBB-BBAAAABBB-ABBBBABBBABABAABAAABABABBBBAABAAB

*002102_00005902 BABABBBAAB-AAAAB-AAA-AAAB-AABA-A-BBB-AABABABA-AAABABAA-BBAABBA-ABABBBBB-BBBA--B-AAABBBABB

*002106_00013640 BBAAABBBBBABB-AABBBBBA-BA-ABBAAABBAA-A-B-ABBAABBAA-ABAAA-BB-ABAAABBB-BA-AABBAABA-AA-AAAAA

*002112_00003986 BAAAAB-BBABBBBAA-BBAAABAAABAAAABBABBBABBBBA-BA-AAA--AB-BABBAA-AAA-ABAAAAAAABB-AABBBBBAAAA

*002112_00004072 BAA-A--BBABBBBAA--BAA--AAABAAA-BBABBBABB-BA-BA-AAAB-A-BBAB-AAAAAA-ABAAAAAAABB-AABBBBBAAAA

*002112_00017360 BAAAAB-BBABBBBAA-BBAAABAAABAAAABBABBBA-BBBA-BA-AAA--AB-BABBAA-AAA-ABAAAAAAABB-AABBBBBAAAA

*002113_00164463 B-B-BBABAABBBABAABABAAAA--BBBABAAA--ABAABAABB-BBBABAAABB-BBBBAAAABABBA-AAAABAA-AABB-AB-AA

*002119_00011770 BBBBBBBAABABBBABBBBABAA-ABAAABBABBB-ABBB-B-BBAAABB-A-BABBABBBBBAABAABBB-AABABB-BAAABB-BAA

*002121_00102400 ABBBBABAAABAA-BAAAA-ABABAAAABBABBBBAABB-AABABBABBB-ABA-ABA-AAAAB---BABABBAABB-ABBBB----A-

*002125_00006978 B-BBBB-AAAABB-BAABABBAA-BBBBBA-AAAAAABAAAAABB-BBB-BA-ABB-A-BBABAAB-BBBBBAAABAABBABB-ABAAA

*002126_00108866 BAABABABBBA-B-BBBB-BAAA-BA-BAAAA---AABBBBABB-AA-BAABB-ABBAABAAABAABBABAABBABA-BAAAAAAB-BB

*002127_00104679 A-ABAA-BA-BB-BAAA-BAAAAABAAA-ABBAABBBABABAAABBBB-AAABBABA-BAABAABBABAB-BB-BAABBBBAABB-AAA

*002130_00044287 ABBBBAB-AB-B-ABABABAAAABABBAA-BBAB-BABABBBAA-BBBAAABAABABBB-AAABAB-BABABBBBBB-B-ABBAAAB--

*002136_00141335 BBAAABBAB-AB--AABBBBBAABAAABBAABBBAAAA-AB-BBAABBAAAABAAABBB-AB-A-BB-BBABAAB-AA-ABAAAAAAA-

*002138_00131890 BAABABABAABBBBBBBBBABBAAAABAABBABB-BBABBBBABABBBABAB-AABAAAB--BAABBBAAAA-ABBABBAAAAA-BBBB

*002140_00016831 B-AB-BABAABBB-BBBBB-BB-AA-B-ABB-B-BBBABBB--BABBBABABA-ABAAA-BABA-BBB--A-B-BBAB-AA--ABBBBB

*002140_00142129 B-ABABABAABBBBBBBBBABBAAAABAABBABBBBBABBBBABA-BBABAB-AABAAAB-ABAABBBAAAABAB-ABBAAAAABBBBB

*002141_00022903 B--BBBAAAAAB-AAAB-A-A-BB-BBAAAABBA-BBABAAB--AAAAABA--BAAB-AAABAAAABABAABBAABBBBBABBA-BAAA

*002142_00060757 B---B-AABABA-AABAB-AAA-AB-BBBAAA---BB-A-A--ABBAAABBBAB-AABBABB-BBBAABBAA-AAAA--AABBBBA-BB

*002144_00068188 A-AAAABAAABABBAABBA-AA-ABB-AAAAA---AAAAAABA---BB-ABAB-A-BAAAABBBBABABABABABAAAAAABBAABBAA

*002145_00147842 -BABABABAAB-BB-BBBBABBAAAAB-AB-ABB--BAB-BBABA---A-AB-AABA-ABA--AA-BB-AAA-AB-ABBAAA-A-BBB-

*002146_00047536 BBBABBAAABABAABBBAABAAABBA-ABAABABBBBBBBBAABA-A-ABA-ABABBABAAAAAAAAAAA-AAABABBAABAAAAAAAA

*002147_00130810 BB-A-BBBAABABBBBABBABBBABABABBBAAAABBAAABB-BB-B-BAABBABBABBBAABABABABBBBBABBABB-ABBA-BABB

*002153_00011598 AAABAAABA-ABB-BAABBABAAABBAAAB-BAABBBABABBB-B-B-BBBABBABABB-ABBABBAB-BBBABBBBABBAAAAAAAAA

*002155_00091042 A-A-AAB-BB-BBBBABBBBBBBBBAB-AAABBA-BBABA-AABBBB-BAAABBABB-BABB-ABAABA--AABAB-B-AA-BBB-AAA

*002157_00073859 --AAA-AB-BBBBBBBABAA-AA--ABAAAB-BBBBBBAB-B-AA-A-BAAB--AAA-BBBBBB-A-BBA-B--B-BA-BBBBA-B-BB

*002161_00068480 BBAAABBBAABA-BBAA-ABBBBBBBBABBAABBBAAABBBBBAB-BBBABAAABABAABAAB-B-BBBAAAAABBABBAAAABBABAA

*002164_00031514 BAAAAB-BBAABBBAA-BBAAABAAABAAAABBABAAAABBBABBA-AAAABAB-AAAAAA-AAA-ABAAAAAAABBBAABBBAABAAA

*002167_00146725 -A-A-BB-BABBAAB-BA-BBAAABABBBBAAA----A-B-B--BB-AABAA----BBBBABBBABAA-AABAAABAABB-AA----AA

*002172_00077408 ABBABAB-ABB-BBABABBABAA-ABBAABBABABAABAB-BABB-ABBABAABABA-BBBBBA-BAAABBABA-A-BBAAAABB-BAA

*002172_00078058 ABBABAB-ABB-BBABABBABAA-ABBAAB-ABABAABAB-BABBAABBABAABABA-BBBBBA-BAAABBABA-A-BBAAAABB-BAA

*002174_00089651 BABBBBBBBAAB-BAAB--BA-BB--BABAABBABAAA-AABA-BAAAA-AABBAABAAAA-AAAABABAABB-ABBBB-ABBAABAAA

*002179_00080099 B-BBBBB-ABABBBABABBABAA-ABBAABB-BABAABBB-BA-BAABBAAA--ABBABBBB-AA-AABB-A-ABABBBAAAAB-BBAA

*002182_00081958 A-A--ABABAAAB-A-BBBBB-BBB-BBBA-A-A-BBB-BAB-A---ABBBBB-ABB-BAAB-BBBBB-B--B--AAAB-ABBA-AABB

*002183_00100869 BBB--BAAABAB-ABBB-ABA--BBAAA-AA-AB-AA-BBBA-B-BAA-BA-BBA-BA-A-A-BAAAAAABAAABA--AA-AAAA-AA-

*002183_00134127 BBBABBAAABABAABBBAABAAABBA-ABAABABBAA-BBBAABABA-ABA-BBABBABAAAABAAAAAABAAABABBAABAAAAAAAA

*002186_00114597 BABABBBAABABAABB-AAB-AAAB-AABAA-BBBB-AABBBABABAAABABAA-BBABAAA-AAABBBBBABBBA--BABAABBAABB

*002191_00012505 B-AAABAAAABAAABBAABABAABBB-AAABBABABB-BAAB--B-BAAB-BBAAA-BAABABA-AABABABABBAB--B-AA-BABBB

*002200_00040951 BBAAABABBBBAAABABAABBAABABBAAAAABBBAAABAABAAB-BBBABBAABABAABABBBBAABA-AAABBAABBAAAABBABBB

*002205_00025761 BBAAAB-BAABBBABBABAAAABAAABAAABBBBBBBBABBBBAA-AAB-ABBBAAAABABBAB-AABBA-BBABAB-ABABBABBBBB

*002206_00132457 BABABBABABBA-ABAB-AAAABB-AAAABBBBABAABBBABBAAA-BAA-ABABAAAAABAABABAABAABBBBAA-ABBBBBBB-AA

*002213_00093893 ABAAAAAAAABABAABBBAABB--ABA-BABAABAAA-AA-AAABBABBAA-AAABABAAAABABBBABAAABAAB-BAABBBAAABBB

*002216_00138458 BA-A-B-BBABBAABABAABBAAABABBBBAAAAAAAAABBBAAB-BAAB-ABBABBBB-ABBBABAA-AAB-AABAABBBAAABAAAA

*002217_00148725 A-A-AAABAABA-ABBAAAABABBBABAABBBBBA-ABA-BB-AB-BAAAABABB-AAAA-ABB-A-BABB-BABA-BBBAAABBABBB

*002224_00034576 AABBBABABBBBABABBAAABABAAABAABABBBBBBABBAA-BAABBBAAABABBABBBBABBBBBAB-AABAAA-BABBBBBAAAAA

*002226_00072666 BAAAABBAAAB-BAABABAABAA-BABAAAB-BBBBB-A-ABBBBB-AAAABAAABBABBBBBBABAABBAABBBBABBABAABBABBB

*002227_00051358 BA--BB-AABBAAABAA-BABBABAABBAABAAA--BABBAB-AA-AAA-BBBAAAA-A-BBBB-AABAB-ABABABABABBB-BABBB

*002227_00062706 BABA-BAAABBAAABAA-BAB-AB--BBAAB--AABB-BB-B-AA---AA-BBAA-A-AABBBBAAABAB-ABABABABABBBABABBB

*002228_00021538 BABABBABAABABAABBBA-AAAABAABBABBB-BBABBABA-ABAABBBABAABAABBA-ABA--AAABAABABAAAAABAAA-ABBB

*002228_00045777 BABABBABAABABAABBBA--AAABAABBABBB--BABBABA-ABAABBBABAABAABBAAABAA-AAABAABABAAA-ABAAABABB-

*002231_00030249 AABABAABAAABBAAAABBBBBABBA-ABABBABABBBABBBBBBAAAAAAAB-ABBABAAABABABB-A-BBABBBA-BBAAAABBAA

*002232_00032150 B-AB-BBBABB--BBBBBBABBAAABB-ABBA-BA-BABBBB-BABBBAA-B--BBAABBBABBA-BBAAABBAAAAB-BBAAABB-BB

*002232_00032236 B-ABAB--ABBBBBB-BBBABB-AABB-AB-A-BA-BABBBB-BABBB-AAB--BBAABB-ABBA-BBAAABBAAAA--B-AAABBBBB

*002233_00094570 ABABA-BBBABBBB-BBB-B-BAABBBBB-BB-AAAABBBBABA-BBAAAAABBBBAB-BBAAAAAABAABAB-BBB-AAABBAABBAA

*002236_00008454 --B-BABBABABBAA-BBBB-AB-AB-BBBA---AAABB-BAAABAB-AABB-AB-ABBABABAB--BBA--AAAABABABB---ABBB

*002236_00021575 -BB-B-BBABABBAABB-BBBAB-ABBBBBA-AAAA-BB-BAAABABBAABBBA-BAB-ABABA---BBAAAAAAABABA-B-AAABB-

*002243_00100840 BBAAABBBBBBBBBABBBBB--AABBABB-BBAAAAABBB-ABA--BABAAABB-BABBBBA-AAAABAABABABBB-BAABBAAABAA

*002245_00002209 BABA-BBB-ABBAABB-AABB-BBABBABAB-ABAAABAABB-AAAABBAAAAABB-AAABABABBAAAABBB-BBAAA-BBBAABBAA

*002245_00004134 BABABBBBBAB-AABBBAAB-BBBABBABAB-ABAAABAABB-AAA--BAAAAABBBAA-BABABBAAA-BBBABBAA-ABBBAA-BAA

*002245_00113261 BABABBBBBABBAABBBAABBBBBABBABABAABAAABAABBAAAAABBAAAAABBBAA-BABABBAAAABBBABBAAA-BBBAABBAA

*002255_00114082 B-B-BB-AAAAAABBBAABBB-A-A-BBBAB-AAAAA--B---ABB--BBBAAAB--A-BBABBBBBB-BBABB--B--B-B-AB-AAA

*002259_00230014 BA-B-BBAABAA-ABAA-BBAAABABBAABABAAABBA-AAB-BABBABAABA-BAA-AABA-AABABB-AABBA-BAAAABBBBABBB

*002260_00005588 BA-BBBAABAABBABABBA-A-BBBABBBA--AAAABAB--BAAAA-AABA--AA-BBABBBAAA-BABABB--ABBB-BABBA-BA-A

*002265_00133029 B-ABABAB-ABBBABBBBBABBAAAABAABBABBBBBABBBBABABBBA-AB-AABAAAB-ABAA-BBAAAABAB-ABBAAAAAB-BBB

*002267_00120189 B-B-BBAAB-AA-B-BBAB-BBBBAABAAAB-ABA-BAA-BB-AB--ABBBA-AB--BBBBABABAABAAB-AABBAA-BBBBAAB-AA

*002268_00111382 A-ABAABBABBBABBBBAB-AAAABABAABABA-AAA-AA-BBBAAABAA-BABBBBBBABBA-ABAAABBBBABAAB-BAAAA-BBBB

*002268_00125638 ABABAABBABBBABBBBAB-AAAABABAABABABAAA-AAABBBAAABAA-BABBBBBBABBA-ABAAABBBBABAABABAAAA-BBBB

*002268_00142323 ABABAABBABBBABBBBAB-AAAABABAABABABAAA-AAABBBAAABAA-BABBBBBBABBA-ABAAABBBBABAABABAAAAABBBB

*002269_00082870 B-BB-B-A-ABBABBA-AAABB-AB-B-BAAAAB-AAAA-BA-BABBBA-ABABB-ABB-BBB-AABABBAB--A---B--A-BBBAB-

*002269_00106496 BBBBBBAAAABBABBAAAAABBAABABBBA-AABAAAAA-BAABABBBAAABABBAABBBBBBBAABABBABAA----BAB--BBBABB

*002271_00011401 B-AAABBBBBABB-AABBBBBA-BA-ABBAAABBA--A-BBABBAABBAAAABAAA-BB-ABAAABBBBBA-AABBAABABAA-AAAAA

*002297_00036872 ABAAAABABBBA-BBBA-AAABAAAB-AAABAAAABABBB-A-ABBB-ABAAABBBB-BABBAAAABBABBAAABBBABABBB-AB-AA

*002297_00042338 ABAAAABABBBAABBBA-A-ABAAAB-AAABAAAABABBBBA-AB-BAABAAABBBB-BABBA-AABBABBAAABBBABAB-B-AB-AA

*002310_00046438 BB-A-BBBAABABBBBABBABBBABABABBBAAAABBAAABBABBABABAABBABBABBBAABABABABBBBBABBABB-ABBAABABB

*002310_00116125 B--AAB-BAABABBBBABBABBB-BABABB--A---BAAA-BA-BAB-BAABBAB-ABBBAABABABABBBBBABBAB--AB---BABB

*002311_00014147 BBAAABABAAABBBBA-BABBBABBAAABAAA-B-AABAAABBBB--ABAAAA-AAB-B-BABAABABABABBAAAAABBAAA-BAAAA

*002311_00092077 BB--ABAB-AABBBBABB-BBB-BB--ABAA-BBBAABAA-BBBBAB-BAAA-AAABBBBBABAABABABABBA-A-ABBA-ABBA-AA

*002320_00045009 A-AAAA--BAAABBA-BBBB--BBB-BBBA---ABBBBABABBA-BA-BBB-BBAB--BAABABBBBBBBB-BA-AAA-AABBA---BB

*002320_00054549 AA-A-A--BAAA-BA-BBBBBBB---BBBABA-A-B--ABAB-A-BAAB-BB-BA-BBBAABABBBBBB--ABABAAA---BBAAABBB

*002320_00122466 AAAAAA--BAAABBA-BBBBBBBBB-BBBABA-A-BBBABABBAABAABBBBBBABBBBAABABBBBBBBBABA-AAA-AABBAAA-BB

*002333_00168866 BB-A-BBBAABABBBB-BBABBBABA-AB-BAAAABB-AABBABBAB-BAABBABBABBBAABABA-A-BBBBABBAAB--BBA-B-BB

*002337_00089857 BBAAABABBBBAAABABAABBAABABBAAAAABBBAAABAABAABABBBABBAABABAABABBBBABBA-AAABBAABBAAAABBABBB

*002337_00127243 BBAAABABBBBAAABABAABBAABABBAAAAABBBAAAB-AB-ABABBBABBAABABAABABBBBABBA-AAABBAABBAAAABBA-BB

*002347_00072805 B-ABABABAABBAB-ABA-BBABABBAAB-BABBBAAAABABBABBABBBBABAAAABBBAABBA--BAA-BAABBBAABBAAAAAAAA

*002353_00041262 BABBBBAAAAA--AAAB--BA-BB-BBAAAABBABBBABAAB-B--AA-BAA-BAABAAAABAAA-BABAABBAABBB-BAB-A-BAAA

*002355_00066871 B-BBBBA-ABAABABB-BA--AB-BABBBABBBBBBABAA-ABBBABABBAAAABABBBAAAAAA-AAABAABBBAAAAABBBBBABBB

*002362_00065026 A-BBB-AABABAABAABAB-B-BBBBAABBA---B--ABBBB-BB-AA-BAABBBAAABB---BB-ABBAABBAA-AAA--BBA--BAA

*002364_00121336 ABBBBAABBAABABABBA-AB-ABAABBBBBAAAABBA-BBB-BB-ABBBBAAAABABAB-ABBA-BBA-BABBAAB-AAABBBBABAA

*002369_00059449 BB-A-BAAAABBABABBABABBB-AAAAAAB-BBBAA-ABBABBBB-AABABAA-BBABBBABABBAAAAAABBBB-BAABBBABABBB

*002370_00020959 B-AAABBBBBABB-AABBBBBAABA-ABBAAABBA--A-BBABBAABBAAAABAAA-BB-ABAAABBBBBA-AABBAABABAA-AAAAA

*002370_00124698 BBAAAB--BBABBB-A-BBB---BABA-BAAABBA--A-BB--BA-BBA-AABAAA-BB-ABAA--BBBB--AABBAA-ABAA-AAAAA

*002372_00029662 BBBABBAAABABAABBBAABAAABBA-ABABBBBBBBBBAAAABA-BAABA-ABABBAAAAAABABAAAABAABBAABAABAABBABAA

*002379_00040654 BABBBBAAABBAAAABBA-ABAB-BABAA-BAAAAAABB-AABAAB-B-BBAAABBAAAABAAAAAAAABBAA-BABABBABBBAABAA

*002380_00129121 B-ABABAAABBAAAABBA-ABABAAABAAABAAAABBBBAAABAAAAAABBAAA-BAAAABAAAA-AAABBABABBBABABBBBAABAA

*002383_00029542 AB--BA-ABBBA-BBBB-AABBAAABAAAAA-B---ABB-BAAABB-AAAAAA-BBB-BABBAAAABBABBAAAA-BA-B-BB-AB-AA

*002386_00037838 BAA-ABBBABB-BAABABBAAABAAAABBBBBBB-AABBBABAABABBBBBBAAABABABABABABB-ABBABABBBAAABBBBBBABB

*002386_00068187 BAA-ABBBABB-BAABABBAAABAAAABBBBBBBBAABBBABAABABBBBBBA-ABABABABABABB-ABBABABBBAAABBBBBBABB

*002387_00043331 AABABAABAAABBAAAABBBBBABBA--BABBABABBB-BBB-BBAA-AAAABAABBABA-ABABABB-A-BBABBBB-BBA-AABBAA

*002389_00111082 BBBBBBBAABABBBABBBBABAA-ABBAABBABBBAABBB-BABBAAABB-A-BABBABBBBBAABAABBB-AABABBBBAAABBABAA

*002393_00073109 BA-BBBABAABBB----BA-A-AABBBBB-BAAAAAA-AABAAB----BAB--A-BBABBBA-A-B-BBAB-AAABAA-AAB-B-B-AA

*002399_00033758 BBBABBAAABABAABBBAABAAABBA-ABAABABB-BBBBBAABABA-ABA-ABABBABAAAAAAAAAAABAAABABBAABAAAA-AAA

*002400_00039266 BAAAABAABBA-BABABBAAAAAABAABBAAABBBAABBBBBBAB-AABBABBBABBABABAABBBBBBBBABAAAAAAAAAABBBBBB

*002401_00081019 ABBABAABAAAAAAAABAABBBA-BA-ABBB-BBBBABBBAB-AAAA-BA--BAABAA-AAA-ABA-AAAB-AABAAA-BB--AAB--B

*002401_00126704 -BBABAA--AAAA-AA-AABBBAA----BBBB--BBABBB-BB---ABBA-BB-ABAAB-AABA-ABAAABA-ABAAA-B-A-A-BABB

*002405_00048618 B-BBBBBB-BABABABB-B--AAA-ABAABA-ABAAA-A--B-BAAAA-A-BABBBBBBAABA--BAAABBBBAB--B--AAAB-BBBB

*002406_00100019 ABBBB-BBAAB-BABBBBBAABBBB--ABBBB-AABBBBAAA-B--ABAAABBBA-BBAABBABAABABBBAB-BABAB-BAABBABBB

*002409_00044930 AABABAABAAABBAAAABBBBBABBA-ABABBABABBBABBBABBAAAAAAABAABB-BAAABABABB-AAB-ABBBB-BBAAAABBAA

*002419_00047915 BAABABBABBAAAABBBA-BAAAAB---A-AA-B-AABBBBAB---ABBABBB-ABBBA-AAABAABBAAAABBAB-AAA-A-AAAABB

*002440_00080488 A-AAAABB-AABABBAAAABB-BB--A-BAA-BBBAAAB-BA-BBBBAAAB---BABAB-AAB-A-ABBBAAAAABABBBBBBBABAAA

*002445_00039864 ABBABAABAAAAAAAABAABBBA-BA-ABBBBBBBBABBBABBAAAABBABBBAABAABAAABABABAAAB-AABAAABBBAAAAB-BB

*002445_00053230 ABBABAABAAAAAAAABAABBBA-BA-ABBBBBBBBABBBABBAAAABBA-BBAABAABAAABABABAAAB-AABAAABBBAAAAB-BB

*002445_00055436 AB-ABAABAAAAAAAABAABBBA-BA-ABBBBBBBBAB-BABBAAAA-BA-BBAABAABAAABABABAAABAAABAAABBBAAAAB-BB

*002449_00004798 BB-BABBAABB--BABBBBABAA-B--AABBBB--A-BBBABA-B--ABBAA-BABBABBBBBA-BBAB-BBAABABBBBAA-BBABAA

*002451_00037918 ABAAAAABAABAAABBAAAABBBBBABAABBBBBAAABAAABBBBBBAABABABBAAAAAAABB-AABABBBBABABBBBAAABBAABB

*002456_00050604 BBBABB-ABBABBBAABBBBBAABB-AABA-BAB-AABAAB-BBBBBBBBABBAAAABB-BBBAABBBABBBBABBBABBBBBABAABB

*002458_00150587 BABBBBBBA-B-BABA-BABA-A-BBBB-A--A--A-BAA---BB--BBAB-AABBB-BBBAAAAB-BB--AA-ABAABAA-BBABAAA

*002460_00007690 B--B-BABABABBAABBBBBBAAA-BAABBAAABA-BBABBBBAABAA-A-BA-BBABBAABBBBBAABBBBBBBABBBB-BBBA-ABB

*002464_00031631 ABBBBABBA-BBBAAAABAA-BABAABABB-ABBBBBBBAAA-ABBABBA-AB-ABBAABB-ABBBB-ABBBBA-AA-ABABBABA-AA

*002469_00136301 BABBBB-BBABBAABABABBBAAABBBBBBAAAAAAAAABBB-ABBBAABAAABABBBBBABBBBBAABAABAAABAABBBAAABAAAA

*002472_00044994 A-AAAABAAABABBAA-BA-AA-ABB-AAA-A----AAAAABA--BBB-ABAB-ABBAAAABBBBABABABABABAAAAAABBAABBAA

*002472_00086678 --A-AABAAAB-BBAA-BAB-AA-BBB-AAB-BA--AAAAAB-BA-BB-ABABAABBAAA-BB-BAB-BABA--BAAA-AAB-A-BB-A

*002472_00141549 A-AAAABAAABABBAAABAAAA-ABB-AAA-A---AAAA-ABA--BBB-ABABAABBAAAAB-BBABABABABABAAAAAAB-AABBAA

*002472_00141635 A-AA-ABAAABABBAA-B--AABABBBAAA-AB-AAAAAAAB--BBBB-ABAB-ABBAAAABBBBABABABABABA-AAAABBAABBAA

*002480_00086359 A-BBBAA-BAABBBABBBAAB-ABAABBBBBAAA-BBAABBB-BB--BBBBAAAABABAAAABBA-BBA-BBBBAAB-ABABBBBABAA

*002484_00027813 AABABAABAAAAABABBA-BBBA--BBAABBBAB-BBBABBB-AA-AABAAA-BBBBABA--BABBBBABAB-ABBABB-AAAB-AABA

*002485_00001177 BABB-BABABA-BAABBBBBBAAA-BAABBAAABABBBA-BBBAABAA-A-BA-BBABBAABBBBBAABBBBBBBABB-B-BBBAAABB

*002488_00138811 B--BABABABABBAABBBBBBAAA-BAABB-AABAB-BABBB-AABAA-A-BABB-ABB-ABBBBBAA-BBBBBBABBBB-BBBAAABB

*002500_00042318 BABABBAAABBBBABBABA-ABA-BAABAA-ABBBBBABAABAA-ABAB-ABABABAA-AABABBAABBBABAAABBABBABBBBABBB

*002506_00059315 B-BABBABBAAAABABBA-BBBAB-ABAABBAABABBBBB-BBABBAABAAA-BBA-ABAAABAABBABBABAABBABB-AAABBAAAA

*002509_00166613 AABABABABBBBABBBAAAABBB-AABA-BBBBABAABABBBABAA-BBBAA-BABBABBAABAAABBBAAABBBAA-BA---ABBBAA

*002516_00009238 -BABA-ABAABBBBB----A-BAAA--AABB---B-B-B-BBABAB-BABAB-AA-AAA-AA-AABBBAAAA-A-AABBAAAAABB-BB

*002521_00066912 BAABABAAAAABBAABBB--BAAABAABBABBBABBABBAAABABAABBBABAABAAB-AAABABABABBAAAABBAABABAAAB-BBB

*002528_00169032 ABBABABBAAAAABAAAAABBBA-BAAABBBBBBBAAABBBB-ABAABBAABBBABBABAAABABABABAB-AABAABBBAAAAABBBB

*002533_00034549 AAAAAA-ABAAABBAABBBBBBBBB-BBBABA-AB-BBA-ABBA-BAABBBBBBABBBBA-BABBB-BBBB-BA-AAA--A-BAAABBB

*002552_00026881 BBAAABAAAABBAAABBABABAA-BA-AAAB-BBBAABABABBBBBBAABABAAAABABBBABBBBAAAAAABBBB-BAABAABBABBB

*002553_00051859 B-BABB-BBAABAABAAAAABAABAABBBBB-ABA--AA-ABABAABBABAAA-AABAAAABBBBBAA-AABAABAAAABBAAABAAAA

*002556_00028891 AAAAAA-BBAAABBA-BBBBBBBBB-B-BABAAA-BBBABABBA-BAABBBBBBABBBBAABABBBBBBBBABA-AAA-AABB-A-ABB

*002559_00006106 BBBABBAA-BABAABBBAABAAABBA-ABAABABBBBBBB-AABABA-ABA-ABABBABAAAAAAAAAAABAAABABBAABAAAAAAAA

*002570_00120626 ABBBBAABAABABBB-BBAAABAA-BBABAABBABAABBBBBABBAB-BBBBBBABBBBB-BAABAAABAABBAAABBBBABBBAAAB-

*002575_00035786 B-ABABABAABBAB-ABA-BBABABBABBBBBBBBAAAABABBBBBABBBBABAAAABABAABBA-B-AA-BAABB-AAB-A-AAAAAA

*002575_00134011 BBABABABAAB-AB-ABA-BBABABBABBBBABB--AAABAB-BB-ABB-B-B-AAABABA-BBAAB-AA-B-ABBBA-BB-AA-AAAA

*002576_00086046 B--B-B-BABABBAABBBBBBAAA--AABBAAABABBBABBBBAA-AAB--BA-BBABBAABBBBBAA---BBBBABB-B-BBBAAABB

*002578_00014930 BABBBBAABA-BB-BABBAAAABBBABBBA-BAAAABABAAB-AA--AABAA-AAABBAABBAAABBABABBBBABBBBBABBABBAAA

*002578_00108087 B-BBBBAABA-BB-BABBAAA-BBBABBBAABAAAABABAABAAAA-AABAA-AA--BAABBAAABBABABBBBABBBBBABBAABAAA

*002580_00106687 AABABA-BAAABBAAAABBBBBBBBABABABBABA-BBAB-B-B-AAAAAAA-AAB-ABAAABABABB---B-ABBBB-BBA-AABAAA

*002586_00054680 B-B--BBBB-BBAABAB-BBB-AABABBBBA--AA--AABBB-AB-BAAB-AABABBBBB-BBBAB---AAB-A-BA-BBBAAAB-AAA

*002594_00007648 BABABBBBBAABBBAA-BBBAABBAABABAABBABAAAABAB-BBAAAA-ABBB-AAAAAA-AAA-AABAAAAAABBBAABBBAABA--

*002594_00044710 BABABBBBBAABBBAA-BBBAABBAABABAABBABAAAABAB-BBAAAA-ABBB-AAAAAA-AAAAAABAAABAABBBAABBBAA-A--

*002597_00013012 B-ABABABAABBAB-ABABBBABABBAABBBABB--AAAB-B-AB-A-BBBA-AAA-BBBAABBABBBAABBAA-B-A-B-AAAAA-AA

*002607_00055794 BBABA-ABAABBBBBBBBBA-BAAAA-AABBABB-BBABB-B-BA-BBABA--AA-AAAB-ABAABBB-AAABABBABBAAAAA-BBBB

*002610_00051157 A-BBBABA-B-BABBBAAAABBBAAABAABBBB-BAABAB-B---ABBBBAAABABAABBAB-AAABABBBAB-BAA-BA-AAABBBAA

*002611_00006570 ABB-BAABAABABBBBBBBAABAA-BBAB-ABBA-AABBBBB-BBABBBBABBBABBBBBAAAABAAABAABBAABBBBBABBBBAAB-

*002614_00027168 B-AAABABAABAAABBAAAABABBBAAABBBBBBAAABBABBBA--BAAAA-ABBAABAAAABB-AABABBBBABABB-BAAABBABBB

*002614_00051822 B-AA-BAB-ABAAABBAAA-BABBBA-ABBBBBBA--BB-BB--BBBAAAAB-BBAAA----BBB-ABABBBB---BBBB-AABB-BBB

*002615_00065603 B-BBBBA-ABABAAABBAB-BAAABBBABBAABBBAABAAAB-AAAAAAA-BABBABBBAABB-BBAA-BBBBABAAB-BAAAB-BBBB

*002626_00076984 B-B-BBBBAABBBABA-BA-AAAABBBB-A-AAAAAABA-BA-BB-BBBABAA-B-BB-BBA-AAB-BB--AAAAB-A-AABBBA-AAA

*002634_00010853 A--AAAABAA-A--B--AAABBBBBABA--BBBB-AABAA--BBB-BA-BAB-BBAAAAAAABB-AABAB-BBABABB-BAAABB-ABB

*002634_00092306 A---AAAB-ABAAABBA--ABBB-B--AABBBBB-AABAAABBBBBBAABABAB-A-A-A--BB-AABABB-B-BABBBB--ABBA-B-

*002638_00033152 BBABABABAABBBBBBBBBABBAAAABAAB--BBBBBAB-BB-BABBB-BAB-AABAAAB-ABAA-BBAAAABABBABB-A-AABBBBB

*002638_00083191 B-ABABABAABBBBBBBBBABBAAAABAAB-ABBBBBABBBBABABBBABAB-AABAAAB-ABAABBBAAAABAB-ABBAAAAABBBBB

*002638_00089151 B-ABABABAABBBBBBBBBABBAAAABAABBABBBBBABBBBA-ABBBABABB-ABAAAB-ABAABBB-AAABABBABBAAAAABBBBB

*002640_00001606 A-ABAAB--AB---ABBB-BA-AABBABB-B---AAAB--BABA-B--AA-A---BABABBAAAAAABAABABABBB--AAB-AA-BAA

*002641_00078067 ABBABAABAABABBBBBBBABBAABBBABBBAAAABBABABBABBABABABBBABAABBBAABABABBBBBBBABBAB-BABBAABABB

*002654_00045213 A-B-BA-AABBABABBBBBA-BABAAAABBB-AA-BBBBBAAAAABABAA-BA-A-BBAB--ABBABA-B-ABBAABBBA-BB-BBBBB

*002657_00076658 AABABAABAAAAA-ABBA-BBBAB-ABAABBAABABBBABABAAA-AABAAA-BBBBABAA-BAB-BBABAB-ABBABB-AAABBAAB-

*002658_00093125 BABABBBAAB-BAABBBAAB-AAAB--ABAAABBBB--BBBAA-ABAAABABAA-BBABAAA-AAABBBBBAABBA--B-BAAB-AABB

*002663_00095810 B--BBB-B-ABBBABA-BABAAAABBBBBABAAAA-A-AABAABBABBBABAAABBBABB-AAAAB-BB-BAAAABAA-AABBB-BAAA

*002664_00048419 AAABAAABABBAAAABBAAAB-B-ABABBABAAB-AA-AA-A-ABBABBAABAAABABAAAABBBBBBBAAABAABBBAABBBAAABBB

*002664_00050223 AAA--AABABBAAAABBAAAB-B-ABABB-B---AAAAAABAAABBAB-AABAAABABA-AABBBBBBBA-ABAABBBAA-B-AAABBB

*002668_00030151 BABABBAAABBAAAB-AABABAABAABBAAB-AAABBABBAB-AAAAAAABB-AA-AABABABBAAABABBABABBBABABBBAAABA-

*002674_00031323 B----BA-ABABBAABBBBBBAAA-BAAB-AAABABBBAB-B-AABAA-ABBAABBABBAABBBBB--B-BB--BA-B--AB-BAAABB

*002674_00044662 B--BBB-BAABBBABA-BABAAA-BBBBBABAAAAAABAABA-BBAB-BABAAABB-ABBBAAAA--BBAB-AAABA--AA--BABAAA

*002684_00082660 BBBABBAAABABAABBBAABAAABBA-ABABBBB-BBB-AAAABA-B-ABA-ABABBAAAAAAAABAAAABAABBAABAABAAAAABAA

*002684_00118041 BBBABBAAABABAABBBA-BAAABBA-ABABBBBBBBBBAAAABA-B-ABA-ABABBAAAAAAA-BAAAABAABBAABAABAAAAABAA

*002684_00118127 BBBABBAAABABAABBBAABAAABBA-ABA-BBBBBBBBAAA-BA-B-ABABABABBAAAAAAAABAAAABAABBAABAABAAAAABAA

*002689_00030312 BBAB-BAB-ABBBABBBBBA---AAABAAB--BB-B-A-BBB-BAB-B-B-B---B-AABBABBA-BBAAAABAAAA-BA-A-ABB-BB

*002699_00004360 BA-B-BAAABABBAABBBBBBAAABBBABBAABBBAAAA-ABBAAAAAAA-BABBABBBAABB-BBAABBBB--BAABAB-AABAABBB

*002703_00086940 BBBABB-BBABBAABABAABBAAAB-BBBBAAAA-AAABBBBAABBBAA-AABBABBBBAABBAAAABBBAA-AABAABABBBA-AAAA

*002721_00012222 BB-A-BBBAABABBBBABBABBBABABABBBAAAABBAAABBABBABABAABBABBABBBAABABABABBBBBABBABB-ABBAABABB

*002732_00029104 AABABAABAAABBAAAABBBBBBBBAAABABBABABBBABB-AB-AAAAAAABAABBABAAABABABBABBB-ABBBB-B-AAAABAAA

*002733_00052891 BABBBBBBBAAB-BAABBBBA-BB--B-BAABBABAAAABABAABAAAAAAABBAABAAAA-AAAAAABAA-B-A-BB-ABBBAABAAA

*002733_00052977 BABBBBBBBAA--BAAB-BBA-BB-BB-BAABBABA-AABABAAB-AAA-AABBAABAAAA-AAAAAABAABB-ABBBBABB-AABAAA

*002733_00057316 BABBBBBBBAAB-BAAB-BBA-BB--B-BAABBABAAAABABAABAAAA-AABBAABAAAA-AAAAAABAABB-ABBBB-BBBA-BAAA

*002733_00064205 BABBBBBBBAAB-BAAB-BBA-BBB-B-BAABBABAAAABABAABAAAA-AABBAABAAAA-AAAAAABAABB-ABBBB-BBBA-BAAA

*002745_00036887 BABABBABAA-BBA-AABAAAAAAA-BBB-BAABA--AABABABAABBABAAABAABAAAABABBBAAAAABAABAAAABBA-ABAAAA

*002745_00036973 BABABBABAA-BB--A-BAAAAAAAABBB-BAABAB-A-BAB-BA-BBABAA--AABAAA-BABBB-A-AABAABAAAAB-A-ABAA-A

*002747_00029129 B-BBBBAB-BABAAABBABBBAAABABABBA-A---ABABABBA-AAAAA-BABBBBABAABB--B-AABBBBABAABABAAABA-BBB

*002749_00008740 -BBABAAABAAAABBBBA-AB-AAA-BBBABAAA-A-AABBB-BBBA-BBBAAABB-AAB-ABBABBBABBABBABB-BAABBABABAA

*002749_00040880 ABBABAAABAAAABBBBA-AB-AAAABB-A-AAAAAAAABBB-BBBABBBBAAABBAAABBABBABBBABBABBABB-BAABBABABAA

*002750_00032878 BABABBBBBABBAABBBAABBBBB-BBABAB-ABA-ABAABB-AAAABBAAAAABBBAA-BABABBAAAABBBAB-AAABBB-AABBAA

*002752_00081130 BAABABAAABBAAAABBA-ABABAAABAAABAAAABBBBAAA-AAAAAABBAAAABAAAABAAAAAAAABBABABBBABABBBBAABAA

*002761_00007607 ABBABAABAABABBBBBBBABBAABBBABBBAAAABBABABBABB-BABABBAABBABBBAABABABBBBBBBABBABBBABBAABABB

*002763_00089637 BAB-BBBB-AABAA-BBA-BBB-B-BBABAB-A-A-ABAABB-AA-AB-AAAAABBBAABB-B--BAAAA-BBABB-AAAB-BA--BAA

*002781_00019515 BAABABABAAABBAABBBA--AAABAABBABBBABBABBAAABAB-ABBBABAABAABBAAABA-ABABBAAAABBAAAABAAABABBB

*002781_00032159 BAABABABAAABBAABB-A-BAAA-AABBABBBABBABB--A-AB-A-BBAB-ABAABB-AABA--BABBAAA-BBAA-ABAAABABBB

*002792_00009411 A-AAAABBAABABBAA-BA-AA-ABB-AAAAA--BAAAAAAB----BB-ABAB-A-BAAABBBBBABABABABABAAA-AABBAABBAA

*002792_00009497 A-AAAABBAABABBAA-BA-AABABB-AAA-A--BAAAAAABA--BBB-ABAB-A-BAAABBBBBABABABA-ABAAAAAABBA-BBAA

*002792_00087911 ABAAAABBAABABBAA-BA-AA-ABB-AAABA--BAAAAAAB---BBB-ABAB-A-BAAABBBBBABABABABABAAAAAABBAABBAA

*002796_00116839 AAABAAABA-ABB-BAABBABAAABBBBABBBAABBBABAB-B-BBBB-BBA-AABABBAABBABBAB-A-BABBBBAABAAAAAAAAA

*002801_00109499 B--B-BABABABBAABBBBBBAAA-BAABBAAABABBBABBBBAA-AA-A-BABBBABBAABBBBBBABBBBBBBABB-B-BBBAAABB

*002822_00034902 B-AAABAA-ABAAABBAAA-BABBBAABBBBBBBAAABB-BBBBBBBAAAABABBAAA----BBB-ABABBBB-B-BBBB-AABB-BBB

*002828_00019921 B-BBBBABAABBBBABABBABAAAAAA-BBBABABBBABA-BAAAB-BBBAAAAABBA-ABBBABAABBABBAABABBAABBBBAAA--

*002829_00010749 B-AAABBBBBABB-AABBBBBA-BA-ABBAAABBA--A-BBABBAABBAAAABAAA-BB-ABAAABBBBBA-AABBAA-ABAABAAAAA

*002831_00023499 ABAAAABABBBA-BBBA-AAAAAAAB-AAABAAAA-AB-BBAAABBBAABAAABBBB-BABBAAAABBABBAAABBBABABBB-AB-AA

*002838_00069633 BBABABBA-BBBBBABBBBABAA-B-AAABBBBBBAAB-B-B-BB-AABB-A-BABBABBBBBAABBABBB-AABABBBBAAABB-BAA

*002840_00007060 B-ABAB-B-A-B---BABBB-BAA--BA-B--BBBAAAABB--BABBAA--BB-ABABAB-ABABBAAABAABABBAB-AA-BA-B-BB

*002841_00124080 BA-BBBABABABBAABBBBBBAAA-BAABBAAABABBBABBBBAA-AA-A-BA-BBABB-ABBBBBBABBBBBBBABBBB-BBBAAABB

*002844_00101748 BABBBBAAAAAB-AAABAA-AABB-BBAAA-BBABBBABAABA-BAAAABA--BAABAAAABAAAABABAABBAABBBBBABBAABAAA

*002856_00019137 B-BBB---BAB-AABA-ABB-AAABBBBB-AAA-AAAAABBB-AB--AABAAA---BBBBA-BBBB--AAABAAABAABB-A-A-A-AA

*002856_00020689 BA-BBB-BBABBAAB-BABB-AAA-BBBBBAAAA---AA-BB-A-B-AABA-ABAB-BBBABBBBBAABAABA-ABAA-B-AAABAAAA

*002862_00075498 BABABBAABBBAAABBAAAABB-BBAABBABB-BABBBAABA-BA-BBAAABAABAAABAAABBAA-AABABABBBBAABAAABB-ABB

*002862_00090463 BABABBAABBBAAABBAAAABB-BBAABBABB-BABBBAABABBA-BBAAABAABAAABAAABBAAAA-BABABBBBAABAAABB-ABB

*002869_00031059 BB-A-BBBAABABBBBABBABBBABABABBBAAAABBAAABBABBABABAABBABBABBBAABABABABBBBBABBAB-BABBAABABB

*002873_00060907 B-AAABA-AABBBBBBABAAAABAAABAAABBBBB-BBABBBBAAAAAB-ABBBAAAABABBAB-AABBABBB-BAB-ABBB-ABBBBB

*002873_00060993 BAAAABABAAB-BBBBABAAAABAAABAAABBBB-BBBABBBBAAA-AB-ABBBAAAAB-BBAB-AABBABBB-BAB-ABBBBABBBBB

*002874_00031401 B-BA--BAAB-B-ABBABAAABA-BAB-AAA-BB-B-ABAABBA-A-AA-ABABABAA-A-BABAAAB-BABAAABBBB-BBBB-ABBB

*002875_00008143 BAAAABAAAABAAABBAABABAABBBBAAABBABABB-BAABBBBBBAABABBAAAAAAABABBAAABABABABBAB-ABABBBBABBB

*002884_00045085 A-BABABABAAABABBABABABBA-BAAAB-BBBBB-BAB-B-BAABAAAAAB-BBABBAAAABA-B-B-ABBBAAAB-BABBBBBBAA

*002884_00049558 AABA-A-AB-AABABBABABA--A--AAAB-BBBBBBBABB--BAABAA--AB-BBA-BAAAABA--BB-ABBBAAABBBAB-BB-BAA

*002885_00037692 B-B-BBBA-BABAABB-AAB-AAA--AABA-AB--BAAABBB-BABAAABABAA-BB-BAA--AAABBBBBA-BBA---ABA-BBAABB

*002885_00056767 BAB-BBBA-BABAABBAA-BB-AA--AABAA--BBB--A-BB-BABA-A-ABAABBBABAAAA-A-BB-BBAB-BA--BABAABBA-BB

*002888_00058051 BAABABABAABBBBBBB-BAB-AAAA-A-BBABBBBBABBBB-BABBBABAB-AABAAAB-ABAABBBAAAABA--ABBAAAAABBB-B

*002893_00007344 BABBBBA-AAAB--AABABBA-B--BBAAA-BBABABABAAB-AAAAAABAAABAAB-AAABAAAABABA-BBAABBB--ABBAABAAA

*002900_00105503 BA-B-BB--BBBBABBABBBABBA---A-A--BBBAAA-----A-BAA-AA---ABABABAAAABBA-ABBBBAA--BB-ABBAAABBB

*002908_00074035 BAABAB-BBABBAABABABBBAAABBBBBBAAABAAAAABBBAABABBABAAABABBBBBABBBBBAA-AABAAABAABBBAAABAAAA

*002923_00033955 BA-A-B-BBAABBBAA-BBAAABAAABAAAABBABAAABBB-ABBA-AAAABAB-BAAAAB-AAA-ABAAAAAAABB-AABBBAAAAAA

*002933_00008537 AAAAA-B-BBBABBBBABBAAABBBB-ABABBAA--ABABAA-BBBB-ABAAAAAAB-BABBABB-BAA-AAAA-ABA-A-BBAABBAA

*002933_00095025 AAAAAABBBBBABBBBABBAAA-BBB-ABABBAAA-ABABAABBBBBAABAAAAAAB-BABBABBABAA-AAAA-ABA-AABBAABBAA

*002939_00033717 AABA-AAB-A-BABABAAABBBBB-B-B-BA----BBABB-A--BBA--BBA--BABABA--BAAAAB--BAABABBAB-BBBBA--AA

*002945_00055335 B-BBBBBAABABBBABBBBABAA-ABBAABBAB-BAABBBA--BB-A-BB-A--ABBABBBB-AA-AA-B--AABABBBBAA-BBABAA

*002958_00060961 BBBBBBAABAABBB-BBBA-AAABAABBAAABAAAAAABAAB-AA--BABBBAAAABB-ABAAABBBBBABBBBABBBBBABBA-BABB

*002968_00052693 AAAAAABB-AABABBAAAABBBBBBA--BAAABBBAAABBBAABBBB-AABA-BBABABAAABAA-ABBBAAAAABABB-BBBBABAAA

*002974_00097783 B-A-AB-AA-BAAAABBAAA-ABAAAB-AA--AA--BBBAAA-A--AAA-BAAAAB--A-BAAAAAA-ABBABAB-BA-AB-BB--B-A

*002979_00056870 A-BBBAAA-ABABBBAABABBABBBBAAABABAAA-AABABB-B-AAABBAA-BBBBAB-AAAABABBBAABBBAAB-ABBBBAAABAA

*002984_00011253 BBBABBAAABABAABBBAABAAABBA-A-AABABBBBBBB-A-BABA-ABA-ABABBABAAAAAAAAAAABAAABABBAABAAAAAAAA

*002995_00020322 B--A-BBBAABABB--ABBABB-A---ABBBAAAABBAAABB-BB-B-BAABB-BBABBB-ABABAB--BBBBAB-A-BBA-BAAB-BB

*003006_00014692 BABABBBAAB-AAAABBAAA-AAAB-AA-A-A-BBB-BABABABA-AAABAAAA-BBAABBA-ABA-BBBB-BBBB--B-ABBBBBA-A

*003017_00051059 AAABAAABA-AB-BAAA-BABAAABAAAAABBAABBBABAB-A-BBBB-BBABBABA-BAABBABBAB-B-B-BBBBABBAAAAAAAAA

*003018_00025772 BABABBBBBAABAABBBAABBBBBABBABABAA-AAABAABBAAA-ABBAAAAABBBAA-BABAB-AAAABBBABBAAA-BBBAABBAA

*003021_00034898 AAAAAABBAABABBAA-BA-AA-ABB-AAA-A--BAAAAA-BA---BB-BBAB-AABAAABBBBBABAAAAABA-AAAAAABBAABBAA

*003025_00090144 BB-BABABAAABABBABAA-BAABBBBBBBB-BBBAAAABABBBBAAB-BBA---AABABAAB----ABA--AAB-BAABB-BAABA-A

*003028_00036492 BA-AABABBABAAAB-AAAABABBBAABBBBBBBAAABBABBBBBBBAAAABABBAAAAAAABB-AAAABABBABABABB-AAB-AB-A

*003044_00080340 BABABBBBBAABABAABA-B-BBBB-B-B-A-BAB-AAABAB-AB-A-ABABBBAABAAAA-BAAAAABAABBBABBBBBBBBABBAB-

*003047_00002363 -AAAA-B-BA-ABBA-BBBBBBBBB--B-ABABA-BBBABAB-A--AABB-BB--BBB-AABABB-BBBBBABA-AAA--AB-AAA-B-

*003051_00123793 BBBABBAAABABAA-BBA-BAAAB-A-ABA-BABB--BBBBA-BA--AABA---AB-AAAAA-AAAA-AABAAABABBAA-A--AAA--

*003056_00030030 BBAAABAABBAABBAAABABBBBBBABBBAAA-AABBAB--AA-BBA-BBAABBABAAB-BBBAAAABB-AABAABB-BABBBAABAAA

*003063_00003442 ABBABAAAB-BABBBBABAA-BAABAABBBA-BABABBBABA-BBBBBA-A--ABBBABAABAABABAABBAABA-BABABAA-ABBAA

*003068_00048406 BABABBBBABBAAA-B-ABAABAABBBBBBB-AAABBBABAAAAABAB-AABABAAABABAAABB-BBAAAABBABBBAABBBAAABBB

*003071_00058170 -ABABAABAA-BBAA-ABBBB-BBB-BAB-BBABABBBABBBAB--A-AAAABAA-BAB-AABABABB-B---ABBBBBB---AABAA-

*003071_00076620 AA-ABAA-AAAB-AAAABBB---BBA-ABABBA-A-BBA---ABAAAAAAAABAAB-ABAAABABABB---BAABB--A-BAAAA-AAA

*003079_00042047 AA-B-AABAAABBBAABB-BBB-B-A-AAABB-BABBBABBB-AA-----AAABBBB-BAAABAB-BB-BAB-ABB-BB-B-ABB-AAA

*003084_00022507 BB-ABBBBAABABBBB-BBABBBABABABBBAAAA-BA-ABB-BBABABAABBABBABBBAABABABAB-BBBABBAAB-ABBAABABB

*003086_00008856 BABBBB---ABBBABA-B-BA-AABBBBBAB-AAAAABAABA-BB-B-BAB-AABBBABBBAAAABBBB-BAAAABAA-AABBBABAAA

*003087_00101374 B-ABA-ABAA--BBBBBBBABBAAAAB-ABBABBBBBABBBB-BABBB--A-AAA-AAAB-ABAABBBAAAABABAABBA-AAA-B-BB

*003088_00002576 BB-A-BAAAABABBABBBBABBB-AAAAAA-ABBBAAA-BBABABBBABBABAA-BBABBBABABBAAAAAABABB-BAABBBA-ABBB

*003088_00106791 BB-A--AAAABABBABBBBABBB-AAAAAABABBBAAAABBABABB-ABBABAA-BBABBBABABBAAAAAABABB-BAABBBABABBB

*003094_00003084 A-BABABABBBA-BBB--AAB-AABBAAAAAAB-BAABBBBAABBBBBAAAA-ABBBABABBAAAABBABBAAAABBABAA-BBABBAA

*003109_00052778 B----B-BA--B--AB-BBB--AABBAA-BAAABAB-BABBBBAABA-BABBA-BBAB-AAB-BBBAA-BB-B-BABBB--BBB-A-BB

*003130_00025836 AABABAABAAABBAAAABBBBB--BABA-AB-ABA-BBABBB-B-AAAAAAABAABBABAAABABABBAB-B-ABBBBAB-AAAABAAA

*003146_00070287 AABABAABAAABBAAAABBBBBABBA-ABA-BABABBBABBB-BBAAAAAAABAABBABAAABABABB-A-BBABBBABBBAAAABBAA

*003163_00188537 B-BBABABABABBAABBB----AA-B-ABBAAA---BBAB-BBAA-AA-AABA-BBA-BAABBBB-AABB-BBBBAB-BB-B-BAA-BB

*003166_00075269 BBABABABAABBAB-ABAABBABBBBBBBBB-BBBAAAABABBBB-ABBBBABAAAABABAABBA-B-AA-BAABBBAABBBBAABAAA

*003170_00046751 A-B-BA-B-ABABBB-BBA-ABAA-BBABAABBABAABB-BBABBAB-BBBBB--BBB-B-BAA-AAAB-ABB--ABBBB-BBBA-ABB

*003204_00008493 BABABB-BAABBBABA-BABAAAABBBBBABAAAAAABAABAABBABBBABAAABBBBBBBAAAAB-BB-BAAAABAAAAABBBABAAA

*003206_00034514 B--B-BABAB--BAABBBBBBAAABBAABBAAABAAAB-BBB-AA-AAAA-BABBBBBBAABBBBBAABBBB--BA-BBB-AABAAABB

*003220_00029126 BABABBBBBAABAABBBAABBBBBABBABABAA--AABAABBAAA-ABBAAAAABBBAA-BABAB-AAAABBBABBAAA-BBBAABBAA

*003222_00051159 B-BABBBAABAAAAAB-AAB-AAAB-AABAA-BB-BAAABBBABABA-ABABA--BBABB-A-AAABBBBBABBBA--BA-AABB-ABB

*003225_00055877 AABABAABAABBBABAABBBBBABBA-AB-BBABABBBBBBB-BBAAA-AAABAABBABAAABABBBB-A-BBABABABB-AAAAABAA

*003227_00002830 B-BABBAABAAABBAABBBBBABB-ABBAABBBBBABABBBBAB-ABABBAA-ABBBBBABAB-BAABABABAABBAABBBBBBBBAAA

*003230_00026689 BAABABAAABBAAAABBA-ABABAA-BAAABAAA-BBBBAAA-AABAAABBAA--BAAAAB-AAAAAAABBAAABBBA-AABBBAABAA

*003237_00058810 A-AA-ABAAAB---AA-BA-AA-ABB-AAAAA-B--AAAAABA-BBBB-AB-BAA-B-AAABBB-ABABABAB-BAAAAAAB-AA-BAA

*003239_00001991 B-BABBAAABBBBABBABAAABA-BABBAAAABBBBBABAABAA--BAA-ABABABAA-AABABAAABBBABAAABBABBBBBB-ABBB

*003240_00006722 B-BBBB-AAAAB-AAAB-BBABBB--BAAAABBABBBABAAB--BA--ABAA-BAABAAAA-AAAABABAABBAABBBBBABBA-BAAA

*003247_00026909 AAAAAABBBBBABBBB-BBAAA-BBB-ABABBAAABABA-A-BBBBB-ABAAAAAABABABBABBABAA-AAAA-ABA-AA-BAABBAA

*003247_00026995 A-AAAABBBBBABBBBABBAAA-BBB-ABABBAA--ABABAA-BB-BAABAAAAAAB-BABBABBABAA-AAAA-ABA-AABBAABBAA

*003249_00065723 BABABBBBABBAAA-B-ABAABAABBBBBB-AAAABBB-BAAAAABAB-AABABAAABABAAABB-BBAAAABBABBBAABBBAAABBB

*003254_00088305 BABAB-BBBAABAABBB-ABBBBBABBABAB-A--AABAABB-AAAABBAAAAABBBAA-BABABBAAAABBBABBAAA-BBBAABBAA

*003256_00058402 BABB-BAA-AABA-A-B--B-A-B---AAAA--ABBBABAAB--B-AAAB-AABAABAAAABAAAABAB-ABBAABBB-BABB--BAAA

*003273_00062418 A-AAAA--BAAABBA-BBBBBBBBBABBBABA-A-BBBABABBA-BAABBBBBBABBBBAAB-BBBBBBBBABA-AAABAABBAAA-BB

*003278_00054706 B-A-ABB-ABABBBBBBBBB--AABBB-AABABBBBBAAABB-B-BBBBA-A-A-BB-BBBAABAAAB-AAAB-B-AB--ABB--A-AA

*003281_00085755 A-AAAA-BAABABBAA-BA-AA-ABB-AAA-A-A-AAAAA-B---BBB-ABA--A-BAAAABBBBABABA-ABABAAAAAABBAAB-AA

*003303_00092283 BBABABBAABBBBBABBBBAAABABABBABBB-B-AABBB-BABBAAABBAA-BABAABBBBBAABBABBB-A-BABBBB-AABB-BAA

*003305_00035153 AAAAAAA-AA-AAAB-AAAA-A-B-AB-ABB-BB-AABAABBBA--BAAAAB--B-AAAAAABBBAABA---BAB-BBBB-AA-B-BBB

*003331_00049374 BAABABBABBAAAABBBAABAAAABB-BAAAA-B-AABBBBABB-AABBABBBBABB-B-AAABAABBAAA-BBABBA-AAAAA-ABBB

*003340_00122076 BB-AABBBBBABB-AABBBBBA-BA--BBAAABBAA-A-BBA-BAABBAAAABAAA-BB-AB-AABBBBBA-AABBAA---AAAAAAAA

*003341_00087067 BABBBBAABA-BB-BABBAAA-BBBABBBAABAAAABABAABAAAA-AABAA-AAABBAABBAAABBABABBBBABBBBBABBA-BAAA

*003349_00001501 BB-B-BABABABBAABBBBBBAAA-BAABBAAA-ABBBABBBBAABAA-A-BAABBABBAABBBBBAABBBBBBBABBBB-BB-AAABB

*003355_00010026 BBBABBBBAA-A-BBAA-ABBBBBBBBABBAABBBAAABBBB-ABABBBAAAAABABAABAAB-A-B-BAAAAABBABBBAAABBABAA

*003355_00030243 BBBABBBBAA-A-BBAA-ABBBBBBBBABBAABBBAAABBBBBABABBBAAAAABABAABAAB-A-B-BAA-AABBABBBAAABBABAA

*003355_00032003 BBBABBBBAA-A-BBAA-ABBBBBBBBABBAABBBAAABBBB-ABAB-BAA-AABABAABAABBA-B-BAA-AABBAB-B-AAB--BAA

*003360_00017060 AAB-BAABAAABBBAAAB-BBBBBAABAAABBAB-BBBABBBAAA-AAAAAABBABBABA-ABAB-BB-B---ABBABBBB-AA--AAA

*003360_00018441 AABBBAABAAABBBAAAB-BBBBBAABAAABBABABBBABBBAAAAAAAAAABB-BBABAAABAB-BB-BAB-ABBABBBBAAABBAAA

*003360_00020084 AABBBAABAAABBBAAAB-BBBBBAABAAA-BABABBBABBB-AAAAAAAAABB-BBABAAABABBB--BAB-ABBABBBBAAABBAAA

*003371_00133257 B--BABAAABBAAA-BB--AB-BAAABAAA--AAABBBBA-ABAAAAAABBAA-ABAAA-BAAAAAAA--BABABBBABABBBBAABAA

*003374_00130443 BABABBBBBAABAABBBAABBBBBABBABABAA-AAABAABB-AA-ABBAAAAABBBAA-BABABBAAAABBBABBAAA-BBBAABBAA

*003374_00130529 BABABBBBBAABAABBBAABBBBBABBABABAA-AAABAAB-AAAAABBAAAAABBBAA-BABABBAAAABBBABBAAA-BBBAABBAA

*003380_00077844 B--AABBAB-ABBAAA-BBBBAA-A-ABBAABBB-AAA-ABABBAABBA-AA-AAA-BBAAB-AAB-BBBAAAABBAABA-AA-AAAAA

*003384_00045371 ABBABABAABABABBBBAABAAABBBBABAABBBBAA-BBBAABABA-ABABB-BBB-BAA-AB-AAAABBABABA-BBABAAA-AA-B

*003385_00059447 ABBABAA-AABABBBBBBBABBAABBBABBBAA---BABAB-ABBABABABBAAB-ABBBAABABABBBBB-BABBAB-BABBAABABB

*003387_00030563 BBA-ABABAABBBBBBBBBABBAAAABAABBABBBBBABBBBABA-BBABABA-ABAAAB-ABAABBBAAAABABBAB-AAAAABBBBB

*003390_00014239 BBABABABAABBAB-ABA-BBABABBAABBBABBBAAAABABBABBABBBBABAAAABBBAABBA-BBAA-BAABBBAABBAAAAAAAA

*003396_00046968 B-AA-BA-BBB--BBAA-A-AAAAA-B-AAB-BBBBBB-B-B-AA-A-BAAA-B-AAABB-BBBB-ABAABBBBBABA-B-B---BBAA

*003401_00042444 A-AAAABAAAB-BBAA-BA-AA-ABB-AAAAA----AA-AAB----BB-ABAB-ABBAAAABBBBABABABABABAAAAAABBAABBAA

*003419_00020271 A-A-AA---AAABBABBBBB--BBB-BBBABA-A-BBBABA--A-BAABBBBBBA-BBBAAB-BBBBBBBBABA-AA-BAAB--A--BB

*003426_00059005 B-ABABABAABBBBBBBBBABBAAAABAABBABB-BBABBBB-BABBBABABBAABAAAB-ABAABBBAAAABAB-ABBAAAAABBBBB

*003448_00058553 BABBBBAAABBAAAABBA-ABABAAABAAABAAAABBBBAAABAA-ABABBAAABBAAAABAAAAAAAABBAAABBBBBAABBB-ABAA

*003471_00054113 BAABABABBBA-BABBBB-BAAAABA-B--AA-B-A-BBBBA-B-AABBAABBBABBAABAAABAABBABAABBAB-ABA----ABBBB

*003480_00062476 AAA-AAAB-A-AAABBAAAA-AB-BAB-AB---B-AABAABBBAB-BAAAA-ABBAAAAAAABB-AAB-B-BB-B-BBBBAAA-B--BB

*003480_00108863 A-A--A-BAABAA-BBAAAA-ABBBABAABBBBB--ABAABB-ABBBA-AABA--AAA-AAABBBAAB-BBBBABABBBBAAAB--BBB

*003481_00064358 AAA-AAABA-BBB-ABABBABBABABBBABABBABBBBBBABB-B--AA--ABAABABBAABBAB--BBA-ABBAAAAAABAAABAAAA

*003481_00065206 AAA-AAABA-BBB-ABABBABBABABBBABABBABBBBBBABB-BB-AA--ABAABABBAABBAB--BBA-ABBAAAAAABAAABAAAA

*003483_00018385 BBAAABBBAABA-BBAA-AB-BBBBBBABBAABBBAAABBBBBABABBBABAAABABAABAAB-B-BBBAAAAABBABBAAAABB-BAA

*003483_00027342 BBAAABBBAABA-BBAA--BBBBBBBBABBAABBBAAABBBBBAB-BBBABAAABABAABAAB-B-BBBAAAAABBABBAAAABBABAA

*003489_00022220 BABBBBAABBABABABBAABABBBAAAABABB-AAAABBA--A-AABABABA-ABBBABBBAABBBBAAB-BBABABBABBBBAAAAAA

*003498_00072650 BA-A-B-BBAABBBAA-BBAAABAAABAAAA-BA-AAABBBBABB--AAAABAB-BAAAABBAAA-ABAAAAAAABB-AA-BBAA-AAA

*003508_00005520 B-BABBAAABAB-ABBBBABAAABAA-ABABBBBBAABBAAAABA-AAABA-ABABBAAAAAABABAAAABAABBAABAABAAB-ABAA

*003508_00017169 BBB-BBAAABABBABBBBABAAABA-AABABBBB--ABB-AA-BA-AAABA-ABABBAAAAAABAB--AABAABBAABAAB-A---BAA

*003529_00004481 BBBABBBBABABBBAB-B-A-A---B-AABB-BAB-A--B-B-BBAA-BAAABBABBABBBBBAABAA-B-ABABA-B-AAAAB--BAA

*003544_00075785 AABBBAABAABBBABAABBBBBABBAAAB-BBABABBBBBBBBBBA-A-AAABAABBABAAABABBBBAA-BBABABAABBAAAAABAA

*003552_00057277 ABBABABBAAAAAAAABAABBBA-BA-ABBBBBBBBABBBBBBAAAABBA-BBAABAABAAABABABAAAB-AABAAABBAAAAAB-BB

*003571_00044178 ABBAB-BAABB-BABBBBBAABBBAAAABBBBAAABBBBBAAAAA-ABAAABAAAABBAB-BABBABAABBABBAABBBABAA-B-BBB

*003574_00110949 BAABABAAABBAAAA-BA-ABABAAABAAAB-AAABBBBA-A-AA-AA-BBAAAAB-AAABA-AAAAAAB-ABABBBABABBBBAABAA

*003582_00052277 AAAAAAABAABAAABBAAAABABBBABAABBBBBAAABAABBBABBBAAAABABBAAAAAAABB-AABABBBBABABBBBAA-BBABBB

*003584_00000495 B--B-BABABABBAABBB-BB-AA-BAABBA-ABABBBABBB-AABAA-A-BA-BBABBAABBBBBAABBBBBBBA-BB--BBB--ABB

*003587_00015020 AAAAAAAB-ABAAABBAAAABABBBABAABBBBBAAABAABBBABBBAAAABABBAAAAAAABB-AABABBBBABABBBBAAABBABBB

*003598_00065012 ABAAAAABAABAAABBAAAABBBBBABAABBBBBAAABAAABBBBBBAABABABBAAAAAAABB-AABABBBBABABBBBAAABBAABB

*003610_00023651 AAAAAA--BAAABBA-BBBBBBBBB-BBBABA-A-BBBABAB-A-BAABBBBBBABBBBAABABBBBBBBBABA-AAABAABBAAA-BB

*003622_00055447 B-ABABAB-ABBBBBBBBBBBBAAAABABBBABBBBBABBBBABABBAABAB-AABAAAB-ABAABBBAAAABAB-ABBAAAAABBBBB

*003623_00034281 B-BABBBBBAAB-BAAB-BBABBB--B-BAABBABAAAABABAABAAAA-AABBA-BAAAA-AAAAAABAABB-ABBB--BBBA-BAAA

*003628_00071281 BAABABAAABBAAAAB-A-ABABA-ABAAABAAAABBBBA-A-AAAAAABBAAAA-AAAABA-AAAAAABBABABBBABABBBBAABAA

*003640_00028100 BBBABBAAABABAABBBAABAAABBA-ABAABAB-BBBBBBAABABA-ABA-ABABBABAAAAAAAAAAABAAABABBAABAAAAAAAA

*003648_00025303 BABABBAABBBAAABBAAAABB-BBAABBABB-BABBBAABABBBABBAAABAABAAABAAABBAAAA-BABAABBBAABAAABBBB-A

*003662_00002276 BABABBBBBAABAABBBAABBBBBABBABABAA-AAABAABBAAAAABBAAAAABBBAA-BABABBAAAAB-BABBAAA-BBBAABBAA

*003666_00005819 BBBABBABBABABAABABAAAABABABBBAABBBBBBBABABBAB-AAABBBABAAABBABBA-BBAABBAAAAAAAAAAABBBBABBB

*003671_00045217 BAABAB-BBAABAABABABABAABAABBBBAAABA-AAABABABBABBABAAABAAB-AAAB-BBBAA-BABAAAAAABBBAAABAAAA

*003673_00022889 BABBBB-BBABBAABABABBBAAABBBBBBAAAA-AAAABBBAABBBAABAAABABBBBBABBBBBAABAABAAABAABBBA-ABAAAA

*003682_00103799 BBABABABAABBAB-ABA-BBABABBAABBB-BBBAAAABABBABBABBBBABAAAABBBAABBA-BBA--BAAB-BAABBAAAAAAAA

*003690_00020419 AAAAAA--BAAABBA-BBBBBBBBB-BBBABA-ABBBBABAB-A-BAABBBBBBABBBBAABABBBBBBBBABA-AAABAABBAAA-BB

*003696_00018388 BAAAABABBBBBBBBBABAAAAAAAABAAAB-BBBBBBABBBBAAAAABA-BBBAAAABBBBBB-AABBA-B-BB-B-ABBBBABBBBB

*003698_00045753 B--B-BABABABBAABBBBBBAAA-BAABBAAA-ABBBABBB-AABAAAA-BABBBABBAABBBBBAABBBBB-BABBBB-BBBAAABB

*003723_00009795 BBAAABABAABABBBAABAABBBABABABABABBBBBAABBBBBBBBAAAAABBBAABBBBA-BBA-ABBA-BABAABB-ABBAABAAA

*003726_00027552 BAA-ABABBBA-BABBB---AAAABAABAA--BBBAABBB-A-B--ABB-A-B-ABBAAB--ABAABBABAA-BAB-A-A-AAAAB-BB

*003728_00046364 BABABBBBBAABAABBBAABBBBBABBABABAA-A-ABAABBAAAAABBAAAAABBBAA-BABABBAAAABBBABBAAA-BBBAABBAA

*003729_00062348 B-ABABABAABBBBBBBBBABBAAAABAABBABBBBBABBBBABABBBABAB-AABAAAB-ABAABBBAAAABAB-ABBAAAAABBBBB

*003735_00022670 BB-AABABABABBBAB-BABBBABAAAABAAA----BABBAB-ABA-AAABAAAAABABABABABBABABABBAAAAABBAAABBB-AA

*003744_00043875 B-BBBBA-ABAABABB-BA--AB-BABBBABBBBBBABAA-ABBBABABBAAAABABBBAAAAAA-AAABAABBBAAAAABBBBBABBB

*003750_00066688 B-ABAB-A-BB-BB-B-BBA----B-AAA-BBB-BAABBB-B-BBAAA-B-ABBABBABBBBB-ABB-BBB-AABABBBBAAAB--BAA

*003779_00045944 B-BBBB-BAABBBABA-BABAAA--B-B-A-AAAAAAB-A-A-BBABB-ABAAABBBBB-BAAAAB-BBA-A-AA-A--AAB----AAA

*003804_00004934 BABBB-BBAA-BB-BAAB-BAAAABB-B-ABAA-A-ABAAAAABBABBBABAAABBBABBBA-AAB-BBBABAAABAABB-BB-ABA-A

*003804_00019300 BABBBB-BAAABB-BAABABAAAABBBBBABAAAAAABAAAAABBABBBABAAABBBABBBAAAAB-BBBABAAABAABBABB-ABAAA

*003810_00037383 BBB-BBAAABABAABBBAABA-ABBABABAABABBBBBBBBA-BABAAABA-ABABBABAAAAAAAAAAAB-AABABBAABA-AAAAAA

*003818_00044503 ABAAAAABAAB-AABBAAAABBBBB-B-ABBBBB-AABAAAB-BBBBA-BABABBA-AAAA-BB-AAB-BB-BAB-BBB-AAABB-ABB

*003844_00004750 BAAAABAAAABAAABAAAAABAAAAABBBAABBA-BBAA-AA-AA-ABBABBAABAAABABBBB-BBAAABBAAABBBAA-AAAAAABB

*003845_00002815 ABA-AAAAAABAAABAAAAABAAAAABBBAABBABAAAAAAABAABABBABAAAAABABABBBBABBAAABBAAABBAAA-AABBAAAA

*003872_00027435 BBABABBAABBBBBABBBBABAA-B-AAABBBBBBAABBB-BABBAAABB-A-BABBABBBBBAABBABBB-AABABBBBAAABB-BAA

*003879_00038335 B-AAABBAABBBBAABABAAAAA-BA-BAAB--BABB-BAABBB-B-AAAABAAABBAAABB-BAB-BBBABABBBBBBBBAABBBBBB

*003882_00076270 AAABAAABABBABAABBBAABB-AABABBABAABAAA-A-BA-ABBA-BAABAAABABAAAABBBBBA-AAABAAB-BAABBBAAABBB

*003883_00010689 B-BB-BABAB-BBAABB--BBAAAB-A-BBA-ABAAABAB-BBAABAAAA-BABBBBBBAABBBBBAABBBBBBBABBBBABBBAAABB

*003889_00012608 BAABABABBBABB-BBBB-BAAAABA-BABAA-B--ABBBBABBBAABBAABBBABBAAB-AABAABB-B-ABB-B-ABA--A-ABBBB

*003901_00041887 AAAAAAABAABAAABBAAAABABBBABAABBBBB-AABA-BB-AB-BAA-A-AB-AAAAAAABB-AABAB-BBABABBBBAAABB-BB-

*003910_00041871 B-BBBBABBA-BBAABABBA-BBAABABABBBAAABBBBBAB-BBAABBBBAAABBBBABAAABA-BAAAB-BBBABAA-BAABBBBAA

*003912_00023350 B-BABBBABABB-ABB--AB-BBBABBABA--AB--ABAA-B-AAA-BBA--AABB-AABB-BAB-AAABBBA-BB-A-ABBBA---AA

*003915_00005980 ABAAAABBBABBBBABBBABA-AABBBBBBBB-AAAABBBBABAABBAAAAABBABABABBAAAAAABAABABABBBBAABBBAABBAA

*003915_00006066 A-AAAABB-AB-B-ABBBABA-AABB-B-BB-AAAAABB-BA-AAB-AAAAAB-A-A-AB-AAAAAABA--ABAB---AA--BAABBAA

*003929_00009015 ABBABABBAAAAABAAAAA-BBA-BAAABBBBBBBAAABBBBBABAABBAABB-ABBAB-AABABABABAB-AABAABBBAAAAABBBB

*003931_00018651 AABBBABABBBBABABBAAABABAAABAABABBBBBBABBAABBAABBBAAABABBABBB-ABBBBBAB-AABAAA-BABBBBBAAAAA

*003946_00076881 B--B-BABABABBAABBBBBBAAA-BAABBAAABABBBABBB-AABAA-A-BABBBABBAABBBBBBABBBBBBBABBBB-BBBA-ABB

*003995_00052087 BABABB-BAABBBAB--B-BAAA-BBBBB-BAAA---BAABA-BB-BBB-B-A-BBBBBBBAA--BBBB-BAAAABAAB--BBBABAAA

*003996_00040345 AAAAAAABAABAAABBAAAABABBBABAABBBBBAAABAABBBABBBAAAABABBAAAAAAA-B-AABABBBBABABBBBAAABBABBB

*004002_00042429 A-BABABBAAAAABAAAAABBBB-BA-ABBBBBBBAAABBBBBAB-ABBAABBBBBBABAAABABABABAB-AABAABBBAAAAABBBB

*004008_00061238 AABABAABAAABBAAAABBBBBBBBABABABBABABBBABBBAB-AAAAAAABAABBABAAABABABB-B-B-ABBBB-BBAAAABAAA

*004011_00032290 BBA-ABBBABAB--ABBABAAAAABAB-ABABAB-A-BAB-BBBAAABA--BABBBB-BAABA--B-A-BBBB-BAABABAAAA--BBB

*004015_00059732 BBBABBA-BAAABB-BBBBBBABB-ABBAABBBB-ABABBBBAA-ABABBAA-ABBBBBABABBBAABABABAABBAABBBBBBBBAAA

*004028_00044367 BAAAABAAABBAAABBBA-ABABAAABAAABAAAABBBBAAA-AAAAAABBAAAABAAAA-AAAAABAABBABABBBABABBBBAABAA

*004047_00017736 B-AAABA--ABAAABBAAA-BABBBAABBBB-BBAAABB-BBBBBBBAAAABABBAAA----BBB-ABAB-BB-B-BBBB-AABB-BBB

*004052_00004955 BA-B-BABBBA-BABBBBBBAA--B-BB-BA-BB-AABBBBA-BB-ABB--BBB-BB-AB-AABAABBAB-ABBAB-ABAA-A-ABBBB

*004052_00018414 BAABABABBBA-BABBBB-BAAAABA-BA-AA-B-AABBB-ABB-AABBAABBBABBAABAAABAABBABAABBAB-ABAAAAAAB-BB

*004077_00037903 B-ABABABAABBB-BB-BBABBAAAABAABBABBBB-ABBBB-BABBBABAB-AABAAAB-ABAABBBAAAABAB-ABBAA-AABBBBB

*004081_00011796 BABBBBA-AAABAAAABA-BA-BB--BAAAA--ABBBABAABAAAAAAABAAB-AABAAAABAAAABABAABBAABBB-BABBA-BAAA

*004090_00008954 B-BBBBAABA-BB-BA-BAAA-BB-ABBBAABAA-ABABAAB-AAA--ABAABAAABBAA-BAAABBAB-BBBBA-B--BABBA--AAA

*004099_00029701 -BBBBAABBAA-BBABBB-AB--BA-BBBBBA-AAB-AABB--BB-ABBBB-AAABABABAABBA-BBA-BBBBAAB-A-AB-BBABAA

*004115_00013549 B--B-BABABABBAABBBBBBAAA-BAABBAAABABBBABBBBAABAA-A-BA-BBABBAABBBBBAABBBBBBBABBBB-BBBAAABB

*004130_00030825 BBBABBAAABABAABBBAABAAABBABABAABAB-BBBBBBAABABA-ABA-ABABBABAAAAAAAAAAABAAABABBAABAAAAAAAA

*004133_00105222 -AB-BAABAAA-BAAAABBBBBBBBABABABBABABBBABBBABBAAAAAAAB-A-BABAA-BABA-B-BBBB-BBB--B-AA-A-AAA

*004134_00004732 B--ABBBBAABABBBBABBABBBABABABBBAAAABBAAABBABBABABA-BBABBABBBAABABABABBB-BAB-ABB-ABBAABABB

*004145_00010448 BABABBBBAABAABA-AAABABAAABABBBABABAAABABAABAABAAAB-BBABABBBABAABB-AAAABABBAB-AAAABBAAAABB

*004147_00060555 B-ABABABBBA-BABBBB-BAAAABA-BA-AA-B-AABBBBA-B-AABBAABBBABB-ABAAABAABBABAABBABAABAAAA-AB-BB

*004152_00042735 B-----AB-BA-BAB-BBBB--AABA-BABA---AAA-BBBABBB---BAABBBABBAABA-ABAABB-BAABBAB-A-AAAA--BABB

*004196_00020628 BABABBBBAABAABA-AAABABAAABABBBABABAAABABAABAABAAAB-BBABABBBABAABB-AAAABABBAB-AAAABBAAAABB

*004196_00025515 BABABBB-AABAABA-AAABABAAABAB-BABABAAAB-BAABAABAAAB-BBABABBBABAABB-AAAABABBAB-AAAABBAAAABB

*004204_00040982 BAB-BBBBBAABA-BBBA-BBBBBA-BABAB-ABAAABAABB-A-AAB-AAAA----AA--ABABB--AABB-ABBAAA-BBB--B---

*004205_00048276 AABBBAABAA-BBBAAABBBBBBBAABAAAB-ABA-BBABBB-AAAA-AAAABBBB--BAAABABABBBBA--ABBABB-BAAABB-AA

*004228_00034372 B-AB-BBBABAAAA-A-ABBAA-AABBA-BA-AA--BBAAABBAA---BAAAA-BABABAAABAABABB-AA-BA-BABAAAA-B-AAA

*004230_00053895 BBBBBBABABABAAABBAB-BAAABABA-BABAB-AABA-AB-BAAAAAA-BABBBB-BAABBBABAAABBBBA-AABABA-A-B-BBB

*004264_00019252 B-A--BABBBA-BABBB--BA-AA-ABBABAAAB-AA-BBB--B---B-AAB-BAB-AABA-ABAABBABAABBABBA-A-AA----BB

*004275_00019212 BBBABBBBABA-BBABABBABAA-ABBAABBABABAABBBABABB-ABBA-A-BABAABBB-BAABAABB-ABA-AB-BAAAABB--AA

*004296_00032076 BBBABBAAABABBABBBBABAAABAA-ABABBBBBAABBAAA-BA-AAABABA-ABBAAAAAAAABAAAABAABBAABAABAABBABAA

*004297_00033565 ABAAAAABAABAAABBAAAABBBBBABAABBBBBAAABAAABBBBBBAABABA-BAAAAA-ABB-AABAB-BBABABB-BAAABBAABB

*004308_00039161 -AAAAA--BAAABBAA-BBBB-BBB-BBBABAAABB-BA-ABBA---ABBBB--ABBBBAABABBBBBBBBABA--AABAABB----B-

*004311_00019649 B-A-ABAAA-BAAAABBA--BABAAABAAA-AA-ABBBBAAABAAAAAABBAA-ABAAAABAAAAAAAABBABABBBABABBBBA-BAA

*004315_00000177 A--AAABBA-BAB-AAB--B-A-ABB--AA--A-B--AAABB-A--BBAB-ABAAABAAABB----BAAAAABABAAAAAABBA-BBAA

*004317_00036296 BA---B-AAAABBA-AB--BA---ABBAAAABBA-BBAB-AB--BAAAA-AA-BA--AAAA-AAAABA-AA-BAABBB-BABBA-BAAA

*004318_00029266 BB-A-BBBAA-ABBBBABBABBBABABABBBAAAABBAAABBABBABABAA-BABBABBBAABABABABBBBBABBAAB-ABBAABABB

*004350_00038321 ABBABABBAAAAAAAABAABBBA-BA-ABB-BBBBBABBBBBBAAAABBA-BBAABAABAAABABABAAAB-AABAAABBBAAAABBBB

*004351_00019477 BBA-ABABAABBAB-ABA-BBABABBAABBBAB-BAAAABABBABBABBBBABAAAABBBAABBA-BBA--BAABBB-ABBAAAAA-AA

*004367_00025493 A-BABAABAAABBBAAAB-BBBBBAABAAAB-ABA-BBABBBA-AAAAAAAABBABBABAAABABB-B-B-B-ABBABBBBAA-BBAAA

*004369_00035386 B-BABBBBBAABAABBBAABBBBBABBABABAABAAABAABB-AAAABBAAAAABBBAA---BABBAAAABBBABBAAA--BBAABBAA

*004371_00000404 AAA-AA--BAAABBA-BBB-BBBBB-BBBABA--BBBBABAB-A-BAA-BBBB-A-BBBAABABB-BBBBBABA--AA-A-B-A---BB

*004372_00019306 BABABBBBBAABAABBBAABBBBBABBABABAA-AAABAABBAAAAABBAAAAABBBAA-BABABBAAAABBBABBAAA-BBBAABBAA

*004381_00025165 B-B-BBBBABB-BAABABBA-ABAAAABBB-BBB-AABBBABA-BABBBBBBA-ABABABABABABB-AB-ABABBBAAABBBBB-BBB

*004408_00062091 BABBBBAABAABB-BABBAAA-BBBABBBAABAAAABABAAB-AAA-AABAA-AAAB-A-BBAAABBABABBBAABBBBBABBA-BAAA

*004411_00025946 BAABABAAABBAAAABBA-ABABAAABAAA-AAA-BBBBAAABAAAAAAB-AAAABAAAABAAAAAAAABBABABBBABABBBBAABAA

*004417_00016029 B-AA-BAB-AAABAABBBAA-AB-AA-BB-B-A-A--BABABB--ABAAAAA-B--BABA-AABA-ABAABABBBBA--B-BBABAB-A

*004422_00036490 A-BABAA-ABBBB-ABABBABAABAB-AABBABA--ABA-B--BBAABBAB-ABABA-BBBBBA-BA-ABBABA-A-BBA-AABBA-AA

*004426_00017118 AAABAAABA-ABB-BAABBABAAA-BBBABBBAABBB-BAB---BBBB-BB-BBABABBAABBABBABAB-BABB--AB--A-AAA--A

*004426_00021201 AAABAAABA-ABB-BAABBABAAABBBBABBBAABBBA-AB-B-BBBB-BBABBABABBAABBABBAB-B--ABBBBABBAAAAAAAAA

*004430_00016356 B-ABABABBBA--ABBBB-BAAAABA-BAA-A--AAABBBBABBB-ABBAABB-AB-AABAAA-AA--ABAA-BABBA-AAAAA-BAB-

*004430_00023866 B--BABABBBABBABBB-ABAAA-BA-B--AA-B--ABB-BA-B-A-BBAABB-ABBAA----BAABBA-AABB-B-A-AAA-AA-BBB

*004466_00002993 -AABABAAAAABBAABBBA--AAABAABBA-BBAB-ABBAAABABAABBBABAABAAB-AAABABABABB-AAABBAABABAAABABBB

*004470_00008973 BABABB-BAABBBABA-BABAAAABBBBBABAAAAAABAABAABB-BBBABAAABBBBB-BAAAAB-BB-BAAAABAA-AABBBABAAA

*004474_00038086 B-BABBBBBAABAABBBAABB-BBABBAB-BAA-A-ABAABB-AAAABBAAAA-B-BAA-BA-AB-AAAABB-ABBAAA-BBB---B-A

*004474_00038172 BABABBBBBAABAABB-AABBBBBABBABABAA-AAABAABBAAAAABBAAAAAB-BAA-BABABBAAAABBBABBAAA-BBBAABBAA

*004491_00135618 AAAAAAABAABAAABBAAAABABBBABAABBBBBAAABAABBBABBBAAAABABBAAAAAAABBBAABABBBBABABBBBAAABBABBB

*004507_00005361 BAABABABBBA-BABBBB-BA-AABA-BABA---AAABBBB--B--A-BAABB-ABBAABAAAB-ABBABA-BBAB-ABAAAAA-BBBB

*004515_00065714 AAABAAABABBAAAABBAAABB--ABABBABAA--AA-AABAAABBABBAABAAABABAAAABBBBBB-AAABAABBBAABBBAAABBB

*004518_00013687 BAABABABBBA-BABBBB-BAAAABA-BA-AA-BBAABBBBABBAAABBAABBBABBAABAAABAABBABAABBAB-A-AAAAAAB-BB

*004526_00049717 BABABBBBBAABAA-BBAABBBBBABBA-ABAAAAAABAABB-AAAABBAAAAABBBAA-BABABBAAAABBBABBAAAABBBAABBAA

*004545_00009710 B-ABABABABBBBABBBBBABBAAAAB-AB-ABBBBBABBBB-BABBBA-ABBABBAAAB-ABBA-BBAAAABAAAA-BAAAAABBBBB

*004557_00053802 --ABAB-AABBB-B-BBB--BAA--B-AA-BBBBBAAB---BABBA-A-B-A-BABBAB-BBBAABBABBBAAABABB-BAAA-BABAA

*004558_00027644 BABBBBBABAAB-BAAA---A-BB--BABAABBABBBABAABA-BAAAA-AABBAABAAAA-AAAABABAABB-ABBBB-ABBA-BAAA

*004560_00044690 BBAAABBBBBABA-AABABBBA-BA-ABBAAABBA--A-BBABAAABBAAAABAABABB-ABAAABBBBBAAAABBAABABAA-AAAAA

*004563_00006896 A-AAAAAAABBA-ABAB-BBAAABBBBAABBBAA-BBABAAB-BBBBA-BABA--BAAAABAAAABABABAAABA-A-AAB-BBBBBBB

*004567_00017186 B-A-A-AB-ABAAABB-AA-BA----ABBBB-BBAA-BBABB-BB-BA-AAB-BBAAAA-AABB-AAAA-ABBA-ABABBAAAB----A

*004577_00004147 AAAAAA--BAAABBA-BBBBBBBBBABBBAB--A-BBBABABBA-BAABBBBBBABBBBAABABBBBBBBBABA-AAABAABBAAA-BB

*004613_00021772 BBAAABAABBAABBAAABABBBBBBABBBAAA-AABBAB-BA--BBA-BBAABBABAAB-BBBAAAABB-AABAABB-BABBBAABAAA

*004613_00056000 B-A-AB-ABBAABBAAAB--BBBBB-BBBA-ABAAB-AB-BA--B---B-A-B-ABAAB-BBBA-AABBBAABAABB-BA---AA-AAA

*004614_00061373 BBAAABABBBBAAA-AB-ABBA-BA-BAAA--BBBA-ABAA--AB-BBBABBAA-ABAABABBBBABBA-AAABBAAB-AAAABBABBB

*004615_00004463 B-B-BBBAABBABAABBBBAABAABABABBB-AAABBABBAAAAABABABABABAABBAB-BABBAB-AABABBABBBAABBBAB-BBB

*004620_00044403 BBBABBAAAAABBBBBABBBB-AAA-BBBABAAAAAAAAABAAABBB-BB-AAABBAAABBABBBBABABBABB-BB-BBABBABAAAA

*004656_00030687 BB-B-BBBABBAAA-ABABBABAAABAAABABABABBBAAA-BAABB-BAAA-ABABABAAA-AABABBAAAABA-AABAAAAAB-AAA

*004661_00036577 B-BBBB-BBABBAABABABBBAAABB-BBBAA----A-ABB--ABBBAABAAABAB-BB-A--BBBA--AAB-AABAA-BB--A-AAAA

*004678_00066957 A-BABAABA-A-AAAABAAB-BA-BA-ABBBBBBBBABBB-B-AA-ABBABBBAA-AABAAABABABA-AB-AAB-AA-BBAAAABABB

*004698_00029830 AABBBABABBBBABABBAAABABAAABAABABBBBBBABBAABBAABBBAAABABBABBB-ABBBBBAB-AABAAA-BABBBBBAAAAA

*004711_00021630 BBBBBBAAAABBBB-BABB-BAAAAAA-BBBABABBBABAA-AAAB-BBBAAAAAB-A-ABBBABAABBABBAABABBAABBBBAAAAA

*004712_00032147 BBBABB-BBAABABBABAABBAABB-ABBAAAAAA-AABBBBAABBBAAAAABB-BBABAABBAAAABBBAA-AABAA---BBBAAAAA

*004753_00024863 B--B-BABABABBAABBBBBBAAA-BAABBAAABABBBABBBBAABAAAA-BA-BBABBAABBBBBAABBBBB-BABBBB-BBBAAABB

*004757_00062852 B-ABABABAABBBBBBBBBABBAAAABAABBABBBBBABBBBABABBBABAB-AABAAAB-ABAABBBAAAABAB-ABBAAAAABBBBB

*004796_00065827 BBABABBAABBBBBABBBBABAA-B-AAABBBBBB-AB-B-BABBAAABB-A-BABBABBBBBAABBABBB-AABABBBBAAABB-BAA

*004866_00028150 BB-AABAABBAABBAAABABB-BB-A-BBAAA-AABBABABA-BBBABBBAAA-ABAAB-BBBAAAABB-AABAABB-BA-BBAABAAA

*004891_00008015 BABBBB-AAAABB-BAABABBAAABBBBBABAAAAAABAAAAABBABBBABAAABB-A-BBABAAB-BBBBBAAABAABBABB-ABAAA

*004891_00008101 BABBBB-AAAABB-BAABABBAAABBBB-ABAAAAAABAAA-ABB-BBBABAA-BB-A-BBABAAB-BBBBBAAABAABBABB-ABAAA

*004917_00038097 BA-ABBBBBAABAABBBAABBBBBABBA--BAA-AAABA-BB-AAAA-BAA-AABBBAA-BABABBAAAAB-BABBAAA-BBBAABBAA

*004924_00032776 ABBABAB--BB-BBABABBABAA-ABBA-BBABABAABAB--ABBAABBABAABABA-BBBBBABBAAABBABA-A-BBAAA-BB-BAA

*004926_00004144 -BABA-AAABAA-ABAB-BBBAABBBBBABABAAABBBBAABBBA-BBBBBBABBAAABBBBBAABBBBB-ABBA-BBAABBBBBABBB

*004929_00021335 ABBABAAABAAAABBBBA-AB-AAAABBBAB-AA-AAAABBBABBBA-BBB-AABBAAAB-ABBA-BBAB-ABBABB--A-BB--A-AA

*004939_00012510 BBAAABAABABBBBBAABAB-AAAAAAABAA-BBBBBBBABBBAAAABBABAABAAAABABAABB-ABAAABAAAABAABBBBABABAA

*004958_00037777 BABA-B-BAABBBABA-BABAAAABBBBBABAAAAAABAA-A-BBABBBABAAABBBBBBBAAAAB-BB-BAAAABAAAAABBBABAAA

*004976_00028071 A-BABAABAAABBBAABB-BBBBB--BAAABBABABBBABBBAAAAAABAAA-B-BBABAAABABBBBABAB-ABBABBBBAABBBAAA

*004990_00026200 A--B-AAABABABBAAA--BBABB-BAAAB-BAAAAAABBB-ABBAAAABAABBBA-ABBA-A-BAABBAABBBAAB-ABB-BAAABAA

*004991_00027310 BABABBBAAABAABA-BAABABAAABABBBABABAAABABBABAABABAB-BBABABBBABAABB-AAAA-ABBAB-AAAABBAAAABB

*005040_00003657 AAAAAABBAB--ABABAAB-BAAB-BBAABBA-ABAABABA--BAABBBABABA-BAAABBBB-B-AA-BBABA-ABBBAAAA--ABAA

*005046_00036143 BAAB-BABBBA-BABBBB---AA-BA-BA-AA-B-A-BBBBA-B-AABBAABBBABBAAB-AABAABBABA-BBA--A-AAAAA---BB

*005072_00000688 ABABAABBBBBABABBABBAABBBBAABBBBBABABBBBABA-B-BBAABBABBABBBAABAABAABABBBAB-BABA-ABAA-BABAA

*005075_00005369 AAAAAAB-BA-ABBA-BBBBBBBBB-BB--B-A--BBBAB-BBAA-AABBBBB---BBBAABABBBBB-BB-BA-AAABA-BBAAA-BB

*005084_00004064 B-ABABBAABBBBBABBBBAAABAA-BBABBBBBBAABBB-BABBAAABBAA-BABAABBBBBAABBABBB-A-BAABBB-AABBBBAA

*005100_00047304 B--B-BABABABBAAB-BBBBAAA-B-ABB-AABABB-ABBB-AA-AAAAABA-BBABBAAB-BBBA-BBBBBBBABBBBBBBBAA-BB

*005125_00016143 ABBABAAAB-BABBBBABAAABAABAABBBABBABABBB-AA-BBBBBAAAABABBBABA-BAABABABBAAABABBABABAAAABBAA

*005135_00007639 B-BABBABAABAABABBAABABAAABABB-BABBBAAAAAAAABA-AABBBBBA-BBBAABAABBBAAAABABBAB-AA-BBBBB-A-A

*005137_00003781 BAAAABAAABBAAABBBA-ABABAAABBAABAAAABBB-AAABAAAAAABBAAAABAAAABAAAAABAABBABABBBA-ABBBBAABAA

*005138_00011969 B-B-BBBBBAA-A-B-BAABB-BBABBABABAABAA-BAABBAAAAA-B-AAAAB-BAA-BAB---AA-ABBBABBA----BB--BBAA

*005192_00018607 BAABAB-B-ABBAABABABBBAAABBBBBBAAAB-AAAABBBAABABBABAAABABBBBBABBBBBAA-AABAAABAABBBAAABAAAA

*005202_00004330 BAB---AAAAABAAAAB--BABBBAB-AAAAB-AB-BABAABABAA-AA-AA--AABAA--BAAAA-A-AA-BAABBBB-A-BA-BAAA

*005206_00021867 B--B-BABABABBAABBBBBBAAA-BAABBAAABABBBABBB-AA-AA-AAB-BBBABBAABBBBBBA-BBBBBBABB-B-B-BA-ABB

*005230_00020059 BBABABABAABBBABBBBBABBAAAABAABAABBBBBABBBB-BABBB--AB--ABAA-BB-BAA-BBAAAABAAAABBA-AAAB-BBB

*005248_00019488 B-A-ABABAA-BAB-ABABBBABABBAA-BBAB-B-AAA-AB-AB-A-BBBABAAAA---AABBAABBAA-BAABBBAA-BAA--AAAA

*005258_00005756 BB-BBBA-ABAABABB-BAB-AB-BABBBABB-B-BABAAA----ABABBAAAABABBBAAAAA-BAAABAABBBAAA-ABBBBBABBB

*005258_00005842 -BB-BBA-ABAABABB-B---AB-BABBBABB--BBABAAAA-BBA-ABBAAAABABBBAAAAAA-A--BAABBB-AA-ABBB-B--BB

*005267_00008341 AAAAAAABAABAAABBAAAABABBBABAABBBBBAAABAAB-BAB-BAAAABABBAAAAAAABB-AABABBBBABABBBBAAABB-BBB

*005281_00017546 AA-B-A-BABABBAB-BB--AABBAABABBBABBBBBABAABBABBA-AA-ABABBA-ABBAAAAB-AA-BABABABAA-AAABBBBAA

*005315_00011123 BBABA-BAABBBBBAB--BABAA-B-AA-BBBB-BA-BBBBB-BB-AABBAA-BABBABBBBBAABBABBBBAAB-BBB-AAABB-BAA

*005324_00007514 BABB--AABBBBABBBBA-B-B---A-ABAB-B-B-ABAAAA-AA-B-BABA-A-A-ABBBAA-B-BA-BABBABABBAB-BB-AA-AA

*005334_00003366 A-BBBAAAABBAABBABAA-BBABABABABAAABABAABBAABBA-BBBAAABAABABBBABBBABBAAABBBBAAABABBAAABBAAA

*005335_00003124 BAABABBABBBB-AAAA-BAABAABAAABABAAAABBABA-AABB-BB-AA-ABABBAAA-B-BBAAB---BABB-BBBB-AABBBBAA

*005351_00029501 B-ABABABAABBABAABAA-BABB--B-BBB-BBBAAAABAB-BB-ABBBBABAAAABABAABBA-B-AA-BAABBBAABBBBAABAAA

*005365_00009124 -A-BBBBBABB-BAABABBAABBAAAAB-B-BABAAAB-BAB-A--B-BBBBAABBABABABABBBBAAABABBBBBAAA-B-B--BBB

*005365_00037240 BABBBBBBABBBBAABABB-ABBAAAABABBBAB-AABBB-BAABABBBBBB--BBABABABAB-BBAA-BABBBBBAAABBBBB-BBB

*005386_00002991 AB-BAABBAB-B-ABAAABAAB-AAB-AAB--AB-AABA-BB-AABB-AAABAABAAA-AAA-BAB-ABAABBBBAA-B-A--AAAB--

*005410_00021340 --BA--B-AAAABBAB-BBAA-BA-BBBABAAAA--AAB-AA---BBAAABB---BBAAABABABAB---AAAAA-A--ABBBABA-BB

*005460_00000183 BAAAAB-AAAB-BAABABBABAA-BA-AAAB-BBBAAB-BAB-BBB-AABABAAABBABBBBBBBBAAABAABBBB-BBABAABBABBB

*005462_00039709 AB--BA-AAB-A--BABAA--BABABABABA-AB-BAABBA-BBABB-B-A-BAA-ABBBABB-ABB-A--B-BA--BABBAAA-BAAA

*005470_00047748 BA-A-B-BBAABBBAA-BBAAABAAA-AAAABBABAAABBBB-BB--AAAABAB-BAAAABBAAA-ABAAAAAAABB-AABBBAAAAAA

*005502_00029386 BAB-BB-AAAAB-AAAB-BBA-BB--BAAAABB--BBAB-A---AAAAABAA--AAB-AA-BAAAABABAABBAABBBBBABBA-BAAA

*005522_00055720 BBABA-BA-BBBBBABBBBABAAB--AAA--BBBB-AB-BABA-BAA-BBAA-B-BBABBBB-AABBABBB---B----B-A-B--BAA

*005533_00002038 BBABABBAABBBBBABBBBABAA-BAAAABBBBBBAABBB-BAB-AAABB-A-BABBABBBBBAABBABBB-AABABBBBA-ABBABAA

*005584_00036262 BB-B-BBBAB-AA-BABABAABAAABAAABABABABBBAABBAAABB-BAAA-ABABABAAA-AAB-ABAAAABABA-BAA--AA--AA

*005605_00009179 A-AAAAAAAABA-AAB-A-ABBABBBBAAABB-BA-A-AAA--B-B-AABABABBAAAAABA-B-AA-ABBBBBBAB-BB-AABBAABB

*005616_00018528 BBAAABAABBAABBAAABABBBBBBABBBAAA-AABBAB-BAA-BBAABBAABBABAAB-BBBAAAABB-AABAABB-BABBBAABAAA

*005622_00054732 BAABABABBBA-BABBBB-BAAAABA-BA-AA-B-AABBB-ABB-AABBAABBBABBAABAAABAABBABAABBAB-ABAAAAAAB-BB

*005623_00010867 AAAAAAAAAABA-ABAA-BBBAABBBBAAA-BAAABB-BAAB-BB-BABBABB-AAAAAABAABAAABABAAABAAB-AAABBBB-BBB

*005628_00027045 A-AAAABAAABA-BAA-BA-AA-ABB-AAA-A--B-AAAAAB---BBBAABABBAB-AAAABBBBAB-BABABABAAAAAAB-A-BBAA

*005638_00012096 AABBBABABBBBABABBAAABABAAABAABABBBBBBBBBBBBBAABBBABABABBABBBBABBBBBBBAAABAABABABBBBBAAAAA

*005655_00043081 BBBABBAAABABBABBBBABAAABAA-ABABBBBBAABBAAAABA-AAABA-ABABBAAAAAABABAAAABAABBAABAABAABBABAA

*005668_00012288 BABABBBAABABAABB-AAB-AAAB-AABAAABBBB-ABBBBABABAAABABAA-BBABAAA-AAABBBBBABBBA--BABAAB-AABB

*005668_00017677 B-B-BBBAABABAABB-AAB-AAAB-AABAAABB-B-ABBBBABABAAABABAA-BBABAAA---ABBBBB-BBBA--BA--ABBAABB

*005699_00000221 A-BABAAB--AB-AAAABB---BBB-BABAB-ABABBBABBB-B--AAAAAABAA-BABAAABABABBAB--BAB-BB--BAAAAB-AA

*005720_00027504 BBBABBBBAABAABABBAABABAAABABBBBBBBBAAA-AAAAAABAABBBBBABABBBA-AABBBAAAABABBAB-AAABBBBABABB

*005777_00022250 AABABAABAAABBB-AABBBBBBBBABAAABBABABBBABBB-B-AAAAAAABAABBABAAABABABB-B-B-ABBBB-BBAAABBAAA

*005789_00000848 BBAAAB-BAAAABAABBBAAAABAAABBBBBBAAAA-BA-ABBBA-BAAAAABBAABABA-AA-AAABAABABBBBA-ABBB-ABABAA

*005828_00001396 BBABABBBABABBBABBBBB-BAABBB-AAA-BBBBBAAABBBBABBBBABAAABBBBBBBAABAAABAAABB-A-ABBBAAAABBBAA

*005849_00004098 B-BABBAAABBAAABAAABABBABAABBAABAAAABBABBAB-AAAAAABBB-AA-AA-ABBBBAAABABBABABBBABABBBABABBB

*005874_00017383 BBBABBAB-ABAABABBAABABAA-BABBBBABBBA-A-A-A-BABA-BBBBBA-BBBAABA-BBBAAAABABBABBAAABBBBA-ABB

*005900_00024588 B-ABABAB-ABBBBBBABBBBBAAAABABB-ABB-BBAA-A-ABABBAABAB-AABABAB-ABA-BAAABAABAB-ABBAA-AA-BBBB

*005909_00022212 BBABABBAABBBBBABBBBABAA-B-AAABBBBBBAABBB-B-BBAAABB-A-BABBABBBB-AABBABBB-AABABBB-AAAB--BAA

*005914_00030485 B-A-ABBBABB-BABA-BB--ABAAAABBBBB-BBAABBA-B-BB--BB-BBA-ABABBBBAABABAAABBABABBBAAABBBBBBABB

*005921_00012847 BB-A-BBBAABABBBBABBABBBABABABBBAAA-BBAAABBABB-BA-AABBABBABBBAABABABABBBBBABBA-B-ABBAAB-BB

*005960_00029760 BAB-BBAAABBABAABA-ABBB-BB-ABBA-BBBBBBABAAA-AAABBAAABAABBA-BABAAAABAB-BABABBBBAABAAABB-ABB

*005969_00014010 BBBABBAAABABAABBBAABAAABBA-ABAABABBAA-BBBAABA-A-ABA-ABABBABAAAABAAAAAABAAABABBAABAAAAAAAA

*005988_00009965 BA-BBBABBAABBBABBB-AABABABABABAAAAABBABBABBBBA-BBB-A-ABBBBAAAAABA-BA--B-BBAABA-BBBBBBBBAA

*006003_00018725 AAAAAABBAABAB-AA-BA-AA-ABB-AAABA-ABAAAAA-B----BB-BBAB-AABAAABBBBB-BAAAAABA-AAAAA-BBAABBAA

*006014_00022081 B-BABBAAABABA-BBBAABA-ABBA--BABBBB--BB-AAA-BA-BAABABABABBAAAA--AABAAAA--ABB--B-AB-AA-ABA-

*006036_00005335 B-ABABABAABBBABBBBBABBAAAABAAB-ABBBBBABBBB-BA-BBA-ABAAABAAAB-ABAA-BBAAAABAAAAABAAAAA-BBBB

*006136_00007858 BA-A-BBAABABABBB-AABAAABBB-ABAAABBB--ABB-AABABAAABABBABBBA-AAA-BAAABBBBABABA-BBABAAB-AABB

*006176_00002078 A--B-AB--ABB-BBBBAA-BABBAABABBBAA-A--AAABB-AB-BBABAABAABAAABBABAAB-AA-BABABABA-ABB-BBBBAA

*006178_00002692 -BBAB-BBAAAABBABABBAAABAABBBABAAAAAAAAB-AAAAB-BAAABBBA-BBABA-ABABAB-BAAAAAAAAAAA-B-A-ABBB

*006211_00024286 B-ABA-ABABBB-ABB-BBA-BAA-ABAAB-AB-B--ABB-B--A-B-AAA--AB-AAAB-A-BA-BBAAAABAAAAABAAAAA---BB

*006266_00025924 AAABAAABAAABB-BAABBA-ABABBBBABBBAABBBA--BAB-BBBA-BBABAABABBAABBABBAB-A--ABBBAAABBAAAAAAAA

*006266_00026167 AAABA-ABAA-BB-BAABBA-AB-BBBBABB-AABBBA-BBA--BBBA-BBAB-A-ABBAABBAB-AB-A--AB-BAA-BBA-AAAAAA

*006299_00031395 BAABABAAABBAAAABBA-ABABAAABAAABAAAABBBBAAA-AABABABBAAABBAAAABAAAAAAAABBAAABBBBBAABBBAABAA

*006328_00001856 B-B-BBBAABABBBA-BB-ABAAB---A-BBA-BBAABB--BAB-AA--B-AA-A----BBBBAABAABBBAAA-ABBBA-AAB-ABAA

*006342_00005274 A-BABAAAB-BABBBBABAAABAABAAB-BA-BABABBBAAAB-BBB-AAABAABBBABA-BAABBBABBAAABA-BABABAAAABBBB

*006346_00015258 BABB-B-BBABBAABABABB-AAA-BBBBBAAAA-AAAABBBAAB-BAABAAABA-BBBBABB--BAABAABAAABAABB-AAA-AAAA

*006348_00017799 A-AAAABBAA-ABBAA-BA-AA-ABBBAAA-A--BAAAAAABA---BB-ABAB-A-BAAABBBBBABABABABABAAAAAABBAABBAA

*006351_00001746 BABBB-A-BAAB-BA-BB-AA-ABA-ABABAAAAABB--BAB-BBAABBB-A-A-BBBAAAAABA-BAAABA-BAA-AB-BAA-BBBAA

*006398_00006953 BABABBAABBBAAABBAAAABB-BBAABBABB-B--BBAABABBB-BBAAABAABAAABAAABBAAAA-BABABBBBAABAA-BB-ABB

*006399_00014728 BBB-BBBBABABBAABBBBBBAB-ABBB-BABAAAA-BB-BAAABABBAABBBABAABBABABABA-BBAA-AAAABBBABBBAAABBB

*006410_00031002 BBBBBBAAAABBABBAAAAABBAABABB-A-A-BBAAAA-BAABABBBAAABABBAABBBBBBBAABABBABAA----BAB--BBBABB

*006410_00031088 BBBBBBAAAABBABBAAAAABBAABABBBA-A-B-AAAA-BAABABBBAAABABBAABBBBBBBAABABBAB-A-B---AB-BB-BABB

*006446_00001479 BA--BBAB-BABB-ABBBBB-AAAAB-A-B-AA-A-BBA--B--ABAAAABB--BBABBA-BBB-BAABB-BBBBABBB-AB--AA-BB

*006452_00010116 A-BB-ABBAB-BAABAAABAABAAABA-A-BBABBAABABBBAA-BBBAAABAABA-ABAAAABAB-BAAABBBB-B-B--B-A-AB--

*006459_00029356 A-AAAABB-AB--BABBBA---AA-BBBBBBB-AAA-BBBBABAAB-AAAAABBA-ABA-BAAAAAA-AA-A-ABBBBAA--BA-B-AA

*006480_00000838 BBA-ABABBABBBBBBABAAAAA-AABAAABBBB-BBBABBBBAAAAAB-ABBBAAA-BABBBB-AABBAB--BBAB-ABBBB-BBBBB

*006518_00007406 ABBBBAAAABBBABBAAAABBBAB-BABAABA-BABAABBAABBBB-BABAABABAABABABBBABB-AABBABBBAAABBAAAAAAAA

*006539_00003629 BBBABBAAABABABBBB-A-AAABBA-AB-ABAB-AA-BBBAABA-AAABA-BBABBABAAAABAAAAAABAAABABBAABAAA-AAAA

*006569_00008618 ABAAAAABAABAAABBAAAABBBBBABA-BBBBBAAABAAABBBBBBAABABABBAAAAAAABB-AABABBBBABABBBBAAABBAABB

*006571_00003147 B-AAABAAAA--AA-AAAAABAAAAABBBA-BB---B-AAAABAABABBABBA-B-AABABBBBBBBAAAB-AAA-BB-ABAAA--ABB

*006619_00006291 BBABABBAABBBBBABBBBABAA-B-AAABBBBBBAAB-B-BABBAAABB-A-BABBABBBBBAABBABBB-AABABBBBAAABB-BAA

*006619_00006399 B-AB-BBAA-BBBBABBBBABAA-BAAAABB-BBBAA-B-AB-BBAAABB-A-BABBABBBBBAABBABBB-AABABBBBAA-BBABAA

*006624_00008401 BBBABBA-ABBA-ABAB-AAAABB-AABAABAAAA-ABB-ABBA-A-BAABABBB-ABAABAAAABAABA-BA-BAAAABBBBBAABAA

*006674_00008443 B-AAABABBBBAA-BAAAABBABBABBABAAABBBAAABAABAABABBBABBAABABAABABB-BABBB-AAAABAABBAAAABBABBB

*006676_00005292 BAAAAB-AAAB-BAABABBABAA-BABAAAB-BBBAABABAB-BBB-AABA-AAABBABBBBBBABAAABAABBBB-BBABAABBABBB

*006710_00005589 BBABABBABABBABBABABBBBBABAB-AAAABABBBABAB-ABB-BABAAABBABBABABB-ABAABABBA--AABBBA-BBBBABAA

*006718_00032439 A-BBBAA-ABBBABBABAABBBAB-BABAA-A-BABAABBAA-BBBA-AAAABAAAABABABBBABBAAABBABBBA--BBAAAABAAA

*006718_00039351 ABBBBAAAABBBABBABAA-BBAB-BABAABA-BABAABBAABBB-ABAAAABAAAABABABBBABBAAABBABBBA-ABBAAAABAAA

*006736_00009947 BBABABABAABBBBBBBBBABBAAAABAABBABBB-BABBBBABA-BBABAB-AABAA-B-ABAABBB-AAABAB-ABBA-AAABBBBB

*006745_00007410 A-BB--BBABBAABA-BABABAAB-BB-ABBAAAA-ABA-AA-BA--B-AAABAA-AAAAABBBBBAB-BAABABA-ABAA-BB-AAAA

*006791_00001184 BBBBBBAAAABBABBAAAAABBAABABBBA-A-B-AAAA-BAABABBBAAABABBAABBBBBBBAABABBABAA----BAB--BBBABB

*006806_00010603 BAAAAB-AAAB-BAABABBABAA-BA-AAAB-BB-AABA-ABBBBBA-ABABAAABBABBBBBB-BAAABAABBBBBBBABAABBABBB

*006806_00010689 BAAA---A-ABBBAABABBABAAA-A-AAAB-BBBAABABAB-BBB--A--BAAABBABBBBBBBBAAABAAB--BBB----AB-ABBB

*006838_00013524 B-BBBBAABBA-ABABBAABABBBABABAABB-AA-ABBA--A-A---BABAB-BBBABB-AABBB-AABBBAABABB-B-BBA--AAA

*006939_00002717 B-ABAB-AAAABB-ABBBA-AAAABAABBABBBA--ABB--ABABAABBBABAA-AABBAAAB--BBAB-AAAABBAABA-AAA-ABBB

*006964_00003505 BBB-BBAAAA-BBBABAB--AAAAAA-BABABAAABAABAABAAA--BABBBBAABBB-ABAAABBABAABBAAAABB-ABBBAAAABB

*006988_00011054 B-BBBBAAABBAAABBBA-ABABBBABAAABAAAAAAB--AABAAB-B-BBAAABBAAAABAAAAAAAABBAA-BA-AABABBBAABAA

*006988_00011140 B-BBBBAAABBAAABBBA-ABABBBABAAABAAAAAABB-AABAA--B-BBAAABBAAAABAAAAAAAABBAA-BA-AABABBBAABAA

*006989_00006855 BAABAB-BBAABAABABABABAABAABBBBAAABA-AAAB-B-BBABBABAA-BAAB-AAABBBBBAA-AABAAAAAABBBAAABAAAA

*007024_00000260 ABAAAAABAABAAABBAAA--BB-BABAABBBBBA-ABAAABBBBBBAABABA-BAAAAAAABB-AABABB-BABABBBB-AAB-AABB

*007039_00008528 ABBABAABAAAAAA-ABAABBAA--ABABBBBBBBBABBBAB-AAAABBA-BBAAAAABAAABBBABAAAB-A-BAAABBBAAAABBBB

*007041_00011868 BBAA---A-AAABBAABBABABB-BABBBA-AAAABBBABABBA-BAAABBBBBAB-BBAA-A-BBABB-BA-AAAAABAABBB--BBB

*007050_00002931 B-BABBBAABAAAAAB-AAB-AAAB-AABAAABB---A-BBBABABAAABABAA-BBABB-A-AAABBBBBABBBAA-BABAABBAABB

*007079_00011883 A-BABA--BBBABBBBBAAAB-AAABAAAAAAB-B-ABBBBAAAB-BAAAAAAABBB-BABBAAAABBABBAAAAABABBABB-A--AA

*007148_00007082 -BB-BAAAB-BABBBBABAABBAABAABBBA-BAB-BBB-AA-BBBBBAAA--ABBBABA-BAABABA-B-AABA-BABABAA-ABBA-

*007160_00000954 BBAAABAAAAAAABBAAAAABBAABA-BBAA-ABAAAABBBABBABBBAAAABB-AABBBBBBBBABABAABAAAB-BABBBBA--AAB

*007279_00006847 A-BBB-B-AABBBA-AABAA-BABAA-ABB-A-BB-BBBAAABA-B-BBA-AB-AB-AABAA-BABB-ABBBBA--BB-BAB--AAAAA

*007290_00002629 BABBBBAAAAAB-AAAB--BAABB-BBAAAABBABBBABAABA-A-AAABAA-BAABAAAABAAAABABAABBAABBBBBABBA-BAAA

*007302_00008208 -BAAABAABABB-BBAA-AA-AAAAABAAAAABB-BBBABBBBAAAABAABAABAAAABABBABB-A-AAABABAABAABBBBABABAA

*007326_00005211 BABABBABAAAAABABBA-BBBAB-ABAABBAABABBBABABBABBAABAAAABBBBABAA-BAABBBABAB-ABBABB-AAABBAAB-

*007343_00001843 BAABABABAA-ABAABBBA--AAABAABBABBBA-BABBAAA-AB-ABBBABAABAABBAAABA-ABABBAABABAAA-ABAAABABBB

*007344_00025846 BBBABBAAABABA-BBBAABAAABB--ABABBABBBBBBB---BAB-AABA-ABABB-AA-AAA-AAAAABAAABABBAA-A-A-A-AA

*007385_00000813 BBABABBBABB-BABAABBBAABAAAABBBB---BAABBBAB-BBBB-BBBBA-ABABBBB-ABABAAAB-ABABBBAAABBBBBBABB

*007445_00002323 B-BABBBBABBAAA-B-ABAABAABBBBBB-BAA-BBB-BAA-AABAB-AABABAA-BABAAAB---B-A-ABBABBBAABB-AAABBB

*007469_00010082 BAAAABBAAAB-BAABABAABAA-BABBAABBBBBBB-A--BBB-B-AAAABAAABBAABBBBBABABBBAABBBBABBABAABBABBB

*007552_00023975 BAAAABAAAABAAABBAABABBABBBBAAABBABABB-BAABBBBBBAABABBAAAABAABABA-AABABABABBAB-ABAAABBABBB

*007564_00001213 ABB-BABBABABBAABBBBBBAB-ABBBBBA-AAAA-BB-AAAABABBAABBBAB-ABBABABAB--BBAA-AAAABA-ABBB-AABBB

*007617_00013580 BBAAABAABAAAB-A-BBABABBAB-BBBAAAAAA--BABAB-A--AAABB--BABA-BAABA-B-A-BABAAAAAAABAA--BAA-B-

*007640_00021983 AAAAAABBBAAAB-AABBBBBBBBB-BBBAAA-A-B-BABAB-A-B--BB-BBBABB-BA-BABBB-BBBBABAA-AABAABBAA-B-B

*007650_00007513 BBB-BBABBABABAABABAAAABABABBBAABBBBBBBABABBABBAAABBBABAAABBABBA-BBAABBAAAAAAAAAAABBBBABBB

*007682_00000935 BBBABBAAABABAABBBAABAAABBA-ABABBBBBBBBBAAAABA-BAABA-ABABBAAAAAAAABAAAABAABBAABAABAABBABAA

*007692_00000631 BAA-ABBAABBBBAABABAAAAABBA-BAABB-BABB-BAAB-BABAAAAABA-A-BAAABBABAAAB-BABA-BBBBBBBAA---BBB

*007699_00008452 B-ABABABAABBBABBBBBABBAAAABAAB-ABBBBBABBBBAB--BBA-AB-AABAAAB-ABAA-BBAAAABAAAABBAAAAABBBBB

*007730_00001598 BBBABBAAAAABBBBBABB-B-AAA-BBBABAAAAAAAAABAAAB-BBBB-AAABB-AABBABBB-ABA-BABB-BB-BBABBABAAAA

*007733_00002128 BAAAABBBBAB-AABABAABBAAA--BB-BAAAAAAA--BBB-AB---A-AA--ABBB-B--B-ABAAAAAB-AA-AABB-B--BAAAA

*007845_00003956 BABABBAAAAABB-BBABBBBAAAABBBBABAAAAAABA-AAAABBBBBB-AAABB-A-BBABBBBABBBBBABBBAABBABBAAAAAA

*007853_00001226 B-BABBBBBABBAABBBAABBBBBABBAB-BAABA-ABAABB-AAAABBAAAA-BBBAA-BABABBAA-BBBBAB-AAB--B-A---AA

*007853_00001604 BABABBBBBABBAABBBAABBBBBABBABA-AABAAABAABBAAAAABBAAAAABBBAA-BABABBAAABBBBABBAA-BBBBAABBAA

*007856_00006769 BABBBBBBBAB-AABA-A-BBA-ABBBBB-AAAA--AAABBB-AB-BAABAAABABBB-B-BBBB-AA-AA---ABAABBB-AA-A-AA

*007905_00005206 BAAAABBABBAABBAAABBBBBBBBAB-BAABBB-BBABBBA--BBA-BBAABAABBABBBBBAAAABBBAABBABBBBAABBBBBAAA

*007910_00002447 ABABAAABABBBABBA-AABBAAB-BBBBABBABAAAAABAABBBAAAABBABABAAAABAABBABB-BAABABBBBAABBBBAAAAAA

*007914_00009662 BBBA-BABAABAABBBBAABABAA-BABBBB-BBBAAABAAA-BB-A-BBBBBAABBBAABAABBBAAAABAABABA-AABBBBBAA-A

*007918_00022685 --AAAAABAABAAABBAAAABABBBABAAB--BBAAABAA---AB-BAAAA-ABBAAAAAA-BB-A-BAB-BBABAB--BAAAB--BBB

*007919_00003853 ABAAA-ABAABABABBAB-AAABAA-BABAB-BBB-BB-BBBB-ABAAABABBBAAAABAABABBAA-BBABBABBBAABABBBBABBB

*007937_00001635 --BBBABABBBBABABBAAAB-BAAABAABABBBB-BABBAA-B-ABBBAAABABBABBB-ABBBBBAB-AABAAA-BABBBBBAAA-A

*007937_00005831 -ABBB-BAB-B-ABA-B-A--ABAA-BA-BABBBB--AB--A-BAABBBAAAB-BBA-BB-ABBB-BABAAABAAAABABBB-BAAA-A

*008093_00001408 B-AAAB-BBABBBBAA-BBAAABAAABAAAABBABBBABBBBA-BA-AAA--AB-BABBAA-AAA-ABAAAAAAABB-AABBBBBAAAA

*008107_00007994 BABBBBABABBBBAABABBAABBAAAABABBBAB-AABBA-BABBAABBBBAABBBBBABABABBBBAAABABBBBAAAABBBBBBAAA

*008163_00001931 ABABAAABABBBABBAAAABBAAB-BABBABBABAAAABBAABBBAABABAABABAABABAABBABB-BABBABBBBAABBBBA-AAAA

*008222_00011433 B-BBBBABA-BBBABA-BABAAAABBBBBABAAAAAABAA-AABBABBBABAAABBBAB-BAAAAB-BB-BAAAABAA-AABBBAB-AA

*008224_00002299 AAABAAAABABAABAABAA-BABBBBAABBABBA-AAABBBBABBAABABAABBBAAABBA-ABBAABBAABBAAAAAABBBBAAA-AA

*008334_00022163 B-BABBAAABAB-ABBBAABAAABBA-ABAABAB-BBBBB-A-BAB--ABA---ABBABAAAAAAA-A-ABA-ABABBAABAAAA-AAA

*008460_00007836 B-BBBBA-AB-BAAABBABBBAAA-ABABBAAA-AAABA-ABBAA-AAAAABA-BBBAB--BB-ABAAABB-BABAABABAAABABBBB

*008584_00008844 BBAAABBBAAAABAABBBAAAABAAABBABBBABAA-BABABBBAABAAAAABBAABABA-AABBAAB-BBABBBBA-ABBAAABABAA

*008584_00008930 BBAAABBBAAAABAABBBAA-ABAAABBABBBABAA-BAB-BBBAABAAAAAB-AA-ABAAAABBAABABBABBBBA--BBAAA-ABAA

*008660_00003497 AAABAAABA-AB--AAA-BABAAABBAAABBBAABBBABAB-B-B-BB-BBABBABABBAABBABBAB-B-BABBBBABBAAAAAAAAA

*008711_00001162 BABABBAB-AAA-B-B-ABBBB-BB--AABB-ABABBBABAB-ABBAA--AA-BBBB-BAABB-ABBBABAB-ABBABB---ABB--B-

*008760_00007425 B-ABABAABBABA--BB-AAAAAAABABBA-B-A-BBAABBABAAABBABBAAAABAAB-ABA-BBBBBAABAABBAA-BB-AAAABAA

*008857_00022988 AAAAAAABAAB--ABBAAAABABBB-BAABBBBBAAABAABBBABBBAAAABABBAAAAAAABB-AABABBBBABABBBBAA-BB-BBB

*008964_00007175 AABABAABAA-BABBAAAA-BBBBABABBAAAAB-BBABBBA-BBBBBABBA-BBABABABABA-AABB-AAABABBBB-BBBBABAAA

*009035_00000963 B--BAB-AABBAAAABB---BABAAAB-AAB--AABBBBA-ABAAAAA-BBA--AB--A-B-A-A--A-BBAB-BBBAB-BBBB--BAA

*009066_00007413 B-B-BB-B-AABAAB-BAABBB-BABBA-A--A-A-A--ABBAAA--BBA-AAABB-AABBABABBAAAAB-BABBAAA--BBA---AA

*009221_00003642 A-BBBAAAABBAABBABAA-BBABABABABAAABA-AABBAABBA-BBBAAABAABABBBABBBABBAAABBBBAAABABBAAABBAAA

*009260_00003173 BBAAABABAAABBBBA-BABBBABBAAABAAA-B-AABAA-BBBB--AAAAAAAAAB-B-BABAABABABABBAAAAABBAAABBABAA

*009314_00008834 B-BBBBABABABBAABBBBBBAAABBAABBAAABABBBAB-B-AABAA-A-BAABBABAAABBBBBBABABBBBBABBAB---BAAABB

*009349_00003107 BABABBBBBAABAABBBAABBBBBABBABABAA-AAABAABBAAA-A-BAAAAABBBAA-BABABBAAAABBBABBAAA-BBBAABBAA

*009349_00005062 B-BABBBBBAA-AABBBAABBBBBABBABA-AAAAAABAABBAAA--BBAAAAABBBAA-BABABBAAAA-BBABB-AA-BB-AA-BAA

*009350_00002031 AAABAAAABABAABAABAA-BABBBBAABBAABABAAABBABABBAABABAABBBAAABBABABBAABBAA-BAAAA-ABBBBAAA-AA

*009381_00000324 ABABAABBBABABABBBB-AABBBBAABBBBBAAABBBBAAABB-BABAAAABBAABBAABBABAABABBBAB-BABABABAABBABAA

*009475_00004544 AB-AAABBBBBAA--BABAA-AABAB-AAABBAAA-A-B-BA-ABBB-A-AAAB--B--A-BAABABA-B-AAA--BABABBBAA--AA

*009805_00001732 BAAA--AA--BA-A-B-ABA-AABB-B-AA--ABABB-BAAB-BB-BAABAB-AAA--AA-ABBAAABABABABBAB-A-ABBBB-BBB

*009826_00013562 BB-ABB-A-BA-AAB-BAABAAAB---ABA----B-BB--BA-BABAAABA-ABAB-ABAAAAAAAAAAABAAABABBAABAAA-AAAA

*010166_00020959 BA-B-B---BBAAA-BBAAABABA---AAABAAA--B-BA-A-AAAAAABBAA--BA-AABAAAA-AAABB-BABBBA-ABBBB--BAA

*010223_00000859 BABBBBAAAAAB-AAAB--BA-BB-BBAAAABBABBBA-AABA-AAAAABAA-BAABAAAABAAAABABA-BBAABBBBBABBABB-AA

*010239_00000974 B--ABB-BBBBBB-BB-BBAAB-BB-BB-A-ABBBBBABAABAABABAB-A----B-B-AABABAAABBBABAAABBABBABBBBABBB

*010335_00000372 BBBABBAAABABBABBBBABAAABAA-ABABBBBBAABBAAAABA-AAABAAABABBAAAAAABABAAAABAABBAABAABAABBABAA

*010338_00003088 AABB-AABAABAABB-BAAAAAAAABBAAAABAAAAABBABB-AAAABABABBBABBAAA-BAABAABBAAABAABBBBAABBBAAAB-

*010379_00005749 AABBBAABAAABBBAAAB-BBBBBAABAAABBABABBBABBBAAAAA-AAAABB-BBABAAABAB-BB-BAB-ABBABBBBAAABBAAA

*010495_00005478 BBBABBAAABABAABBBAABAAABBA-ABAABABBBBBBBBAABABA-ABA-ABABBAAAAAAAAAAAAABAAABABBAABAAAAAAAA

*010603_00005197 BBBABBAB-AAABB-ABBBABBBB-A-AAABBBBBABABBA-AB--AABBAABBBBBBBABAB-BAABABABAABAAABBB---BBAAA

*010609_00002438 BBABABAB-ABBBBBB-BB-BBAAAA-ABB-ABABAAAAAB-ABABAAABAB-AABBBABAABABBAAABAABABAABBAABBAABBBB

*010687_00001438 BAABABA-AABB-BBBB-BABBAA-AB-AB---BB-BABBB--BA-BB-BA-BA-BAAAB-ABAABBBAAAABAB-A-BA-AA-BBB-B

*010783_00001596 AAA-AAA-AAB-AABBA--ABABBBABAAB--BBA-ABAABBBABBBAAAABABB--AAAAA-B-A-BABBBBABABBBB-AA-B-BBB

*010846_00004034 AAAAAAABBABAABBBBAABABBABB-BBBABBBBAABBA-BAAAAAABBBBBABBBBBA-AABBBABAB-AABBBAABABBBBBAABB

*010935_00000953 BABBBBAABA-BB-BABBAAA-BBBABBBAABAAA-BABAABAAAA-AABAA-AAABBAABBAAABBABAB-BBABBBBBABBA-BAAA

*010944_00002208 BBAA-BBBAABABBBBABBABBBABABABBBAAA-BBAAABBABBABABA-BBABBABBBAABABABABBBBBABAAAB-ABBAABABB

*010952_00000823 BABABBBBBABA-AB-B-AAABBABAAAABBBBABAABBBABBAAABBAA-ABABAAAAABAABAAA-BAABBBBAAAABBBBBBBBAA

*010955_00001385 BBBABBB-ABABBBABABBABAA-ABBAABBABABAABBB-BABBAABBAAAABABAABBBBBA-BAABB-ABA-AB-BAAAABB-BAA

*011064_00000439 BAB--BBBBAB-AABABABBBAAABBBBBB-AAAAAAAABBB-ABBBAAB-AABABBBBBABBBB-AA-AABA---AABBBAAA--A-A

*011087_00000602 ABBABABAABBAB-BBBBBAABABAAAABBB-AAABBBBBAAAAABABAAABAAAABBABABABBABAABB-BBAABBBAB-B-BBBBB

*011143_00000051 BABABBBBBAABBBAABBBBAABBAABABAABBABAAAABABAAB-AAA-ABBBBABAAAA-AAAAAABAAABAABBBBABBBAABA--

*011517_00002321 BABABBBBABBAAA-B-ABAABAABBBBBB--AAABBBBBABAAA-AB-BABABAAABABAAABB-BBAAAABBABBBAABBBAAABBB

*011547_00001005 B-ABAB-BBAABAABABABABAABAABBBBAAABA-AAABAB-BBABBABAAABAAB-AAABBBBBAA-AABAAAAAABB-AAABA-AA

*011705_00000515 B-AAABABAAAABABBABAA-ABAAABBABBAABAA-BABABBBABBBAAAABBAABAAAAAABBBABBBBABBBBABAABAAA-BAAA

*011802_00001592 AABABAAB-AABBAAAABBBBBBBBAAABABBAB-BBBABBBAB-AAAAAAABAABBABAAABABABB-BAB-ABBBB-BBAAAABAAA

*011981_00000147 BABABBBBBAABAABBBAABBBBBABBABABAA-AAABAABBAAA-ABBAAAAAB-BAA-BABABBAAAABBBABBAAA-BBBAABBAA

*012064_00001310 AAAAAA--BAAABBA-BBBBBBBBB-BBBABA-A-BBBABABBA-BAABBBBBBABBBBAABABBBBBBBBABA-AAABAABBAAA-BB

*012460_00001709 BBAAABBAABBBBAABABAAAAABBA-BAABB-B-BB-BAABBB-B-AAAABAAABBAAABBABAAABBBABAABBBBBBBAABB-BBB

*013529_00001017 A-BABAABAAABBAA--B-BBBB-BA--BAB-AB-B--A-B--B-AA-A-AA-AA-BABAAABAB--BBBBB-ABBB-BBB-A-ABAAA

*014034_00001102 BBB-BBAAAAABBBBBABBBB-AAABBBBABAAAA--AA-B-AABBBBBB-ABABBAA-BBABBBB-B-BBABB-BBBBB-B-ABA-AA

*014526_00000184 BABABBBAABBABAABBBBAABAABABABBB-AAABBBBBAAAAABABABABABAAABAB-BABBAB-AABBBBABBBAABBB-BABBB

*015131_00000615 B-BABBBBAAAAA-BAAAA-BBBBBB-ABBAABB-AAA-BBBAABA-BBAAAAABA-AABAAB-A-BA-AAAAABBABBBAAABBA-AA

*015215_00000473 B-BBBBA-ABAABABB-BA--AB-BABBBABBBBBBABAA-ABBBABABBAAAABABBBAAAAAA-AAABAABBBAAAAABBBBBABBB
